# Halogen Atom Participation in Guiding the Stereochemical Outcomes of Acetal Substitution Reactions

**DOI:** 10.1002/anie.202209401

**Published:** 2022-09-16

**Authors:** Krystyna M. Demkiw, Wouter A. Remmerswaal, Thomas Hansen, Gijsbert A. van der Marel, Jeroen D. C. Codée, K. A. Woerpel

**Affiliations:** ^1^ Department of Chemistry New York University 100 Washington Square East New York NY 10003 USA; ^2^ Leiden Institute of Chemistry Leiden University Einsteinweg 55 2300 RA Leiden The Netherlands

**Keywords:** Diastereoselectivity, Glycosylation, Halonium Ion, Hyperconjugation, Oxocarbenium Ion

## Abstract

Acetal substitution reactions of α‐halogenated five‐ and six‐membered rings can be highly stereoselective. Erosion of stereoselectivity occurs as nucleophilicity increases, which is consistent with additions to a halogen‐stabilized oxocarbenium ion, not a three‐membered‐ring halonium ion. Computational investigations confirmed that the open‐form oxocarbenium ions are the reactive intermediates involved. Kinetic studies suggest that hyperconjugative effects and through‐space electrostatic interactions can both contribute to the stabilization of halogen‐substituted oxocarbenium ions.

## Graphical abstract

Nucleophilic additions to α‐haloacetals proceed via an oxocarbenium ion intermediate where the carbon‐halogen bond can either adopt a pseudo‐axial or a pseudo‐equatorial position. *trans*‐Selectivity is typically observed when a halogen atom is positioned at C‐2 of an acetal because hyperconjugative interactions involving the pseudo‐axial carbon‐halogen bond stabilize the oxocarbenium ion intermediate.

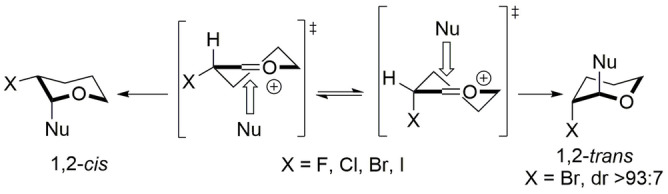

## Introduction

Considerable attention has been directed towards the synthesis of 2‐deoxysugars because this structural motif is found in many biologically active compounds.^[1]^ Glycosylation reactions with these sugars, however, often proceed with low stereoselectivity.^[2]^ The challenge of controlling stereochemistry has been addressed by installing participating groups on the 2‐deoxyglycosyl donor, such as halogen atoms, to control the stereochemical outcome, followed by removal of the participating group.^[3]^ Because halogenated glycosyl donors are usually multiply substituted,^[4]^ the influence of the C‐2 halogen atom on selectivity cannot be evaluated directly, which can make it difficult to rationalize the diastereoselectivities of glycosylation reactions.[^4a^, ^5^]

We here demonstrate the strong influence of a single α‐halogen atom on the stereochemical outcome of acetal substitution reactions of furan‐ and pyran‐derived acetals. The stereoselectivities observed using nucleophiles of varying reactivities are consistent with the intermediacy of open‐form oxocarbenium ions as reactive intermediates and not with S_N_2‐type substitution reactions of three‐membered‐ring onium ions. This interpretation is supported by computational and kinetic studies.

## Results and Discussion

Substitution reactions of acetal **1** with carbon nucleophiles revealed that the presence of a single halogen atom at C‐2 can lead to highly stereoselective substitution reactions (Table 1). The preference for the 1,2‐*trans* products upon substitutions with allyltrimethylsilane, a reagent that reacts irreversibly with carbocations,^[6]^ increased along the series F<Cl<Br≈I, with the substitutions of the fluorinated pyran favoring the 1,2‐*cis* product.^[7]^ While the reactions of the acetals bearing a bromine or iodine atom furnished a single stereoisomer (entries 3 and 4), reactions with 2‐chloropyran **1** **b** proceeded with lower selectivity (entry 2). By contrast, the presence of a fluorine atom in 2‐fluoropyran **1** **a** resulted in highly stereoselective formation of the 1,2‐*cis* isomer (entry 1). A trichloroacetimidate leaving group was used as the leaving group for 2‐fluoropyran **1** **a** because an acetate leaving group would not ionize with an electron‐withdrawing fluorine atom at C‐2.^[8]^ This choice of leaving group is unlikely to affect diastereoselectivity, however, considering that, with the other acetals, selectivities did not depend upon the leaving group (entries 2, 5, and 6).^[9]^ Allylations at higher temperatures (entries 7 and 8) remained diastereoselective for the chlorinated and brominated pyrans. The influence on stereoselectivity of a halogen atom at C‐2 contrasts with the inability of a methyl group at C‐2 to control diastereoselectivity (entry 9).^[10]^

**Table 1:** Allylations of halopyran **1**.^[a]^

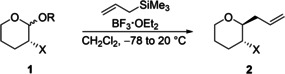					
					
Entry	Compound	X	R	*trans* : *cis*	Yield [%]^[b]^
1	**a**	F	CNHCCl_3_	4 : 96	25
2	**b**	Cl	Ac	86 : 14	66
3	**c**	Br	Ac	>99 : 1	77
4	**d**	I	Ac	>99 : 1	66
5	**e**	Cl	Me	84 : 16	64^[c]^
6	**f**	Cl	CNHCCl_3_	78 : 22	59
7^[d]^	**b**	Cl	Ac	82 : 18	74
8^[d]^	**c**	Br	Ac	>99 : 1	88
9^[e]^	**g**	Me	Ac	48 : 52	57

[a] Diastereomeric ratios were determined by ^13^C{^1^H} NMR^[11]^ and ^1^H NMR spectroscopy. [b] Isolated yield. [c] Conversion. [d] Reaction conducted at 20 °C. [e] From Ref. [10].

The diastereoselectivities of acetal substitution reactions depended upon the reactivity of the nucleophile. As illustrated for the chlorine‐ and bromine‐substituted acetals **5a** and **6**, substitution with ethanol, which is more nucleophilic than allyltrimethylsilane,^[12]^ occurred with generally lower selectivity (Table 2). As the nucleophilicity of the alcohols decreased upon incorporation of electron‐withdrawing atoms,[^12b^, ^13^] as measured by field inductive effect parameters (*F*),^[14]^ selectivity increased to match the outcome observed for allyltrimethylsilane (Table 1, entries 2 and 3).^[15]^ Additions of weaker nucleophiles became increasingly selective for the *trans*‐isomer with yields ranging from 38–72 % over two steps from hemiacetals **3** and **4**. The reactions with hexafluoroisopropanol were highly diastereoselective but low‐yielding (8–21 %) due to the poor nucleophilicity of the alcohol.[^14a^, ^16^] These results reflect those of the additions of hexafluoroisopropanol to carbenium ions that often proceed with low conversion.^[17]^ In all cases, the substitution reactions were confirmed to be under kinetic control.^[9a]^ Because the nucleophilicities of carbohydrate‐derived secondary alcohols are similar to the nucleophilicities of 2,2‐difluoroethanol and 2,2,2‐trifluoroethanol,^[18]^ the results in Table 2 suggest that installation of a halogen atom at C‐2 could be more generally useful for controlling the stereoselectivities of glycosylation reactions.

**Table 2:** Substitutions of halopyrans **5a** and **6** with *O*‐nucleophiles.^[a]^

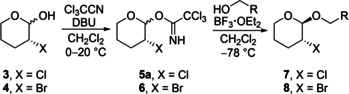					
					
Entry	Compound **7** or **8**	Nucleophile	*F* Number	*trans* : cis X=Cl	*trans* : *cis* X=Br
1	**7** **a**/**8** **a**	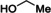	0.00	84 : 16	93 : 7
					
2	**7** **b**/**8** **b**	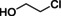	0.13	86 : 14	98 : 2
					
3	**7** **c**/**8** **c**	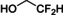	0.29	92 : 8	>99 : 1
					
4	**7** **d**/**8** **d**	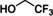	0.38	94 : 6	>99 : 1
					
5	**7** **e**/**8** **e**	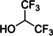	>0.38	>99 : 1	>99 : 1

[a] Diastereomeric ratios were determined by ^13^C{^1^H} NMR^[11]^ and ^1^H NMR spectroscopy.

As illustrated for ethanol as the nucleophile, the selectivities did not depend strongly on parameters such as the leaving group (Table 3).^[19]^ Activation of the imidate leaving group with BF_3_ ⋅ OEt_2_ or Me_3_SiOTf gave similar results (entries 1–4), which suggests that triflate intermediates are likely not responsible for the stereoselectivity.^[20]^ The diastereoselectivities increased as solvent polarity decreased (entries 1, 5, and 6). The similar stereochemical outcome in the polar solvent acetonitrile does not support a mechanism where displacement of acetonitrile from a nitrilium ion occurs in the stereochemistry‐determining step (entries 6 and 7).^[21]^

**Table 3:** Influence of conditions on stereoselectivity of reactions with *O*‐nucleophiles.^[a]^

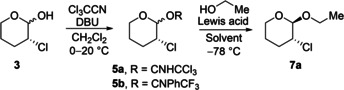				
				
Entry	Leaving Group (R)^[b]^	Solvent	Lewis Acid	*trans* : *cis*
1	CNHCCl_3_	CH_2_Cl_2_	BF_3_ ⋅ OEt_2_	86 : 14
2	CNHCCl_3_	CH_2_Cl_2_	Me_3_SiOTf	86 : 14
3	CNPhCF_3_	CH_2_Cl_2_	BF_3_ ⋅ OEt_2_	85 : 15
4	CNPhCF_3_	CH_2_Cl_2_	Me_3_SiOTf	86 : 14
5	CNHCCl_3_	PhMe	BF_3_ ⋅ OEt_2_	91 : 9
6	CNHCCl_3_	MeCN	BF_3_ ⋅ OEt_2_	66 : 34
7	CNPhCF_3_	MeCN	Me_3_SiOTf	66 : 34

[a] Diastereomeric ratios were determined by ^13^C{^1^H} NMR^[11]^ and ^1^H NMR spectroscopy. [b] The trifluoroacetimidate leaving group was installed using 2,2,2‐trifluoro‐*N*‐phenylacetimidoyl chloride and Cs_2_CO_3_.

The stereochemical outcomes of substitutions of fluorinated pyran **1** **a** with oxygen nucleophiles showed a different pattern (Table 4). Substitutions initiated with Me_3_SiOTf using strong *O*‐nucleophiles occurred with moderate *trans*‐selectivity, while the addition of a weak nucleophile, hexafluoroisopropanol, was low‐yielding and unselective.^[22]^ The stereochemistry of the substitution reactions differed from the reaction using allyltrimethylsilane (Table 1, entry 1). Slight differences in stereoselectivities were observed using BF_3_ ⋅ OEt_2_ compared to Me_3_SiOTf, but not enough to justify that a covalent triflate[^20^, ^23^] was responsible for the selectivities in those reactions. The different behavior of the fluorine‐substituted acetal **1** **a** compared to the chlorine‐ and bromine‐substituted acetals **5a** and **6** reflects the relative abilities of halogen atoms to stabilize nearby carbocations.^[24]^

**Table 4:** Substitutions of fluoropyran **1a** with *O*‐nucleophiles.^[a]^

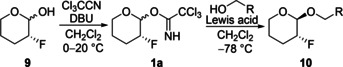					
					
Entry	Compound	Nucleophile	*F* Number	*trans* : *cis* (BF_3_ ⋅ OEt_2_)	*trans* : *cis* (Me_3_SiOTf)
1	**10** **a**	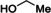	0.00	77 : 23	81 : 19
					
2	**10** **b**	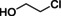	0.13	65 : 35	75 : 25
					
3	**10** **c**	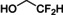	0.29	67 : 33	76 : 24
					
4	**10** **d**	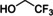	0.38	61 : 39	76 : 24
					
5	**10** **e**	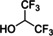	>0.38	43 : 57	41 : 59

[a] Diastereomeric ratios were determined by ^13^C{^1^H} NMR^[11]^ and ^1^H NMR spectroscopy.

**Scheme 1:**
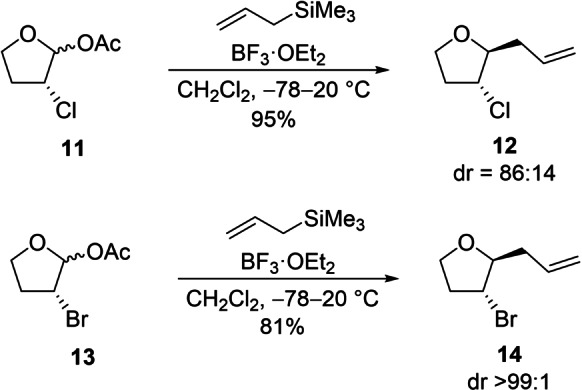
Substitutions of halofurans **11** and **13** with allyltrimethylsilane.

Substitutions of halogenated five‐membered‐ring acetals revealed similar trends between nucleophilicity and diastereoselectivity to those of six‐membered‐ring acetals.^[25]^ Stereoselectivities of substitutions of the furan acetals with allyltrimethylsilane are nearly identical to those observed for the pyran acetals (Scheme 1). Differences in selectivities between the C‐2‐Cl and C‐2‐Br substrates were observed with oxygen nucleophiles, however: additions of strongly nucleophilic alcohols to chlorinated acetal **17** were unselective, whereas additions to brominated acetal **18** were *trans*‐selective (Table 5). Decreasing the nucleophilicity of the alcohol restored the high 1,2‐*trans* selectivity (entries 3–5).^[22]^

**Table 5:** Substitutions of halofurans **17** and **18** with *O*‐nucleophiles.^[a]^

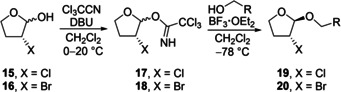					
					
Entry	Compound **19** or **20**	Nucleophile	*F* Number	*trans* : *cis* X=Cl	*trans* : *cis* X=Br
1	**19** **a**/**20** **a**	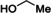	0.00	59 : 41	89 : 11
					
2	**19** **b**/**20** **b**	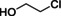	0.13	64 : 36	96 : 4
					
3	**19** **c**/**20** **c**	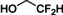	0.29	88 : 12	>99 : 1
					
4	**19** **d**/**20** **d**	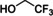	0.38	88 : 12	>99 : 1
					
5	**19** **e**/**20** **e**	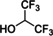	>0.38	>99 : 1	>99 : 1

[a] Diastereomeric ratios were determined by ^13^C{^1^H} NMR^[11]^ and ^1^H NMR spectroscopy.

**Figure 1:**
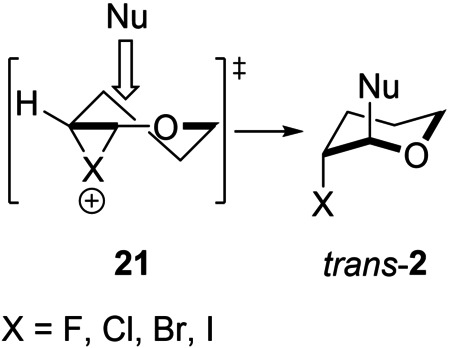
Nucleophilic additions to halonium ion intermediates.

**Figure 2:**
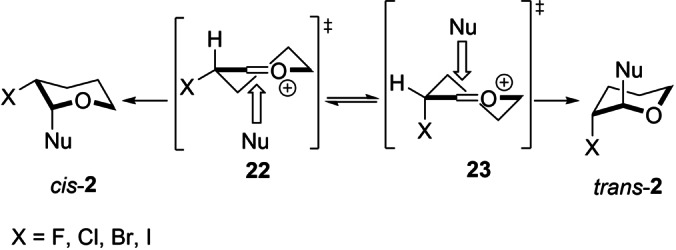
Nucleophilic additions to α‐halogenated oxocarbenium ion intermediates.

To probe the origin of stereoselection for the highly stereoselective reactions, we considered possible reactive intermediates. Reactions through three‐membered‐ring halonium ion intermediates, which are often invoked to explain the role of halogen atoms,^[26]^ are inconsistent with the selectivity patterns. Had the reactions occurred by nucleophilic ring‐opening of halonium ion **21**, the *trans*‐isomer would be the sole product (Figure 1). In contrast to the experimental results, selectivity would increase with increased nucleophilicity, considering that a stabilized halonium ion could be opened more readily with stronger nucleophiles, but less reactive nucleophiles would need to react with an open‐form intermediate (i.e., an oxocarbenium ion, Figure 2). This reaction with the oxocarbenium ion would occur from the face opposite to the axial halogen atom.^[27]^ Computational studies also indicated that cyclic chloronium and bromonium ions are unlikely to be reactive intermediates; these cyclic ions were never found to be energetic minima.

To understand the role of oxocarbenium ion intermediates in the observed stereoselectivities, we applied the conformational energy landscape (CEL) mapping method, in which the energy of the ions are computed as a function of their shape.^[28]^ A single‐point benchmark study for comparison to computed CCSD(T) energies was performed on the lowest pseudo‐axial and pseudo‐equatorial conformers of each pyranyl and furanyl cation.^[29]^ The recently developed revDSD‐PBEP86‐D4 double‐hybrid functional was used as the optimal level of theory because it performed best of all density functional theory methods at a fraction of the cost of wavefunction‐based methods.^[30]^ All computations were performed using ORCA5.0.3.^[31]^

**Figure 3:**
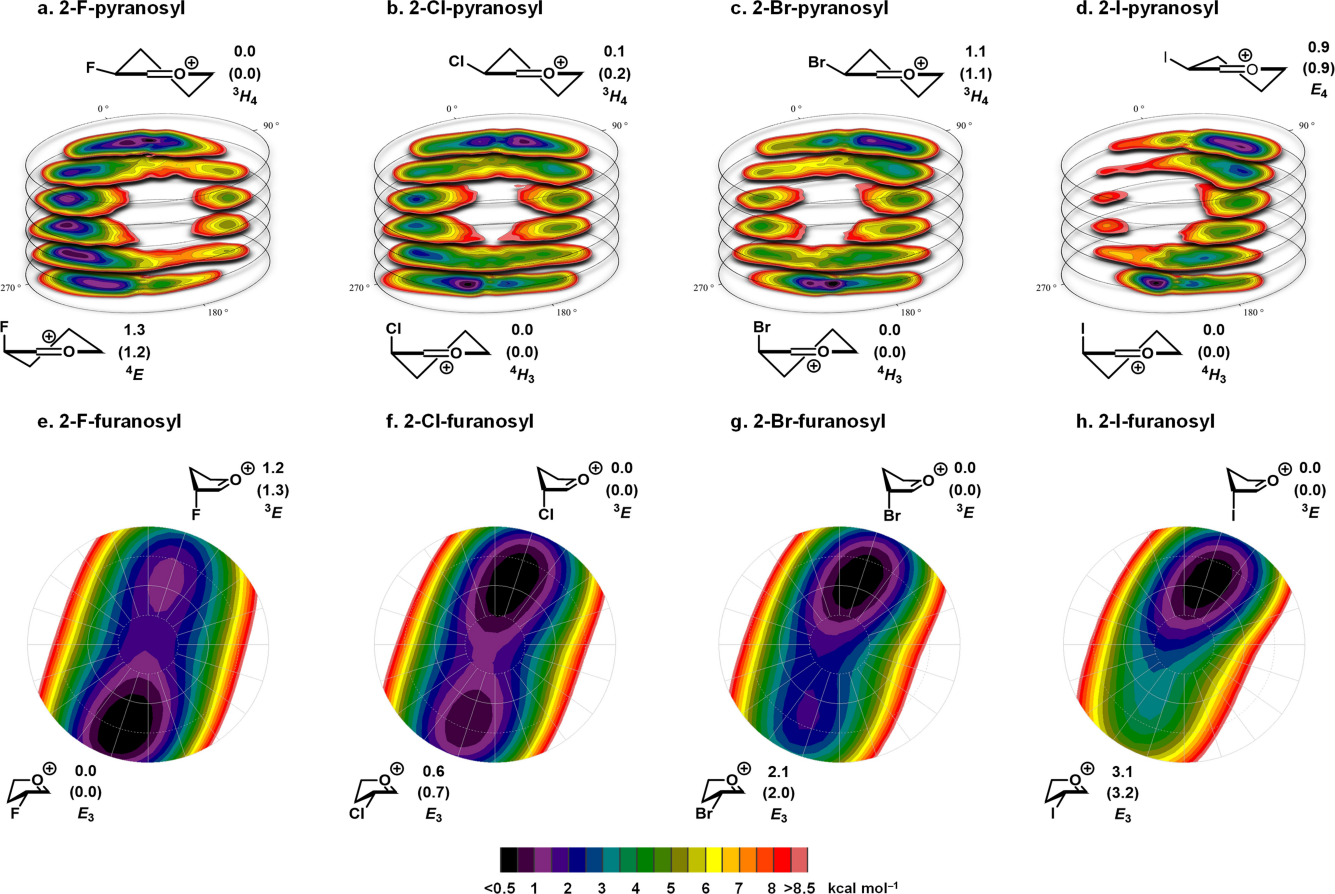
Conformational energy landscape (CEL) maps of halopyranyl and ‐furanyl oxocarbenium ions in which the local minima identified are shown with their respective energy. Energies of all conformations in the CEL are computed at SMD(dichloromethane)‐revDSD‐PBEP86‐D4‐DKH‐def2TZVPP//PCM(dichloromethane)‐B3LYP‐D3BJ‐DKH‐def2TZVP and expressed as Gibbs free energy (*T*=195.15 K) in kcal mol^−1^, and between parentheses reference Gibbs free energies (*T*=195.15 K) for that specific conformation are given in SMD(dichloromethane)‐CCSD(T)‐DKH‐def2TZVPP//PCM(dichloromethane)‐B3LYP‐D3BJ‐DKH‐def2TZVP. Oxocarbenium ion CEL maps for: a) 2‐F‐pyranyl, b) 2‐Cl‐pyranyl, c) 2‐Br‐pyranyl, d) 2‐I‐pyranyl, e) 2‐F‐furanyl, f) 2‐Cl‐furanyl, g) 2‐Br‐furanyl, h) 2‐I‐furanyl.

The computed maps (Figure 3) provide a clear indication of the conformational preferences of oxocarbenium ion intermediates. For the six‐membered‐ring oxocarbenium ions, the preference for the half‐chair conformer, in which the C‐2‐halogen is pseudo‐axially oriented (i.e., the ^4^ *H*_3_ conformer),^[32]^ increases along the series F<Cl<Br≈I. The furanyl maps (Figure 3e‐h) reveal a similar trend, with the preference for the ^3^ *E* conformer increasing in the same order (F<Cl<Br<I). The pseudo‐axial preference for the larger halogens (Br and I) can be rationalized by a greater stabilization of the cationic center through donation of electron density from the σ_C‐X_ bond.^[33]^ This hyperconjugative stabilization correlates with the high 1,2‐*trans* selectivities (Tables 1, 2, 5) observed for reactions of the C‐2‐Br acetals, with the ^4^ *H*_3_ pyran oxocarbenium ion and the ^3^ *E* furan oxocarbenium ion, which would be attacked preferentially by a nucleophile from the bottom face through a chair‐like transition state.^[27]^ Attack on the ^3^ *E* furan oxocarbenium ion by a nucleophile is most favorable from the top face because this mode of attack minimizes developing eclipsing interactions in the transition state.^[6a]^ This mode of addition is stereoelectronically and sterically preferred, so it is difficult to deconvolute if steric interactions of the bromine atom contribute significantly to the stereoselectivity. By contrast, a fluorine atom adopts an equatorial orientation in the C‐2‐F substituted oxocarbenium ions, likely because σ_C‐H_ is a better electron donor than σ_C‐F_.^[34]^ In the case of the pyran acetal **1** **a**, addition would occur with *cis*‐selectivity through a chair‐like transition state,^[27]^ as observed experimentally with allyltrimethylsilane.

The conformational preferences for the chlorinated cations are more complex. Both the furanyl and pyranyl oxocarbenium ions show low preferences for the pseudo‐axial conformers (0.6 and 0.1 kcal mol^−1^, respectively). The preference for this conformer accounts for the observed *trans*‐selectivity found for reactions with allyltrimethylsilane and weaker *O*‐nucleophiles. With increasing nucleophilicity, the rates of addition may approach the diffusion rate limit,^[20]^ where nucleophilic addition can occur to both faces of the cationic carbon atom.^[35]^ The increasing *trans*‐selectivity observed in the reactions of the C‐2‐Cl pyran donor with decreasing nucleophilicity of the *O*‐nucleophiles may be influenced by developing steric interactions between the incoming nucleophile and the C‐2‐halogen atom when attack occurs on the ^4^ *H*_3_ ion. With decreasing nucleophilicity, the transition state will be more product‐like, increasing these destabilizing steric interactions and therefore leading to higher *trans*‐selectivity.

**Scheme 2:**
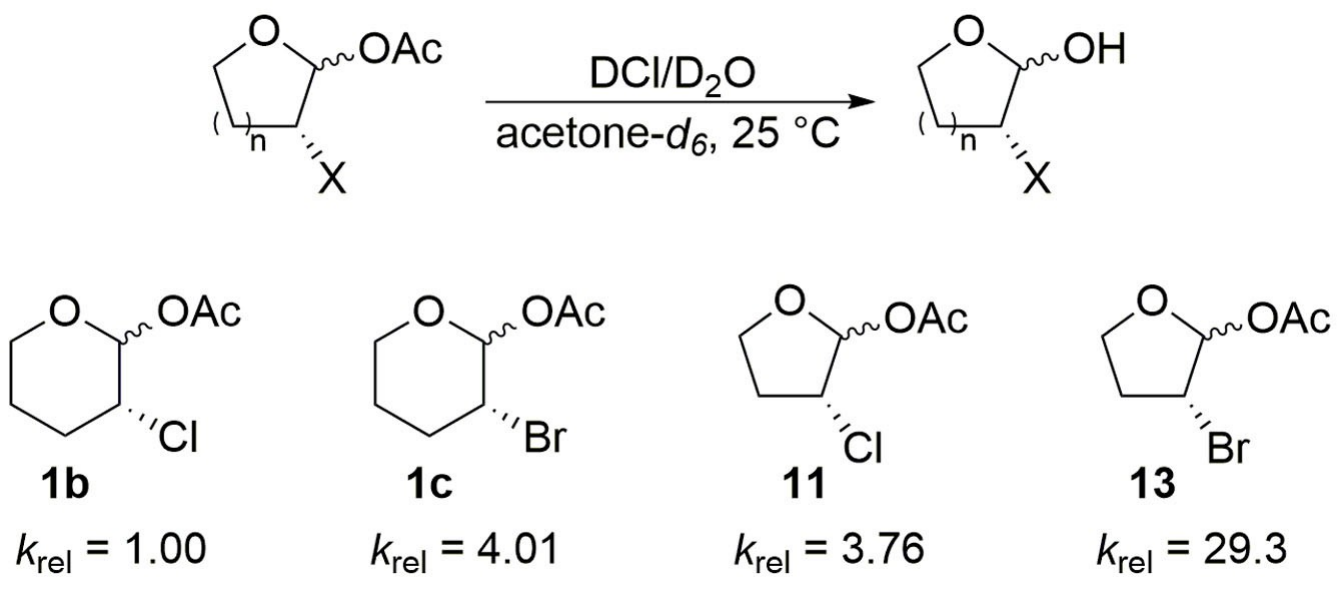
Rates of hydrolysis of haloacetals.

Kinetic studies of hydrolysis reactions provided insight into how the presence of a halogen atom influences the stabilization of cyclic oxocarbenium ion intermediates (Scheme 2). The rates of acetal hydrolysis reveal how the inductive effects exerted by a halogen atom, which should decelerate formation of an oxocarbenium ion,^[36]^ are compensated by hyperconjugative interactions, which should accelerate formation of oxocarbenium ions.^[37]^ The acid‐catalyzed hydrolysis reactions occurred more slowly than the substitution reactions using Lewis acids (e.g., Table 1), so the course of the reactions could be monitored by ^1^H NMR spectroscopy. Analysis of the rates of hydrolysis of mixtures of stereoisomers of the α‐haloacetals provided a relative assessment of the overall effect of the halogen atom at C‐2, allowing comparisons between different systems. These experiments showed that acetals bearing less electronegative bromine atoms underwent hydrolysis faster than acetals with chlorine atoms, consistent with observations for acyclic acetals.^[36]^ The corresponding fluorinated acetal, which did not ionize using Lewis acids, was not anticipated to ionize under the milder conditions shown in Scheme 2. The faster hydrolysis rates of the five‐membered rings compared to the six‐membered rings align with the generally higher reactivity of furan acetals over pyran acetals.[^22^, ^38^] The larger influence of halogen atoms in the five‐membered‐ring acetals compared to the six‐membered‐ring acetals is consistent with the computational studies (Figure 3) that show a generally larger preference for the axial conformers in five‐membered rings, likely because of better overlap between σ_C‐X_ and π*_C‐O_^+^.

**Scheme 3:**
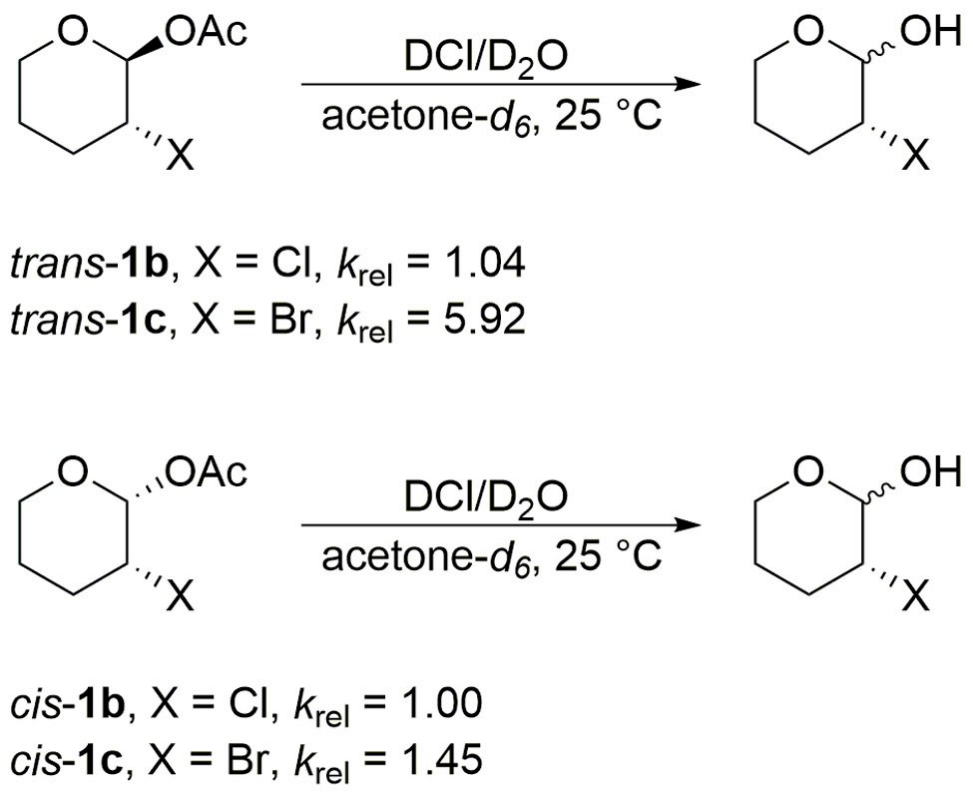
Rates of hydrolysis of halopyran stereoisomers.

Analysis of the rates of hydrolysis of the individual stereoisomers of halogenated pyrans **1** **b** and **1** **c** indicate the importance of the orientation of the C‐2 halogen atom on the ionization of the leaving group, which, in accordance with the Hammond postulate,^[37]^ should reflect the degree of stabilization of the oxocarbenium ion intermediate. The four acetals all adopted conformations that place the leaving group in an axial orientation, as inferred from analysis of ^1^H NMR spectra (Scheme 3), so the orientation of the leaving group to the most electron‐donating group, the endocyclic oxygen atom,^[22]^ are similar. The acetals differ only in the halogen atom and whether it adopts an axial or equatorial orientation. For the chlorine‐substituted pyran **1** **b**, the two acetals ionized at similar rates, indicating that the carbon‐chlorine bond did not stabilize the developing oxocarbenium ion intermediate, which is consistent with the calculations (Figure 3). By contrast, the diaxial isomer of the bromine‐substituted pyran ionized four times more rapidly than the conformer with the equatorial bromine atom. This modest difference suggests that the bromine atom does not facilitate loss of the leaving group through formation of a bromonium ion intermediate, just as neighboring groups such as acyloxy groups or thiophenyl groups do not participate in ionization by formation of onium ion intermediates.^[39]^ Instead, the carbon‐bromine bond is a stronger electron donor by hyperconjugation when oriented axially. The difference in rates between the halogen‐containing derivatives is also relatively small, with the chlorinated systems possessing a more inductively withdrawing halogen atom at C‐2, resulting in slower ionization. Consistent with the improved orbital overlap in furans, it was not possible to measure the rates of ionization of the *trans*‐substituted acetals because they hydrolyzed too readily.

## Conclusion

In conclusion, the presence of a C‐2‐halogen atom can guide the stereochemical courses of acetal substitution reactions through a halogen‐stabilized oxocarbenium ion. Diastereoselectivity is high for pyrans and furans that contain a chlorine, bromine, or iodine atom, whereas these reactions are generally unselective for systems with α‐fluorine atoms. Hyperconjugative interactions (σ_C‐X_→π*_C‐O_^+^) contribute to the stability of the intermediate, which increase as the donor ability of the carbon‐halogen bond increases (F≪Cl<Br≈I).^[22]^ In carbohydrate‐derived substrates, the influence of the halogen atom at C‐2 could depend upon the other electron‐withdrawing substituents in the ring, just as participation by an acyloxy group can be affected by how nearby alkoxy groups are functionalized.^[40]^ Nevertheless, the presence of a single halogen atom atom at C‐2 is sufficient to confer high stereoselectivity.

## Conflict of interest

The authors declare no conflict of interest.

1

## Supporting information

As a service to our authors and readers, this journal provides supporting information supplied by the authors. Such materials are peer reviewed and may be re‐organized for online delivery, but are not copy‐edited or typeset. Technical support issues arising from supporting information (other than missing files) should be addressed to the authors.

Supporting Information

Supporting Information

## Data Availability

The data that support the findings of this study are available from the corresponding authors upon reasonable request.
