# Supplementary material for: Halogen Atom Participation in Guiding the Stereochemical Outcomes of Acetal Substitution Reactions
Source: Angew Chem Int Ed Engl. 2022 Sep 16;61(42):e202209401. doi: 10.1002/anie.202209401 (PMC9561118; doi:10.1002/anie.202209401)
Supplement: Supplementary file 2 — Supporting Information [file ANIE-61-0-s001.pdf]

Angewandte Chemie
Eine Zeitschrift der Gesellschaft Deutscher Chemiker

Supporting Information

Halogen Atom Participation in Guiding the Stereochemical Outcomes of Acetal Substitution Reactions

K. M. Demkiw, W. A. Remmerswaal, T. Hansen, G. A. van der Marel, J. D. C. Codée, K. A. Woerpel**

Supporting Information

Table of Contents

I. General Experimental S3

II. Synthesis of Substrates S4

III. Nucleophile Additions to α-Haloacetals S9

- A. Additions of Carbon Nucleophiles to α-Haloacetals S10
- B. Additions of Oxygen Nucleophiles to α-Haloacetals S14

IV. Parameter Screen for *O*-Glycosylation Conditions S38

V. Epimerization Studies S41

- A. α-Chlorinated Pyrans S42
  - a. Acetal **7a** S42
  - b. Acetal **7b** S44
  - c. Acetal **7c** S45
  - d. Acetal **7d** S46
  - e. Acetal **7e** S47
- B. α-Brominated Pyrans S48
  - a. Acetal **8a** S48
  - b. Acetal **8b** S49
  - c. Acetal **8c** S50
  - d. Acetal **8d** S51
  - e. Acetal **8e** S52

C. α-Fluorinated Pyrans S54
a. Acetal **10a** S54
b. Acetal **10b** S555
c. Acetal **10c** S56
d. Acetal **10d** S57
e. Acetal **10e** S58
D. α-Chlorinated Pyrans S54
a. Acetal **19a** S54
b. Acetal **19b** S55
c. Acetal **19c** S60
d. Acetal **19d** S61
e. Acetal **19e** S62
E. α-Brominated Pyrans S63
a. Acetal **20a** S63
b. Acetal **20b** S65
c. Acetal **20c** S66
d. Acetal **20d** S67
e. Acetal **20e** S68
VI. Kinetic Experiments S70
A. General Procedure S70
B. Rate Data Collection S70
C. Kinetic Data S70
VII. Stereochemical Correlations and Proofs S75
A. Assignment of Relative Stereochemical Configurations by ^{1}H NMR Coupling Constants S75
B. Assignment of the Relative Stereochemical Configuration of Alkenes **12** and **14** by Derivatization to Furan **S6** S91
VIII. Computational Methods S94
A. Generation of the Computational Energy Landscapes S94
B. Computational Investigations of Cyclic Halonium Cations S94
C. Benchmark of Conformational Energy Landscape Methods S95
a. **Supplementary Table S1**. Single point benchmark energies for CCSD(T), DLPNO-CCSD(T), RI-MP2 and DLPNO-MP2. .... S96
b. **Supplementary Table S2**. Single point benchmark energies for revDSD-PBEP86-D4, DSD-PBEP86, RSX-QIDH and DSD-PBEB95. .... S97
c. **Supplementary Table S3**. Single point benchmark energies for ωB97X-2, RSX-0DH, PBE0-QIDH and B97M-V S98
d. **Supplementary Table S4**. Single point benchmark energies for PBE0-DH, B2PLYP, B2PLYP-D4, B2PLYP-D3(BJ) and mPW2PLYPb. S99

e. **Supplementary Table S5**. Single point benchmark energies for mPW2PLYP-D4, PBE0, PBE0-D4, PBE0-D3(BJ) and B97M-V-D4. .... S100

f. **Supplementary Table S6**. Single point benchmark energies for B97M-V-D3(BJ), ωB97M-V, ωB97X-V, B3LYP, B3LYP-D4. .... S101

g. **Supplementary Table S7**. Single point benchmark energies for B3LYP-D3(BJ), r^2SCAN, r^2SCAN-D4, r^2SCAN-D3(BJ) and ωb97M-V-D4. .... S102

h. **Supplementary Table S8**. Single point benchmark energies for ωB97M-V-D3(BJ), ωB97X-D4, ωB97X-D3(BJ), SCAN and SCAN-D4. .... S103

i. **Supplementary Table S9**. Single point benchmark energies for SCAN-D3(BJ), M06-2X, rSCAN, rSCAN-D4 and rSCAN-D3(BJ). S104

j. **Supplementary Table S10**. Single point benchmark energies for OLYP, OLYP-D4, OLYP-D3(BJ), PW6B95, PW6B95-D3(BJ), PW6B95-D4. .... S105

k. **Supplementary Table S11**. Cartesian coordinates (in Å), energies (*E*, *H* and *G*, in kcal/mol, T=195.15 K), and number of imaginary vibrational frequencies (N_{imag}) of selected CEL geometries: the lowest ^3E, E_3 and flat geometries from each furanyl cations, while for the pyranyl cations the lowest $^3H_4$ and $^4H_3$ are given. All were optimized with CEL dihedral angle constrains (furanyl: C1–C2–C3–C4, C5–O5–C1–C2; pyranyl: C1–C2–C3–C4, C3–C4–C5–O5, C5–O5–C1–C2) at PCM(dichloromethane)-B3LYP-D3(BJ)BJ-(SARC)-DKH-def2TZVP. S106

IX. X-Ray Crystallographic Data S114

X. References S116

XI. Selected ^{1}H, ^{13}C{^{1}H}, and ^{19}F{^{1}H} NMR Spectra S117

I. General Experimental

^{1}H NMR and ^{13}C{^{1}H} NMR spectra were obtained at room temperature using Bruker AVIII-400 (400 MHz and 100 MHz, respectively) and AVIIIHD-400 (400 MHz and 100 MHz, respectively) spectrometers. ^{19}F{^{1}H} NMR spectra were obtained at room temperature using a AVIII-400 (377 MHz) spectrometer. All spectroscopic data are reported as follows: chemical shifts are reported in ppm on the δ scale, ^{1}H and ^{13}C{^{1}H} NMR spectra are internally referenced to tetramethylsilane (^{1}H NMR: $CDCl_3$ δ 0.00; ^{13}C{^{1}H} NMR: $CDCl_3$ δ 0.00), ^{19}F{^{1}H} NMR spectra are externally referenced to trifluorotoluene (^{19}F{^{1}H} NMR: $CDCl_3$ δ –63.72), multiplicity (br = broad, s = singlet, d = doublet, t = triplet, q = quartet, m = multiplet), coupling constants (Hz), and integration. Ratios of products were derived from one-pulse ^{1}H NMR or ^{13}C{^{1}H} NMR integrations using diagnostic peaks in the unpurified reaction mixture.[1] Multiplicities of carbon peaks were defined using HSQC experiments. Infrared (IR) spectra were recorded using a Thermo Nicolet AVATAR Fourier Transform IR spectrometer using attenuated total reflectance (ATR). High-resolution mass spectra were acquired on an Agilent 6224 Accurate-Mass time-of-flight spectrometer and were obtained using peak matching. The ionization sources used were either atmospheric pressure chemical ionization (APCI) or electrospray ionization (ESI), as indicated. Liquid chromatography was performed using forced flow (flash chromatography) of the indicated solvent system on silica gel (SiO_2) 60 (230–400 mesh). Tetrahydrofuran, diethyl ether,

dichloromethane, and methanol were dried and degassed using a solvent purification system before use. All dry reactions were run under a nitrogen atmosphere in glassware that had been flame-dried under reduced pressure. Unless otherwise noted, all reagents and substrates were commercially available. 2,2,2-Trifluoro-*N*-phenylacetimidoyl chloride was prepared using a known method.[2]

II. Synthesis of Substrates

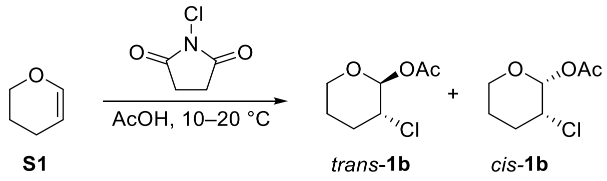

(2*R,3*S**)-3-Chlorotetrahydro-*2H*-pyran-2-yl acetate (*trans*-1b) and (2*R**,3*R**)-3-chlorotetrahydro-*2H*-pyran-2-yl acetate (cis-1b)**.

To a cooled (10 °C) suspension of *N*-chlorosuccinimide (2.539 g, 19.01 mmol) in AcOH (12 mL) was added 3,4-dihydro-*2H*-pyran (2.2 mL, 24 mmol). After 1 h, the mixture was warmed to 20 °C and stirred for an additional 1.5 h. Saturated aqueous $NaHCO_3$ (15 mL) was then added, the layers were separated, and the aqueous layer was extracted with Et_2O (2 × 20 mL). The combined organic layers were washed with H_2O (3 × 15 mL), saturated $NaHCO_3$ (2 × 15 mL), and brine (1 × 15 mL), dried over Na_2SO_4, filtered, and concentrated *in vacuo*. ^1H NMR and $^{13}C\{^1H\}$ NMR spectroscopic analysis of the unpurified reaction mixture revealed that acetal **1b** was formed as a 74:26 mixture of diastereomers (*trans*-**1b**:*cis*-**1b**). Purification by flash chromatography (10:90 EtOAc:hexanes) afforded acetal *trans*-**1b** and acetal *cis*-**1b** as a light yellow oil (3.361 g, 78%) with a diastereomeric ratio of 73:27. The spectroscopic data (^1H NMR, $^{13}C\{^1H\}$ NMR, IR, HRMS) are consistent with the data reported in literature.[3]

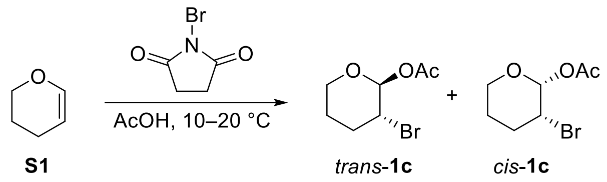

(2*R,3*S**)-3-Bromotetrahydro-*2H*-pyran-2-yl acetate (*trans*-1c) and (2*R**,3*R**)-3-bromotetrahydro-*2H*-pyran-2-yl acetate (*cis*-1c)**.

To a cooled (10 °C) suspension of *N*-bromosuccinimide (3.412 g, 19.17 mmol) in AcOH (12 mL) was added 3,4-dihydro-*2H*-pyran (2.2 mL, 24 mmol). After 1 h, the mixture was warmed to 20 °C and stirred for an additional 19 h. Saturated aqueous $NaHCO_3$ (15 mL) was then added, the layers were separated, and the aqueous layer was extracted with Et_2O (2 × 20 mL). The combined organic layers were washed with H_2O (3 × 15 mL), saturated $NaHCO_3$ (2 × 15 mL), and brine (1 × 15 mL), dried over Na_2SO_4, filtered, and concentrated *in vacuo*. ^1H NMR and $^{13}C\{^1H\}$ NMR spectroscopic analysis of the unpurified reaction mixture revealed that acetal **1c** was formed as a 68:42 mixture of diastereomers

(*trans*-**1c**:*cis*-**1c**). Purification by flash chromatography (10:90 EtOAc:hexanes) afforded acetal *trans*-**1c** and acetal *cis*-**1c** as a light yellow oil (1.831 g, 34%) with a diastereomeric ratio of 73:27. The spectroscopic data (^{1}H NMR, ^{13}C{^{1}H} NMR, HRMS) are consistent with the data reported in literature.[3]

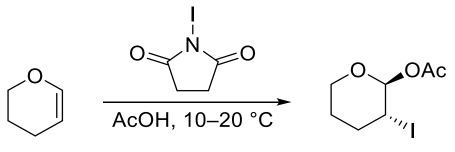

(2*R,3*S**)-3-Iodotetrahydro-*2H*-pyran-2-yl acetate (1d)**.

To a cooled (10 °C) suspension of *N*-iodosuccinimide (2.974 g, 13.22 mmol) in AcOH (11 mL) was added 3,4-dihydro-*2H*-pyran (1.5 mL, 17 mmol). After 15 min, the mixture was warmed to 20 °C and stirred for an additional 1 h. Et_2O (15 mL) and H_2O (10 mL) were then added, and the layers were separated. The organic layer was washed with H_2O (3 × 15 mL), saturated $NaHCO_3$ (2 × 15 mL), and brine (1 × 15 mL), dried over Na_2SO_4, filtered, and concentrated *in vacuo*. ^{1}H NMR and ^{13}C{^{1}H} NMR spectroscopic analysis of the unpurified reaction mixture revealed that acetal **1d** was formed as a single diastereomer. Purification by flash chromatography (10:90 EtOAc:hexanes) afforded acetal **1d** as a colorless oil (2.023 g, 57%). The spectroscopic data (^{1}H NMR, ^{13}C{^{1}H} NMR) are consistent with the data reported in literature:[4]

^{1}H NMR (400 MHz, $CDCl_3$) δ 5.89 (d, *J* = 5.8, 1H), 4.15–4.13 (m, 1H), 4.07–4.01 (m, 1H), 3.78–3.72 (m, 1H), 2.43–2.36 (m, 1H), 2.13 (s, 3H), 2.15–2.06 (m, 1H), 1.87–1.79 (m, 1H), 1.69–1.60 (m, 1H);

^{13}C{^{1}H} NMR (100 MHz, $CDCl_3$) δ 169.0 (C), 95.4 (CH), 65.0 (CH_2), 32.5 (CH_2), 26.2 (C), 25.2 (CH_2), 20.9 (CH_3);

IR (ATR) 2949, 1754, 1196, 1062, 1033, 863 cm^{-1};

HRMS (ESI) *m / z* calcd for $C_7H_{11}INaO_3$ $(M + Na)^+$ 292.9645, found 292.9656.

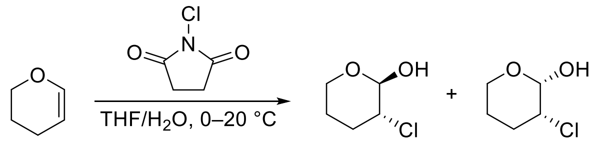

(2*R,3*R**)-3-Chlorotetrahydro-*2H*-pyran-2-ol (*trans*-3) and (2*R**,3*S**)-3-chlorotetrahydro-*2H*-pyran-2-ol (*cis*-3)**.

A reported procedure[5] was used to prepare hemiacetal **3**. To a cooled (0 °C) suspension of *N*-chlorosuccinimide (3.185 g, 23.85 mmol) in H_2O (5 mL) was added a solution of 3,4-dihydro-*2H*-pyran (2.2 mL, 24.1 mmol) in THF (10 mL). After 1 h, the mixture was warmed to 20 °C and stirred for an additional 3 h. Saturated aqueous $NaHCO_3$ (15 mL) was then added, the layers were separated, and the aqueous layer was extracted with CH_2Cl_2 (2 × 20 mL). The combined organic layers were dried

over Na_2SO_4, filtered, and concentrated *in vacuo*. ^1H NMR and $^{13}C\{^1H\}$ NMR spectroscopic analysis of the unpurified reaction mixture revealed that hemiacetal **3** was formed as a 58:42 mixture of diastereomers (*trans*-**3**:*cis*-**3**). Purification by flash chromatography (30:70 EtOAc:hexanes) afforded hemiacetal *trans*-**3** and hemiacetal *cis*-**3** as a colorless oil (2.902 g, 88%) with a diastereomeric ratio of 62:38. The spectroscopic data (^1H NMR, $^{13}C\{^1H\}$ NMR, HRMS) are consistent with the data reported in literature.[5]

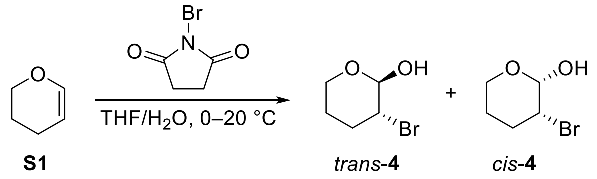

(2*R,3*R**)-3-Bromotetrahydro-*2H*-pyran-2-ol (*trans*-4) and (2*R**,3*S**)-3-bromotetrahydro-*2H*-pyran-2-ol (*cis*-4)**.

A reported procedure[5] was used to prepare hemiacetal **4**. To a cooled (0 °C) suspension of *N*-bromosuccinimide (4.285 g, 24.08 mmol) in H_2O (5 mL) was added a solution of 3,4-dihydro-*2H*-pyran (2.2 mL, 24 mmol) in THF (10 mL). After 1 h, the mixture was warmed to 20 °C and stirred for an additional 2.5 h. Saturated aqueous $NaHCO_3$ (15 mL) was then added, the layers were separated, and the aqueous layer was extracted with CH_2Cl_2 (2 × 20 mL). The combined organic layers were dried over Na_2SO_4, filtered, and concentrated *in vacuo*. ^1H NMR and $^{13}C\{^1H\}$ NMR spectroscopic analysis of the unpurified reaction mixture revealed that hemiacetal **4** was formed as a 51:49 mixture of diastereomers (*trans*-**4**:*cis*-**4**). Purification by flash chromatography (30:70 EtOAc:hexanes) afforded hemiacetal *trans*-**4** and hemiacetal *cis*-**4** as a colorless oil (4.102 g, 94%) with a diastereomeric ratio of 63:37. The spectroscopic data (^1H NMR, $^{13}C\{^1H\}$ NMR, HRMS) are consistent with the data reported in literature.[5]

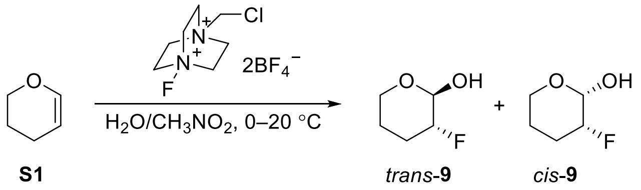

(2*R,3*R**)-3-Fluorotetrahydro-*2H*-pyran-2-ol (*trans*-9) and (2*R**,3*S**)-3-fluorotetrahydro-*2H*-pyran-2-ol (*cis*-9)**.

A reported procedure[5] was used to prepare hemiacetal **9**. To a cooled (0 °C) solution of 3,4-dihydro-*2H*-pyran (2.0 mL, 22 mmol) in CH_3NO_2 (55 mL) and H_2O (11 mL) was added Selectfluor® (11.714 g, 33.066 mmol). After 1 h, the mixture was warmed to 20 °C and stirred for an additional 12 h. The reaction mixture was then heated to reflux (110 °C) for 2 h and then concentrated *in vacuo* to remove nitromethane. Saturated aqueous $NaHCO_3$ (60 mL) was then added, the layers were separated, and the aqueous layer was extracted with CH_2Cl_2 (2 × 60 mL). The combined organic layers were dried over Na_2SO_4, filtered, and concentrated *in vacuo*. ^1H NMR and $^{13}C\{^1H\}$ NMR spectroscopic analysis of

the unpurified reaction mixture revealed that hemiacetal **9** was formed as a 60:40 mixture of diastereomers (*trans*-**9**:*cis*-**9**). Purification by flash chromatography (30:70 EtOAc:hexanes) afforded hemiacetal *trans*-**9** and hemiacetal *cis*-**9** as a colorless oil (1.198 g, 45%) with a diastereomeric ratio of 79:21. The spectroscopic data (^{1}H NMR, ^{13}C{^{1}H} NMR, HRMS) are consistent with the data reported in literature.[5]

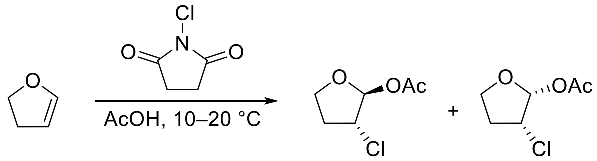

S2 *trans*-**11** *cis*-**11**

(2*R,3*S**)-3-Chlorotetrahydrofuran-2-yl acetate (*trans*-11) and (2*R**,3*R**)-3-chlorotetrahydrofuran-2-yl acetate (*cis*-11)**.

A reported procedure[5] was adapted to prepare acetal **11**. To a cooled (10 °C) suspension of *N*-chlorosuccinimide (2.33 g, 17.4 mmol) in AcOH (10 mL) was added 2,3-dihydro-2*H*-furan (1.60 mL, 21.6 mmol). After 1 h, the mixture was warmed to 20 °C and stirred for an additional 16 h. Saturated aqueous $NaHCO_3$ (15 mL) was then added, the layers were separated, and the aqueous layer was extracted with Et_2O (2 × 20 mL). The combined organic layers were washed with H_2O (1 × 15 mL), dried over Na_2SO_4, filtered, and concentrated *in vacuo*. ^{1}H NMR and ^{13}C{^{1}H} NMR spectroscopic analysis of the unpurified reaction mixture revealed that acetal **11** was formed as a 91:9 mixture of diastereomers (*trans*-**11**:*cis*-**11**). Purification by flash chromatography (10:90 EtOAc:hexanes) afforded acetal *trans*-**11** and acetal *cis*-**11** as a colorless oil (3.4162 g, 96%) with a diastereomeric ratio of 78:22. This mixture was used for characterization:

IR (ATR) 2908, 1735, 1224, 1007, 917, 901 cm^{-1};

HRMS (APCI) *m / z* calcd for C_4H_6ClO ((M + H) – $C_2H_4O_2$)$^+$ 105.0102, found 105.0100.

Anal. Calcd for $C_6H_9ClO_3$: C, 43.79; H, 5.51. Found: C, 43.87; H, 5.68.

Major Diastereomer *trans*-**11**:

^{1}H NMR (400 MHz, $CDCl_3$) δ 6.24 (s, 1H), 4.33 (dd, *J* = 5.5, 1.0, 1H), 4.26–4.20 (m, 2H), 2.58–2.48 (m, 1H), 2.24–2.18 (m, 1H), 2.06 (s, 3H);

^{13}C{^{1}H} NMR (100 MHz, $CDCl_3$) δ 169.5 (C), 102.3 (CH), 68.1 (CH_2), 59.8 (CH), 32.8 (CH_2), 21.1 (CH_3).

Minor Diastereomer *cis*-**11**:

^{1}H NMR (400 MHz, $CDCl_3$, diagnostic peaks) δ 6.30 (d, *J* = 4.0, 1H), 2.13 (s, 3H);

^{13}C{^{1}H} NMR (100 MHz, $CDCl_3$) δ 169.9 (C), 95.4 (CH), 67.2 (CH_2), 55.2 (CH), 31.8 (CH_2), 21.0 (CH_3).

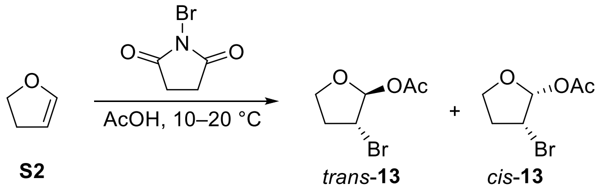

(2*R,3*S**)-3-bromotetrahydrofuran-2-yl acetate (*trans*-13) and (2*R**,3*R**)-3-bromotetrahydrofuran-2-yl acetate (*cis*-13)**.

A reported procedure[3] was used to prepare acetal **13**. To a cooled (10 °C) suspension of *N*-chlorosuccinimide (2.469 g, 13.87 mmol) in AcOH (7 mL) was added 2,3-dihydro-2*H*-furan (0.95 mL, 12.5 mmol). After 15 min, the mixture was warmed to 20 °C and stirred for an additional 16 h. Et_2O (10 mL) was then added, the layers were separated, and the organic layer was extracted with H_2O (4 × 15 mL) and saturated $NaHCO_3$ (3 × 15 mL). The combined organic layers were washed with brine (1 × 15 mL), dried over Na_2SO_4, filtered, and concentrated *in vacuo*. ^1H NMR and $^{13}C\{^1H\}$ NMR spectroscopic analysis of the unpurified reaction mixture revealed that acetal **13** was formed as a 91:9 mixture of diastereomers (*trans*-**13**:*cis*-**13**). Purification by flash chromatography (10:90 EtOAc:hexanes) afforded acetal *trans*-**13** and acetal *cis*-**13** as a colorless oil (0.832 g, 32%) with a diasteromeric ratio of 91:9. The spectroscopic data (^1H NMR, $^{13}C\{^1H\}$ NMR, IR, HRMS) are consistent with the data reported in literature.[3]

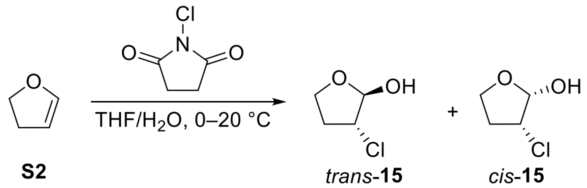

(2*R,3*R**)-3-Chlorotetrahydrofuran-2-ol (*trans*-15) and (2*R**,3*S**)-3-chlorotetrahydrofuran-2-ol (*cis*-15)**.

A reported procedure[5] was adapted to prepare hemiacetal **15**. To a cooled (0 °C) suspension of *N*-chlorosuccinimide (3.555 g, 26.62 mmol) in H_2O (6 mL) was added a solution of 2,3-dihydro-2*H*-furan (2.0 mL, 26 mmol) in THF (11 mL). After 1 h, the mixture was warmed to 20 °C and stirred for an additional 17 h. Saturated aqueous $NaHCO_3$ (15 mL) was then added, the layers were separated, and the aqueous layer was extracted with CH_2Cl_2 (2 × 20 mL). The combined organic layers were washed with brine (1 × 15 mL), dried over Na_2SO_4, filtered, and concentrated *in vacuo*. ^1H NMR and $^{13}C\{^1H\}$ NMR spectroscopic analysis of the unpurified reaction mixture revealed that hemiacetal **15** was formed as a 90:10 mixture of diastereomers (*trans*-**15**:*cis*-**15**). Purification by flash chromatography (30:70 EtOAc:hexanes) afforded hemiacetal *trans*-**15** and hemiacetal *cis*-**15** as a colorless oil (1.904 g, 60%) with a diastereomeric ratio of 85:15. This mixture was used for characterization. The spectroscopic data (^1H NMR, $^{13}C\{^1H\}$ NMR) are consistent with the data reported in literature:[6]

IR (ATR) 3392, 2963, 993, 924, 855, 740 cm^{-1};

HRMS (ESI) *m / z* calcd for C_4H_6ClO ((M + H) – H_2O)$^+$ 105.0102, found 105.0105.

Major Diastereomer *trans*-**15**:

^{1}H NMR (400 MHz, $CDCl_3$) δ 5.48 (d, *J* = 3.0, 1H), 4.25 (dd, *J* = 5.6, 1.3, 1H), 4.20–4.16 (m, 2H), 3.18 (d, *J* = 2.9, 1H), 2.64–2.55 (m, 1H), 2.18–2.12 (m, 1H);

$^{13}C\{^{1}H\}$ NMR (100 MHz, $CDCl_3$) δ 103.2 (CH), 67.0 (CH_2), 61.0 (CH), 32.9 (CH_2);

Minor Diastereomer *cis*-**15**:

^{1}H NMR (400 MHz, $CDCl_3$, diagnostic peaks) δ 5.37 (dd, *J* = 8.1, 3.5, 1H), 4.33–4.29 (m, 1H), 3.95–3.90 (m, 1H), 3.11 (d, *J* = 2.7, 1H), 2.55–2.43 (m, 1H), 2.34–2.26 (m, 1H);

$^{13}C\{^{1}H\}$ NMR (100 MHz, $CDCl_3$) δ 97.0 (CH), 65.1 (CH_2), 59.9 (CH), 33.1 (CH_2).

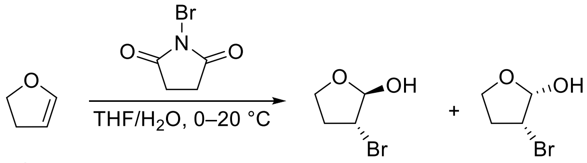

S2 *trans*-**16** *cis*-**16**

(2*R**,**3*R****)-3-Bromotetrahydrofuran-2-ol (**trans**-16) and (2*R****,**3*S****)-3-bromotetrahydrofuran-2-ol (*cis*-16)**.

A reported procedure[5] was adapted to prepare hemiacetal **16**. To a cooled (0 °C) suspension of *N*-bromosuccinimide (4.739 g, 26.63 mmol) in H_2O (6 mL) was added a solution of 2,3-dihydro-*2H*-furan (2.0 mL, 26 mmol) in THF (11 mL). After 1 h, the mixture was warmed to 20 °C and stirred for an additional 1 h. Saturated aqueous $NaHCO_3$ (15 mL) was then added, the layers were separated, and the aqueous layer was extracted with CH_2Cl_2 (2 × 20 mL). The combined organic layers were washed with brine (1 × 15 mL), dried over Na_2SO_4, filtered, and concentrated *in vacuo*. ^{1}H NMR and $^{13}C\{^{1}H\}$ NMR spectroscopic analysis of the unpurified reaction mixture revealed that hemiacetal **16** was formed as a 93:7 mixture of diastereomers (*trans*-**16**:*cis*-**16**). Purification by flash chromatography (30:70 EtOAc:hexanes) afforded hemiacetal *trans*-**16** and hemiacetal *cis*-**16** as a colorless oil (2.951 g, 68%) with a diastereomeric ratio of 85:15. This mixture was used for characterization:

IR (ATR) 3380, 2900, 1254, 988, 921, 850 cm^{-1};

HRMS (APCI) *m / z* calcd for C_4H_6BrO ((M + H) – H_2O)$^+$ 148.9597, found 148.9593.

Major Diastereomer *trans*-**16**:

^{1}H NMR (400 MHz, $CDCl_3$) δ 5.61 (d, *J* = 2.8, 1H), 4.24 (dd, *J* = 5.6, 1.1, 1H), 4.21–4.15 (m, 2H), 3.16 (br s, 1H), 2.75–2.64 (m, 1H), 2.28–2.20 (m, 1H);

$^{13}C\{^{1}H\}$ NMR (100 MHz, $CDCl_3$) δ 103.5 (CH), 67.1 (CH), 50.8 (CH_2), 33.4 (CH_2).

Minor Diastereomer *cis*-**16**:

^{1}H NMR (400 MHz, $CDCl_3$) δ 5.25 (dd, *J* = 7.8, 3.5, 1H), 4.34–4.29 (m, 1H), 4.22–4.15 (m, 1H), 3.95–3.88 (m, 1H), 3.28 (br s, 1H), 2.61–2.51 (m, 1H), 2.45–2.35 (m, 1H);

$^{13}C\{^{1}H\}$ NMR (100 MHz, $CDCl_3$) δ 96.8 (CH), 65.4 (CH), 51.4 (CH_2), 33.5 (CH_2).

III. Nucleophile Additions to α-Haloacetals

A. Additions of Carbon Nucleophiles to α-Haloacetals

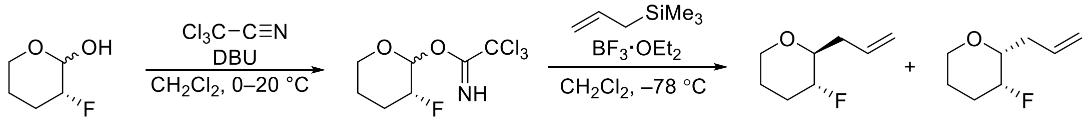

(2*R**,3*S****)-2-Allyl-3-fluorotetrahydro-*2H*-pyran (*trans*-2a) and (2*R****,3*R****)-2-allyl-3-fluorotetrahydro-*2H*-pyran (*cis*-2a)**.

A reported procedure[7] was adapted to prepare trichloroacetimidate **1a**. To a cooled (0 °C) solution of hemiacetal **9** (0.131 g, 1.09 mmol) in CH_2Cl_2 (18 mL) was added trichloroacetonitrile (1.1 mL, 11 mmol) and DBU (20 µL, 0.13 mmol). After 5 min, the mixture was warmed to 20 °C and stirred for an additional 2 h. The reaction mixture was then concentrated *in vacuo* and trichloroacetimidate **1a** was directly used without further purification. To a cooled (–78 °C) solution of trichloroacetimidate **1a** (1.09 mmol) and allyltrimethylsilane (690 µL, 4.34 mmol) in CH_2Cl_2 (9 mL) was added $BF_3·OEt_2$ (270 µL, 2.15 mmol) dropwise over 2 min. After 1 h, Et_3N (100 µL) was added, and the reaction mixture was concentrated *in vacuo*. ^{1}H NMR and ^{13}C{^{1}H} NMR spectroscopic analysis of the unpurified reaction mixture revealed that alkene **2a** was formed as a 4:96 mixture of diastereomers (*trans*-**2a**:*cis*-**2a**). Purification by flash chromatography (5:95 Et_2O:pentanes) afforded the major diastereomer *trans*-**2a** as a colorless oil (0.039 g, 25%). The relative stereochemical configurations of the two compounds were assigned by ^{1}H NMR coupling constants; details are provided later in Section VII:

Major Diastereomer *cis*-**2a**:

^{1}H NMR (400 MHz, $CDCl_3$) δ 5.88–5.78 (m, 1H), 5.18–5.09 (m, 2H), 4.50 (dddd, J = 47.8, 2.7, 2.7, 0.7 1H), 4.05–4.01 (m, 1H), 3.51–3.45 (m, 1H), 3.35 (dt, J = 29.2, 7.1, 1H), 2.48–2.32 (m, 2H), 2.21–2.14 (m, 1H), 2.05–1.93 (m, 1H), 1.76–1.56 (m, 1H). 1.45–1.39 (m, 1H);

^{13}C{^{1}H} NMR (100 MHz, $CDCl_3$) δ 134.1 (CH), 117.8 (CH_2), 87.8–86.0 (br d, $^1J_{C–F}$ = 177.3, CH), 78.0–77.8 (br d, $^2J_{C–F}$ = 18.8, CH), 68.2 (CH_2), 36.0 (br d, $^3J_{C–F}$ = 5.0, CH_2) 28.7–28.4 (br d, $^2J_{C–F}$ = 22.0, CH_2), 20.4 (CH_2);

^{19}F{^{1}H} NMR (377 MHz, $CDCl_3$) δ –191.4 (s);

IR (ATR) 2949, 1643, 1093, 1052, 912, 889 cm^{-1};

HRMS (APCI) m / z calcd for $C_8H_{13}O$ ((M + H) – HF)$^+$ 125.09611, found 125.0966.

Minor Diastereomer *trans*-**2a**:

^{13}C{^{1}H} NMR (100 MHz, $CDCl_3$, diagnostic peaks) δ 134.3, 36.2.

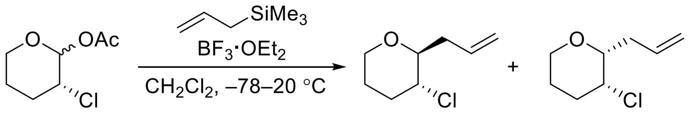

1b trans-**2b** cis-**2b**

General procedure for the additions of carbon nucleophiles to α-halo acetals ((2*R,3*S**)-2-allyl-3-chlorotetrahydro-2*H*-pyran (*trans*-2b) and (2*R**,3*R**)-2-allyl-3-chlorotetrahydro-2*H*-pyran (*cis*-2b))**.

To a cooled (–78 °C) solution of acetal **1b** (0.182 g, 1.07 mmol) and allyltrimethylsilane (655 µL, 4.12 mmol) in CH_2Cl_2 (6 mL) was added $BF_3{\cdot}OEt_2$ (260 µL, 2.07 mmol) dropwise over 2 min. After 1 h, the mixture was warmed to 20 °C and stirred for an additional 2 h. Saturated aqueous $NaHCO_3$ (5 mL) was then added, the layers were separated, and the aqueous layer was extracted with CH_2Cl_2 (3 × 10 mL). The combined organic layers were washed with brine (1 × 15 mL), dried over Na_2SO_4, filtered, and concentrated *in vacuo*. ^{1}H NMR and ^{13}C{^{1}H} NMR spectroscopic analysis of the unpurified reaction mixture revealed that alkene **2b** was formed as an 86:14 mixture of diastereomers (*trans*-**2b**:*cis*-**2b**). Purification by flash chromatography (2:98 EtOAc:hexanes) afforded the major diastereomer *trans*-**2b** as a light yellow oil (0.114 g, 66%). The relative stereochemical configurations of the two compounds were assigned by ^{1}H NMR coupling constants; details are provided later in Section VII:

Major Diastereomer *trans*-**2b**:

^{1}H NMR (400 MHz, $CDCl_3$) δ 5.93–5.82 (m, 1H), 5.18–5.08 (m, 2H), 4.00–3.93 (m, 1H), 3.64 (ddd, *J* = 10.8, 10.1, 4.7, 1H), 3.45–3.38 (m, 1H), 3.32 (ddd, *J* = 10.1, 7.6, 2.9, 1H), 2.72–2.64 (m, 1H), 2.37–2.27 (m, 2H), 1.84–1.64 (m, 3H);

^{13}C{^{1}H} NMR (100 MHz, $CDCl_3$) δ 134.1 (CH), 117.4 (CH_2), 81.9 (CH), 68.1 (CH_2), 58.5 (CH), 36.9 (CH_2), 34.8 (CH_2), 27.2 (CH_2);

IR (ATR) 3077, 2851, 1115, 1091, 913, 764 cm^{-1};

HRMS (ESI) *m / z* calcd for $C_8H_{13}O$ ((M + H) – HCl)$^+$ 125.0961, found 125.0963.

Minor Diastereomer *cis*-**2b**:

^{1}H NMR (400 MHz, $CDCl_3$, diagnostic peaks) δ 5.82–5.72 (m, 1H), 4.06–4.02 (m, 1H);

^{13}C{^{1}H} NMR (100 MHz, $CDCl_3$) δ 133.6 (CH), 118.0 (CH_2), 78.6 (CH), 68.6 (CH_2), 59.3 (CH), 38.0 (CH_2), 32.0 (CH_2), 22.0 (CH_2).

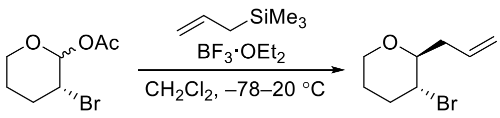

1c **2c**

(2*R,3*S**)-2-Allyl-3-bromotetrahydro-2*H*-pyran (2c)**.

Alkene **2c** was prepared using the representative procedure for additions of carbon nucleophiles to α-halo acetals using acetal **1c** (0.313 g, 1.40 mmol), allyltrimethylsilane (860 µL, 5.41 mmol), and $BF_3·OEt_2$ (340 µL, 2.71 mmol) in CH_2Cl_2 (14 mL). ^{1}H NMR and ^{13}C{^{1}H} NMR spectroscopic analysis of the unpurified reaction mixture revealed that alkene **2c** was formed as a single diastereomer (dr >99:1). Purification by flash chromatography (2:98 EtOAc:hexanes) afforded alkene **2c** as a light yellow oil (0.285 g, 99%). The relative stereochemical configurations of the compound was assigned by ^{1}H NMR coupling constants; details are provided later in Section VII:

^{1}H NMR (400 MHz, $CDCl_3$) δ 5.91–5.81 (m, 1H), 5.17–5.10 (m, 2H), 4.03–3.99 (m, 1H), 3.80 (ddd, *J* = 11.8, 10.0, 4.5, 1H), 3.49–3.42 (m, 2H), 2.74–2.69 (m, 1H), 2.47–2.31 (m, 2H), 2.03–1.93 (m, 1H), 1.82–1.70 (m, 1H), 1.65–1.61 (m, 1H);

^{13}C{^{1}H} NMR (100 MHz, $CDCl_3$) δ 134.0 (CH), 117.5 (CH_2), 81.7 (CH), 68.3 (CH_2), 51.5 (CH), 37.9 (CH_2), 35.9 (CH_2), 28.4 (CH_2);

IR (ATR) 2849, 1433, 1080, 1019, 913, 716 cm^{-1};

HRMS (APCI) *m / z* calcd for $C_8H_{13}O$ ((M + H) – HBr)$^+$ 125.0961, found 125.0960.

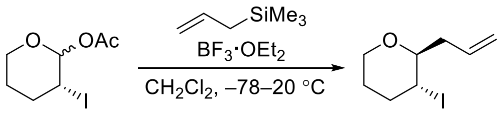

1d **2d**

(2*R,3*S**)-2-Allyl-3-iodotetrahydro-2*H*-pyran (2d)**.

Alkene **2d** was prepared using the representative procedure for additions of carbon nucleophiles to α-halo acetals using acetal **1d** (0.300 g, 1.11 mmol), allyltrimethylsilane (705 µL, 4.44 mmol), and $BF_3·OEt_2$ (280 µL, 2.23 mmol) in CH_2Cl_2 (11 mL). ^{1}H NMR and ^{13}C{^{1}H} NMR spectroscopic analysis of the unpurified reaction mixture revealed that alkene **2d** was formed as a single diastereomer (dr >99:1). Purification by flash chromatography (3:97 EtOAc:hexanes) afforded alkene **2d** as a light yellow oil (0.184 g, 66%). The relative stereochemical configurations of the compound was assigned by ^{1}H NMR coupling constants; details are provided later in Section VII:

^{1}H NMR (400 MHz, $CDCl_3$) δ 5.89–5.79 (m, 1H), 5.18–5.10 (m, 2H), 4.10–4.06 (m, 1H), 3.94 (ddd, *J* = 12.1, 10.3, 4.4, 1H), 3.58–3.48 (m, 2H), 2.79–2.73 (m, 1H), 2.58–2.53 (m, 1H), 2.42–2.34 (m, 1H), 2.26–2.15 (m, 1H), 1.82–1.70 (m, 1H), 1.49–1.45 (m, 1H);

^{13}C{^{1}H} NMR (100 MHz, $CDCl_3$) δ 134.0 (CH), 117.5 (CH_2), 81.9 (CH), 68.7 (CH_2), 39.6 (CH_2), 38.4 (CH_2), 32.0 (CH), 29.8 (CH_2);

IR (ATR) 3075, 2941, 1641, 1092, 1016, 914 cm^{-1};

HRMS (ESI) *m / z* calcd for $C_8H_{16}NO$ ((M + NH_4) – HI)$^+$ 142.1226, found 142.1227.

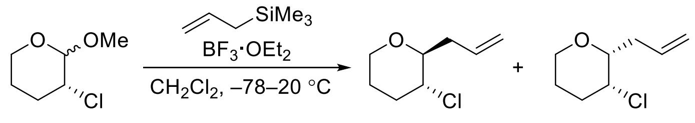

1e *trans*-**2e** *cis*-**2e**

(2*R,3*S**)-2-Allyl-3-chlorotetrahydro-2*H*-pyran (*trans*-2e) and (2*R**,3*R**)-2-allyl-3-chlorotetrahydro-2*H*-pyran (*cis*-2e)**.

Alkene **2e** was prepared using the representative procedure for additions of carbon nucleophiles to α-halo acetals using acetal **1e** (0.164 g, 1.09 mmol), allyltrimethylsilane (670 µL, 4.22 mmol), and $BF_3·OEt_2$ (270 µL, 2.15 mmol) in CH_2Cl_2 (11 mL). ^{1}H NMR and ^{13}C{^{1}H} NMR spectroscopic analysis of the unpurified reaction mixture revealed that alkene **2e** was formed as an 84:16 mixture of diastereomers (*trans*-**2e**:*cis*-**2e**) in 67% conversion. The spectroscopic data are consistent with those of the same alkene prepared from acetal **1b** and allyltrimethylsilane.

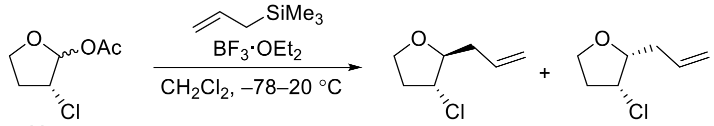

11 *trans*-**12** *cis*-**12**

(2*R,3*S**)-2-Allyl-3-chlorotetrahydrofuran (*trans*-12) and (2*R**,3*R**)-2-allyl-3-chlorotetrahydrofuran (*cis*-12)**.

Alkene **12** was prepared using the representative procedure for additions of carbon nucleophiles to α-halo acetals using acetal **11** (1.014 g, 6.16 mmol), allyltrimethylsilane (3.90 mL, 24.5 mmol), and $BF_3·OEt_2$ (1.50 mL, 11.9 mmol) in CH_2Cl_2 (10 mL). ^{1}H NMR and ^{13}C{^{1}H} NMR spectroscopic analysis of the unpurified reaction mixture revealed that alkene **12** was formed as an 86:14 mixture of diastereomers (*trans*-**12**:*cis*-**12**). Purification by flash chromatography (3:97 EtOAc:hexanes) afforded the major diastereomer *trans*-**12** as a colorless oil (0.798 g, 88%) and the minor diastereomer *cis*-**12** as a colorless oil (0.066 g, 7%). The relative stereochemical configurations of the two compounds were assigned by derivatization of alkene *trans*-**12** to form furan **S6**; experimental details are provided later in Section VII:

Major Diastereomer *trans*-**12**:

^{1}H NMR (400 MHz, $CDCl_3$) δ 5.88–5.78 (m, 1H), 5.17–5.11 (m, 2H), 4.03–3.93 (m, 4H), 2.45–2.36 (m, 2H), 2.33–2.26 (m, 1H), 2.17–2.09 (m, 1H);

$^{13}C\{^1H\}$ NMR (100 MHz, $CDCl_3$) δ 133.6 (CH), 117.9 (CH_2), 86.1 (CH), 66.5 (CH_2), 59.4 (CH), 37.7 (CH_2), 36.1 (CH_2);

IR (ATR) 2981, 1065, 997, 916, 836, 712 cm^{-1};

HRMS (APCI) *m / z* calcd for C_4H_6ClO ((M + H) – C_4H_8)$^+$ 105.0102, found 105.0099.

Minor Diastereomer *cis*-**12**:

^1H NMR (400 MHz, $CDCl_3$) δ 5.88–5.78 (m, 1H), 5.22–5.09 (m, 2H), 4.44–4.42 (m, 1H), 4.17–4.11 (m, 1H), 3.94–3.86 (m, 2H), 2.57–2.42 (m, 3H), 2.32–2.25 (m 1H);

$^{13}C\{^1H\}$ NMR (100 MHz, $CDCl_3$, diagnostic peaks) δ 134.0 (CH), 117.7 (CH_2), 81.8 (CH), 65.8 (CH_2), 61.7 (CH), 36.9 (CH_2), 35.7 (CH_2).

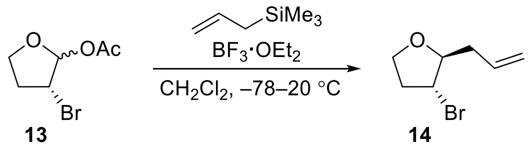

(2*R,3*S**)-2-Allyl-3-bromotetrahydrofuran (14)**.

Alkene **14** was prepared using the representative procedure for additions of carbon nucleophiles to α-halo acetals using acetal **13** (0.219 g, 1.05 mmol), allyltrimethylsilane (640 µL, 4.03 mmol), and BF_3·OEt_2 (250 µL, 1.99 mmol) in CH_2Cl_2 (10 mL). ^1H NMR and $^{13}C\{^1H\}$ NMR spectroscopic analysis of the unpurified reaction mixture revealed that alkene **14** was formed as a single diastereomer (dr >99:1). Purification by flash chromatography (3:97 EtOAc:hexanes) afforded alkene **14** as a light yellow oil (0.163 g, 81%). The relative stereochemical configuration of alkene **14** was assigned by derivatization to form furan **S6**; experimental details are provided later in Section VII:

^1H NMR (400 MHz, $CDCl_3$) δ 5.88–5.78 (m, 1H), 5.17–5.11 (m, 2H), 4.10 (q, *J* = 5.5, 1H), 4.02–3.92 (m, 3H), 2.53–2.39 (m, 2H), 2.32–2.20 (m, 2H);

$^{13}C\{^1H\}$ NMR (100 MHz, $CDCl_3$) δ 133.6 (CH), 117.9 (CH_2), 86.2 (CH), 66.7 (CH_2), 48.8 (CH), 37.6 (CH_2), 36.5 (CH_2);

IR (ATR) 2980, 1181, 1062, 997, 916, 834 cm^{-1};

HRMS (APCI) *m / z* calcd for $C_7H_{11}O$ ((M + H) – HBr)$^+$ 111.0804, found 111.0807.

B. Additions of Oxygen Nucleophiles to α-Haloacetals

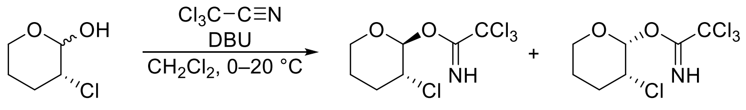

3 *trans*-**5a** *cis*-**5a**

General procedure for trichloroacetimidate formation ((2*R,3*S**)-3-chlorotetrahydro-2*H*-pyran-2-yl 2,2,2-trichloroacetimidate (*trans*-5a) and (2*R**,3*R**)-3-chlorotetrahydro-2*H*-pyran-2-yl 2,2,2-trichloroacetimidate (*cis*-5a))**.

A reported procedure[7] was adapted to prepare trichloroacetimidate **5a**. To a cooled (0 °C) solution of hemiacetal **3** (0.302 g, 2.24 mmol) in CH_2Cl_2 (22 mL) was added trichloroacetonitrile (2.3 mL, 23 mmol) and DBU (33 µL, 0.22 mmol). After 5 min, the mixture was warmed to 20 °C and stirred for an additional 1 h. The reaction mixture was then concentrated *in vacuo* and trichloroacetimidate **5a** was directly used without further purification.

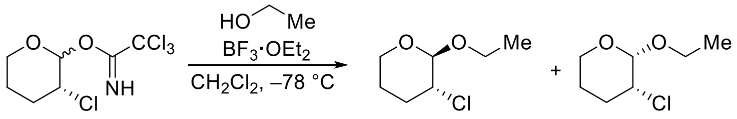

5a *trans*-**7a** *cis*-**7a**

General procedure for the additions of oxygen nucleophiles to α-halo acetals (2*R,3*R**)-3-chloro-2-ethoxytetrahydro-2*H*-pyran (*trans*-7a) and (2*R**,3*S**)-3-chloro-2-ethoxytetrahydro-2*H*-pyran (*cis*-7a)**.

To a cooled (–78 °C) solution of trichloroacetimidate **5a** (2.24 mmol) and ethanol (520 µL, 8.91 mmol) in CH_2Cl_2 (20 mL) was added $BF_3{\cdot}OEt_2$ (560 µL, 4.46 mmol) dropwise over 2 min. After 1 h, Et_3N (300 µL) was added, and the reaction mixture was warmed to 20 °C and concentrated *in vacuo*. ^{1}H NMR and $^{13}C\{^1H\}$ NMR spectroscopic analysis of the unpurified reaction mixture revealed that acetal **7a** was formed as an 84:16 mixture of diastereomers (*trans*-**7a**:*cis*-**7a**). Purification by flash chromatography (5:95 EtOAc:hexanes) afforded acetal **7a** as a colorless oil (0.158 g, 43%) with a diastereomeric ratio of 91:9. This mixture was used for characterization. The relative stereochemical configurations of the two compounds were assigned by ^{1}H NMR coupling constants; details are provided later in Section VII:

IR (ATR) 2973, 1135, 1072, 1044, 1002, 872 cm^{-1};

HRMS (APCI) *m / z* calcd for $C_5H_{12}ClNO$ ((M + NH_4) – C_2H_5O)$^+$ 137.0602, found 137.0600.

Major Diastereomer *trans*-**7a**:

^{1}H NMR (400 MHz, $CDCl_3$) δ 4.52 (d, *J* = 4.3, 1H), 3.92–3.79 (m, 3H), 3.60–3.52 (m, 2H), 2.35–2.28 (m, 1H), 1.99–1.79 (m, 2H), 1.57–1.47 (m, 1H), 1.24 (t, *J* = 7.1, 3H);

$^{13}C\{^{1}H\}$ NMR (100 MHz, $CDCl_3$) δ 100.9 (CH), 63.9 (CH_2), 62.4 (CH_2), 56.9 (CH), 29.5 (CH_2), 22.4 (CH_2), 15.1 (CH_3).

Minor Diastereomer *cis*-**7a**:

^{1}H NMR (400 MHz, $CDCl_3$, diagnostic peaks) δ 4.73 (d, J = 2.6, 1H), 3.99 (ddd, J = 11.3, 4.4, 2.9, 1H), 1.27 (t, J = 7.1, 3H);

$^{13}C\{^{1}H\}$ NMR (100 MHz, $CDCl_3$) δ 97.6 (CH), 63.6 (CH_2), 59.3 (CH_2), 57.3 (CH), 28.4 (CH_2), 25.7 (CH_2), 14.9 (CH_3).

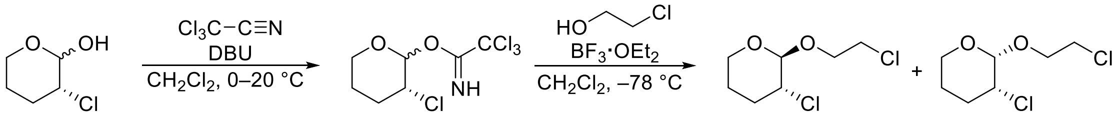

3 5a *trans*-**7b** *cis*-**7b**

(2*R**,3*S****)-3-Chloro-2-(2-chloroethoxy)tetrahydro-2*H*-pyran (*trans*-7b) and (2*R****,3*R****)-3-chloro-2-(2-chloroethoxy)tetrahydro-2*H*-pyran (*cis*-7b)**.

Trichloroacetimidate **5a** was prepared using the representative procedure for trichloroacetimidate formation using hemiacetal **3** (0.298 g, 2.21 mmol), trichloroacetonitrile (2.3 mL, 23 mmol), and DBU (40 µL, 0.27 mmol) in CH_2Cl_2 (22 mL). Acetal **7b** was prepared using the representative procedure for additions of oxygen nucleophiles to α-halo acetals using trichloroacetimidate **5a** (2.21 mmol), 2-chloroethanol (600 µL, 8.95 mmol), and $BF_3·OEt_2$ (560 µL, 4.46 mmol) in CH_2Cl_2 (22 mL). ^{1}H NMR and $^{13}C\{^{1}H\}$ NMR spectroscopic analysis of the unpurified reaction mixture revealed that acetal **7b** was formed as an 86:14 mixture of diastereomers (*trans*-**7b**:*cis*-**7b**). Purification by flash chromatography (5:95 EtOAc:hexanes) afforded acetal *trans*-**7b** and acetal *cis*-**7b** as a colorless oil (0.303 g, 68%) with a diastereomeric ratio of 90:10. This mixture was used for characterization. The relative stereochemical configurations of the two compounds were assigned by ^{1}H NMR coupling constants; details are provided later in Section VII:

IR (ATR) 2950, 1132, 1071, 1031, 872, 731 cm^{-1};

HRMS (APCI) m / z calcd for $C_7H_{12}ClO_2$ ((M + H) – HCl)$^+$ 163.0520, found 163.0514.

Major Diastereomer *trans*-**7b**:

^{1}H NMR (400 MHz, $CDCl_3$) δ 4.61 (d, J = 3.9, 1H), 4.02–3.96 (m, 1H), 3.94–3.88 (m, 2H), 3.83–3.75 (m, 1H), 3.69–3.66 (m, 2H), 3.62–3.57 (m, 1H), 2.35–2.29 (m, 1H), 2.03–1.93 (m, 1H), 1.89–1.84 (m, 1H), 1.55–1.47 (m, 1H);

$^{13}C\{^{1}H\}$ NMR (100 MHz, $CDCl_3$) δ 101.0 (CH), 68.2 (CH_2), 62.2 (CH_2), 56.3 (CH), 42.7 (CH_2), 28.8 (CH_2), 21.8 (CH_2).

Minor Diastereomer *cis*-**7b**:

^{1}H NMR (400 MHz, $CDCl_3$, diagnostic peaks) δ 4.77 (d, J = 2.8, 1H), 2.21–2.11 (m, 1H);

^{13}C{^{1}H} NMR (100 MHz, $CDCl_3$) δ 98.2 (CH), 68.5 (CH_2), 59.5 (CH_2), 56.9 (CH), 40.8 (CH_2), 28.2 (CH_2), 25.7 (CH_2).

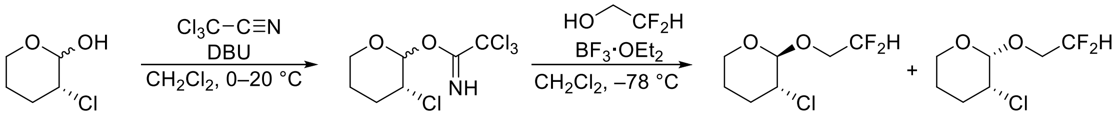

3 **5a** *trans*-**7c** *cis*-**7c**

(2*R,3*S**)-3-Chloro-2-(2,2-difluoroethoxy)tetrahydro-2*H*-pyran (*trans*-7c) and (2*R**,3*R**)-3-chloro-2-(2,2-difluoroethoxy)tetrahydro-2*H*-pyran (*cis*-7c)**.

Trichloroacetimidate **5a** was prepared using the representative procedure for trichloroacetimidate formation using hemiacetal **3** (0.302 g, 2.24 mmol), trichloroacetonitrile (2.3 mL, 23 mmol), and DBU (33 µL, 0.22 mmol) in CH_2Cl_2 (22 mL). Acetal **7c** was prepared using the representative procedure for additions of oxygen nucleophiles to α-halo acetals using trichloroacetimidate **5a** (2.24 mmol), 2,2-difluoroethanol (560 µL, 8.85 mmol), and $BF_3{\cdot}OEt_2$ (560 µL, 4.46 mmol) in CH_2Cl_2 (22 mL). ^{1}H NMR and ^{13}C{^{1}H} NMR spectroscopic analysis of the unpurified reaction mixture revealed that acetal **7c** was formed as a 92:8 mixture of diastereomers (*trans*-**7c**:*cis*-**7c**). Purification by flash chromatography (5:95 EtOAc:hexanes) afforded acetal *trans*-**7c** and acetal *cis*-**7c** and as a light yellow oil (0.197 g, 44%) with a diastereomeric ratio of 94:6. This mixture was used for characterization: The relative stereochemical configurations of the two compounds were assigned by ^{1}H NMR coupling constants; details are provided later in Section VII:

IR (ATR) 2958, 1101, 1069, 1051, 832, 798 cm^{-1};

HRMS (APCI) *m / z* calcd for C_5H_8ClO ((M + H) – $C_2H_4F_2O$)$^+$ 119.0258, found 119.0261.

Major Diastereomer *trans*-**7c**:

^{1}H NMR (400 MHz, $CDCl_3$) δ 6.08–5.78 (m, 1H), 4.62 (d, J = 3.8, 1H), 3.95–3.84 (m, 3H), 3.81–3.71 (m, 1H), 3.63–3.58 (m, 1H), 2.35–2.28 (m, 1H), 2.04–1.94 (m, 1H), 1.89–1.82 (m, 1H), 1.56–1.47 (m, 1H);

^{13}C{^{1}H} NMR (100 MHz, $CDCl_3$) δ 114.1 (br t, $^{1}J_{C-F}$ = 241.1, CH), 101.2 (CH), 67.1 (br t, $^{2}J_{C-F}$ = 28.7, CH_2), 62.2 (CH_2), 55.9 (CH), 28.6 (CH_2), 21.6 (CH_2);

^{19}F{^{1}H} NMR (377 MHz, $CDCl_3$) δ –126.2 (s).

Minor Diastereomer *cis*-**7c**:

^{1}H NMR (400 MHz, $CDCl_3$, diagnostic peaks) δ 4.78 (d, J = 2.8, 1H), 4.58–4.46 (m, 2H), 4.01–3.99 (ddd, J = 11.6, 4.5, 3.0, 1H), 2.19–2.09 (m, 1H), 1.77–1.73 (m, 2H);

^{13}C{^{1}H} NMR (100 MHz, $CDCl_3$, diagnostic peaks) δ 98.5 (CH), 59.5 (CH_2), 56.5 (CH), 28.1 (CH_2);

^{19}F{^{1}H} NMR (377 MHz, $CDCl_3$) δ –126.8 (d, J = 61.2).

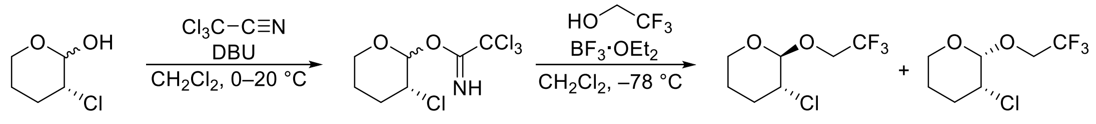

(2*R,3*S**)-3-Chloro-2-(2,2,2-trifluoroethoxy)tetrahydro-2*H*-pyran (*trans*-7d) and (2*R**,3*R**)-3-chloro-2-(2,2,2-trifluoroethoxy)tetrahydro-2*H*-pyran (*cis*-7d)**.

Trichloroacetimidate **5a** was prepared using the representative procedure for trichloroacetimidate formation using hemiacetal **3** (0.205 g, 1.50 mmol), trichloroacetonitrile (1.5 mL, 15 mmol), and DBU (22 µL, 0.15 mmol) in CH_2Cl_2 (15 mL). Acetal **7d** was prepared using the representative procedure for additions of oxygen nucleophiles to α-halo acetals using trichloroacetimidate **5a** (1.50 mmol), 2,2,2-trifluoroethanol (420 µL, 5.84 mmol), and $BF_3{\cdot}OEt_2$ (370 µL, 2.95 mmol) in CH_2Cl_2 (15 mL). ^{1}H NMR and ^{13}C{^{1}H} NMR spectroscopic analysis of the unpurified reaction mixture revealed that pyran **7d** was formed as a 94:6 mixture of diastereomers (*trans*-**7d**:*cis*-**7d**). Purification by flash chromatography (5:95 EtOAc:hexanes) afforded acetal *trans*-**7d** and acetal *cis*-**7d** as a colorless oil (0.135 g, 41%) with a diastereomeric ratio of 98:2. This mixture was used for characterization. The relative stereochemical configurations of the two compounds were assigned by ^{1}H NMR coupling constants; details are provided later in Section VII:

IR (ATR) 2958, 1158, 1058, 977, 874, 735 cm^{-1};

HRMS (APCI) *m / z* calcd for C_5H_8ClO ((M + H) – $C_2H_3F_3O$)$^+$ 119.0258, found 119.0262.

Major Diastereomer *trans*-**7d**:

^{1}H NMR (400 MHz, $CDCl_3$) δ 4.71 (d, J = 3.2, 1H), 4.08–3.82 (m, 4H), 3.66– (dt, J = 11.4, 4.2, 1H), 2.36–2.28 (m, 1H), 2.10–2.00 (m, 1H), 1.91–1.84 (m, 1H), 1.54–1.46 (m, 1H);

^{13}C{^{1}H} NMR (100 MHz, $CDCl_3$) δ 123.7 (br q, $^{1}J_{C-F}$ = 277.7, CF_3), 100.4 (CH), 64.4 (br q, $^{2}J_{C-F}$ = 35.1, CH_2), 61.7 (CH_2), 55.4 (CH), 27.7 (CH_2), 20.8 (CH_2);

^{19}F{^{1}H} NMR (377 MHz, $CDCl_3$) δ –75.19 (s, 1F);

Minor Diastereomer *cis*-**7d**:

^{1}H NMR (400 MHz, $CDCl_3$, diagnostic peaks) δ 4.83 (d, J = 2.5, 1H);

$^{13}C\{^1H\}$ NMR (100 MHz, $CDCl_3$, diagnostic peaks) δ 98.3, 59.6, 56.2.

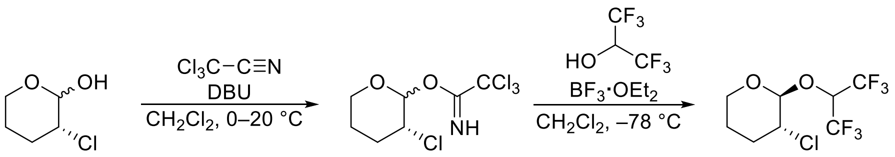

3 5a 7e

(2*R,3*S**)-3-Chloro-2-((1,1,1,3,3,3-hexafluoropropan-2-yl)oxy)tetrahydro-2*H*-pyran (7e)**.

Trichloroacetimidate **5a** was prepared using the representative procedure for trichloroacetimidate formation using hemiacetal **3** (0.205 g, 1.50 mmol), trichloroacetonitrile (1.5 mL, 15 mmol), and DBU (22 µL, 0.15 mmol) in CH_2Cl_2 (15 mL). Acetal **7e** was prepared using the representative procedure for additions of oxygen nucleophiles to α-halo acetals using trichloroacetimidate **5a** (1.50 mmol), hexafluoro-2-propanol (620 µL, 5.90 mmol), and $BF_3·OEt_2$ (370 µL, 2.95 mmol) in CH_2Cl_2 (12 mL). ^1H NMR and $^{13}C\{^1H\}$ NMR spectroscopic analysis of the unpurified reaction mixture revealed that alkene **7e** was formed as a single diastereomer (dr >99:1). Purification by flash chromatography (3:97 EtOAc:hexanes) afforded acetal **7e** as a white solid (0.062 g, 21%). The spectroscopic data (^1H NMR, $^{13}C\{^1H\}$ NMR, $^{19}F\{^1H\}$ NMR, IR, HRMS) are consistent with the data reported in literature.[8]

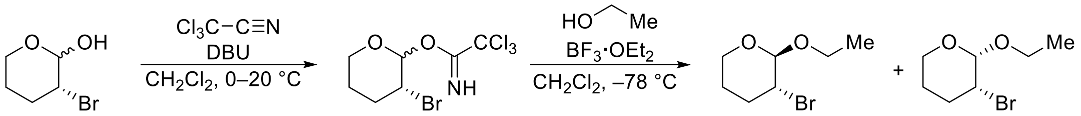

4 6 *trans*-8a *cis*-8a

(2*R,3*R**)-3-Bromo-2-ethoxytetrahydro-2*H*-pyran (*trans*-8a) and (2*R**,3*S**)-3-bromo-2-ethoxytetrahydro-2*H*-pyran (*cis*-8a)**.

Trichloroacetimidate **6** was prepared using the representative procedure for trichloroacetimidate formation using hemiacetal **4** (0.402 g, 2.22 mmol), trichloroacetonitrile (2.2 mL, 22 mmol), and DBU (30 µL, 0.20 mmol) in CH_2Cl_2 (20 mL). Acetal **8a** was prepared using the representative procedure for additions of oxygen nucleophiles to α-halo acetals using trichloroacetimidate **6** (2.22 mmol), ethanol (520 µL, 8.91 mmol), and $BF_3·OEt_2$ (560 µL, 4.46 mmol) in CH_2Cl_2 (20 mL). ^1H NMR and $^{13}C\{^1H\}$ NMR spectroscopic analysis of the unpurified reaction mixture revealed that acetal **8a** was formed as a 93:7 mixture of diastereomers (*trans*-**8a**:*cis*-**8a**). Purification by flash chromatography (5:95 EtOAc:hexanes) afforded acetal *trans*-**8a** and acetal *cis*-**8a** as a colorless oil (0.193 g, 42%) with a diastereomeric ratio of 96:4. This mixture was used for characterization. The relative stereochemical configurations of the two compounds were assigned by ^1H NMR coupling constants; details are provided later in Section VII:

IR (ATR) 2973, 1204, 1131, 1069, 1042, 867 cm^{-1};

HRMS (ESI) *m / z* calcd for C_5H_8BrO ((M + H) – C_2H_6O)$^+$ 162.9753, found 162.9747.

Anal. Calcd for for $C_7H_{13}BrO_2$: C, 40.21; H, 6.27. Found: C, 40.48; H, 6.38.

Major Diastereomer *trans*-**8a**:

^{1}H NMR (400 MHz, $CDCl_3$) δ 4.59 (d, *J* = 4.8, 1H), 3.98–3.90 (m, 2H), 3.87–3.79 (m, 1H), 3.61–3.53 (m, 2H), 2.44–2.35 (m, 1H), 1.99–1.84 (m, 2H), 1.60–1.50 (m, 1H), 1.24 (t, *J* = 7.1, 1H);

^{13}C{^{1}H} NMR (100 MHz, $CDCl_3$) δ 101.2 (CH), 64.0 (CH_2), 62.8 (CH_2), 49.7 (CH), 30.6 (CH_2), 23.7 (CH_2), 15.1 (CH_3).

Minor Diastereomer *cis*-**8a**:

^{1}H NMR (400 MHz, $CDCl_3$, diagnostic peaks) δ 4.71 (dd, *J* = 2.7, 1H), 4.10 (ddd, *J* = 11.5, 4.3, 2.8, 1H), 2.10–2.02 (m, 1H), 1.83–1.69 (m, 1H), 1.27 (t, *J* = 7.1, 1H);

^{13}C{^{1}H} NMR (100 MHz, $CDCl_3$) δ 97.5 (CH), 63.7 (CH_2), 59.3 (CH_2), 49.4 (CH), 29.1 (CH_2), 26.8 (CH_2), 14.9 (CH_3).

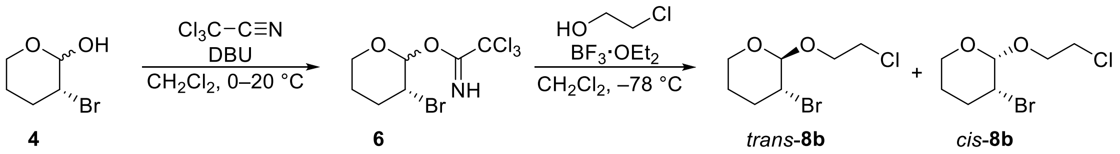

(2*R**,3*S****)-3-Bromo-2-(2-chloroethoxy)tetrahydro-2*H*-pyran (*trans*-8b) and (2*R****,3*R****)-3-bromo-2-(2-chloroethoxy)tetrahydro-2*H*-pyran (*cis*-8b)**.

Trichloroacetimidate **6** was prepared using the representative procedure for trichloroacetimidate formation using hemiacetal **4** (0.403 g, 2.23 mmol), trichloroacetonitrile (2.2 mL, 22 mmol), and DBU (30 µL, 0.20 mmol) in CH_2Cl_2 (20 mL). Acetal **8b** was prepared using the representative procedure for additions of oxygen nucleophiles to α-halo acetals using trichloroacetimidate **6** (2.23 mmol), 2-chloroethanol (600 µL, 8.95 mmol), and $BF_3 \cdot OEt_2$ (560 µL, 4.46 mmol) in CH_2Cl_2 (20 mL). ^{1}H NMR and ^{13}C{^{1}H} NMR spectroscopic analysis of the unpurified reaction mixture revealed that acetal **8b** was formed as a 98:2 mixture of diastereomers (*trans*-**8b**:*cis*-**8b**). Purification by flash chromatography (5:95 EtOAc:hexanes) afforded acetal *trans*-**8b** and acetal *cis*-**8b** as a colorless oil (0.209 g, 38%) with a diastereomeric ratio of 98:2. This mixture was used for characterization. The relative stereochemical configurations of the two compounds were assigned by ^{1}H NMR coupling constants; details are provided later in Section VII:

IR (ATR) 2951, 1131, 1068, 1029, 991, 868 cm^{-1};

HRMS (APCI) *m / z* calcd for $C_7H_{12}ClO_2$ ((M + H) – HBr)$^+$ 163.0520, found 163.0513.

Major Diastereomer *trans*-**8b**:

^{1}H NMR (400 MHz, $CDCl_3$) δ 4.68 (d, *J* = 4.3, 1H), 4.03–3.91 (m, 3H), 3.81–3.75 (m, 1H), 3.68–3.65 (m, 2H), 3.63–3.58 (m, 1H), 2.44–2.37 (m, 1H), 2.00–1.90 (m, 2H), 1.59–1.50 (m, 1H);

^{13}C{^{1}H} NMR (100 MHz, $CDCl_3$) δ 101.3 (CH), 68.4 (CH_2), 62.6 (CH_2), 48.9 (CH), 42.7 (CH_2), 29.8 (CH_2), 23.0 (CH_2).

Minor Diastereomer *cis*-**8b**:

^{1}H NMR (400 MHz, $CDCl_3$, diagnostic peaks) δ 4.77 (d, *J* = 2.7, 1H), 4.10 (ddd, *J* = 11.7, 4.4, 2.9, 1H);

^{13}C{^{1}H} NMR (100 MHz, $CDCl_3$) δ 98.1 (CH), 68.1 (CH_2), 59.5 (CH_2), 48.8 (CH), 42.5 (CH_2), 29.0 (CH_2), 26.8 (CH_2).

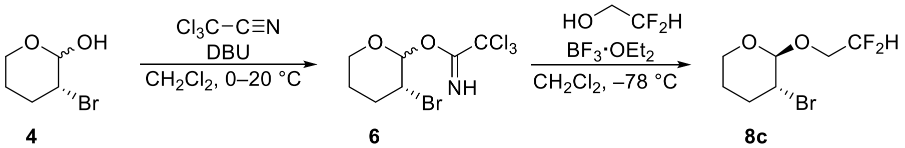

(2*R,3*S**)-3-Bromo-2-(2,2-difluoroethoxy)tetrahydro-2*H*-pyran (7c)**.

Trichloroacetimidate **6** was prepared using the representative procedure for trichloroacetimidate formation using hemiacetal **4** (0.405 g, 2.24 mmol), trichloroacetonitrile (2.2 mL, 22 mmol), and DBU (30 µL, 0.20 mmol) in CH_2Cl_2 (20 mL). Acetal **8c** was prepared using the representative procedure for additions of oxygen nucleophiles to α-halo acetals using trichloroacetimidate **6** (2.24 mmol), 2,2-difluoroethanol (565 µL, 8.92 mmol), and $BF_3\cdot OEt_2$ (560 µL, 4.46 mmol) in CH_2Cl_2 (20 mL). ^{1}H NMR, ^{13}C{^{1}H} NMR, and ^{19}F{^{1}H} NMR spectroscopic analysis of the unpurified reaction mixture revealed that acetal **8c** was formed as a single diastereomer (dr >99:1). Purification by flash chromatography (5:95 EtOAc:hexanes) afforded acetal **8c** as a colorless oil (0.330 g, 60%). The relative stereochemical configuration of acetal **8c** was assigned by ^{1}H NMR coupling constants; details are provided later in Section VII:

^{1}H NMR (400 MHz, $CDCl_3$) δ 6.08–5.78 (m, 1H), 4.69 (d, *J* = 4.3, 1H), 4.02–3.99 (m, 1H), 3.94–3.84 (m, 2H), 3.84–3.71 (m, 1H), 3.64–3.59 (m, 1H), 2.43–2.35 (m, 1H), 2.01–1.91 (m, 2H), 1.59–1.51 (m, 1H);

^{13}C{^{1}H} NMR (100 MHz, $CDCl_3$) δ 114.1 (br t, $^{1}J_{C-F}$ = 241.2, CH), 101.5 (CH), 67.1 (br t, $^{2}J_{C-F}$ = 28.6, CH_2), 62.6 (CH_2), 48.2 (CH), 29.6 (CH_2), 22.9 (CH_2);

^{19}F{^{1}H} NMR (377 MHz, $CDCl_3$) δ –126.2 (s);

IR (ATR) 2954, 1096, 1066, 1049, 954, 868 cm^{-1};

HRMS (APCI) *m / z* calcd for C_5H_8BrO ((M + H) – $C_2H_4F_2O)^+$ 162.9753, found 162.9746.

Anal. Calcd for $C_7H_{11}BrF_2O_2$: C, 34.31; H, 4.52. Found: C, 34.58; H, 4.71.

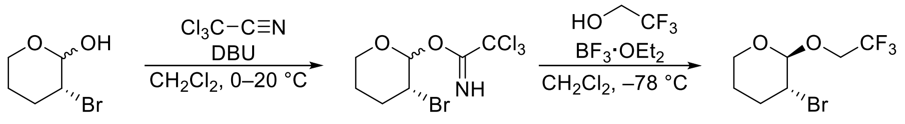

4 6 8d

(2*R,3*S**)-3-Bromo-2-(2,2,2-trifluoroethoxy)tetrahydro-2*H*-pyran (8d)**.

Trichloroacetimidate **6** was prepared using the representative procedure for trichloroacetimidate formation using hemiacetal **4** (0.401 g, 2.22 mmol), trichloroacetonitrile (2.2 mL, 22 mmol), and DBU (30 µL, 0.20 mmol) in CH_2Cl_2 (20 mL). Acetal **8d** was prepared using the representative procedure for additions of oxygen nucleophiles to α-halo acetals using trichloroacetimidate **6** (2.22 mmol), 2,2,2-trifluoroethanol (640 µL, 8.89 mmol), and BF_3·OEt_2 (560 µL, 4.46 mmol) in CH_2Cl_2 (20 mL). ^1H NMR, $^{13}C\{^1H\}$ NMR, and $^{19}F\{^1H\}$ NMR spectroscopic analysis of the unpurified reaction mixture revealed that acetal **8d** was formed as a single diastereomer (dr >99:1). Purification by flash chromatography (5:95 EtOAc:hexanes) afforded acetal **8d** as a colorless oil (0.422 g, 72%). The relative stereochemical configuration of acetal **8d** was assigned by ^1H NMR coupling constants; details are provided later in Section VII:

^1H NMR (400 MHz, $CDCl_3$) δ 4.79 (d, *J* = 3.4, 1H), 4.08–3.85 (m, 4H), 3.67–3.62 (m, 1H), 2.43–2.35 (m, 1H), 2.08–1.92 (m, 2H), 1.53–1.50 (m, 1H);

$^{13}C\{^1H\}$ NMR (100 MHz, $CDCl_3$) δ 123.8 (br q, $^1J_{C–F}$ = 278.3, CF_3), 100.7 (CH), 64.6 (br q, $^2J_{C–F}$ = 35.0, CH_2), 62.3 (CH_2), 47.8 (CH), 28.8 (CH_2), 22.1 (CH_2);

$^{19}F\{^1H\}$ NMR (377 MHz, $CDCl_3$) δ –74.7 (s, 1F), –75.1 (s, 2F);

IR (ATR) 2956, 1278, 1159, 1072, 974, 797 cm^{-1};

HRMS (APCI) *m / z* calcd for $C_7H_{10}F_3O_2$ ((M + H) – HBr)$^+$ 183.0627, found 183.0619.

Anal. Calcd for for $C_7H_{10}BrF_3O_2$: C, 31.96; H, 3.83. Found: C, 32.18; H, 4.02.

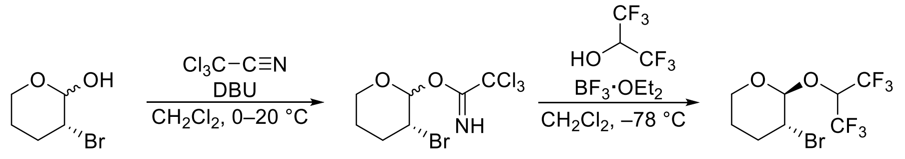

4 6 8e

(2*R,3*S**)-3-Bromo-2-((1,1,1,3,3,3-hexafluoropropan-2-yl)oxy)tetrahydro-2*H*-pyran (8e)**.

Trichloroacetimidate **6** was prepared using the representative procedure for trichloroacetimidate formation using hemiacetal **4** (0.410 g, 2.26 mmol), trichloroacetonitrile (2.2 mL, 22 mmol), and DBU (30 µL, 0.20 mmol) in CH_2Cl_2 (20 mL). Acetal **8e** was prepared using the representative procedure for additions of oxygen nucleophiles to α-halo acetals using trichloroacetimidate **6** (2.26 mmol), hexafluoro-2-propanol (940 µL, 8.95 mmol), and $BF_3·OEt_2$ (560 µL, 4.46 mmol) in CH_2Cl_2 (20 mL). ^{1}H NMR, ^{13}C{^{1}H} NMR, and ^{19}F{^{1}H} NMR spectroscopic analysis of the unpurified reaction mixture revealed that acetal **8e** was formed as a single diastereomer (dr >99:1). Purification by flash chromatography (5:95 EtOAc:hexanes) afforded acetal **8e** as a white solid (0.100 g, 13%). The spectroscopic data (^{1}H NMR, ^{13}C{^{1}H} NMR, ^{19}F{^{1}H} NMR, IR, HRMS) are consistent with the data reported in literature.[8]

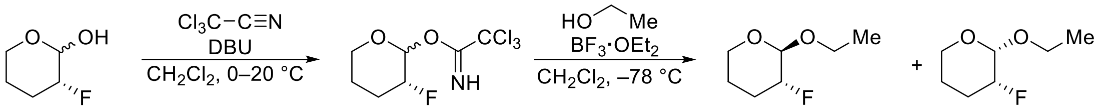

9 **1a** *trans*-**10a** *cis*-**10a**

(2*R**,3*R****)-2-Ethoxy-3-fluorotetrahydro-2*H*-pyran (*trans*-10a) and (2*R****,3*S****)-2-ethoxy-3-fluorotetrahydro-2*H*-pyran (*cis*-10a)**.

Trichloroacetimidate **1a** was prepared using the representative procedure for trichloroacetimidate formation using hemiacetal **9** (0.312 g, 2.60 mmol), trichloroacetonitrile (2.5 mL, 25 mmol), and DBU (40 µL, 0.27 mmol) in CH_2Cl_2 (25 mL). Acetal **10a** was prepared using the representative procedure for additions of oxygen nucleophiles to α-halo acetals using trichloroacetimidate **1a** (2.60 mmol), ethanol (580 µL, 9.93 mmol), and $BF_3·OEt_2$ (630 µL, 5.02 mmol) in CH_2Cl_2 (25 mL). ^{1}H NMR and ^{13}C{^{1}H} NMR spectroscopic analysis of the unpurified reaction mixture revealed that acetal **10a** was formed as a 77:23 mixture of diastereomers (*trans*-**10a**:*cis*-**10a**). Purification by flash chromatography (5:95 EtOAc:hexanes) afforded acetal **10a** as a colorless oil (0.096 g, 26%) with a diastereomeric ratio of 70:30. This mixture was used for characterization. The relative stereochemical configurations of the two compounds were assigned by ^{1}H NMR coupling constants; details are provided later in Section VII:

IR (ATR) 2956, 1078, 1045, 1026, 971, 814 cm^{-1};

HRMS (ESI) *m / z* calcd for $C_7H_{14}FO_2$ $(M + H)^+$ 149.0972, found 149.0972.

Major Diastereomer *trans*-**10a**:

^{1}H NMR (400 MHz, $CDCl_3$) δ 4.66 (dd, J = 6.8, 3.2, 1H), 4.45–4.30 (m, 1H), 3.83–3.0 (m, 2H), 3.59–3.52 (m, 2H), 2.07–1.84 (m, 3H), 1.47–1.42 (m, 1H), 1.24 (t, J = 7.1, 3H);

^{13}C{^{1}H} NMR (100 MHz, $CDCl_3$) δ 97.6 (br d, $^2J_{C–F}$ = 29.3, CH), 86.9 (br d, $^1J_{C–F}$ = 173.0, CH), 63.32 (CH_2), 60.8 (CH_2), 25.0 (br d, $^2J_{C–F}$ = 20.0, CH_2), 20.9 (br d, $^3J_{C–F}$ = 3.0, CH_2), 15.1 (CH_3);

$^{19}F\{^1H\}$ NMR (377 MHz, $CDCl_3$) δ –191.2 (s).

Minor Diastereomer *cis*-**10a**:

^1H NMR (400 MHz, $CDCl_3$, diagnostic peaks) δ 4.81 (t, J = 3.2, 1H), 4.53 (dddd, J = 48.2, 10.8, 4.7, 3.2, 1H), 3.74 (td, J = 11.3, 3.2, 1H), 1.28 (t, J = 7.0, 3H);

$^{13}C\{^1H\}$ NMR (100 MHz, $CDCl_3$, diagnostic peaks) 96.2 (br d, $^2J_{C–F}$ = 20.5, CH), 87.4 (br d, $^1J_{C–F}$ = 184.8, CH), 63.34 (CH_2), 59.4 (CH_2), 14.1 (CH_3);

$^{19}F\{^1H\}$ NMR (377 MHz, $CDCl_3$) δ –189.92 (br s).

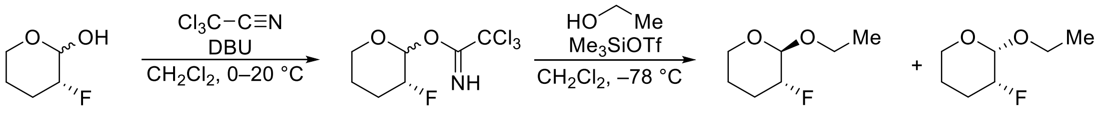

(2*R,3*R**)-2-ethoxy-3-fluorotetrahydro-2*H*-pyran (*trans*-10a) and (2*R**,3*S**)-2-ethoxy-3-fluorotetrahydro-2*H*-pyran (*cis*-10a).**

Trichloroacetimidate **1a** was prepared using the representative procedure for trichloroacetimidate formation using hemiacetal **9** (0.152 g, 1.27 mmol), trichloroacetonitrile (1.3 mL, 1.3 mmol), and DBU (20 µL, 0.13 mmol) in CH_2Cl_2 (12 mL). Acetal **10a** was prepared using the representative procedure for additions of oxygen nucleophiles to α-halo acetals using trichloroacetimidate **1a** (1.27 mmol), ethanol (290 µL, 4.97 mmol), and Me_3SiOTf (450 µL, 2.49 mmol) in CH_2Cl_2 (10 mL). ^1H NMR and $^{13}C\{^1H\}$ NMR spectroscopic analysis of the unpurified reaction mixture revealed that acetal **10a** was formed as a 81:19 mixture of diastereomers (*trans*-**10a**:*cis*-**10a**). The spectroscopic data are consistent with those of the same acetal prepared from trichloroacetimidate **1a** and $BF_3·OEt_2$.

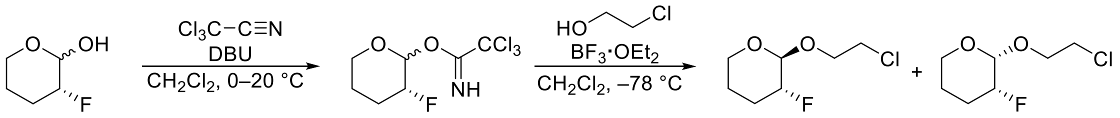

(2*R,3*S**)-2-(2-Chloroethoxy)-3-fluorotetrahydro-2*H*-pyran (*trans*-10b) and (2*R**,3*R**)-2-(2-chloroethoxy)-3-fluorotetrahydro-2*H*-pyran (*cis*-10b).**

Trichloroacetimidate **1a** was prepared using the representative procedure for trichloroacetimidate formation using hemiacetal **9** (0.305 g, 2.54 mmol), trichloroacetonitrile (2.5 mL, 25 mmol), and DBU (40 µL, 0.27 mmol) in CH_2Cl_2 (25 mL). Acetal **10b** was prepared using the representative procedure for additions of oxygen nucleophiles to α-halo acetals using trichloroacetimidate **1a** (2.54 mmol), 2-chloroethanol (680 µL, 10.2 mmol), and $BF_3·OEt_2$ (630 µL, 5.02 mmol) in CH_2Cl_2 (25 mL). ^1H NMR and

$^{13}C\{^1H\}$ NMR spectroscopic analysis of the unpurified reaction mixture revealed that acetal **10b** was formed as a 65:35 mixture of diastereomers (*trans*-**10b**:*cis*-**10b**). Purification by flash chromatography (5:95 EtOAc:hexanes) afforded the major diastereomer **10b** as a colorless oil (0.197 g, 42%) and a mixture of acetal *trans*-**10b** and acetal *cis*-**10b** as a colorless oil (0.062 g, 13%) with a diastereomeric ratio of 22:78. The relative stereochemical configurations of the two compounds were assigned by ^1H NMR coupling constants; details are provided later in Section VII:

Major Diastereomer *trans*-**10b**:

^1H NMR (400 MHz, $CDCl_3$) δ 4.73 (dd, J = 6.9, 2.7, 1H), 4.50–4.36 (m, 1H), 4.02–3.97 (m, 1H), 3.90–3.84 (m, 1H), 3.80–3.74 (m, 1H), 3.69–3.66 (m, 2H), 3.61–3.56 (m, 1H), 2.10–1.87 (m, 3H), 1.49–1.40 (m, 1H);

$^{13}C\{^1H\}$ NMR (100 MHz, $CDCl_3$) δ 97.7 (br d, $^2J_{C-F}$ = 30.0, CH), 86.3 (br d, $^1J_{C-F}$ = 172.1, CH), 67.8 (CH_2), 60.7 (CH_2), 42.8 (CH_2), 24.6 (br d, $^2J_{C-F}$ = 20.2, CH_2), 20.4 (br d, $^3J_{C-F}$ = 2.5, CH_2);

$^{19}F\{^1H\}$ NMR (377 MHz, $CDCl_3$) δ –191.70 (m);

IR (ATR) 2957, 1146, 1117, 1037, 967, 878 cm^{-1};

HRMS (ESI) *m / z* calcd for C_5H_8FO ((M + H) – $C_2H_5ClO)^+$ 103.0554, found 103.0555.

Minor Diastereomer *cis*-**10b**:

^1H NMR (400 MHz, $CDCl_3$, diagnostic peaks) δ 4.86 (t, J = 3.0, 1H), 4.53 (dddd, J = 47.9, 10.8, 4.7, 3.2, 1H), 3.73–3.69 (m, 2H), 3.54–3.48 (m, 1H), 1.81–1.64 (m, 2H);

$^{13}C\{^1H\}$ NMR (100 MHz, $CDCl_3$) δ 96.8 (br d, $^2J_{C-F}$ = 20.9, CH), 87.6 (br d, $^1J_{C-F}$ = 184.7, CH), 68.1 (CH_2), 59.7 (CH_2), 42.7 (CH_2), 24.4 (br d, $^2J_{C-F}$ = 18.9, CH_2), 23.8 (br d, $^3J_{C-F}$ = 8.2, CH_2);

$^{19}F\{^1H\}$ NMR (377 MHz, $CDCl_3$) δ –189.86 (m).

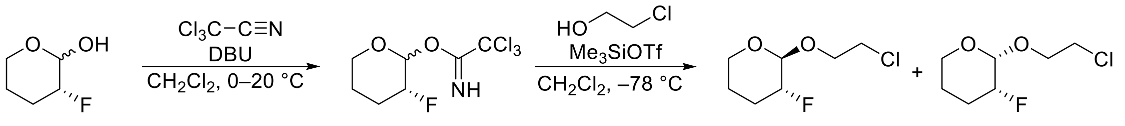

9 1a *trans*-**10b** *cis*-**10b**

(2*R,3*S**)-2-(2-Chloroethoxy)-3-fluorotetrahydro-2*H*-pyran (*trans*-10b) and (2*R**,3*R**)-2-(2-chloroethoxy)-3-fluorotetrahydro-2*H*-pyran (*cis*-10b)**.

Trichloroacetimidate **1a** was prepared using the representative procedure for trichloroacetimidate formation using hemiacetal **9** (0.250 g, 2.08 mmol), trichloroacetonitrile (2.5 mL, 25 mmol), and DBU (40 µL, 0.27 mmol) in CH_2Cl_2 (25 mL). Acetal **10b** was prepared using the representative procedure for additions of oxygen nucleophiles to α-halo acetals using trichloroacetimidate **1a** (2.08 mmol), 2-

chloroethanol (560 µL, 8.35 mmol), and Me_3SiOTf (750 µL, 4.15 mmol) in CH_2Cl_2 (21 mL). ^1H NMR and $^{13}C\{^1H\}$ NMR spectroscopic analysis of the unpurified reaction mixture revealed that acetal **10b** was formed as a 75:25 mixture of diastereomers (*trans*-**10b**:*cis*-**10b**). Purification by flash chromatography (5:95 EtOAc:hexanes) afforded the major diastereomer *trans*-**10b** as a colorless oil (0.196 g, 52%) and a mixture of acetal *trans*-**10b** and acetal *trans*-**10b** as a colorless oil (0.062 g, 16%) with a diastereomeric ratio of 28:72. The spectroscopic data are consistent with those of the same acetal prepared from trichloroacetimidate **1a** and $BF_3{\cdot}OEt_2$.

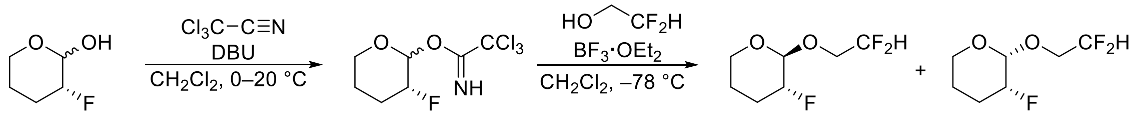

(2*R,3*S**)-2-(2,2-Difluoroethoxy)-3-fluorotetrahydro-2*H*-pyran (*trans*-10c) and (2*R**,3*R**)-2-(2,2-difluoroethoxy)-3-fluorotetrahydro-2*H*-pyran (*cis*-10c)**.

Trichloroacetimidate **1a** was prepared using the representative procedure for trichloroacetimidate formation using hemiacetal **9** (0.295 g, 2.46 mmol), trichloroacetonitrile (2.5 mL, 25 mmol), and DBU (40 µL, 0.27 mmol) in CH_2Cl_2 (25 mL). Acetal **10c** was prepared using the representative procedure for additions of oxygen nucleophiles to α-halo acetals using trichloroacetimidate **1a** (2.46 mmol), 2,2-difluoroethanol (630 µL, 9.95 mmol), and $BF_3{\cdot}OEt_2$ (630 µL, 5.02 mmol) in CH_2Cl_2 (25 mL). ^1H NMR, $^{13}C\{^1H\}$ NMR, and $^{19}F\{^1H\}$ NMR spectroscopic analysis of the unpurified reaction mixture revealed that acetal **10c** was formed as a 67:33 mixture of diastereomers (*trans*-**10c**:*cis*-**10c**). Purification by flash chromatography (5:95 EtOAc:hexanes) afforded the major diastereomer *trans*-**10c** as a colorless oil (0.167 g, 37%) and a mixture of acetal *trans*-**10c** and acetal *cis*-**10c** as a colorless oil (0.092 g, 20%) with a diastereomeric ratio of 22:78. The relative stereochemical configurations of the two compounds were assigned by ^1H NMR coupling constants; details are provided later in Section VII:

Major Diastereomer *trans*-**10c**:

^1H NMR (400 MHz, $CDCl_3$) δ 6.07–5.77 (m, 1H), 4.73 (dd, J = 6.9, 2.7, 1H), 4.51–4.37 (m, 1H), 3.96–3.69 (m, 3H), 3.65–3.57 (m, 1H), 2.06–1.88 (m, 3H), 1.49–1.42 (m 1H);

$^{13}C\{^1H\}$ NMR (100 MHz, $CDCl_3$) δ 114.1 (br t, $^1J_{C–F}$ = 241.0, CH), 98.1–97.8 (br d, $^2J_{C–F}$ = 30.6, CH), 86.8–85.1 (br d, $^1J_{C–F}$ = 172.3, CH), 66.7 (br t, $^2J_{C–F}$ = 28.3, CH_2), 60.8 (CH_2), 24.6–24.4 (br d, $^2J_{C–F}$ = 20.0, CH_2), 20.3–20.2 (d, $^3J_{C–F}$ = 2.5, CH_2);

$^{19}F\{^1H\}$ NMR (377 MHz, $CDCl_3$) δ –126.2 (d, J = 4.6, 2F), –191.9 (s, 1F);

IR (ATR) 2958, 1130, 1081, 974, 823, 800 cm^{-1};

HRMS (APCI) *m / z* calcd for C_5H_8FO ((M + H) – $C_2H_4F_2O)^+$ 103.0554, found 103.0553.

Minor Diastereomer *cis*-**10c**:

^{1}H NMR (400 MHz, $CDCl_3$, diagnostic peaks) δ 6.12–5.82 (m, 1H), 4.86 (t, *J* = 2.9, 1H), 4.54 (dddd, *J* = 47.7, 10.9, 4.8, 3.2, 1H), 3.55–3.50 (m, 1H), 1.81–1.67 (m, 2H);

$^{13}C\{^{1}H\}$ NMR (100 MHz, $CDCl_3$, diagnostic peaks) δ 97.0 (br d, $^{2}J_{C–F}$ = 21.1, CH), 87.3 (br d, $^{1}J_{C–F}$ = 185.1, CH), 66.8 (br t, $^{2}J_{C–F}$ = 30.8, CH_2), 59.7 (CH_2), 24.2 (br d, $^{2}J_{C–F}$ = 19.1, CH_2), 23.6 (d, $^{3}J_{C–F}$ = 8.2, CH_2);

$^{19}F\{^{1}H\}$ NMR (377 MHz, $CDCl_3$) δ –126.0 (s, 1F), –126.1 (s, 1F), –190.2 (br s, 1F).

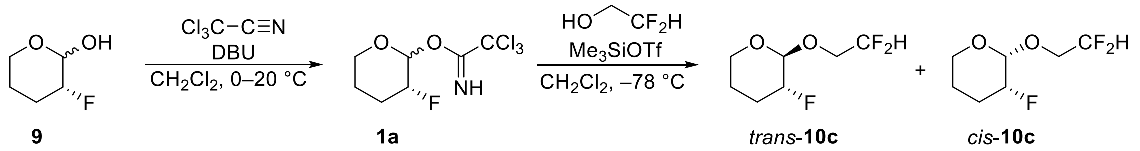

(2*R,3*S**)-2-(2,2-Difluoroethoxy)-3-fluorotetrahydro-2*H*-pyran (*trans*-10c) and (2*R**,3*R**)-2-(2,2-difluoroethoxy)-3-fluorotetrahydro-2*H*-pyran (*cis*-10c)**.

Trichloroacetimidate **1a** was prepared using the representative procedure for trichloroacetimidate formation using hemiacetal **9** (0.158 g, 1.32 mmol), trichloroacetonitrile (1.3 mL, 1.3 mmol), and DBU (20 µL, 0.13 mmol) in CH_2Cl_2 (12 mL). Acetal **10c** was prepared using the representative procedure for additions of oxygen nucleophiles to α-halo acetals using trichloroacetimidate **1a** (1.32 mmol), 2,2-difluoroethanol (320 µL, 5.05 mmol), and Me_3SiOTf (450 µL, 2.49 mmol) in CH_2Cl_2 (10 mL). ^{1}H NMR and $^{13}C\{^{1}H\}$ NMR spectroscopic analysis of the unpurified reaction mixture revealed that acetal **10c** was formed as a 76:24 mixture of diastereomers (*trans*-**10c**:*cis*-**10c**). The spectroscopic data are consistent with those of the same acetal prepared from trichloroacetimidate **1a** and BF_3·OEt_2.

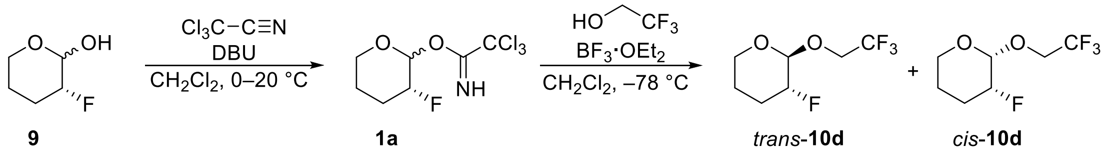

(2*R,3*S**)-3-Fluoro-2-(2,2,2-trifluoroethoxy)tetrahydro-2*H*-pyran (*trans*-10d) and (2*R**,3*R**)-3-fluoro-2-(2,2,2-trifluoroethoxy)tetrahydro-2*H*-pyran (*cis*-10d)**.

Trichloroacetimidate **1a** was prepared using the representative procedure for trichloroacetimidate formation using hemiacetal **9** (0.308 g, 2.56 mmol), trichloroacetonitrile (2.5 mL, 25 mmol), and DBU (40 µL, 0.27 mmol) in CH_2Cl_2 (25 mL). Acetal **10d** was prepared using the representative procedure for additions of oxygen nucleophiles to α-halo acetals using trichloroacetimidate **1a** (2.56 mmol), 2,2,2-trifluoroethanol (720 µL, 10.0 mmol), and BF_3·OEt_2 (630 µL, 5.02 mmol) in CH_2Cl_2 (25 mL). ^{1}H NMR,

$^{13}C\{^1H\}$ NMR, and $^{19}F\{^1H\}$ NMR spectroscopic analysis of the unpurified reaction mixture revealed that acetal **10d** was formed as a 61:39 mixture of diastereomers (*trans*-**10d**:*cis*-**10d**). Purification by flash chromatography (5:95 EtOAc:hexanes) afforded the major diastereomer *trans*-**10d** as a colorless oil (0.113 g, 22%) and the minor diastereomer *cis*-**10d** as colorless oil (0.088 g, 17%). The relative stereochemical configurations of the two compounds were assigned by ^1H NMR coupling constants; details are provided later in Section VII:

Major Diastereomer *trans*-**10d**:

^1H NMR (400 MHz, $CDCl_3$) δ 4.81 (dd, J = 7.0, 2.1, 1H), 4.55–4.41 (m, 1H), 4.09–4.00 (m, 1H), 3.96–3.87 (m, 1H), 3.84–3.77 (m, 1H), 3.66–3.61 (m, 1H), 2.06–1.91 (m, 3H), 1.47–1.42 (m, 1H);

$^{13}C\{^1H\}$ NMR (100 MHz, $CDCl_3$) δ 123.77 (br q, $^1J_{C–F}$ = 275.5, CF_3), 97.3 (br d, $^2J_{C–F}$ = 31.8, CH), 85.5 (br d, $^1J_{C–F}$ = 171.9, CH), 64.1 (br q, $^2J_{C–F}$ = 35.0, CH_2), 60.6 (CH_2), 24.0 (br d, $^2J_{C–F}$ = 19.8, CH_2), 19.8 (br d, $^3J_{C–F}$ = 1.9, CH_2);

$^{19}F\{^1H\}$ NMR (377 MHz, $CDCl_3$) δ –75.3 (3F), –192.4 (1F);

IR (ATR) 2959, 1278, 1156, 1059, 972, 799 cm^{-1};

HRMS (ESI) m / z calcd for C_5H_8FO ((M + H) – $C_2H_3F_3O)^+$ 103.0554, found 103.0556.

Minor Diastereomer *cis*-**10d**:

^1H NMR (400 MHz, $CDCl_3$) δ 4.91 (t, J = 2.7, 1H), 4.75 (dddd, J = 47.8, 11.0, 4.8, 3.1, 1H), 4.08–3.93 (m, 2H), 3.73 (td, J = 11.3, 3.2, 1H), 3.57–3.51 (m, 1H), 2.13–2.01 (m, 1H), 1.98–1.90 (m, 1H), 1.82–1.69 (m, 2H);

$^{13}C\{^1H\}$ NMR (100 MHz, $CDCl_3$) δ 123.8 (br d, $^1J_{C–F}$ = 278.9, CF_3), 96.8 (br d, $^2J_{C–F}$ = 21.2, CH), 87.1 (br d, $^1J_{C–F}$ = 186.2, CH), 64.6 (br q, $^2J_{C–F}$ = 34.7, CH_2), 59.8 (CH_2), 24.1 (br d, $^2J_{C–F}$ = 19.0, CH_2), 23.6 (br d, $^3J_{C–F}$ = 8.2, CH_2);

$^{19}F\{^1H\}$ NMR (377 MHz, $CDCl_3$) δ –75.2 (s, 3F), –190.5 (br s, 1F).

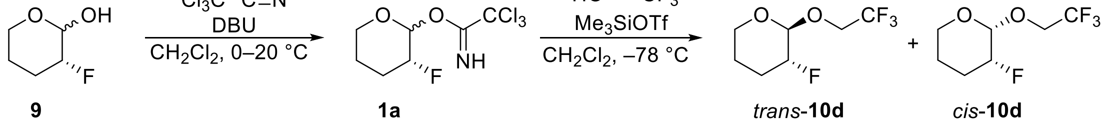

(2*R**,3*S****)-3-Fluoro-2-(2,2,2-trifluoroethoxy)tetrahydro-2*H*-pyran (*trans*-10d) and (2*R****,3*R****)-3-fluoro-2-(2,2,2-trifluoroethoxy)tetrahydro-2*H*-pyran (*cis*-10d)**.

Trichloroacetimidate **1a** was prepared using the representative procedure for trichloroacetimidate formation using hemiacetal **9** (0.152 g, 1.27 mmol), trichloroacetonitrile (1.3 mL, 1.3 mmol), and DBU

(20 µL, 0.13 mmol) in CH_2Cl_2 (12 mL). Acetal **10d** was prepared using the representative procedure for additions of oxygen nucleophiles to α-halo acetals using trichloroacetimidate **1a** (1.27 mmol), 2,2,2-trifluoroethanol (360 µL, 5.00 mmol), and Me_3SiOTf (450 µL, 2.49 mmol) in CH_2Cl_2 (10 mL). ^{1}H NMR and ^{13}C{^{1}H} NMR spectroscopic analysis of the unpurified reaction mixture revealed that acetal **10d** was formed as a 76:24 mixture of diastereomers (*trans*-**10d**:*cis*-**10d**). The spectroscopic data are consistent with those of the same acetal prepared from trichloroacetimidate **1a** and $BF_3·OEt_2$.

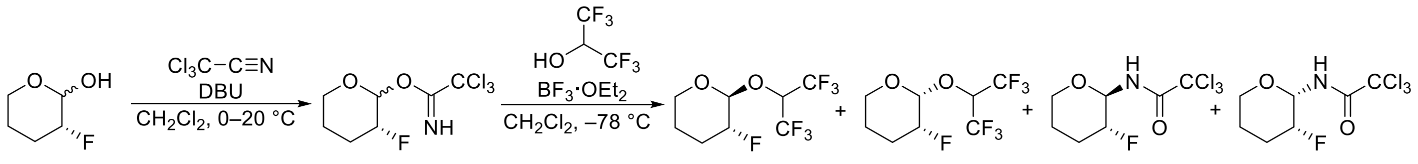

(2*R,3*S**)-3-Fluoro-2-((1,1,1,3,3,3-hexafluoropropan-2-yl)oxy)tetrahydro-2*H*-pyran (*trans*-10e), (2*R**,3*R**)-3-fluoro-2-((1,1,1,3,3,3-hexafluoropropan-2-yl)oxy)tetrahydro-2*H*-pyran (*cis*-10e), 2,2,2-trichloro-*N*-((2*R**,3*R**)-3-fluorotetrahydro-2*H*-pyran-2-yl)acetamide (*trans*-S3). and 2,2,2-trichloro-*N*-((2*R**,3*S**)-3-fluorotetrahydro-2*H*-pyran-2-yl)acetamide (*cis*-S3).**

Trichloroacetimidate **1a** was prepared using the representative procedure for trichloroacetimidate formation using hemiacetal **9** (0.354 g, 2.95 mmol), trichloroacetonitrile (2.9 mL, 29 mmol), and DBU (43 µL, 0.29 mmol) in CH_2Cl_2 (25 mL). Acetal **10e** was prepared using the representative procedure for additions of oxygen nucleophiles to α-halo acetals using trichloroacetimidate **1a** (2.92 mmol), hexafluoro-2-propanol (1.8 mL, 17 mmol), and $BF_3·OEt_2$ (730 µL, 5.81 mmol) in CH_2Cl_2 (25 mL). ^{1}H NMR, ^{13}C{^{1}H} NMR, and ^{19}F{^{1}H} NMR spectroscopic analysis of the unpurified reaction mixture revealed that acetal **10e** was formed as a 43:57 mixture of diastereomers (*trans*-**10e**:*cis*-**10e**). Analysis of the ^{1}H NMR and ^{13}C{^{1}H} NMR spectra of the unpurified reaction mixture also revealed the formation of amide **S3** as a 39:61 mixture of diastereomers (*trans*-**S3**:*cis*-**S3**). Purification by flash chromatography (5:95 EtOAc:hexanes) afforded the major diastereomer *cis*-**10e** as a white solid (0.089 g, 11%), the minor diastereomer *trans*-**10e** as a white solid (0.026 g, 3%), amide *cis*-**S3** as a white solid (0.049 g, 6%), and a 13:87 mixture of amide *trans*-**S3** and trichloroacetamide as a white solid (0.207 g). The spectroscopic data for acetal **10e** (^{1}H NMR, ^{13}C{^{1}H} NMR, ^{19}F{^{1}H} NMR, IR, HRMS) are consistent with the data reported in literature.[8] A 13:87 mixture of amide *trans*-**S3** and trichloroacetamide was used for the characterization of amide *trans*-**S3**. X-ray quality crystals of amide *cis*-**S3** were grown by the slow evaporation of a solution of amide *cis*-**S3** in MeOH. The relative stereochemical configuration of amide *cis*-**S3** was assigned by X-ray crystallographic analysis:

Major Diastereomer amide *cis*-**S3**:

mp = 72–74 °C;

^{1}H NMR (400 MHz, $CDCl_3$) δ 7.40 (br s, 1H), 5.16 (ddd, J = 22.3, 9.1, 0.6, 1H), 4.66 (m, 1H), 4.10–4.05 (m, 1H), 3.70–3.64 (m, 1H), 2.33–2.25 (m, 1H), 1.99–1.92 (m, 1H), 1.90–1.73 (m, 1H), 1.52–1.46 (m, 1H);

^{13}C{^{1}H} NMR (100 MHz, $CDCl_3$) δ 161.5 (C), 92.1 (C), 87.7 (br d, $^1J_{C–F}$ = 177.8, CH), 78.9 (br d, $^2J_{C–F}$ = 16.6, CH), 67.1 (CH_2), 27.7 (br d, $^2J_{C–F}$ = 20.6, CH_2), 19.1 (CH_2);

^{19}F{^{1}H} NMR (377 MHz, $CDCl_3$) δ –207.0 (s);

IR (ATR) 1706, 1528, 1215, 1083, 823, 708 cm^{-1};

HRMS (ESI) m / z calcd for $C_7H_{10}Cl_3FNO_2$ (M + H)$^+$ 263.9756, found 263.9761.

Minor Diastereomer amide *trans*-**S3**:

mp = 111–117 °C;

^{1}H NMR (400 MHz, $CDCl_3$) δ 7.11 (br s, 1H), 5.05 (td, J = 8.7, 2.5, 1H), 4.43–4.42 m, 1H), 4.00–3.96 (m, 1H), 3.59 (td, J = 11.6, 2.5, 1H), 2.39–2.33 (m, 1H), 1.83–1.75 (m, 3H);

^{13}C{^{1}H} NMR (100 MHz, $CDCl_3$) δ 162.0 (C), 91.8 (C), 88.2 (br d, $^1J_{C–F}$ = 184.6, CH), 81.4 (br d, $^2J_{C–F}$ = 24.5, CH), 67.0 (CH_2), 29.4 (br d, $^2J_{C–F}$ = 18.9, CH_2), 24.3 (br d, $^3J_{C–F}$ = 8.9,CH_2);

^{19}F{^{1}H} NMR (377 MHz, $CDCl_3$) δ –186.5 (s).

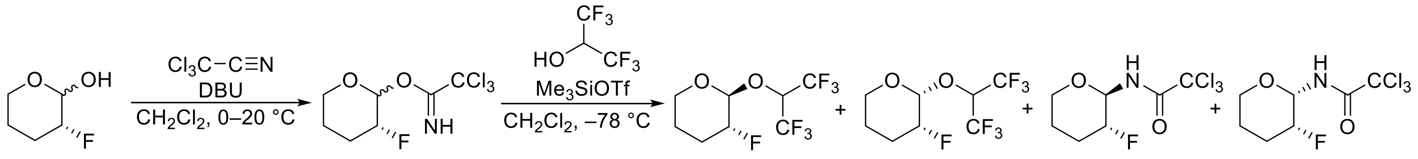

9 1a *trans*-10e *cis*-10e *trans*-S3 *cis*-S3

(2*R,3*S**)-3-Fluoro-2-((1,1,1,3,3,3-hexafluoropropan-2-yl)oxy)tetrahydro-2*H*-pyran (*trans*-10e), (2*R**,3*R**)-3-fluoro-2-((1,1,1,3,3,3-hexafluoropropan-2-yl)oxy)tetrahydro-2*H*-pyran (*cis*-10e), 2,2,2-trichloro-*N*-((2*R**,3*R**)-3-fluorotetrahydro-2*H*-pyran-2-yl)acetamide (*trans*-S3). and 2,2,2-trichloro-*N*-((2*R**,3*S**)-3-fluorotetrahydro-2*H*-pyran-2-yl)acetamide (*cis*-S3).**

Trichloroacetimidate **1a** was prepared using the representative procedure for trichloroacetimidate formation using hemiacetal **9** (0.146 g, 1.22 mmol), trichloroacetonitrile (1.3 mL, 1.3 mmol), and DBU (20 µL, 0.13 mmol) in CH_2Cl_2 (12 mL). Acetal **10e** was prepared using the representative procedure for additions of oxygen nucleophiles to α-halo acetals using trichloroacetimidate **1a** (1.22 mmol), hexafluoro-2-propanol (530 µL, 5.03 mmol), and Me_3SiOTf (450 µL, 2.49 mmol) in CH_2Cl_2 (10 mL). ^{1}H NMR and ^{13}C{^{1}H} NMR spectroscopic analysis of the unpurified reaction mixture revealed that acetal **10e** was formed as a 41:59 mixture of diastereomers (*trans*-**10e**:*cis*-**10e**), and amide **S3** was formed as

a 53:47 mixture of diastereomers (*trans*-**S3**:*cis*-**S3**). The spectroscopic data are consistent with those of the same acetal prepared from trichloroacetimidate **1a** and BF_3·OEt_2.

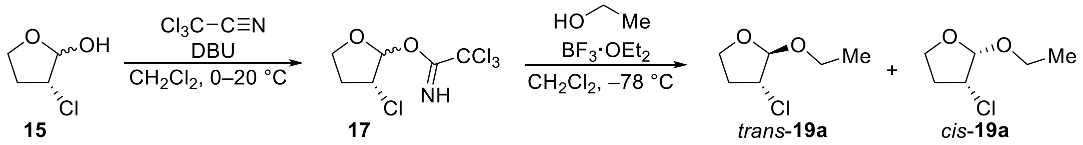

(2*R,3*R**)-3-Chloro-2-ethoxytetrahydrofuran (*trans*-19a) and (2*R**,3*S**)-3-chloro-2-ethoxytetrahydrofuran (*cis*-19a)**.

Trichloroacetimidate **17** was prepared using the representative procedure for trichloroacetimidate formation using hemiacetal **15** (0.308 g, 2.51 mmol), trichloroacetonitrile (2.5 mL, 25 mmol), and DBU (40 µL, 0.27 mmol) in CH_2Cl_2 (25 mL). Acetal **19a** was prepared using the representative procedure for additions of oxygen nucleophiles to α-halo acetals using trichloroacetimidate **17** (2.51 mmol), ethanol (575 µL, 9.85 mmol), and BF_3·OEt_2 (620 µL, 4.94 mmol) in CH_2Cl_2 (25 mL). ^1H NMR and $^{13}C\{^1H\}$ NMR spectroscopic analysis of the unpurified reaction mixture revealed that acetal **19a** was formed as a 59:41 mixture of diastereomers (*trans*-**19a**:*cis*-**19a**). Purification by flash chromatography (5:95 EtOAc:hexanes) afforded the major diastereomer *trans*-**19a** as a colorless oil (0.108 g, 29%) and the minor diastereomer *cis*-**19a** as a colorless oil (0.077 g, 20%). The relative stereochemical configurations of the two compounds were assigned by spectroscopic correlation to acetal **19e**; details are provided later in Section VII:

Major Diastereomer *trans*-**19a**:

^1H NMR (400 MHz, $CDCl_3$) δ 5.10 (s, 1H), 4.22 (dd, J = 5.8, 1.5, 1H), 4.15 (dt, J = 8.1, 8.1, 7.4, 1H), 4.05 (td, J = 12.7, 3.6, 1H), 3.76–3.68 (m, 1H), 3.52–3.44 (m, 1H), 2.58–2.49 (m, 1H), 2.14–2.08 (m, 1H), 1.19 (t, J = 7.1, 3H);

$^{13}C\{^1H\}$ NMR (100 MHz, $CDCl_3$) δ 108.1 (CH), 66.4 (CH_2), 63.0 (CH_2), 60.5 (CH), 33.5 (CH_2), 15.1 (CH_3);

IR (ATR) 2977, 1099, 1077, 1036, 1016, 912 cm^{-1};

HRMS (ESI) *m / z* calcd for C_4H_6ClO ((M + H) – C_2H_6O)$^+$ 105.0102, found 105.0102.

Minor Diastereomer *cis*-**19a**:

^1H NMR (400 MHz, $CDCl_3$) δ 4.99 (d, J = 4.1, 1H), 4.12–4.04 (m, 2H), 3.89 (q, J = 7.9, 1H), 3.79 (dq, J = 9.8, 7.1, 1H), 3.60 (dq, J = 9.8, 7.1, 1H), 2.45–2.37 (m, 1H), 2.28–2.19 (m, 1H), 1.26 (t, J = 7.1, 3H);

$^{13}C\{^1H\}$ NMR (100 MHz, $CDCl_3$) δ 101.1 (CH), 65.2 (CH_2), 63.6 (CH_2), 56.0 (CH), 32.1 (CH_2), 15.1 (CH_3).

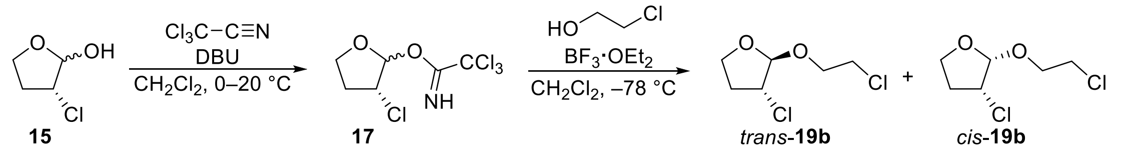

(2*R,3*S**)-3-Chloro-2-(2-chloroethoxy)tetrahydrofuran (*trans*-19b) and (2*R**,3*R**)-3-chloro-2-(2-chloroethoxy)tetrahydrofuran (*cis*-19b)**.

Trichloroacetimidate **17** was prepared using the representative procedure for trichloroacetimidate formation using hemiacetal **15** (0.311 g, 2.54 mmol), trichloroacetonitrile (2.5 mL, 25 mmol), and DBU (40 µL, 0.27 mmol) in CH_2Cl_2 (25 mL). Acetal **19b** was prepared using the representative procedure for additions of oxygen nucleophiles to α-halo acetals using trichloroacetimidate **17** (2.54 mmol), 2-chloroethanol (660 µL, 9.85 mmol), and BF_3·OEt_2 (620 µL, 4.94 mmol) in CH_2Cl_2 (25 mL). ^{1}H NMR and ^{13}C{^{1}H} NMR spectroscopic analysis of the unpurified reaction mixture revealed that acetal **19b** was formed as a 64:36 mixture of diastereomers (*trans*-**19b**:*cis*-**19b**). Purification by flash chromatography (5:95 EtOAc:hexanes) afforded the major diastereomer *trans*-**19b** as a colorless oil (0.284 g, 60%) and the minor diastereomer *trans*-**19b** as a colorless oil (0.155 g, 33%). The relative stereochemical configurations of the two compounds were assigned by spectroscopic correlation to acetal **19e**; details are provided later in Section VII:

Major Diastereomer *trans*-**19b**:

^{1}H NMR (400 MHz, $CDCl_3$) δ 5.15 (s, 1H), 4.28 (dd, *J* = 5.8, 1.2, 1H), 4.21–4.13 (m, 1H), 4.11–4.05 (m, 1H), 3.94–3.88 (m, 1H), 3.74–3.67 (m, 1H), 3.63–3.60 (m, 2H), 2.60–2.51 (m, 1H), 2.16–2.10 (m, 1H);

^{13}C{^{1}H} NMR (100 MHz, $CDCl_3$) δ 108.3 (CH), 67.3 (CH_2), 66.8 (CH_2), 60.2 (CH), 42.9 (CH_2), 33.3 (CH_2);

IR (ATR) 2901, 1107, 1085, 1030, 1007, 923 cm^{-1};

HRMS (ESI) *m / z* calcd for C_4H_6ClO ((M + H) – $C_2H_5ClO)^+$ 105.0102, found 105.0101.

Minor Diastereomer *cis*-**19b**:

^{1}H NMR (400 MHz, $CDCl_3$) δ 5.02 (d, *J* = 4.0, 1H), 4.13–4.07 (m, 2H), 3.97–3.89 (m, 2H), 3.86–3.80 (m, 1H), 3.69–3.66 (m, 2H), 2.46–2.38 (m, 1H), 2.28–2.20 (m, 1H);

^{13}C{1H} NMR (100 MHz, $CDCl_3$) δ 101.5 (CH), 67.9 (CH_2), 65.7 (CH_2), 55.8 (CH), 42.6 (CH_2), 31.8 (CH_2).

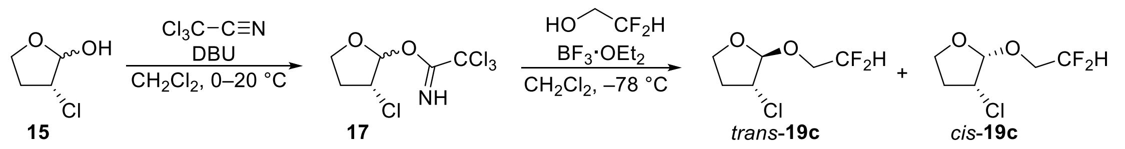

(2*R,3*S**)-3-Chloro-2-(2,2-difluoroethoxy)tetrahydrofuran (*trans*-19c) and (2*R**,3*R**)-3-chloro-2-(2,2-difluoroethoxy)tetrahydrofuran (*cis*-19c)**.

Trichloroacetimidate **17** was prepared using the representative procedure for trichloroacetimidate formation using hemiacetal **15** (0.299 g, 2.44 mmol), trichloroacetonitrile (2.50 mL, 24.9 mmol), and DBU (40 µL, 0.27 mmol) in CH_2Cl_2 (25 mL). Acetal **19c** was prepared using the representative procedure for additions of oxygen nucleophiles to α-halo acetals using trichloroacetimidate **17** (2.44 mmol), 2,2-difluoroethanol (625 µL, 9.87 mmol), and $BF_3{\cdot}OEt_2$ (620 µL, 4.94 mmol) in CH_2Cl_2 (25 mL). ^{1}H NMR and ^{13}C{^{1}H} NMR spectroscopic analysis of the unpurified reaction mixture revealed that acetal **19c** was formed as an 88:12 mixture of diastereomers (*trans*-**19c**:*cis*-**19c**). Purification by flash chromatography (5:95 EtOAc:hexanes) afforded the major diastereomer *trans*-**19c** as a colorless oil (0.092 g, 20%) and the minor diastereomer *cis*-**19c** as colorless oil (0.051 g, 11%). The relative stereochemical configurations of the two compounds were assigned by spectroscopic correlation to acetal **19e**; details are provided later in Section VII:

Major Diastereomer *trans*-**19c**:

^{1}H NMR (400 MHz, $CDCl_3$) 5.86 (tdd, J = 55.5, 4.9, 3.6, 1H), 5.15 (s, 1H), 4.28 (d, J = 5.8, 1.3, 1H), 4.23–4.17 (m, 1H), 4.08 (td, J = 8.6, 3.4, 1H), 3.87–3.76 (m, 1H), 3.74–3.63 (m, 1H), 2.60–2.50 (m, 1H), 2.17–2.11 (m, 1H);

^{13}C{^{1}H} NMR (100 MHz, $CDCl_3$) δ 114.1 (br t, $^1J_{C–F}$ = 245.8, CH), 108.5 (CH), 67.0 (CH_2), 66.2 (br t, $^2J_{C–F}$ = 27.9, CH_2), 60.0 (CH), 33.1 (CH_2);

^{19}F{^{1}H} NMR (377 MHz, $CDCl_3$) δ–126.4 (m);

IR (ATR) 2908, 1115, 1065, 1028, 988, 909 cm^{-1}.

Minor Diastereomer *cis*-**19c**:

^{1}H NMR (400 MHz, $CDCl_3$) 5.95 (tdd, J = 55.5, 4.8, 3.9, 1H), 5.02 (d, J = 4.0, 1H), 4.14–4.05 (m, 2H), 3.96–3.74 (m, 3H), 2.47–2.39 (m, 1H), 2.30–2.17 (m, 1H);

^{13}C{1H} NMR (100 MHz, $CDCl_3$) δ 114.3 (br t, $^1J_{C–F}$ = 241.7, CH), 101.7 (CH), 66.8 (br t, $^2J_{C–F}$ = 28.9, CH_2), 65.9 (CH_2), 55.6 (CH), 31.6 (CH_2).

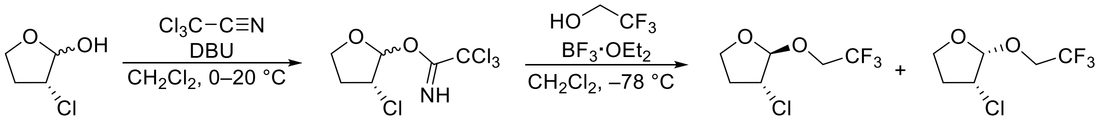

15 **17** *trans*-**19d** *cis*-**19d**

(2*R,3*S**)-3-Chloro-2-(2,2,2-trifluoroethoxy)tetrahydrofuran (*trans*-19d) and (2*R**,3*R**)-3-chloro-2-(2,2,2-trifluoroethoxy)tetrahydrofuran (*cis*-19d)**.

Trichloroacetimidate **17** was prepared using the representative procedure for trichloroacetimidate formation using hemiacetal **15** (0.403 g, 3.29 mmol), trichloroacetonitrile (3.3 mL, 33 mmol), and DBU (50 µL, 0.34 mmol) in CH_2Cl_2 (30 mL). Acetal **19d** was prepared using the representative procedure for additions of oxygen nucleophiles to α-halo acetals using trichloroacetimidate **17** (3.29 mmol), 2,2,2-trifluoroethanol (940 µL, 13.1 mmol), and $BF_3·OEt_2$ (820 µL, 6.53 mmol) in CH_2Cl_2 (20 mL). ^{1}H NMR and ^{13}C{^{1}H} NMR spectroscopic analysis of the unpurified reaction mixture revealed that acetal **19d** was formed as an 88:12 mixture of diastereomers (*trans*-**19d**:*cis*-**19d**). Purification by bulb-to-bulb Kugelrohr distillation (<1 atm at 60 °C) afforded acetal *trans*-**19d** as a colorless oil (0.158 g, 23%) and acetal *cis*-**19d** as a colorless oil (0.054 g, 8%). The relative stereochemical configurations of the two compounds were assigned by spectroscopic correlation to acetal **19e**; details are provided later in Section VII:

Major Diastereomer *trans*-**19d**:

^{1}H NMR (400 MHz, $CDCl_3$) δ 5.19 (s, 1H), 4.32 (dd, *J* = 5.8, 1.3, 1H), 4.25–4.19 (m, 1H), 4.09 (td, *J* = 8.7, 3.3, 1H), 3.99–3.83 (m, 2H), 2.61–2.52 (m, 1H), 2.19–2.12 (m, 1H);

^{13}C{^{1}H} NMR (100 MHz, $CDCl_3$) δ 123.7 (br q, $^1J_{C–F}$ = 279.3, CF_3), 107.9 (CH), 67.3 (CH_2), 63.6 (br q, $^2J_{C–F}$ = 35.0, CH_2), 59.9 (CH), 33.0 (CH_2);

^{19}F{^{1}H} NMR (377 MHz, $CDCl_3$) δ –75.3 (s);

IR (ATR) 2963, 1275, 1140, 1084, 943, 828 cm^{-1};

HRMS (ESI) *m / z* calcd for C_4H_6ClO ((M + H) – $C_2H_3F_3O$)$^+$ 105.0102, found 105.0100.

Minor Diastereomer *cis*-**19d**:

^{1}H NMR (400 MHz, $CDCl_3$) δ 5.05 (d, *J* = 3.9, 1H), 4.14–4.06 (m, 2H), 4.04–3.92 (m, 3H), 2.48–2.40 (m, 1H), 2.32–2.21 (m, 1H);

^{13}C{^{1}H} NMR (100 MHz, $CDCl_3$) δ 123.8 (br q, $^1J_{C–F}$ = 278.1, CF_3), 101.4 (CH), 66.2 (CH), 64.2 (br q, $^2J_{C–F}$ = 35.2, CH_2), 55.4 (CH_2), 31.3 (CH_2).

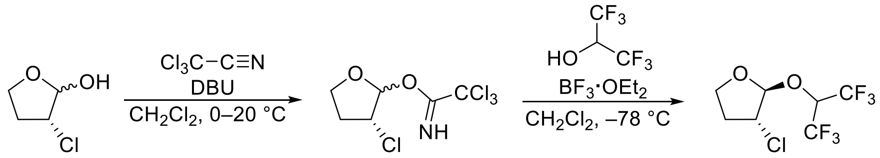

15 17 19e

(2*R,3*S**)-3-Chloro-2-((1,1,1,3,3,3-hexafluoropropan-2-yl)oxy)tetrahydrofuran (19e)**.

Trichloroacetimidate **17** was prepared using the representative procedure for trichloroacetimidate formation using hemiacetal **15** (2.46 mmol), trichloroacetonitrile (2.50 mL, 2.49 mmol), and DBU (40

µL, 0.27 mmol) in CH_2Cl_2 (25 mL). Acetal **19e** was prepared using the representative procedure for additions of oxygen nucleophiles to α-halo acetals using trichloroacetimidate **17** (2.46 mmol), hexafluoroisopropanol (1.05 mL, 10.0 mmol), and $BF_3{\cdot}OEt_2$ (620 µL, 4.94 mmol) in CH_2Cl_2 (25 mL). ^{1}H NMR and ^{13}C{^{1}H} NMR spectroscopic analysis of the unpurified reaction mixture revealed that acetal **19e** was formed as a single diastereomer (dr >99:1). Purification by flash chromatography (5:95 EtOAc:hexanes) afforded acetal **19e** as a white solid (0.088 g, 13%). The spectroscopic data (^{1}H NMR, ^{13}C{^{1}H} NMR, ^{19}F{^{1}H} NMR, IR, HRMS) are consistent with the data reported in literature.[8]

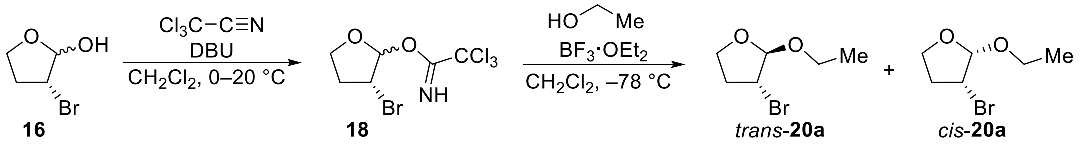

(2*R,3*R**)-3-Bromo-2-ethoxytetrahydrofuran (*trans*-20a) and (2*R**,3*S**)-3-bromo-2-ethoxytetrahydrofuran (*cis*-20a)**.

Trichloroacetimidate **18** was prepared using the representative procedure for trichloroacetimidate formation using hemiacetal **16** (0.281 g, 1.69 mmol), trichloroacetonitrile (1.2 mL, 12 mmol), and DBU (20 µL, 0.13 mmol) in CH_2Cl_2 (12 mL). Acetal **20a** was prepared using the representative procedure for additions of oxygen nucleophiles to α-halo acetals using trichloroacetimidate **18** (1.69 mmol), ethanol (290 µL, 4.95 mmol), and $BF_3{\cdot}OEt_2$ (300 µL, 2.39 mmol) in CH_2Cl_2 (12 mL). ^{1}H NMR and ^{13}C{^{1}H} NMR spectroscopic analysis of the unpurified reaction mixture revealed that acetal **20a** was formed as an 89:11 mixture of diastereomers (*trans*-**20a**:*cis*-**20a**). Purification by flash chromatography (5:95 EtOAc:hexanes) afforded the major diastereomer *trans*-**20a** as a colorless oil (0.132 g, 40%) and the minor diastereomer *cis*-**20a** as a colorless oil (0.003 g, 1%). The relative stereochemical configurations of the two compounds were assigned by spectroscopic correlation to acetal **20e**; details are provided later in Section VII:

Major Diastereomer *trans*-**20a**:

^{1}H NMR (400 MHz, $CDCl_3$) δ 5.23 (br s, 1H), 4.21 (dd, J = 5.9, 1.5, 1H), 4.15 (q, J = 8.2, 1H), 4.06 (td, J = 8.5, 4.4, 1H), 3.75–3.68 (m, 1H), 3.52–3.44 (m, 1H), 2.64 (dddd, J = 14.4, 8.4, 6.1, 1H), 2.21 (dddd, J = 14.0, 7.0, 3.4, 1.7, 1H), 1.18 (t, J = 7.1, 3H);

^{13}C{1H} NMR (100 MHz, $CDCl_3$) δ 108.5 (CH), 66.6 (CH_2), 63.0 (CH_2), 50.2 (CH), 34.0 (CH_2), 15.1 (CH_3);

IR (ATR) 2976, 1198, 1072, 1031, 1011, 911 cm^{-1};

HRMS (APCI) *m / z* calcd for C_4H_6BrO ((M + H) – C_2H_6O)$^+$ 148.9597, found 148.9591.

Minor Diastereomer *cis*-**20a**:

^{1}H NMR (400 MHz, $CDCl_3$) δ 4.97 (d, *J* = 4.0, 1H), 4.07–4.01 (m, 2H), 3.88 (q, *J* = 7.9, 1H), 3.78 (ddd, *J* = 9.8, 7.1, 1H), 3.59 (ddd, *J* = 9.8, 7.1, 1H), 2.50–2.41 (m, 1H), 2.38–2.28 (m, 1H), 1.25 (t, *J* = 7.1, 1H);

$^{13}C\{^1H\}$ NMR (100 MHz, $CDCl_3$) δ 101.2 (CH), 65.6 (CH_2), 63.6 (CH_2), 45.4 (CH), 32.7 (CH_2), 15.1 (CH_3).

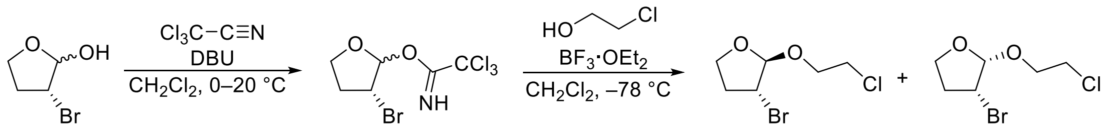

(2*R,3*S**)-3-Bromo-2-(2-chloroethoxy)tetrahydrofuran (*trans*-20b) and (2*R**,3*R**)-3-bromo-2-(2-chloroethoxy)tetrahydrofuran (*cis*-20b)**.

Trichloroacetimidate **18** was prepared using the representative procedure for trichloroacetimidate formation using hemiacetal **16** (0.405 g, 2.44 mmol), trichloroacetonitrile (2.5 mL, 25 mmol), and DBU (40 µL, 0.27 mmol) in CH_2Cl_2 (25 mL). Acetal **20b** was prepared using the representative procedure for additions of oxygen nucleophiles to α-halo acetals using trichloroacetimidate **18** (2.44 mmol), 2-chloroethanol (650 µL, 9.70 mmol), and $BF_3{\cdot}OEt_2$ (600 µL, 4.78 mmol) in CH_2Cl_2 (25 mL). ^1H NMR and $^{13}C\{^1H\}$ NMR spectroscopic analysis of the unpurified reaction mixture revealed that acetal **20b** was formed as a 96:4 mixture of diastereomers (*trans*-**20b**:*cis*-**20b**). Purification by flash chromatography (5:95 EtOAc:hexanes) afforded the major diastereomer *trans*-**20b** as a light yellow oil (0.409 g, 73%). The relative stereochemical configurations of the two compounds were assigned by spectroscopic correlation to acetal **20e**; details are provided later in Section VII:

Major Diastereomer *trans*-**20b**:

^1H NMR (400 MHz, $CDCl_3$) δ 5.27 (s, 1H), 4.26 (dd, *J* = 5.9, 1.4, 1H), 4.21–4.15 (m, 1H), 4.08 (td, *J* = 8.5, 3.3, 1H), 3.93–3.88 (m, 1H), 3.74–3.68 (m, 1H), 3.63–3.60 (m, 2H), 2.71–2.62 (m, 1H), 2.26–2.19 (m, 1H);

$^{13}C\{^1H\}$ NMR (100 MHz, $CDCl_3$) δ 108.7 (CH), 67.3 (CH_2), 67.0 (CH_2), 49.7 (CH), 42.9 (CH_2), 33.8 (CH_2);

IR (ATR) 2959, 1437, 1299, 1102, 1026, 1003 cm^{-1};

HRMS (ESI) *m / z* calcd for $C_6H_{10}BrO_2$ ((M + H) – HCl)$^+$ 192.9859, found 192.9866.

Minor Diastereomer *cis*-**20b**:

^1H NMR (400 MHz, $CDCl_3$, diagnostic peaks) δ 5.02 (d, *J* = 3.9, 1H);

$^{13}C\{^1H\}$ NMR (100 MHz, $CDCl_3$, diagnostic peaks) δ 113.2 (CH).

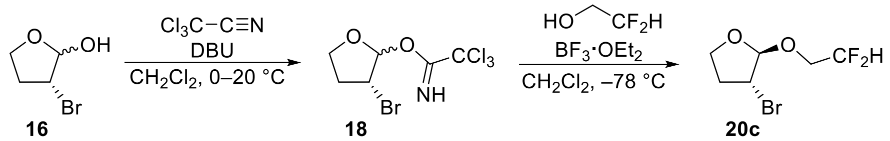

(2*R,3*S**)-3-Bromo-2-(2,2-difluoroethoxy)tetrahydrofuran (20c)**.

Trichloroacetimidate **18** was prepared using the representative procedure for trichloroacetimidate formation using hemiacetal **16** (0.222 g, 1.34 mmol), trichloroacetonitrile (1.2 mL, 12 mmol), and DBU (20 µL, 0.13 mmol) in CH_2Cl_2 (12 mL). Acetal **20c** was prepared using the representative procedure for additions of oxygen nucleophiles to α-halo acetals using trichloroacetimidate **18** (1.34 mmol), 2,2-difluoroethanol (305 µL, 4.82 mmol), and $BF_3{\cdot}OEt_2$ (300 µL, 2.39 mmol) in CH_2Cl_2 (12 mL). ^{1}H NMR, ^{13}C{^{1}H} NMR, and ^{19}F{^{1}H} NMR spectroscopic analysis of the unpurified reaction mixture revealed that acetal **20c** was formed as a single diastereomer (dr >99:1). Purification by flash chromatography (5:95 EtOAc:hexanes) afforded acetal **20c** as a colorless oil (0.110 g, 35%). The relative stereochemical configurations of acetal **20c** was assigned by spectroscopic correlation to acetal **20e**; details are provided later in Section VII:

^{1}H NMR (400 MHz, $CDCl_3$) δ 5.86 (tdd, *J* = 55.4, 4.8, 3.5, 1H), 5.27 (s, 1H), 4.26 (dd, *J* = 4.8, 3.5, 1H), 4.20 (dt, *J* = 8.4, 7.1, 1H), 4.08 (td, *J* = 8.5, 3.3, 1H), 3.87–3.76 (m, 1H), 3.70–3.63 (m, 1H), 2.70–2.61 (m, 1H), 2.27–2.20 (m, 1H);

^{13}C{^{1}H} NMR (100 MHz, $CDCl_3$) δ 114.1 (br t, $^{1}J_{C\text{–}F}$ = 241.1, CH), 108.9 (CH), 67.2 (CH_2), 66.2 (br t, $^{2}J_{C\text{–}F}$ = 28.2, CH_2), 49.3 (CH), 33.6 (CH_2);

^{19}F{^{1}H} NMR (377 MHz, $CDCl_3$) δ –126.4 (d, *J* = 4.0);

IR (ATR) 2904, 1316, 1104, 1073, 1034, 921 cm^{-1};

HRMS (ESI) *m / z* calcd for C_4H_6BrO ((M + H) – $C_2H_4F_2O$)$^+$ 148.9597, found 148.9602.

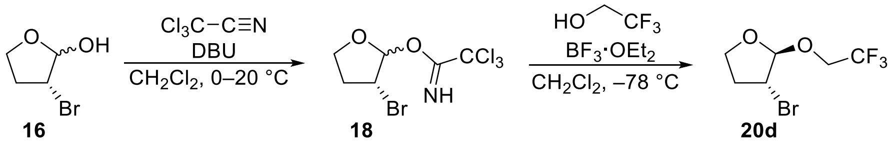

(2*R,3*S**)-3-Bromo-2-(2,2,2-trifluoroethoxy)tetrahydrofuran (20d)**.

Trichloroacetimidate **18** was prepared using the representative procedure for trichloroacetimidate formation using hemiacetal **16** (0.244 g, 1.47 mmol), trichloroacetonitrile (1.2 mL, 12 mmol), and DBU (20 µL, 0.13 mmol) in CH_2Cl_2 (12 mL). Acetal **20d** was prepared using the representative procedure for additions of oxygen nucleophiles to α-halo acetals using trichloroacetimidate **18** (1.47 mmol), 2,2,2-

trifluoroethanol (350 µL, 4.86 mmol), and $BF_3{\cdot}OEt_2$ (300 µL, 2.39 mmol) in CH_2Cl_2 (12 mL). ^1H NMR, $^{13}C\{^1H\}$ NMR, and $^{19}F\{^1H\}$ NMR spectroscopic analysis of the unpurified reaction mixture revealed that acetal **20d** was formed as a single diastereomer (dr >99:1). Purification by flash chromatography (5:95 EtOAc:hexanes) afforded acetal **20d** as a colorless oil (0.176 g, 48%). The relative stereochemical configurations of acetal **20d** was assigned by correlation to spectroscopic acetal **20e**; details are provided later in Section VII:

^1H NMR (400 MHz, $CDCl_3$) δ 5.31 (s, 1H), 4.29 (dd, J = 5.8, 1.2, 1H), 4.22 (dt, J = 8.7, 7.2, 1H), 4.09 (td, J = 8.6, 3.1, 1H), 4.01–3.91 (m, 1H), 3.90–3.80 (m, 1H), 2.71–2.62 (m, 1H), 2.28–2.24 (m, 1H);

$^{13}C\{^1H\}$ NMR (100 MHz, $CDCl_3$) δ 123.8 (br q, $^1J_{C-F}$ = 278.1, CF_3), 108.3 (CH), 67.5 (CH_2), 63.6 (br q, $^2J_{C-F}$ = 34.8, CH_2), 49.1 (CH), 33.4 (CH_2);

$^{19}F\{^1H\}$ NMR (377 MHz, $CDCl_3$) δ –75.3 (s);

IR (ATR) 2954, 1277, 1156, 1081, 1040, 959 cm^{-1};

HRMS (APCI) m / z calcd for C_4H_6BrO ((M + H) – $C_2H_3F_3O)^+$ 148.9597, found 148.9597.

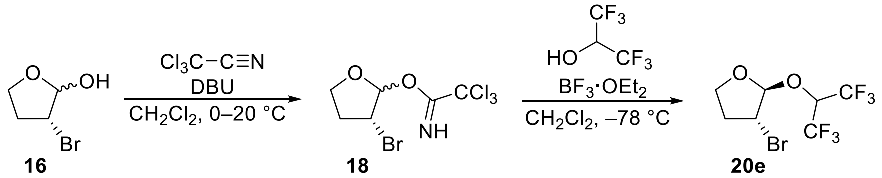

16 18 20e

(2*R,3*S**)-3-Bromo-2-((1,1,1,3,3,3-hexafluoropropan-2-yl)oxy)tetrahydrofuran (20e)**.

Trichloroacetimidate **18** was prepared using the representative procedure for trichloroacetimidate formation using hemiacetal **16** (0.404 g, 2.43 mmol), trichloroacetonitrile (2.5 mL, 25 mmol), and DBU (40 µL, 0.26 mmol) in CH_2Cl_2 (25 mL). Acetal **20e** was prepared using the representative procedure for additions of oxygen nucleophiles to α-halo acetals using trichloroacetimidate **18** (2.43 mmol), hexafluoro-2-propanol (1.000 µL, 9.523 mmol), and $BF_3{\cdot}OEt_2$ (600 µL, 4.78 mmol) in CH_2Cl_2 (25 mL). ^1H NMR, $^{13}C\{^1H\}$ NMR, and $^{19}F\{^1H\}$ NMR spectroscopic analysis of the unpurified reaction mixture revealed that acetal **20e** was formed as a single diastereomer (dr >99:1). Purification by flash chromatography (5:95 EtOAc:hexanes) afforded acetal **20e** as a white solid (0.135 g, 18%). The spectroscopic data (^1H NMR, $^{13}C\{^1H\}$ NMR, $^{19}F\{^1H\}$ NMR, IR, HRMS) are consistent with the data reported in literature.[8]

IV. Parameter Screen for *O*-Glycosylation Conditions

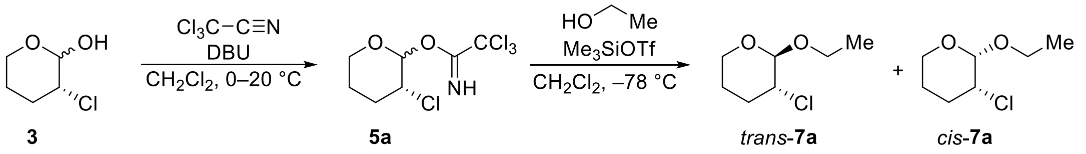

(2*R,3*R**)-3-Chloro-2-ethoxytetrahydro-2*H*-pyran (*trans*-7a) and (2*R**,3*S**)-3-chloro-2-ethoxytetrahydro-2*H*-pyran (*cis*-7a).**

Trichloroacetimidate **5a** was prepared using the representative procedure for trichloroacetimidate formation using hemiacetal **3** (0.322 g, 2.39 mmol), trichloroacetonitrile (2.2 mL, 22 mmol), and DBU (40 µL, 0.27 mmol) in CH_2Cl_2 (22 mL). Acetal **7a** was prepared using the representative procedure for additions of oxygen nucleophiles to α-halo acetals using trichloroacetimidate **5a** (2.39 mmol), ethanol (520 µL, 8.91 mmol), and Me_3SiOTf (800 µL, 4.43 mmol) in CH_2Cl_2 (25 mL). ^1H NMR and $^{13}C\{^1H\}$ NMR spectroscopic analysis of the unpurified reaction mixture revealed that acetal **7a** was formed as an 86:14 mixture of diastereomers (*trans*-**7a**:*cis*-**7a**). The spectroscopic data are consistent with those of the same acetal prepared from hemiacetal **5a** and $BF_3{\cdot}OEt_2$ in CH_2Cl_2.

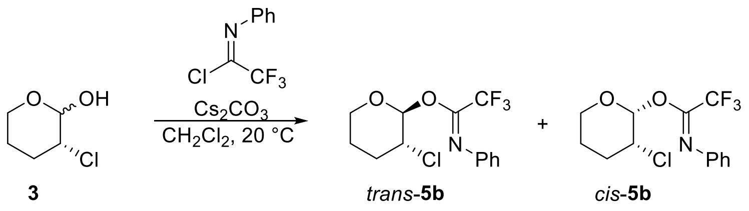

(2*R,3*S**)-3-Chlorotetrahydro-2*H*-pyran-2-yl (*E*)-2,2,2-trifluoro-*N*-phenylacetimidate (*trans*-7a) and (2*R**,3*R**)-3-chlorotetrahydro-2*H*-pyran-2-yl (*E*)-2,2,2-trifluoro-*N*-phenylacetimidate (*cis*-7a).**

A reported procedure[9] was adapted to prepare trifluoroacetimidate **5b**. To a solution of hemiacetal **3** (0.314 g, 2.30 mmol) in CH_2Cl_2 (20 mL) was added Cs_2CO_3 (2.194 g, 6.734) mmol and 2,2,2-trifluoro-*N*-phenylacetimidoyl chloride (0.482 g, 2.32 mmol). After 13 h, the reaction mixture was filtered through silica (½ inch) with CH_2Cl_2 (20 mL) and then concentrated *in vacuo* to afford trifluoroacetimidate **5b** as a colorless oil (0.580 g, 81%). Trifluoroacetimidate **5b** was used directly without further purification.

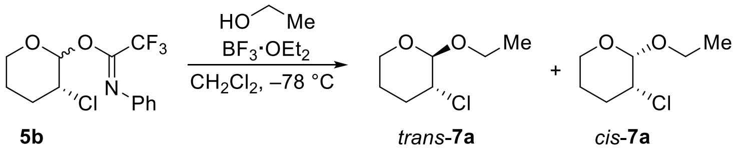

(2*R,3*R**)-3-Chloro-2-ethoxytetrahydro-2*H*-pyran (*trans*-7a) and (2*R**,3*S**)-3-chloro-2-ethoxytetrahydro-2*H*-pyran (*cis*-7a).**

Acetal **7a** was prepared using the representative procedure for additions of oxygen nucleophiles to α-halo acetals using trifluoroacetimidate **5b** (0.060 g, 0.20 mmol), ethanol (40 µL, 0.69 mmol), and

BF_3·OEt_2 (40 µL, 0.32 mmol) in CH_2Cl_2 (2 mL). ^1H NMR and $^{13}C\{^1H\}$ NMR spectroscopic analysis of the unpurified reaction mixture revealed that acetal **7a** was formed as an 85:15 mixture of diastereomers (*trans*-**7a**:*cis*-**7a**). The spectroscopic data are consistent with those of the same acetal prepared from hemiacetal **5a** and BF_3·OEt_2 in CH_2Cl_2.

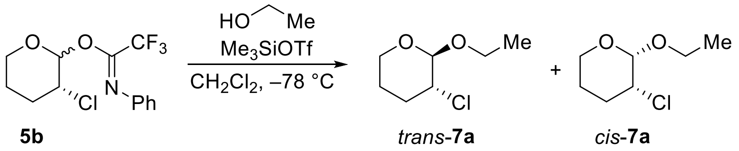

(2*R,3*R**)-3-Chloro-2-ethoxytetrahydro-2*H*-pyran (*trans*-7a) and (2*R**,3*S**)-3-chloro-2-ethoxytetrahydro-2*H*-pyran (*cis*-7a).**

Acetal **7a** was prepared using the representative procedure for additions of oxygen nucleophiles to α-halo acetals using trifluoroacetimidate **5b** (0.075 g, 0.24 mmol), ethanol (54 µL, 0.92 mmol), and Me_3SiOTf (85 µL, 0.47 mmol) in CH_2Cl_2 (3 mL). ^1H NMR and $^{13}C\{^1H\}$ NMR spectroscopic analysis of the unpurified reaction mixture revealed that acetal **7a** was formed as an 86:14 mixture of diastereomers (*trans*-**7a**:*cis*-**7a**). The spectroscopic data are consistent with those of the same acetal prepared from hemiacetal **5a** and BF_3·OEt_2 in CH_2Cl_2.

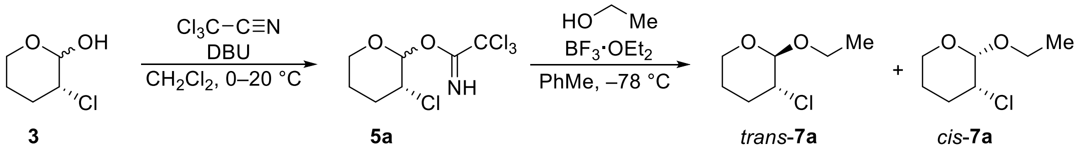

(2*R,3*R**)-3-Chloro-2-ethoxytetrahydro-2*H*-pyran (*trans*-7a) and (2*R**,3*S**)-3-chloro-2-ethoxytetrahydro-2*H*-pyran (*cis*-7a).**

Trichloroacetimidate **5a** was prepared using the representative procedure for trichloroacetimidate formation using hemiacetal **3** (0.070 g, 0.51 mmol), trichloroacetonitrile (515 µL, 5.14 mmol), and DBU (8 µL, 0.5 mmol) in CH_2Cl_2 (5 mL). Acetal **7a** was prepared using the representative procedure for additions of oxygen nucleophiles to α-halo acetals using trichloroacetimidate **5a** (0.51 mmol), ethanol (120 µL, 2.05 mmol), and BF_3·OEt_2 (130 µL, 1.04 mmol) in CH_2Cl_2 (5 mL). ^1H NMR and $^{13}C\{^1H\}$ NMR spectroscopic analysis of the unpurified reaction mixture revealed that acetal **7a** was formed as a 91:9 mixture of diastereomers (*trans*-**7a**:*cis*-**7a**). The spectroscopic data are consistent with those of the same acetal prepared from hemiacetal **5a** and BF_3·OEt_2 in CH_2Cl_2.

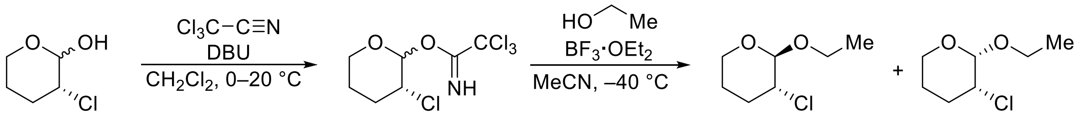

(2*R,3*R**)-3-Chloro-2-ethoxytetrahydro-2*H*-pyran (*trans*-7a) and (2*R**,3*S**)-3-chloro-2-ethoxytetrahydro-2*H*-pyran (*cis*-7a).**

Trichloroacetimidate **5a** was prepared using the representative procedure for trichloroacetimidate formation using hemiacetal **3** (0.072 g, 0.53 mmol), trichloroacetonitrile (515 µL, 5.14 mmol), and DBU (8 µL, 0.5 mmol) in CH_2Cl_2 (5 mL). Acetal **7a** was prepared using the representative procedure for additions of oxygen nucleophiles to α-halo acetals using trichloroacetimidate **5a** (0.53 mmol), ethanol (120 µL, 2.05 mmol), and $BF_3{\cdot}OEt_2$ (130 µL, 1.04 mmol) in CH_2Cl_2 (5 mL). ^{1}H NMR and ^{13}C{^{1}H} NMR spectroscopic analysis of the unpurified reaction mixture revealed that acetal **7a** was formed as a 66:34 mixture of diastereomers (*trans*-**7a**:*cis*-**7a**). The spectroscopic data are consistent with those of the same acetal prepared from hemiacetal **5a** and $BF_3{\cdot}OEt_2$ in CH_2Cl_2.

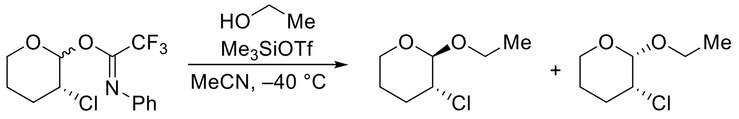

(2*R,3*R**)-3-Chloro-2-ethoxytetrahydro-2*H*-pyran (*trans*-7a) and (2*R**,3*S**)-3-chloro-2-ethoxytetrahydro-2*H*-pyran (*cis*-7a).**

Acetal **7a** was prepared using the representative procedure for additions of oxygen nucleophiles to α-halo acetals using trifluoroacetimidate **5b** (0.067 g, 0.22 mmol), ethanol (40 µL, 0.69 mmol), and Me_3SiOTf (60 µL, 0.33 mmol) in CH_2Cl_2 (2 mL). ^{1}H NMR and ^{13}C{^{1}H} NMR spectroscopic analysis of the unpurified reaction mixture revealed that acetal **7a** was formed as a 66:34 mixture of diastereomers (*trans*-**7a**:*cis*-**7a**). The spectroscopic data are consistent with those of the same acetal prepared from hemiacetal **5a** and $BF_3{\cdot}OEt_2$ in CH_2Cl_2.

V. Epimerization Studies

Epimerization studies were performed on all acetals to ensure that the diastereomeric ratios listed in Tables **2–5** are representative of kinetically controlled additions at –78 °C. All acetals were resubjected to the standard conditions for additions of *O*-nucleophiles, and the reactions were observed for epimerization (denoted by changes in the diastereomeric ratio of the starting acetal) and substitution

(denoted by the formation of a new acetal product resulting from activation of the previous acetal and subsequent addition of the alcohol). The epimerization and substitution reactions should proceed through the same mechanism, namely formation of an oxocarbenium ion intermediate followed by trapping with an alcohol. Either reaction would indicate that activation of the product acetal had occurred. Considering that the diastereomeric ratio of the acetal under kinetic control might be the same as the ratio under thermodynamic control, the substitutions of other alcohols are a better test for whether the reactions are under kinetic control, or whether the reaction conditions are causing the product ratio to be determined by thermodynamic control. The reactions were performed at varied temperatures (–78, 0, and 20 °C), although all the original substitution reactions were performed at –78 °C. For all the acetals, epimerization and substitution did not occur at this temperature, confirming that the diastereomer ratios shown in Tables 2–5 represent kinetically controlled stereoselectivity. Epimerization and substitution were only observed at the higher temperatures in some cases.

Because the rate of protonation of the acetal decreases as the electron-withdrawing abilities of the leaving group increases, acetals with the more basic ethoxy groups were more readily ionizable (denoted by epimerization and substitution) than acetals with halogenated leaving groups. This phenomenon had been observed with other acetals.[10] The products of epimerization or substitution at higher temperatures reflect the diastereomeric ratio under thermodynamic control. This ratio favors the formation of the *trans*-product because hyperconjugative interactions between the carbon–halogen bond and the exocyclic carbon–oxygen bond ($\sigma_{C-X} \rightarrow \sigma^*_{C-OR}$) are promoted in the 1,2-diaxial conformation.[8]

A. α-Chlorinated Pyrans

a. Acetal 7a

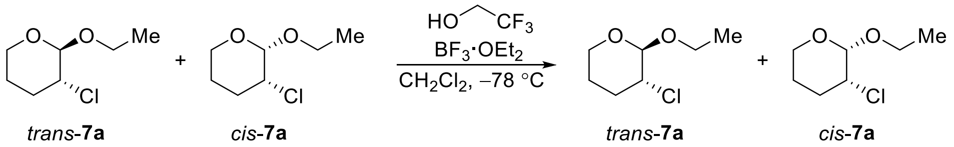

Epimerization studies on acetal **7a** were performed following the representative procedure using acetal **7a** (0.053 g, 0.32 mmol, dr 77:23), 2,2,2-trifluoroethanol (90 µL, 1.3 mmol), and $BF_3 \cdot OEt_2$ (76 µL, 0.61 mmol) in CH_2Cl_2 (3 mL) at –78 °C for 1 h. ^1H NMR and $^{13}C\{^1H\}$ NMR spectroscopic analysis of the unpurified reaction mixture revealed a 100:0 mixture of products (**7a**:**7d**). Acetal **7a** was recovered as a 78:22 mixture of diastereomers.

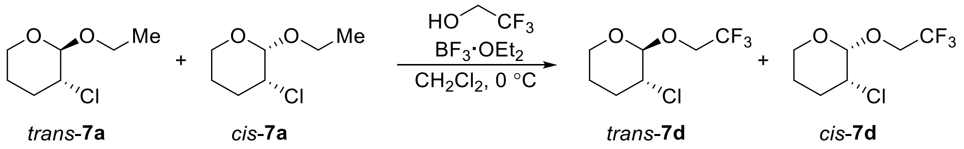

Epimerization studies on acetal **7a** were performed following the representative procedure using acetal **7a** (0.055 g, 0.33 mmol, dr 77:23), 2,2,2-trifluoroethanol (90 µL, 1.3 mmol), and $BF_3{\cdot}OEt_2$ (80 µL, 0.64 mmol) in CH_2Cl_2 (3 mL) at 0 °C for 1 h. ^{1}H NMR and ^{13}C{^{1}H} NMR spectroscopic analysis of the unpurified reaction mixture revealed a 0:100 mixture of products (**7a**:**7d**). Acetal **7d** was formed as a 69:31 mixture of diastereomers.

Note: The observance of the substitution product indicates that acetal formation was reversible with acetal **7a** with $BF_3{\cdot}OEt_2$ at 0 °C.

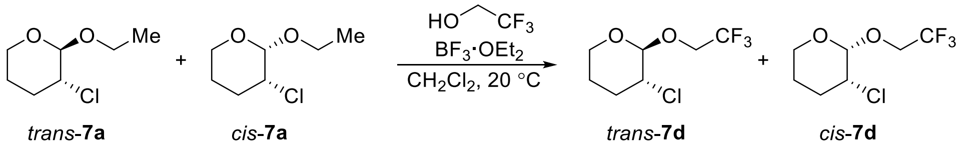

Epimerization studies on acetal **7a** were performed following the representative procedure using acetal **7a** (0.051 g, 0.32 mmol, dr 77:23), 2,2,2-trifluoroethanol (90 µL, 1.3 mmol), and $BF_3{\cdot}OEt_2$ (80 µL, 0.64 mmol) in CH_2Cl_2 (3 mL) at 20 °C for 1 h. ^{1}H NMR and ^{13}C{^{1}H} NMR spectroscopic analysis of the unpurified reaction mixture revealed a 0:100 mixture of products (**7a**:**7d**). Acetal **7d** was formed as a 65:35 mixture of diastereomers.

Note: The observance of the substitution product indicates that acetal formation was reversible with acetal **7a** with $BF_3{\cdot}OEt_2$ at 20 °C.

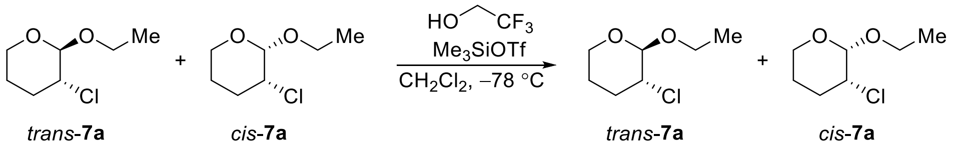

Epimerization studies on acetal **7a** were performed following the representative procedure using acetal **7a** (0.050 g, 0.30 mmol, dr 78:22), 2,2,2-trifluoroethanol (90 µL, 1.3 mmol), and Me_3SiOTf (110 µL, 0.609 mmol) in CH_2Cl_2 (3 mL) at –78 °C for 1 h. ^{1}H NMR and ^{13}C{^{1}H} NMR spectroscopic analysis of the unpurified reaction mixture revealed a 100:0 mixture of products (**7a**:**7d**). Acetal **7a** was recovered as a 78:22 mixture of diastereomers.

Conclusion for Acetal 7a: Under the conditions listed in Tables 2 ($BF_3{\cdot}OEt_2$ at –78 °C) and 3 (Me_3SiOTf at –78 °C), epimerization did not occur. Acetal formation was reversible at higher temperatures (≥ 0 °C), however.

b. Acetal 7b

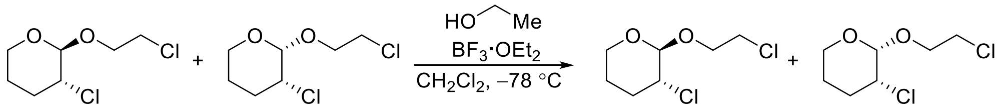

trans-**7b** *cis*-**7b** *trans*-**7b** *cis*-**7b**

Epimerization studies on acetal **7b** were performed following the representative procedure using acetal **7b** (0.060 g, 0.30 mmol, dr 71:29), ethanol (70 µL, 1.2 mmol), and $BF_3{\cdot}OEt_2$ (75 µL, 0.60 mmol) in CH_2Cl_2 (3 mL) at –78 °C for 1 h. ^1H NMR and $^{13}C\{^1H\}$ NMR spectroscopic analysis of the unpurified reaction mixture revealed a 100:0 mixture of products (**7b**:**7a**). Acetal **7b** was recovered as a 69:31 mixture of diastereomers.

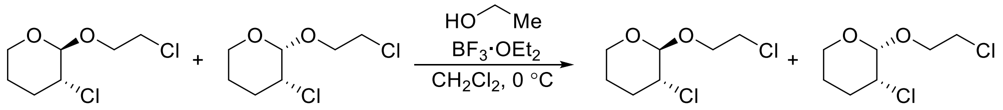

trans-**7b** *cis*-**7b** *trans*-**7b** *cis*-**7b**

Epimerization studies on acetal **7b** were performed following the representative procedure using acetal **7b** (0.063 g, 0.32 mmol, dr 71:29), ethanol (70 µL, 1.2 mmol), and $BF_3{\cdot}OEt_2$ (75 µL, 0.60 mmol) in CH_2Cl_2 (3 mL) at 0 °C for 1 h. ^1H NMR and $^{13}C\{^1H\}$ NMR spectroscopic analysis of the unpurified reaction mixture revealed a 100:0 mixture of products (**7b**:**7a**). Acetal **7b** was recovered as a 65:35 mixture of diastereomers.

Note: Epimerization might have occurred with acetal **7b** with $BF_3{\cdot}OEt_2$ at 0 °C.

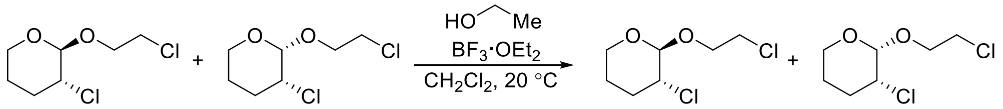

trans-**7b** *cis*-**7b** *trans*-**7b** *cis*-**7b**

Epimerization studies on acetal **7b** were performed following the representative procedure using acetal **7b** (0.059 g, 0.30 mmol, dr 71:29), ethanol (70 µL, 1.2 mmol), and BF3·OEt_2 (75 µL, 0.60 mmol) in CH_2Cl_2 (3 mL) at –78 °C for 1 h. ^1H NMR and $^{13}C\{^1H\}$ NMR spectroscopic analysis of the unpurified reaction mixture revealed a 100:0 mixture of products (**7b**:**7a**). Acetal **7b** was recovered as a 66:34 mixture of diastereomers.

Note: Epimerization might have occurred with acetal **7b** with $BF_3{\cdot}OEt_2$ at 20 °C.

Conclusion for Acetal 7b: Under the conditions listed in Table 2 ($BF_3{\cdot}OEt_2$ at –78 °C), epimerization did not occur. Epimerization might have occurred at higher temperatures (≥ 0 °C), however.

c. Acetal 7c

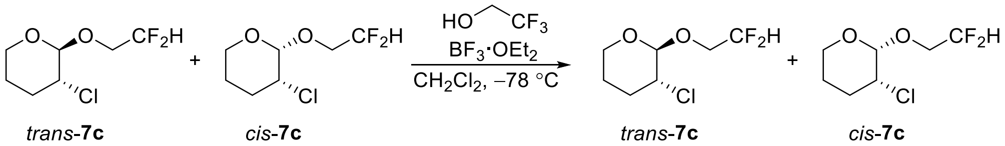

Epimerization studies on acetal **7c** were performed following the representative procedure using acetal **7c** (0.021 g, 0.10 mmol, dr 86:14), 2,2,2-trifluoroethanol (30 µL, 0.42 mmol), and $BF_3{\cdot}OEt_2$ (25 µL, 0.20 mmol) in CH_2Cl_2 (1 mL) at –78 °C for 1 h. ^{1}H NMR and ^{13}C{^{1}H} NMR spectroscopic analysis of the unpurified reaction mixture revealed a 100:0 mixture of products (**7c**:**7d**). Acetal **7c** was recovered as an 81:19 mixture of diastereomers.

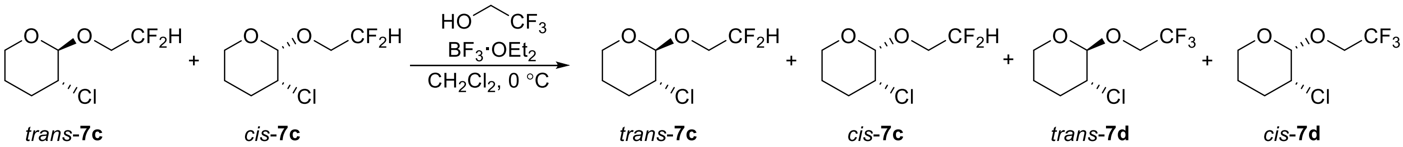

Epimerization studies on acetal **7c** were performed following the representative procedure using acetal **7c** (0.025 g, 0.12 mmol, dr 86:14), 2,2,2-trifluoroethanol (30 µL, 0.42 mmol), and $BF_3{\cdot}OEt_2$ (25 µL, 0.20 mmol) in CH_2Cl_2 (1 mL) at 0 °C for 1 h. ^{1}H NMR and ^{13}C{^{1}H} NMR spectroscopic analysis of the unpurified reaction mixture revealed a 19:81 mixture of products (**7c**:**7d**). Acetal **7c** was recovered as a 63:37 mixture of diastereomers. Acetal **7d** was formed as a 69:31 mixture of diastereomers.

Note: Epimerization occurred with acetal **7c** with $BF_3{\cdot}OEt_2$ at 0 °C. The observance of the substitution product indicates that acetal formation was reversible with acetal **7c** with $BF_3{\cdot}OEt_2$ at 0 °C.

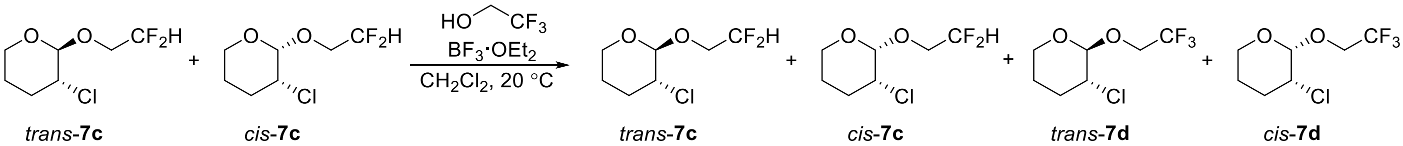

Epimerization studies on acetal **7c** were performed following the representative procedure using acetal **7c** (0.022 g, 0.11 mmol, dr 86:14), 2,2,2-trifluoroethanol (30 µL, 0.42 mmol), and $BF_3{\cdot}OEt_2$ (25 µL, 0.20 mmol) in CH_2Cl_2 (1 mL) at 20 °C for 1 h. ^{1}H NMR and ^{13}C{^{1}H} NMR spectroscopic analysis of the

unpurified reaction mixture revealed a 28:72 mixture of products (**7c**:**7d**). Acetal **7c** was recovered as a 67:33 mixture of diastereomers. Acetal **7d** was formed as a 66:34 mixture of diastereomers.

Note: Epimerization occurred with acetal **7c** with $BF_3{\cdot}OEt_2$ at 20 °C. The observance of the substitution product indicates that acetal formation was reversible with acetal **7c** with $BF_3{\cdot}OEt_2$ at 20 °C.

Conclusion for Acetal 7c: Under the conditions listed in Table 2 ($BF_3{\cdot}OEt_2$ at –78 °C), epimerization did not occur. Epimerization occurred at higher temperatures (≥ 0 °C), however. Acetal formation was also reversible at higher temperatures (≥ 0 °C).

d. Acetal 7d

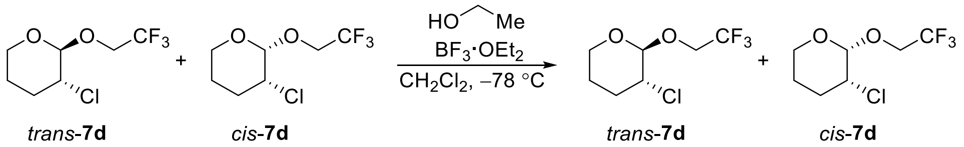

Epimerization studies on acetal **7d** were performed following the representative procedure using acetal **7d** (0.059 g, 0.27 mmol, dr 67:33), ethanol (55 µL, 0.94 mmol), and $BF_3{\cdot}OEt_2$ (60 µL, 0.48 mmol) in CH_2Cl_2 (3 mL) at –78 °C for 1 h. ^{1}H NMR and ^{13}C{^{1}H} NMR spectroscopic analysis of the unpurified reaction mixture revealed a 100:0 mixture of products (**7d**:**7a**). Acetal **7d** was recovered as a 66:34 mixture of diastereomers.

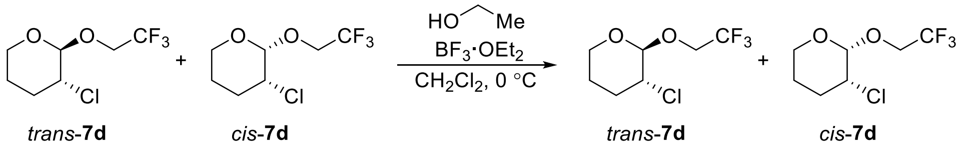

Epimerization studies on acetal **7d** were performed following the representative procedure using acetal **7d** (0.053 g, 0.24 mmol, dr 67:33), ethanol (55 µL, 0.94 mmol), and $BF_3{\cdot}OEt_2$ (60 µL, 0.48 mmol) in CH_2Cl_2 (3 mL) at 0 °C for 1 h. ^{1}H NMR and ^{13}C{^{1}H} NMR spectroscopic analysis of the unpurified reaction mixture revealed a 100:0 mixture of products (**7d**:**7a**). Acetal **7d** was recovered as a 69:31 mixture of diastereomers.

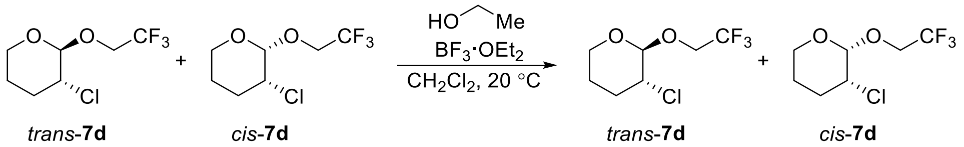

Epimerization studies on acetal **7d** were performed following the representative procedure using acetal **7d** (0.053 g, 0.24 mmol, dr 67:33), ethanol (55 µL, 0.94 mmol), and $BF_3{\cdot}OEt_2$ (60 µL, 0.48 mmol) in

CH_2Cl_2 (3 mL) at 20 °C for 1 h. ^{1}H NMR and ^{13}C{^{1}H} NMR spectroscopic analysis of the unpurified reaction mixture revealed a 100:0 mixture of products (**7d**:**7a**). Acetal **7d** was recovered as a 66:34 mixture of diastereomers.

Conclusion for Acetal 7d: Under the conditions listed in Table 2 ($BF_3 \cdot OEt_2$ at –78 °C), epimerization did not occur. Epimerization also did not occur at higher temperatures (≥ 0 °C).

e. Acetal 7e

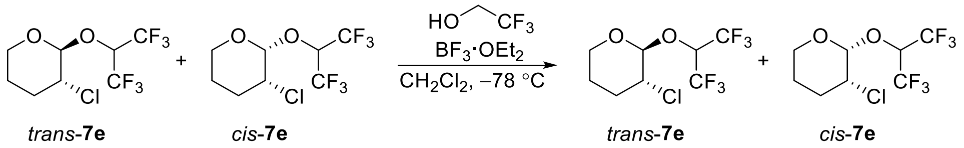

Epimerization studies on acetal **7e** were performed following the representative procedure using acetal **7e** (0.019 g, 0.066 mmol, dr >99:1), 2,2,2-trifluoroethanol (25 µL, 0.35 mmol), and $BF_3 \cdot OEt_2$ (21 µL, 0.17 mmol) in CH_2Cl_2 (1 mL) at –78 °C for 1 h. ^{1}H NMR and ^{13}C{^{1}H} NMR spectroscopic analysis of the unpurified reaction mixture revealed a 100:0 mixture of products (**7e**:**7d**). Acetal **7e** was recovered as a >99:1 mixture of diastereomers.

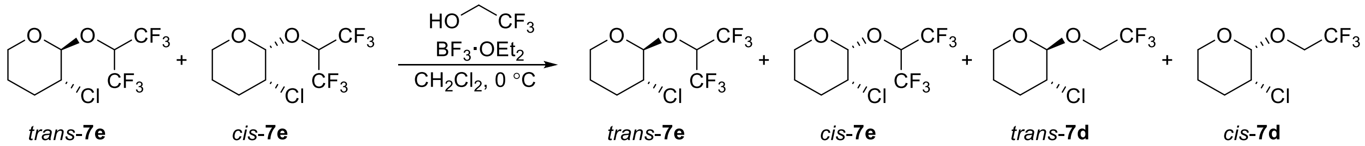

Epimerization studies on acetal **7e** were performed following the representative procedure using acetal **7e** (0.019 g, 0.066 mmol, dr >99:1), 2,2,2-trifluoroethanol (25 µL, 0.35 mmol), and $BF_3 \cdot OEt_2$ (21 µL, 0.17 mmol) in CH_2Cl_2 (1 mL) at 0 °C for 1 h. ^{1}H NMR and ^{13}C{^{1}H} NMR spectroscopic analysis of the unpurified reaction mixture revealed an 87:13 mixture of products (**7e**:**7d**). Acetal **7e** was recovered as a >99:1 mixture of diastereomers. Acetal **7a** was formed as a 70:30 mixture of diastereomers.

Note: Epimerization occurred with acetal **7e** with $BF_3 \cdot OEt_2$ at 0 °C. The observance of the substitution product indicates that acetal formation was reversible with acetal **7e** with $BF_3 \cdot OEt_2$ at 0 °C.

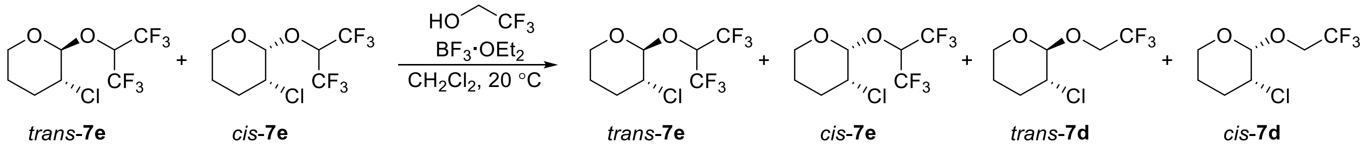

Epimerization studies on acetal **7e** were performed following the representative procedure using acetal **7e** (0.023 g, 0.080 mmol, dr >99:1), 2,2,2-trifluoroethanol (25 µL, 0.35 mmol), and $BF_3{\cdot}OEt_2$ (21 µL, 0.17 mmol) in CH_2Cl_2 (1 mL) at 20 °C for 1 h. ^{1}H NMR and $^{13}C\{^1H\}$ NMR spectroscopic analysis of the unpurified reaction mixture revealed a 5:95 mixture of products (**7e**:**7d**). Acetal **7e** was recovered as a >99:1 mixture of diastereomers. Acetal **7d** was formed as a 65:35 mixture of diastereomers.

Note: Epimerization occurred with acetal **7e** with $BF_3{\cdot}OEt_2$ at 20 °C. The observance of the substitution product indicates that acetal formation was reversible with acetal **7e** with $BF_3{\cdot}OEt_2$ at 20 °C.

Conclusion for Acetal 7e: Under the conditions listed in Table 2 ($BF_3{\cdot}OEt_2$ at –78 °C), epimerization did not occur. Epimerization occurred at higher temperatures (≥ 0 °C), however. Acetal formation was also reversible at higher temperatures (≥ 0 °C).

B. α-Brominated Pyrans

a. Acetal 8a

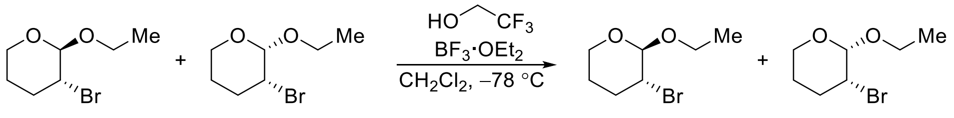

Epimerization studies on acetal **8a** were performed following the representative procedure using acetal **8a** (0.041 g, 0.20 mmol, dr 96:4), 2,2,2-trifluoroethanol (55 µL, 0.76 mmol), and $BF_3{\cdot}OEt_2$ (50 µL, 0.40 mmol) in CH_2Cl_2 (2 mL) at –78 °C for 1 h. ^{1}H NMR and $^{13}C\{^1H\}$ NMR spectroscopic analysis of the unpurified reaction mixture revealed a 100:0 mixture of products (**8a**:**8d**). Acetal **8a** was recovered as a 96:4 mixture of diastereomers.

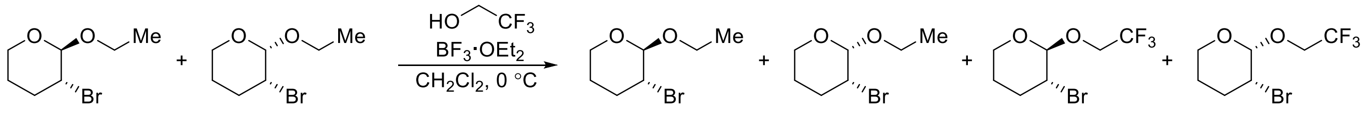

Epimerization studies on acetal **8a** were performed following the representative procedure using acetal **8a** (0.057 g, 0.27 mmol, dr 96:4), 2,2,2-trifluoroethanol (70 µL, 0.97 mmol), and $BF_3{\cdot}OEt_2$ (60 µL, 0.48 mmol) in CH_2Cl_2 (2 mL) at 0 °C for 1 h. ^{1}H NMR and $^{13}C\{^1H\}$ NMR spectroscopic analysis of the unpurified reaction mixture revealed a 12:88 mixture of products (**8a**:**8d**). Acetal **8a** was recovered as a >99:1 mixture of diastereomers. Acetal **8d** was formed as a 56:44 mixture of diastereomers.

Note: Epimerization occurred with acetal **8a** with $BF_3·OEt_2$ at 0 °C. The observance of the substitution product indicates that acetal formation was reversible with acetal **8a** with $BF_3·OEt_2$ at 0 °C.

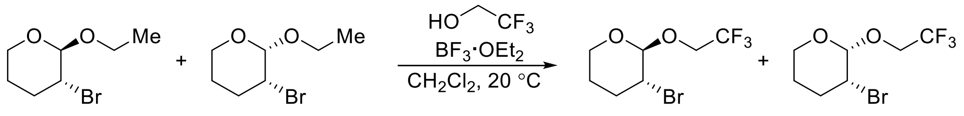

trans-**8a** *cis*-**8a** *trans*-**8d** *cis*-**8d**

Epimerization studies on acetal **8a** were performed following the representative procedure using acetal **8a** (0.051 g, 0.25 mmol, dr 96:4), 2,2,2-trifluoroethanol (70 µL, 0.97 mmol), and $BF_3·OEt_2$ (60 µL, 0.48 mmol) in CH_2Cl_2 (2 mL) at 20 °C for 1 h. ^{1}H NMR and ^{13}C{^{1}H} NMR spectroscopic analysis of the unpurified reaction mixture revealed a 0:100 mixture of products (**8a**:**8d**). Acetal **8d** was formed as a 63:37 mixture of diastereomers.

Note: The observance of the substitution product indicates that acetal formation was reversible with acetal **8a** with $BF_3·OEt_2$ at 20 °C.

Conclusion for Acetal 8a: Under the conditions listed in Table 2 ($BF_3·OEt_2$ at –78 °C), epimerization did not occur. Epimerization occurred at higher temperatures (≥ 0 °C), however. Acetal formation was also reversible at higher temperatures (≥ 0 °C).

b. Acetal 8b

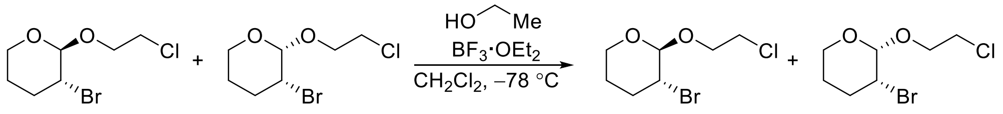

trans-**8b** *cis*-**8b** *trans*-**8b** *cis*-**8b**

Epimerization studies on acetal **8b** were performed following the representative procedure using acetal **8b** (0.070 g, 0.29 mmol, dr 61:39), ethanol (60 µL, 1.0 mmol), and $BF_3·OEt_2$ (60 µL, 0.48 mmol) in CH_2Cl_2 (3 mL) at –78 °C for 1 h. ^{1}H NMR and ^{13}C{^{1}H} NMR spectroscopic analysis of the unpurified reaction mixture revealed a 100:0 mixture of products (**8b**:**8a**). Acetal **8b** was recovered as a 66:34 mixture of diastereomers.

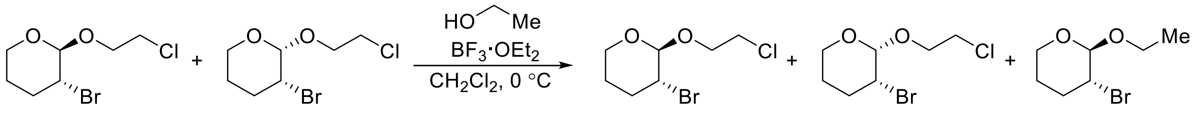

trans-**8b** *cis*-**8b** *trans*-**8b** *cis*-**8b** *trans*-**8a**

Epimerization studies on acetal **8b** were performed following the representative procedure using acetal **8b** (0.061 g, 0.25 mmol, dr 61:39), ethanol (60 µL, 1.0 mmol), and $BF_3·OEt_2$ (60 µL, 0.48 mmol) in

CH_2Cl_2 (3 mL) at 0 °C for 1 h. ^{1}H NMR and ^{13}C{^{1}H} NMR spectroscopic analysis of the unpurified reaction mixture revealed a 77:23 mixture of products (**8b**:**8a**). Acetal **8b** was recovered as a 61:39 mixture of diastereomers. Acetal **8a** was formed as a >99:1 mixture of diastereomers.

Note: The observance of the substitution product indicates that acetal formation was reversible with acetal **8b** with $BF_3{\cdot}OEt_2$ at 0 °C.

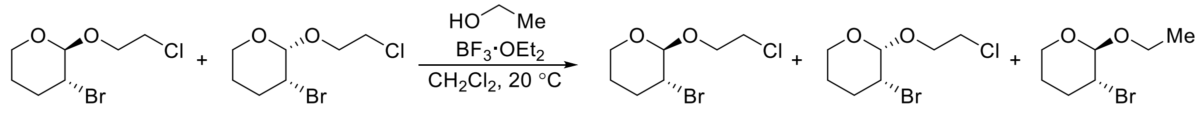

Epimerization studies on acetal **8b** were performed following the representative procedure using acetal **8b** (0.066 g, 0.27 mmol, dr 61:39), ethanol (60 µL, 1.0 mmol), and $BF_3{\cdot}OEt_2$ (60 µL, 0.48 mmol) in CH_2Cl_2 (3 mL) at 20 °C for 1 h. ^{1}H NMR and ^{13}C{^{1}H} NMR spectroscopic analysis of the unpurified reaction mixture revealed a 65:35 mixture of products (**8b**:**8a**). Acetal **8b** was recovered as a 59:41 mixture of diastereomers. Acetal **8a** was formed as a >99:1 mixture of diastereomers.

Note: Epimerization occurred with acetal **8b** with $BF_3{\cdot}OEt_2$ at 20 °C. The observance of the substitution product indicates that acetal formation was reversible with acetal **8b** with $BF_3{\cdot}OEt_2$ at 20 °C.

Conclusion for Acetal 8b: Under the conditions listed in Table 2 ($BF_3{\cdot}OEt_2$ at –78 °C), epimerization did not occur. Epimerization occurred at higher temperatures (≥ 0 °C), however. Acetal formation was also reversible at higher temperatures (≥ 0 °C).

c. Acetal 8c

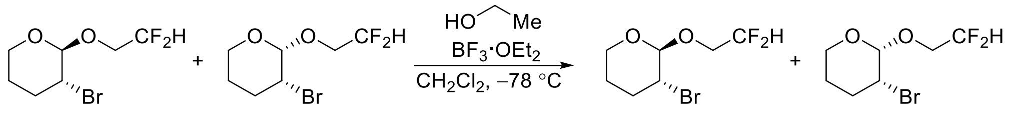

Epimerization studies on acetal **8c** were performed following the representative procedure using acetal **8c** (0.035 g, 0.14 mmol, dr 68:32), ethanol (35 µL, 0.60 mmol), and $BF_3{\cdot}OEt_2$ (35 µL, 0.28 mmol) in CH_2Cl_2 (2 mL) at –78 °C for 1 h. ^{1}H NMR and ^{13}C{^{1}H} NMR spectroscopic analysis of the unpurified reaction mixture revealed a 100:0 mixture of products (**8c**:**8a**). Acetal **8c** was recovered as a 65:35 mixture of diastereomers.

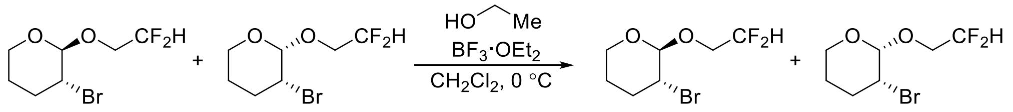

Epimerization studies on acetal **8c** were performed following the representative procedure using acetal **8c** (0.037 g, 0.15 mmol, dr 68:32), ethanol (35 µL, 0.60 mmol), and $BF_3 \cdot OEt_2$ (35 µL, 0.28 mmol) in CH_2Cl_2 (2 mL) at 0 °C for 1 h. ^1H NMR and $^{13}C\{^1H\}$ NMR spectroscopic analysis of the unpurified reaction mixture revealed a 100:0 mixture of products (**8c**:**8a**). Acetal **8c** was recovered as a 64:36 mixture of diastereomers.

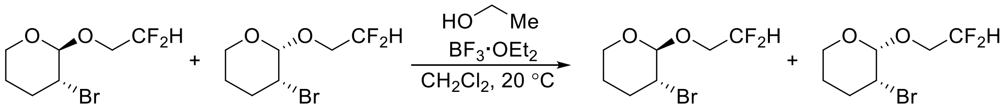

trans-**8c** *cis*-**8c** *trans*-**8c** *cis*-**8c**

Epimerization studies on acetal **8c** were performed following the representative procedure using acetal **8c** (0.038 g, 0.16 mmol, dr 68:32), ethanol (35 µL, 0.60 mmol), and $BF_3 \cdot OEt_2$ (35 µL, 0.28 mmol) in CH_2Cl_2 (2 mL) at 20 °C for 1 h. ^1H NMR and $^{13}C\{^1H\}$ NMR spectroscopic analysis of the unpurified reaction mixture revealed a 100:0 mixture of products (**8c**:**8a**). Acetal **8c** was recovered as a 64:36 mixture of diastereomers.

Conclusion for Acetal 8c: Under the conditions listed in Table 2 ($BF_3 \cdot OEt_2$ at –78 °C), epimerization did not occur. Epimerization also did not occur at higher temperatures (≥ 0 °C).

d. Acetal 8d

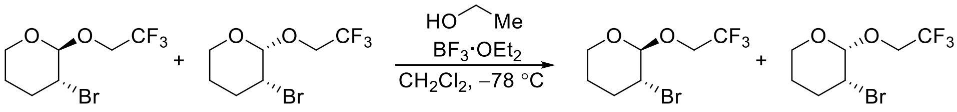

trans-**8d** *cis*-**8d** *trans*-**8d** *cis*-**8d**

Epimerization studies on acetal **8d** were performed following the representative procedure using acetal **8d** (0.036 g, 0.13 mmol, dr 71:29), ethanol (26 µL, 0.45 mmol), and $BF_3 \cdot OEt_2$ (30 µL, 0.24 mmol) in CH_2Cl_2 (1 mL) at –78 °C for 1 h. ^1H NMR and $^{13}C\{^1H\}$ NMR spectroscopic analysis of the unpurified reaction mixture revealed a 100:0 mixture of products (**8d**:**8a**). Acetal **8d** was recovered as a 70:30 mixture of diastereomers.

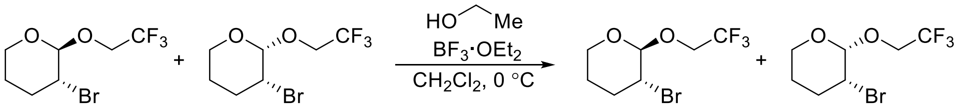

trans-**8d** *cis*-**8d** *trans*-**8d** *cis*-**8d**

Epimerization studies on acetal **8d** were performed following the representative procedure using acetal **8d** (0.035 g, 0.14 mmol, dr 71:29), ethanol (26 µL, 0.45 mmol), and $BF_3 \cdot OEt_2$ (30 µL, 0.24 mmol) in

CH_2Cl_2 (1 mL) at 0 °C for 1 h. ^1H NMR and $^{13}C\{^1H\}$ NMR spectroscopic analysis of the unpurified reaction mixture revealed a 100:0 mixture of products (**8d**:**8a**). Acetal **8d** was recovered as a 71:29 mixture of diastereomers.

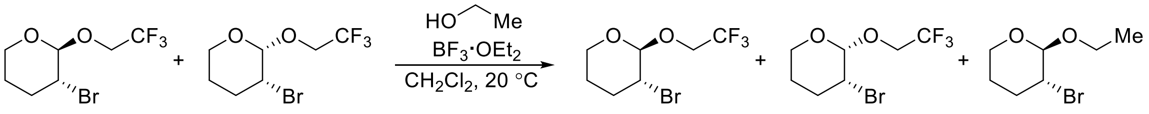

trans-**8d** *cis*-**8d** *trans*-**8d** *cis*-**8d** *trans*-**8a**

Epimerization studies on acetal **8d** were performed following the representative procedure using acetal **8d** (0.034 g, 0.13 mmol, dr 71:29), ethanol (26 µL, 0.45 mmol), and $BF_3{\cdot}OEt_2$ (30 µL, 0.24 mmol) in CH_2Cl_2 (1 mL) at 20 °C for 1 h. ^1H NMR and $^{13}C\{^1H\}$ NMR spectroscopic analysis of the unpurified reaction mixture revealed a 97:3 mixture of products (**8d**:**8a**). Acetal **8d** was recovered as a 68:32 mixture of diastereomers. Acetal **8a** was formed as a >99:1 mixture of diastereomers.

Note: The observance of the substitution product indicates that acetal formation was reversible with acetal **8d** with $BF_3{\cdot}OEt_2$ at 20 °C.

Conclusion for Acetal 8d: Under the conditions listed in Table 2 ($BF_3{\cdot}OEt_2$ at –78 °C), neither epimerization nor acetal substitution occurred. They also did not occur at 0 °C. At higher temperatures (≥ 20 °C), traces of acetal substitution product **8a** were observed. Should acetal **8d** ionize to form an oxocarbenium ion intermediate, the reaction of this intermediate to form either acetal **8a** or **8d** depends upon the concentration and nucleophilicity of the alcohol nucleophile. The concentration of ethanol in solution (4 equiv) is higher than the concentration of 2,2,2-trifluoroethanol that would be formed. Ethanol is also more nucleophilic than 2,2,2-triflurorothanol due to the absence of electron-withdrawing groups. As a result, the rate of addition of ethanol to the oxocarbenium ion intermediate would be faster than the rate of addition of 2,2,2-trifluoroethanol. Consequently, acetal **8d** would likely undergo substitution to form **8a** faster than it would epimerize to change the diastereomer ratio of **8d**.

e. Acetal 8e

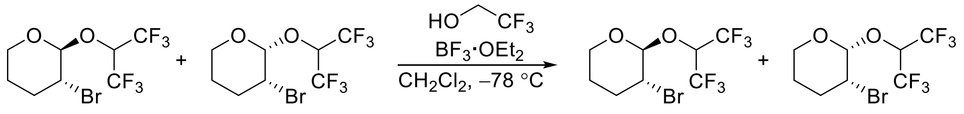

trans-**8e** *cis*-**8e** *trans*-**8e** *cis*-**8e**

Epimerization studies on acetal **8e** were performed following the representative procedure using acetal **8e** (0.021 g, 0.063 mmol, dr >99:1), 2,2,2-trifluoroethanol (20 µL, 0.28 mmol), and $BF_3{\cdot}OEt_2$ (15 µL, 0.12 mmol) in CH_2Cl_2 (1 mL) at –78 °C for 1 h. ^1H NMR and $^{13}C\{^1H\}$ NMR spectroscopic analysis of the

unpurified reaction mixture revealed a 100:0 mixture of products (**8e**:**8d**). Acetal **8e** was recovered as a >99:1 mixture of diastereomers.

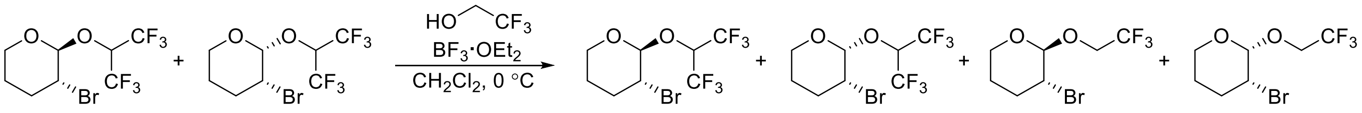

trans-**8e** *cis*-**8e** *trans*-**8e** *cis*-**8e** *trans*-**8d** *cis*-**8d**

Epimerization studies on acetal **8e** were performed following the representative procedure using acetal **8e** (0.021 g, 0.063 mmol, dr >99:1), 2,2,2-trifluoroethanol (20 µL, 0.28 mmol), and $BF_3{\cdot}OEt_2$ (15 µL, 0.12 mmol) in CH_2Cl_2 (1 mL) at 0 °C for 1 h. ^1H NMR and $^{13}C\{^1H\}$ NMR spectroscopic analysis of the unpurified reaction mixture revealed a 38:62 mixture of products (**8e**:**8d**). Acetal **8e** was recovered as a >99:1 mixture of diastereomers. Acetal **8d** was formed as a 69:31 mixture of diastereomers.

Note: The observance of the substitution product indicates that acetal formation was reversible with acetal **8e** with $BF_3{\cdot}OEt_2$ at 0 °C.

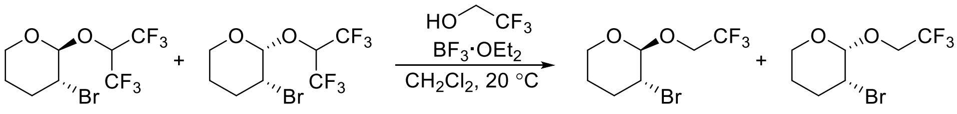

trans-**8e** *cis*-**8e** *trans*-**8d** *cis*-**8d**

Epimerization studies on acetal **8e** were performed following the representative procedure using acetal **8e** (0.021 g, 0.063 mmol, dr >99:1), 2,2,2-trifluoroethanol (20 µL, 0.28 mmol), and $BF_3{\cdot}OEt_2$ (15 µL, 0.12 mmol) in CH_2Cl_2 (1 mL) at 20 °C for 1 h. ^1H NMR and $^{13}C\{^1H\}$ NMR spectroscopic analysis of the unpurified reaction mixture revealed a 0:100 mixture of products (**8e**:**8d**). Acetal **8d** was formed as a 62:38 mixture of diastereomers.

Note: The observance of the substitution product indicates that acetal formation was reversible with acetal **8e** with $BF_3{\cdot}OEt_2$ at 20 °C.

Conclusion for Acetal 8e: Under the conditions listed in Table 2 ($BF_3{\cdot}OEt_2$ at –78 °C), neither epimerization nor acetal substitution occurred. At higher temperatures (0 °C), acetal substitution product **8d** was observed, but epimerization of **8e** was not observed. Should acetal **8e** ionize to form an oxocarbenium ion intermediate, the reaction of this intermediate to form either acetal **8e** or **8d** depends upon the concentration and nucleophilicity of the alcohol nucleophile. The concentration of 2,2,2-trifluroroethanol in solution (4 equiv) is higher than the concentration of hexafluoroisopropanol that would be formed. 2,2,2-Trifluroroethanol is also more nucleophilic than hexafluoroisopropanol because it has fewer electron-withdrawing groups. As a result, the rate of addition of 2,2,2-trifluroroethanol to the oxocarbenium ion intermediate would be faster than the rate of addition of

hexafluoroisopropanol. Consequently, acetal **8e** would likely undergo substitution to form **8d** faster than it would epimerize to change the diastereomer ratio of **8e**.

C. α-Fluorinated Pyrans

a. Acetal 10a

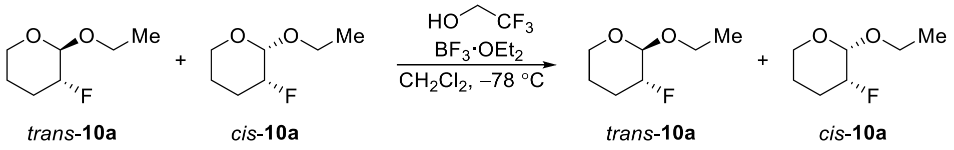

Representative procedure for epimerization studies.

To a cooled (–78 °C) solution of acetal **10a** (0.019 g, 0.13 mmol, dr 70:30) and 2,2,2-trifluoroethanol (30 µL, 0.42 mmol) in CH_2Cl_2 (1 mL) was added $BF_3{\cdot}OEt_2$ (25 µL, 0.20 mmol) dropwise over 2 min. After 1 h, Et_3N (20 µL) was added. The mixture was then warmed to 20 °C and concentrated *in vacuo*. ^1H NMR and $^{13}C\{^1H\}$ NMR spectroscopic analysis of the unpurified reaction mixture revealed a 100:0 mixture of products (**10a**:**10d**). Acetal **10a** was recovered as a 72:28 mixture of diastereomers.

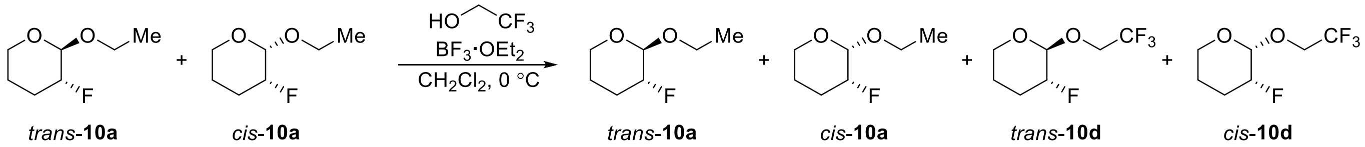

Epimerization studies on acetal **10a** were performed following the representative procedure using acetal **10a** (0.013 g, 0.088 mmol, dr 70:30), 2,2,2-trifluoroethanol (30 µL, 0.42 mmol), and $BF_3{\cdot}OEt_2$ (25 µL, 0.20 mmol) in CH_2Cl_2 (1 mL) at 0 °C for 1 h. ^1H NMR and $^{13}C\{^1H\}$ NMR spectroscopic analysis of the unpurified reaction mixture revealed a 76:24 mixture of products (**10a**:**10d**). Acetal **10a** was recovered as a 71:29 mixture of diastereomers. Acetal **10d** was formed as a 61:39 mixture of diastereomers.

Note: Epimerization occurred with acetal **10a** with $BF_3{\cdot}OEt_2$ at 0 °C.

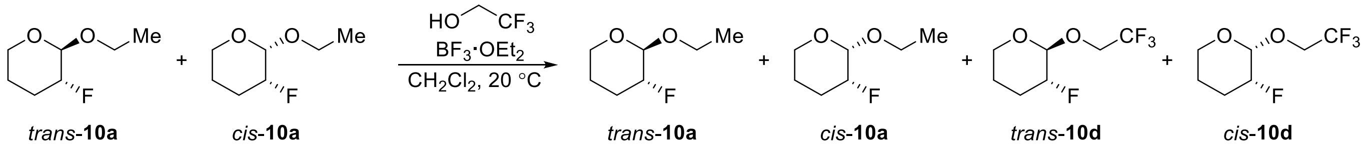

Epimerization studies on acetal **10a** were performed following the representative procedure using acetal **10a** (0.015 g, 0.10 mmol, dr 70:30), 2,2,2-trifluoroethanol (30 µL, 0.42 mmol), and BF_3·OEt_2 (25 µL, 0.20 mmol) in CH_2Cl_2 (1 mL) at 20 °C for 1 h. ^{1}H NMR and ^{13}C{^{1}H} NMR spectroscopic analysis of the unpurified reaction mixture revealed a 12:88 mixture of products (**10a**:**10d**). Acetal **10a** was recovered as a 81:19 mixture of diastereomers. Acetal **10d** was formed as a 72:28 mixture of diastereomers.

Note: Epimerization occurred with acetal **10a** with BF_3·OEt_2 at 20 °C.

Conclusion for Acetal 10a: Under the conditions listed in Table 4 (BF_3·OEt_2 at –78 °C), epimerization did not occur. Epimerization did occur at higher temperatures (≥ 0 °C), however.

b. Acetal 10b

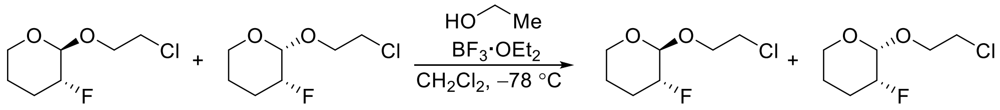

trans-**10b** *cis*-**10b** *trans*-**10b** *cis*-**10b**

Epimerization studies on acetal **10b** were performed following the representative procedure using acetal **10b** (0.014 g, 0.077 mmol, dr 64:32), ethanol (20 µL, 0.34 mmol), and BF_3·OEt_2 (20 µL, 0.16 mmol) in CH_2Cl_2 (1 mL) at –78 °C for 1 h. ^{1}H NMR and ^{13}C{^{1}H} NMR spectroscopic analysis of the unpurified reaction mixture revealed a 100:0 mixture of products (**10b**:**10a**). Acetal **10b** was recovered as a 66:34 mixture of diastereomers.

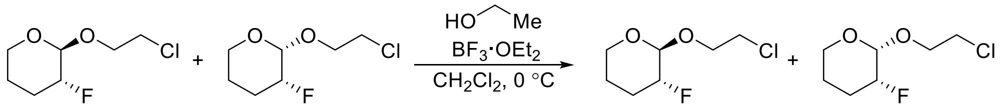

trans-**10b** *cis*-**10b** *trans*-**10b** *cis*-**10b**

Epimerization studies on acetal **10b** were performed following the representative procedure using acetal **10b** (0.015 g, 0.082 mmol, dr 64:32), ethanol (20 µL, 0.34 mmol), and BF_3·OEt_2 (20 µL, 0.16 mmol) in CH_2Cl_2 (1 mL) at 0 °C for 1 h. ^{1}H NMR and ^{13}C{^{1}H} NMR spectroscopic analysis of the unpurified reaction mixture revealed a 100:0 mixture of products (**10b**:**10a**). Acetal **10b** was recovered as a 65:35 mixture of diastereomers.

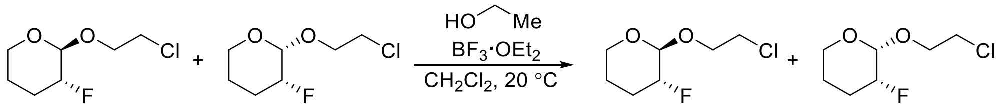

trans-**10b** *cis*-**10b** *trans*-**10b** *cis*-**10b**

Epimerization studies on acetal **10b** were performed following the representative procedure using acetal **10b** (0.015 g, 0.082 mmol, dr 64:32), ethanol (20 µL, 0.34 mmol), and $BF_3 \cdot OEt_2$ (20 µL, 0.16 mmol) in CH_2Cl_2 (1 mL) at 20 °C for 1 h. ^{1}H NMR and ^{13}C{^{1}H} NMR spectroscopic analysis of the unpurified reaction mixture revealed a 100:0 mixture of products (**10b**:**10a**). Acetal **10b** was recovered as a 65:35 mixture of diastereomers.

Conclusion for Acetal 10b: Under the conditions listed in Table 4 ($BF_3 \cdot OEt_2$ at –78 °C), epimerization did not occur. Epimerization also did not occur at higher temperatures (≥ 0 °C).

c. Acetal 10c

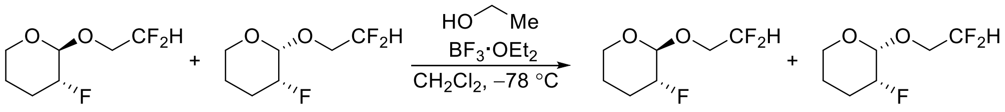

trans-**10c** *cis*-**10c** *trans*-**10c** *cis*-**10c**

Epimerization studies on acetal **10c** were performed following the representative procedure using acetal **10c** (0.016 g, 0.087 mmol, dr >99:1), ethanol (20 µL, 0.34 mmol), and $BF_3 \cdot OEt_2$ (20 µL, 0.16 mmol) in CH_2Cl_2 (1 mL) at –78 °C for 1 h. ^{1}H NMR and ^{13}C{^{1}H} NMR spectroscopic analysis of the unpurified reaction mixture revealed a 100:0 mixture of products (**10c**:**10a**). Acetal **10c** was recovered as a >99:1 mixture of diastereomers.

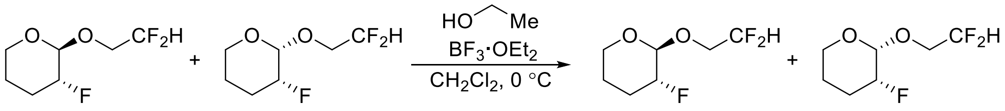

trans-**10c** *cis*-**10c** *trans*-**10c** *cis*-**10c**

Epimerization studies on acetal **10c** were performed following the representative procedure using acetal **10c** (0.016 g, 0.087 mmol, dr >99:1), ethanol (20 µL, 0.34 mmol), and $BF_3 \cdot OEt_2$ (20 µL, 0.16 mmol) in CH_2Cl_2 (1 mL) at 0 °C for 1 h. ^{1}H NMR and ^{13}C{^{1}H} NMR spectroscopic analysis of the unpurified reaction mixture revealed a 100:0 mixture of products (**10c**:**10a**). Acetal **10c** was recovered as a >99:1 mixture of diastereomers.

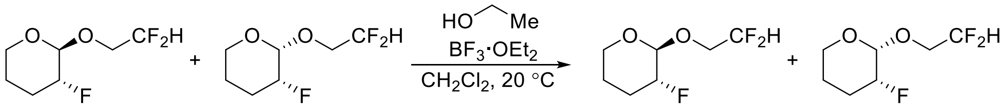

trans-**10c** *cis*-**10c** *trans*-**10c** *cis*-**10c**

Epimerization studies on acetal **10c** were performed following the representative procedure using acetal **10c** (0.017 g, 0.092 mmol, dr >99:1), ethanol (20 µL, 0.34 mmol), and $BF_3 \cdot OEt_2$ (20 µL, 0.16

mmol) in CH_2Cl_2 (1 mL) at 20 °C for 1 h. ^1H NMR and $^{13}C\{^1H\}$ NMR spectroscopic analysis of the unpurified reaction mixture revealed a 100:0 mixture of products (**10c**:**10a**). Acetal **10c** was recovered as a >99:1 mixture of diastereomers.

Conclusion for Acetal 10c: Under the conditions listed in Table 4 ($BF_3{\cdot}OEt_2$ at –78 °C), epimerization did not occur. Epimerization also did not occur at higher temperatures (≥ 0 °C).

d. Acetal 10d

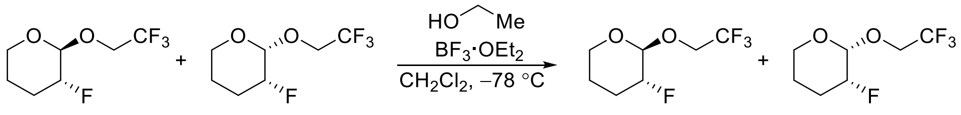

trans-**10d** *cis*-**10d** *trans*-**10d** *cis*-**10d**

Epimerization studies on acetal **10d** were performed following the representative procedure using acetal **10d** (0.018 g, 0.089 mmol, dr >99:1), ethanol (20 µL, 0.34 mmol), and $BF_3{\cdot}OEt_2$ (20 µL, 0.16 mmol) in CH_2Cl_2 (1 mL) at –78 °C for 1 h. ^1H NMR and $^{13}C\{^1H\}$ NMR spectroscopic analysis of the unpurified reaction mixture revealed a 100:0 mixture of products (**10d**:**10a**). Acetal **10d** was recovered as a >99:1 mixture of diastereomers.

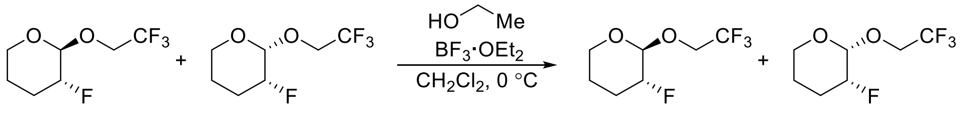

trans-**10d** *cis*-**10d** *trans*-**10d** *cis*-**10d**

Epimerization studies on acetal **10d** were performed following the representative procedure using acetal **10d** (0.014 g, 0.069 mmol, dr >99:1), ethanol (20 µL, 0.34 mmol), and $BF_3{\cdot}OEt_2$ (20 µL, 0.16 mmol) in CH_2Cl_2 (1 mL) at 0 °C for 1 h. ^1H NMR and $^{13}C\{^1H\}$ NMR spectroscopic analysis of the unpurified reaction mixture revealed a 100:0 mixture of products (**10d**:**10a**). Acetal **10d** was recovered as a >99:1 mixture of diastereomers.

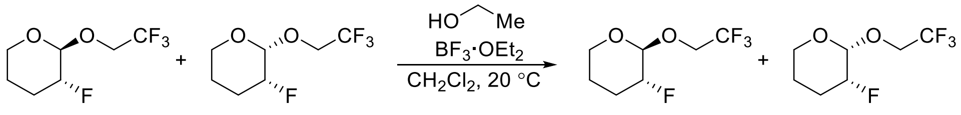

trans-**10d** *cis*-**10d** *trans*-**10d** *cis*-**10d**

Epimerization studies on acetal **10d** were performed following the representative procedure using acetal **10d** (0.016 g, 0.079 mmol, dr >99:1), ethanol (20 µL, 0.34 mmol), and $BF_3{\cdot}OEt_2$ (20 µL, 0.16 mmol) in CH_2Cl_2 (1 mL) at 20 °C for 1 h. ^1H NMR and $^{13}C\{^1H\}$ NMR spectroscopic analysis of the

unpurified reaction mixture revealed a 100:0 mixture of products (**10d**:**10a**). Acetal **10d** was recovered as a >99:1 mixture of diastereomers.

Conclusion for Acetal 10d: Under the conditions listed in Table 4 (BF_3·OEt_2 at –78 °C), epimerization did not occur. Epimerization also did not occur at higher temperatures (≥ 0 °C).

e. Acetal 10e

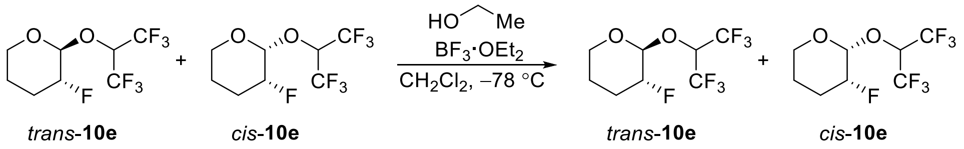

Epimerization studies on acetal **10e** were performed following the representative procedure using acetal **10e** (0.011 g, 0.041 mmol, dr 1:>99), ethanol (10 µL, 0.17 mmol), and BF_3·OEt_2 (10 µL, 0.080 mmol) in CH_2Cl_2 (1 mL) at –78 °C for 1 h. ^{1}H NMR and ^{13}C{^{1}H} NMR spectroscopic analysis of the unpurified reaction mixture revealed a 100:0 mixture of products (**10e**:**10a**). Acetal **10d** was recovered as a 1:>99 mixture of diastereomers.

Conclusion for Acetal 10e: Under the conditions listed in Table 4 (BF_3·OEt_2 at –78 °C), epimerization did not occur.

D. α-Chlorinated Furans

a. Acetal 19a

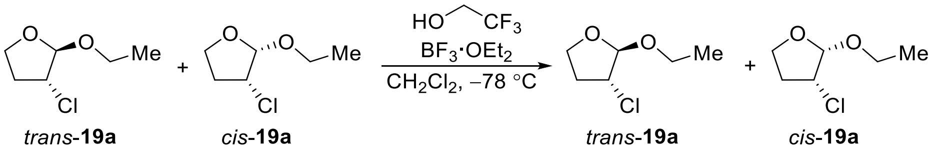

Epimerization studies on acetal **19a** were performed following the representative procedure using acetal **19a** (0.049 g, 0.33 mmol, dr 94:6), 2,2,2-trifluoroethanol (95 µL, 1.3 mmol), and BF_3·OEt_2 (80 µL, 0.664 mmol) in CH_2Cl_2 (3 mL) at –78 °C for 1 h. ^{1}H NMR and ^{13}C{^{1}H} NMR spectroscopic analysis of the unpurified reaction mixture revealed a 100:0 mixture of products (**19a**:**19d**). Acetal **19a** was recovered as a 95:5 mixture of diastereomers.

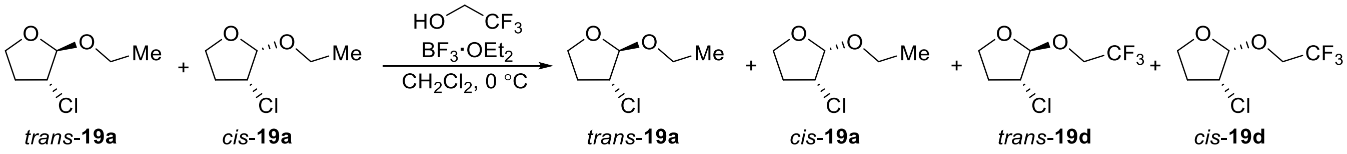

Epimerization studies on acetal **19a** were performed following the representative procedure using acetal **19a** (0.053 g, 0.35 mmol, dr 94:6), 2,2,2-trifluoroethanol (95 µL, 1.3 mmol), and $BF_3{\cdot}OEt_2$ (80 µL, 0.664 mmol) in CH_2Cl_2 (3 mL) at 0 °C for 1 h. ^{1}H NMR and $^{13}C\{^1H\}$ NMR spectroscopic analysis of the unpurified reaction mixture revealed a 0:100 mixture of products (**19a**:**19d**). Acetal **19d** was formed as a 95:5 mixture of diastereomers.

Note: The observance of the substitution product indicates that acetal formation was reversible with acetal **19a** with $BF_3{\cdot}OEt_2$ at 0 °C.

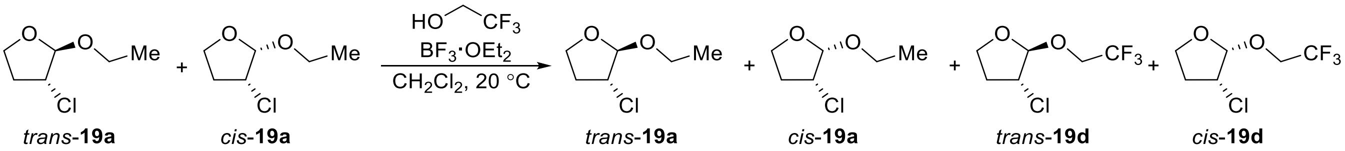

Epimerization studies on acetal **19a** were performed following the representative procedure using acetal **19a** (0.051 g, 0.34 mmol, dr 94:6), 2,2,2-trifluoroethanol (95 µL, 1.3 mmol), and $BF_3{\cdot}OEt_2$ (80 µL, 0.664 mmol) in CH_2Cl_2 (3 mL) at 20 °C for 1 h. ^{1}H NMR and $^{13}C\{^1H\}$ NMR spectroscopic analysis of the unpurified reaction mixture revealed a 0:100 mixture of products (**19a**:**19d**). Acetal **19d** was formed as a 94:6 mixture of diastereomers.

Note: The observance of the substitution product indicates that acetal formation was reversible with acetal **19a** with $BF_3{\cdot}OEt_2$ at 20 °C.

Conclusion for Acetal 19a: Under the conditions listed in Table 5 ($BF_3{\cdot}OEt_2$ at –78 °C), epimerization did not occur. Epimerization also did not occur at higher temperatures (≥ 0 °C). Acetal formation was reversible at higher temperatures (≥ 0 °C), however.

b. Acetal 19a

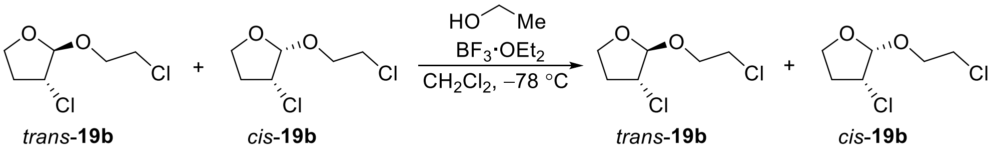

Epimerization studies on acetal **19b** were performed following the representative procedure using acetal **19b** (0.061 g, 0.33 mmol, dr >99:1), ethanol (75 µL, 1.3 mmol), and $BF_3{\cdot}OEt_2$ (80 µL, 0.64 mmol) in CH_2Cl_2 (3 mL) at –78 °C for 1 h. ^{1}H NMR and $^{13}C\{^1H\}$ NMR spectroscopic analysis of the unpurified reaction mixture revealed a 100:0 mixture of products (**19b**:**19a**). Acetal **19b** was recovered as a >99:1 mixture of diastereomers.

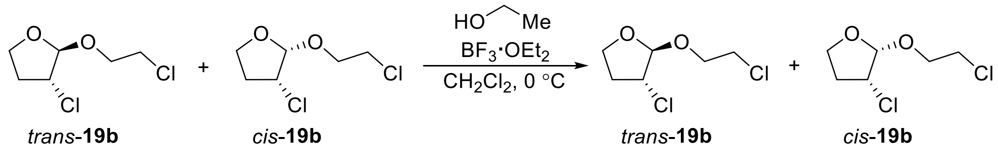

Epimerization studies on acetal **19b** were performed following the representative procedure using acetal **19b** (0.062 g, 0.34 mmol, dr >99:1), ethanol (75 µL, 1.3 mmol), and $BF_3{\cdot}OEt_2$ (80 µL, 0.64 mmol) in CH_2Cl_2 (3 mL) at 0 °C for 1 h. ^1H NMR and $^{13}C\{^1H\}$ NMR spectroscopic analysis of the unpurified reaction mixture revealed a 100:0 mixture of products (**19b**:**19a**). Acetal **19b** was recovered as a >99:1 mixture of diastereomers.

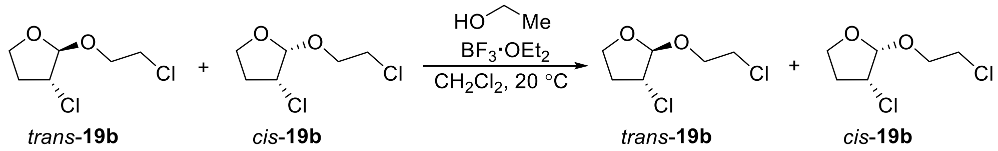

Epimerization studies on acetal **19b** were performed following the representative procedure using acetal **19b** (0.061 g, 0.33 mmol, dr >99:1), ethanol (75 µL, 1.3 mmol), and $BF_3{\cdot}OEt_2$ (80 µL, 0.64 mmol) in CH_2Cl_2 (3 mL) at 20 °C for 1 h. ^1H NMR and $^{13}C\{^1H\}$ NMR spectroscopic analysis of the unpurified reaction mixture revealed a 100:0 mixture of products (**19b**:**19a**). Acetal **19b** was recovered as a >99:1 mixture of diastereomers.

Conclusion for Acetal 19b: Under the conditions listed in Table 5 ($BF_3{\cdot}OEt_2$ at –78 °C), epimerization did not occur. Epimerization also did not occur at higher temperatures (≥ 0 °C).

c. Acetal 19c

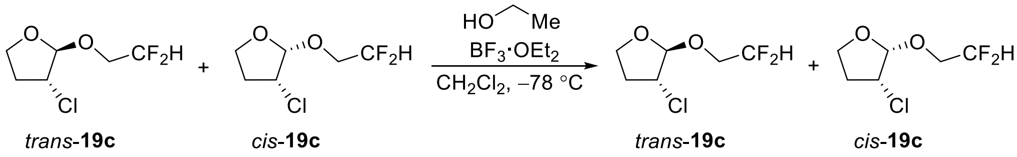

Epimerization studies on acetal **19c** were performed following the representative procedure using acetal **19c** (0.029 g, 0.16 mmol, dr 1:>99), ethanol (40 µL, 0.69 mmol), and $BF_3{\cdot}OEt_2$ (40 µL, 0.32 mmol) in CH_2Cl_2 (1.5 mL) at –78 °C for 1 h. ^1H NMR and $^{13}C\{^1H\}$ NMR spectroscopic analysis of the unpurified reaction mixture revealed a 100:0 mixture of products (**19c**:**19a**). Acetal **19c** was recovered as a 1:>99 mixture of diastereomers.

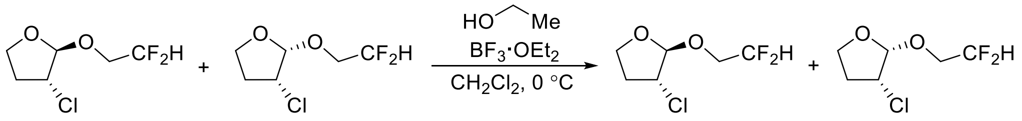

trans-**19c** *cis*-**19c** *trans*-**19c** *cis*-**19c**

Epimerization studies on acetal **19c** were performed following the representative procedure using acetal **19c** (0.031 g, 0.17 mmol, dr 1:>99), ethanol (40 µL, 0.69 mmol), and $BF_3{\cdot}OEt_2$ (40 µL, 0.32 mmol) in CH_2Cl_2 (1.5 mL) at 0 °C for 1 h. ^{1}H NMR and ^{13}C{^{1}H} NMR spectroscopic analysis of the unpurified reaction mixture revealed a 100:0 mixture of products (**19c**:**19a**). Acetal **19c** was recovered as a 1:>99 mixture of diastereomers.

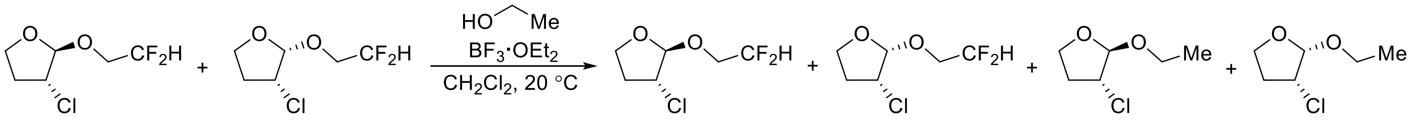

trans-**19c** *cis*-**19c** *trans*-**19c** *cis*-**19c** *trans*-**19a** *cis*-**19a**

Epimerization studies on acetal **19c** were performed following the representative procedure using acetal **19c** (0.030 g, 0.16 mmol, dr 1:>99), ethanol (40 µL, 0.69 mmol), and $BF_3{\cdot}OEt_2$ (40 µL, 0.32 mmol) in CH_2Cl_2 (1.5 mL) at 20 °C for 1 h. ^{1}H NMR and ^{13}C{^{1}H} NMR spectroscopic analysis of the unpurified reaction mixture revealed a 47:53 mixture of products (**19c**:**19a**). Acetal **19c** was recovered as a 77:23 mixture of diastereomers. Acetal **19a** was formed as a 94:6 mixture of diastereomers.

Note: Epimerization occurred with acetal **19c** with $BF_3{\cdot}OEt_2$ at 20 °C. The observance of the substitution product indicates that acetal formation was reversible with acetal **19c** with $BF_3{\cdot}OEt_2$ at 20 °C.

Conclusion for Acetal 19c: Under the conditions listed in Table 5 ($BF_3{\cdot}OEt_2$ at –78 °C), epimerization did not occur. Epimerization occurred at higher temperatures (≥ 20 °C), however. Acetal formation was also reversible at higher temperatures (≥ 20 °C).

d. Acetal 19d

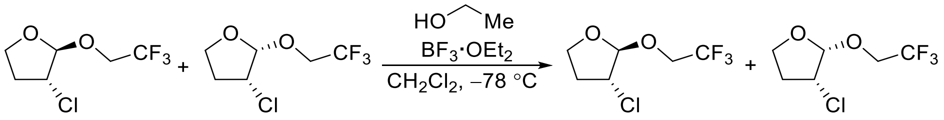

trans-**19d** *cis*-**19d** *trans*-**19d** *cis*-**19d**

Epimerization studies on acetal **19d** were performed following the representative procedure using acetal **19d** (0.033 g, 0.16 mmol, dr 4:96), ethanol (35 µL, 0.60 mmol), and $BF_3{\cdot}OEt_2$ (40 µL, 0.32 mmol)

in CH_2Cl_2 (2 mL) at –78 °C for 1 h. ^{1}H NMR and ^{13}C{^{1}H} NMR spectroscopic analysis of the unpurified reaction mixture revealed a 100:0 mixture of products (**19d**:**19a**). Acetal **19d** was recovered as a 4:96 mixture of diastereomers.

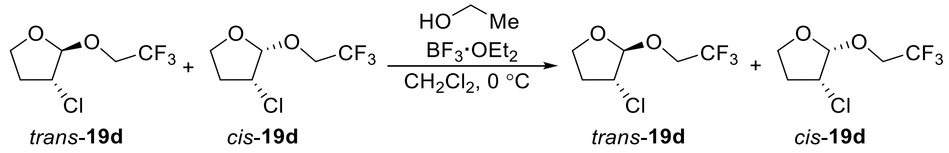

Epimerization studies on acetal **19d** were performed following the representative procedure using acetal **19d** (0.032 g, 0.16 mmol, dr 4:96), ethanol (35 µL, 0.60 mmol), and $BF_3{\cdot}OEt_2$ (40 µL, 0.32 mmol) in CH_2Cl_2 (2 mL) at 0 °C for 1 h. ^{1}H NMR and ^{13}C{^{1}H} NMR spectroscopic analysis of the unpurified reaction mixture revealed a 100:0 mixture of products (**19d**:**19a**). Acetal **19d** was recovered as a 2:98 mixture of diastereomers.

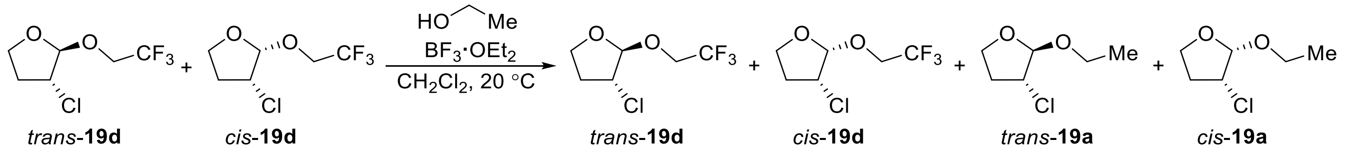

Epimerization studies on acetal **19d** were performed following the representative procedure using acetal **19d** (0.030 g, 0.15 mmol, dr 4:96), ethanol (35 µL, 0.60 mmol), and $BF_3{\cdot}OEt_2$ (40 µL, 0.32 mmol) in CH_2Cl_2 (2 mL) at 20 °C for 1 h. ^{1}H NMR and ^{13}C{^{1}H} NMR spectroscopic analysis of the unpurified reaction mixture revealed an 89:11 mixture of products (**19d**:**19a**). Acetal **19d** was recovered as a 5:95 mixture of diastereomers. Acetal **19a** was formed as an 83:17 mixture of diastereomers.

Note: Epimerization occurred with acetal **19d** with $BF_3{\cdot}OEt_2$ at 20 °C. The observance of the substitution product indicates that acetal formation was reversible with acetal **19d** with $BF_3{\cdot}OEt_2$ at 20 °C.

Conclusion for Acetal 19d: Under the conditions listed in Table 5 ($BF_3{\cdot}OEt_2$ at –78 °C), epimerization did not occur. Epimerization occurred at higher temperatures (≥ 20 °C), however. Acetal formation was also reversible at higher temperatures (≥ 20 °C).

e. Acetal 19e

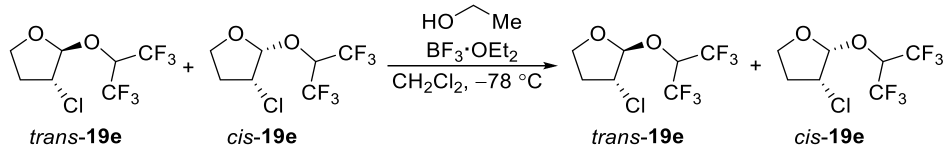

Epimerization studies on acetal **19e** were performed following the representative procedure using acetal **19e** (0.056 g, 0.21 mmol, dr >99:1), ethanol (45 µL, 0.77 mmol), and $BF_3·OEt_2$ (45 µL, 0.36 mmol) in CH_2Cl_2 (3 mL) at –78 °C for 1 h. ^{1}H NMR and ^{13}C{^{1}H} NMR spectroscopic analysis of the unpurified reaction mixture revealed a 100:0 mixture of products (**19e**:**19a**). Acetal **19e** was recovered as a >99:1 mixture of diastereomers.

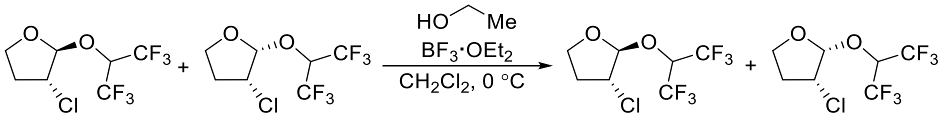

trans-**19e** *cis*-**19e** *trans*-**19e** *cis*-**19e**

Epimerization studies on acetal **19e** were performed following the representative procedure using acetal **19e** (0.059 g, 0.22 mmol, dr >99:1), ethanol (45 µL, 0.77 mmol), and $BF_3·OEt_2$ (45 µL, 0.36 mmol) in CH_2Cl_2 (3 mL) at 0 °C for 1 h. ^{1}H NMR and ^{13}C{^{1}H} NMR spectroscopic analysis of the unpurified reaction mixture revealed a 100:0 mixture of products (**19e**:**19a**). Acetal **19e** was recovered as a >99:1 mixture of diastereomers.

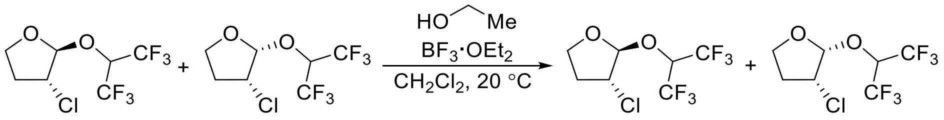

trans-**19e** *cis*-**19e** *trans*-**19e** *cis*-**19e**

Epimerization studies on acetal **19e** were performed following the representative procedure using acetal **19e** (0.051 g, 0.19 mmol, dr >99:1), ethanol (45 µL, 0.77 mmol), and $BF_3·OEt_2$ (45 µL, 0.36 mmol) in CH_2Cl_2 (3 mL) at 0 °C for 1 h. ^{1}H NMR and ^{13}C{^{1}H} NMR spectroscopic analysis of the unpurified reaction mixture revealed a 100:0 mixture of products (**19e**:**19a**). Acetal **19e** was recovered as a >99:1 mixture of diastereomers.

Conclusion for Acetal 19e: Under the conditions listed in Table 5 ($BF_3·OEt_2$ at –78 °C), epimerization did not occur. Epimerization also did not occur at higher temperatures (≥ 0 °C).

E. α-Brominated Furans

a. Acetal 20a

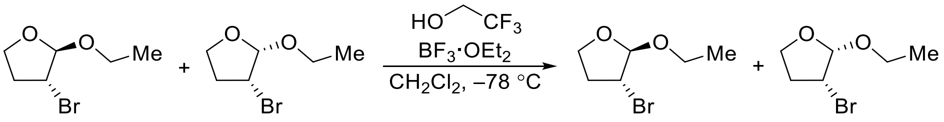

trans-**20a** *cis*-**20a** *trans*-**20a** *cis*-**20a**

Epimerization studies on acetal **20a** were performed following the representative procedure using acetal **20a** (0.057 g, 0.29 mmol, dr >99:1), 2,2,2-trifluoroethanol (90 µL, 1.3 mmol), and $BF_3{\cdot}OEt_2$ (80 µL, 0.64 mmol) in CH_2Cl_2 (3 mL) at –78 °C for 1 h. ^1H NMR and $^{13}C\{^1H\}$ NMR spectroscopic analysis of the unpurified reaction mixture revealed a 100:0 mixture of products (**20a**:**20d**). Acetal **20a** was recovered as a >99:1 mixture of diastereomers.

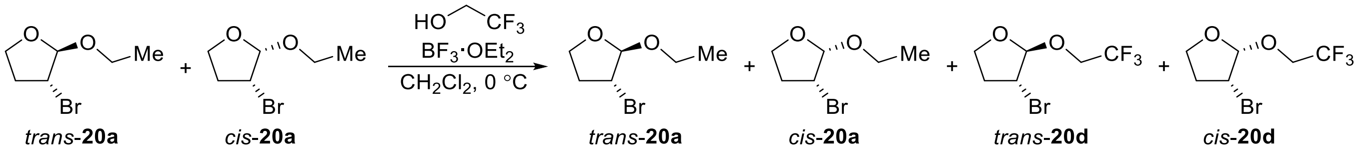

Epimerization studies on acetal **20a** were performed following the representative procedure using acetal **20a** (0.060 g, 0.31 mmol, dr >99:1), 2,2,2-trifluoroethanol (90 µL, 1.3 mmol), and $BF_3{\cdot}OEt_2$ (80 µL, 0.64 mmol) in CH_2Cl_2 (3 mL) at 0 °C for 1 h. ^1H NMR and $^{13}C\{^1H\}$ NMR spectroscopic analysis of the unpurified reaction mixture revealed a 2:98 mixture of products (**20a**:**20d**). Acetal **20a** was recovered as a >99:1 mixture of diastereomers. Acetal **20d** was formed as a >99:1 mixture of diastereomers.

Note: The observance of the substitution product indicates that acetal formation was reversible with acetal **20a** with $BF_3{\cdot}OEt_2$ at 0 °C.

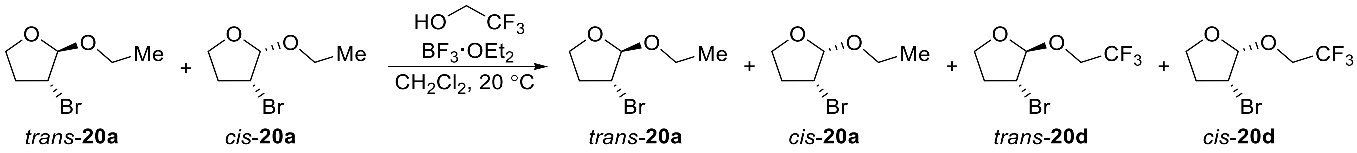

Epimerization studies on acetal **20a** were performed following the representative procedure using acetal **20a** (0.064 g, 0.33 mmol, dr >99:1), 2,2,2-trifluoroethanol (90 µL, 1.3 mmol), and $BF_3{\cdot}OEt_2$ (80 µL, 0.64 mmol) in CH_2Cl_2 (3 mL) at 20 °C for 1 h. ^1H NMR and $^{13}C\{^1H\}$ NMR spectroscopic analysis of the unpurified reaction mixture revealed a 6:94 mixture of products (**20a**:**20d**). Acetal **20a** was recovered as a >99:1 mixture of diastereomers. Acetal **20d** was formed as a >99:1 mixture of diastereomers.

Note: The observance of the substitution product indicates that acetal formation was reversible with acetal **20a** with $BF_3{\cdot}OEt_2$ at 20 °C.

Conclusion for Acetal 20a: Under the conditions listed in Table 5 ($BF_3{\cdot}OEt_2$ at –78 °C), epimerization did not occur. Epimerization also did not occur at higher temperatures (≥ 0 °C). Acetal formation was reversible at higher temperatures (≥ 0 °C), however.

b. Acetal 20b

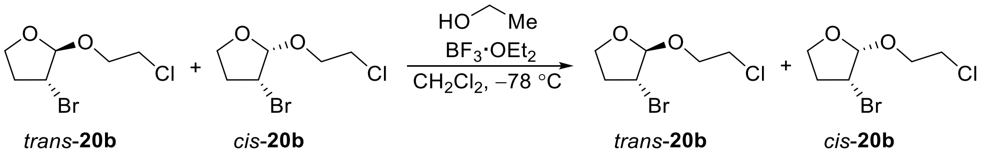

Epimerization studies on acetal **20b** were performed following the representative procedure using acetal **20b** (0.061 g, 0.27 mmol, dr 97:3), ethanol (60 µL, 1.0 mmol), and BF_3·OEt_2 (75 µL, 0.60 mmol) in CH_2Cl_2 (3 mL) at –78 °C for 1 h. ^1H NMR and $^{13}C\{^1H\}$ NMR spectroscopic analysis of the unpurified reaction mixture revealed a 100:0 mixture of products (**20b**:**20a**). Acetal **20b** was recovered as a >99:1 mixture of diastereomers.

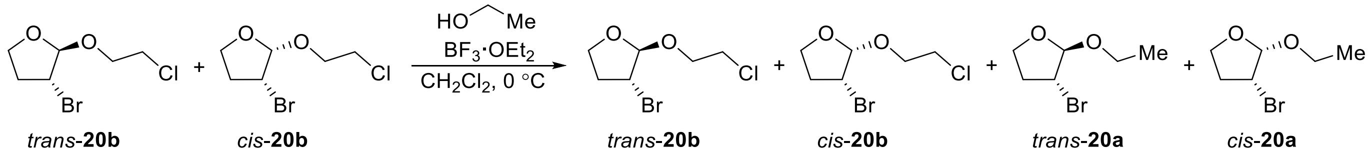

Epimerization studies on acetal **20b** were performed following the representative procedure using acetal **20b** (0.064 g, 0.28 mmol, dr 97:3), ethanol (60 µL, 1.0 mmol), and BF_3·OEt_2 (75 µL, 0.60 mmol) in CH_2Cl_2 (3 mL) at 0 °C for 1 h. ^1H NMR and $^{13}C\{^1H\}$ NMR spectroscopic analysis of the unpurified reaction mixture revealed a 85:15 mixture of products (**20b**:**20a**). Acetal **20b** was recovered as a 96:4 mixture of diastereomers. Acetal **20a** was formed as a 92:8 mixture of diastereomers.

Note: The observance of the substitution product indicates that acetal formation was reversible with acetal **20b** with BF_3·OEt_2 at 0 °C.

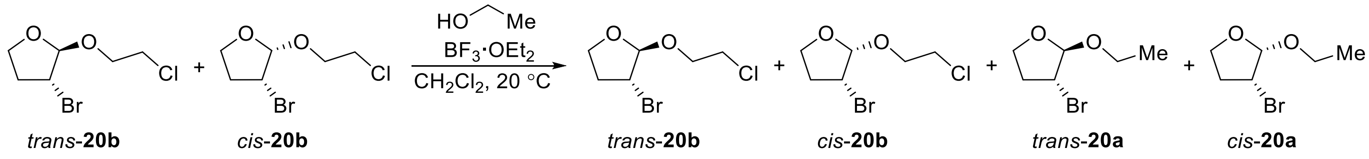

Epimerization studies on acetal **20b** were performed following the representative procedure using acetal **20b** (0.060 g, 0.26 mmol, dr 97:3), ethanol (60 µL, 1.0 mmol), and BF_3·OEt_2 (75 µL, 0.60 mmol) in CH_2Cl_2 (3 mL) at 20 °C for 1 h. ^1H NMR and $^{13}C\{^1H\}$ NMR spectroscopic analysis of the unpurified reaction mixture revealed a 31:69 mixture of products (**20b**:**20a**). Acetal **20b** was recovered as an 84:16 mixture of diastereomers. Acetal **20a** was formed as a 94:6 mixture of diastereomers.

Note: Epimerization occurred with acetal **20b** with $BF_3{\cdot}OEt_2$ at 20 °C. The observance of the substitution product indicates that acetal formation was reversible with acetal **20b** with $BF_3{\cdot}OEt_2$ at 20 °C.

Conclusion for Acetal 20b: Under the conditions listed in Table 5 ($BF_3{\cdot}OEt_2$ at –78 °C), epimerization did not occur. Epimerization occurred at higher temperatures (≥ 20 °C), however. Acetal formation was also reversible at higher temperatures (≥ 0 °C).

c. Acetal 20c

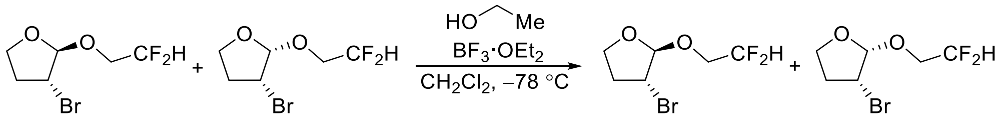

trans-**20c** *cis*-**20c** *trans*-**20c** *cis*-**20c**

Epimerization studies on acetal **20c** were performed following the representative procedure using acetal **20c** (0.062 g, 0.27 mmol, dr >99:1), ethanol (61 µL, 1.0 mmol), and $BF_3{\cdot}OEt_2$ (75 µL, 0.60 mmol) in CH_2Cl_2 (3 mL) at –78 °C for 1 h. ^1H NMR and $^{13}C\{^1H\}$ NMR spectroscopic analysis of the unpurified reaction mixture revealed a 100:0 mixture of products (**20c**:**20a**). Acetal **20c** was recovered as a >99:1 mixture of diastereomers.

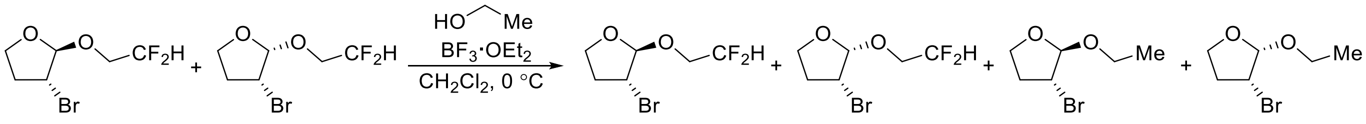

trans-**20c** *cis*-**20c** *trans*-**20c** *cis*-**20c** *trans*-**20a** *cis*-**20a**

Epimerization studies on acetal **20c** were performed following the representative procedure using acetal **20c** (0.059 g, 0.26 mmol, dr >99:1), ethanol (61 µL, 1.0 mmol), and $BF_3{\cdot}OEt_2$ (75 µL, 0.60 mmol) in CH_2Cl_2 (3 mL) at 0 °C for 1 h. ^1H NMR and $^{13}C\{^1H\}$ NMR spectroscopic analysis of the unpurified reaction mixture revealed an 85:15 mixture of products (**20c**:**20a**). Acetal **20c** was recovered as a >99:1 mixture of diastereomers. Acetal **20a** was formed as a 93:7 mixture of diastereomers.

Note: The observance of the substitution product indicates that acetal formation was reversible with acetal **20c** with $BF_3{\cdot}OEt_2$ at 0 °C.

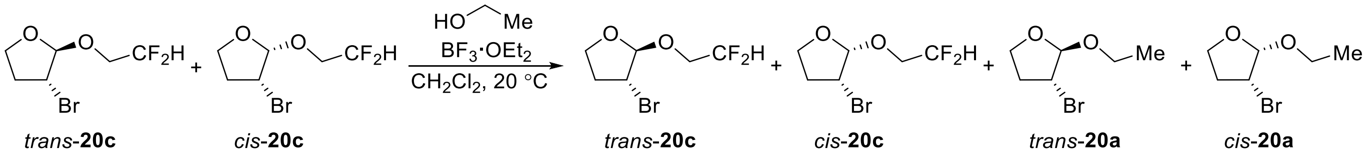

Epimerization studies on acetal **20c** were performed following the representative procedure using acetal **20c** (0.060 g, 0.26 mmol, dr >99:1), ethanol (61 µL, 1.0 mmol), and $BF_3·OEt_2$ (75 µL, 0.60 mmol) in CH_2Cl_2 (3 mL) at 20 °C for 1 h. ^{1}H NMR and ^{13}C{^{1}H} NMR spectroscopic analysis of the unpurified reaction mixture revealed a 27:73 mixture of products (**20c**:**20a**). Acetal **20c** was recovered as a >99:1 mixture of diastereomers. Acetal **20a** was formed as a 93:7 mixture of diastereomers.

Note: The observance of the substitution product indicates that acetal formation was reversible with acetal **20c** with $BF_3·OEt_2$ at 20 °C.

Conclusion for Acetal 20c: Under the conditions listed in Table 5 ($BF_3·OEt_2$ at –78 °C), epimerization did not occur. Epimerization also did not occur at higher temperatures (≥ 0 °C). Acetal formation was reversible at higher temperatures (≥ 0 °C), however.

d. Acetal 20d

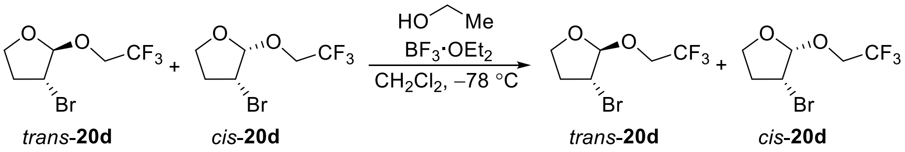

Epimerization studies on acetal **20d** were performed following the representative procedure using acetal **20d** (0.061 g, 0.24 mmol, dr >99:1), ethanol (56 µL, 0.96 mmol), and $BF_3·OEt_2$ (60 µL, 0.48 mmol) in CH_2Cl_2 (3 mL) at –78 °C for 1 h. ^{1}H NMR and ^{13}C{^{1}H} NMR spectroscopic analysis of the unpurified reaction mixture revealed a 100:0 mixture of products (**20d**:**20a**). Acetal **20d** was recovered as a >99:1 mixture of diastereomers.

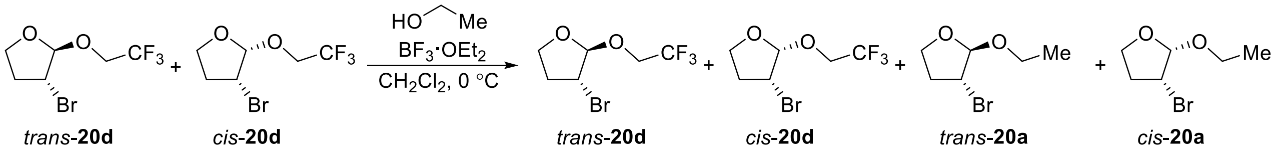

Epimerization studies on acetal **20d** were performed following the representative procedure using acetal **20d** (0.063 g, 0.25 mmol, dr >99:1), ethanol (56 µL, 0.96 mmol), and $BF_3·OEt_2$ (60 µL, 0.48 mmol) in CH_2Cl_2 (3 mL) at 0 °C for 1 h. ^{1}H NMR and ^{13}C{^{1}H} NMR spectroscopic analysis of the

unpurified reaction mixture revealed a 96:4 mixture of products (**20d**:**20a**). Acetal **20d** was recovered as a >99:1 mixture of diastereomers. Acetal **20a** was formed as a >99:1 mixture of diastereomers.

Note: The observance of the substitution product indicates that acetal formation was reversible with acetal **20d** with $BF_3{\cdot}OEt_2$ at 0 °C.

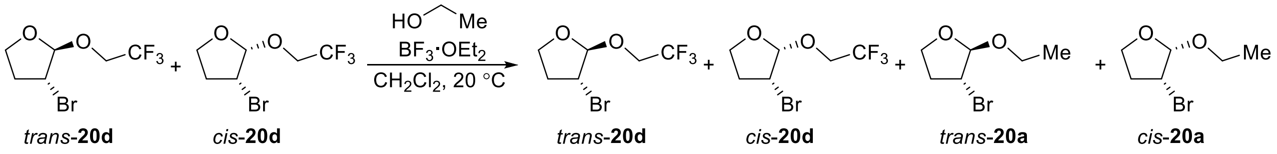

Epimerization studies on acetal **20d** were performed following the representative procedure using acetal **20d** (0.061 g, 0.24 mmol, dr >99:1), ethanol (56 µL, 0.96 mmol), and $BF_3{\cdot}OEt_2$ (60 µL, 0.48 mmol) in CH_2Cl_2 (3 mL) at 20 °C for 1 h. ^{1}H NMR and ^{13}C{^{1}H} NMR spectroscopic analysis of the unpurified reaction mixture revealed a 15:85 mixture of products (**20d**:**20a**). Acetal **20d** was recovered as a >99:1 mixture of diastereomers. Acetal **20a** was formed as a 94:6 mixture of diastereomers.

Note: The observance of the substitution product indicates that acetal formation was reversible with acetal **20d** with $BF_3{\cdot}OEt_2$ at 20 °C.

Conclusion for Acetal 20d: Under the conditions listed in Table 5 ($BF_3{\cdot}OEt_2$ at –78 °C), epimerization did not occur. Epimerization also did not occur at higher temperatures (≥ 0 °C). Acetal formation was reversible at higher temperatures (≥ 0 °C), however.

e. Acetal 20e

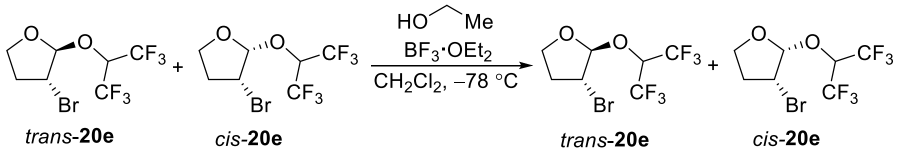

Epimerization studies on acetal **20e** were performed following the representative procedure using acetal **20e** (0.019 g, 0.060 mmol, dr >99:1), ethanol (15 µL, 0.26 mmol), and $BF_3{\cdot}OEt_2$ (15 µL, 0.12 mmol) in CH_2Cl_2 (1 mL) at –78 °C for 1 h. ^{1}H NMR and ^{13}C{^{1}H} NMR spectroscopic analysis of the unpurified reaction mixture revealed a 100:0 mixture of products (**20e**:**20a**). Acetal **20e** was recovered as a >99:1 mixture of diastereomers.

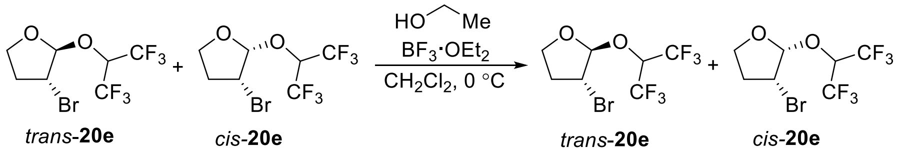

Epimerization studies on acetal **20e** were performed following the representative procedure using acetal **20e** (0.022 g, 0.069 mmol, dr >99:1), ethanol (15 µL, 0.26 mmol), and $BF_3{\cdot}OEt_2$ (15 µL, 0.12 mmol) in CH_2Cl_2 (1 mL) at 0 °C for 1 h. ^1H NMR and $^{13}C\{^1H\}$ NMR spectroscopic analysis of the unpurified reaction mixture revealed a 100:0 mixture of products (**20e**:**20a**). Acetal **20e** was recovered as a >99:1 mixture of diastereomers.

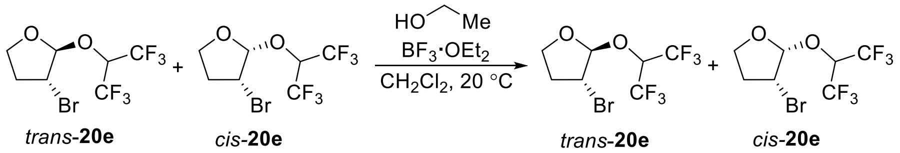

Epimerization studies on acetal **20e** were performed following the representative procedure using acetal **20e** (0.019 g, 0.060 mmol, dr >99:1), ethanol (15 µL, 0.26 mmol), and $BF_3{\cdot}OEt_2$ (15 µL, 0.12 mmol) in CH_2Cl_2 (1 mL) at 0 °C for 1 h. ^1H NMR and $^{13}C\{^1H\}$ NMR spectroscopic analysis of the unpurified reaction mixture revealed a 100:0 mixture of products (**20e**:**20a**). Acetal **20e** was recovered as a >99:1 mixture of diastereomers.

Conclusion for Acetal 20e: Under the conditions listed in Table 5 ($BF_3{\cdot}OEt_2$ at –78 °C), epimerization did not occur. Epimerization also did not occur at higher temperatures (≥ 0 °C).

VI. Kinetic Experiments

A. General Procedure

A reported procedure[11] was used to determine the rates of hydrolysis for acetals **1b**, **1c**, **11**, and **13**. A stock solution (1 mL, 0.2 M) of acetal (0.2 mmol) and 1,3-dimethyoxybenzene (0.075 mmol) in acetone-d_6 was prepared. A stock solution of DCl in D_2O (1.0 mL, 2.52 M) was prepared from a commercially available solution of DCl (35 wt. % in D_2O).

B. Rate Data Collection

A solution of acetal and 1,3-dimethyoxybenzene in acetone-d_6 (500 µL, 0.2 M) was added to a NMR tube and a ^{1}H NMR one-pulse spectrum was acquired. The stock solution of DCl in D_2O (125 µL, 2.52 M) was then added to the NMR tube, the NMR tube was inverted, and a second ^{1}H NMR one-pulse spectrum was acquired. The sample remained in the probe and ^{1}H NMR one-pulse experiments were performed at regular time increments (every 5 or 10 min) with the probe temperature held constant at 25 °C. Hydrolysis rates were calculated by following the decrease in acetal concentration and plotting ln [acetal] against time (sec). Rate constants were determined from the slope of the resulting graph.

C. Kinetic Data

The following kinetic graphs were used to determine the rates of hydrolysis of acetals **1b**, **1c**, **11**, **13**, *anti*-**1b**, *syn*-**1b**, *anti*-**1c**, and *syn*-**1b**, as represented in Schemes 2 and 3. All experiments were performed in triplicate, and the average rate was used. Each run is denoted by a trendline, which is representative of k_{obs}. The rates of acetal hydrolysis were determined using the equation $k = k_{obs}/[DCl]$. The rates of hydrolysis of the *trans*- and *cis*-anomers of acetals **1b** and **1c** was extrapolated from the kinetic data from acetals **1b** and **1c**, respectively.

Graph **S1**. Rate of hydrolysis of acetal **1b** plotted as ln [acetal] versus time (sec) over approximately four half-lives. Rate $k = 3.139 \times 10^{-5}$.

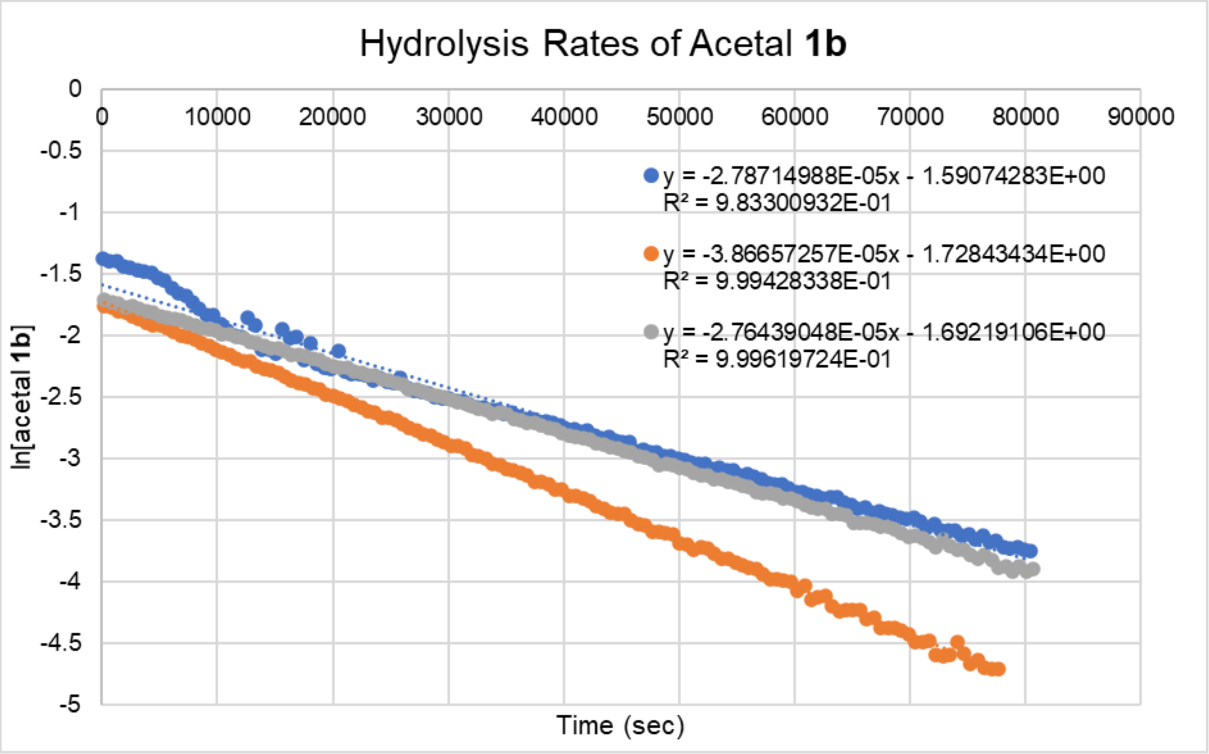

Graph **S2**. Rate of hydrolysis of acetal **1c** plotted as ln [acetal] versus time (sec) over approximately three half-lives. Rate $k = 1.258 \times 10^{-4}$.

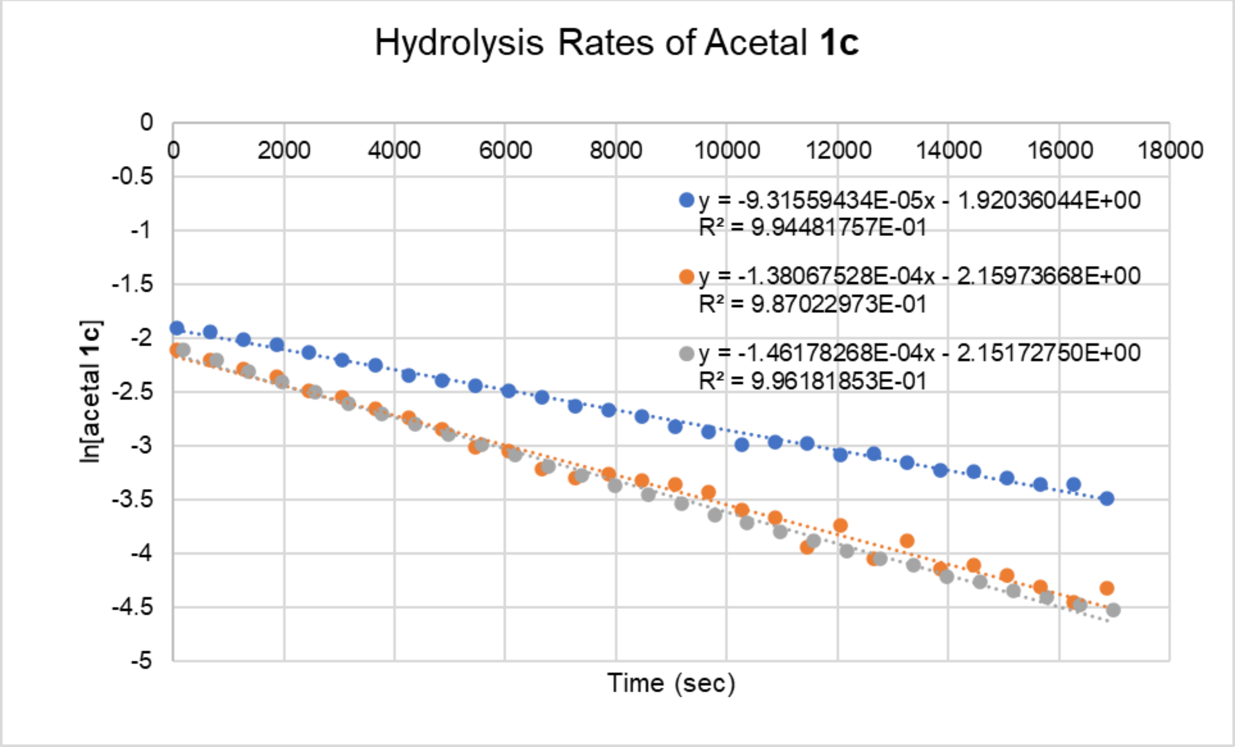

Graph **S3**. Rate of hydrolysis of acetal **11** plotted as ln [acetal] versus time (sec) over approximately six half-lives. Rate $k = 1.183 \times 10^{-4}$.

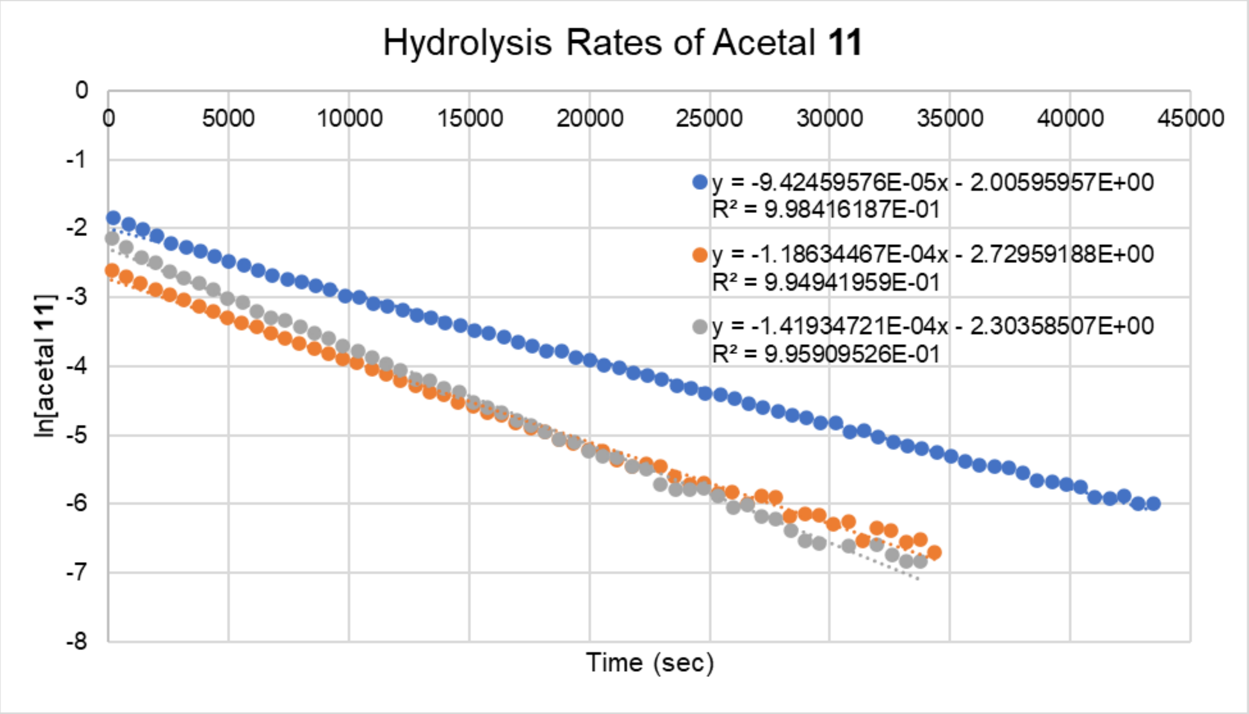

Graph **S4**. Rate of hydrolysis of acetal **13** plotted as ln [acetal] versus time (sec) over approximately six half-lives. Rate $k = 9.210 \times 10^{-4}$.

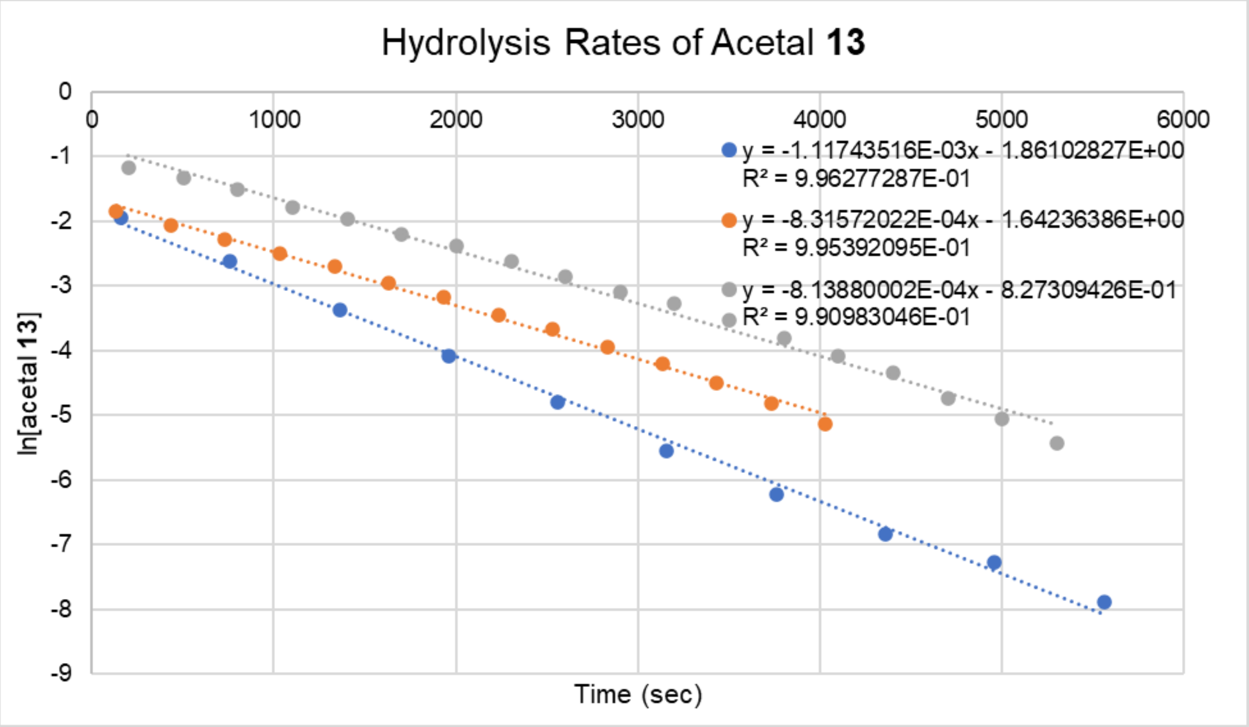

Graph **S5**. Rate of hydrolysis of acetal *anti*-**1b** plotted as ln [acetal] versus time (sec) over approximately four half-lives. Rate $k = 3.167 \times 10^{-5}$.

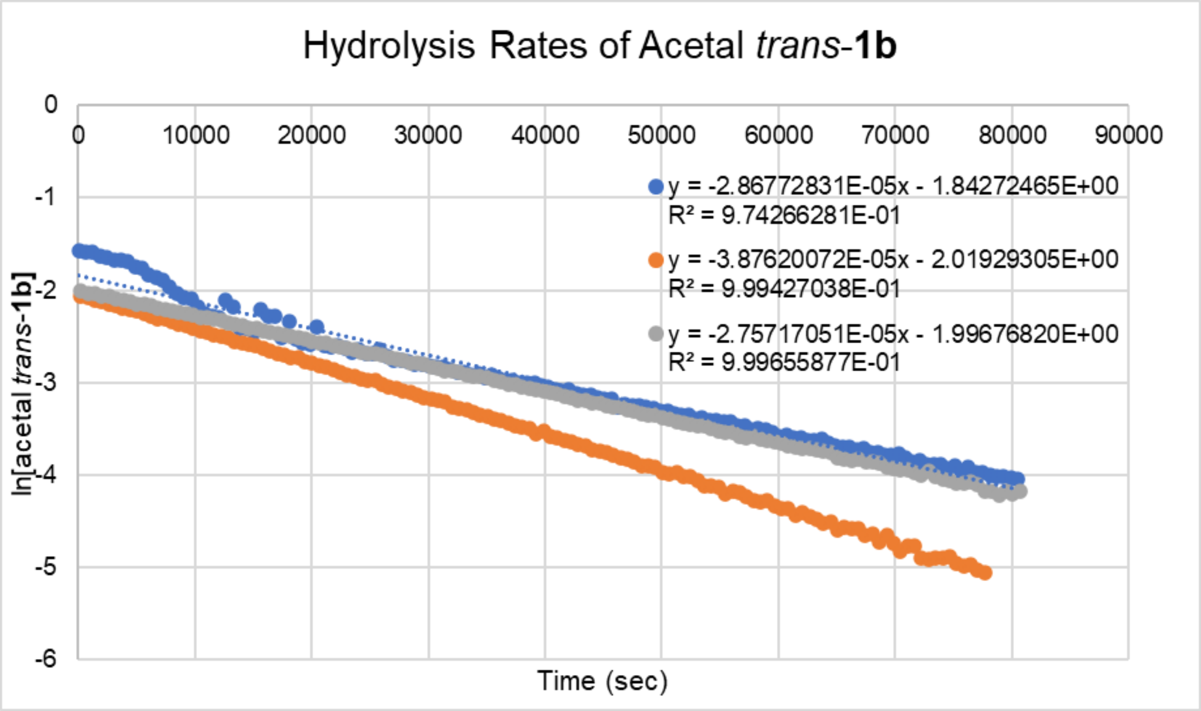

Graph **S6**. Rate of hydrolysis of acetal *cis*-**1b** plotted as ln [acetal] versus time (sec) over approximately four half-lives. Rate $k = 3.073 \times 10^{-5}$.

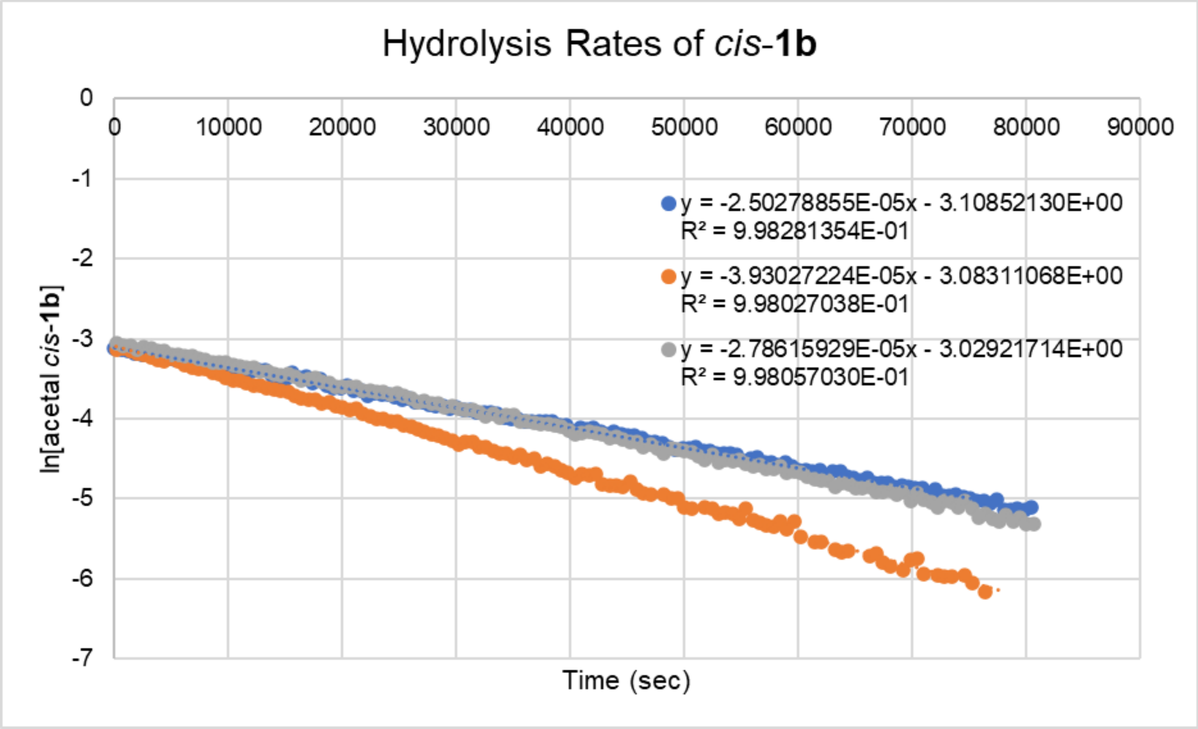

Graph **S7**. Rate of hydrolysis of acetal *trans*-**1c** plotted as ln [acetal] versus time (sec) over approximately three half-lives. Rate k = 1.818 × 10^{-4}.

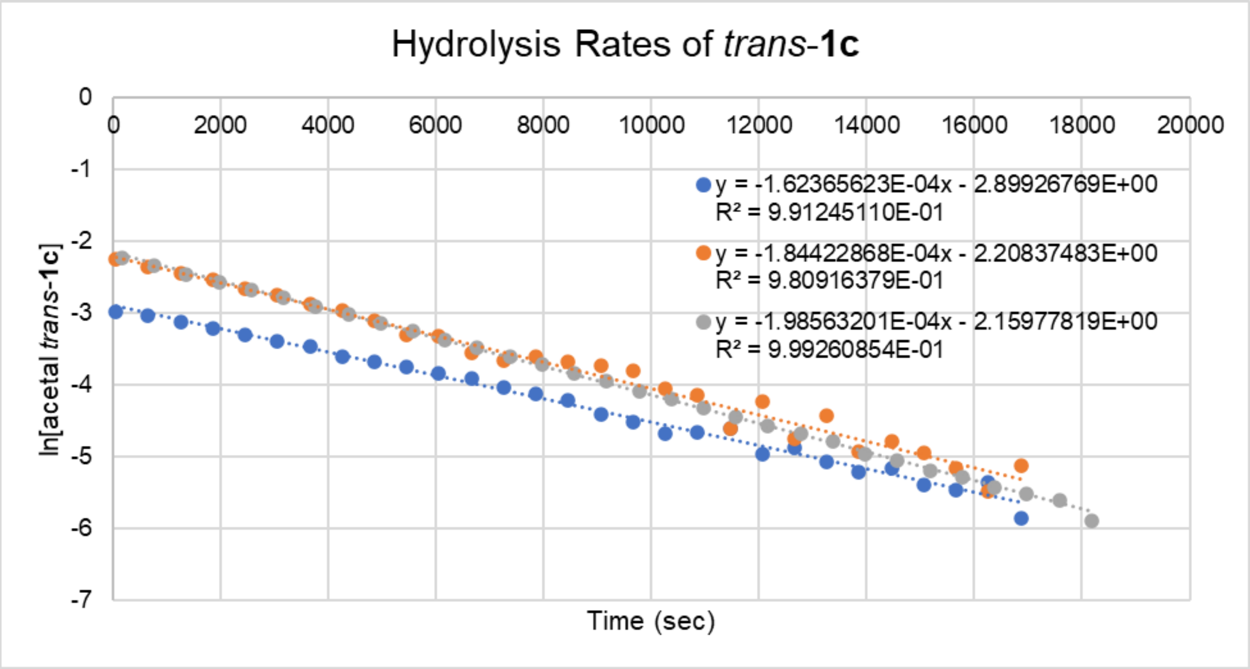

Graph **S8**. Rate of hydrolysis of acetal *cis*-**1c** plotted as ln [acetal] versus time (sec) over approximately four half-lives. Rate k = 4.461 × 10^{-5}.

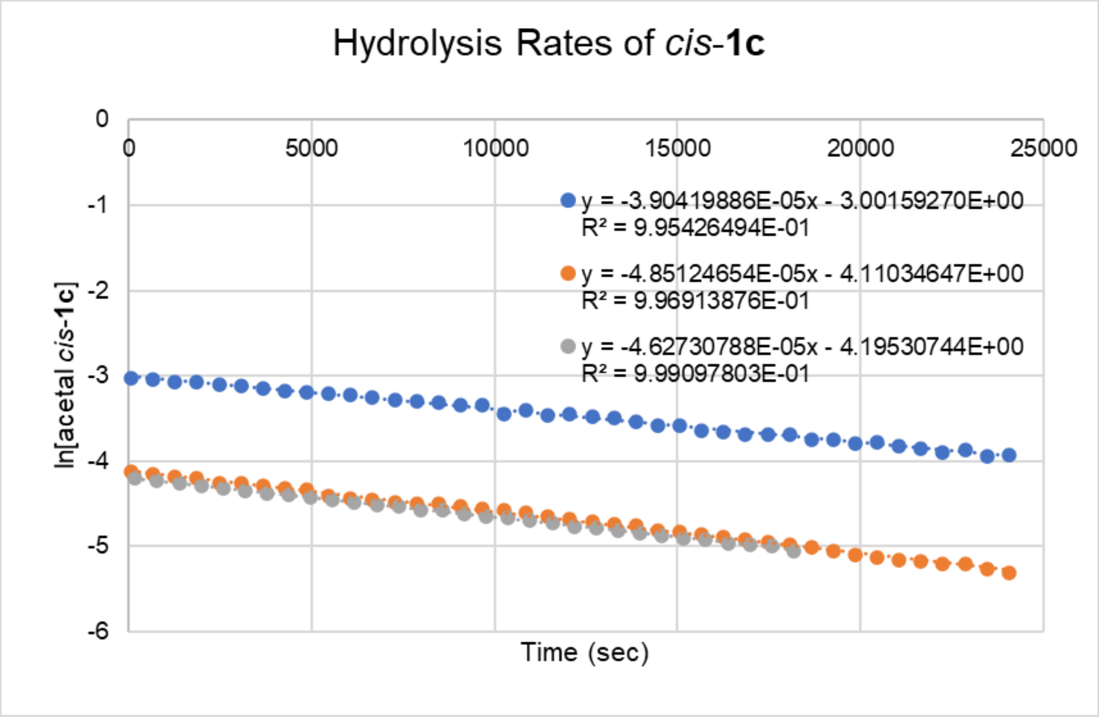

VII. Stereochemical Correlations and Proofs

A. Assignment of Relative Stereochemical Configurations by ^{1}H NMR Coupling Constants

a. ^{1}H NMR Coupling Constants of α-Halogenated Pyrans

Peaks were assigned in the ^{1}H NMR spectra by ^{1}H NMR chemical shifts and ^{1}H NMR coupling constants. The stereochemical configurations of the products were determined using ^{1}H NMR coupling constants because the coupling constants between protons on six-membered rings are well-documented.[12] Protons will either adopt an axial or equatorial orientation on the ring (or a mixture of these two conformers), and the coupling between adjacent protons (the coupling constant, *J*) will help to elucidate those orientations. The relative orientation between protons on the six-membered ring are represented by the coupling constant, and they are as follows: axial–axial, 8 to 13 Hz; axial–equatorial, 2 to 6 Hz; equatorial–equatorial, 2 to 9 Hz.[12] A-values of substituents on six-membered rings provide insight on the positioning of the allyl group on the ring, which is expected to be equatorial in alkenes **2a**, **2b**, **2c**, **2d**, **12**, and **14**.[13]

Anomeric interactions of $n_O \rightarrow \sigma^*_{C–O}$ are responsible for the axial orientation of the C-1 alkoxy substituent on the α-haloacetals.[8] The coupling constants (*J*) of the anomeric proton on these compounds are less defined because they are highly influenced by the alkoxy substituent at C-1. A more electron-withdrawing substituent at C-1 will have a greater axial preference due to a stronger anomeric interaction.[8, 14] As a result, the coupling constant of the anomeric proton on both the 1,2-*trans* isomer and the 1,2-*cis* isomer decreases as the preference for the axial orientation of the C-1 alkoxy group increases (i.e., there is a greater abundance of the conformer with an equatorial–equatorial interaction for the anomeric proton in the 1,2-*trans* isomer, and there is a greater abundance of the conformer with an equatorial–axial interaction for the anomeric proton in the 1,2-*cis* isomer).[14a] Because the coupling constant of the anomeric proton is not always consistent, other coupling constants in the six-membered ring were used to determine the relative stereochemistry of the α-haloacetals. The *J*-value coupling constant of the anomeric proton, however, was used as a relative comparison between the two anomers. In all cases for the α-chloroacetals and α-bromoacetals, it was observed that the coupling constant of the 1,2-*trans* isomer was larger than that of the 1,2-*cis* isomer.

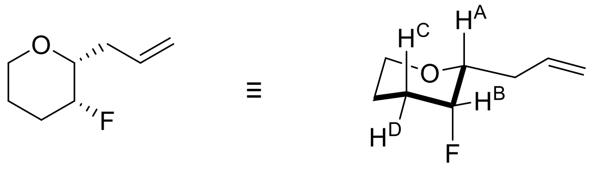

Alkene *cis*-2a (1,2-*cis*, major diastereomer):

^{1}H NMR (400 MHz, $CDCl_3$) δ 4.50 (H^B: dddd, J = 47.8 (eq^B–F), 2.7 (eq^B–ax^A), 2.7 (eq^B–ax^C), 0.7 (eq^B–eq^D), 1H)

Note: The splitting pattern of H^B indicates that H^B is equatorial (dddd, J = 47.8, 2.7, 2.7, 0.7 Hz), which places the fluorine atom in an axial orientation. The structure is 1,2-*cis* because the larger allyl group should adopt an equatorial orientation, as noted in X-ray crystallographic structures of similar compounds.[15]

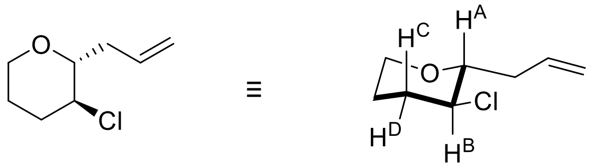

Alkene *trans*-2b (1,2-*trans*, major diastereomer):

^{1}H NMR (400 MHz, $CDCl_3$) δ 3.64 (H^B: ddd, J = 10.8 (ax^B–ax^A), 10.1 (ax^B–ax^C), 4.7 (ax^B–eq^D), 1H)

Note: The splitting pattern of H^B indicates that H^B is axial (ddd, J = 10.8, 10.1, 4.7 Hz), which places the chlorine atom in an equatorial orientation. The splitting pattern of H^B also indicates that H^A is axial, which places the allyl group in an equatorial orientation. The structure is 1,2-*trans* diequatorial because the larger allyl group should adopt an equatorial orientation, as noted in X-ray crystallographic structures of similar compounds.[15]

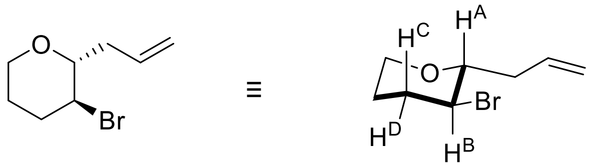

Alkene *trans*-2c (1,2-*trans*, major diastereomer):

^{1}H NMR (400 MHz, $CDCl_3$) δ 3.80 (H^B: ddd, J = 11.8 (ax^B–ax^A), 10.0 (ax^B–ax^C), 4.5 (ax^B–eq^D), 1H)

Note: The splitting pattern of H^B indicates that H^B is axial (ddd, J = 11.8, 10.0, 4.5 Hz), which places the bromine atom in an equatorial orientation. The splitting pattern of H^B also indicates that H^A is axial, which places the allyl group in an equatorial orientation. The structure is 1,2-*trans* diequatorial because the larger allyl group should adopt an equatorial orientation, as noted in X-ray crystallographic structures of similar compounds.[15]

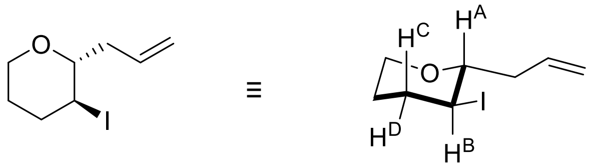

Alkene *trans*-2d (1,2-*trans*, major diastereomer):

^{1}H NMR (400 MHz, $CDCl_3$) δ 3.94 (H^B: ddd, J = 12.1 (ax^B–ax^A), 10.3 (ax^B–ax^C), 4.4 (ax^B–eq^D), 1H)

Note: The splitting pattern of H^B indicates that H^B is axial (ddd, *J* = 12.1, 10.3, 4.4 Hz), which places the iodine atom in an equatorial orientation. The splitting pattern of H^B also indicates that H^A is axial, which places the allyl group in an equatorial orientation. The structure is 1,2-*trans* diequatorial because the larger allyl group should adopt an equatorial orientation, as noted in X-ray crystallographic structures of similar compounds.[15]

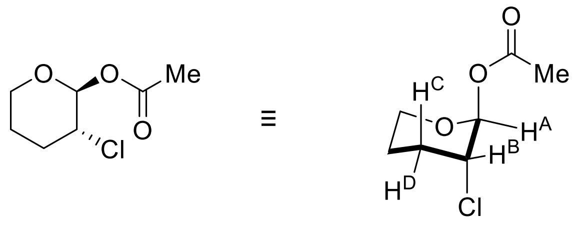

Acetal *trans*-1b (1,2-*trans*, major diastereomer):

^{1}H NMR (400 MHz, $CDCl_3$) δ 5.78 (H^A: d, *J* = 5.0 (eq^A–eq^B), 1H)

Note: The splitting pattern of H^A indicates that H^A is equatorial (d, *J* = 5.0 Hz), which places the acetate group in an axial orientation. This configuration of an acetate group at C-1 in an axial orientation is consistent with the anomeric effect.[5, 8] The *J*-value coupling constant of H^A in acetal *trans*-**1b** is similar to that of the 1,2-*trans* isomer of the α-chloro hemiacetal.[5]

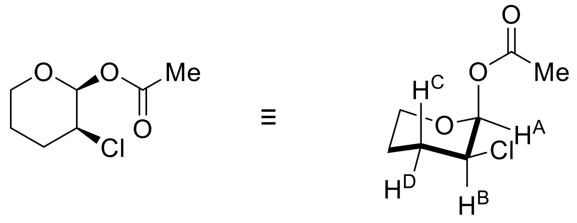

Acetal *cis*-1b (1,2-*cis*, minor diastereomer):

^{1}H NMR (400 MHz, $CDCl_3$) δ 6.09 (H^A: d, *J* = 2.9 (eq^A–ax^B), 1H)

Note: The splitting pattern of H^A indicates that H^A is equatorial (d, *J* = 2.9 Hz), which places the acetate group in an axial orientation. This configuration of an acetate group at C-1 in an axial orientation is consistent with the anomeric effect.[5, 8] The *J*-value coupling constant of H^A in acetal *cis*-**1b** is similar to that of the 1,2-*cis* isomer of the α-chloro hemiacetal.[5]

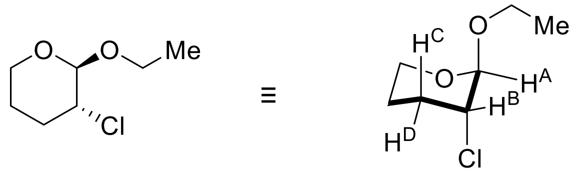

Acetal *trans*-7a (1,2-*trans*, major diastereomer):

^{1}H NMR (400 MHz, $CDCl_3$) δ 4.52 (H^A: d, *J* = 4.3 (eq^A–eq^B), 1H)

Note: The splitting pattern of H^A indicates that H^A is equatorial (d, *J* = 4.9 Hz), which places the alkoxy group in an axial orientation. This configuration of an alkoxy group at C-1 in an axial orientation is consistent with the anomeric effect.[5, 8] When compared to the *J*-value coupling constants of the 1,2-*cis* isomer of acetal *cis*-**7a**, it can be concluded that the chlorine atom in acetal *trans*-**7a** is in an axial orientation. The *J*-value coupling constant of H^A in acetal *trans*-**7a** is similar to that of all the other 1,2-*trans* α-chloroacetals.

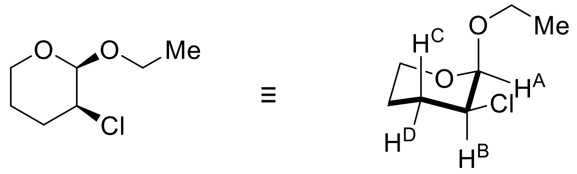

Acetal *cis*-7a (1,2-*cis*, minor diastereomer):

^{1}H NMR (400 MHz, $CDCl_3$) δ 4.73 (H^A: d, *J* = 2.6 (eq^A–ax^B), 1H); 3.99 (H^B: ddd, *J* = 11.3 (ax^B–ax^C), 4.4 (ax^B–eq^D), 2.9 (eq^A–ax^B), 1H)

Note: The splitting pattern of H^B indicates that H^B is axial (ddd, *J* = 11.3, 4.4, 2.9 Hz), which places the chlorine atom in an equatorial orientation. The splitting pattern of H^A and H^B also indicates that H^A is equatorial (d, *J* = 2.9 Hz), which places the alkoxy group in an axial orientation. This configuration of an alkoxy group at C-1 in an axial orientation is consistent with the anomeric effect.[5, 8]

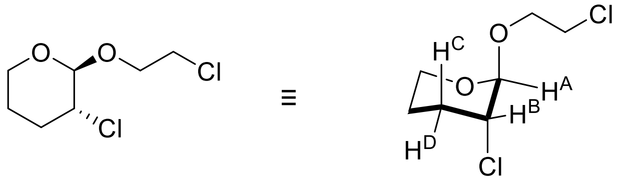

Acetal *trans*-7b (1,2-*trans*, major diastereomer):

^{1}H NMR (400 MHz, $CDCl_3$) δ 4.61 (H^A: d, *J* = 3.9 (eq^A–eq^B), 1H)

Note: The splitting pattern of H^A indicates that H^A is equatorial (d, *J* = 3.9 Hz), which places the alkoxy group in an axial orientation. This configuration of an alkoxy group at C-1 in an axial orientation is consistent with the anomeric effect.[5, 8] When compared to the *J*-value coupling constants of the 1,2-*cis* isomer of acetal *cis*-**7b**, it can be concluded that the chlorine atom in acetal *trans*-**7b** is in an axial orientation. The *J*-value coupling constant of H^A in acetal *trans*-**7b** is similar to that of all the other 1,2-*trans* α-chloroacetals.

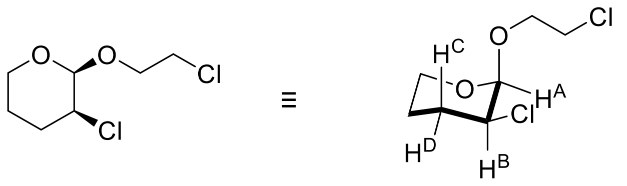

Acetal *cis*-7b (1,2-*cis*, minor diastereomer):

^{1}H NMR (400 MHz, CDCl$_3$) δ 4.77 (H^A: d, *J* = 2.8 (ax^B–eq^A), 1H)

Note: The splitting pattern of H^A indicates that H^A is equatorial (d, *J* = 2.8 Hz), which places the alkoxy group in an axial orientation. This configuration of an alkoxy group at C-1 in an axial orientation is consistent with the anomeric effect.[5, 8]. The *J*-value coupling constant of H^A in acetal *cis*-**7b** is similar to that of the other 1,2-*cis* α-chloroacetals.

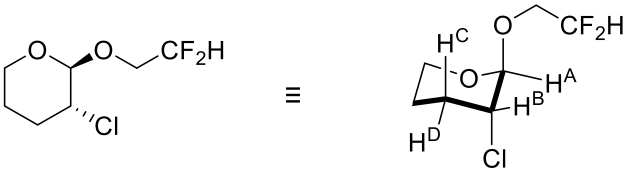

Acetal *trans*-7c (1,2-*trans*, major diastereomer):

^{1}H NMR (400 MHz, CDCl$_3$) δ 4.62 (H^A: d, *J* = 3.8 (eq^A–eq^B), 1H)

Note: The splitting pattern of H^A indicates that H^A is equatorial (d, *J* = 3.8 Hz), which places the alkoxy group in an axial orientation. This configuration of an alkoxy group at C-1 in an axial orientation is consistent with the anomeric effect.[5, 8] When compared to the *J*-value coupling constants of the 1,2-*cis* isomer of acetal *cis*-**7c**, it can be concluded that the chlorine atom in acetal *trans*-**7c** is in an axial orientation. The *J*-value coupling constant of H^A in acetal *trans*-**7c** is similar to that of all the other 1,2-*trans* α-chloroacetals.

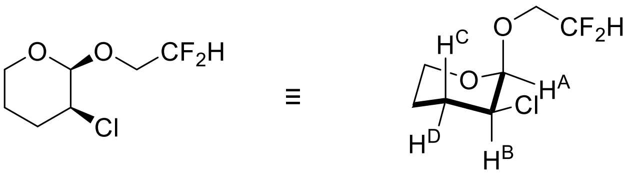

Acetal *cis*-7c (1,2-*cis*, minor diastereomer):

^{1}H NMR (400 MHz, CDCl$_3$) δ 4.78 (H^A: d, *J* = 2.8 (eq^A–ax^B), 1H); 3.99 (H^B: ddd, *J* = 11.6 (ax^B–ax^C), 4.5 (ax^B–eq^D), 3.0 (eq^A–ax^B), 1H)

Note: The splitting pattern of H^B indicates that H^B is axial (ddd, *J* = 11.6, 4.5, 3.0 Hz), which places the chlorine atom in an equatorial orientation. The splitting pattern of H^A and H^B also indicates that H^A is equatorial (d, *J* = 2.8 Hz), which places the alkoxy group in an axial orientation. This configuration of an alkoxy group at C-1 in an axial orientation is consistent with the anomeric effect.[5, 8]

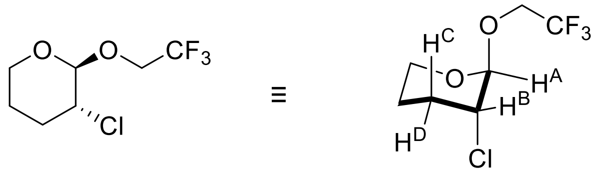

Acetal *trans*-7d (1,2-*trans*, major diastereomer):

^{1}H NMR (400 MHz, $CDCl_3$) δ 4.71 (H^A: d, J = 3.2 (eq^A–eq^B), 1H)

Note: The splitting pattern of H^A indicates that H^A is equatorial (d, J = 3.2 Hz), which places the alkoxy group in an axial orientation. This configuration of an alkoxy group at C-1 in an axial orientation is consistent with the anomeric effect.[5, 8] When compared to the J-value coupling constants of the 1,2-*cis* isomer of acetal *cis*-**7d**, it can be concluded that the chlorine atom in acetal *trans*-**7d** is in an axial orientation. The J-value coupling constant of H^A in acetal *trans*-**7d** is similar to that of all the other 1,2-*trans* α-chloroacetals.

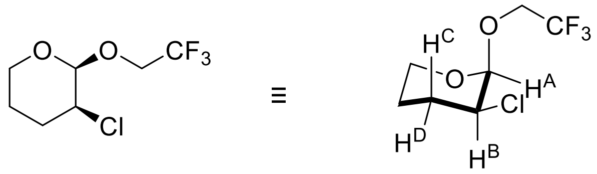

Acetal *cis*-7d (1,2-*cis*, minor diastereomer):

^{1}H NMR (400 MHz, $CDCl_3$) δ 4.83 (H^A: d, J = 2.5 (eq^A–ax^B))

Note: The splitting pattern of H^A indicates that H^A is equatorial (d, J = 2.5 Hz), which places the alkoxy group in an axial orientation. This configuration of an alkoxy group at C-1 in an axial orientation is consistent with the anomeric effect.[5, 8]. The J-value coupling constant of H^A in acetal *cis*-**7d** is similar to that of the other 1,2-*cis* α-chloroacetals.

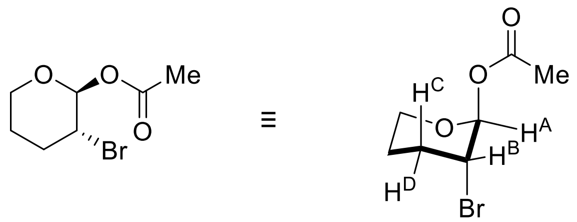

Acetal *trans*-1c (1,2-*trans*, major diastereomer):

^{1}H NMR (400 MHz, $CDCl_3$) δ 5.86 (H^A: d, J = 5.3 (eq^A–eq^B), 1H)

Note: The splitting pattern of H^A indicates that H^A is equatorial (d, J = 5.3 Hz), which places the acetate group in an axial orientation. This configuration of an acetate group at C-1 in an axial orientation is consistent with the anomeric effect.[5, 8] The J-value coupling constant of H^A in acetal *trans*-**1c** is similar to that of the 1,2-*trans* isomer of the α-bromo hemiacetal.[5]

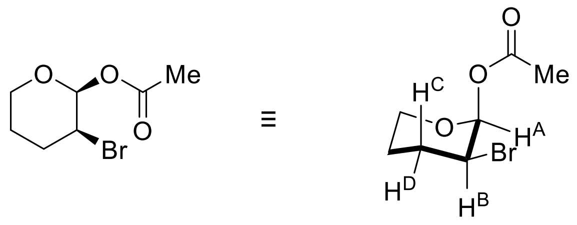

Acetal *cis*-1c (1,2-*cis*, minor diastereomer):

^{1}H NMR (400 MHz, $CDCl_3$) δ 6.08 (H^A: d, J = 2.8 (eq^A–ax^B), 1H)

Note: The splitting pattern of H^A indicates that H^A is equatorial (d, J = 2.8 Hz), which places the acetate group in an axial orientation. This configuration of an acetate group at C-1 in an axial orientation is consistent with the anomeric effect.[5, 8] The J-value coupling constant of H^A in acetal *cis*-**1c** is similar to that of the 1,2-*cis* isomer of the α-bromo hemiacetal.[5]

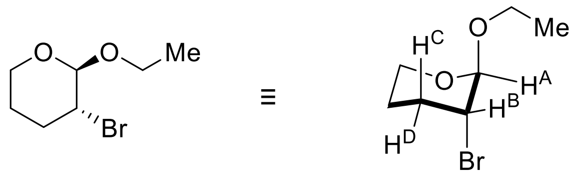

Acetal *trans*-8a (1,2-*trans*, major diastereomer):

^{1}H NMR (400 MHz, $CDCl_3$) δ 4.59 (H^A: d, J = 4.8 (eq^A–eq^B), 1H)

Note: The splitting pattern of H^A indicates that H^A is equatorial (d, J = 4.8 Hz), which places the alkoxy group in an axial orientation. This configuration of an alkoxy group at C-1 in an axial orientation is consistent with the anomeric effect.[5, 8] When compared to the J-value coupling constants of the 1,2-*cis* isomer of acetal *cis*-**8a**, it can be concluded that the bromine atom in acetal *trans*-**8a** is in an axial orientation. The J-value coupling constant of H^A in acetal *trans*-**8a** is similar to that of all the other 1,2-*trans* α-bromoacetals.

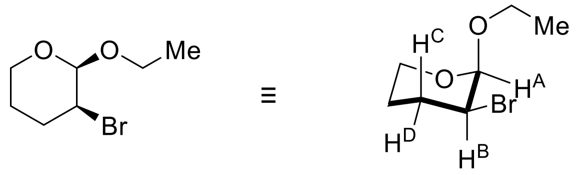

Acetal *cis*-8a (1,2-*cis*, minor diastereomer):

^{1}H NMR (400 MHz, $CDCl_3$) δ 4.71 (H^A: d, J = 2.7 (eq^A–ax^B), 1H); 4.10 (H^B: ddd, J = 11.5 (ax^B–ax^C), 4.3 (ax^B–eq^D), 2.8 (eq^A–ax^B), 1H)

Note: The splitting pattern of H^B indicates that H^B is axial (ddd, J = 11.5, 4.3, 2.8 Hz), which places the bromine atom in an equatorial orientation. The splitting pattern of H^A and H^B also indicates that H^A is equatorial (d, J = 2.7 Hz), which places the alkoxy group in an axial orientation. This configuration of an alkoxy group at C-1 in an axial orientation is consistent with the anomeric effect.[5, 8]

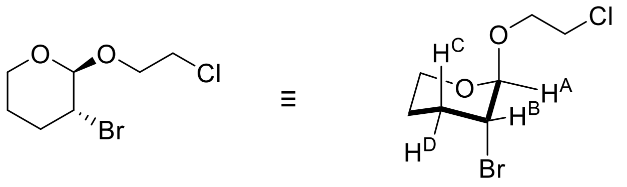

Acetal *trans*-8b (1,2-*trans*, major diastereomer):

^{1}H NMR (400 MHz, CDCl$_3$) δ 4.68 (H^A: d, *J* = 4.3 (eq^A–eq^B), 1H)

Note: The splitting pattern of H^A indicates that H^A is equatorial (d, *J* = 4.3 Hz), which places the alkoxy group in an axial orientation. This configuration of an alkoxy group at C-1 in an axial orientation is consistent with the anomeric effect.[5, 8] When compared to the *J*-value coupling constants of the 1,2-*cis* isomer of acetal *cis*-**8b**, it can be concluded that the bromine atom in acetal *trans*-**8b** is in an axial orientation. The *J*-value coupling constant of H^A in acetal *trans*-**8b** is similar to that of all the other 1,2-*trans* α-bromoacetals.

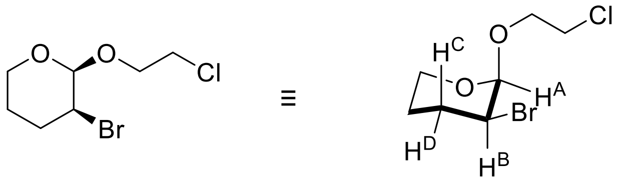

Acetal *cis*-8b (1,2-*cis*, minor diastereomer):

^{1}H NMR (400 MHz, CDCl$_3$) δ 4.77 (H^A: d, *J* = 2.7 (eq^A–ax^B), 1H); 4.10 (H^B: ddd, *J* = 11.7 ax^B–ax^C), 4.4 (ax^B–eq^D), 2.9 (eq^A–ax^B), 1H)

Note: The splitting pattern of H^B indicates that H^B is axial (ddd, *J* = 11.7, 4.5, 2.9 Hz), which places the bromine atom in an equatorial orientation. The splitting pattern of H^A and H^B also indicates that H^A is equatorial (d, *J* = 2.7 Hz), which places the alkoxy group in an axial orientation. This configuration of an alkoxy group at C-1 in an axial orientation is consistent with the anomeric effect.[5, 8]

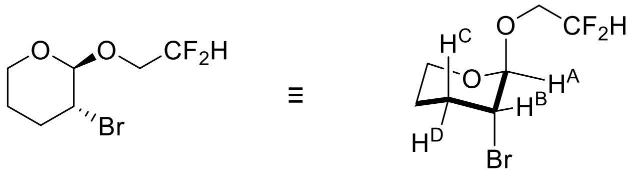

Acetal *trans*-8c (1,2-*trans*, major diastereomer):

^{1}H NMR (400 MHz, CDCl$_3$) δ 4.69 (H^A: d, *J* = 4.3 (eq^A–eq^B), 1H)

Note: The splitting pattern of H^A indicates that H^A is equatorial (d, *J* = 4.3 Hz), which places the alkoxy group in an axial orientation. This configuration of an alkoxy group at C-1 in an axial orientation is

consistent with the anomeric effect.[5, 8] The *J*-value coupling constant of H^A in acetal *trans*-**8c** is similar to that of all the other 1,2-*trans* α-bromoacetals.

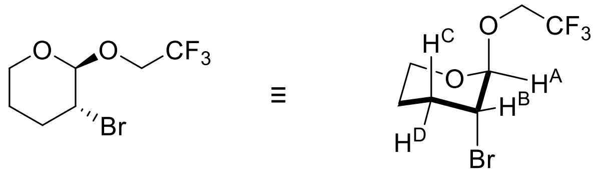

Acetal 8d (1,2-*trans*, major diastereomer):

^{1}H NMR (400 MHz, $CDCl_3$) δ 4.79 (H^A: d, *J* = 3.4 (eq^A–eq^B), 1H)

Note: The splitting pattern of H^A indicates that H^A is equatorial (d, *J* = 3.4 Hz), which places the alkoxy group in an axial orientation. This configuration of an alkoxy group at C-1 in an axial orientation is consistent with the anomeric effect.[5, 8] The *J*-value coupling constant of H^A in acetal **8d** is similar to that of the 1,2-*trans* α-chloroacetal *trans*-**7d**.

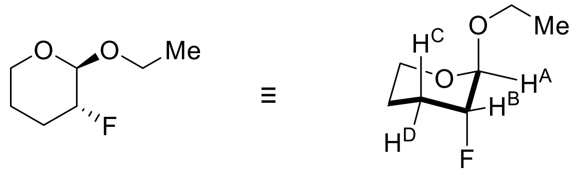

Acetal *trans*-10a (1,2-*trans*, major diastereomer):

^{1}H NMR (400 MHz, $CDCl_3$) δ 4.66 (H^A: dd, *J* = 6.8 (eq^A–F), 3.2 (eq^A– eq^B), 1H)

Note: The splitting pattern of H^A indicates that H^A is equatorial (*J* = 3.2 Hz), which places the alkoxy group in an axial orientation. This configuration of an alkoxy group at C-1 in an axial orientation is consistent with the anomeric effect.[5, 8] When compared to the *J*-value coupling constants of the 1,2-*cis* isomer of acetal *cis*-**10a**, it can be concluded that the fluorine atom in acetal *trans*-**10a** is in an axial orientation. The *J*-value coupling constant of H^A in acetal *trans*-**10a** is similar to that of all the other 1,2-*trans* α-fluoroacetals. The $^3J_{H–F}$ coupling constant of H^A (*J* = 6.8 Hz) indicates that the fluorine atom is axial.[16] The $^3J_{H–F}$ coupling constant of H^A in acetal *trans*-**10a** is similar to that of all the other 1,2-*trans* α-fluoroacetals.

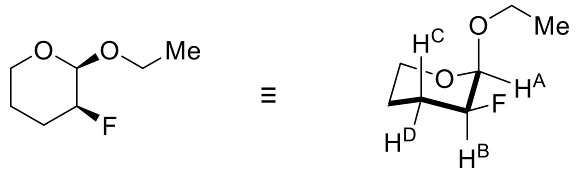

Acetal *cis*-10a (1,2-*cis*, minor diastereomer):

^{1}H NMR (400 MHz, $CDCl_3$) δ 4.81 (H^A: t, *J* = 3.2 (eq^A–F), (eq^A–ax^B), 1H); 4.53 (H^B: dddd, *J* = 48.2 (ax^B–F), 10.8 (ax^B–ax^C), 4.7 (ax^B–eq^D), 3.2 (eq^A–ax^B), 1H)

Note: The splitting pattern of H^B^ indicates that H^B^ is axial (dddd, *J* = 48.2, 10.8, 4.7, 3.2 Hz), which places the fluorine atom in an equatorial orientation. The splitting pattern of H^A and H^B also indicates that H^A is equatorial (*J* = 3.2 Hz), which places the alkoxy group in an axial orientation. This configuration of an alkoxy group at C-1 in an axial orientation is consistent with the anomeric effect.[5, 8] The *J*-value coupling constant of H^A in acetal *cis*-**10a** is similar to that of all the other 1,2-*cis* α-fluoroacetals. The $^3J_{H–F}$ coupling constant of H^A (*J* = 3.2 Hz) indicates that the fluorine atom is equatorial.[16] The $^3J_{H–F}$ coupling constant of H^A in acetal *cis*-**10a** is similar to that of all the other 1,2-*cis* α-fluoroacetals.

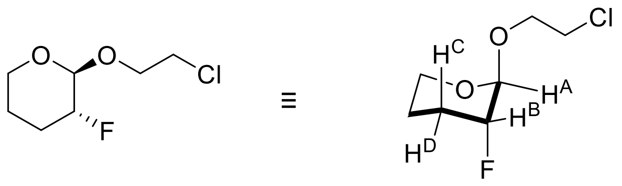

Acetal *trans*-10b (1,2-*trans*, major diastereomer):

^{1}H NMR (400 MHz, $CDCl_3$) δ 4.73 (H^A: dd, *J* = 6.9 (eq^A– F), 2.7 (eq^A– eq^B), 1H)

Note: The splitting pattern of H^A indicates that H^A is equatorial (*J* = 2.7 Hz), which places the alkoxy group in an axial orientation. This configuration of an alkoxy group at C-1 in an axial orientation is consistent with the anomeric effect.[5, 8] When compared to the *J*-value coupling constants of the 1,2-*cis* isomer of acetal *cis*-**10b**, it can be concluded that the fluorine atom in acetal *trans*-**10b** is in an axial orientation. The *J*-value coupling constant of H^A in acetal *trans*-**10b** is similar to that of all the other 1,2-*trans* α-fluoroacetals. The $^3J_{H–F}$ coupling constant of H^A (*J* = 6.9 Hz) indicates that the fluorine atom is axial.[16] The $^3J_{H–F}$ coupling constant of H^A in acetal *trans*-**10b** is similar to that of all the other 1,2-*trans* α-fluoroacetals.

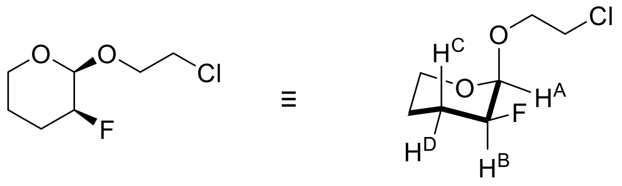

Acetal *cis*-10b (1,2-*cis*, minor diastereomer):

^{1}H NMR (400 MHz, $CDCl_3$) δ 4.86 (H^A: t, *J* = 3.0 (eq^A–F), (eq^A–ax^B) 1H); 4.53 (H^B: dddd, *J* = 47.9 (ax^B–F), 10.8 (ax^B–ax^C), 4.7 (ax^B–eq^D), 3.2 (eq^A–ax^B), 1H)

Note: The splitting pattern of H^B indicates that H^B is axial (dddd, *J* = 47.9, 10.8, 4.7, 3.2 Hz), which places the fluorine atom in an equatorial orientation. The splitting pattern of H^A and H^B also indicates that H^A is equatorial (*J* = 3.0 Hz), which places the alkoxy group in an axial orientation. This configuration of an alkoxy group at C-1 in an axial orientation is consistent with the anomeric effect.[5, 8]

The *J*-value coupling constant of H^A in acetal *cis*-**10b** is similar to that of all the other 1,2-*cis* α-fluoroacetals. The $^3J_{H–F}$ coupling constant of H^A (J = 3.0 Hz) indicates that the fluorine atom is equatorial.[16] The $^3J_{H–F}$ coupling constant of H^A in acetal *cis*-**10b** is similar to that of all the other 1,2-*cis* α-fluoroacetals.

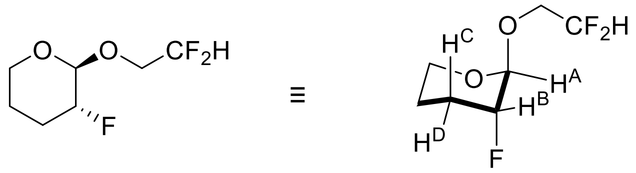

Acetal *trans*-10c (1,2-*trans*, major diastereomer):

^{1}H NMR (400 MHz, $CDCl_3$) δ 4.73 (H^A: dd, J = 6.9 (eq^A–F), 2.7 (eq^A–eq^B), 1H)

Note: The splitting pattern of H^A indicates that H^A is equatorial (J = 2.7 Hz), which places the alkoxy group in an axial orientation. This configuration of an alkoxy group at C-1 in an axial orientation is consistent with the anomeric effect.[5, 8] When compared to the *J*-value coupling constants of the 1,2-*cis* isomer of acetal *cis*-**10c**, it can be concluded that the fluorine atom in acetal *trans*-**10c** is in an axial orientation. The *J*-value coupling constant of H^A in acetal *trans*-**10c** is similar to that of all the other 1,2-*trans* α-fluoroacetals. The $^3J_{H–F}$ coupling constant of H^A (J = 6.9 Hz) indicates that the fluorine atom is axial.[16] The $^3J_{H–F}$ coupling constant of H^A in acetal *trans*-**10c** is similar to that of all the other 1,2-*trans* α-fluoroacetals.

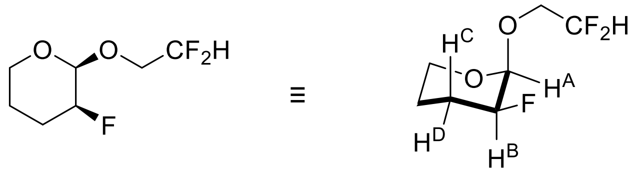

Acetal *cis*-10c (1,2-*cis*, minor diastereomer):

^{1}H NMR (400 MHz, $CDCl_3$) δ 4.86 (H^A: t, J = 2.9 (eq^A–F), (eq^A–ax^B), 1H); 4.54 (H^B: dddd, J = 47.7 (ax^B–F), 10.9 (ax^B–ax^C), 4.8 (ax^B–eq^D), 3.2 (eq^A–ax^B), 1H)

Note: The splitting pattern of H^B indicates that H^B is axial (dddd, J = 47.7, 10.9, 4.8, 3.2 Hz), which places the fluorine atom in an equatorial orientation. The splitting pattern of H^A and H^B also indicates that H^A is equatorial (J = 2.9 Hz), which places the alkoxy group in an axial orientation. This configuration of an alkoxy group at C-1 in an axial orientation is consistent with the anomeric effect.[5, 8] The *J*-value coupling constant of H^A in acetal *cis*-**10c** is similar to that of all the other 1,2-*cis* α-fluoroacetals. The $^3J_{H–F}$ coupling constant of H^A (J = 2.9 Hz) indicates that the fluorine atom is equatorial.[16] The $^3J_{H–F}$ coupling constant of H^A in acetal *cis*-**10c** is similar to that of all the other 1,2-*cis* α-fluoroacetals.

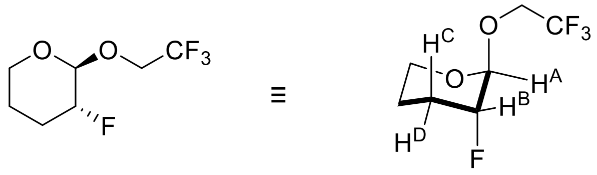

Acetal *trans*-10d (1,2-*trans*, major diastereomer):

^{1}H NMR (400 MHz, $CDCl_3$) δ 4.81 (H^A: dd, J = 7.0 (eq^A–F), 2.1 (eq^A–eq^B), 1H)

Note: The splitting pattern of H^A indicates that H^A is equatorial (J = 2.1 Hz), which places the alkoxy group in an axial orientation. This configuration of an alkoxy group at C-1 in an axial orientation is consistent with the anomeric effect.[5, 8] When compared to the *J*-value coupling constants of the 1,2-*cis* isomer of acetal *cis*-**10d**, it can be concluded that the fluorine atom in acetal *trans*-**10d** is in an axial orientation. The *J*-value coupling constant of H^A in acetal *trans*-**10d** is similar to that of all the other 1,2-*trans* α-fluoroacetals. The $^3J_{H–F}$ coupling constant of H^A (J = 7.0 Hz) indicates that the fluorine atom is axial.[16] The $^3J_{H–F}$ coupling constant of H^A in acetal *trans*-**10d** is similar to that of all the other 1,2-*trans* α-fluoroacetals.

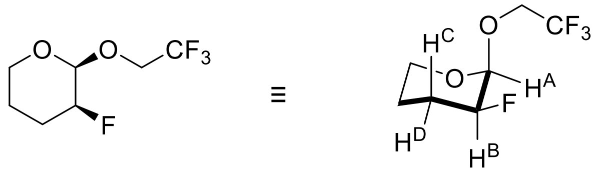

Acetal *cis*-10d (1,2-*cis*, minor diastereomer):

^{1}H NMR (400 MHz, $CDCl_3$) δ 4.91 (H^A: t, J = 2.7 (eq^A–F), (eq^A–ax^B), 1H); 4.75 (H^B: dddd, J = 47.8 (ax^B–F), 11.0 (ax^B–ax^C), 4.8 (ax^B–eq^D), 3.1 (eq^A–ax^B), 1H)

Note: The splitting pattern of H^B indicates that H^B is axial (dddd, J = 47.8, 11.0, 4.8, 3.1 Hz), which places the fluorine atom in an equatorial orientation. The splitting pattern of H^A and H^B also indicates that H^A is equatorial (J = 2.7 Hz), which places the alkoxy group in an axial orientation. This configuration of an alkoxy group at C-1 in an axial orientation is consistent with the anomeric effect.[5, 8] The *J*-value coupling constant of H^A in acetal *cis*-**10d** is similar to that of all the other 1,2-*cis* α-fluoroacetals. The $^3J_{H–F}$ coupling constant of H^A (J = 2.7 Hz) indicates that the fluorine atom is equatorial.[16] The $^3J_{H–F}$ coupling constant of H^A in acetal *cis*-**10d** is similar to that of all the other 1,2-*cis* α-fluoroacetals.

b. ^{1}H NMR Coupling Constants of α-Halogenated Furans

The relative stereochemical configurations of furans **19a–d** and **20a–d** were assigned by comparison of the spectroscopic data to that of furans **19e** and **20e**, whose structures were determined by X-ray crystallographic analysis. For all conformations of the halogenated furans, the alkoxy substituent at C-1

should be in an axial orientation because of the strong anomeric interaction between the lone pairs on the endocyclic oxygen atom and the exocyclic carbon–oxygen anti-bonding orbital ($n_O \rightarrow \sigma^*_{C-OR}$).[8] The anomeric proton of the 1,2-*trans* isomers of acetal **19e** and acetal **20e** had a very small *J*-value coupling constant, which is represented in the ^{1}H NMR spectra as a singlet (s).[8] The similar splitting pattern of the anomeric proton on acetals *trans*-**19a–d** and **trans**-**20a–d** indicate that the relative stereochemical configuration of the major diastereomer positions the halogen atom and the alkoxy group in a 1,2-transdiaxial relationship. Consequently, the larger coupling constants (*J*) of acetals *cis*-**19a–d** and *cis*-**20a–d** are consistent with an axial–equatorial interaction of protons,[17] which is represented in the ^{1}H NMR spectra as a doublet (d). The relative stereochemical configuration of the minor diastereomer positions the halogen atom and the alkoxy group in a 1,2-*cis* relationship.

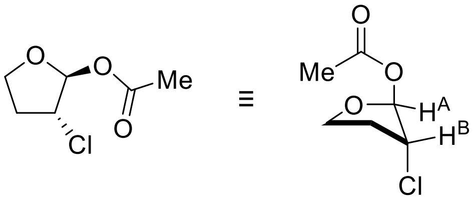

Acetal *trans*-11 (1,2-*trans*, major diastereomer):

^{1}H NMR (400 MHz, $CDCl_3$, diagnostic peaks) δ 6.24 (H^A: s, 1H)

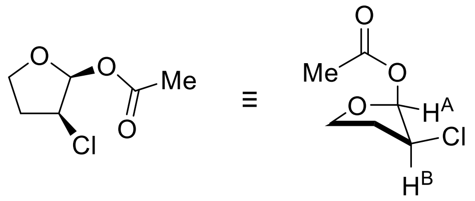

Acetal *cis*-11 (1,2-*cis*, minor diastereomer):

^{1}H NMR (400 MHz, $CDCl_3$, diagnostic peaks) δ 6.30 (H^A: d, J = 4.0 (eq^A–ax^B), 1H)

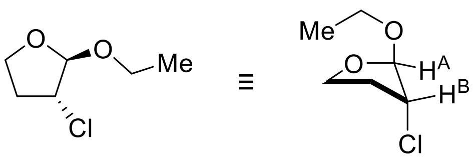

Acetal *trans*-19a (1,2-*trans*, major diastereomer):

^{1}H NMR (400 MHz, $CDCl_3$, diagnostic peaks) δ 5.10 (H^A: s, 1H)

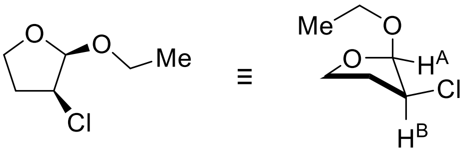

Acetal *cis*-19a (1,2-*cis*, minor diastereomer):

^{1}H NMR (400 MHz, $CDCl_3$, diagnostic peaks) δ 4.99 (H^A: d, *J* = 4.1 (eq^A–ax^B), 1H)

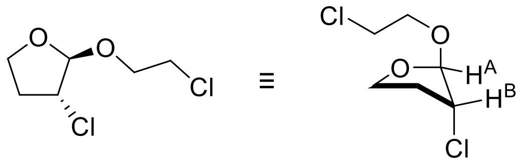

Acetal *trans*-19b (1,2-*trans*, major diastereomer):

^{1}H NMR (400 MHz, $CDCl_3$, diagnostic peaks) δ 5.15 (H^A: s, 1H)

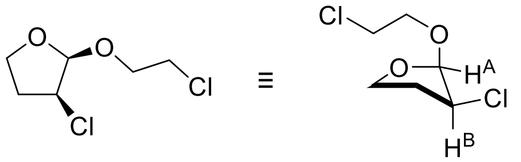

Acetal *cis*-19b (1,2-*cis*, minor diastereomer):

^{1}H NMR (400 MHz, $CDCl_3$, diagnostic peaks) δ 5.02 (H^A: d, *J* = 4.0 (eq^A–ax^B), 1H)

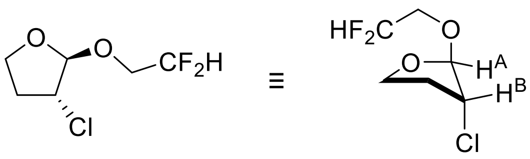

Acetal *trans*-19c (1,2-*trans*, major diastereomer):

^{1}H NMR (400 MHz, $CDCl_3$, diagnostic peaks) δ 5.15 (H^A: s, 1H)

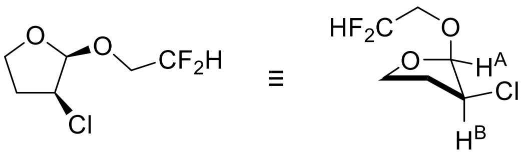

Acetal *cis*-19c (1,2-*cis*, minor diastereomer):

^{1}H NMR (400 MHz, $CDCl_3$, diagnostic peaks) δ 5.02 (H^A: d, *J* = 4.0 (eq^A–ax^B), 1H)

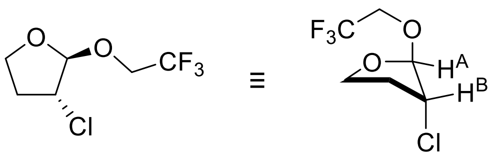

Acetal *trans*-19d (1,2-*trans*, major diastereomer):

^{1}H NMR (400 MHz, $CDCl_3$, diagnostic peaks) δ 5.19 (H^A: s, 1H)

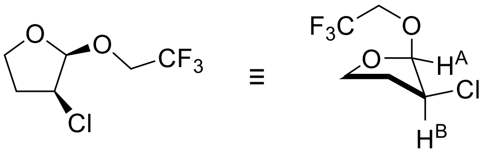

Acetal *cis*-19d (1,2-*cis*, minor diastereomer):

^{1}H NMR (400 MHz, $CDCl_3$, diagnostic peaks) δ 5.05 (H^A: d, *J* = 3.9 (eq^A–ax^B), 1H)

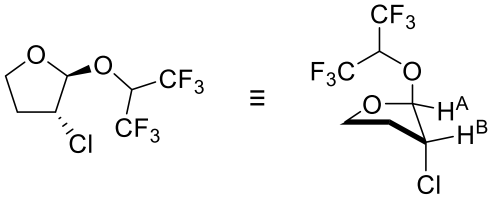

Acetal 20e (1,2-*trans*, major diastereomer):

^{1}H NMR (400 MHz, $CDCl_3$, diagnostic peaks) δ 5.35 (H^A: s, 1H)

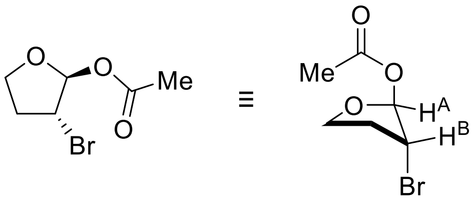

Acetal *trans*-1c (1,2-*trans*, major diastereomer):

^{1}H NMR (400 MHz, $CDCl_3$, diagnostic peaks) δ 6.35 (H^A: s, 1H)

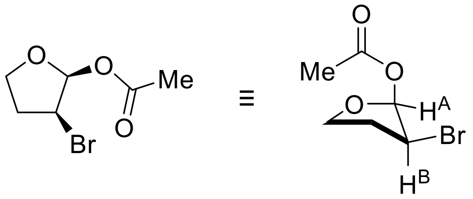

Acetal *cis*-1c (1,2-*cis*, minor diastereomer):

^{1}H NMR (400 MHz, $CDCl_3$, diagnostic peaks) δ 6.30 (H^A: d, *J* = 4.0 (eq^A–ax^B), 1H)

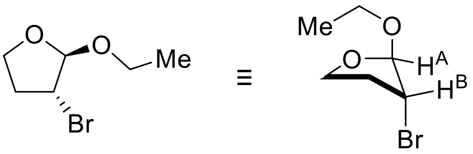

Acetal *trans*-20a (1,2-*trans*, major diastereomer):

^{1}H NMR (400 MHz, $CDCl_3$, diagnostic peaks) δ 5.23 (H^A: s, 1H)

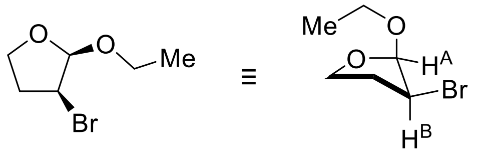

Acetal *cis*-20a (1,2-*cis*, minor diastereomer):

^{1}H NMR (400 MHz, $CDCl_3$, diagnostic peaks) δ 4.97 (H^A: d, *J* = 4.0 (eq^A–ax^B), 1H)

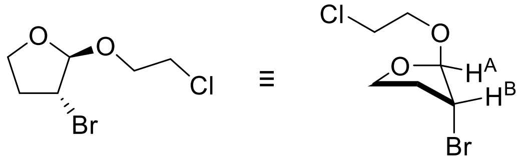

Acetal *trans*-20b (1,2-*trans*, major diastereomer):

^{1}H NMR (400 MHz, $CDCl_3$, diagnostic peaks) δ 5.27 (H^A: s, 1H)

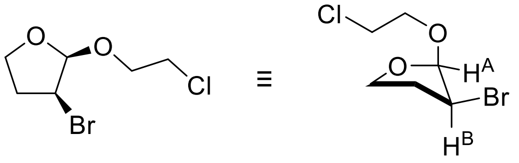

Acetal *cis*-20b (1,2-*cis*, minor diastereomer):

^{1}H NMR (400 MHz, $CDCl_3$, diagnostic peaks) δ 5.02 (H^A: d, *J* = 3.9 (eq^A–ax^B), 1H)

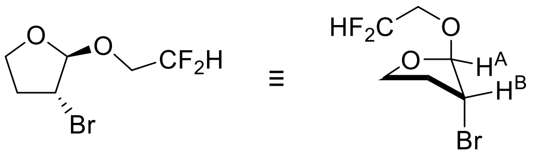

Acetal 20c (1,2-*trans*, major diastereomer):

^{1}H NMR (400 MHz, $CDCl_3$, diagnostic peaks) δ 5.27 (H^A: s, 1H)

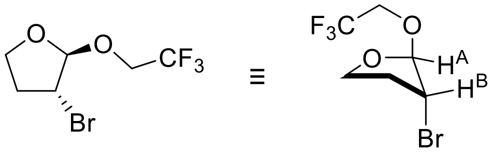

Acetal 20d (1,2-*trans*, major diastereomer):

^{1}H NMR (400 MHz, $CDCl_3$, diagnostic peaks) δ 5.31 (H^A: s, 1H)

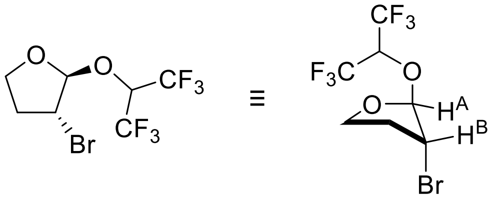

Acetal 20e (1,2-*trans*, major diastereomer):

^{1}H NMR (400 MHz, $CDCl_3$, diagnostic peaks) δ 5.46 (H^A: s, 1H)

B. Assignment of the Relative Stereochemical Configuration of Alkenes 12 and 14 by Derivatization to Furan S6

The stereochemical configurations of alkenes **12** and **14** were assigned by derivatization to furan **S6**. Cyclization of alcohols **S5** and **S8** resulted from an intramolecular displacement of the halogen atom by the hydroxyl group upon treatment with a base. An *anti*-orientation between the alcohol and the halogen atom is necessary for this transformation to occur because the formation of the *cis*-fused bicyclic ring is energetically favored, whereas the *trans*-fused ring is not.[18] This conformation is also energetically favored in the *cis*-fused ring in the hydrocarbon bicyclo[3.3.0]octane.[19] This orientation is only found in the major diastereomer of each addition reaction, signifying that there is 1,2-*trans* relationship between the halogen atom and the alkene in alkenes *trans*-**12** and **14**.

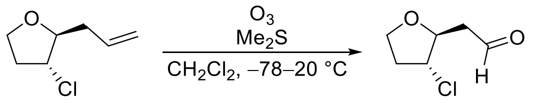

trans-**12** **S4**

2-((2*R,3*S**)-3-Chlorotetrahydrofuran-2-yl)acetaldehyde (S4)**.

To a cooled (–78 °C) solution of alkene *trans*-**12** (0.305 g, 2.08 mmol, dr >99:1) in CH_2Cl_2 (20 mL) was passed through O_3 (g) until the solution was saturated, as indicated by a change to a light blue color (approximately 10 min). The reaction mixture was stirred at –78 °C for an additional 30 min and then warmed to 20 °C. N_2 (g) was passed through the solution and dimethyl sulfide (1 mL, 14 mmol) was added. After 16 h, the reaction mixture was then concentrated *in vacuo* and aldehyde **S4** was directly used without further purification.

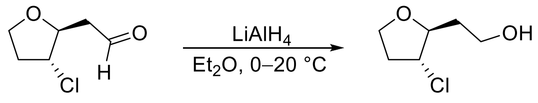

S4 **S5**

2-((2*R,3*S**)-3-Chlorotetrahydrofuran-2-yl)ethan-1-ol (S5)**.

To a cooled (0 °C) suspension of lithium aluminum hydride (0.174 g, 4.58 mmol) in Et_2O (20 mL) was added a solution of aldehyde **S4** (2.08 mmol) in Et_2O (10 mL) dropwise over 2 min by cannula. After 1 h, the reaction was warmed to 20 °C, and after an additional 3 h, MeOH (1 mL) and H_2O (20 mL) were then carefully added. NaOH (15 mL, 1.0 M in H_2O) was added, and the layers were separated. The organic layer was washed with brine (1 × 20 mL), dried over Na_2SO_4, filtered, and concentrated *in vacuo*. ^{1}H NMR and $^{13}C\{^1H\}$ NMR spectroscopic analysis of the unpurified reaction mixture revealed

that alcohol **S5** was formed as a single diastereomer (dr >99:1). Alcohol **S5** was recovered as a colorless oil (0.068 g, 22%):

^{1}H NMR (400 MHz, $CDCl_3$) δ 4.06–3.95 (m, 4H), 3.83–3.79 (m, 2H), 2.50–2.41 (m, 1H), 2.38–2.35 (m, 1H), 2.18–2.11 (m, 1H), 2.00–1.93 (m, 1H), 1.76–1.68 (m, 1H);

^{13}C{^{1}H} NMR (100 MHz, $CDCl_3$) δ 86.2 (CH), 66.6 (CH_2), 60.8 (CH_2), 59.9 (CH), 35.8 (CH_2), 35.1 (CH_2);

IR (ATR) 3384, 2948, 1052, 1014, 813, 709 cm^{-1};

HRMS (ESI) *m / z* calcd for $C_6H_{11}O_2$ ((M + H) – HCl)$^+$ 115.0754, found 115.0752.

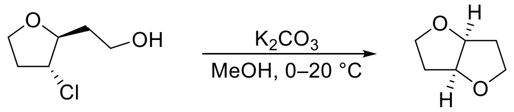

S5 S6

(3a*R,6a*R**)-Hexahydrofuro[3,2-*b*]furan (S6)**.

To a solution of alcohol **S5** (0.060 g, 0.40 mmol, dr >99:1) in MeOH (4 mL) was added K_2CO_3 (0.220 g, 1.50 mmol) at 0 °C. After 1 h, the reaction mixture was warmed to 20 °C and was stirred for an additional 48 h. H_2O (5 mL) was then added, the layers were separated, and the aqueous layer was extracted with CH_2Cl_2 (3 × 5 mL). The combined organic layers were dried over Na_2SO_4, filtered, and concentrated *in vacuo*. ^{1}H NMR and ^{13}C{^{1}H} NMR spectroscopic analysis revealed alcohol **S5** (dr >99:1) and furan **S6** in a ratio of 77:23. The spectroscopic data of furan **S6** are consistent with those of the same furan prepared from alcohol **S8** and K_2CO_3.

Note: Based on the formation of furan **S6**, the relative stereochemical configuration of major diastereomer *trans*-**12** positions the chlorine atom and the alkene in a 1,2-*trans* relationship.

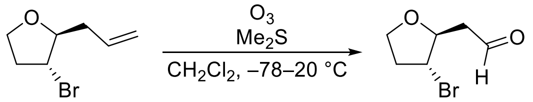

14 S7

2-((2*R,3*S**)-3-Bromotetrahydrofuran-2-yl)acetaldehyde (S7).**

To a cooled (–78 °C) solution of alkene **14** (0.775 g, 4.06 mmol, dr >99:1) in CH_2Cl_2 (40 mL) was passed through O_3 (g) until the solution was saturated, as indicated by a change to a light blue color (approximately 10 min). The reaction mixture was stirred at –78 °C for an additional 30 min and then warmed to 20 °C. N_2 (g) was passed through the solution and dimethyl sulfide (2 mL, 27 mmol) was

added. After 16 h, the reaction mixture was then concentrated *in vacuo* and aldehyde **S7** was directly used without further purification.

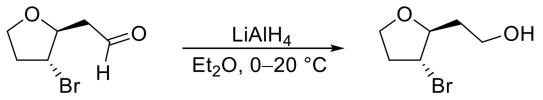

2-((2*R,3*S**)-3-Bromotetrahydrofuran-2-yl)ethan-1-ol (S8)**.

To a cooled (0 °C) suspension of lithium aluminum hydride (0.270 g, 7.11 mmol) in Et_2O (35 mL) was added a solution of aldehyde **S7** (0.681 g, 3.53 mmol, dr >99:1) in Et_2O (18 mL) dropwise over 2 min by cannula. After 1 h, the reaction was warmed to 20 °C, and after an additional 3 h, MeOH (1 mL) and H_2O (20 mL) were then carefully added. NaOH (15 mL, 1.0 M in H_2O) was added, and the layers were separated. The organic layer was washed with brine (1 × 20 mL), dried over Na_2SO_4, filtered, and concentrated *in vacuo*. ^{1}H NMR and ^{13}C{^{1}H} NMR spectroscopic analysis of the unpurified reaction mixture revealed that alcohol **S8** was recovered as a single diastereomer (dr >99:1). Purification by flash chromatography (25:75 EtOAc:hexanes) afforded alcohol **S8** as a colorless oil (0.225 g, 42%):

^{1}H NMR (400 MHz, $CDCl_3$) δ 4.16–4.11 (m, 1H), 4.04–3.94 (m, 3H), 3.83–3.79 (m, 2H), 2.58–2.49 (m, 1H), 2.38–2.36 (m, 1H), 2.28–2.21 (m, 1H), 2.03–1.96 (m, 1H), 1.75–1.66 (m, 1H);

^{13}C{^{1}H} NMR (100 MHz, $CDCl_3$) δ 86.5 (CH), 66.8 (CH_2), 60.8 (CH_2), 49.1 (CH), 36.2 (CH_2), 35.0 (CH_2);

IR (ATR) 3382, 2880, 1182, 1049, 1013, 808 cm^{-1};

HRMS (ESI) *m / z* calcd for $C_6H_{10}BrO$ ((M + H) – H_2O)$^+$ 176.9910, found 176.9902.

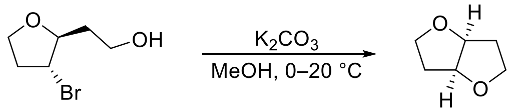

(3a*R,6a*R**)-Hexahydrofuro[3,2-*b*]furan (S6)**.

To a solution of alcohol **S8** (0.200 g, 1.03 mmol, dr >99:1) in MeOH (10 mL) was added K_2CO_3 (0.588 g, 4.25 mmol) at 0 °C. After 1 h, the reaction mixture was warmed to 20 °C and was stirred for an additional 48 h. H_2O (10 mL) was then added, the layers were separated, and the aqueous layer was extracted with CH_2Cl_2 (3 × 10 mL). The combined organic layers were dried over Na_2SO_4, filtered, and concentrated *in vacuo*. ^{1}H NMR and ^{13}C{^{1}H} NMR spectroscopic analysis revealed the presence of only furan **S6**. Furan **S6** was formed as a light yellow oil (0.082 g, 69%):

^{1}H NMR (400 MHz, $CDCl_3$) δ 4.61–4.57 (m, 2H), 3.88–3.80 (m, 4H), 2.04–2.00 (m, 4H);

$^{13}C\{^1H\}$ NMR (100 MHz, $CDCl_3$) δ 83.5 (CH), 68.0 (CH_2), 34.6 (CH_2);

IR (ATR) 2944, 2868, 1102, 1050, 1031, 732 cm^{-1}.

Note: Based on the formation of furan **S6**, the relative stereochemical configuration of major diastereomer **14** positions the bromine atom and the alkene in a 1,2-*trans* relationship.

VIII. Computational Methods

A. Generation of the Computational Energy Landscapes

The workflow for the generation of computational energy landscapes (CEL) for both pyranyl[20] and furanyl[21] cations was adapted for computations in ORCA5.03.[22] Initial geometries were constructed by a constrained relaxed potential energy surface scan using the AM1 semi-empirical method and the VerySlowConv keyword. All bond distances of directly connected atoms (*i.e.* C1–H1 O5–C1, C2–X2, *etc.*) were constrained. For furanyl cations the C1–C2–C3–C4 and the C5–O5–C1–C2 dihedral angles were scanned from –40 to 40 degrees with 10 degree steps, generating 81 unique structures. For the pyranyl cations the C1–C2–C3–C4, C3–C4–C5–O5 and the C5–O5–C1–C2 dihedral angles were scanned from –60 to 60 degrees with 15 degree steps, generating 729 unique structures. The subsequent output geometries were then used as starting geometries for the CEL computations with only dihedral constrains on the C1–C2–C3–C4 and the C5–O5–C1–C2 (furanyl cations) or on the C1–C2–C3–C4, C3–C4–C5–O5 and the C5–O5–C1–C2 (pyranyl cations) dihedral angles. The CEL computations were then performed by re-optimization of the AM1 geometries, placing constrains on the mentioned dihedral angles, with DFT using the keywords TightSCF, DEFGRID and SlowConv at SMD(dichloromethane)-revDSD-PBEP86-D4-DKH-def2TZVPP//PCM(dichloromethane)-B3LYP-D3(BJ)-DKH-def2TZVP[23] and additional SARC[24] basis functions on iodine. The CEL maps were visualized using Origin 9.0.0[25][6] in identical fashion to Hansen *et al.*[20, 26]

B. Computational Investigations of Cyclic Halonium Cations

To investigate the possibility of cyclic halonium cations being involved as reactive intermediates in glycosylation reactions, their existence in solvent phase was investigated. Initial geometries of possible pyranyl halonium cations were constructed for the ^3E and E_3 conformations, while for pyranyl halonium cations the $^3H_4$, $^4H_3$, B_{25} and ^{25}B conformations were constructed. These initial fluoro-, chloro-, bromo- and iodonium structures were then optimized at PCM(dichloromethane)-B3LYP-D3(BJ)-(SARC)-DKH-def2TZVP. All cyclic halonium ions formed ring-opened oxocarbenium ions upon optimization. Thus, no local minima were identified for any of the studied cyclic halonium cations.

C. Benchmark of Conformational Energy Landscape Methods

The computational method of the conformational energy landscapes was evaluated by a single point energy benchmark, using a set of selected CEL geometries. The selected geometries included the lowest electronic energy ^{3}E, E_3 and flat geometries from each furanyl cation, while for the pyranyl cations the lowest electronic energy $^{3}H_4$ and $^{4}H_3$ were used. For this geometry set consisting of 20 cations reference '*golden-standard'* energies were computed at SMD(dichloromethane)-CCSD(T)-DKH-def2TZVPP. The basis set employed for the benchmark calculations was always (SARC[24])-DKH-def2TZVPP[23i] with the keywords DEFGRID3(BJ), TightSCF, SARC/J[23j] and AutoAux.[27] The following dispersion corrections by Grimme were evaluated when possible: D3(BJ)[23g] with Becke-Johnson dampening[23h] (here always denoted as D3(BJ)(BJ)) and D4.[23b] Implicit solvation in dichloromethane was included with the SMD model.[28] The following methods and functionals were evaluated (given here in no particular order): DLPNO-CCSD(T),[29] RI-MP2, DLPNO-MP2,[30] DSD-PBEP86,[31] DSD-PBEB95,[32] revDSD-PBEP86-D4,[23a] M06-2X,[33] B2PLYP, B2PLYP-D3(BJ), B2PLYP-D4,[34] mPW2PLYPb, mPW2PLYP-D4,[35] RSX-QIDH, RSX-0DH,[36] PBE0-QIDH,[37] B97M-V, B97M-V-D3(BJ), B97M-V-D4,[38] PBE0-DH,[39] PBE0, PBE0-D3(BJ), PBE0-D4,[40] ωB97M-V, ωb97M-V-D3(BJ), ωB97M-V-D4,[41] ωB97X-V, ωB97X-2,[42] ωB97X-D3(BJ), ωB97X-D4,[43] B3LYP, B3LYP-D3(BJ), B3LYP-D4,[23c-f] SCAN, SCAN-D3(BJ), SCAN-D4,[44] rSCAN, rSCAN-D3(BJ), rSCAN-D4,[45] r^2SCAN, r^2SCAN-D3(BJ), r^2SCAN-D4,[46] OLYP, OLYP-D3(BJ), OLYP-D4,[23d, 47] PW6B95, PW6B95-D3(BJ), PW6B95-D4.[48]

From this benchmark, we selected the revDSD-PBEP86-D4 (Supplementary Table S2) functional by the group of Martin as our method of single point energy correction for the CEL calculations. This method performed best of all DFT-based methods for our systems with a fraction of the cost of WFT-based methods. Based on these benchmark results we can offer some general advice concerning the choice of method, here we discuss the most striking results from this benchmark. First of all we wish to comment on the performance of DLPNO-CCSD(T) and DLPNO-MP2 methods, which, as expected, provide almost identical results to the classical non-local methods at a fraction of the cost (Supplementary Table S1). Furthermore, all screened functionals of hybrid quality and up performed reasonably well for relative energies (RMSD = < 0.5 kcal/mol). On the subject of dispersion, we noticed that the inclusion of dispersion generally decreased the accuracy of the computations (D4 performing better than D3(BJ)). In these particular systems the influence of dispersion is minimal and thus inclusion of it provides numerical noise, in contrast to for instance transition state geometries. The inclusion of dispersion, however, is generally advisable since the accuracy-decrease for systems with minimal dispersion is small, while the accuracy increase for systems with many dispersion interactions is major.[49] Thus, it is generally advisable to include dispersion.

a. **Supplementary Table S1.** Single point benchmark energies for CCSD(T), DLPNO-CCSD(T), RI-MP2 and DLPNO-MP2.

IX.	CCSD(T)	DLPNO-CCSD(T)	RI-MP2	DLPNO-MP2
RMSD-absolute[a]	n/a	3.48	54.33	54.62
RMSD-relative[b]	n/a	0.14	0.25	0.24
		Absolute Energies		
2-F-furanyl E_3	–207452.18	–207449.67	–207401.46	–207401.26
2-F-furanyl flat	–207450.96	–207448.42	–207400.12	–207399.93
2-F-furanyl ^3E	–207451.25	–207448.70	–207400.54	–207400.34
2-Cl-furanyl E_3	–434181.78	–434179.35	–434121.37	–434121.16
2-Cl-furanyl flat	–434181.23	–434178.75	–434120.74	–434120.54
2-Cl-furanyl ^3E	–434182.70	–434180.11	–434122.45	–434122.24
2-Br-furanyl E_3	–1779189.63	–1779185.67	–1779136.93	–1779136.58
2-Br-furanyl flat	–1779190.07	–1779186.06	–1779137.38	–1779137.03
2-Br-furanyl ^3E	–1779192.13	–1779187.95	–1779139.74	–1779139.38
2-I-furanyl E_3	–4606978.50	–4606974.56	–4606947.92	–4606947.56
2-I-furanyl flat	–4606979.36	–4606975.35	–4606948.85	–4606948.49
2-I-furanyl ^3E	–4606981.86	–4606977.72	–4606951.74	–4606951.38
2-F-pyranyl $^3H_4$	–232086.95	–232084.17	–232025.05	–232024.79
2-F-pyranyl $^4H_3$	–232089.29	–232086.49	–232027.53	–232027.27
2-Cl-pyranyl $^3H_4$	–458818.42	–458815.66	–458747.04	–458746.76
2-Cl-pyranyl $^4H_3$	–458818.72	–458816.02	–458747.34	–458747.08
2-Br-pyranyl $^3H_4$	–1803827.91	–1803823.48	–1803764.46	–1803764.04
2-Br-pyranyl $^4H_3$	–1803827.17	–1803822.97	–1803763.54	–1803763.13
2-I-pyranyl $^3H_4$	–4631617.34	–4631612.96	–4631576.31	–4631575.87
2-I-pyranyl $^4H_3$	–4631616.24	–4631611.88	–4631575.07	–4631574.62

All methods are with SMD(dichloromethane)-(SARC)-DKH-def2TZVPP and energies are reported in kcal/mol.[a] RMSD values were calculated by comparing the absolute single point energy of the method with energies computed at SMD(dichloromethane)-CCSD-(SARC)-DKH-def2TZVPP.[b] RMSD values were calculated by comparing the relative single point energy (within each cation separately) of the method with energies computed at SMD(dichloromethane)-CCSD(T)-DKH-def2TZVPP.

b. Supplementary Table S2. Single point benchmark energies for revDSD-PBEP86-D4, DSD-PBEP86, RSX-QIDH and DSD-PBEB95.

	revDSD-PBEP86-D4	DSD-PBEP86	RSX-QIDH	DSD-PBEB95
RMSD-absolute[a]	393.33	445.44	509.78	564.34
RMSD-relative[b]	0.07	0.12	0.22	0.13
		Absolute Energies		
2-F-furanyl E_3	–207491.81	–207515.61	–207471.29	–207528.66
2-F-furanyl flat	–207490.62	–207514.34	–207469.83	–207527.37
2-F-furanyl ^3E	–207490.85	–207514.56	–207469.95	–207527.60
2-Cl-furanyl E_3	–434314.05	–434344.09	–434322.41	–434381.94
2-Cl-furanyl flat	–434313.55	–434343.56	–434321.67	–434381.39
2-Cl-furanyl ^3E	–434314.95	–434345.03	–434323.10	–434382.89
2-Br-furanyl E_3	–1779591.04	–1779642.62	–1779694.70	–1779757.73
2-Br-furanyl flat	–1779591.53	–1779643.12	–1779695.15	–1779758.22
2-Br-furanyl ^3E	–1779593.55	–1779645.24	–1779697.33	–1779760.38
2-I-furanyl E_3	–4607636.49	–4607718.82	–4607849.34	–4607923.75
2-I-furanyl flat	–4607637.41	–4607719.76	–4607850.13	–4607924.67
2-I-furanyl ^3E	–4607639.80	–4607722.31	–4607852.82	–4607927.27
2-F-pyranyl $^3H_4$	–232132.33	–232158.93	–232110.56	–232171.38
2-F-pyranyl $^4H_3$	–232134.67	–232161.33	–232113.19	–232173.80
2-Cl-pyranyl $^3H_4$	–458956.46	–458989.34	–458963.49	–459026.56
2-Cl-pyranyl $^4H_3$	–458956.73	–458989.63	–458964.13	–459026.88
2-Br-pyranyl $^3H_4$	–1804235.12	–1804289.64	–1804337.77	–1804404.15
2-Br-pyranyl $^4H_3$	–1804234.23	–1804288.67	–1804337.05	–1804403.15
2-I-pyranyl $^3H_4$	–4632281.09	–4632366.44	–4632493.01	–4632570.77
2-I-pyranyl $^4H_3$	–4632279.86	–4632365.14	–4632491.80	–4632569.52

All methods are with SMD(dichloromethane)-(SARC)-DKH-def2TZVPP and energies are reported in kcal/mol.[a] RMSD values were calculated by comparing the absolute single point energy of the method with energies computed at SMD(dichloromethane)-CCSD(T)-(SARC)-DKH-def2TZVPP.[b] RMSD values were calculated by comparing the relative single point energy (within each cation separately) of the method with energies computed at SMD(dichloromethane)-CCSD(T)-(SARC)-DKH-def2TZVPP.

c. **Supplementary Table S3.** Single point benchmark energies for ωB97X-2, RSX-0DH, PBE0-QIDH and B97M-V.

X.	ωb97X-2	RSX-0DH	PBE0-QIDH	B97M-V
RMSD-absolute[a]	589.78	604.03	638.11	754.37
RMSD-relative[b]	0.04	0.30	0.18	0.40
		Absolute Energies		
2-F-furanyl E_3	–207410.46	–207436.80	–207583.84	–207807.18
2-F-furanyl flat	–207409.28	–207435.32	–207582.46	–207805.89
2-F-furanyl ^3E	–207409.60	–207435.33	–207582.56	–207806.14
2-Cl-furanyl E_3	–434143.56	–434313.04	–434449.81	–434696.96
2-Cl-furanyl flat	–434142.98	–434312.22	–434449.22	–434696.70
2-Cl-furanyl ^3E	–434144.42	–434313.50	–434450.65	–434698.45
2-Br-furanyl E_3	–1778838.99	–1779778.60	–1779843.81	–1780056.66
2-Br-furanyl flat	–1778839.43	–1779779.03	–1779844.33	–1780057.31
2-Br-furanyl ^3E	–1778841.50	–1779781.09	–1779846.47	–1780059.65
2-I-furanyl E_3	–4605860.43	–4608024.85	–4608014.98	–4607980.16
2-I-furanyl flat	–4605861.31	–4608025.58	–4608015.90	–4607981.24
2-I-furanyl ^3E	–4605863.79	–4608028.15	–4608018.49	–4607983.85
2-F-pyranyl $^3H_4$	–232033.33	–232071.72	–232240.18	–232498.63
2-F-pyranyl $^4H_3$	–232035.62	–232074.38	–232242.70	–232501.01
2-Cl-pyranyl $^3H_4$	–458768.16	–458949.54	–459108.09	–459390.68
2-Cl-pyranyl $^4H_3$	–458768.48	–458950.37	–459108.49	–459390.52
2-Br-pyranyl $^3H_4$	–1803465.28	–1804417.14	–1804504.00	–1804751.95
2-Br-pyranyl $^4H_3$	–1803464.49	–1804416.62	–1804503.00	–1804750.32
2-I-pyranyl $^3H_4$	–4630487.25	–4632663.92	–4632675.78	–4632675.74
2-I-pyranyl $^4H_3$	–4630486.10	–4632662.74	–4632674.45	–4632674.22

All methods are with SMD(dichloromethane)-(SARC)-DKH-def2TZVPP and energies are reported in kcal/mol.[a] RMSD values were calculated by comparing the absolute single point energy of the method with energies computed at SMD(dichloromethane)-CCSD(T)-(SARC)-DKH-def2TZVPP.[b] RMSD values were calculated by comparing the relative single point energy (within each cation separately) of the method with energies computed at SMD(dichloromethane)-CCSD(T)-(SARC)-DKH-def2TZVPP.

d. **Supplementary Table S4.** Single point benchmark energies for PBE0-DH, B2PLYP, B2PLYP-D4, B2PLYP-D3(BJ) and mPW2PLYPb.

XI.	PBE0-DH	B2PLYP	B2PLYP-D4	B2PLYP-D3(BJ)	mPW2PLYP
RMSD-absolute[a]	810.45	845.93	850.33	851.51	868.27
RMSD-relative[b]	0.21	0.17	0.21	0.20	0.16
			Absolute Energies		
2-F-furanyl E_3	–207615.60	–207689.66	–207692.88	–207693.78	–207688.79
2-F-furanyl flat	–207614.24	–207688.51	–207691.73	–207692.62	–207687.62
2-F-furanyl ^3E	–207614.22	–207688.64	–207691.89	–207692.78	–207687.73
2-Cl-furanyl E_3	–434518.13	–434587.85	–434591.86	–434593.05	–434595.15
2-Cl-furanyl flat	–434517.62	–434587.44	–434591.50	–434592.66	–434594.72
2-Cl-furanyl ^3E	–434518.93	–434588.74	–434592.89	–434594.06	–434596.01
2-Br-furanyl E_3	–1780022.00	–1780072.06	–1780076.42	–1780077.63	–1780095.47
2-Br-furanyl flat	–1780022.57	–1780072.61	–1780077.00	–1780078.20	–1780096.02
2-Br-furanyl ^3E	–1780024.59	–1780074.51	–1780078.99	–1780080.21	–1780097.92
2-I-furanyl E_3	–4608296.47	–4608316.53	–4608321.27	–4608322.64	–4608354.53
2-I-furanyl flat	–4608297.44	–4608317.54	–4608322.30	–4608323.68	–4608355.52
2-I-furanyl ^3E	–4608299.86	–4608319.77	–4608324.62	–4608326.06	–4608357.75
2-F-pyranyl $^3H_4$	–232276.88	–232355.48	–232360.20	–232361.35	–232355.01
2-F-pyranyl $^4H_3$	–232279.36	–232357.73	–232362.43	–232363.61	–232357.29
2-Cl-pyranyl $^3H_4$	–459181.32	–459255.47	–459261.18	–459262.60	–459263.14
2-Cl-pyranyl $^4H_3$	–459181.67	–459255.63	–459261.21	–459262.66	–459263.36
2-Br-pyranyl $^3H_4$	–1804687.05	–1804741.29	–1804747.39	–1804748.86	–1804765.09
2-Br-pyranyl $^4H_3$	–1804685.95	–1804740.25	–1804746.14	–1804747.66	–1804764.11
2-I-pyranyl $^3H_4$	–4632962.03	–4632986.18	–4632992.73	–4632994.40	–4633024.55
2-I-pyranyl $^4H_3$	–4632960.62	–4632984.79	–4632991.29	–4632992.92	–4633023.18

All methods are with SMD(dichloromethane)-(SARC)-DKH-def2TZVPP and energies are reported in kcal/mol.[a] RMSD values were calculated by comparing the absolute single point energy of the method with energies computed at SMD(dichloromethane)-CCSD(T)-(SARC)-DKH-def2TZVPP.[b] RMSD values were calculated by comparing the relative single point energy (within each cation separately) of the method with energies computed at SMD(dichloromethane)-CCSD(T)-(SARC)-DKH-def2TZVPP.

e. **Supplementary Table S5.** Single point benchmark energies for mPW2PLYP-D4, PBE0, PBE0-D4, PBE0-D3(BJ) and B97M-V-D4.

XII.	mPW2PLYP-D4	PBE0	PBE0-D4	PBE0-D3(BJ)	B97M-V-D4[b]
RMSD-absolute[a]	870.98	881.32	886.43	887.10	939.48
RMSD-relative[b]	0.17	0.28	0.35	0.32	0.37
			Absolute Energies		
2-F-furanyl E_3	–207690.82	–207615.88	–207619.72	–207620.49	–207928.25
2-F-furanyl flat	–207689.65	–207614.61	–207618.45	–207619.21	–207926.97
2-F-furanyl ^3E	–207689.78	–207614.52	–207618.40	–207619.15	–207927.16
2-Cl-furanyl E_3	–434597.66	–434529.05	–434533.86	–434534.74	–434839.35
2-Cl-furanyl flat	–434597.25	–434528.72	–434533.59	–434534.44	–434839.11
2-Cl-furanyl ^3E	–434598.58	–434530.00	–434534.96	–434535.80	–434840.78
2-Br-furanyl E_3	–1780098.17	–1780092.91	–1780098.15	–1780098.88	–1780246.65
2-Br-furanyl flat	–1780098.73	–1780093.57	–1780098.84	–1780099.56	–1780247.31
2-Br-furanyl ^3E	–1780100.67	–1780095.51	–1780100.89	–1780101.60	–1780249.56
2-I-furanyl E_3	–4608357.43	–4608422.81	–4608428.53	–4608429.22	–4608215.65
2-I-furanyl flat	–4608358.43	–4608423.90	–4608429.65	–4608430.33	–4608216.72
2-I-furanyl ^3E	–4608360.71	–4608426.16	–4608432.01	–4608432.72	–4608219.21
2-F-pyranyl $^3H_4$	–232358.00	–232275.42	–232281.07	–232282.04	–232640.86
2-F-pyranyl $^4H_3$	–232360.28	–232277.77	–232283.40	–232284.41	–232643.22
2-Cl-pyranyl $^3H_4$	–459266.73	–459190.55	–459197.41	–459198.46	–459554.27
2-Cl-pyranyl $^4H_3$	–459266.87	–459190.67	–459197.37	–459198.47	–459554.09
2-Br-pyranyl $^3H_4$	–1804768.89	–1804756.14	–1804763.47	–1804764.38	–1804963.11
2-Br-pyranyl $^4H_3$	–1804767.79	–1804754.76	–1804761.85	–1804762.86	–1804961.49
2-I-pyranyl $^3H_4$	–4633028.60	–4633086.44	–4633094.35	–4633095.23	–4632932.38
2-I-pyranyl $^4H_3$	–4633027.20	–4633084.91	–4633092.75	–4633093.62	–4632930.86

All methods are with SMD(dichloromethane)-(SARC)-DKH-def2TZVPP and energies are reported in kcal/mol[a] RMSD values were calculated by comparing the absolute single point energy of the method with energies computed at SMD(dichloromethane)-CCSD(T)-(SARC)-DKH-def2TZVPP.[b] RMSD values were calculated by comparing the relative single point energy (within each cation separately) of the method with energies computed at SMD(dichloromethane)-CCSD(T)-(SARC)-DKH-def2TZVPP.

f.Supplementary Table S6. Single point benchmark energies for B97M-V-D3(BJ), ωB97M-V, ωB97X-V, B3LYP, B3LYP-D4.

XIII.	B97M-V-D3(BJ)	ωB97M-V	ωB97X-V	B3LYP	B3LYP-D4
RMSD-absolute[a]	947.02	964.72	1034.89	1052.09	1062.17
RMSD-relative[b]	0.39	0.12	0.25	0.37	0.39
			Absolute Energies		
2-F-furanyl E_3	–207933.18	–207767.05	–207782.20	–207738.58	–207745.99
2-F-furanyl flat	–207931.84	–207765.84	–207781.01	–207737.52	–207744.93
2-F-furanyl ^3E	–207932.05	–207765.98	–207781.12	–207737.42	–207744.91
2-Cl-furanyl E_3	–434846.03	–434711.01	–434710.05	–434676.02	–434685.25
2-Cl-furanyl flat	–434845.76	–434710.45	–434709.40	–434675.81	–434685.14
2-Cl-furanyl ^3E	–434847.49	–434711.76	–434710.55	–434676.88	–434686.42
2-Br-furanyl E_3	–1780254.17	–1780243.99	–1780270.23	–1780296.29	–1780306.35
2-Br-furanyl flat	–1780254.81	–1780244.46	–1780270.66	–1780296.95	–1780307.06
2-Br-furanyl ^3E	–1780257.12	–1780246.41	–1780272.48	–1780298.59	–1780308.94
2-I-furanyl E_3	–4608224.35	–4608434.60	–4608586.58	–4608642.07	–4608653.03
2-I-furanyl flat	–4608225.40	–4608435.43	–4608587.35	–4608643.19	–4608654.19
2-I-furanyl ^3E	–4608228.05	–4608437.78	–4608589.57	–4608645.03	–4608656.29
2-F-pyranyl $^3H_4$	–232646.93	–232442.76	–232466.36	–232411.63	–232422.37
2-F-pyranyl $^4H_3$	–232649.40	–232445.04	–232468.65	–232413.75	–232424.47
2-Cl-pyranyl $^3H_4$	–459562.19	–459388.36	–459395.63	–459350.80	–459363.80
2-Cl-pyranyl $^4H_3$	–459562.10	–459388.71	–459396.17	–459350.81	–459363.50
2-Br-pyranyl $^3H_4$	–1804971.93	–1804923.03	–1804957.53	–1804972.51	–1804986.43
2-Br-pyranyl $^4H_3$	–1804970.41	–1804922.20	–1804956.96	–1804971.30	–1804984.73
2-I-pyranyl $^3H_4$	–4632942.47	–4633114.03	–4633274.24	–4633318.49	–4633333.47
2-I-pyranyl $^4H_3$	–4632940.90	–4633112.92	–4633273.19	–4633316.95	–4633331.78

All methods are with SMD(dichloromethane)-(SARC)-DKH-def2TZVPP and energies are reported in kcal/mol.[a] RMSD values were calculated by comparing the absolute single point energy of the method with energies computed at SMD(dichloromethane)-CCSD(T)-(SARC)-DKH-def2TZVPP.[b] RMSD values were calculated by comparing the relative single point energy (within each cation separately) of the method with energies computed at SMD(dichloromethane)-CCSD(T)-(SARC)-DKH-def2TZVPP.

g. **Supplementary Table S7.** Single point benchmark energies for B3LYP-D3(BJ), r^2SCAN, r^2SCAN-D4, r^2SCAN-D3(BJ) and ωb97M-V-D4.

XIV.	B3LYP-D3(BJ)	r^2SCAN	r^2SCAN-D4	r^2SCAN-D3(BJ)	ωb97M-V-D4
RMSD-absolute[a]	1064.04	1103.86	1105.41	1105.80	1144.01
RMSD-relative[b]	0.34	0.57	0.59	0.59	0.25
			Absolute Energies		
2-F-furanyl E_3	–207747.84	–207734.21	–207735.35	–207735.69	–207886.81
2-F-furanyl flat	–207746.74	–207732.93	–207734.07	–207734.41	–207885.62
2-F-furanyl ^3E	–207746.72	–207733.25	–207734.40	–207734.74	–207885.68
2-Cl-furanyl E_3	–434687.43	–434688.05	–434689.47	–434689.89	–434851.42
2-Cl-furanyl flat	–434687.25	–434687.89	–434689.32	–434689.74	–434850.85
2-Cl-furanyl ^3E	–434688.52	–434689.78	–434691.22	–434691.64	–434852.01
2-Br-furanyl E_3	–1780308.30	–1780348.71	–1780350.23	–1780350.63	–1780431.60
2-Br-furanyl flat	–1780308.99	–1780349.48	–1780351.00	–1780351.40	–1780432.05
2-Br-furanyl ^3E	–1780310.85	–1780351.96	–1780353.50	–1780353.90	–1780433.83
2-I-furanyl E_3	–4608654.97	–4608731.24	–4608732.88	–4608733.29	–4608667.30
2-I-furanyl flat	–4608656.13	–4608732.48	–4608734.12	–4608734.53	–4608668.10
2-I-furanyl ^3E	–4608658.29	–4608735.17	–4608736.82	–4608737.24	–4608670.22
2-F-pyranyl $^3H_4$	–232424.69	–232409.13	–232410.86	–232411.33	–232583.20
2-F-pyranyl $^4H_3$	–232426.87	–232411.48	–232413.20	–232413.68	–232585.46
2-Cl-pyranyl $^3H_4$	–459366.39	–459365.32	–459367.40	–459367.96	–459549.38
2-Cl-pyranyl $^4H_3$	–459366.21	–459365.05	–459367.09	–459367.65	–459549.79
2-Br-pyranyl $^3H_4$	–1804988.82	–1805027.62	–1805029.81	–1805030.34	–1805131.18
2-Br-pyranyl $^4H_3$	–1804987.34	–1805025.70	–1805027.84	–1805028.40	–1805130.52
2-I-pyranyl $^3H_4$	–4633335.90	–4633410.44	–4633412.77	–4633413.33	–4633367.18
2-I-pyranyl $^4H_3$	–4633334.18	–4633408.71	–4633411.03	–4633411.59	–4633366.15

All methods are with SMD(dichloromethane)-(SARC)-DKH-def2TZVPP and energies are reported in kcal/mol.[a] RMSD values were calculated by comparing the absolute single point energy of the method with energies computed at SMD(dichloromethane)-CCSD(T)-(SARC)-DKH-def2TZVPP.[b] RMSD values were calculated by comparing the relative single point energy (within each cation separately) of the method with energies computed at SMD(dichloromethane)-CCSD(T)-(SARC)-DKH-def2TZVPP.

h. **Supplementary Table S8.** Single point benchmark energies for ωB97M-V-D3(BJ), ωB97X-D4, ωB97X-D3(BJ), SCAN and SCAN-D4.

XV.	ωB97M-V-D3(BJ)	ωB97X-D4	ωB97X-D3(BJ)	SCAN	SCAN-D4
RMSD-absolute[a]	1146.89	1219.18	1220.33	1280.45	1281.33
RMSD-relative[b]	0.25	0.19	0.28	0.66	0.67
			Absolute Energies		
2-F-furanyl E_3	–207889.38	–207904.74	–207906.12	–207768.16	–207768.80
2-F-furanyl flat	–207888.14	–207903.57	–207904.90	–207766.88	–207767.53
2-F-furanyl ^3E	–207888.21	–207903.63	–207904.95	–207767.29	–207767.94
2-Cl-furanyl E_3	–434854.43	–434855.03	–434856.59	–434752.97	–434753.78
2-Cl-furanyl flat	–434853.81	–434854.44	–434855.93	–434752.84	–434753.66
2-Cl-furanyl ^3E	–434854.98	–434855.61	–434857.03	–434754.83	–434755.66
2-Br-furanyl E_3	–1780434.48	–1780463.73	–1780464.98	–1780506.12	–1780506.99
2-Br-furanyl flat	–1780434.91	–1780464.19	–1780465.40	–1780506.91	–1780507.78
2-Br-furanyl ^3E	–1780436.70	–1780466.07	–1780467.17	–1780509.51	–1780510.38
2-I-furanyl E_3	–4608670.17	–4608826.70	–4608827.83	–4609038.30	–4609039.24
2-I-furanyl flat	–4608670.97	–4608827.49	–4608828.58	–4609039.52	–4609040.45
2-I-furanyl ^3E	–4608673.16	–4608829.76	–4608830.84	–4609042.29	–4609043.23
2-F-pyranyl $^3H_4$	–232586.50	–232610.57	–232612.07	–232447.28	–232448.27
2-F-pyranyl $^4H_3$	–232588.84	–232612.85	–232614.43	–232449.61	–232450.60
2-Cl-pyranyl $^3H_4$	–459553.11	–459562.45	–459564.06	–459434.56	–459435.77
2-Cl-pyranyl $^4H_3$	–459553.60	–459562.89	–459564.65	–459434.17	–459435.36
2-Br-pyranyl $^3H_4$	–1805134.82	–1805172.91	–1805174.21	–1805189.36	–1805190.62
2-Br-pyranyl $^4H_3$	–1805134.28	–1805172.15	–1805173.74	–1805187.24	–1805188.49
2-I-pyranyl $^3H_4$	–4633370.86	–4633536.33	–4633537.51	–4633721.76	–4633723.11
2-I-pyranyl $^4H_3$	–4633369.80	–4633535.16	–4633536.36	–4633720.04	–4633721.38

All methods are with SMD(dichloromethane)-(SARC)-DKH-def2TZVPP and energies are reported in kcal/mol.[a] RMSD values were calculated by comparing the absolute single point energy of the method with energies computed at SMD(dichloromethane)-CCSD(T)-(SARC)-DKH-def2TZVPP.[a] RMSD values were calculated by comparing the relative single point energy (within each cation separately) of the method with energies computed at SMD(dichloromethane)-CCSD(T)-(SARC)-DKH-def2TZVPP.

i. **Supplementary Table S9.** Single point benchmark energies for SCAN-D3(BJ), M06-2X, rSCAN, rSCAN-D4 and rSCAN-D3(BJ).

XVI.	SCAN-D3(BJ)	M06-2X	rSCAN	rSCAN-D4	rSCAN-D3(BJ)
RMSD-absolute[a]	1282.02	1308.51	1416.08	1417.92	1418.36
RMSD-relative[b]	0.67	0.18	0.57	0.60	0.60
			Absolute Energies		
2-F-furanyl E_3	–207769.45	–207760.61	–207803.18	–207804.53	–207804.92
2-F-furanyl flat	–207768.17	–207759.32	–207801.90	–207803.25	–207803.63
2-F-furanyl ^3E	–207768.58	–207759.35	–207802.18	–207803.54	–207803.92
2-Cl-furanyl E_3	–434754.51	–434728.45	–434801.06	–434802.76	–434803.23
2-Cl-furanyl flat	–434754.39	–434727.98	–434800.91	–434802.62	–434803.09
2-Cl-furanyl ^3E	–434756.40	–434729.38	–434802.77	–434804.50	–434804.97
2-Br-furanyl E_3	–1780507.70	–1780458.41	–1780635.28	–1780637.11	–1780637.56
2-Br-furanyl flat	–1780508.49	–1780458.96	–1780636.06	–1780637.90	–1780638.35
2-Br-furanyl ^3E	–1780511.10	–1780461.08	–1780638.54	–1780640.40	–1780640.84
2-I-furanyl E_3	–4609039.94	–4609144.26	–4609265.90	–4609267.88	–4609268.35
2-I-furanyl flat	–4609041.16	–4609145.22	–4609267.15	–4609269.14	–4609269.61
2-I-furanyl ^3E	–4609043.95	–4609147.76	–4609269.84	–4609271.85	–4609272.34
2-F-pyranyl $^3H_4$	–232449.19	–232439.40	–232487.57	–232489.62	–232490.14
2-F-pyranyl $^4H_3$	–232451.51	–232441.85	–232489.94	–232491.97	–232492.51
2-Cl-pyranyl $^3H_4$	–459436.78	–459409.15	–459487.81	–459490.29	–459490.91
2-Cl-pyranyl $^4H_3$	–459436.37	–459409.47	–459487.55	–459489.98	–459490.61
2-Br-pyranyl $^3H_4$	–1805191.61	–1805140.89	–1805323.68	–1805326.31	–1805326.91
2-Br-pyranyl $^4H_3$	–1805189.48	–1805139.83	–1805321.77	–1805324.33	–1805324.95
2-I-pyranyl $^3H_4$	–4633724.09	–4633827.24	–4633954.62	–4633957.43	–4633958.06
2-I-pyranyl $^4H_3$	–4633722.37	–4633825.84	–4633952.83	–4633955.63	–4633956.25

All methods are with SMD(dichloromethane)-(SARC)-DKH-def2TZVPP and energies are reported in kcal/mol.[a] RMSD values were calculated by comparing the absolute single point energy of the method with energies computed at SMD(dichloromethane)-CCSD(T)-(SARC)-DKH-def2TZVPP.[b] RMSD values were calculated by comparing the relative single point energy (within each cation separately) of the method with energies computed at SMD(dichloromethane)-CCSD(T)-(SARC)-DKH-def2TZVPP.

j. Supplementary Table S10. Single point benchmark energies for OLYP, OLYP-D4, OLYP-D3(BJ), PW6B95, PW6B95-D3(BJ), PW6B95-D4.

XVII.	OLYP	OLYP-D4	OLYP-D3(BJ)	PW6B95	PW6B95-D3(BJ)	PW6B95-D4
RMSD-absolute[b]	1831.08	1861.81	1864.82	2160.65	2164.17	2165.86
RMSD-relative[c]	0.36	0.61	0.37	0.28	0.33	0.34
Absolute Energies						
2-F-furanyl E_3	–207776.84	–207805.22	–207808.74	–208088.14	–208090.58	–208092.18
2-F-furanyl flat	–207775.71	–207804.02	–207807.42	–208086.90	–208089.34	–208090.94
2-F-furanyl ^3E	–207775.64	–207804.27	–207807.59	–208086.92	–208089.37	–208091.00
2-Cl-furanyl E_3	–434751.85	–434784.21	–434788.53	–435196.90	–435200.08	–435201.85
2-Cl-furanyl flat	–434751.71	–434784.22	–434788.35	–435196.60	–435199.80	–435201.59
2-Cl-furanyl ^3E	–434752.87	–434786.07	–434790.02	–435197.98	–435201.23	–435203.08
2-Br-furanyl E_3	–1780844.29	–1780877.93	–1780881.55	–1781362.07	–1781365.54	–1781367.37
2-Br-furanyl flat	–1780845.04	–1780878.73	–1780882.26	–1781362.70	–1781366.19	–1781368.01
2-Br-furanyl ^3E	–1780846.71	–1780881.11	–1780884.44	–1781364.75	–1781368.29	–1781370.17
2-I-furanyl E_3	–4610149.69	–4610184.46	–4610187.89	–4610442.76	–4610446.68	–4610448.35
2-I-furanyl flat	–4610150.89	–4610185.69	–4610189.09	–4610443.82	–4610447.75	–4610449.41
2-I-furanyl ^3E	–4610152.72	–4610188.15	–4610191.61	–4610446.20	–4610450.23	–4610451.90
2-F-pyranyl $^3H_4$	–232452.59	–232491.81	–232495.82	–232809.73	–232813.30	–232815.48
2-F-pyranyl $^4H_3$	–232454.63	–232493.97	–232498.18	–232812.04	–232815.61	–232817.79
2-Cl-pyranyl $^3H_4$	–459429.30	–459473.41	–459478.06	–459920.46	–459924.91	–459927.30
2-Cl-pyranyl $^4H_3$	–459429.22	–459472.86	–459477.87	–459920.51	–459924.88	–459927.24
2-Br-pyranyl $^3H_4$	–1805523.11	–1805568.61	–1805572.65	–1806087.32	–1806092.12	–1806094.53
2-Br-pyranyl $^4H_3$	–1805521.76	–1805566.41	–1805571.10	–1806085.84	–1806090.52	–1806092.88
2-I-pyranyl $^3H_4$	–4634828.62	–4634875.46	–4634879.45	–4635168.42	–4635173.75	–4635175.95
2-I-pyranyl $^4H_3$	–4634827.27	–4634874.05	–4634877.94	–4635166.94	–4635172.20	–4635174.45

All methods are with SMD(dichloromethane)-(SARC)-DKH-def2TZVPP and energies are reported in kcal/mol.[a] RMSD values were calculated by comparing the absolute single point energy of the method with energies computed at SMD(dichloromethane)-CCSD(T)-(SARC)-DKH-def2TZVPP.[b] RMSD values were calculated by comparing the relative single point energy (within each cation separately) of the method with energies computed at SMD(dichloromethane)-CCSD(T)-(SARC)-DKH-def2TZVPP.

k. **Supplementary Table S11.** Cartesian coordinates (in Å), energies (*E*, *H* and *G*, in kcal/mol, T=195.15 K), and number of imaginary vibrational frequencies (N_{imag}) of selected CEL geometries: the lowest ^3E, E_3 and flat geometries from each furanyl cations, while for the pyranyl cations the lowest $^3H_4$ and $^4H_3$ are given. All were optimized with CEL dihedral angle constrains (furanyl: C1–C2–C3–C4, C5–O5–C1–C2; pyranyl: C1–C2–C3–C4, C3–C4–C5–O5, C5–O5–C1–C2) at PCM(dichloromethane)-B3LYP-D3(BJ)BJ-(SARC)-DKH-def2TZVP.

2-F-furanyl E_3
E = -207731.85
H = -207668.65
G = -207681.71
N_{imag} = 0

O	0.109691	-1.039341	-1.107224
C	1.070500	-0.823745	-0.351122
C	0.847742	0.290157	0.624096
C	-0.661725	0.529798	0.525227
C	-1.040221	-0.086421	-0.815102
H	1.964389	-1.436479	-0.454736
H	1.439943	1.148651	0.278435
H	-0.908897	1.587093	0.564441
H	-1.155608	0.007476	1.344950
H	-1.027017	0.601587	-1.656794
H	-1.925555	-0.712415	-0.829516
F	1.286758	-0.066361	1.877344

2-F-furanyl flat
E = -207730.88
H = -207668.01
G = -207680.57
N_{imag} = 1

O	0.145713	-0.952393	-1.178994
C	1.114040	-0.729838	-0.437619
C	0.845808	0.247490	0.670409
C	-0.622523	0.630808	0.457385
C	-1.079395	-0.162793	-0.762877
H	2.043023	-1.266351	-0.621777
H	1.539836	1.089557	0.611652
H	-0.715740	1.700010	0.286533
H	-1.196922	0.360609	1.342308
H	-1.320025	0.432919	-1.638468
H	-1.832766	-0.925201	-0.588947
F	1.078950	-0.424818	1.860394

2-F-furanyl ^3E
E = -207730.77
H = -207667.50
G = -207680.53
N_{imag} = 0

O	0.166722	-0.851745	-1.239440
C	1.145611	-0.624093	-0.511433
C	0.819669	0.198926	0.711359
C	-0.575446	0.716500	0.390632
C	-1.101224	-0.224086	-0.678567
H	2.104624	-1.064258	-0.777495
H	1.562654	0.963444	0.930973
H	-0.498515	1.733042	0.005299
H	-1.199996	0.723594	1.282457
H	-1.575441	0.244093	-1.534706
H	-1.673819	-1.078368	-0.326218
F	0.825161	-0.737048	1.747141

2-Cl-furanyl E_3
E = -434668.90
H = -434606.46
G = -434620.01
N_{imag} = 0

O	0.088493	-1.031557	-1.134534
C	1.052369	-0.811194	-0.378748
C	0.838815	0.291506	0.587208
C	-0.676531	0.536661	0.499423
C	-1.059111	-0.080988	-0.840529
H	1.950468	-1.414239	-0.495464
H	1.434118	1.145313	0.243959
H	-0.910452	1.597411	0.533088
H	-1.184332	0.027583	1.316375
H	-1.051100	0.605205	-1.683837
H	-1.945445	-0.705759	-0.845592
Cl	1.462709	-0.159942	2.198651

2-Cl-furanyl flat
E = -434668.87
H = -434606.73
G = -434619.73
N_{imag} = 1

O	0.122713	-0.961277	-1.182084
C	1.100021	-0.705498	-0.454956
C	0.845245	0.283773	0.615521
C	-0.632979	0.658719	0.424765
C	-1.098515	-0.174249	-0.770169
H	2.036576	-1.223090	-0.649031
H	1.544480	1.117442	0.560740
H	-0.716945	1.722288	0.213834
H	-1.213685	0.429723	1.314328
H	-1.363354	0.395960	-1.655626
H	-1.844521	-0.934004	-0.557523
Cl	1.220963	-0.609788	2.140201

2-Cl-furanyl ^3E
E = -434669.76
H = -434607.20
G = -434620.61
N_{imag} = 0

O	0.138689	-0.878064	-1.224349
C	1.131960	-0.596583	-0.522356
C	0.829982	0.263485	0.646455
C	-0.581543	0.758333	0.347832
C	-1.116395	-0.243667	-0.664206
H	2.095171	-1.021174	-0.794105
H	1.577820	1.021584	0.857221
H	-0.508119	1.752266	-0.095319
H	-1.200319	0.816386	1.239946
H	-1.626686	0.178405	-1.523467
H	-1.670340	-1.082127	-0.248753
Cl	0.929781	-0.968845	1.981101

2-Br-furanyl E_3
E = -1780291.18
H = -1780228.92
G = -1780243.17
N_{imag} = 0

O	0.088479	-1.052219	-1.100891
C	1.094270	-0.688168	-0.454829
C	0.836444	0.336981	0.568675
C	-0.673889	0.592105	0.462723
C	-1.088300	-0.149237	-0.808087
H	2.041343	-1.180868	-0.658802
H	1.484205	1.205936	0.470867
H	-0.877276	1.657316	0.381882
H	-1.202622	0.196465	1.325565
H	-1.171098	0.468577	-1.698328
H	-1.938281	-0.815839	-0.710206
Br	1.406726	-0.571050	2.221432

2-Br-furanyl flat
E = -1780291.84
H = -1780229.99
G = -1780243.47
N_{imag} = 1

O	0.133768	-0.937956	-1.197588
C	1.114210	-0.648197	-0.478353
C	0.839450	0.323319	0.586968
C	-0.647297	0.654201	0.423293
C	-1.102724	-0.192484	-0.767755
H	2.067551	-1.120563	-0.699190
H	1.535089	1.157895	0.608073
H	-0.760711	1.715751	0.211433
H	-1.218406	0.415215	1.315810
H	-1.401511	0.371328	-1.646483
H	-1.823653	-0.972695	-0.542680
Br	1.264233	-0.765812	2.186471

2-Br-furanyl ^3E
E = -1780293.23
H = -1780230.94
G = -1780244.82
N_{imag} = 0

O	0.146611	-0.857625	-1.235324
C	1.142071	-0.540950	-0.540594
C	0.827030	0.302210	0.620853
C	-0.598928	0.755821	0.347920
C	-1.114516	-0.263609	-0.658268
H	2.116119	-0.923249	-0.832929
H	1.562620	1.056939	0.875008
H	-0.555565	1.751537	-0.097573
H	-1.214246	0.800408	1.242959
H	-1.654827	0.142533	-1.506723
H	-1.638823	-1.115057	-0.230095
Br	0.982454	-1.108958	2.014766

2-I-furanyl E_3
E = -4608636.00
H = -4608573.92
G = -4608588.34
N_{imag} = 0

O	0.064124	-1.058991	-1.094623
C	1.081434	-0.683991	-0.459771
C	0.841023	0.352889	0.537683
C	-0.667980	0.617929	0.445146
C	-1.100351	-0.144564	-0.807303
H	2.026718	-1.167416	-0.688604
H	1.511151	1.205726	0.462494
H	-0.866073	1.683045	0.348360
H	-1.199508	0.238035	1.312083
H	-1.191385	0.457408	-1.707704
H	-1.954996	-0.801408	-0.685963
I	1.455842	-0.698661	2.338201

2-I-furanyl flat
E = -4608636.72
H = -4608575.10
G = -4608588.93
N_{imag} = 1

O	0.108841	-0.950745	-1.189625
C	1.102888	-0.643737	-0.483185
C	0.846381	0.345546	0.551643
C	-0.639273	0.683358	0.403289
C	-1.113541	-0.190491	-0.762302
H	2.054982	-1.104779	-0.727765
H	1.562261	1.160436	0.597027
H	-0.749173	1.740998	0.168879
H	-1.208938	0.465915	1.301087
H	-1.425972	0.353470	-1.648796
H	-1.838242	-0.957154	-0.504448
I	1.299787	-0.902816	2.294196

2-I-furanyl ^3E
E = -4608638.48
H = -4608576.43
G = -4608590.64
N_{imag} = 0

O	0.120350	-0.872383	-1.228667
C	1.129703	-0.541360	-0.546755
C	0.836950	0.321920	0.588200
C	-0.589955	0.782339	0.327431
C	-1.122215	-0.258476	-0.649089
H	2.098153	-0.923205	-0.855219
H	1.584567	1.065901	0.837948
H	-0.546320	1.768428	-0.139598
H	-1.202862	0.846027	1.222413
H	-1.678257	0.130622	-1.495443
H	-1.648211	-1.091674	-0.187211
I	1.018097	-1.228141	2.125992

2-F-pyranyl $^{3}H_{4}$
E = -232406.06
H = -232324.49
G = -232338.12
N_{imag} = 0

O	-0.341843	-1.642981	-0.208580
C	0.739927	-1.373164	0.337033
C	1.232966	0.005474	0.632240
C	0.496583	1.080962	-0.140309
C	-0.999950	0.767073	-0.056237
C	-1.320849	-0.576129	-0.653777
H	1.369954	-2.231247	0.577203
H	1.080851	0.137466	1.715185
H	0.838080	1.074582	-1.177940
H	0.717733	2.054829	0.291710
H	-1.571754	1.512341	-0.612011
H	-1.341928	0.806308	0.980911
H	-1.224513	-0.617026	-1.737440
H	-2.274148	-0.992603	-0.342865
F	2.598892	-0.005883	0.394878

2-F-pyranyl $^{4}H_{3}$
E = -232403.92
H = -232322.42
G = -232336.26
N_{imag} = 0

O	-0.468860	-1.618105	-0.029417
C	0.739088	-1.410891	0.160761
C	1.332972	-0.067457	0.453308
C	0.340857	1.073891	0.390706
C	-0.783107	0.796628	-0.608090
C	-1.466330	-0.511850	-0.317134
H	1.371400	-2.299636	0.169319
H	1.769369	-0.163039	1.456549
H	0.882286	1.983863	0.133637
H	-0.072211	1.213174	1.392654
H	-0.388304	0.786875	-1.627090
H	-1.543213	1.577415	-0.557421
H	-2.020033	-0.935037	-1.150153
H	-2.080529	-0.503314	0.582006
F	2.386614	0.077483	-0.449635

2-Cl-pyranyl $^{3}H_{4}$
E = -459342.74
H = -459261.92
G = -459276.03
N_{imag} = 0

O	-0.459619	-1.606182	-0.220095
C	0.599432	-1.411503	0.402830
C	1.158440	-0.075076	0.720507
C	0.542070	1.041040	-0.107726
C	-0.974409	0.840297	-0.129562
C	-1.331256	-0.487628	-0.744318
H	1.115013	-2.316772	0.723051
H	0.980861	0.072413	1.793236
H	0.948059	1.007783	-1.120329
H	0.803413	2.001630	0.331412
H	-1.449420	1.616667	-0.731921
H	-1.388490	0.911764	0.878837
H	-1.158987	-0.540540	-1.817810
H	-2.328737	-0.843680	-0.504325
Cl	2.943630	-0.210212	0.526212

2-Cl-pyranyl $^{4}H_{3}$
E = -459342.75
H = -459261.79
G = -459275.92
N_{imag} = 0

O	-0.545856	-1.592538	-0.138086
C	0.597533	-1.445270	0.332367
C	1.234630	-0.142275	0.630591
C	0.347691	1.069464	0.435984
C	-0.696976	0.849321	-0.658236
C	-1.456108	-0.437561	-0.475011
H	1.139106	-2.371615	0.518189
H	1.709068	-0.187595	1.610085
H	0.961430	1.942867	0.225048
H	-0.150257	1.249929	1.393975
H	-0.219442	0.852819	-1.640648
H	-1.425423	1.661277	-0.650916
H	-1.956570	-0.792309	-1.370411
H	-2.148070	-0.429156	0.366181
Cl	2.609246	-0.227358	-0.579111

2-Br-pyranyl $^{3}H_{4}$
E = -1804964.75
H = -1804884.23
G = -1804898.84
N_{imag} = 0

O	-0.598525	-1.558006	0.212443
C	0.391242	-1.236953	0.899653
C	1.031630	0.092216	0.873815
C	0.636309	0.925232	-0.331245
C	-0.878952	0.818043	-0.519724
C	-1.281742	-0.616298	-0.749962
H	0.751770	-2.020231	1.565359
H	0.804027	0.575319	1.829897
H	1.150426	0.553054	-1.218976
H	0.937956	1.958697	-0.173868
H	-1.193409	1.391956	-1.392963
H	-1.406141	1.223561	0.346788
H	-0.979165	-1.008229	-1.719437
H	-2.331543	-0.826914	-0.569514
Br	2.966119	-0.271447	0.947733

2-Br-pyranyl $^{4}H_{3}$
E = -1804966.29
H = -1804885.59
G = -1804900.18
N_{imag} = 0

O	-0.685638	-1.516468	0.280847
C	0.394282	-1.277656	0.866590
C	1.097045	0.011523	0.845606
C	0.379734	1.141907	0.148803
C	-0.516213	0.636105	-0.980926
C	-1.415680	-0.487603	-0.537336
H	0.793354	-2.112753	1.438203
H	1.507400	0.249025	1.824002
H	1.096984	1.876425	-0.211795
H	-0.228311	1.639278	0.912923
H	0.092791	0.305584	-1.825004
H	-1.154954	1.442957	-1.342604
H	-1.821587	-1.077646	-1.352955
H	-2.215746	-0.171989	0.131050
Br	2.676539	-0.658689	-0.197404

2-I-pyranyl $^{3}H_{4}$
E = -4633309.75
H = -4633229.12
G = -4633244.04
N_{imag} = 0

O	-0.674826	0.310557	1.507595
C	0.214844	1.092672	1.073906
C	1.075383	0.819273	-0.075688
C	0.562267	-0.233033	-1.059485
C	-0.922725	-0.490427	-0.795727
C	-1.136010	-0.829825	0.653793
H	0.361327	1.985386	1.677374
H	1.396023	1.752615	-0.531046
H	1.105141	-1.169401	-0.944977
H	0.728690	0.126100	-2.073759
H	-1.266671	-1.336573	-1.392711
H	-1.534294	0.374574	-1.065735
H	-0.556734	-1.690411	0.988233
H	-2.176717	-0.938850	0.942591
I	2.824302	0.227343	1.095636

2-I-pyranyl $^{4}H_{3}$
E = -4633311.38
H = -4633230.89
G = -4633245.77
N_{imag} = 0

O	-0.702700	0.289242	1.507434
C	0.383243	0.878716	1.263021
C	1.081709	0.855650	-0.013362
C	0.373304	0.157719	-1.147781
C	-0.520619	-0.971766	-0.640854
C	-1.423510	-0.529101	0.481303
H	0.758518	1.475599	2.090336
H	1.530608	1.817413	-0.246680
H	1.088060	-0.206942	-1.882351
H	-0.236549	0.919556	-1.647242
H	0.092019	-1.812150	-0.307600
H	-1.153577	-1.339422	-1.449455
H	-1.829791	-1.349084	1.065347
H	-2.226778	0.131965	0.158468
I	2.786063	-0.317395	0.769416

IX. X-Ray Crystallographic Data

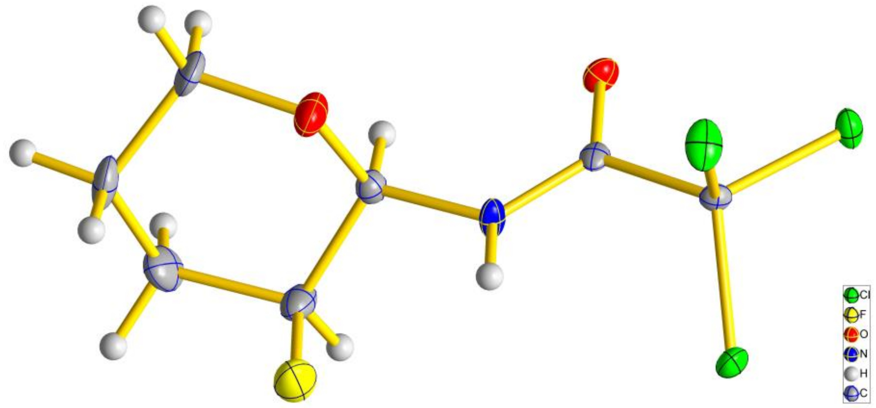

Figure S1. The molecular structure of amide *cis*-**S3** (ellipsoids set at 50% probability).

A colorless platelet-like specimen of $C_7H_9Cl_3FNO_2$, approximate dimensions 0.020 mm x 0.200 mm x 0.270 mm, was used for the X-ray crystallographic analysis. The X-ray intensity data were measured on a Bruker D8 SMART APEXII three-circle diffractometer system equipped with a Incotec microfocus sealed X-ray tube (MoKα, λ = 0.71073 Å) and a multilayer optics monochromator.

A total of 1620 frames were collected. The total exposure time was 16.25 hours. The frames were integrated with the Bruker SAINT software package using a narrow-frame algorithm. The integration of the data using a monoclinic unit cell yielded a total of 8031 reflections to a maximum θ angle of 28.44° (0.75 Å resolution), of which 2491 were independent (average redundancy 3.224, completeness = 99.5%, R_{int} = 5.22%, R_{sig} = 5.63%) and 2298 (92.25%) were greater than $2\sigma(F^2)$. The final cell constants of a = 9.6870(18) Å, b = 5.9907(11) Å, c = 8.9661(17) Å, β = 100.508(6)°, volume = 511.59(17) $Å^3$, are based upon the refinement of the XYZ-centroids of 3131 reflections above 20 σ(I) with 6.801° < 2θ < 56.37°. Data were corrected for absorption effects using the Multi-Scan method (SADABS). The ratio of minimum to maximum apparent transmission was 0.877. The calculated minimum and maximum transmission coefficients (based on crystal size) are 0.7970 and 0.9830.

The structure was solved and refined using the Bruker SHELXTL Software Package, using the space group P 1 c 1, with Z = 2 for the formula unit, $C_7H_9Cl_3FNO_2$. The final anisotropic full-matrix least-squares refinement on F^2 with 132 variables converged at R1 = 4.03%, for the observed data and wR2 = 9.45% for all data. The goodness-of-fit was 1.144. The largest peak in the final difference electron density synthesis was 0.482 $e^-/Å^3$ and the largest hole was -0.456 $e^-/Å^3$ with an RMS deviation of 0.115 $e^-/Å^3$. On the basis of the final model, the calculated density was 1.717 g/cm^3 and F(000), 268 e^-.

Table S12. Sample and crystal data for amide *cis*-**S3**.

Identification code	21kaw7h	
Chemical formula	$C_7H_9Cl_3FNO_2$	
Formula weight	264.50 g/mol	
Temperature	100(2) K	
Wavelength	0.71073 Å	
Crystal size	0.020 x 0.200 x 0.270 mm	
Crystal habit	colorless platelet	
Crystal system	monoclinic	
Space group	P 1 c 1	
Unit cell dimensions	a = 9.6870(18) Å	α = 90°
	b = 5.9907(11) Å	β = 100.508(6)°
	c = 8.9661(17) Å	γ = 90°
Volume	511.59(17) $Å^3$	
Z	2	
Density (calculated)	1.717 g/cm^3	
Absorption coefficient	0.882 mm^{-1}	
F(000)	268	

Table S13. Data collection and structure refinement for amide *cis*-**S3**.

Diffractometer	Bruker D8b SMART APEXII three-circle diffractometer	
Radiation source	Incotec microfocus sealed X-ray tube (MoKα, λ = 0.71073 Å)	
Theta range for data collection	2.14 to 28.44°	
Index ranges	-12<=h<=12, -7<=k<=7, -12<=l<=12	
Reflections collected	8031	
Independent reflections	2491 [R(int) = 0.0522]	
Coverage of independent reflections	99.5%	
Absorption correction	Multi-Scan	
Max. and min. transmission	0.9830 and 0.7970	
Structure solution technique	direct methods	
Structure solution program	SHELXT (Sheldrick 2015)	
Refinement method	Full-matrix least-squares on F^2	
Refinement program	SHELXL-2018/3 (Sheldrick, 2018)	
Function minimized	Σ $w(F_o^2 - F_c^2)^2$	
Data / restraints / parameters	2491 / 3 / 132	
Goodness-of-fit on F^2	1.144	
Final R indices	2298 data; I>2σ(I)	R1 = 0.0403, wR2 = 0.0917
	all data	R1 = 0.0463, wR2 = 0.0945
Weighting scheme	$w=1/[\sigma^2(F_o^2)+(0.0445P)^2$ where $P=(F_o^2+2F_c^2)/3$	
Absolute structure parameter	0.17(12)	
Largest diff. peak and hole	0.482 and -0.456 $eÅ^{-3}$	
R.M.S. deviation from mean	0.115 $eÅ^{-3}$	

X. References

[1] D. A. L. Otte, D. E. Borchmann, C. Lin, M. Weck, K. A. Woerpel, *Org. Lett.* **2014**, *16*, 1566–1569.
[2] S. Yin, L. Li, L. Su, H. Li, Y. Zhao, Y. Wu, R. Liu, F. Zou, G. Ni, *Carbohydr. Res.* **2022**, *517*, 108575.
[3] T. Haidzinskaya, H. A. Kerchner, J. Liu, M. P. Watson, *Org. Lett.* **2015**, *17*, 3857–3859.
[4] T. R. Reddy, D. S. Rao, K. Babachary, S. Kashyap, *Eur. J. Org. Chem.* **2016**, *2016*, 291–301.
[5] T. M. Barbosa, R. V. Viesser, R. J. Abraham, R. Rittner, C. F. Tormena, *RSC Adv.* **2015**, *5*, 35412–35420.
[6] A. El-Qisairi, P. M. Henry, *J. Organomet. Chem.* **2000**, *603*, 50–60.
[7] E. Durantie, C. Bucher, R. Gilmour, *Chem. - Eur. J.* **2012**, *18*, 8208–8215.
[8] K. M. Demkiw, C. T. Hu, K. A. Woerpel, *J. Org. Chem.* **2022**, *87*, 5315–5327.
[9] H. R. Khatri, H. Nguyen, J. K. Dunaway, J. Zhu, *Chem. - Eur. J.* **2015**, *21*, 13553–13557.
[10] T. H. Fife, L. K. Jao, *J. Am. Chem. Soc.* **1968**, *90*, 4081–4085.
[11] a) A. Garcia, D. A. L. Otte, W. A. Salamant, J. R. Sanzone, K. A. Woerpel, *Angew. Chem., Int. Ed.* **2015**, *54*, 3061–3064; b) A. Garcia, D. A. L. Otte, W. A. Salamant, J. R. Sanzone, K. A. Woerpel, *J. Org. Chem.* **2015**, *80*, 4470–4480.
[12] C. A. G. Haasnoot, *J. Am. Chem. Soc.* **1993**, *115*, 1460–1468.
[13] a) C. H. Bushweller, J. W. O'Neil, *J. Org. Chem.* **1970**, *35*, 276–278; b) H. J. Schneider, V. Hoppen, *J. Org. Chem.* **1978**, *43*, 3866–3873; c) N. D. Bartolo, K. M. Demkiw, E. M. Valentín, C. T. Hu, A. A. Arabi, K. A. Woerpel, *J. Org. Chem.* **2021**, *86*, 7203–7217.
[14] a) A. Ouedraogo, J. Lessard, *Can. J. Chem.* **1991**, *69*, 474–480; b) A. J. Briggs, R. Glenn, P. G. Jones, A. J. Kirby, P. Ramaswamy, *J. Am. Chem. Soc.* **1984**, *106*, 6200–6206.
[15] a) S. Källström, R. B. C. Jagt, R. Sillanpää, B. L. Feringa, A. J. Minnaard, R. Leino, *Eur. J. Org. Chem.* **2006**, *2006*, 3826–3833; b) G. Mehta, N. S. Likhite, *Tetrahedron Lett.* **2009**, *50*, 5263–5266; c) C. Reddy, S. A. Babu, N. A. Aslam, V. Rajkumar, *Eur. J. Org. Chem.* **2013**, 2362–2380.
[16] J. W. Emsley, L. Phillips, V. Wray, *Fluorine Coupling Constants*, Pergamin Press, **1976**.
[17] L. Crombie, R. D. Wyvill, *J. Chem. Soc., Perkin Trans. 1* **1985**, 1971–1981.
[18] H.-R. Krüger, H. Marschall, P. Weyerstahl, F. Nerdel, *Chem. Ber.* **1973**, *106*, 91–104.
[19] W. C. Agosta, S. Wolff, *J. Org. Chem.* **1975**, *40*, 1699–1701.
[20] T. Hansen, L. Lebedel, W. A. Remmerswaal, S. van der Vorm, D. P. A. Wander, M. Somers, H. S. Overkleeft, D. V. Filippov, J. Désiré, A. Mingot, Y. Bleriot, G. A. van der Marel, S. Thibaudeau, J. D. C. Codée, *ACS Cent. Sci.* **2019**, *5*, 781–788.
[21] E. R. van Rijssel, P. van Delft, G. Lodder, H. S. Overkleeft, G. A. van der Marel, D. V. Filippov, J. D. C. Codée, *Angew. Chem., Int. Ed.* **2014**, *53*, 10381–10385.
[22] a) F. Neese, F. Wennmohs, U. Becker, C. Riplinger, *J. Chem. Phys.* **2020**, *152*, 224108; b) F. Neese, *WIREs Comput. Mol. Sci.* **2022**, e1606.
[23] a) G. Santra, N. Sylvetsky, J. M. L. Martin, *J. Phys. Chem. A* **2019**, *123*, 5129–5143; b) E. Caldeweyher, C. Bannwarth, S. Grimme, *J. Chem. Phys.* **2017**, *147*, 034112; c) A. D. Becke, *J. Chem. Phys.* **1993**, *98*, 5648–5652; d) C. Lee, W. Yang, R. G. Parr, *Phys. Rev. B.* **1988**, *37*, 785–789; e) S. H. Vosko, L. Wilk, M. Nusair, *Can. J. Phys.* **1980**, *58*, 1200–1211; f) P. J. Stephens, F. J. Devlin, C. F. Chabalowski, M. J. Frisch, *J. Phys. Chem.* **1994**, *98*, 11623–11627; g) S. Grimme, J. Antony, S. Ehrlich, H. Krieg, *J. Chem. Phys.* **2010**, *132*, 154104; h) S. Grimme, S. Ehrlich, L. Goerigk, *J. Comput. Chem.* **2011**, *32*, 1456–1465; i) F. Weigend, R. Ahlrichs, *Phys. Chem. Chem. Phys.* **2005**, *7*, 3297–3305; j) F. Weigend, *Phys. Chem. Chem. Phys.* **2006**, *8*, 1057–1065.
[24] J. D. Rolfes, F. Neese, D. A. Pantazis, *J. Comput. Chem.* **2020**, *41*, 1842–1849.
[25] OriginPro, 9.0.0., OriginLab Corporation, Northampton, MA, USA.
[26] J. M. Madern, T. Hansen, E. R. van Rijssel, H. A. V. Kistemaker, S. van der Vorm, H. S. Overkleeft, G. A. van der Marel, D. V. Filippov, J. D. C. Codée, *J. Org. Chem.* **2019**, *84*, 1218–1227.
[27] G. L. Stoychev, A. A. Auer, F. Neese, *J. Chem. Theory Comput.* **2017**, *13*, 554–562.
[28] A. V. Marenich, C. J. Cramer, D. G. Truhlar, *J. Phys. Chem. B.* **2009**, *113*, 6378–6396.
[29] Y. Guo, C. Riplinger, U. Becker, D. G. Liakos, Y. Minenkov, L. Cavallo, F. Neese, *J. Chem. Phys.* **2018**, *148*, 011101.
[30] a) P. Pinski, F. Neese, *J. Chem. Phys.* **2018**, *148*, 031101; b) P. Pinski, F. Neese, *J. Chem. Phys.* **2019**, *150*, 164102.
[31] S. Kozuch, J. M. L. Martin, *Phys. Chem. Chem. Phys.* **2011**, *13*, 20104–20107.
[32] S. Kozuch, J. M. L. Martin, *J. Comput. Chem.* **2013**, *34*, 2327–2344.
[33] Y. Zhao, D. G. Truhlar, *Theor. Chem. Acc.* **2008**, *120*, 215–241.
[34] S. Grimme, *J. Chem. Phys.* **2006**, *124*, 034108.
[35] T. Schwabe, S. Grimme, *Phys. Chem. Chem. Phys.* **2006**, *8*, 4398–4401.
[36] É. Brémond, Á. J. Pérez-Jiménez, J. C. Sancho-García, C. Adamo, *J. Chem. Phys.* **2019**, *150*, 201102.
[37] É. Brémond, J. C. Sancho-García, Á. J. Pérez-Jiménez, C. Adamo, *J. Chem. Phys.* **2014**, *141*, 031101.
[38] N. Mardirossian, M. Head-Gordon, *J. Chem. Phys.* **2015**, *142*, 074111.
[39] E. Brémond, C. Adamo, *J. Chem. Phys.* **2011**, *135*, 024106.
[40] S. Grimme, *J. Comput. Chem.* **2004**, *25*, 1463–1473.
[41] N. Mardirossian, M. Head-Gordon, *J. Chem. Phys.* **2016**, *144*, 214110.
[42] J.-D. Chai, M. Head-Gordon, *J. Chem. Phys.* **2009**, *131*, 174105.
[43] N. Mardirossian, M. Head-Gordon, *Phys. Chem. Chem. Phys.* **2014**, *16*, 9904–9924.
[44] J. Sun, A. Ruzsinszky, J. P. Perdew, *Phys. Rev. Lett.* **2015**, *115*, 036402.
[45] A. P. Bartók, J. R. Yates, *J. Chem. Phys.* **2019**, *150*, 161101.
[46] J. W. Furness, A. D. Kaplan, J. Ning, J. P. Perdew, J. Sun, *J. Phys. Chem. Lett.* **2020**, *11*, 8208–8215.
[47] a) B. Miehlich, A. Savin, H. Stoll, H. Preuss, *Chem. Phys. Lett.* **1989**, *157*, 200–206; b) N. C. Handy, A. J. Cohen, *Mol. Phys.* **2001**, *99*, 403–412.
[48] Y. Zhao, D. G. Truhlar, *J. Phys. Chem. A* **2005**, *109*, 5656–5667.
[49] L. Goerigk, A. Hansen, C. Bauer, S. Ehrlich, A. Najibi, S. Grimme, *Phys. Chem. Chem. Phys.* **2017**, *19*, 32184–32215.

XI. Selected ^{1}H, $^{13}C\{^{1}H\}$, and $^{19}F\{^{1}H\}$ NMR Spectra

The following pages contain ^{1}H, $^{13}C\{^{1}H\}$, and $^{19}F\{^{1}H\}$ spectra for all new compounds. Unpurified ^{1}H or $^{13}C\{^{1}H\}$ NMR spectra are also included if they were used to calculate diastereomeric ratios. There are no resonances with chemical shifts beyond the shown area of the spectra.

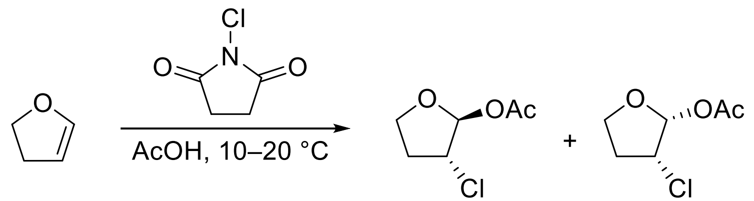

S2 trans-11 cis-11

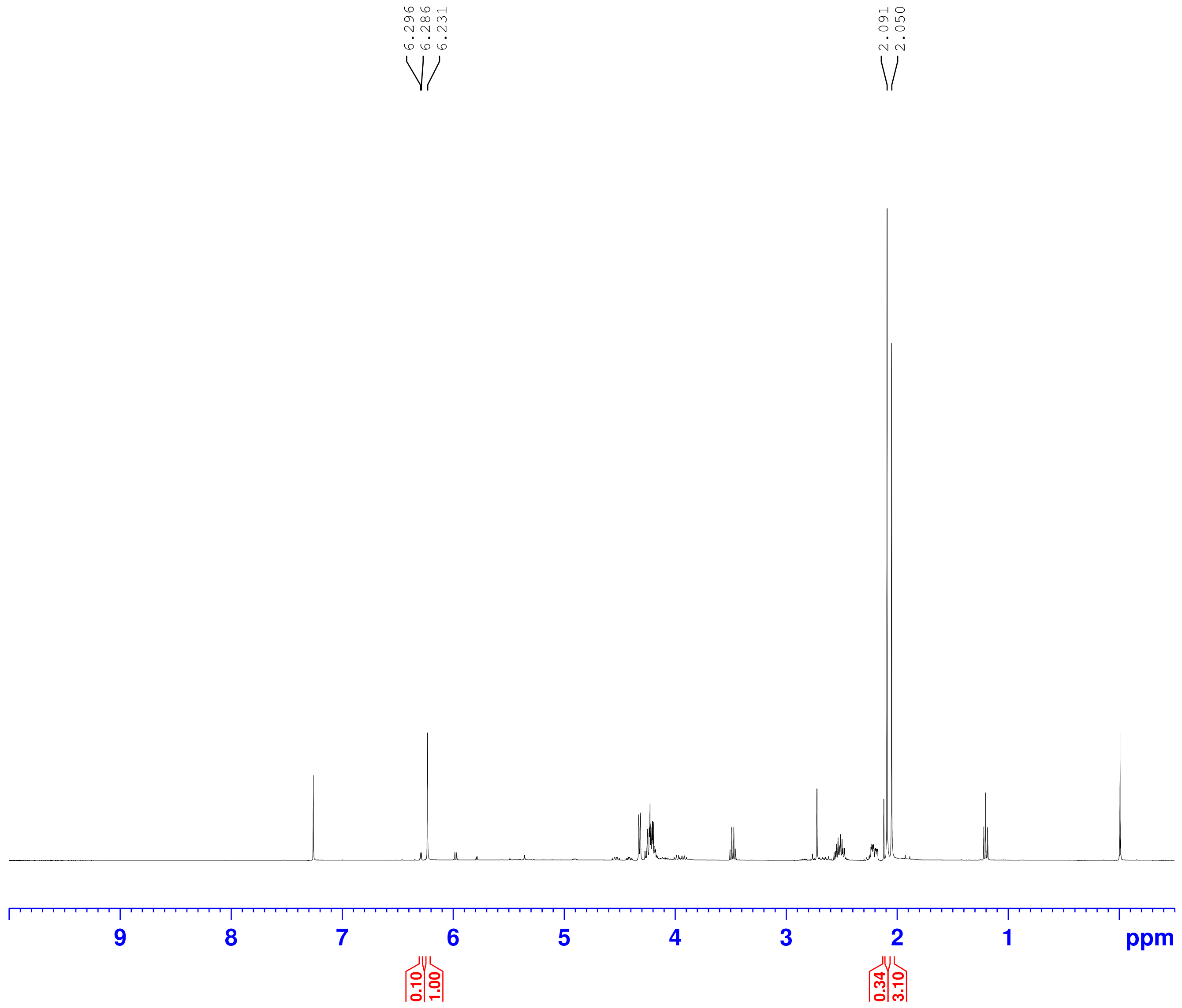


```
Current Data Parameters
NAME             kmd-3-179
EXPNO                    2
PROCNO                   1

F2 - Acquisition Parameters
Date_            20191115
Time                10.57 h
INSTRUM             spect
PROBHD    Z150354_0001 (
PULPROG              zg30
TD                  65536
SOLVENT             CDCl3
NS                      4
DS                      0
SWH              8012.820 Hz
FIDRES           0.244532 Hz
AQ              4.0894465 sec
RG                   92.4
DW                 62.400 usec
DE                  30.00 usec
TE                  298.0 K
D1            30.00000000 sec
TD0                     1
SFO1          400.3024719 MHz
NUC1                   1H
P1                  12.00 usec
PLW1           4.67290020 W

F2 - Processing parameters
SI                  65536
SF            400.3000094 MHz
WDW                    EM
SSB      0
LB                   0.30 Hz
GB       0
PC                   1.00
```

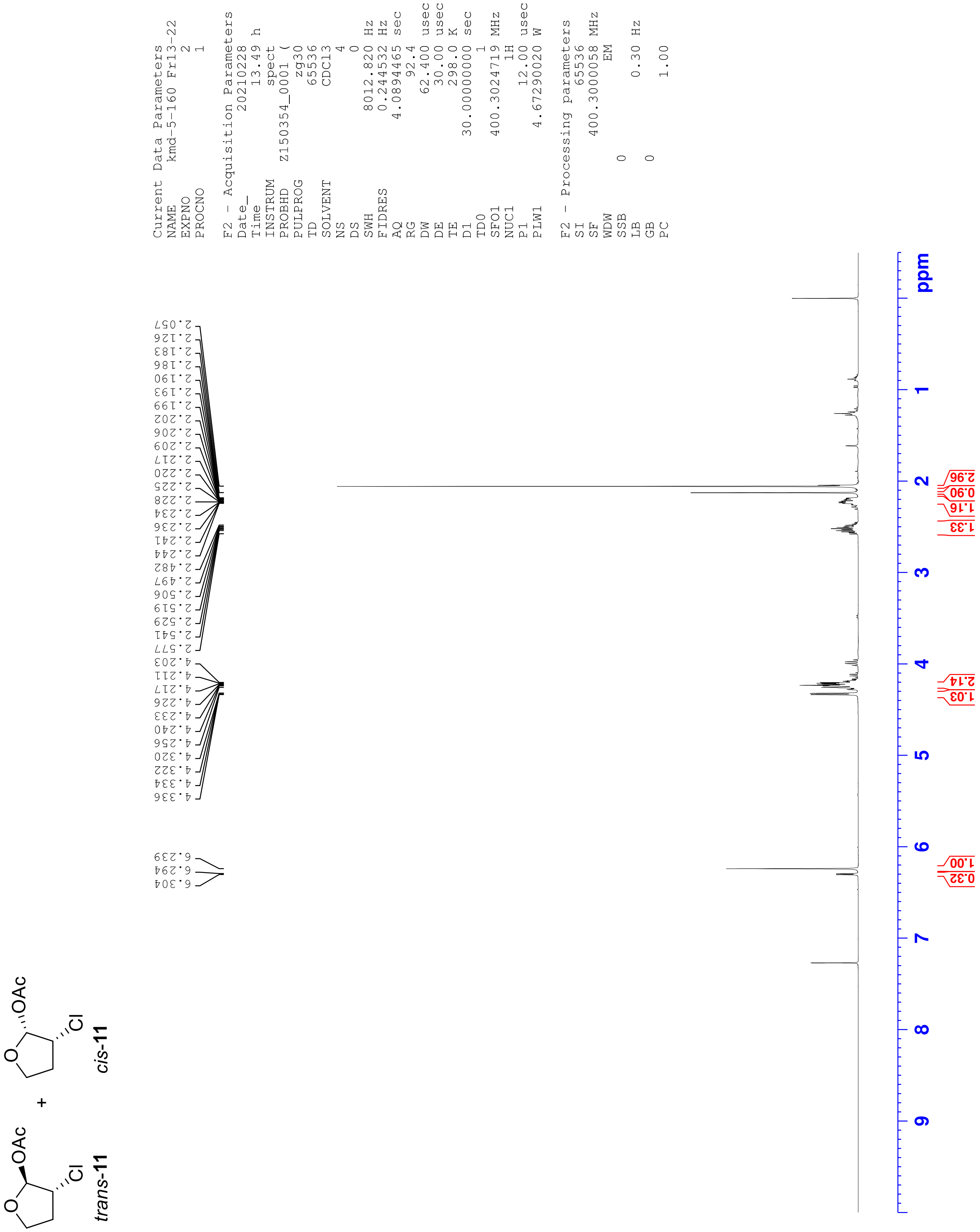
trans-11
cis-11
OAc
Cl
O
Current Data Parameters
NAME kmd-5-160 Fr13-22
EXPNO 2
PROCNO 1
F2 - Acquisition Parameters
Date_ 20210228
Time 13.49 h
INSTRUM spect
PROBHD Z150354_0001 (
PULPROG zg30
TD 65536
SOLVENT CDCl3
NS 4
DS 0
SWH 8012.820 Hz
FIDRES 0.244532 Hz
AQ 4.0894465 sec
RG 92.4
DW 62.400 usec
DE 30.00 usec
TE 298.0 K
D1 30.00000000 sec
TD0 1
SFO1 400.3024719 MHz
NUC1 1H
P1 12.00 usec
PLW1 4.67290020 W
F2 - Processing parameters
SI 65536
SF 400.3000058 MHz
WDW EM
SSB 0
LB 0.30 Hz
GB 0
PC 1.00
ppm

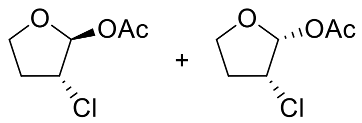

trans-**11** + *cis*-**11**

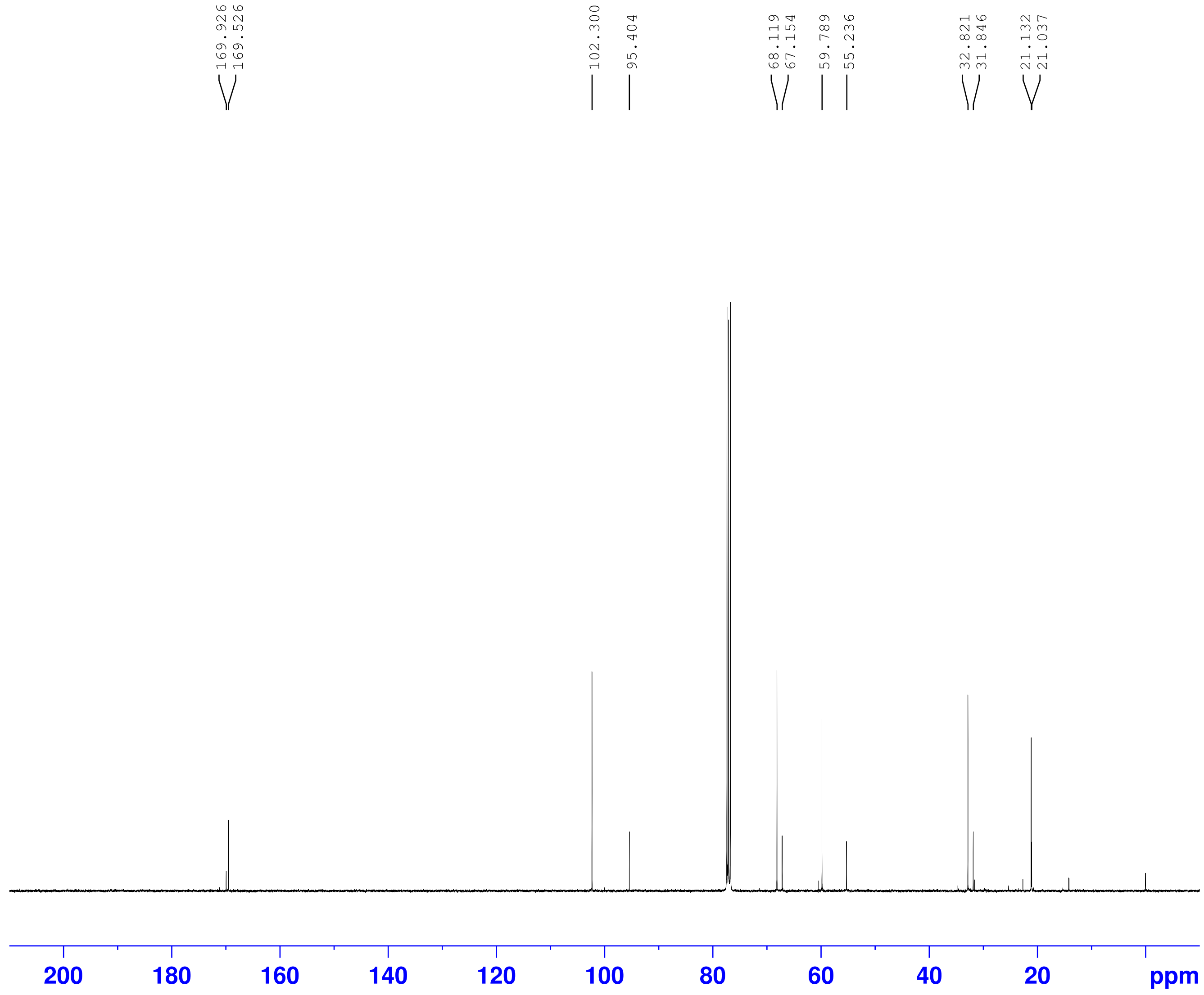


```
Current Data Parameters
NAME      kmd-5-160 Fr13-22
EXPNO                   3
PROCNO                  1

F2 - Acquisition Parameters
Date_            20210228
Time                14.05 h
INSTRUM             spect
PROBHD    Z150354_0001 (
PULPROG           zgpg30
TD                 65356
SOLVENT            CDCl3
NS                   256
DS                     4
SWH            24038.461 Hz
FIDRES          0.735616 Hz
AQ             1.3594048 sec
RG                  31.4
DW                20.800 usec
DE                 25.00 usec
TE                 298.0 K
D1            2.00000000 sec
D11           0.03000000 sec
TD0                    1
SFO1         100.6655806 MHz
NUC1                 13C
P1                 10.00 usec
PLW1         19.75099945 W
SFO2         400.3016012 MHz
NUC2                  1H
CPDPRG[2         waltz16
PCPD2              80.00 usec
PLW2          4.67290020 W
PLW12         0.10514000 W
PLW13         0.05280100 W

F2 - Processing parameters
SI                 32768
SF           100.6555150 MHz
WDW                   EM
SSB      0
LB                  1.00 Hz
GB       0
PC                  1.40
```

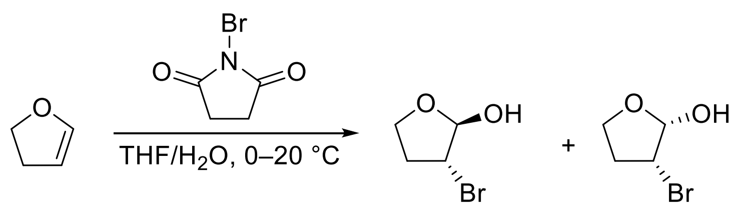

S2 ... *trans*-**16** + *cis*-**16**

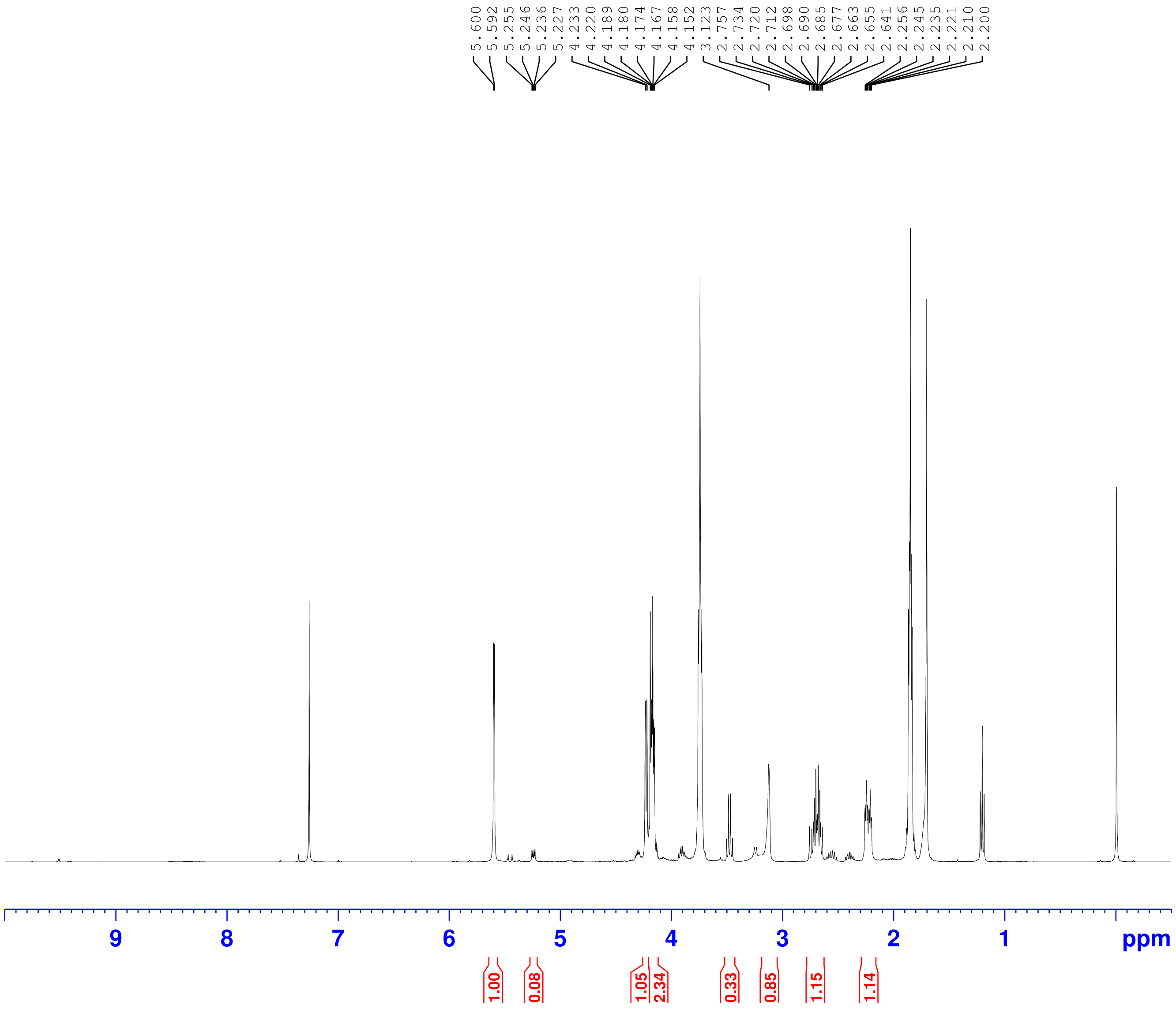


```
Current Data Parameters
NAME             kmd-3-136
EXPNO                    1
PROCNO                   1

F2 - Acquisition Parameters
Date_             20191009
Time                 11.44 h
INSTRUM              spect
PROBHD    Z150354_0001 (
PULPROG               zg30
TD                   65536
SOLVENT              CDCl3
NS                      16
DS                       2
SWH               8012.820 Hz
FIDRES            0.244532 Hz
AQ               4.0894465 sec
RG                  103.17
DW                  62.400 usec
DE                   30.00 usec
TE                   298.0 K
D1              1.00000000 sec
TD0                      1
SFO1           400.3024719 MHz
NUC1                    1H
P1                   12.00 usec
PLW1            4.67290020 W

F2 - Processing parameters
SI                   65536
SF             400.3000095 MHz
WDW                     EM
SSB      0
LB                    0.30 Hz
GB       0
PC                    1.00
```

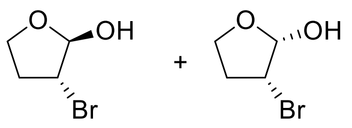

trans-**16** + *cis*-**16**

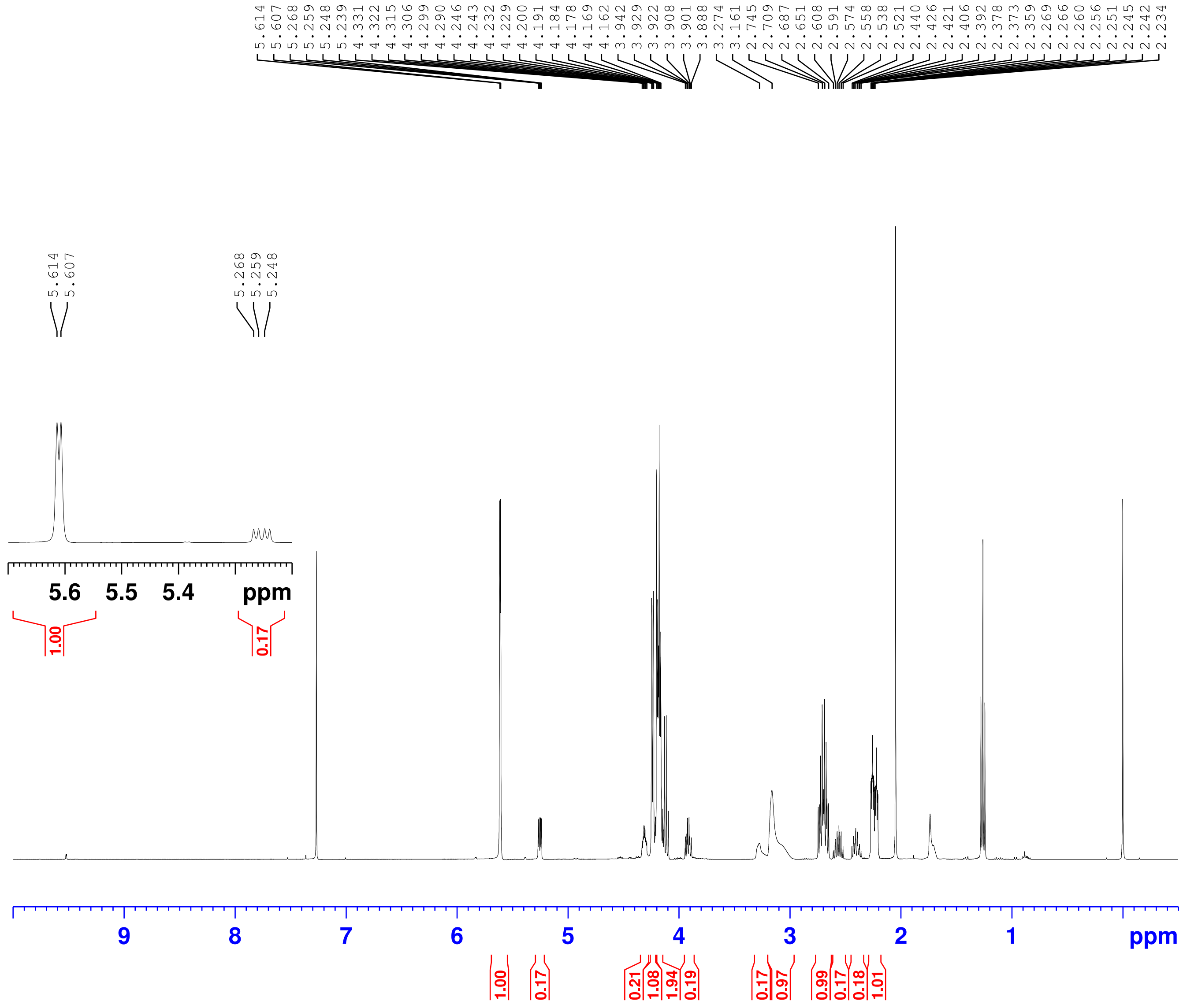


```
Current Data Parameters
NAME       kmd-3-136 Fr10-18
EXPNO                      2
PROCNO                     1

F2 - Acquisition Parameters
Date_             20191009
Time                 15.40 h
INSTRUM              spect
PROBHD    Z150354_0001 (
PULPROG               zg30
TD                   65536
SOLVENT              CDCl3
NS                       4
DS                       0
SWH               8012.820 Hz
FIDRES            0.244532 Hz
AQ               4.0894465 sec
RG                    92.4
DW                  62.400 usec
DE                   30.00 usec
TE                   298.0 K
D1             30.00000000 sec
TD0                      1
SFO1           400.3024719 MHz
NUC1                    1H
P1                   12.00 usec
PLW1            4.67290020 W

F2 - Processing parameters
SI                   65536
SF             400.3000065 MHz
WDW                     EM
SSB       0
LB                    0.30 Hz
GB        0
PC                    1.00
```

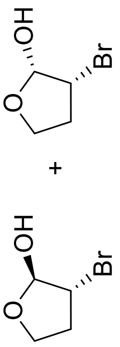

trans-16 + cis-16

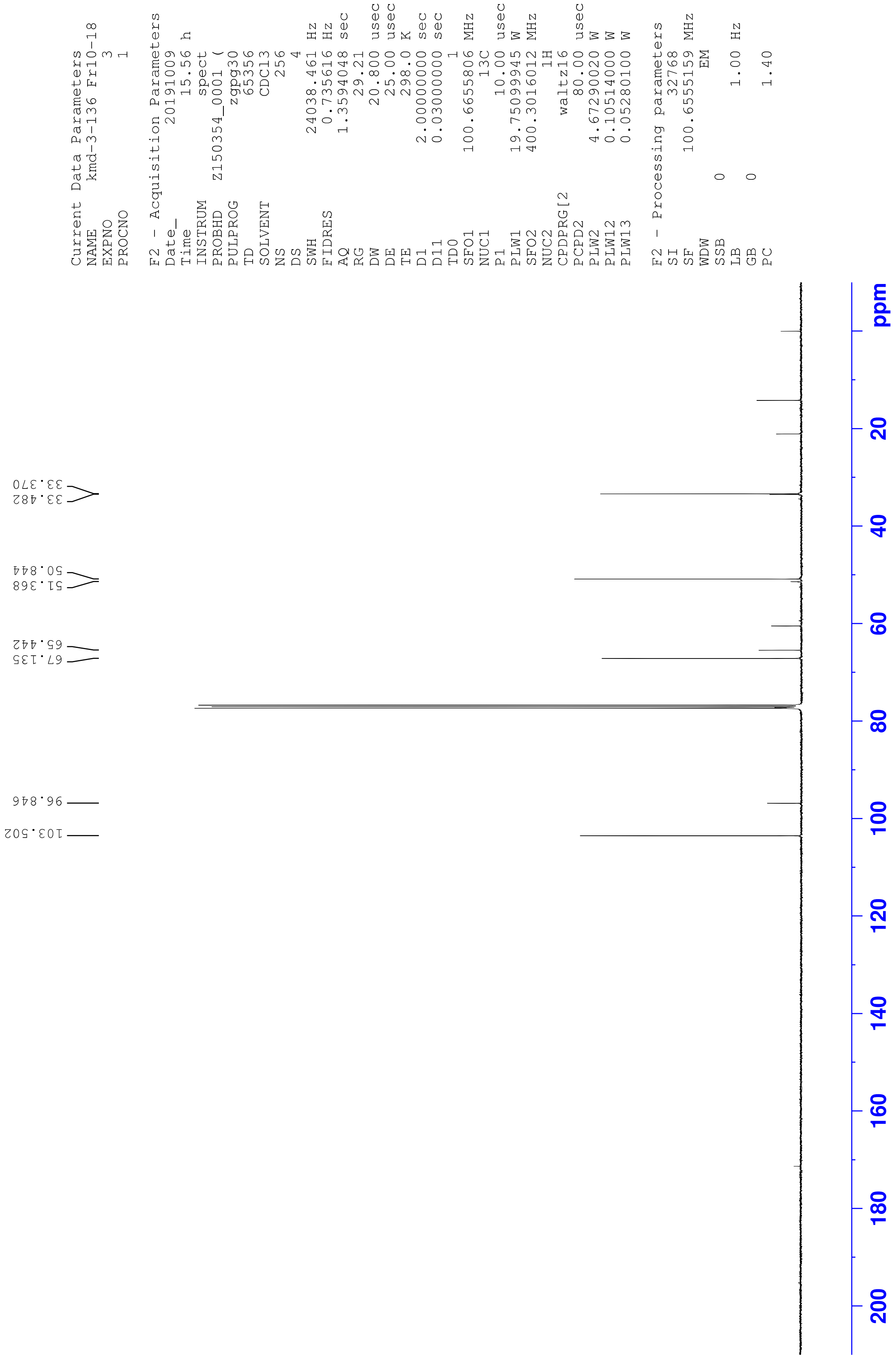

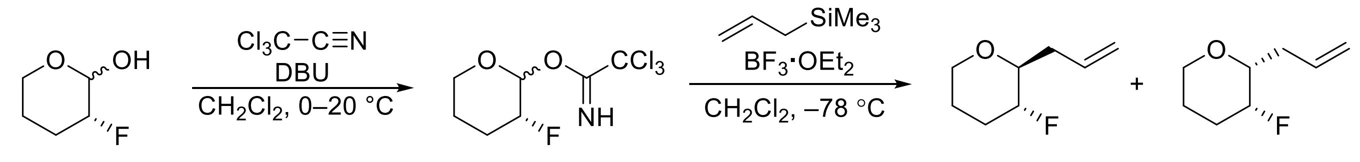

9 **1a** *trans*-**2a** *cis*-**2a**

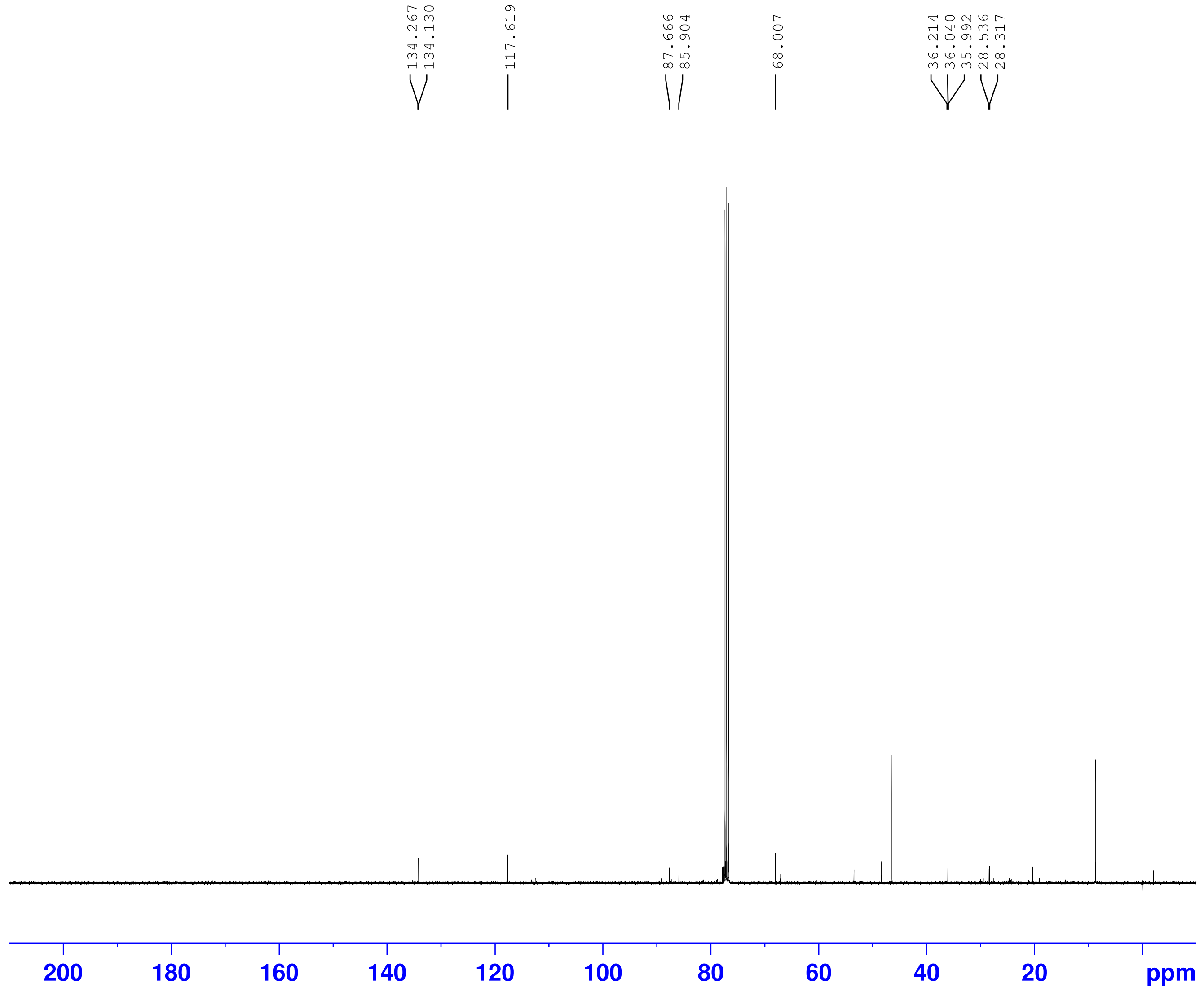


```
Current Data Parameters
NAME          kmd-6-168
EXPNO                 4
PROCNO                1

F2 - Acquisition Parameters
Date_          20220225
Time               4.03 h
INSTRUM           spect
PROBHD   Z150354_0001 (
PULPROG         zgpg30
TD                65356
SOLVENT           CDCl3
NS                 1024
DS                    4
SWH           24038.461 Hz
FIDRES         0.735616 Hz
AQ            1.3594048 sec
RG                25.77
DW               20.800 usec
DE                25.00 usec
TE                298.0 K
D1           2.00000000 sec
D11          0.03000000 sec
TD0                   1
SFO1        100.6655806 MHz
NUC1                13C
P1                10.00 usec
PLW1        17.99699974 W
SFO2        400.3016012 MHz
NUC2                 1H
CPDPRG[2        waltz16
PCPD2             80.00 usec
PLW2         4.64209986 W
PLW12        0.10445000 W
PLW13        0.05245300 W

F2 - Processing parameters
SI               131072
SF          100.6555154 MHz
WDW                  EM
SSB      0
LB       0 Hz
GB       0
PC                 1.40
```

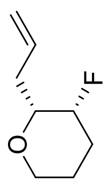
O
F

cis-2a

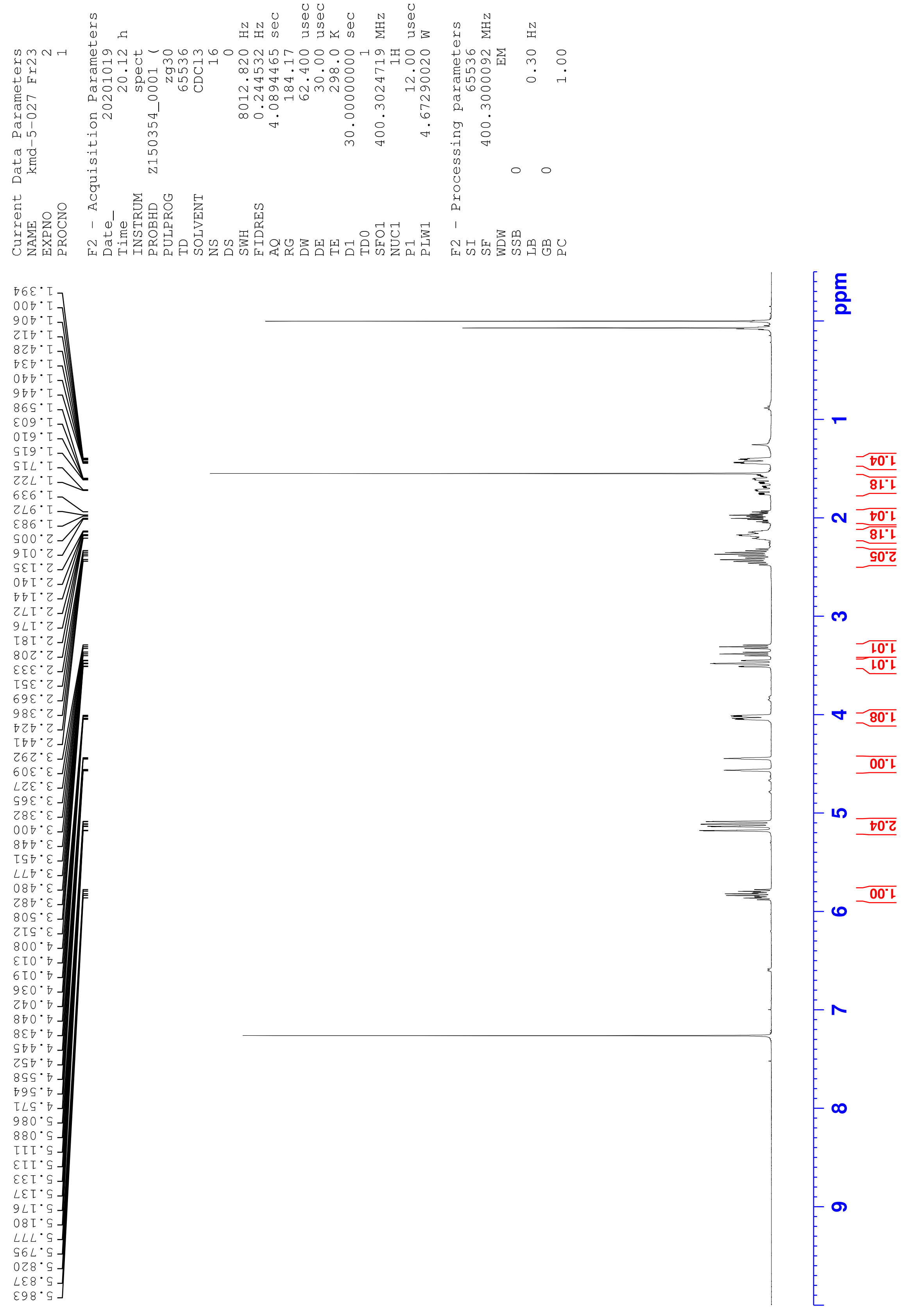
Current Data Parameters
NAME kmd-5-027 Fr23
EXPNO 2
PROCNO 1
F2 - Acquisition Parameters
Date_ 20201019
Time 20.12 h
INSTRUM spect
PROBHD Z150354_0001 (
PULPROG zg30
TD 65536
SOLVENT CDCl3
NS 16
DS 0
SWH 8012.820 Hz
FIDRES 0.244532 Hz
AQ 4.0894465 sec
RG 184.17
DW 62.400 usec
DE 30.00 usec
TE 298.0 K
D1 30.00000000 sec
TD0 1
SFO1 400.3024719 MHz
NUC1 1H
P1 12.00 usec
PLW1 4.67290020 W
F2 - Processing parameters
SI 65536
SF 400.3000092 MHz
WDW EM
SSB 0
LB 0.30 Hz
GB 0
PC 1.00
1.394
1.400
1.406
1.412
1.428
1.434
1.440
1.446
1.598
1.603
1.610
1.615
1.715
1.722
1.939
1.972
1.983
2.005
2.016
2.135
2.140
2.144
2.172
2.176
2.181
2.208
2.333
2.351
2.369
2.386
2.424
2.441
3.292
3.309
3.327
3.365
3.382
3.400
3.448
3.451
3.477
3.480
3.482
3.508
3.512
4.008
4.013
4.019
4.036
4.042
4.048
4.438
4.445
4.452
4.558
4.564
4.571
5.086
5.088
5.111
5.113
5.133
5.137
5.176
5.180
5.777
5.795
5.820
5.837
5.863
1.04
1.18
1.04
1.18
2.05
1.01
1.01
1.08
1.00
2.04
1.00
ppm
1
2
3
4
5
6
7
8
9

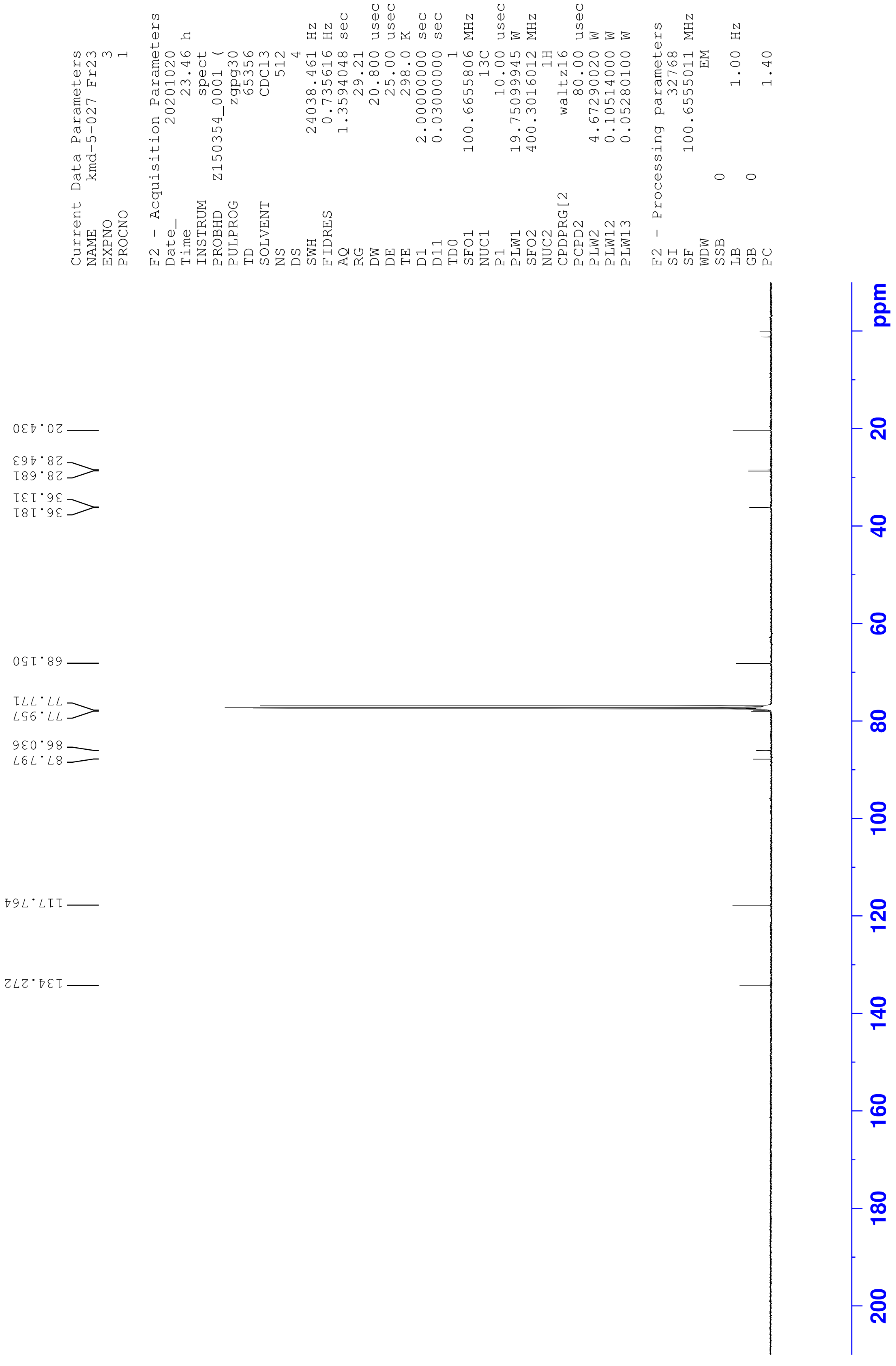
Current Data Parameters
NAME kmd-5-027 Fr23
EXPNO 3
PROCNO 1
F2 - Acquisition Parameters
Date_ 20201020
Time 23.46 h
INSTRUM spect
PROBHD Z150354_0001 (
PULPROG zgpg30
TD 65356
SOLVENT CDCl3
NS 512
DS 4
SWH 24038.461 Hz
FIDRES 0.735616 Hz
AQ 1.3594048 sec
RG 29.21
DW 20.800 usec
DE 25.00 usec
TE 298.0 K
D1 2.00000000 sec
D11 0.03000000 sec
TD0 1
SFO1 100.6655806 MHz
NUC1 13C
P1 10.00 usec
PLW1 19.75099945 W
SFO2 400.3016012 MHz
NUC2 1H
CPDPRG[2 waltz16
PCPD2 80.00 usec
PLW2 4.67290020 W
PLW12 0.10514000 W
PLW13 0.05280100 W
F2 - Processing parameters
SI 32768
SF 100.6555011 MHz
WDW EM
SSB 0
LB 1.00 Hz
GB 0
PC 1.40
20.430
28.463
28.681
36.131
36.181
68.150
77.771
77.957
86.036
87.797
117.764
134.272
ppm
20
40
60
80
100
120
140
160
180
200

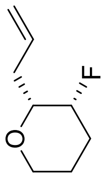
O
F

cis-**2a**

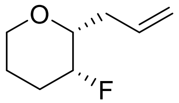

cis-**2a**

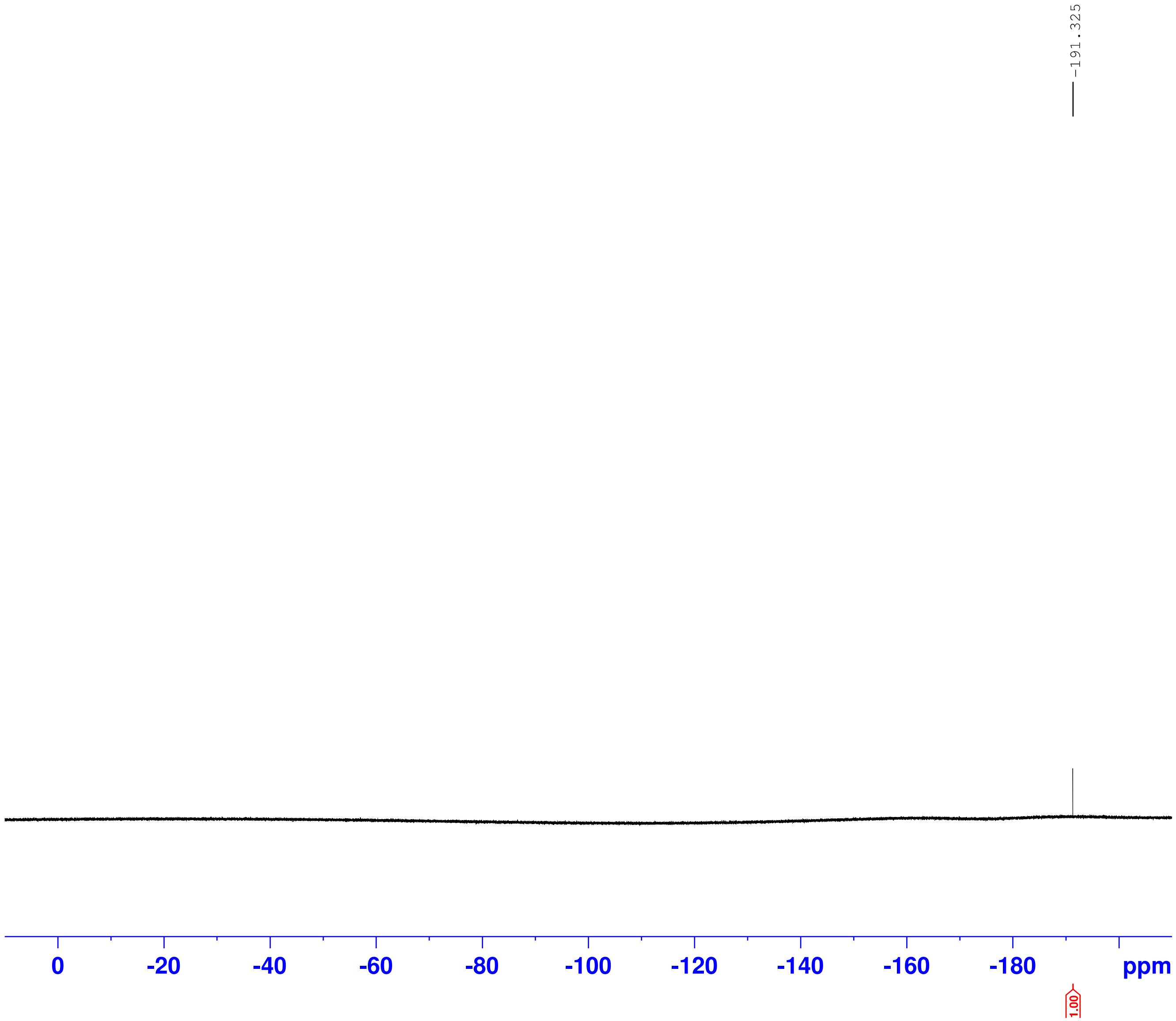


```
Current Data Parameters
NAME      kmd-5-027 Fr23 19F
EXPNO                  2
PROCNO                 1

F2 - Acquisition Parameters
Date_           20210105
Time               14.07 h
INSTRUM            spect
PROBHD    Z108618_0422 (
PULPROG       zgfhigqn.2
TD                131072
SOLVENT            CDCl3
NS                    16
DS                     4
SWH           89285.711 Hz
FIDRES         1.362392 Hz
AQ            0.7340032 sec
RG                200.67
DW                 5.600 usec
DE                  6.50 usec
TE                 301.9 K
D1            1.00000000 sec
D11           0.03000000 sec
D12           0.00002000 sec
TD0                    1
SFO1         376.5453925 MHz
NUC1                 19F
P1                 15.00 usec
PLW1         16.89999962 W
SFO2         400.2216009 MHz
NUC2                  1H
CPDPRG[2         waltz16
PCPD2              90.00 usec
PLW2         14.69999981 W
PLW12         0.40832999 W

F2 - Processing parameters
SI                 65536
SF           376.5834187 MHz
WDW                   EM
SSB      0
LB                  0.30 Hz
GB       0
PC                  1.00
```

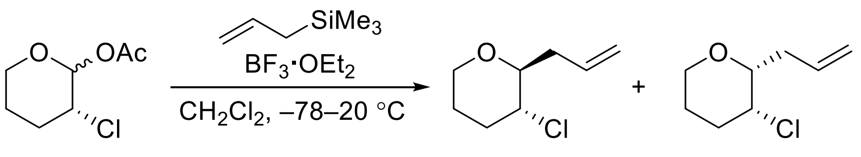

1b *trans*-**2b** *cis*-**2b**

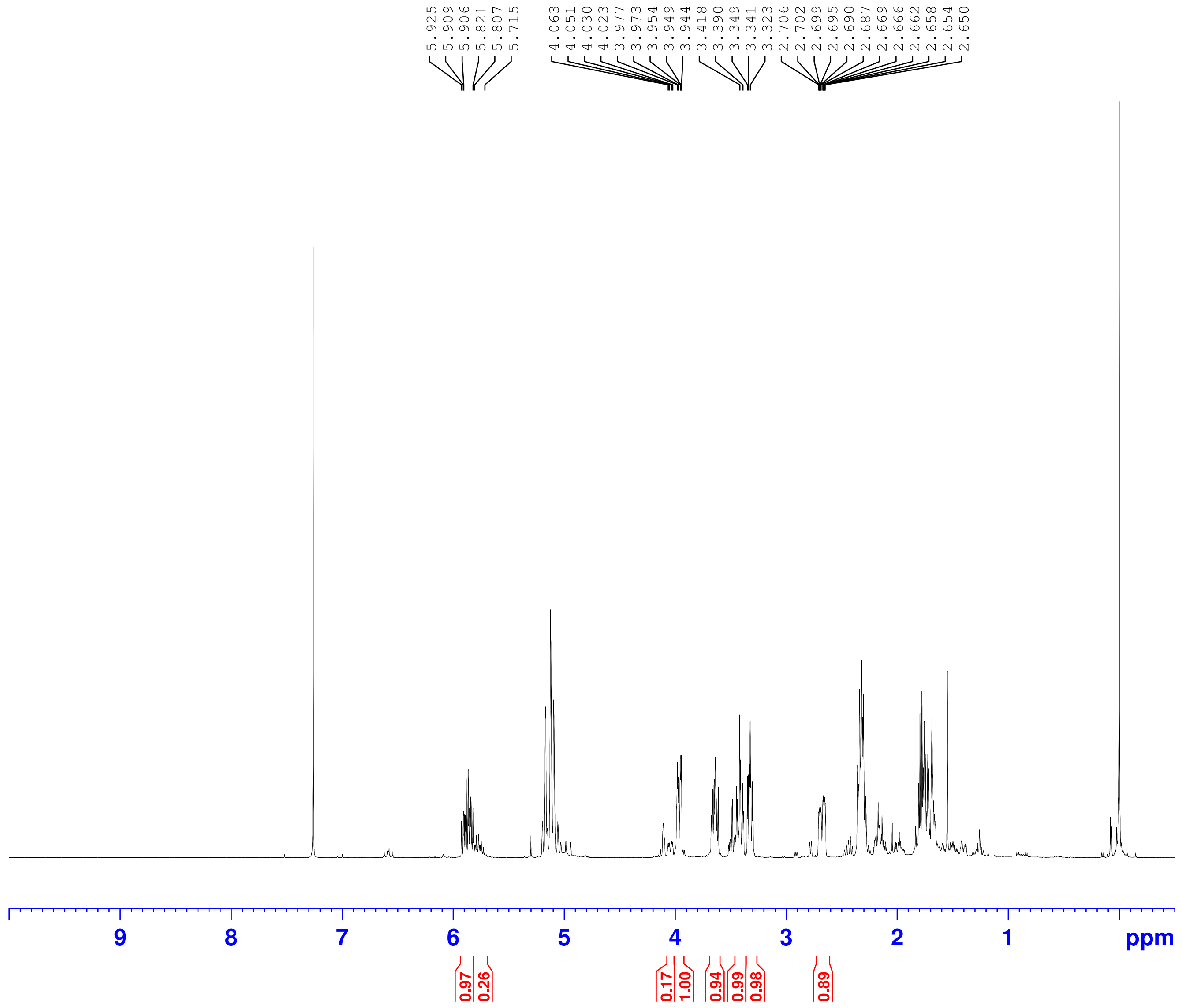


```
Current Data Parameters
NAME             kmd-3-174
EXPNO                    2
PROCNO                   1

F2 - Acquisition Parameters
Date_             20191112
Time                 11.57 h
INSTRUM              spect
PROBHD   Z150354_0001 (
PULPROG               zg30
TD                   65536
SOLVENT              CDCl3
NS                       4
DS                       0
SWH               8012.820 Hz
FIDRES            0.244532 Hz
AQ               4.0894465 sec
RG                    92.4
DW                  62.400 usec
DE                   30.00 usec
TE                   298.0 K
D1             30.00000000 sec
TD0                      1
SFO1           400.3024719 MHz
NUC1                    1H
P1                   12.00 usec
PLW1            4.67290020 W

F2 - Processing parameters
SI                   65536
SF             400.3000094 MHz
WDW                     EM
SSB      0
LB                    0.30 Hz
GB       0
PC                    1.00
```

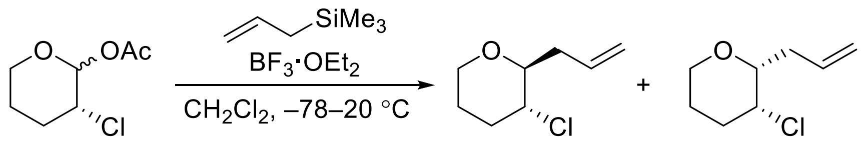

1b *trans*-**2b** *cis*-**2b**

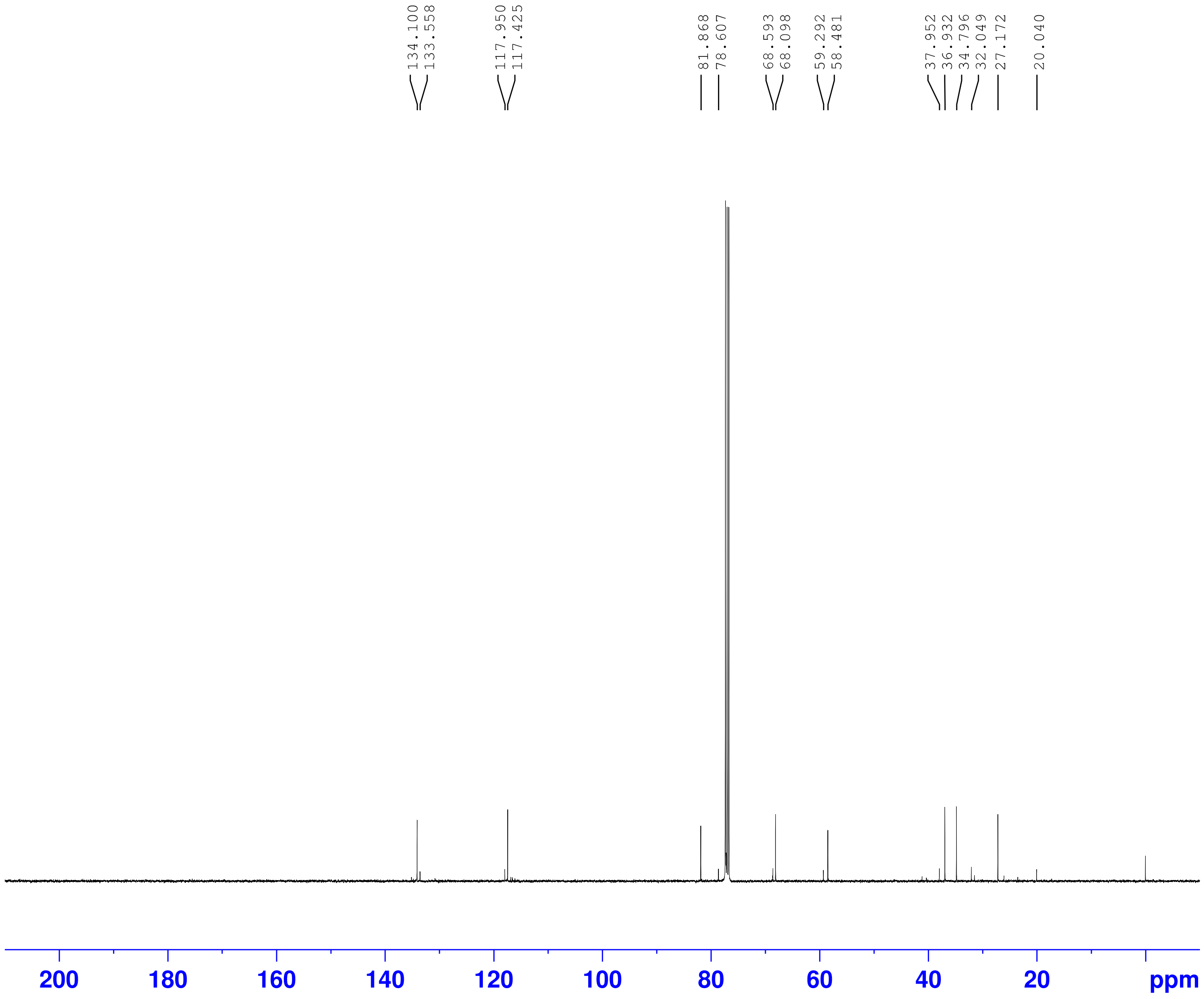


```
Current Data Parameters
NAME              kmd-3-174
EXPNO                     3
PROCNO                    1

F2 - Acquisition Parameters
Date_              20191112
Time                  13.07 h
INSTRUM               spect
PROBHD    Z150354_0001 (
PULPROG             zgpg30
TD                    65356
SOLVENT               CDCl3
NS                      256
DS                        4
SWH               24038.461 Hz
FIDRES             0.735616 Hz
AQ                1.3594048 sec
RG                     31.4
DW                   20.800 usec
DE                    25.00 usec
TE                    298.0 K
D1               2.00000000 sec
D11              0.03000000 sec
TD0                       1
SFO1           100.6655806 MHz
NUC1                    13C
P1                    10.00 usec
PLW1            19.75099945 W
SFO2           400.3016012 MHz
NUC2                     1H
CPDPRG[2            waltz16
PCPD2                 80.00 usec
PLW2             4.67290020 W
PLW12            0.10514000 W
PLW13            0.05280100 W

F2 - Processing parameters
SI                    32768
SF             100.6555157 MHz
WDW                      EM
SSB       0
LB                     1.00 Hz
GB        0
PC                     1.40
```

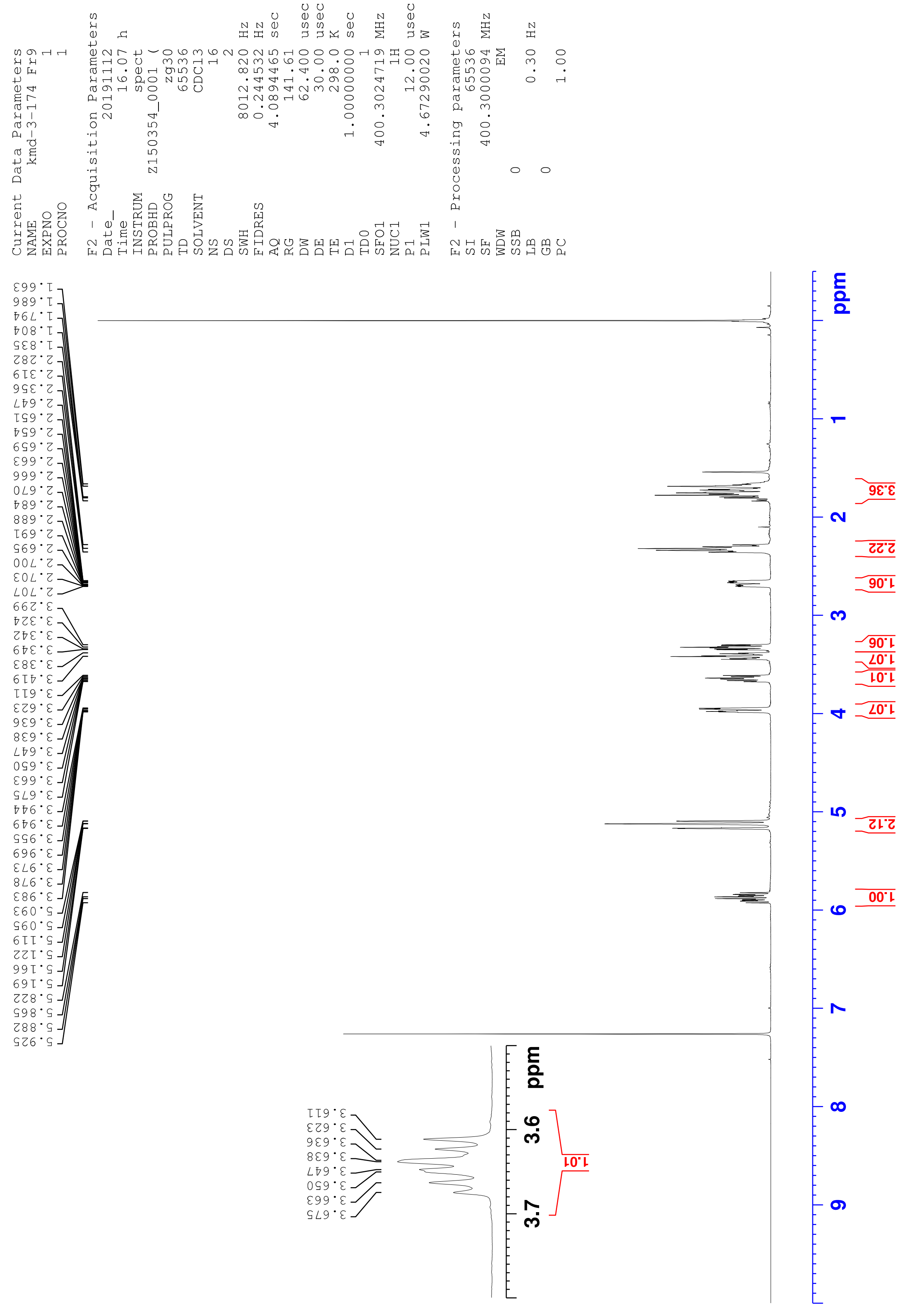
Current Data Parameters
NAME kmd-3-174 Fr9
EXPNO 1
PROCNO 1
F2 - Acquisition Parameters
Date_ 20191112
Time 16.07 h
INSTRUM spect
PROBHD Z150354_0001 (
PULPROG zg30
TD 65536
SOLVENT CDCl3
NS 16
DS 2
SWH 8012.820 Hz
FIDRES 0.244532 Hz
AQ 4.0894465 sec
RG 141.61
DW 62.400 usec
DE 30.00 usec
TE 298.0 K
D1 1.00000000 sec
TD0 1
SFO1 400.3024719 MHz
NUC1 1H
P1 12.00 usec
PLW1 4.67290020 W
F2 - Processing parameters
SI 65536
SF 400.3000094 MHz
WDW EM
SSB 0
LB 0.30 Hz
GB 0
PC 1.00
1.663 1.686 1.794 1.804 1.835 2.282 2.319 2.356 2.647 2.651 2.654 2.659 2.663 2.666 2.670 2.684 2.688 2.691 2.695 2.700 2.703 2.707 3.299 3.324 3.342 3.349 3.383 3.419 3.611 3.623 3.636 3.638 3.647 3.650 3.663 3.675 3.944 3.949 3.955 3.969 3.973 3.978 3.983 5.093 5.095 5.119 5.122 5.166 5.169 5.822 5.865 5.882 5.925
3.36 2.22 1.06 1.06 1.07 1.01 1.07 2.12 1.00
ppm
1 2 3 4 5 6 7 8 9
3.611 3.623 3.636 3.638 3.647 3.650 3.663 3.675
1.01
3.6 3.7 ppm

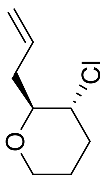

trans-**2b**

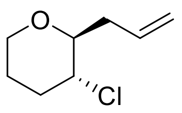

trans-**2b**

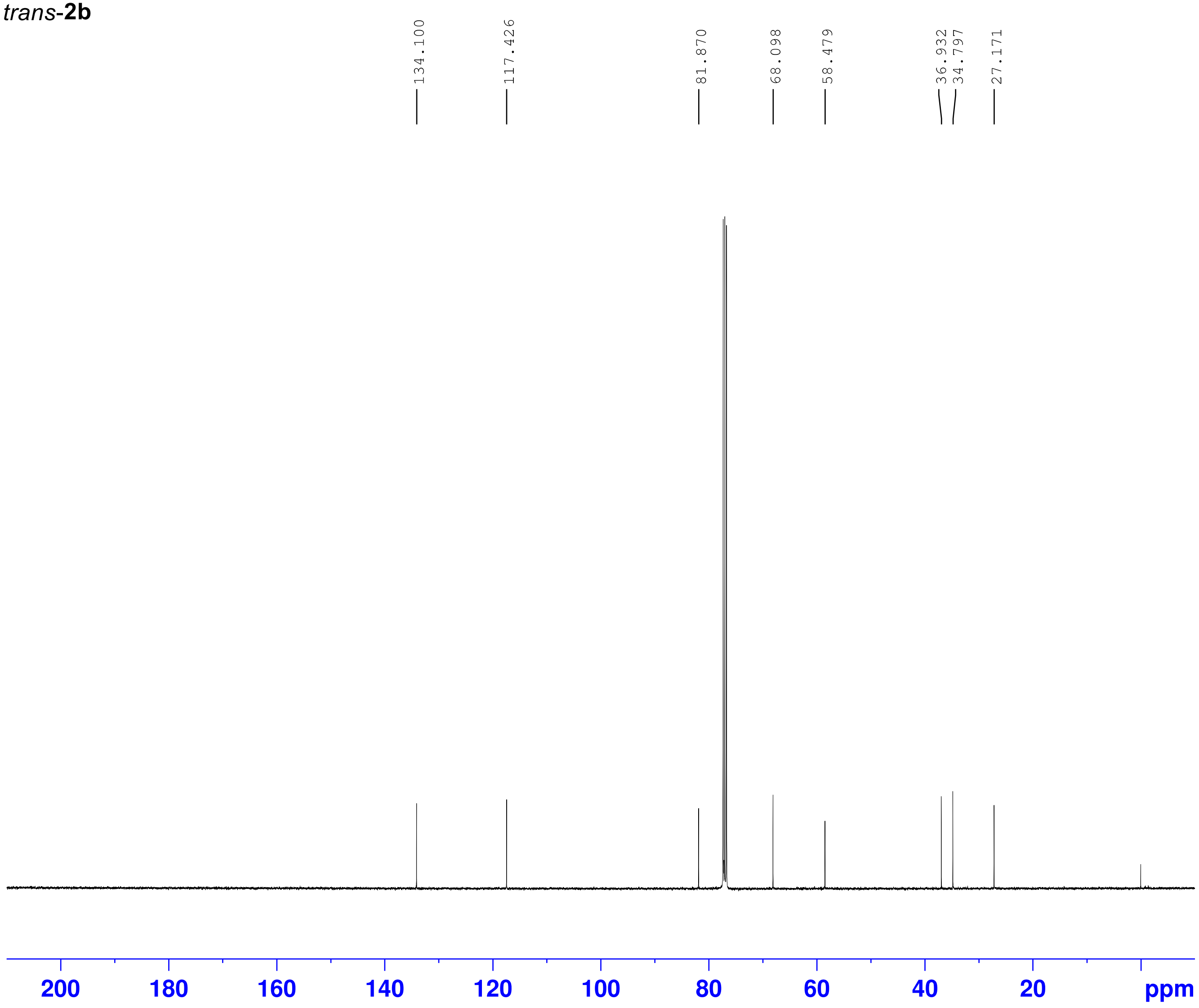

Current Data Parameters

NAME	kmd-3-041 Fr24-29
EXPNO	2
PROCNO	1

F2 - Acquisition Parameters

Date_	20190730
Time	20.20 h
INSTRUM	spect
PROBHD	Z150354_0001 (
PULPROG	zgpg30
TD	65356
SOLVENT	CDCl3
NS	256
DS	4
SWH	24038.461 Hz
FIDRES	0.735616 Hz
AQ	1.3594048 sec
RG	29.21
DW	20.800 usec
DE	25.00 usec
TE	298.0 K
D1	2.00000000 sec
D11	0.03000000 sec
TD0	1
SFO1	100.6655806 MHz
NUC1	13C
P1	10.00 usec
PLW1	19.75099945 W
SFO2	400.3016012 MHz
NUC2	1H
CPDPRG[2	waltz16
PCPD2	80.00 usec
PLW2	4.67290020 W
PLW12	0.10514000 W
PLW13	0.05280100 W

F2 - Processing parameters

SI		32768
SF		100.6555157 MHz
WDW		EM
SSB	0	
LB		1.00 Hz
GB	0	
PC		1.40

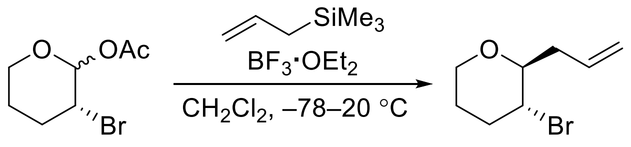

1c **2c**

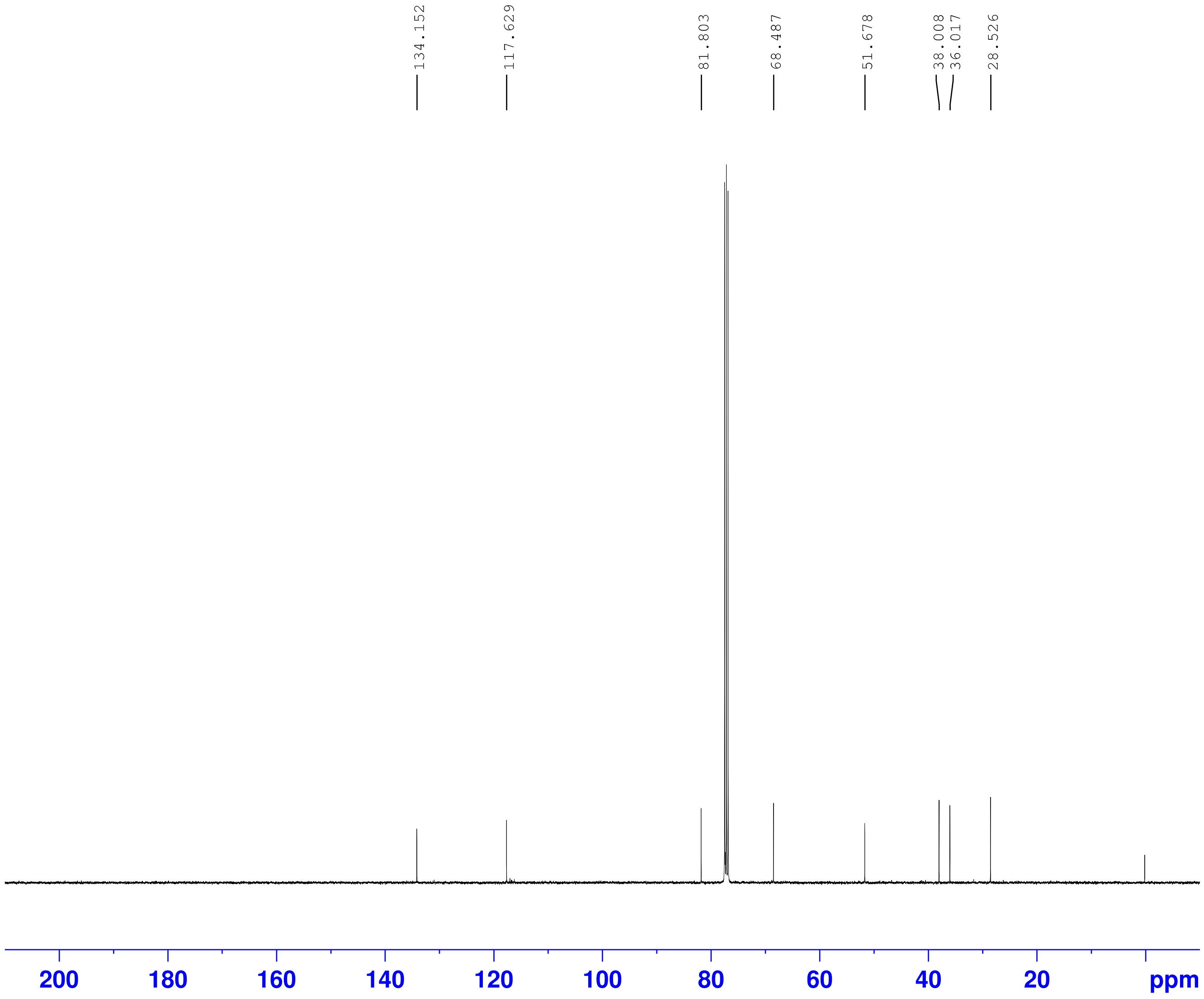


```
Current Data Parameters
NAME             kmd-3-176
EXPNO                    3
PROCNO                   1

F2 - Acquisition Parameters
Date_             20191113
Time                 20.12 h
INSTRUM              spect
PROBHD    Z150354_0001 (
PULPROG            zgpg30
TD                  65356
SOLVENT             CDCl3
NS                    256
DS                      4
SWH             24038.461 Hz
FIDRES           0.735616 Hz
AQ              1.3594048 sec
RG                   31.4
DW                 20.800 usec
DE                  25.00 usec
TE                  298.0 K
D1             2.00000000 sec
D11            0.03000000 sec
TD0                     1
SFO1          100.6655806 MHz
NUC1                  13C
P1                  10.00 usec
PLW1          19.75099945 W
SFO2          400.3016012 MHz
NUC2                   1H
CPDPRG[2          waltz16
PCPD2               80.00 usec
PLW2           4.67290020 W
PLW12          0.10514000 W
PLW13          0.05280100 W

F2 - Processing parameters
SI                  32768
SF            100.6555014 MHz
WDW                    EM
SSB       0
LB                   1.00 Hz
GB        0
PC                   1.40
```

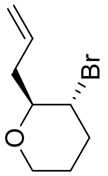

2c

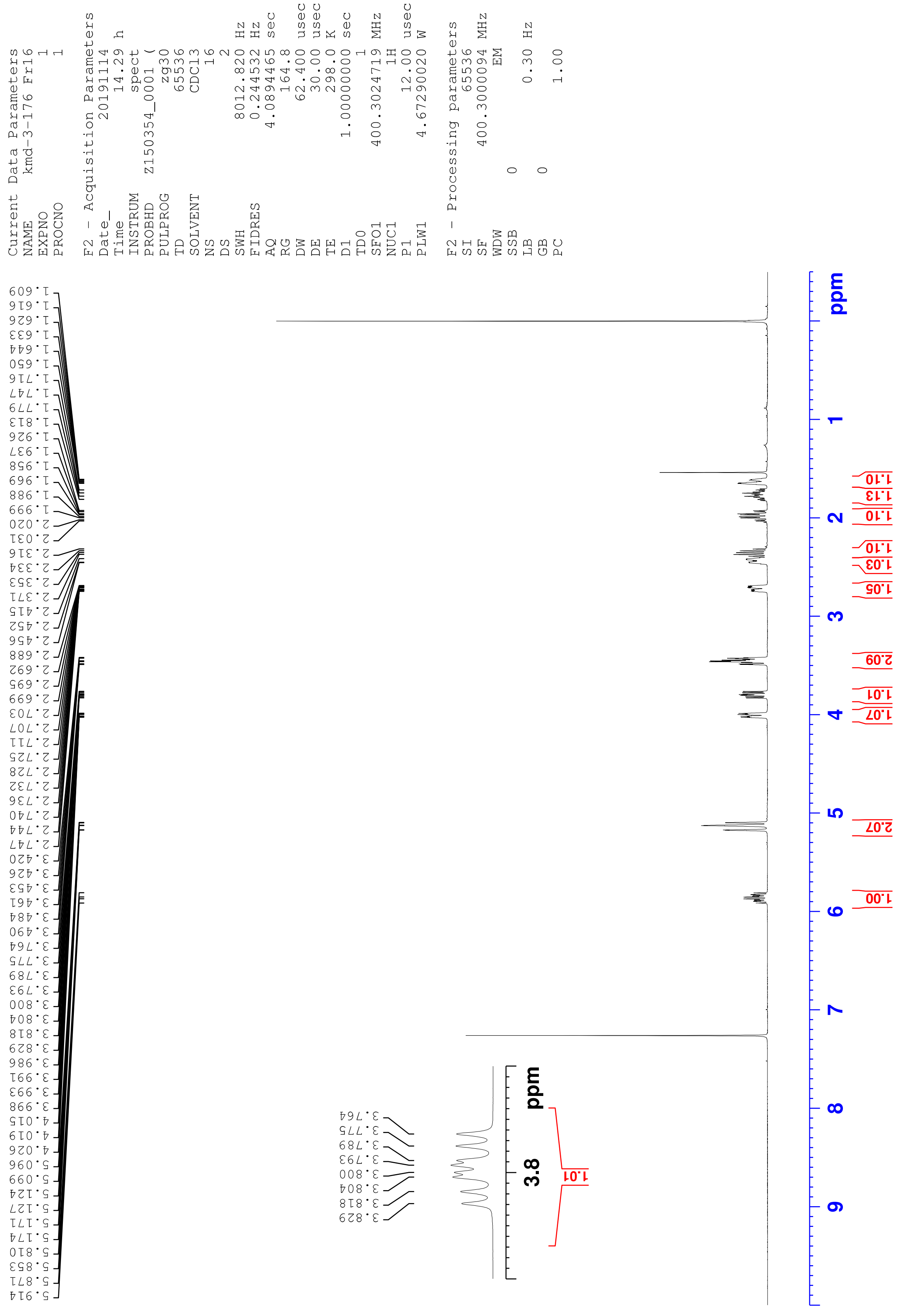
Current Data Parameters
NAME kmd-3-176 Fr16
EXPNO 1
PROCNO 1
F2 - Acquisition Parameters
Date_ 20191114
Time 14.29 h
INSTRUM spect
PROBHD Z150354_0001 (
PULPROG zg30
TD 65536
SOLVENT CDCl3
NS 16
DS 2
SWH 8012.820 Hz
FIDRES 0.244532 Hz
AQ 4.0894465 sec
RG 164.8
DW 62.400 usec
DE 30.00 usec
TE 298.0 K
D1 1.00000000 sec
TD0 1
SFO1 400.3024719 MHz
NUC1 1H
P1 12.00 usec
PLW1 4.67290020 W
F2 - Processing parameters
SI 65536
SF 400.3000094 MHz
WDW EM
SSB 0
LB 0.30 Hz
GB 0
PC 1.00
1.609 1.616 1.626 1.633 1.644 1.650 1.716 1.747 1.779 1.813 1.926 1.937 1.958 1.969 1.988 1.999 2.020 2.031 2.316 2.334 2.353 2.371 2.415 2.452 2.456 2.688 2.692 2.695 2.699 2.703 2.707 2.711 2.725 2.728 2.732 2.736 2.740 2.744 2.747 3.420 3.426 3.453 3.461 3.484 3.490 3.764 3.775 3.789 3.793 3.800 3.804 3.818 3.829 3.986 3.991 3.993 3.998 4.015 4.019 4.026 5.096 5.099 5.124 5.127 5.171 5.174 5.810 5.853 5.871 5.914
3.764 3.775 3.789 3.793 3.800 3.804 3.818 3.829
3.8
ppm
1.01
1.10 1.13 1.10 1.10 1.03 1.05 2.09 1.01 1.07 2.07 1.00
ppm
1 2 3 4 5 6 7 8 9

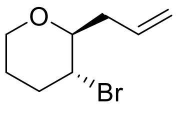

2c

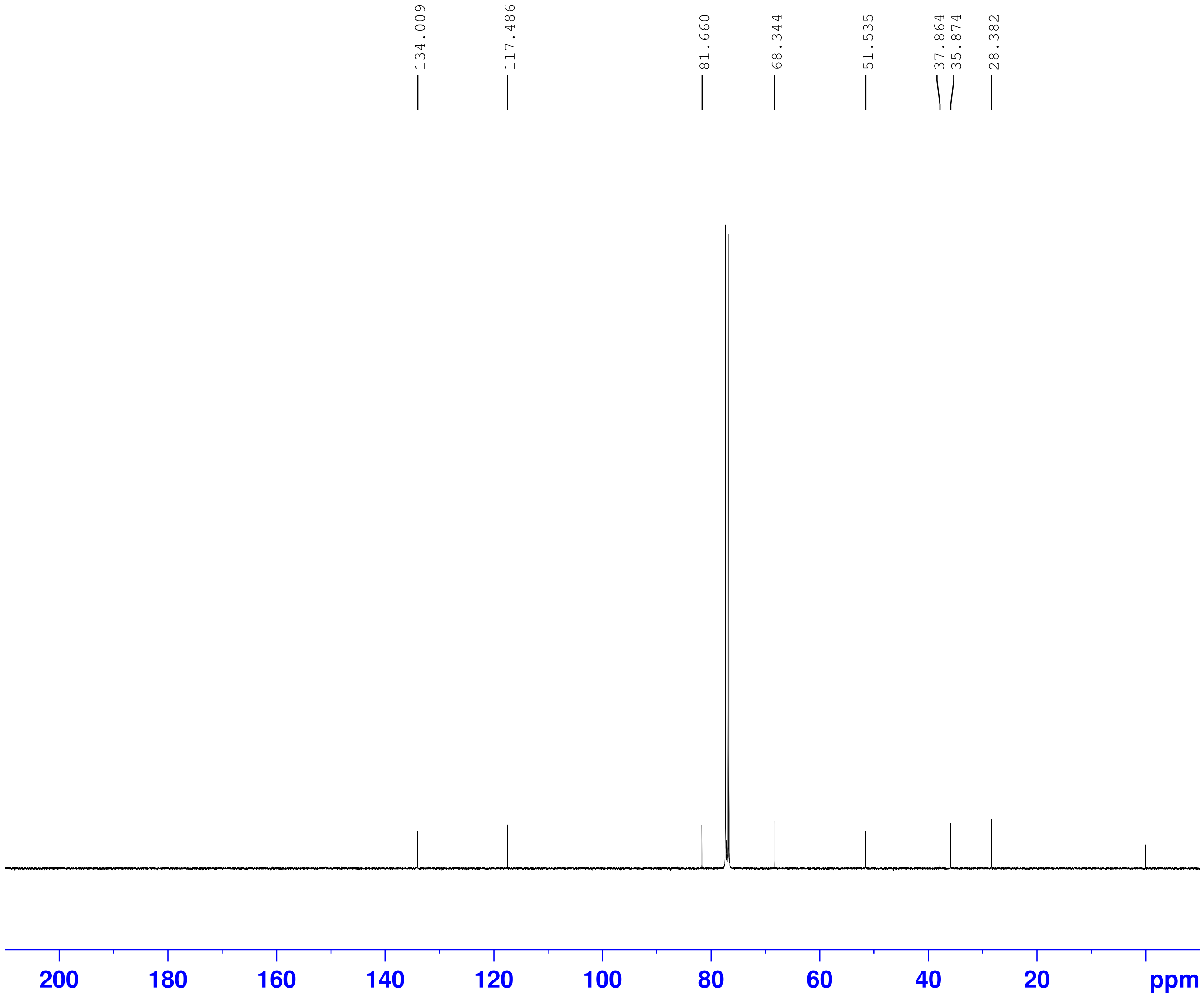


```
Current Data Parameters
NAME       kmd-3-176 Fr16
EXPNO                   2
PROCNO                  1

F2 - Acquisition Parameters
Date_            20191114
Time                15.03 h
INSTRUM             spect
PROBHD    Z150354_0001 (
PULPROG           zgpg30
TD                 65356
SOLVENT            CDCl3
NS                   256
DS                     4
SWH            24038.461 Hz
FIDRES          0.735616 Hz
AQ             1.3594048 sec
RG                 39.47
DW                20.800 usec
DE                 25.00 usec
TE                 298.0 K
D1            2.00000000 sec
D11           0.03000000 sec
TD0                    1
SFO1         100.6655806 MHz
NUC1                 13C
P1                 10.00 usec
PLW1         19.75099945 W
SFO2         400.3016012 MHz
NUC2                  1H
CPDPRG[2         waltz16
PCPD2              80.00 usec
PLW2          4.67290020 W
PLW12         0.10514000 W
PLW13         0.05280100 W

F2 - Processing parameters
SI                 32768
SF           100.6555158 MHz
WDW                   EM
SSB      0
LB                  1.00 Hz
GB       0
PC                  1.40
```

WILEY-VCH

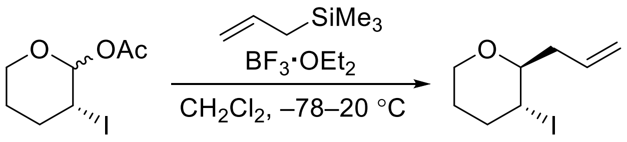

1d 2d

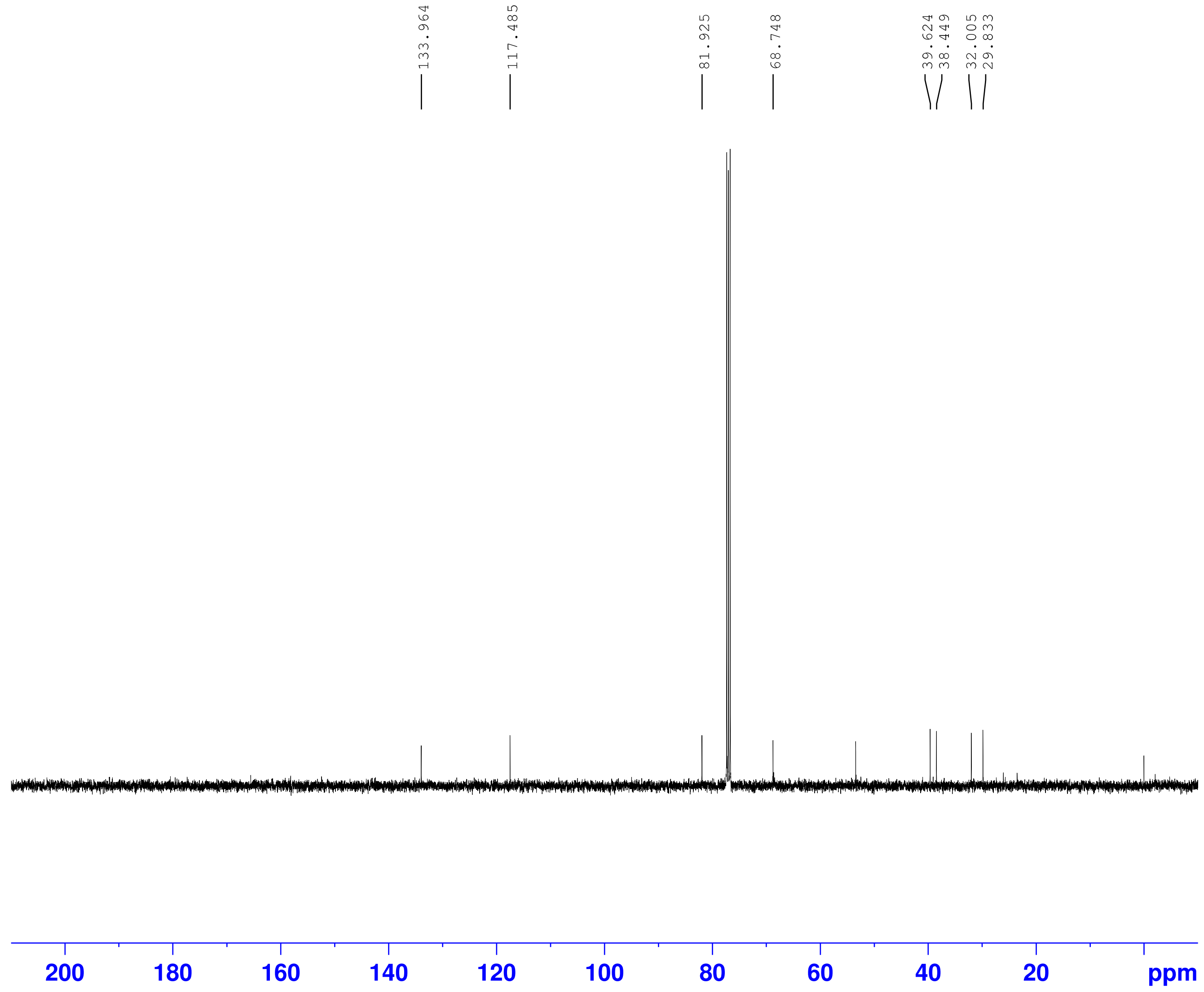


```
Current Data Parameters
NAME             kmd-5-132
EXPNO                    3
PROCNO                   1

F2 - Acquisition Parameters
Date_             20210125
Time                 10.06 h
INSTRUM              spect
PROBHD   Z108618_0422 (
PULPROG            zgpg30
TD                  65536
SOLVENT             CDCl3
NS                    256
DS                      4
SWH             24038.461 Hz
FIDRES           0.733596 Hz
AQ              1.3631488 sec
RG                 200.67
DW                 20.800 usec
DE                   6.50 usec
TE                  299.4 K
D1             2.00000000 sec
D11            0.03000000 sec
TD0                     1
SFO1         100.6454626 MHz
NUC1                  13C
P1                  10.00 usec
PLW1          57.00000000 W
SFO2         400.2216009 MHz
NUC2                   1H
CPDPRG[2          waltz16
PCPD2               90.00 usec
PLW2          14.69999981 W
PLW12          0.40832999 W
PLW13          0.20539001 W

F2 - Processing parameters
SI                  32768
SF            100.6353990 MHz
WDW                    EM
SSB      0
LB                   1.00 Hz
GB       0
PC                   1.40
```

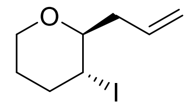

2d

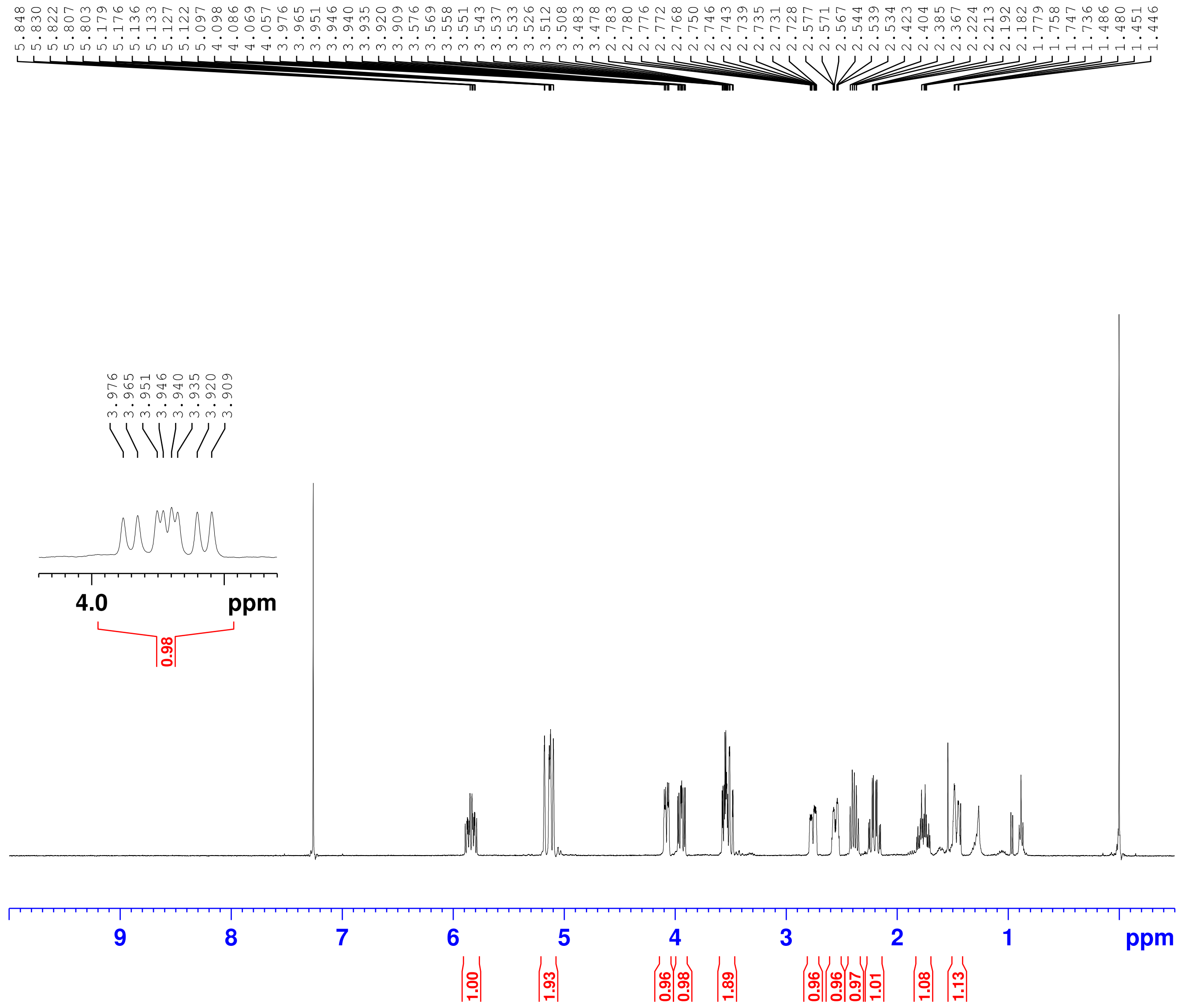

Current Data Parameters
NAME kmd-5-132 Fr12-14
EXPNO 2
PROCNO 1

F2 - Acquisition Parameters
Date_ 20210125
Time 11.36 h
INSTRUM spect
PROBHD Z108618_0422 (
PULPROG zg30
TD 65536
SOLVENT CDCl3
NS 4
DS 0
SWH 5597.015 Hz
FIDRES 0.170807 Hz
AQ 5.8545494 sec
RG 139.37
DW 89.333 usec
DE 6.50 usec
TE 298.9 K
D1 30.00000000 sec
TD0 1
SFO1 400.2224013 MHz
NUC1 1H
P1 15.00 usec
PLW1 14.25000000 W

F2 - Processing parameters
SI 65536
SF 400.2200099 MHz
WDW EM
SSB 0
LB 0.30 Hz
GB 0
PC 1.00

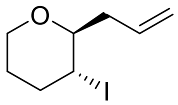

2d

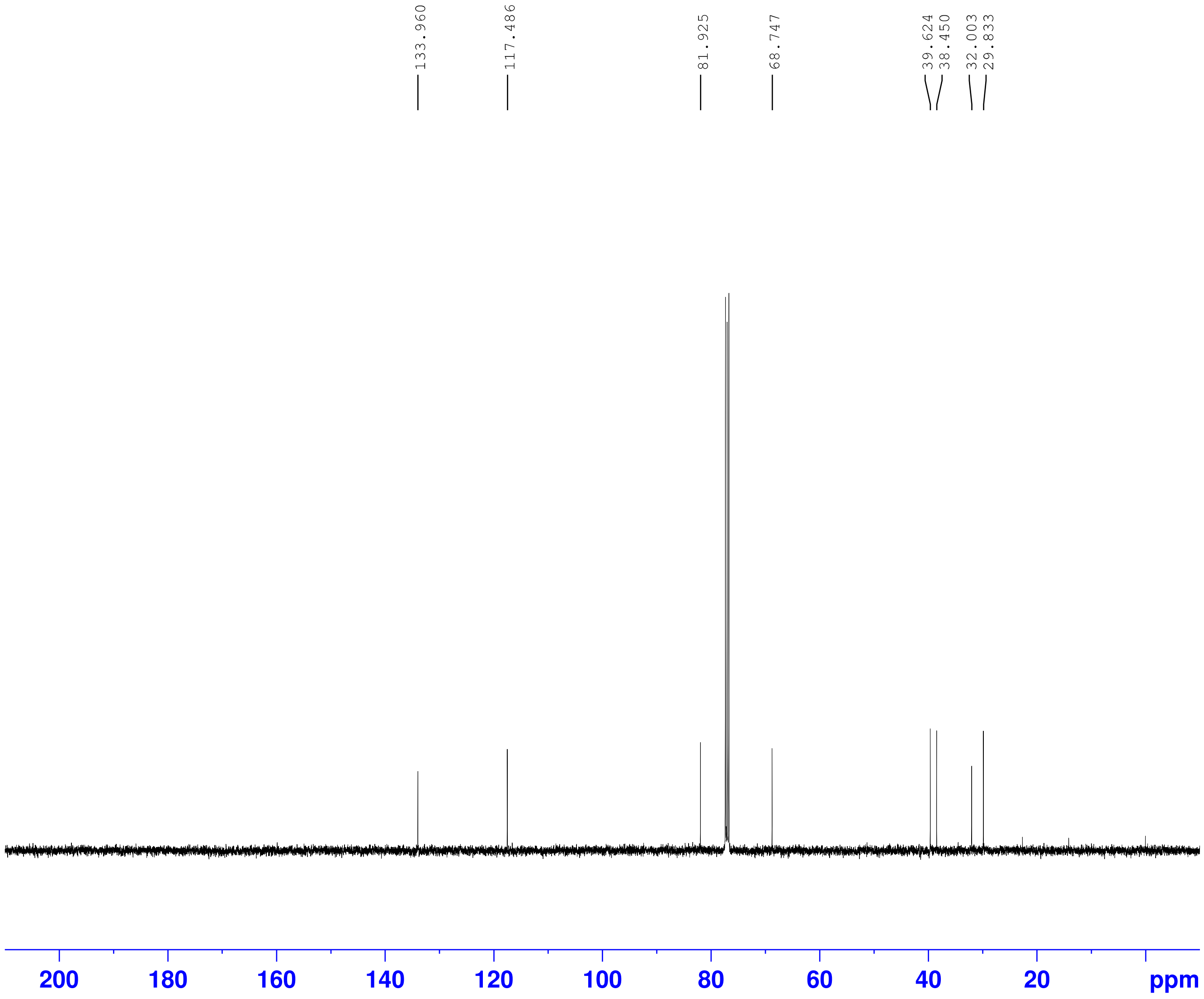


```
Current Data Parameters
NAME      kmd-5-132 Fr12-14
EXPNO                     3
PROCNO                    1

F2 - Acquisition Parameters
Date_              20210125
Time                  11.52 h
INSTRUM               spect
PROBHD      Z108618_0422 (
PULPROG             zgpg30
TD                   65536
SOLVENT              CDCl3
NS                     256
DS                       4
SWH              24038.461 Hz
FIDRES            0.733596 Hz
AQ               1.3631488 sec
RG                  200.67
DW                  20.800 usec
DE                    6.50 usec
TE                   299.6 K
D1              2.00000000 sec
D11             0.03000000 sec
TD0                      1
SFO1          100.6454626 MHz
NUC1                   13C
P1                   10.00 usec
PLW1           57.00000000 W
SFO2          400.2216009 MHz
NUC2                    1H
CPDPRG[2           waltz16
PCPD2                90.00 usec
PLW2           14.69999981 W
PLW12           0.40832999 W
PLW13           0.20539001 W

F2 - Processing parameters
SI                   32768
SF             100.6353990 MHz
WDW                     EM
SSB        0
LB                    1.00 Hz
GB         0
PC                    1.40
```

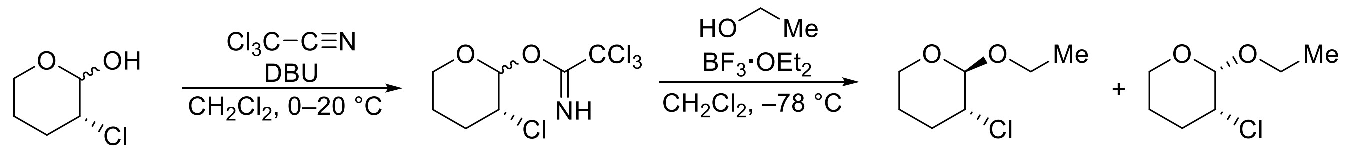

3 **5a** *trans*-**7a** *cis*-**7a**

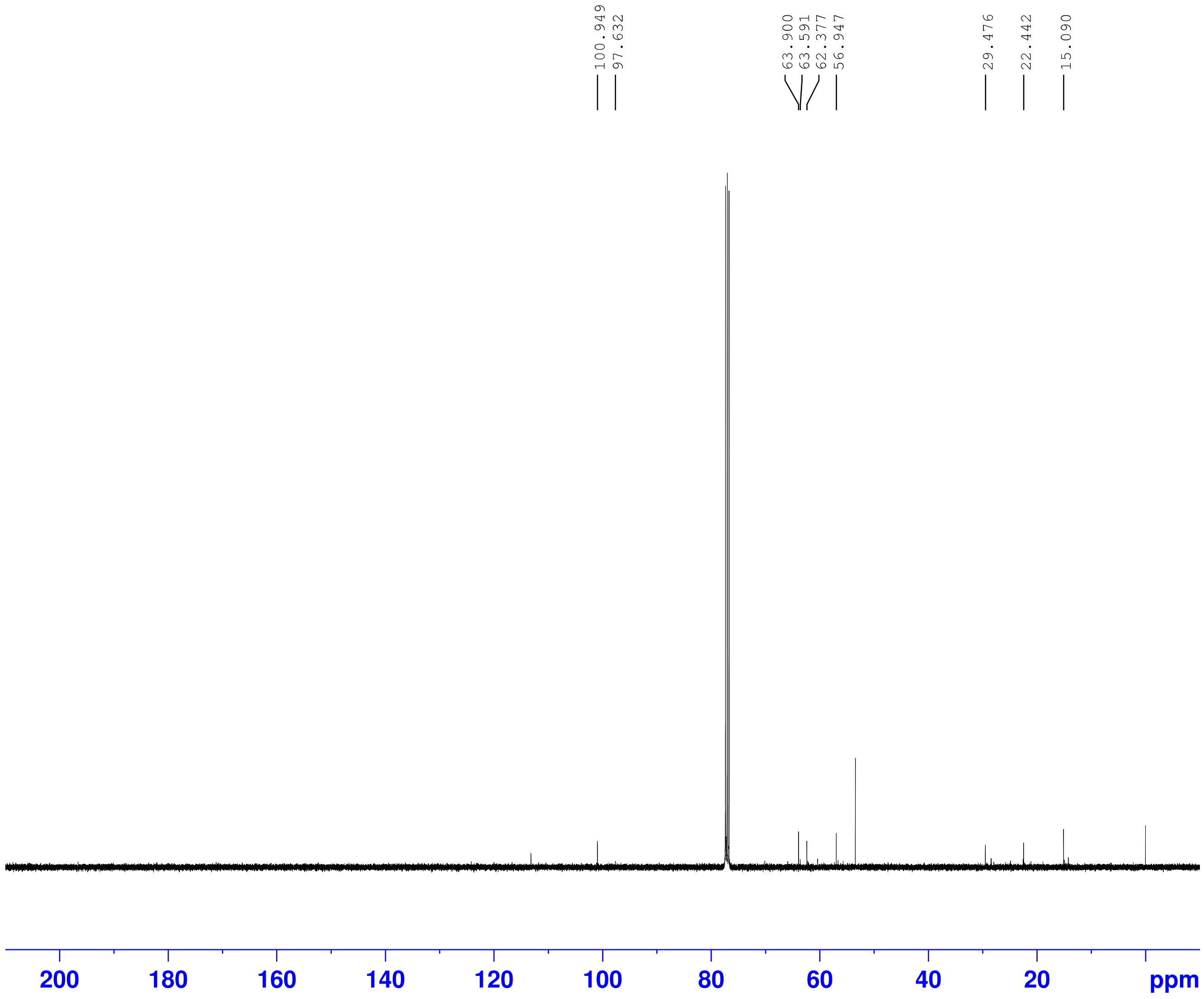


```
Current Data Parameters
NAME              kmd-5-178
EXPNO                     3
PROCNO                    1

F2 - Acquisition Parameters
Date_              20210316
Time                  17.05 h
INSTRUM               spect
PROBHD    Z150354_0001 (
PULPROG             zgpg30
TD                   65356
SOLVENT              CDCl3
NS                     256
DS                       4
SWH             24038.461 Hz
FIDRES           0.735616 Hz
AQ              1.3594048 sec
RG                   36.68
DW                  20.800 usec
DE                   25.00 usec
TE                   298.0 K
D1             2.00000000 sec
D11            0.03000000 sec
TD0                      1
SFO1          100.6655806 MHz
NUC1                   13C
P1                   10.00 usec
PLW1          19.75099945 W
SFO2          400.3016012 MHz
NUC2                    1H
CPDPRG[2           waltz16
PCPD2                80.00 usec
PLW2           4.67290020 W
PLW12          0.10514000 W
PLW13          0.05280100 W

F2 - Processing parameters
SI                  131072
SF            100.6555151 MHz
WDW                     EM
SSB       0
LB        0 Hz
GB        0
PC                    1.40
```

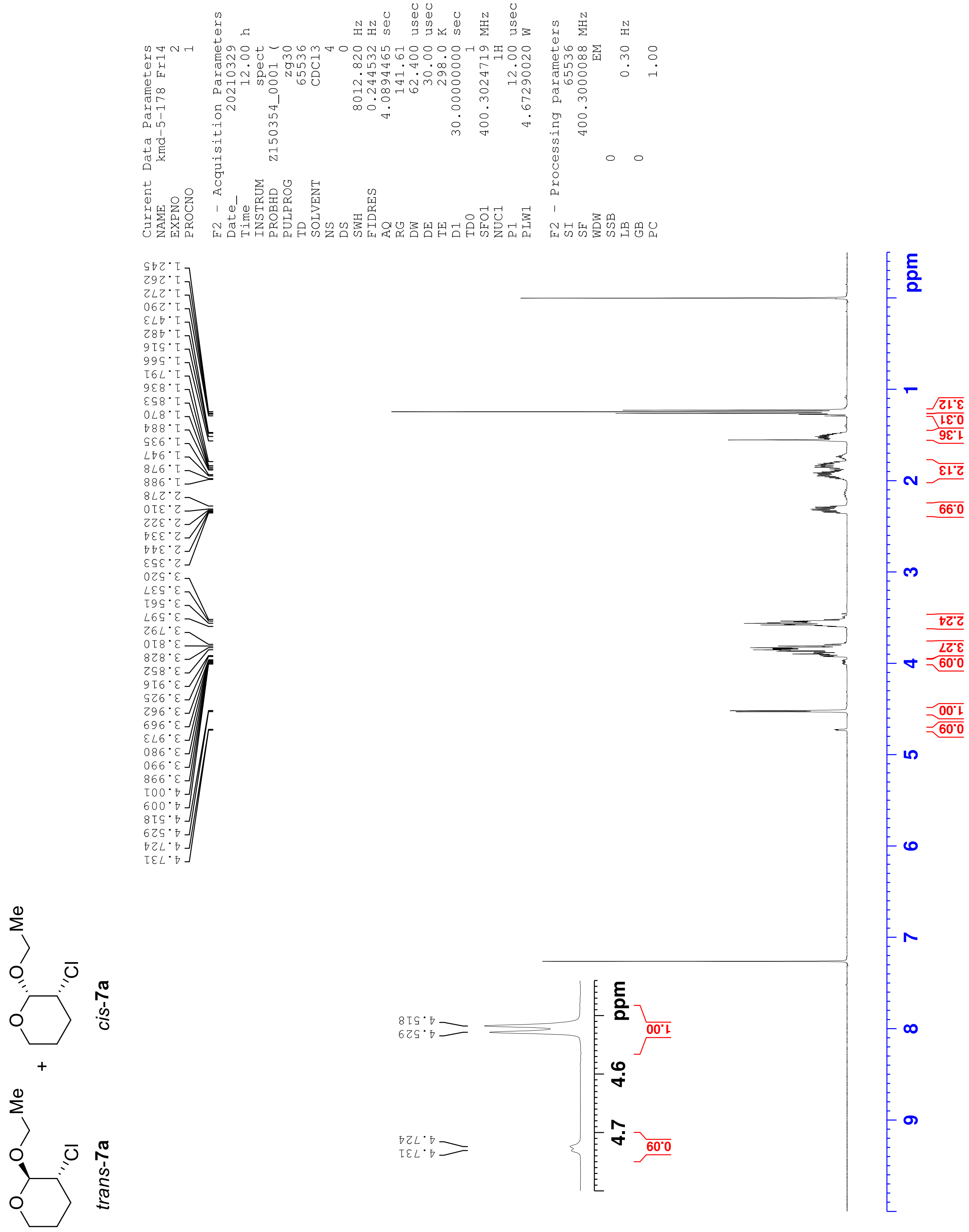
trans-7a
cis-7a
Me
Cl
+
Current Data Parameters
NAME kmd-5-178 Fr14
EXPNO 2
PROCNO 1
F2 - Acquisition Parameters
Date_ 20210329
Time 12.00 h
INSTRUM spect
PROBHD Z150354_0001 (
PULPROG zg30
TD 65536
SOLVENT CDCl3
NS 4
DS 0
SWH 8012.820 Hz
FIDRES 0.244532 Hz
AQ 4.0894465 sec
RG 141.61
DW 62.400 usec
DE 30.00 usec
TE 298.0 K
D1 30.00000000 sec
TD0 1
SFO1 400.3024719 MHz
NUC1 1H
P1 12.00 usec
PLW1 4.67290020 W
F2 - Processing parameters
SI 65536
SF 400.3000088 MHz
WDW EM
SSB 0
LB 0.30 Hz
GB 0
PC 1.00
1.245 1.262 1.272 1.290 1.473 1.482 1.516 1.566 1.791 1.836 1.853 1.870 1.884 1.935 1.947 1.978 1.988 2.278 2.310 2.322 2.334 2.344 2.353 3.520 3.537 3.561 3.597 3.792 3.810 3.828 3.852 3.916 3.925 3.962 3.969 3.973 3.980 3.990 3.998 4.001 4.009 4.518 4.529 4.724 4.731
3.12 0.31 1.36 2.13 0.99 2.24 3.27 0.09 1.00 0.09
4.7 4.6 ppm
ppm

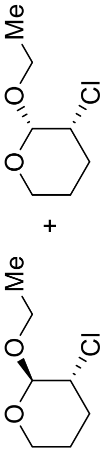

trans-7a + *cis*-7a

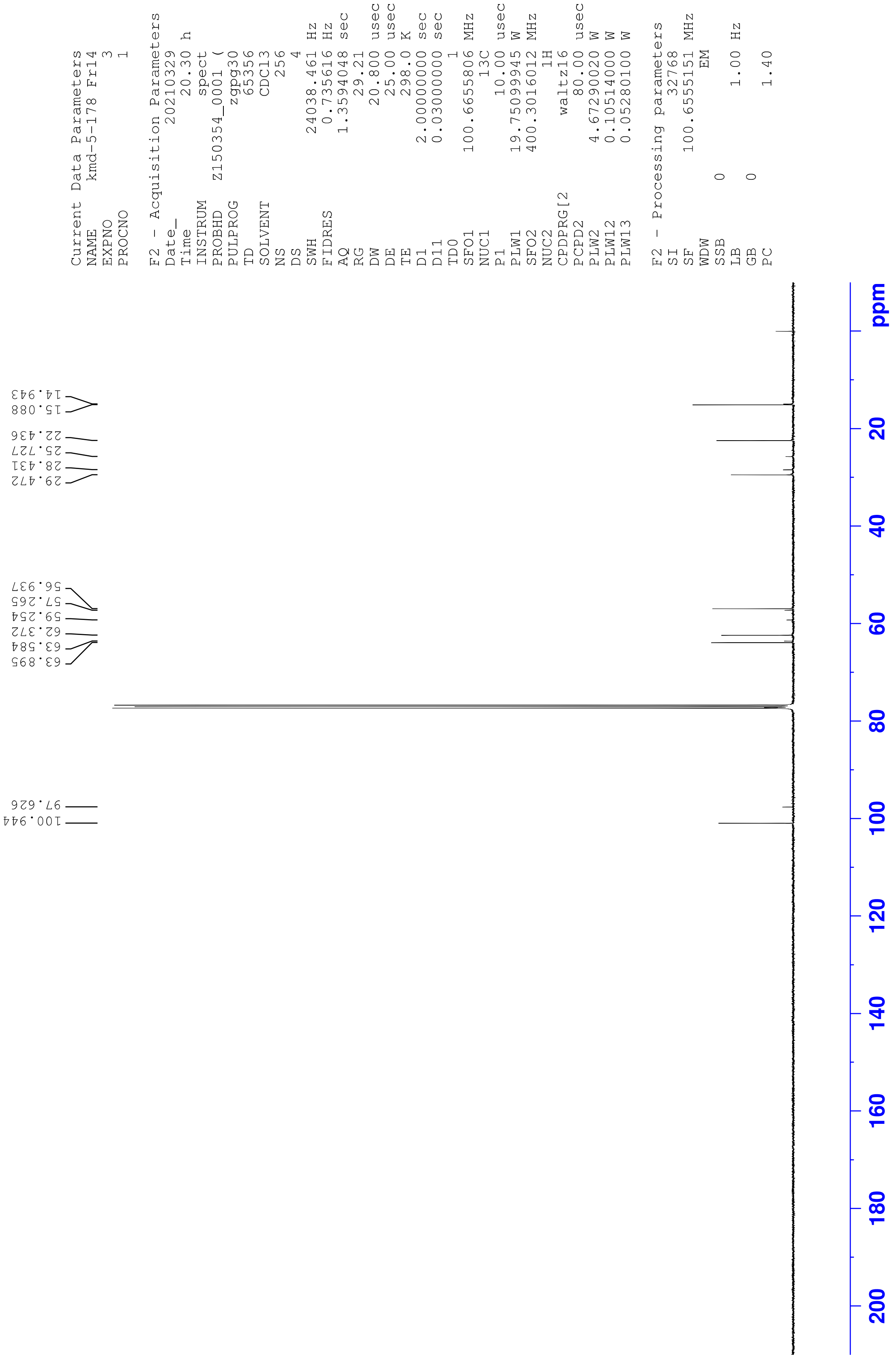

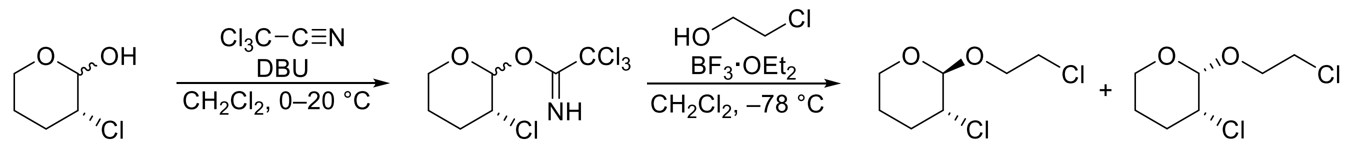

3 5a *trans*-7b *cis*-7b

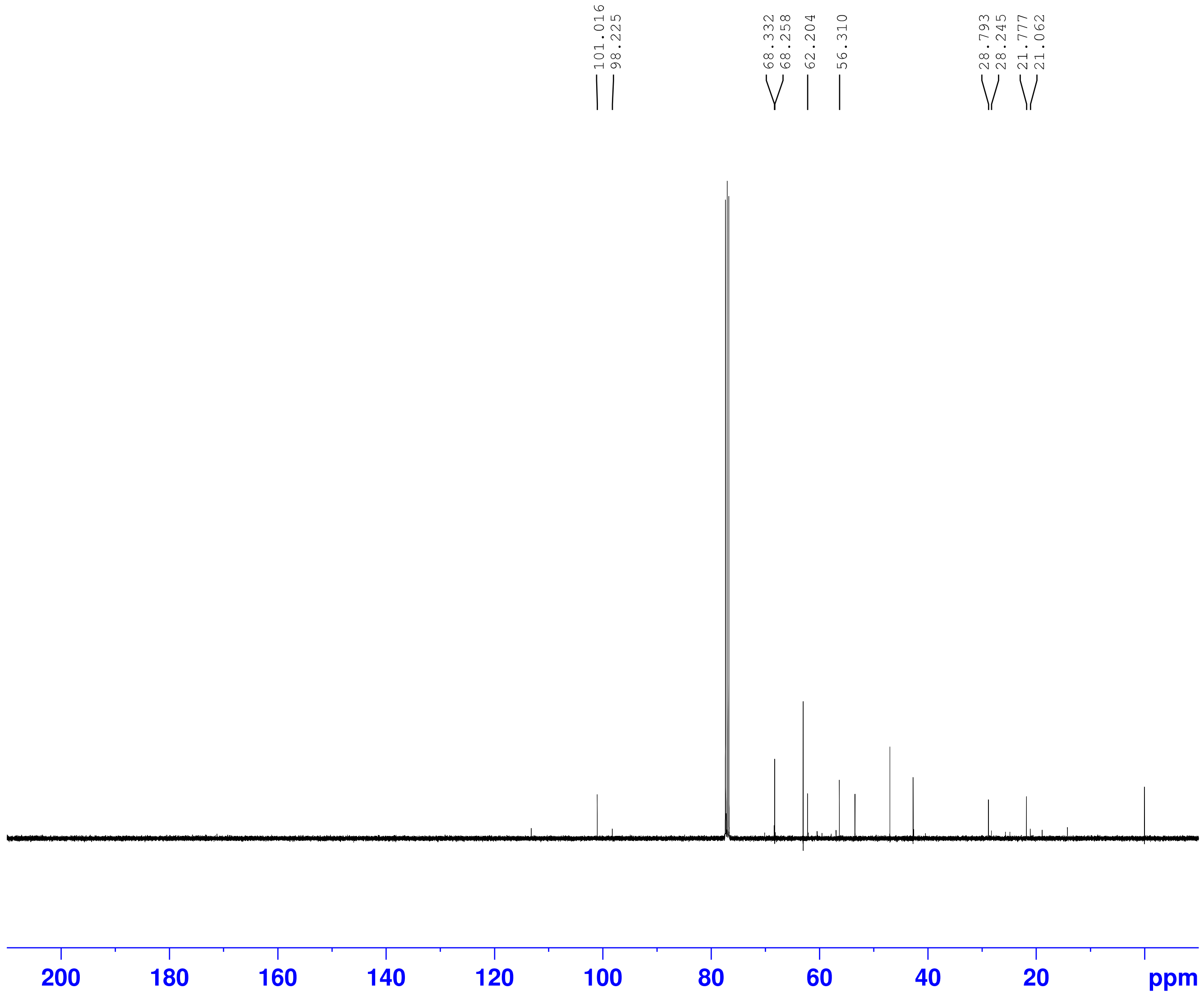


```
Current Data Parameters
NAME             kmd-5-177
EXPNO                    3
PROCNO                   1

F2 - Acquisition Parameters
Date_             20210315
Time                 16.26 h
INSTRUM              spect
PROBHD    Z150354_0001 (
PULPROG            zgpg30
TD                  65356
SOLVENT             CDCl3
NS                    256
DS                      4
SWH             24038.461 Hz
FIDRES           0.735616 Hz
AQ              1.3594048 sec
RG                  29.21
DW                 20.800 usec
DE                  25.00 usec
TE                  298.0 K
D1             2.00000000 sec
D11            0.03000000 sec
TD0                     1
SFO1          100.6655806 MHz
NUC1                  13C
P1                  10.00 usec
PLW1          19.75099945 W
SFO2          400.3016012 MHz
NUC2                   1H
CPDPRG[2          waltz16
PCPD2               80.00 usec
PLW2           4.67290020 W
PLW12          0.10514000 W
PLW13          0.05280100 W

F2 - Processing parameters
SI                 131072
SF            100.6555156 MHz
WDW                    EM
SSB      0
LB       0 Hz
GB       0
PC                   1.40
```

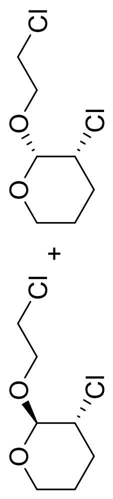

trans-7b + *cis*-7b

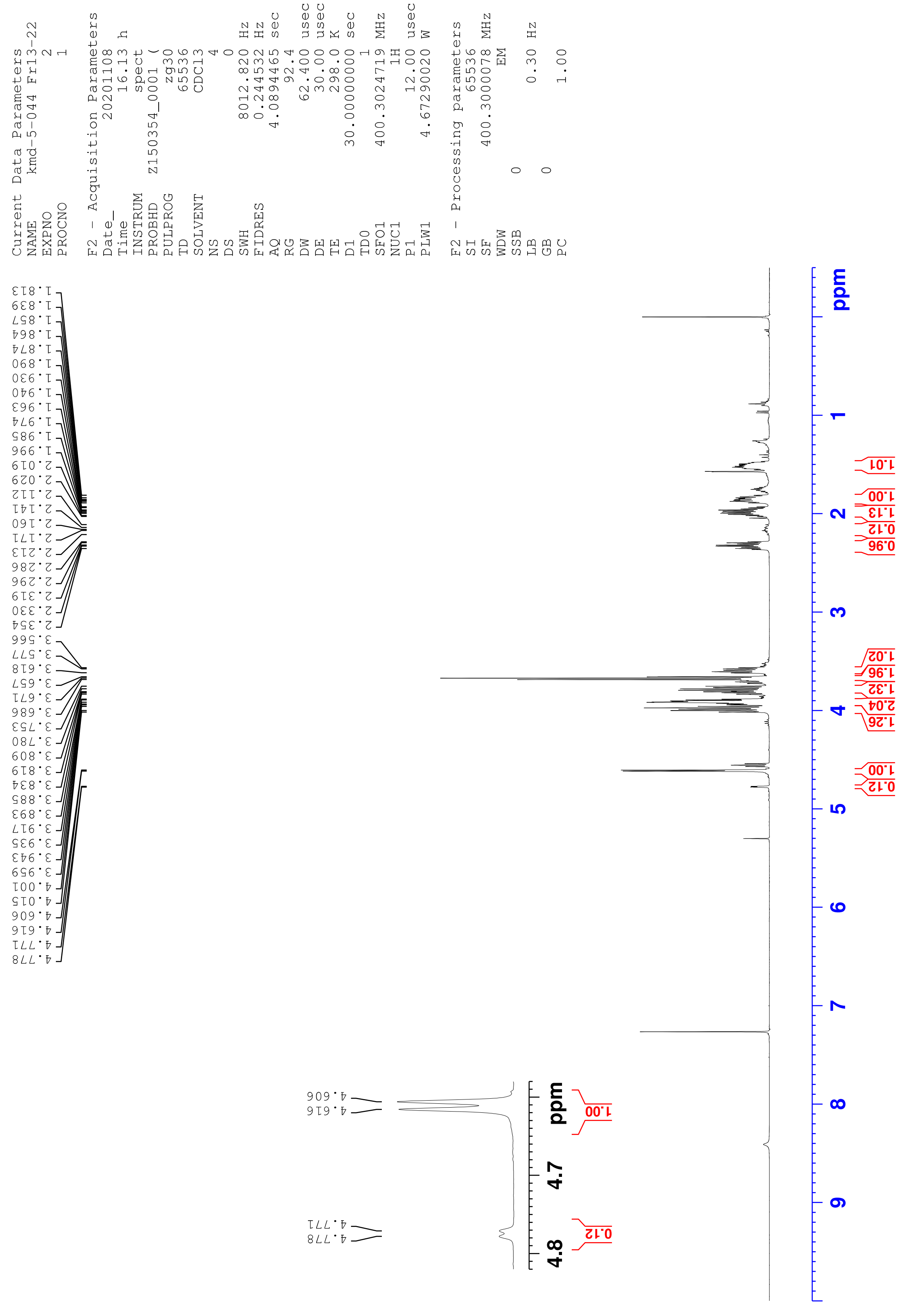

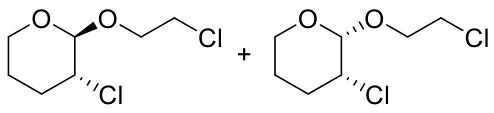

trans-**7b** *cis*-**7b**

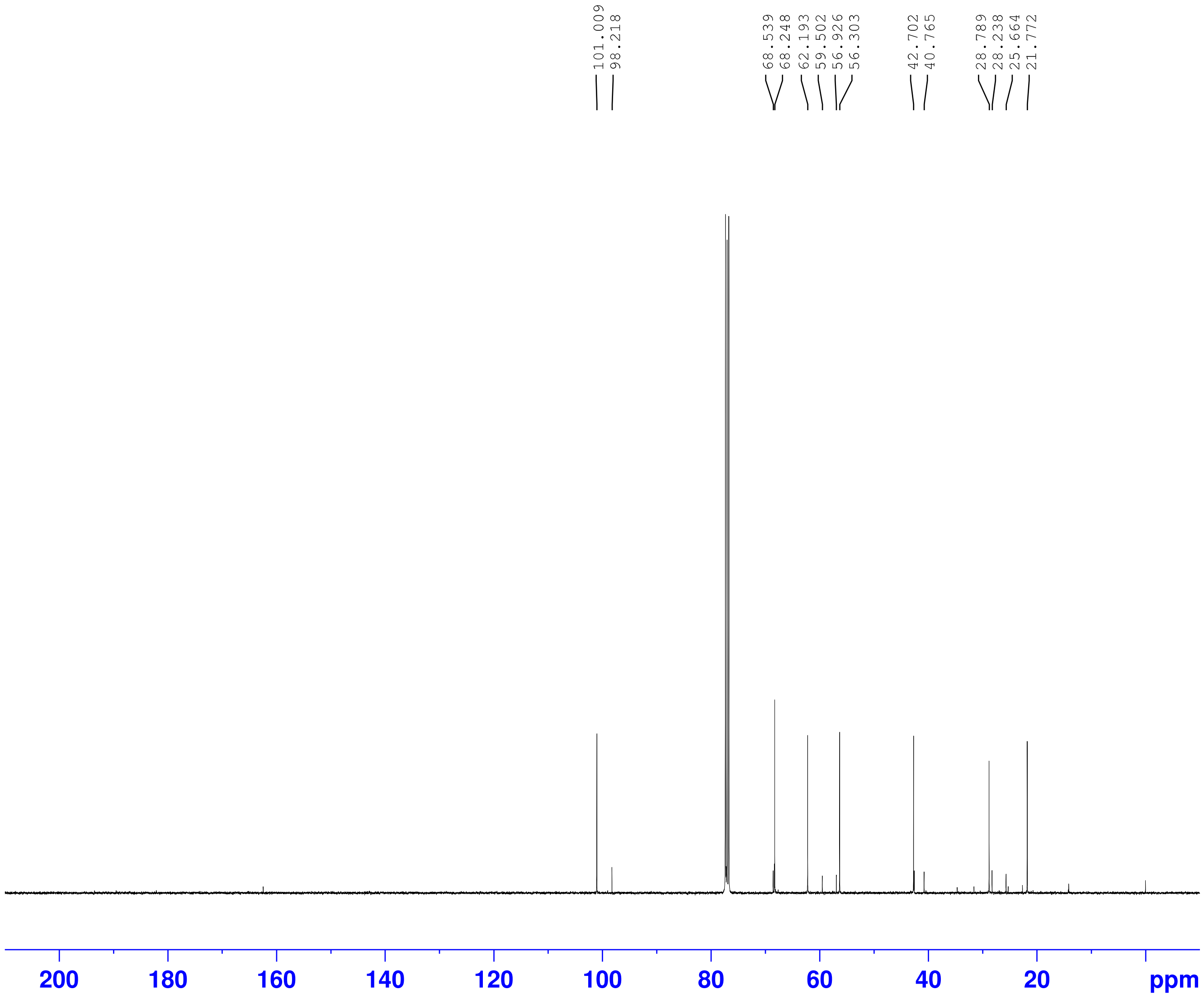


```
Current Data Parameters
NAME      kmd-5-044 Fr13-22
EXPNO                  3
PROCNO                 1

F2 - Acquisition Parameters
Date_           20201108
Time               18.01 h
INSTRUM            spect
PROBHD    Z150354_0001 (
PULPROG          zgpg30
TD                 65356
SOLVENT            CDCl3
NS                   256
DS                     4
SWH            24038.461 Hz
FIDRES          0.735616 Hz
AQ             1.3594048 sec
RG                 45.21
DW                20.800 usec
DE                 25.00 usec
TE                 298.0 K
D1            2.00000000 sec
D11           0.03000000 sec
TD0                    1
SFO1         100.6655806 MHz
NUC1                 13C
P1                 10.00 usec
PLW1         19.75099945 W
SFO2         400.3016012 MHz
NUC2                  1H
CPDPRG[2         waltz16
PCPD2              80.00 usec
PLW2          4.67290020 W
PLW12         0.10514000 W
PLW13         0.05280100 W

F2 - Processing parameters
SI                 32768
SF           100.6555158 MHz
WDW                   EM
SSB      0
LB                  1.00 Hz
GB       0
PC                  1.40
```

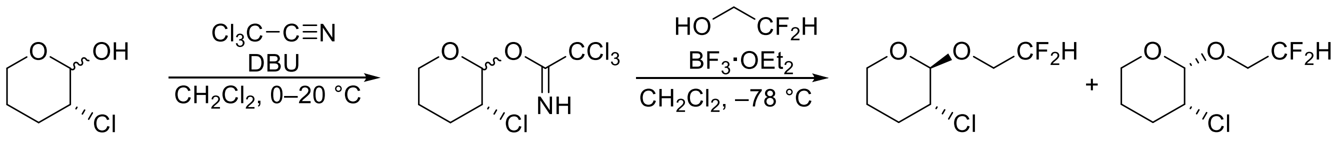

3 5a *trans*-7c *cis*-7c

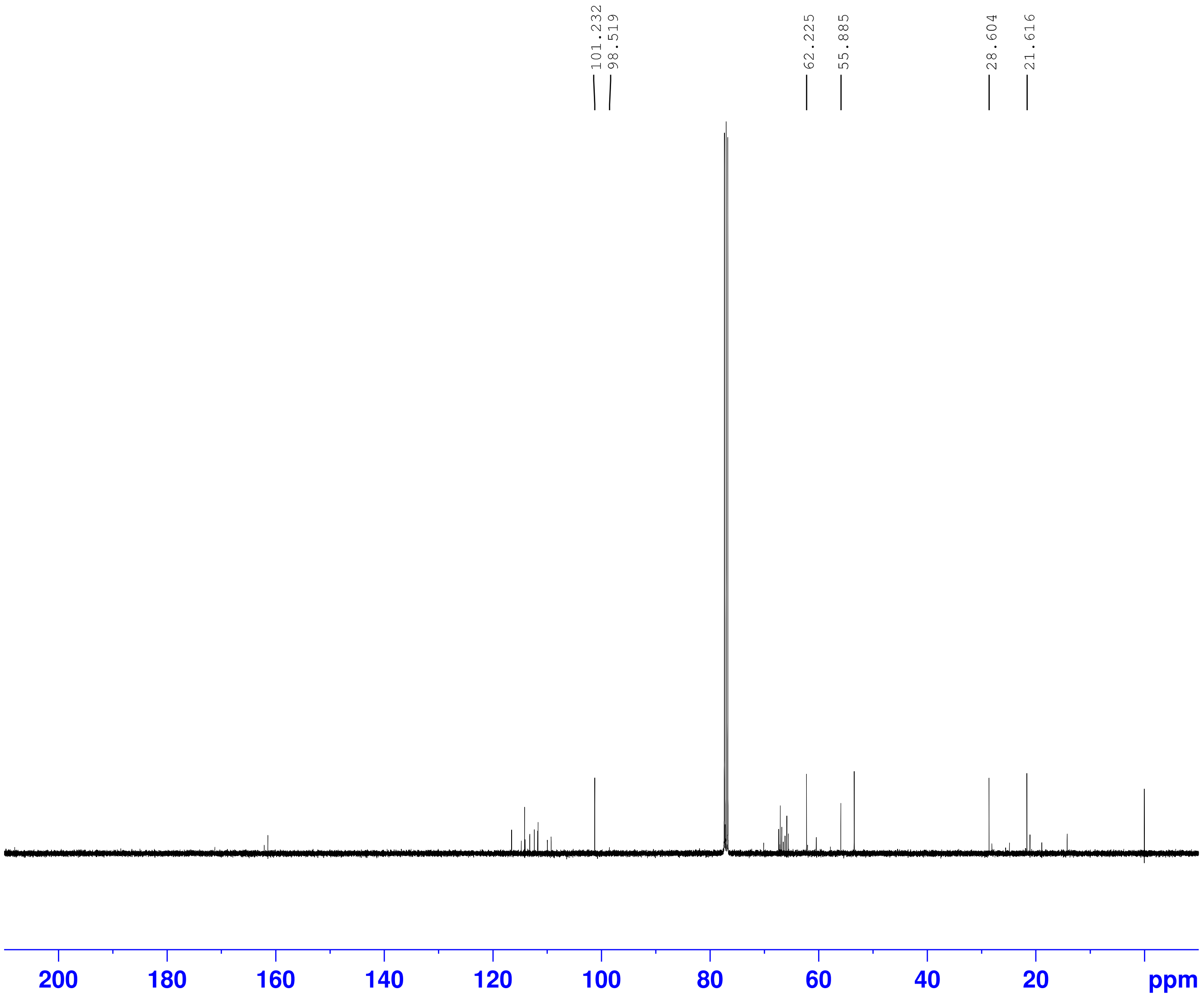


```
Current Data Parameters
NAME             kmd-5-179
EXPNO                    3
PROCNO                   1

F2 - Acquisition Parameters
Date_             20210316
Time                 17.24 h
INSTRUM              spect
PROBHD   Z150354_0001 (
PULPROG            zgpg30
TD                   65356
SOLVENT              CDCl3
NS                     256
DS                       4
SWH              24038.461 Hz
FIDRES            0.735616 Hz
AQ               1.3594048 sec
RG                    31.4
DW                  20.800 usec
DE                   25.00 usec
TE                   298.0 K
D1              2.00000000 sec
D11             0.03000000 sec
TD0                      1
SFO1           100.6655806 MHz
NUC1                   13C
P1                   10.00 usec
PLW1           19.75099945 W
SFO2           400.3016012 MHz
NUC2                    1H
CPDPRG[2           waltz16
PCPD2                80.00 usec
PLW2            4.67290020 W
PLW12           0.10514000 W
PLW13           0.05280100 W

F2 - Processing parameters
SI                  131072
SF             100.6555157 MHz
WDW                     EM
SSB      0
LB       0 Hz
GB       0
PC                    1.40
```

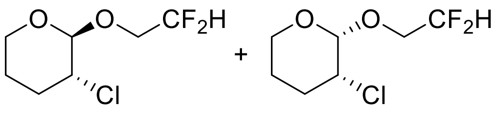

trans-**7c** + *cis*-**7c**

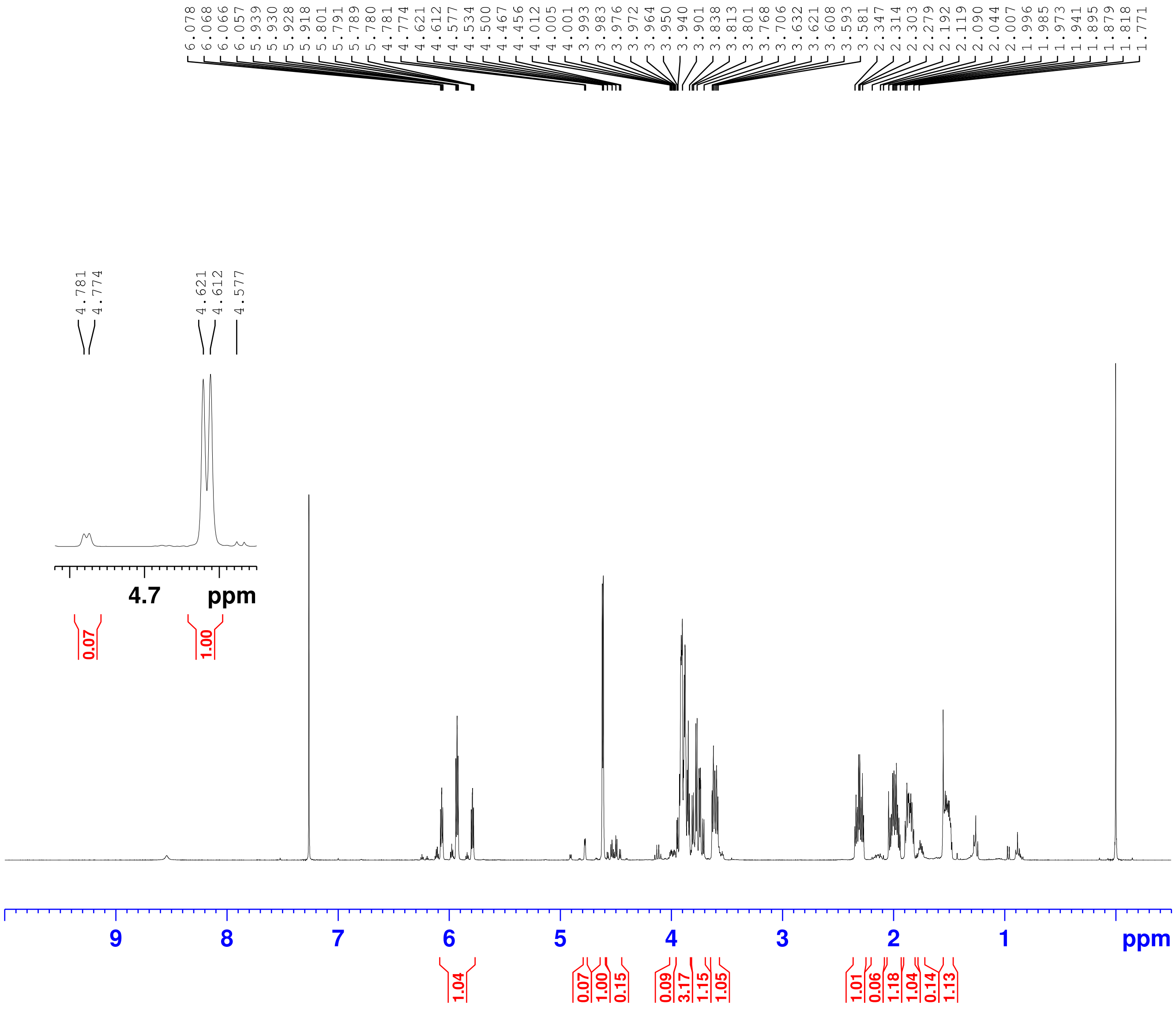


```
Current Data Parameters
NAME          kmd-5-179 Fr14-21
EXPNO                     2
PROCNO                    1

F2 - Acquisition Parameters
Date_              20210317
Time                  10.40 h
INSTRUM               spect
PROBHD     Z150354_0001 (
PULPROG                zg30
TD                    65536
SOLVENT               CDCl3
NS                        4
DS                        0
SWH                8012.820 Hz
FIDRES             0.244532 Hz
AQ                4.0894465 sec
RG                     92.4
DW                   62.400 usec
DE                    30.00 usec
TE                    298.0 K
D1              30.00000000 sec
TD0                       1
SFO1            400.3024719 MHz
NUC1                     1H
P1                    12.00 usec
PLW1             4.67290020 W

F2 - Processing parameters
SI                    65536
SF              400.3000086 MHz
WDW                      EM
SSB       0
LB                     0.30 Hz
GB        0
PC                     1.00
```

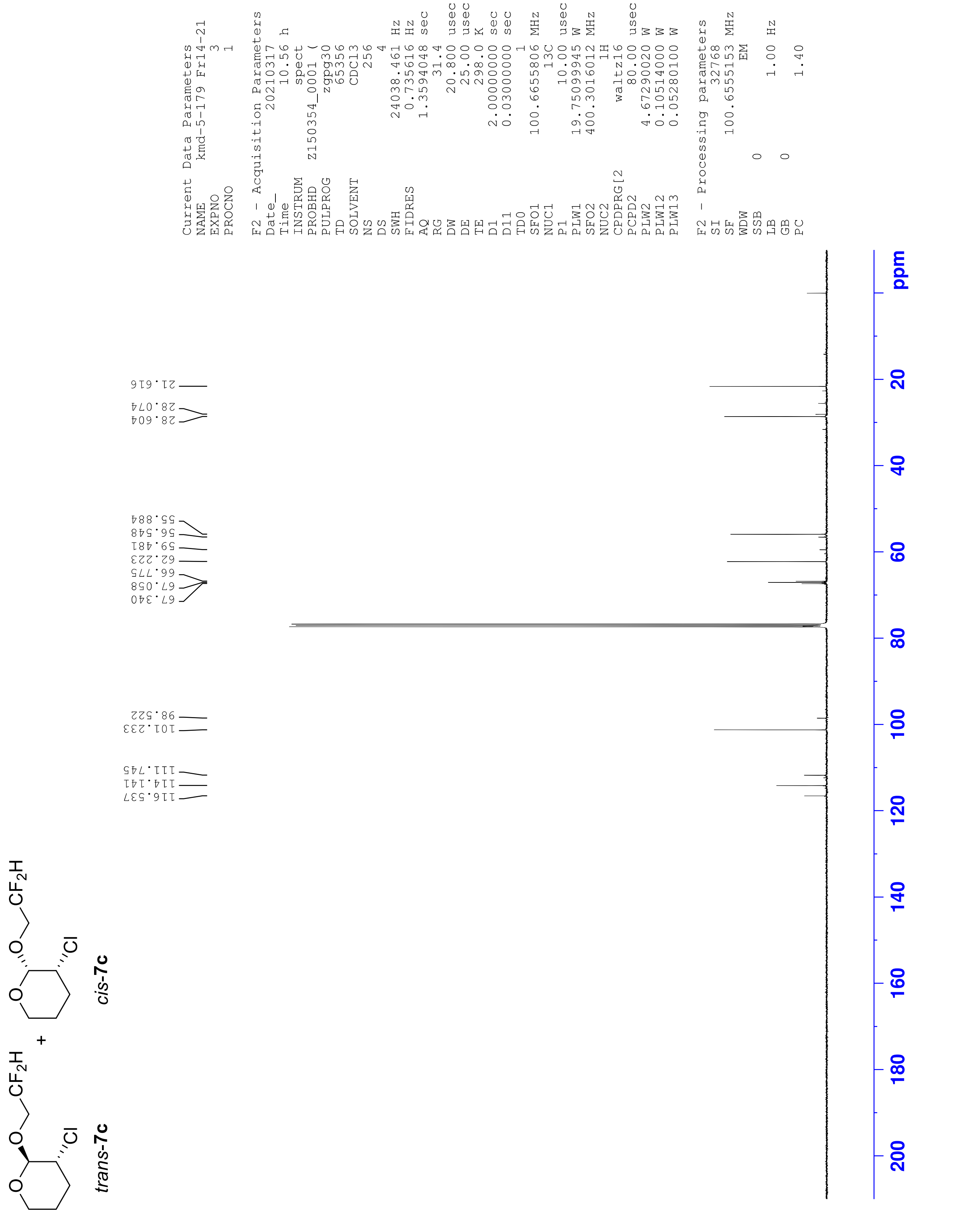
Current Data Parameters
NAME kmd-5-179 Fr14-21
EXPNO 3
PROCNO 1
F2 - Acquisition Parameters
Date_ 20210317
Time 10.56 h
INSTRUM spect
PROBHD Z150354_0001 (
PULPROG zgpg30
TD 65356
SOLVENT CDCl3
NS 256
DS 4
SWH 24038.461 Hz
FIDRES 0.735616 Hz
AQ 1.3594048 sec
RG 31.4
DW 20.800 usec
DE 25.00 usec
TE 298.0 K
D1 2.00000000 sec
D11 0.03000000 sec
TD0 1
SFO1 100.6655806 MHz
NUC1 13C
P1 10.00 usec
PLW1 19.75099945 W
SFO2 400.3016012 MHz
NUC2 1H
CPDPRG[2 waltz16
PCPD2 80.00 usec
PLW2 4.67290020 W
PLW12 0.10514000 W
PLW13 0.05280100 W
F2 - Processing parameters
SI 32768
SF 100.6555153 MHz
WDW EM
SSB 0
LB 1.00 Hz
GB 0
PC 1.40
21.616
28.074
28.604
55.884
56.548
59.481
62.223
66.775
67.058
67.340
98.522
101.233
111.745
114.141
116.537
ppm
20
40
60
80
100
120
140
160
180
200
trans-7c
cis-7c

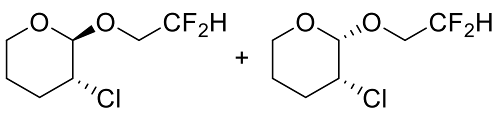

trans-**7c** + *cis*-**7c**

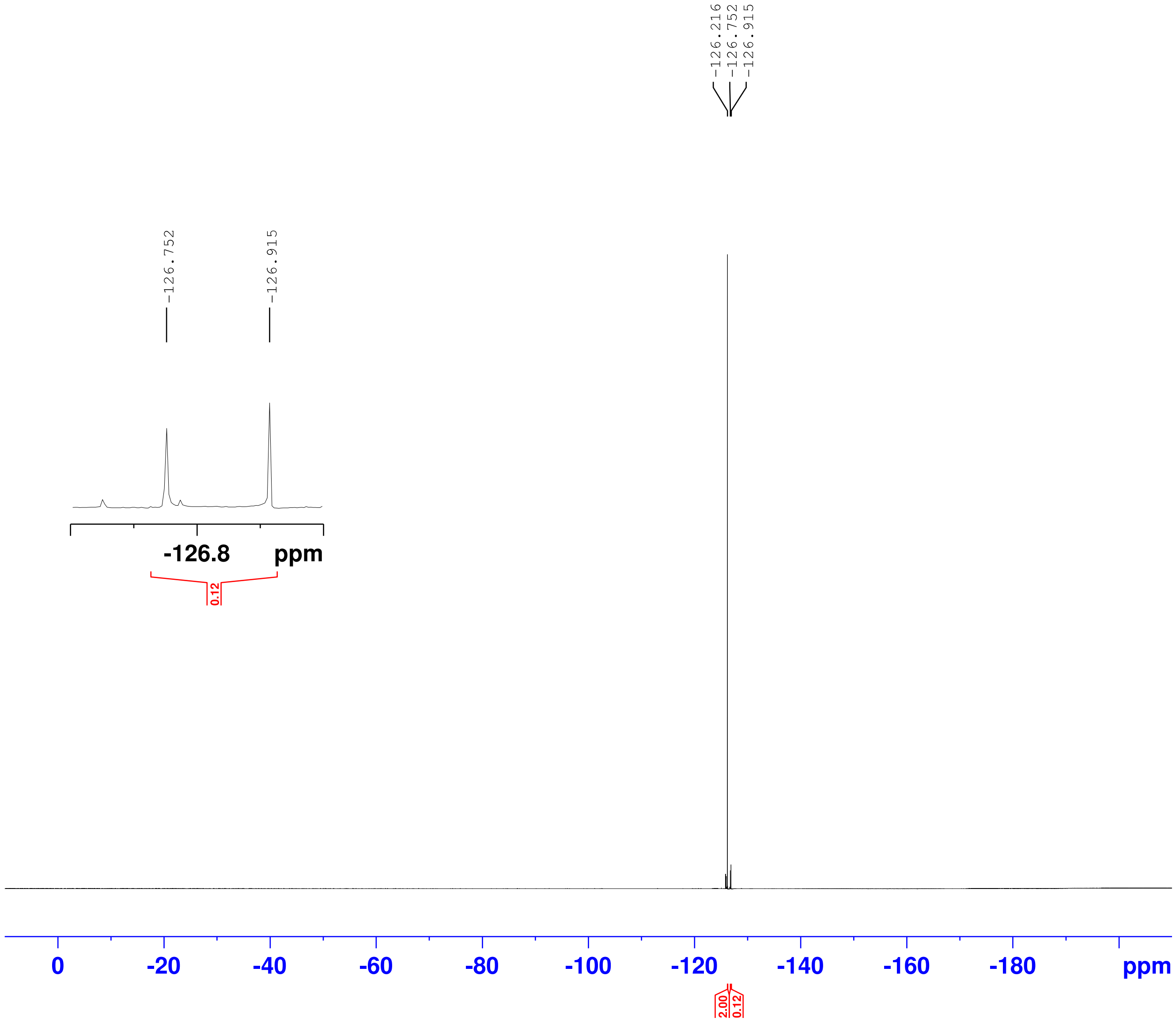


```
Current Data Parameters
NAME       kmd-5-179 Fr14-21 19F
EXPNO                    1
PROCNO                   1

F2 - Acquisition Parameters
Date_             20210405
Time                 10.17 h
INSTRUM              spect
PROBHD     Z108618_0422 (
PULPROG        zgfhigqn.2
TD                  131072
SOLVENT              CDCl3
NS                      16
DS                       4
SWH              89285.711 Hz
FIDRES            1.362392 Hz
AQ               0.7340032 sec
RG                  200.67
DW                   5.600 usec
DE                    6.50 usec
TE                   298.3 K
D1              1.00000000 sec
D11             0.03000000 sec
D12             0.00002000 sec
TD0                      1
SFO1           376.5453925 MHz
NUC1                   19F
P1                   15.00 usec
PLW1           16.89999962 W
SFO2           400.2216009 MHz
NUC2                    1H
CPDPRG[2           waltz16
PCPD2                90.00 usec
PLW2           14.69999981 W
PLW12           0.40832999 W

F2 - Processing parameters
SI                   65536
SF             376.5834175 MHz
WDW                     EM
SSB      0
LB                    0.30 Hz
GB       0
PC                    1.00
```

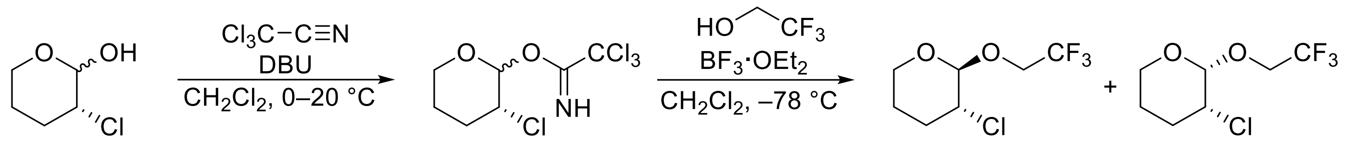

3 **5a** *trans*-**7d** *cis*-**7d**

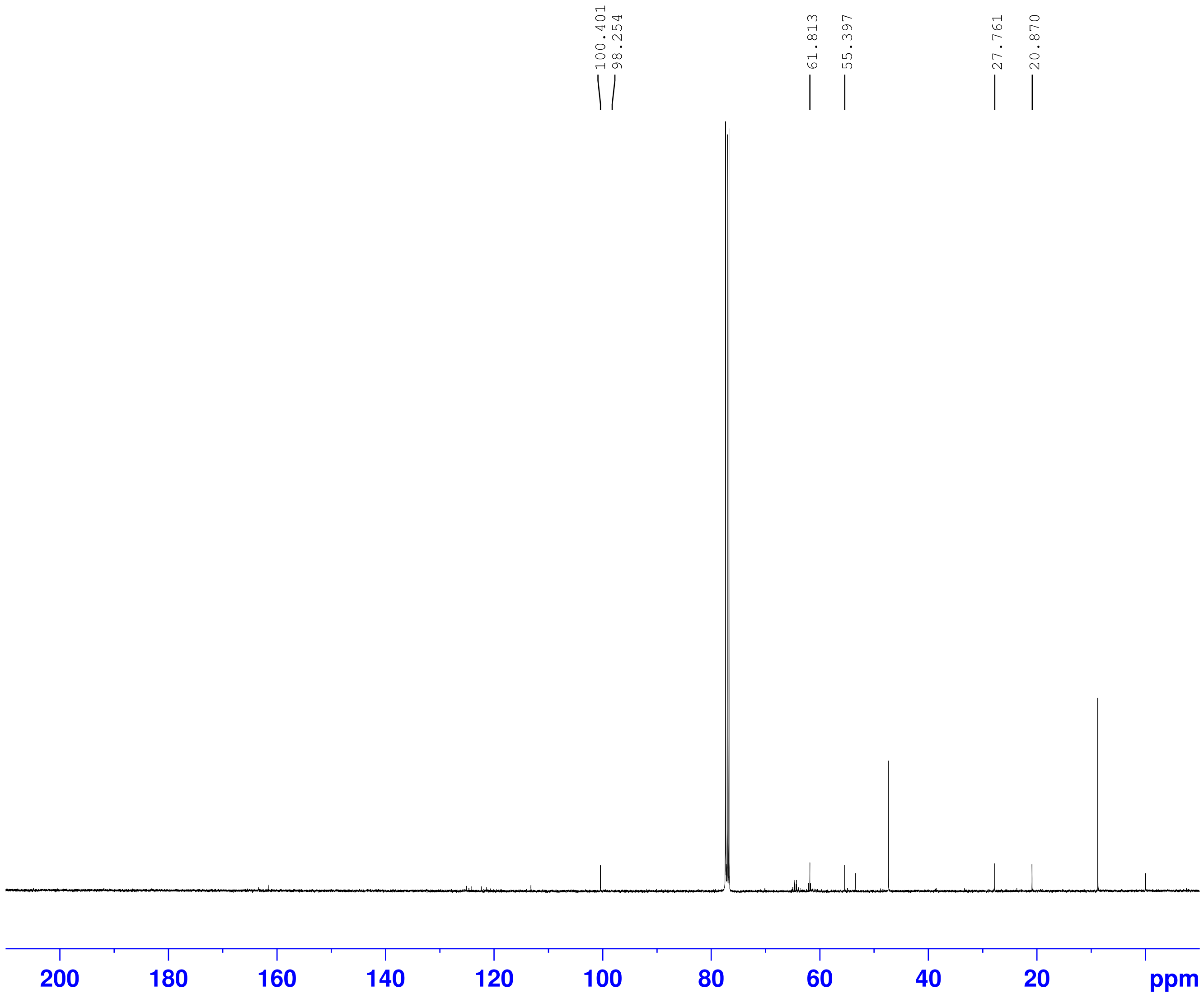

Current Data Parameters		
NAME	kmd-7-009	
EXPNO	3	
PROCNO	1	
F2 - Acquisition Parameters		
Date_	20220402	
Time	14.10	h
INSTRUM	spect	
PROBHD	Z150354_0001 (	
PULPROG	zgpg30	
TD	65356	
SOLVENT	CDCl3	
NS	256	
DS	4	
SWH	24038.461	Hz
FIDRES	0.735616	Hz
AQ	1.3594048	sec
RG	51.78	
DW	20.800	usec
DE	25.00	usec
TE	298.0	K
D1	2.00000000	sec
D11	0.03000000	sec
TD0	1	
SFO1	100.6655806	MHz
NUC1	13C	
P1	10.00	usec
PLW1	17.99699974	W
SFO2	400.3016012	MHz
NUC2	1H	
CPDPRG[2	waltz16	
PCPD2	80.00	usec
PLW2	4.64209986	W
PLW12	0.10445000	W
PLW13	0.05245300	W
F2 - Processing parameters		
SI	32768	
SF	100.6555144	MHz
WDW	EM	
SSB	0	
LB	1.00	Hz
GB	0	
PC	1.40	

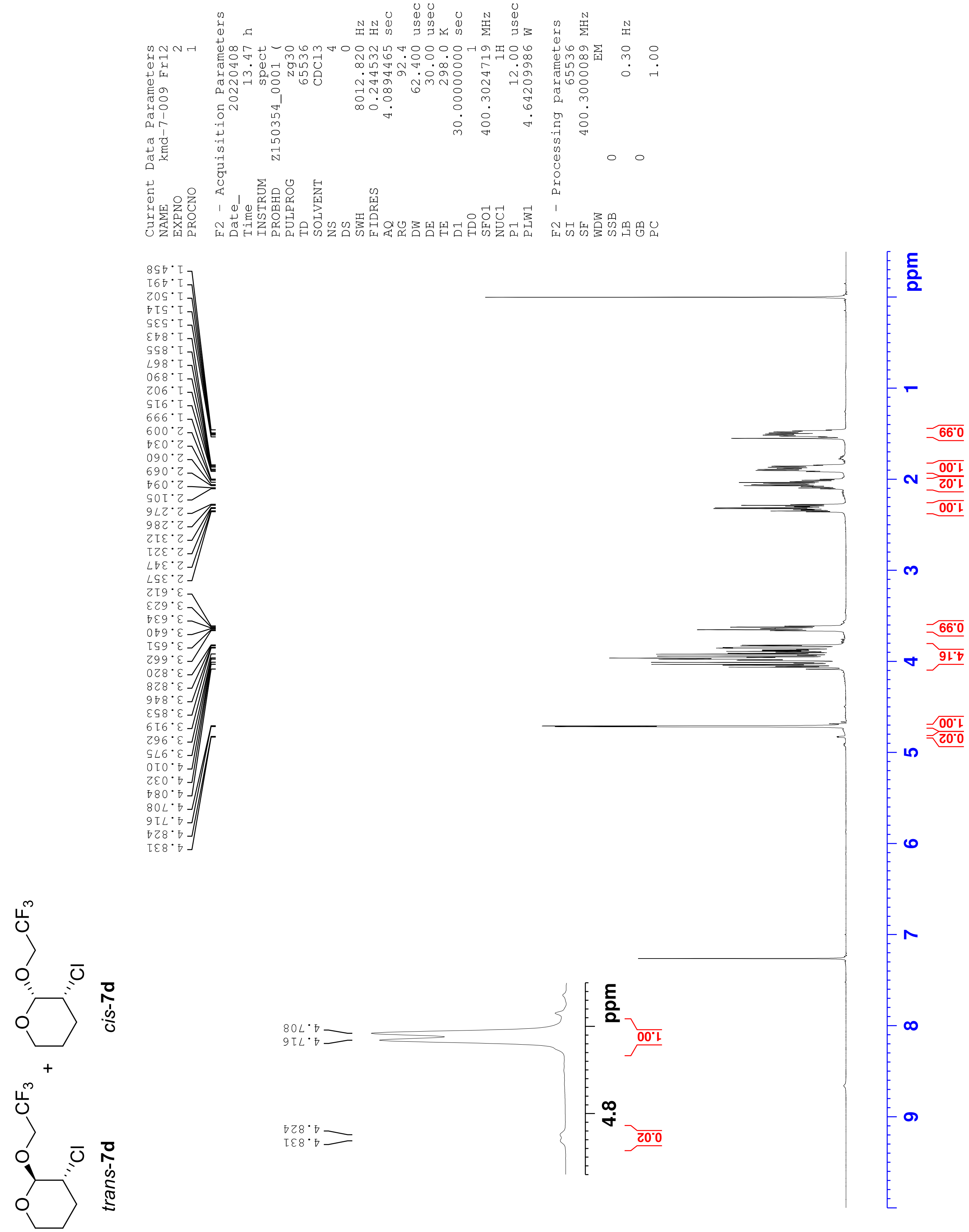
trans-7d
cis-7d
Current Data Parameters
NAME kmd-7-009 Fr12
EXPNO 2
PROCNO 1
F2 - Acquisition Parameters
Date_ 20220408
Time 13.47 h
INSTRUM spect
PROBHD Z150354_0001 (
PULPROG zg30
TD 65536
SOLVENT CDCl3
NS 4
DS 0
SWH 8012.820 Hz
FIDRES 0.244532 Hz
AQ 4.0894465 sec
RG 92.4
DW 62.400 usec
DE 30.00 usec
TE 298.0 K
D1 30.00000000 sec
TD0 1
SFO1 400.3024719 MHz
NUC1 1H
P1 12.00 usec
PLW1 4.64209986 W
F2 - Processing parameters
SI 65536
SF 400.3000089 MHz
WDW EM
SSB 0
LB 0.30 Hz
GB 0
PC 1.00
1.458 1.491 1.502 1.514 1.535 1.843 1.855 1.867 1.890 1.902 1.915 1.999 2.009 2.034 2.060 2.069 2.094 2.105 2.276 2.286 2.312 2.321 2.347 2.357 3.612 3.623 3.634 3.640 3.651 3.662 3.820 3.828 3.846 3.853 3.919 3.962 3.975 4.010 4.032 4.084 4.708 4.716 4.824 4.831
4.831 4.824 4.716 4.708
4.8 ppm
0.02 1.00
ppm 1 2 3 4 5 6 7 8 9
0.99 1.00 1.02 1.00 0.99 4.16 1.00 0.02

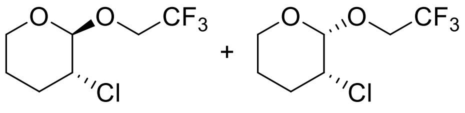

trans-**7d** *cis*-**7d**

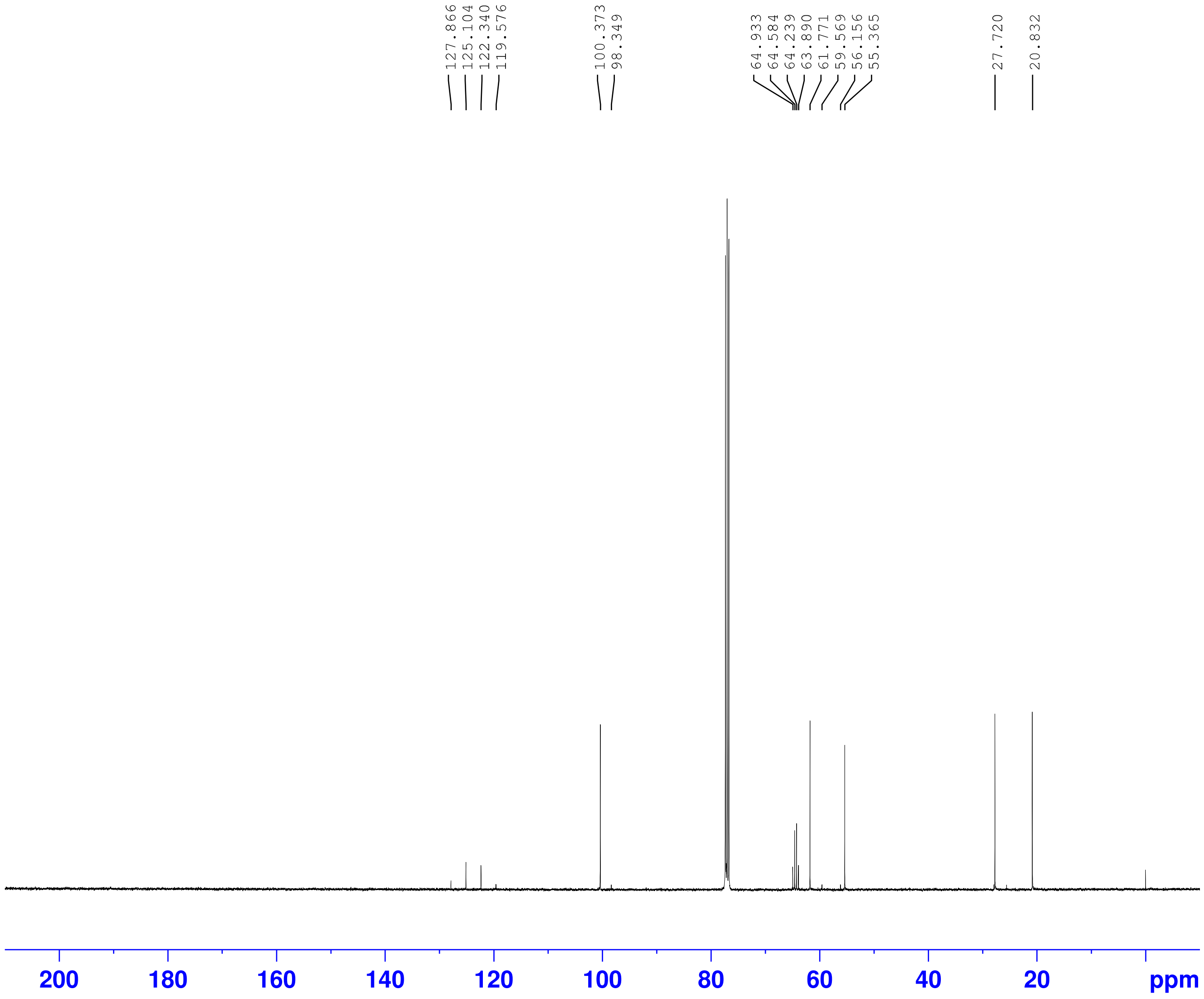


```
Current Data Parameters
NAME      kmd-7-009 Fr12
EXPNO                  3
PROCNO                 1

F2 - Acquisition Parameters
Date_           20220408
Time               18.38 h
INSTRUM            spect
PROBHD    Z150354_0001 (
PULPROG          zgpg30
TD                 65356
SOLVENT            CDCl3
NS                   256
DS                     4
SWH            24038.461 Hz
FIDRES          0.735616 Hz
AQ             1.3594048 sec
RG                  31.4
DW                20.800 usec
DE                 25.00 usec
TE                 298.0 K
D1            2.00000000 sec
D11           0.03000000 sec
TD0                    1
SFO1         100.6655806 MHz
NUC1                 13C
P1                 10.00 usec
PLW1         17.99699974 W
SFO2         400.3016012 MHz
NUC2                  1H
CPDPRG[2         waltz16
PCPD2              80.00 usec
PLW2          4.64209986 W
PLW12         0.10445000 W
PLW13         0.05245300 W

F2 - Processing parameters
SI                 32768
SF           100.6555151 MHz
WDW                   EM
SSB      0
LB                  1.00 Hz
GB       0
PC                  1.40
```

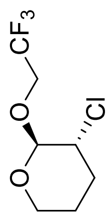

trans-**7d**

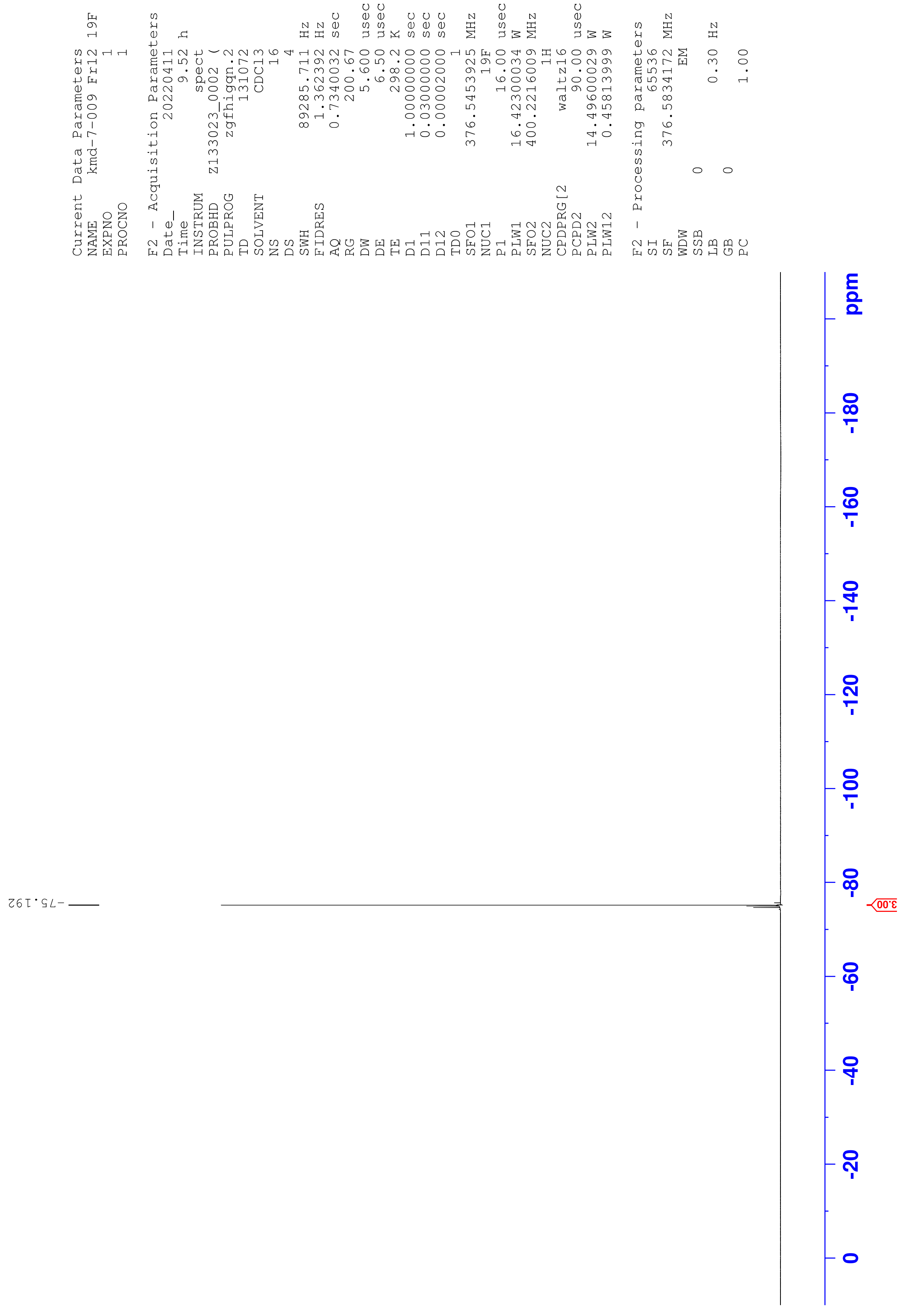
-75.192
Current Data Parameters
NAME kmd-7-009 Fr12 19F
EXPNO 1
PROCNO 1
F2 - Acquisition Parameters
Date_ 20220411
Time 9.52 h
INSTRUM spect
PROBHD Z133023_0002 (
PULPROG zgfhigqn.2
TD 131072
SOLVENT CDCl3
NS 16
DS 4
SWH 89285.711 Hz
FIDRES 1.362392 Hz
AQ 0.7340032 sec
RG 200.67
DW 5.600 usec
DE 6.50 usec
TE 298.2 K
D1 1.00000000 sec
D11 0.03000000 sec
D12 0.00002000 sec
TD0 1
SFO1 376.5453925 MHz
NUC1 19F
P1 16.00 usec
PLW1 16.42300034 W
SFO2 400.2216009 MHz
NUC2 1H
CPDPRG[2 waltz16
PCPD2 90.00 usec
PLW2 14.49600029 W
PLW12 0.45813999 W
F2 - Processing parameters
SI 65536
SF 376.5834172 MHz
WDW EM
SSB 0
LB 0.30 Hz
GB 0
PC 1.00
3.00
0
-20
-40
-60
-80
-100
-120
-140
-160
-180
ppm

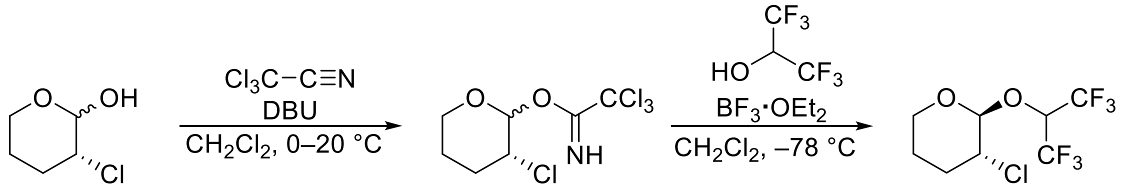

3 5a 7e

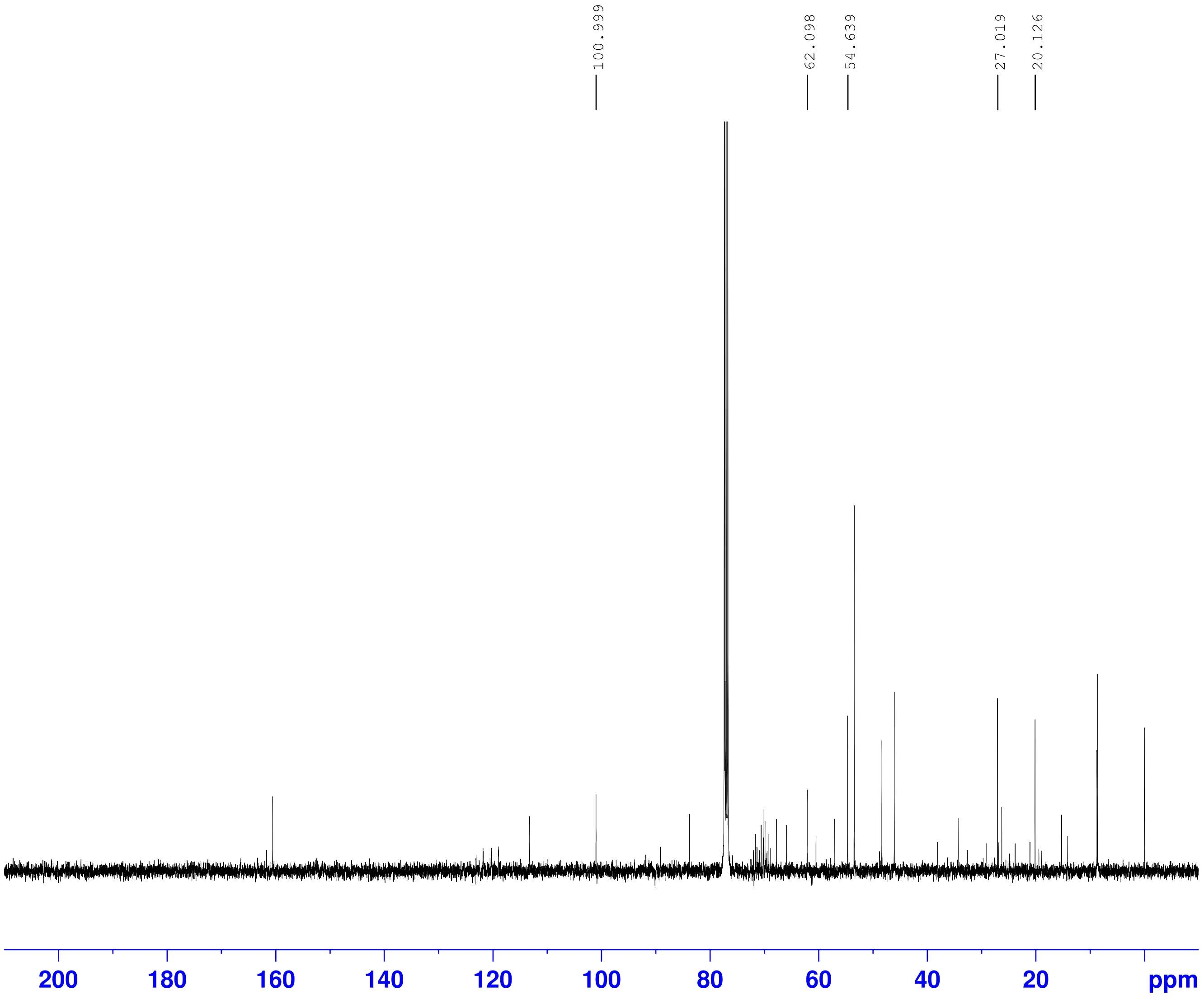


```
Current Data Parameters
NAME             kmd-5-181
EXPNO                    3
PROCNO                   1

F2 - Acquisition Parameters
Date_             20210316
Time                 16.42 h
INSTRUM              spect
PROBHD    Z150354_0001 (
PULPROG            zgpg30
TD                   65356
SOLVENT              CDCl3
NS                     256
DS                       4
SWH              24038.461 Hz
FIDRES            0.735616 Hz
AQ               1.3594048 sec
RG                   29.21
DW                  20.800 usec
DE                   25.00 usec
TE                   298.0 K
D1              2.00000000 sec
D11             0.03000000 sec
TD0                      1
SFO1           100.6655806 MHz
NUC1                   13C
P1                   10.00 usec
PLW1           19.75099945 W
SFO2           400.3016012 MHz
NUC2                    1H
CPDPRG[2           waltz16
PCPD2                80.00 usec
PLW2            4.67290020 W
PLW12           0.10514000 W
PLW13           0.05280100 W

F2 - Processing parameters
SI                   32768
SF             100.6555145 MHz
WDW                     EM
SSB      0
LB                    1.00 Hz
GB       0
PC                    1.40
```

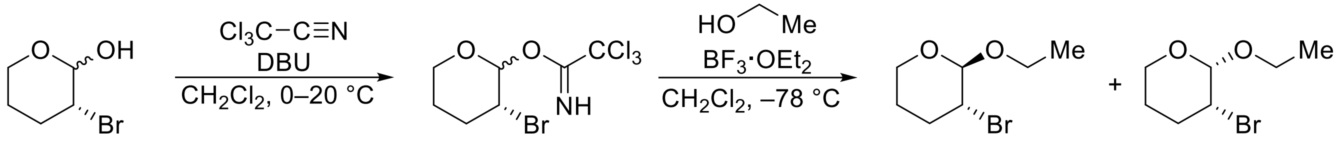

4 **6** *trans*-**8a** *cis*-**8a**

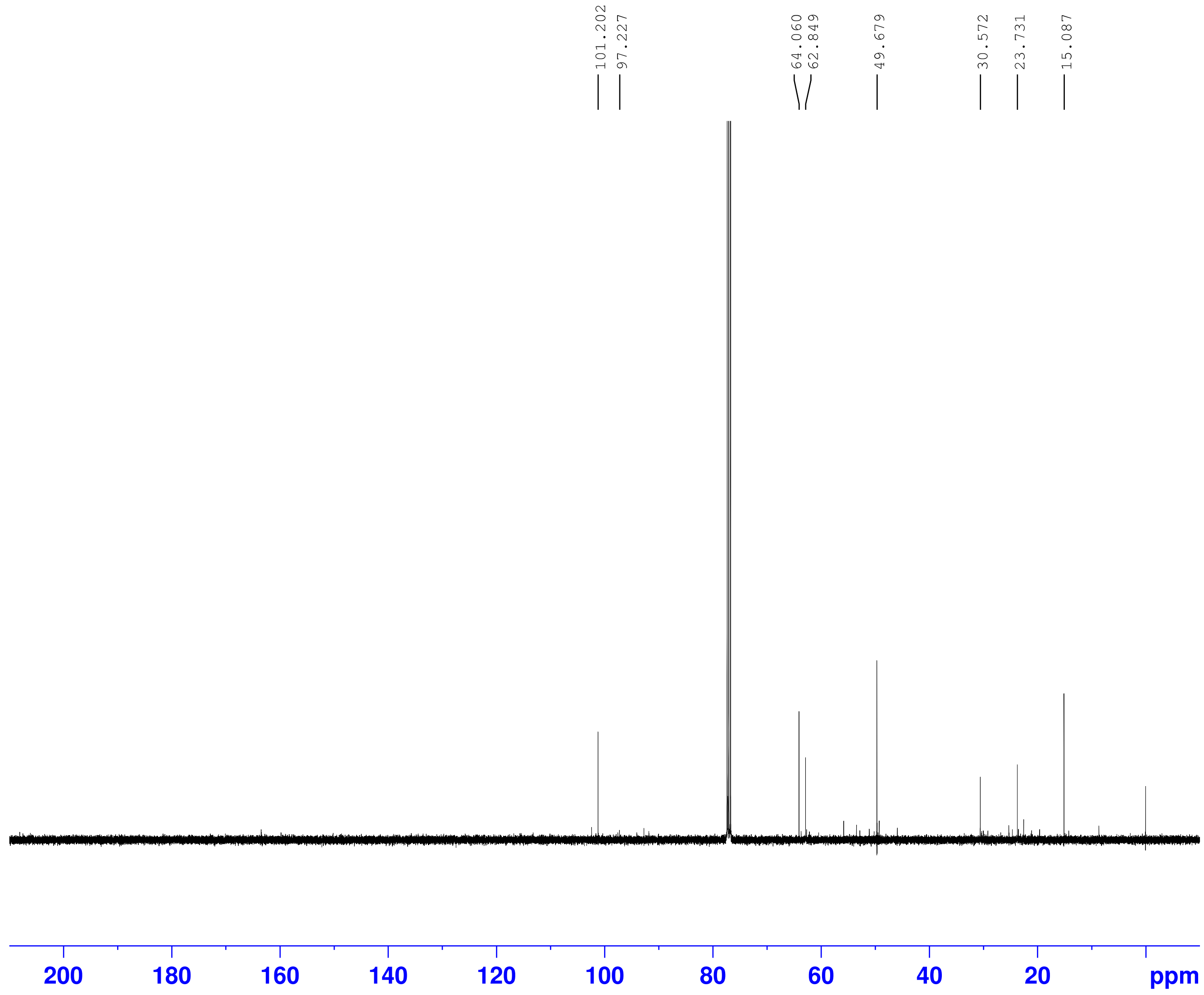


```
Current Data Parameters
NAME             kmd-5-126
EXPNO                    3
PROCNO                   1

F2 - Acquisition Parameters
Date_             20210118
Time                 21.26 h
INSTRUM              spect
PROBHD     Z150354_0001 (
PULPROG            zgpg30
TD                   65356
SOLVENT              CDCl3
NS                     256
DS                       4
SWH              24038.461 Hz
FIDRES            0.735616 Hz
AQ               1.3594048 sec
RG                   29.21
DW                  20.800 usec
DE                   25.00 usec
TE                   298.0 K
D1              2.00000000 sec
D11             0.03000000 sec
TD0                      1
SFO1           100.6655806 MHz
NUC1                   13C
P1                   10.00 usec
PLW1           19.75099945 W
SFO2           400.3016012 MHz
NUC2                    1H
CPDPRG[2           waltz16
PCPD2                80.00 usec
PLW2            4.67290020 W
PLW12           0.10514000 W
PLW13           0.05280100 W

F2 - Processing parameters
SI                  131072
SF             100.6555151 MHz
WDW                     EM
SSB       0
LB        0 Hz
GB        0
PC                    1.40
```

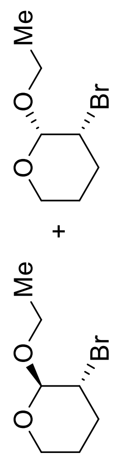

trans-**8a** + *cis*-**8a**

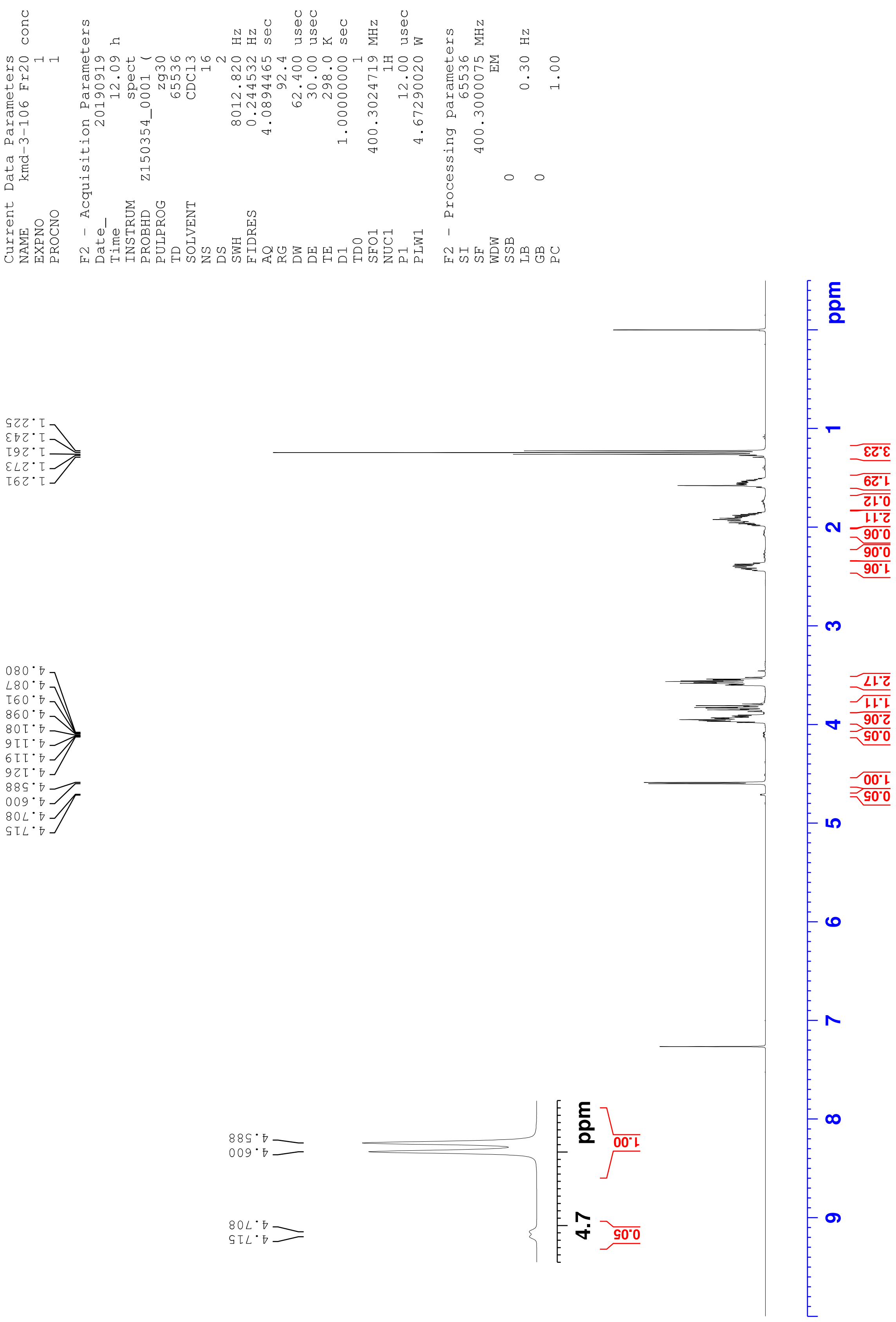

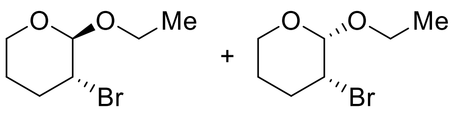

trans-**8a** + *cis*-**8a**

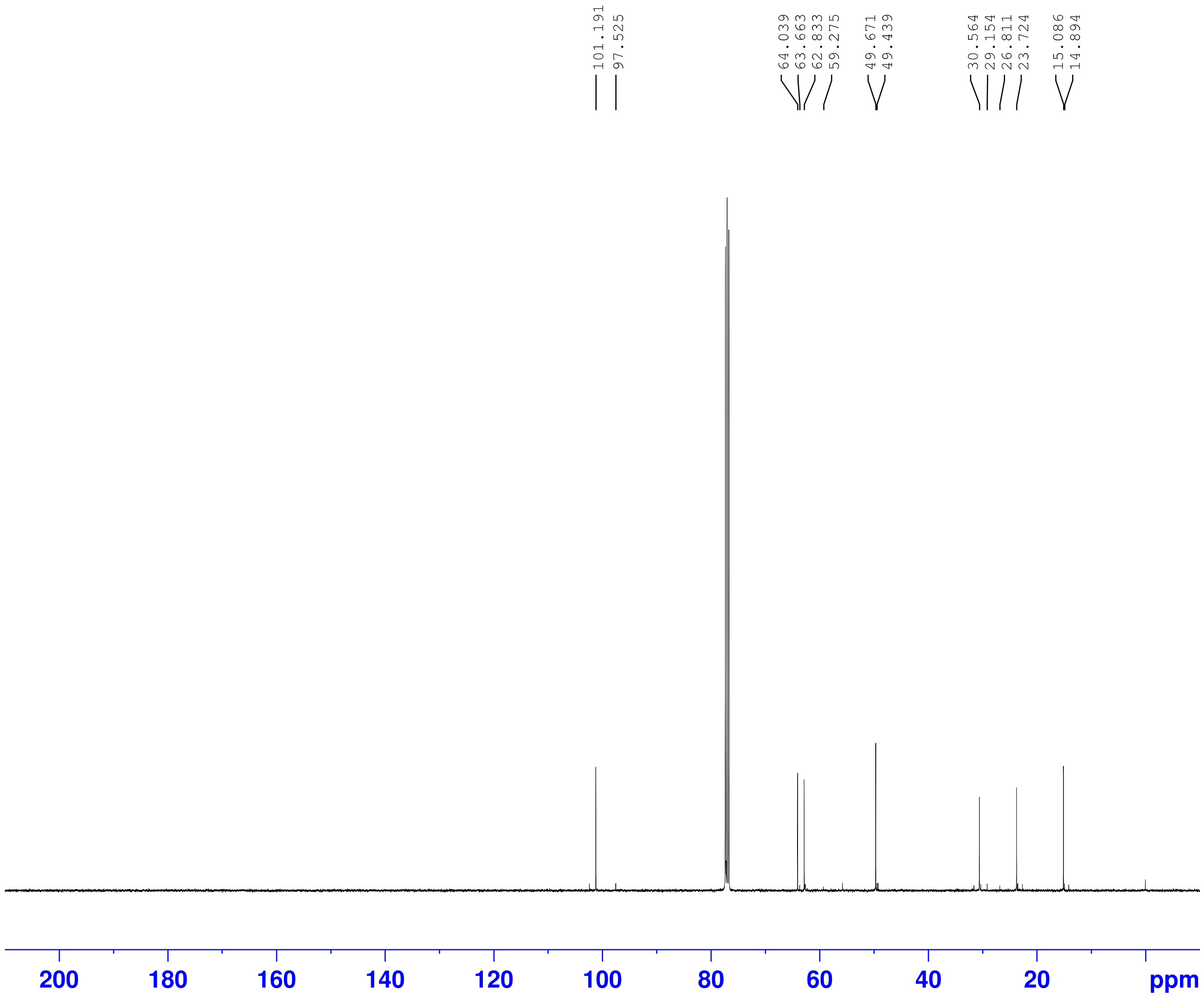


```
Current Data Parameters
NAME       kmd-5-126 Fr11-14
EXPNO                  3
PROCNO                 1

F2 - Acquisition Parameters
Date_            20210119
Time                14.18 h
INSTRUM             spect
PROBHD   Z150354_0001 (
PULPROG           zgpg30
TD                 65356
SOLVENT            CDCl3
NS                   256
DS                     4
SWH            24038.461 Hz
FIDRES          0.735616 Hz
AQ             1.3594048 sec
RG                 36.68
DW                20.800 usec
DE                 25.00 usec
TE                 298.0 K
D1            2.00000000 sec
D11           0.03000000 sec
TD0                    1
SFO1        100.6655806 MHz
NUC1                 13C
P1                 10.00 usec
PLW1         19.75099945 W
SFO2        400.3016012 MHz
NUC2                  1H
CPDPRG[2         waltz16
PCPD2              80.00 usec
PLW2          4.67290020 W
PLW12         0.10514000 W
PLW13         0.05280100 W

F2 - Processing parameters
SI                 32768
SF          100.6555151 MHz
WDW                   EM
SSB      0
LB                  1.00 Hz
GB       0
PC                  1.40
```

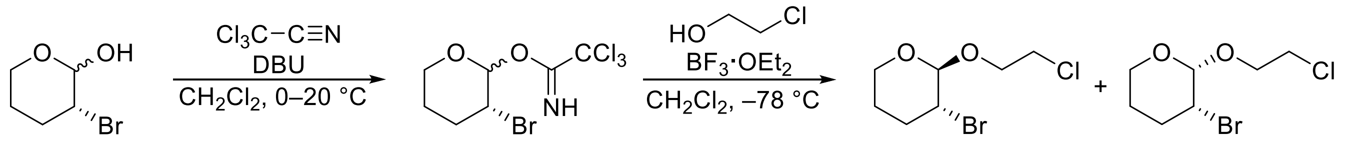

4 → **6** → *trans*-**8b** + *cis*-**8b**

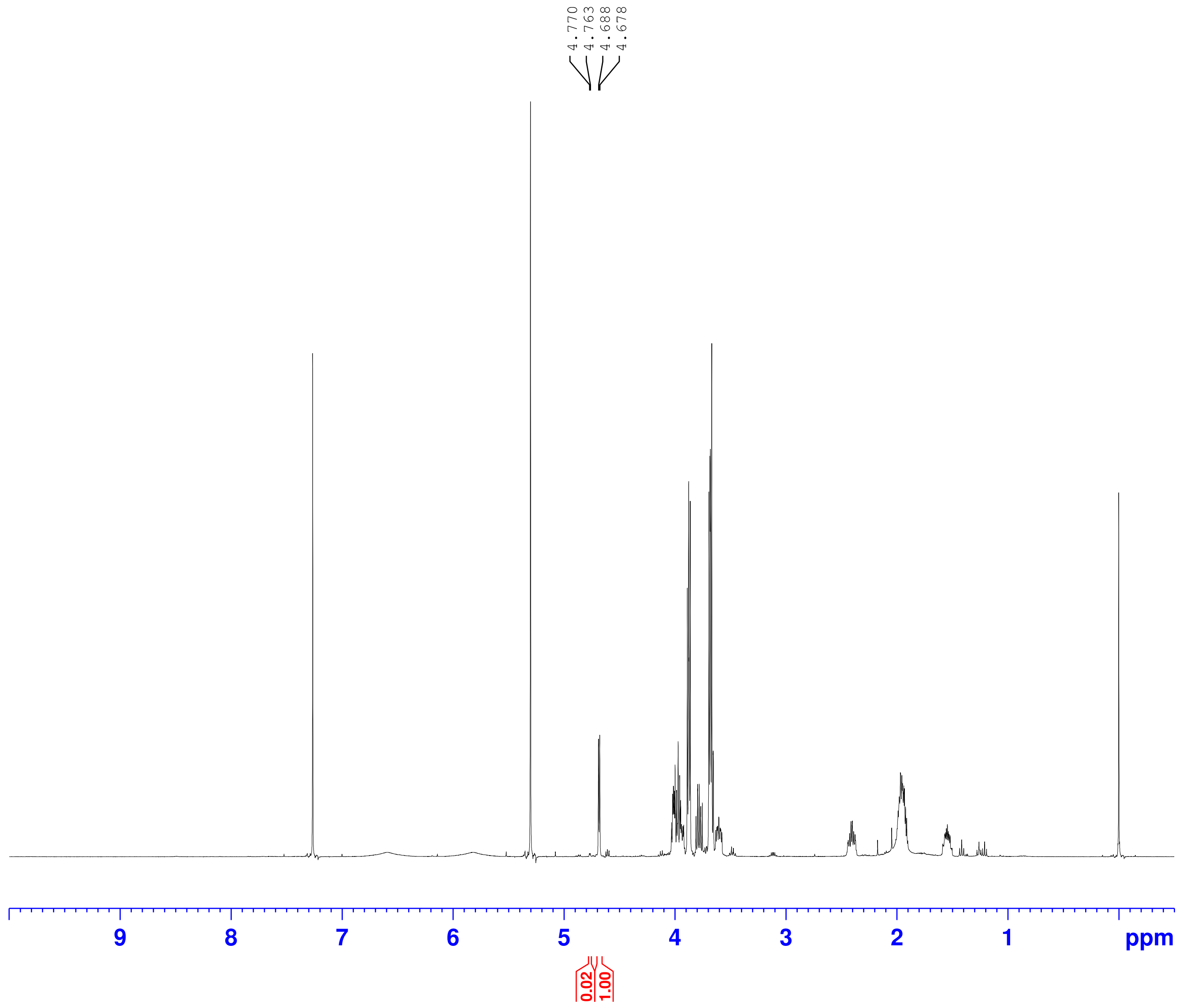


```
Current Data Parameters
NAME             kmd-5-127
EXPNO                    2
PROCNO                   1

F2 - Acquisition Parameters
Date_             20210118
Time                 17.26 h
INSTRUM              spect
PROBHD    Z150354_0001 (
PULPROG               zg30
TD                   65536
SOLVENT              CDCl3
NS                       4
DS                       0
SWH               8012.820 Hz
FIDRES            0.244532 Hz
AQ               4.0894465 sec
RG                    92.4
DW                  62.400 usec
DE                   30.00 usec
TE                   298.0 K
D1             30.00000000 sec
TD0                      1
SFO1           400.3024719 MHz
NUC1                    1H
P1                   12.00 usec
PLW1            4.67290020 W

F2 - Processing parameters
SI                   65536
SF             400.3000078 MHz
WDW                     EM
SSB      0
LB                    0.30 Hz
GB       0
PC                    1.00
```

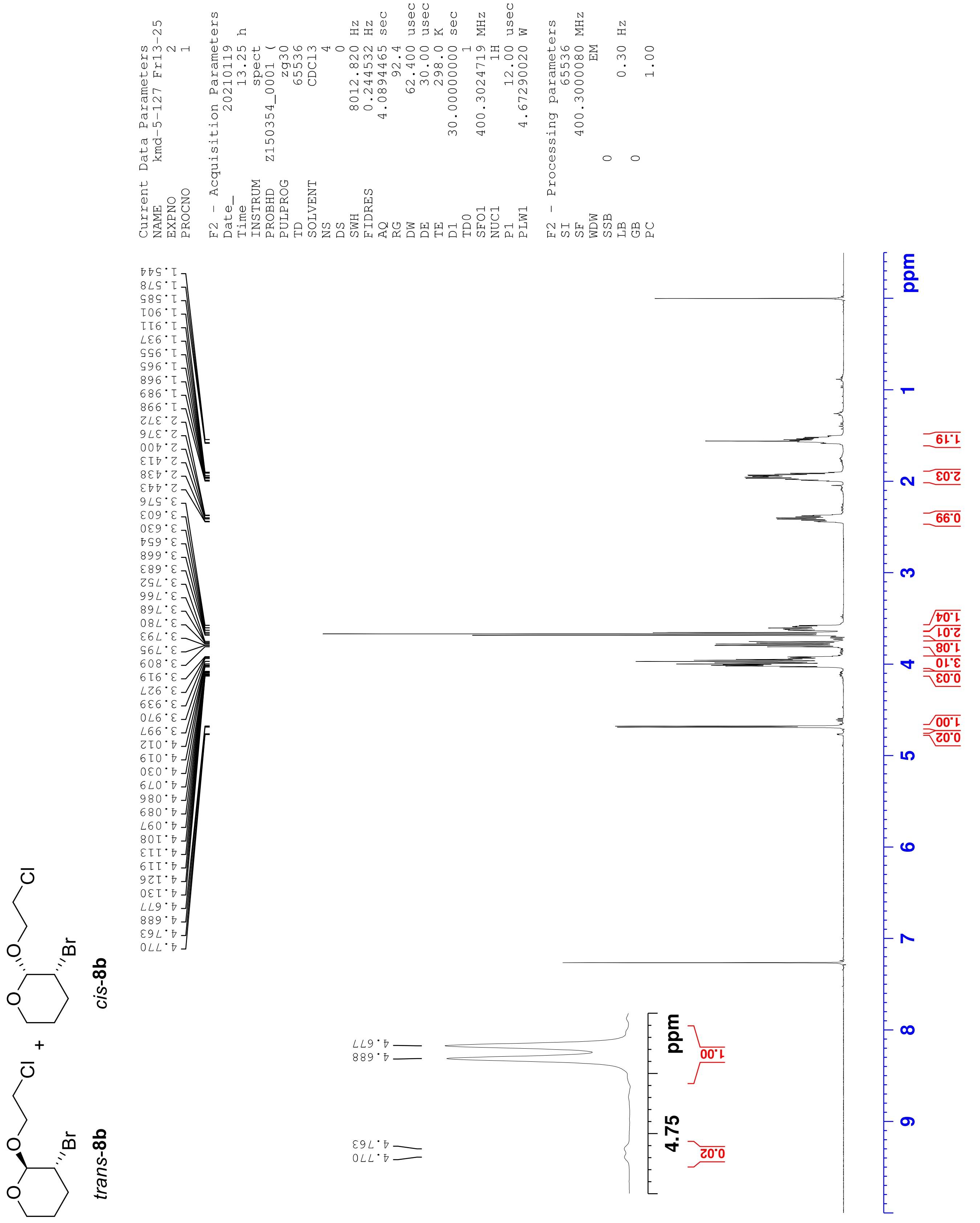
trans-8b
cis-8b
Br
Br
Cl
Cl
O
+
Current Data Parameters
NAME kmd-5-127 Fr13-25
EXPNO 2
PROCNO 1
F2 - Acquisition Parameters
Date_ 20210119
Time 13.25 h
INSTRUM spect
PROBHD Z150354_0001 (
PULPROG zg30
TD 65536
SOLVENT CDCl3
NS 4
DS 0
SWH 8012.820 Hz
FIDRES 0.244532 Hz
AQ 4.0894465 sec
RG 92.4
DW 62.400 usec
DE 30.00 usec
TE 298.0 K
D1 30.00000000 sec
TD0 1
SFO1 400.3024719 MHz
NUC1 1H
P1 12.00 usec
PLW1 4.67290020 W
F2 - Processing parameters
SI 65536
SF 400.3000080 MHz
WDW EM
SSB 0
LB 0.30 Hz
GB 0
PC 1.00
1.544
1.578
1.585
1.901
1.911
1.937
1.955
1.965
1.968
1.989
1.998
2.372
2.376
2.400
2.413
2.438
2.443
3.576
3.603
3.630
3.654
3.668
3.683
3.752
3.766
3.768
3.780
3.793
3.795
3.809
3.919
3.927
3.939
3.970
3.997
4.012
4.019
4.030
4.079
4.086
4.089
4.097
4.108
4.113
4.119
4.126
4.130
4.677
4.688
4.763
4.770
1.19
2.03
0.99
1.04
2.01
1.08
3.10
0.03
1.00
0.02
ppm
4.75
ppm

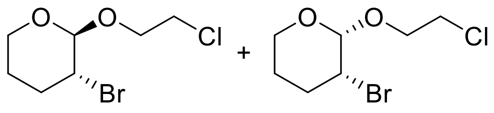

trans-**8b** *cis*-**8b**

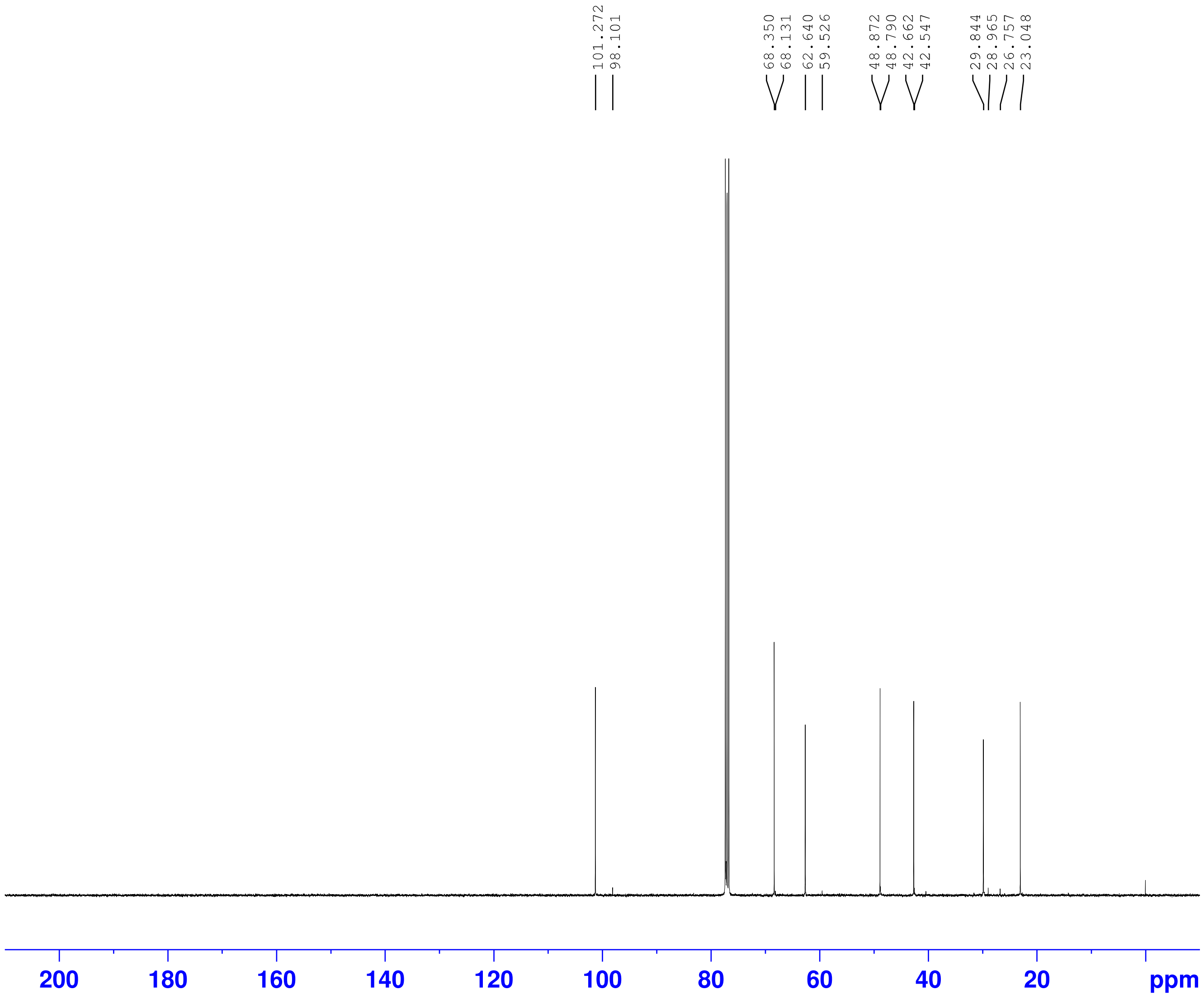


```
Current Data Parameters
NAME      kmd-5-127 Fr13-25
EXPNO                     3
PROCNO                    1

F2 - Acquisition Parameters
Date_              20210119
Time                19.36 h
INSTRUM               spect
PROBHD    Z150354_0001 (
PULPROG             zgpg30
TD                    65356
SOLVENT               CDCl3
NS                      256
DS                        4
SWH           24038.461 Hz
FIDRES         0.735616 Hz
AQ            1.3594048 sec
RG                    51.78
DW               20.800 usec
DE                25.00 usec
TE                298.0 K
D1           2.00000000 sec
D11          0.03000000 sec
TD0                       1
SFO1        100.6655806 MHz
NUC1                    13C
P1                10.00 usec
PLW1        19.75099945 W
SFO2        400.3016012 MHz
NUC2                     1H
CPDPRG[2           waltz16
PCPD2             80.00 usec
PLW2         4.67290020 W
PLW12        0.10514000 W
PLW13        0.05280100 W

F2 - Processing parameters
SI                    32768
SF          100.6555151 MHz
WDW                      EM
SSB       0
LB                 1.00 Hz
GB        0
PC                     1.40
```

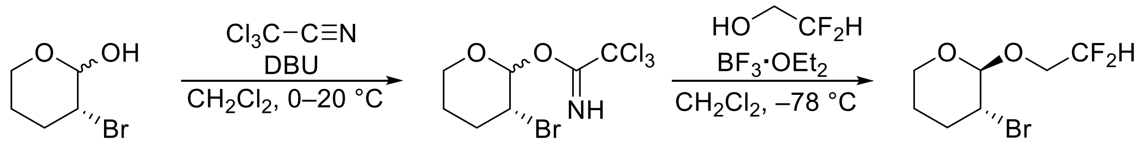

4 **6** **8c**

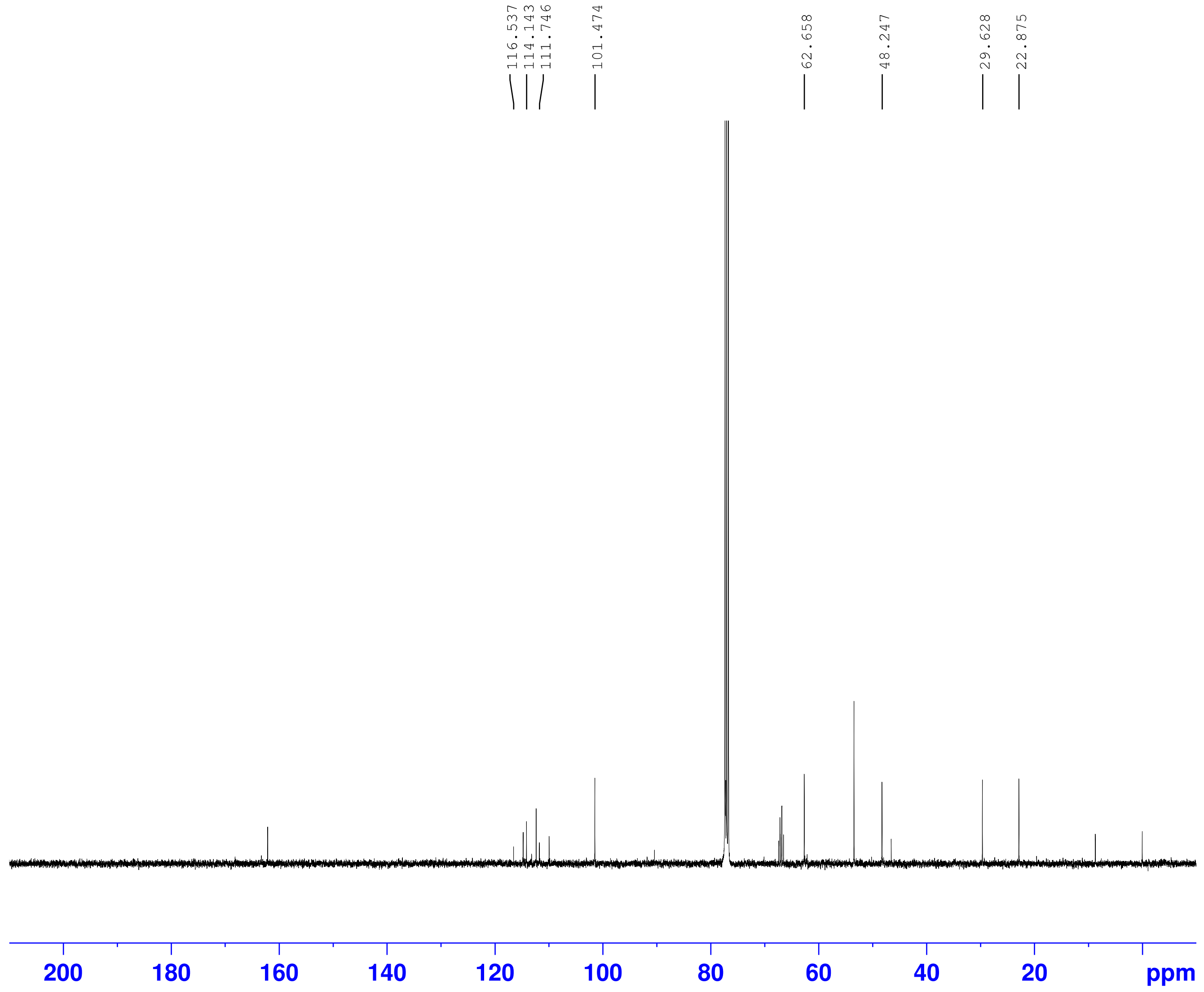


```
Current Data Parameters
NAME            kmd-5-128
EXPNO                   3
PROCNO                  1

F2 - Acquisition Parameters
Date_            20210118
Time                22.07 h
INSTRUM             spect
PROBHD    Z150354_0001 (
PULPROG           zgpg30
TD                 65356
SOLVENT            CDCl3
NS                   256
DS                     4
SWH            24038.461 Hz
FIDRES          0.735616 Hz
AQ             1.3594048 sec
RG                 25.77
DW                20.800 usec
DE                 25.00 usec
TE                 298.0 K
D1            2.00000000 sec
D11           0.03000000 sec
TD0                    1
SFO1        100.6655806 MHz
NUC1                 13C
P1                 10.00 usec
PLW1         19.75099945 W
SFO2        400.3016012 MHz
NUC2                  1H
CPDPRG[2         waltz16
PCPD2              80.00 usec
PLW2          4.67290020 W
PLW12         0.10514000 W
PLW13         0.05280100 W

F2 - Processing parameters
SI                 32768
SF          100.6555151 MHz
WDW                   EM
SSB      0
LB                  1.00 Hz
GB       0
PC                  1.40
```

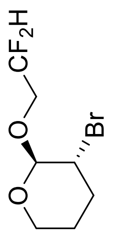

8c

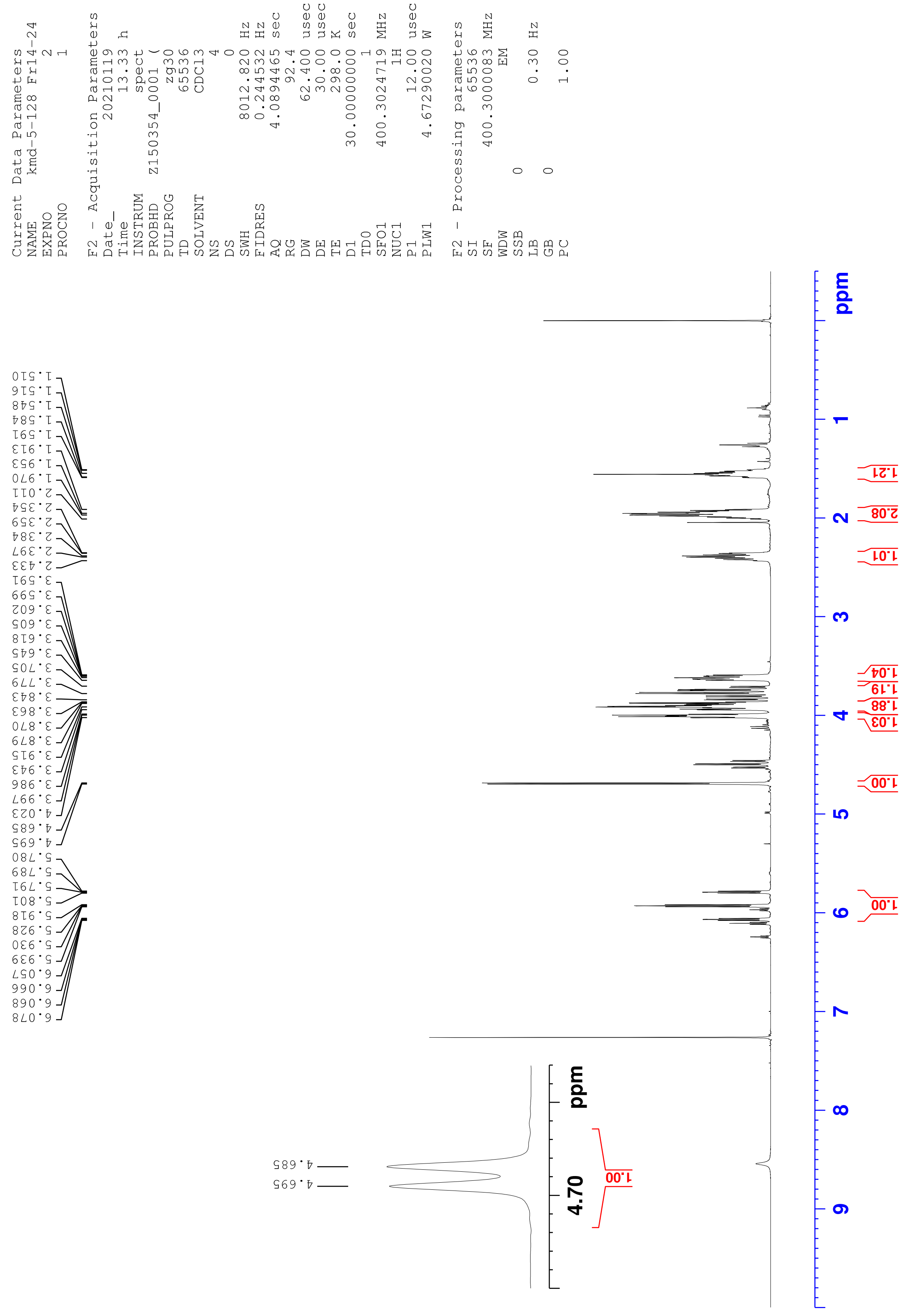
Current Data Parameters
NAME kmd-5-128 Fr14-24
EXPNO 2
PROCNO 1
F2 - Acquisition Parameters
Date_ 20210119
Time 13.33 h
INSTRUM spect
PROBHD Z150354_0001 (
PULPROG zg30
TD 65536
SOLVENT CDCl3
NS 4
DS 0
SWH 8012.820 Hz
FIDRES 0.244532 Hz
AQ 4.0894465 sec
RG 92.4
DW 62.400 usec
DE 30.00 usec
TE 298.0 K
D1 30.00000000 sec
TD0 1
SFO1 400.3024719 MHz
NUC1 1H
P1 12.00 usec
PLW1 4.67290020 W
F2 - Processing parameters
SI 65536
SF 400.3000083 MHz
WDW EM
SSB 0
LB 0.30 Hz
GB 0
PC 1.00
ppm
4.70
1.00

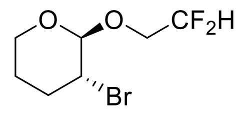

8c

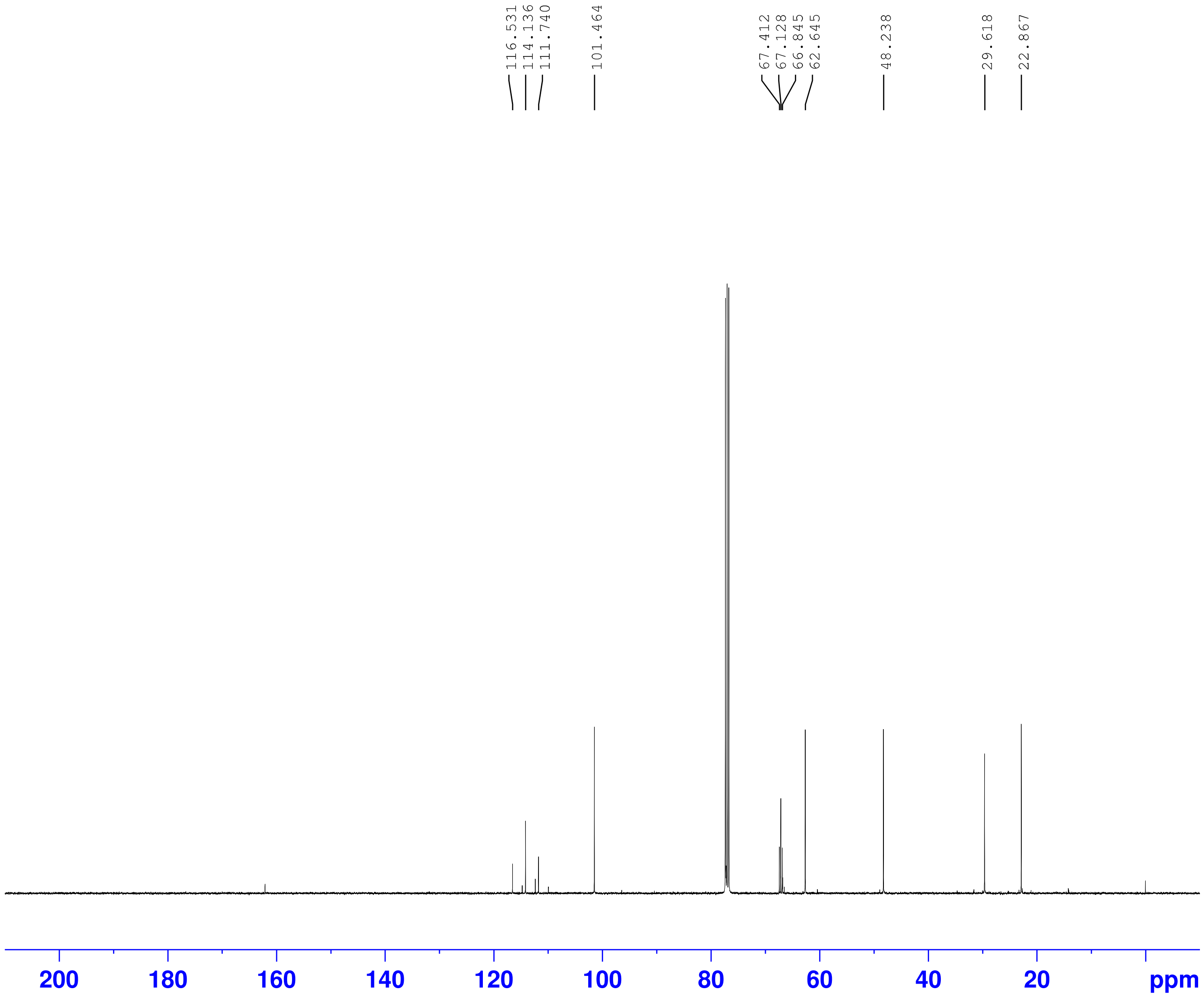


```
Current Data Parameters
NAME      kmd-5-128 Fr14-24
EXPNO                     3
PROCNO                    1

F2 - Acquisition Parameters
Date_              20210119
Time                  20.17 h
INSTRUM               spect
PROBHD    Z150354_0001 (
PULPROG             zgpg30
TD                   65356
SOLVENT              CDCl3
NS                     256
DS                       4
SWH              24038.461 Hz
FIDRES            0.735616 Hz
AQ               1.3594048 sec
RG                   45.21
DW                  20.800 usec
DE                   25.00 usec
TE                   298.0 K
D1              2.00000000 sec
D11             0.03000000 sec
TD0                      1
SFO1          100.6655806 MHz
NUC1                   13C
P1                   10.00 usec
PLW1           19.75099945 W
SFO2          400.3016012 MHz
NUC2                    1H
CPDPRG[2           waltz16
PCPD2                80.00 usec
PLW2            4.67290020 W
PLW12           0.10514000 W
PLW13           0.05280100 W

F2 - Processing parameters
SI                   32768
SF            100.6555157 MHz
WDW                     EM
SSB       0
LB                    1.00 Hz
GB        0
PC                    1.40
```

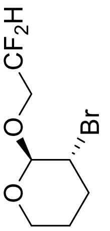

8c

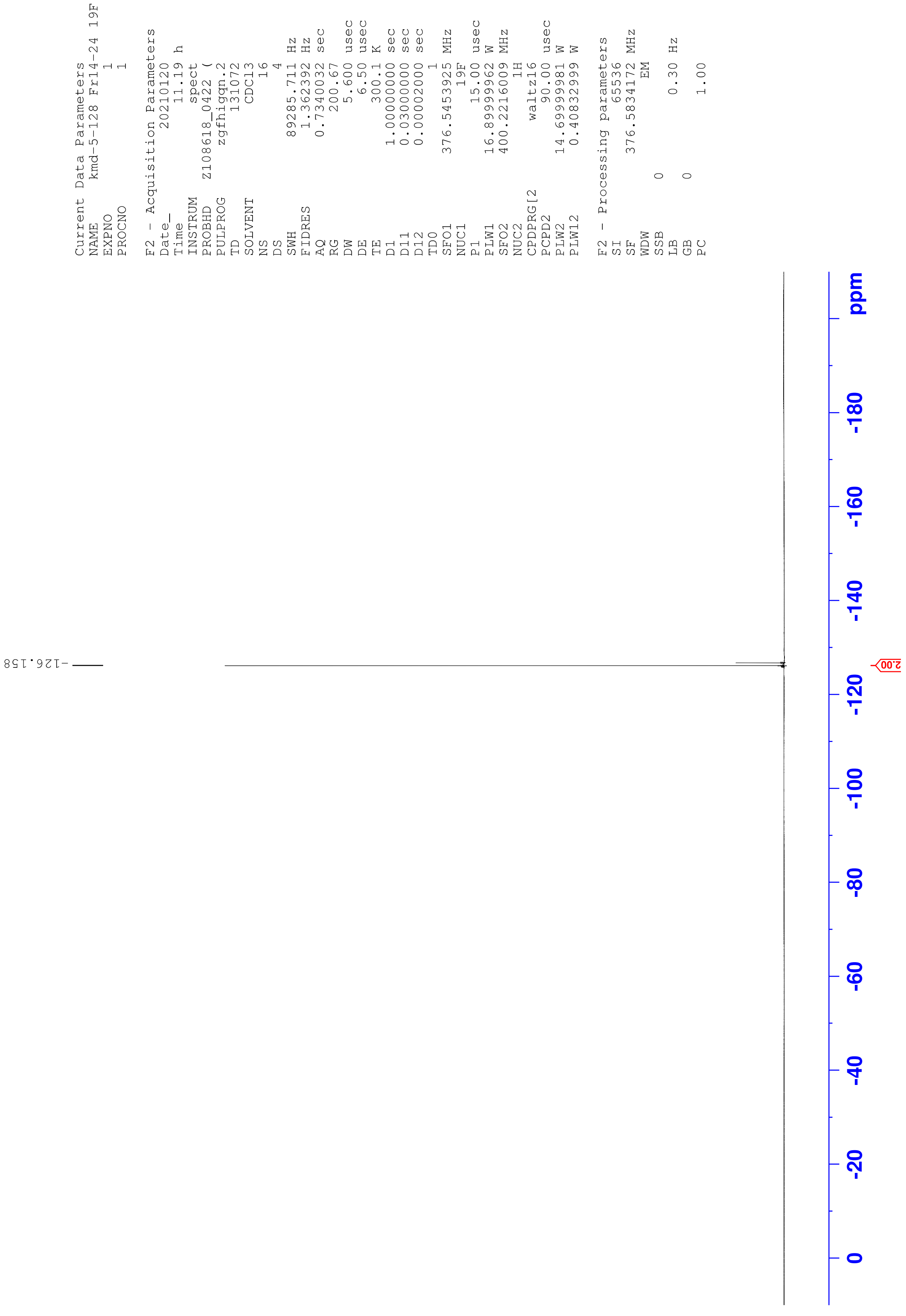
Current Data Parameters
NAME kmd-5-128 Fr14-24 19F
EXPNO 1
PROCNO 1
F2 - Acquisition Parameters
Date_ 20210120
Time 11.19 h
INSTRUM spect
PROBHD Z108618_0422 (
PULPROG zgfhigqn.2
TD 131072
SOLVENT CDCl3
NS 16
DS 4
SWH 89285.711 Hz
FIDRES 1.362392 Hz
AQ 0.7340032 sec
RG 200.67
DW 5.600 usec
DE 6.50 usec
TE 300.1 K
D1 1.00000000 sec
D11 0.03000000 sec
D12 0.00002000 sec
TD0 1
SFO1 376.5453925 MHz
NUC1 19F
P1 15.00 usec
PLW1 16.89999962 W
SFO2 400.2216009 MHz
NUC2 1H
CPDPRG[2 waltz16
PCPD2 90.00 usec
PLW2 14.69999981 W
PLW12 0.40832999 W
F2 - Processing parameters
SI 65536
SF 376.5834172 MHz
WDW EM
SSB 0
LB 0.30 Hz
GB 0
PC 1.00
-126.158
2.00
ppm
-180
-160
-140
-120
-100
-80
-60
-40
-20
0

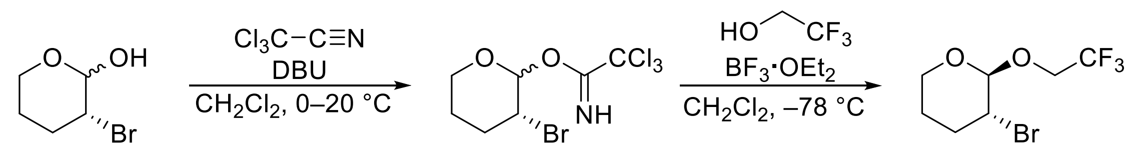

4 **6** **8d**

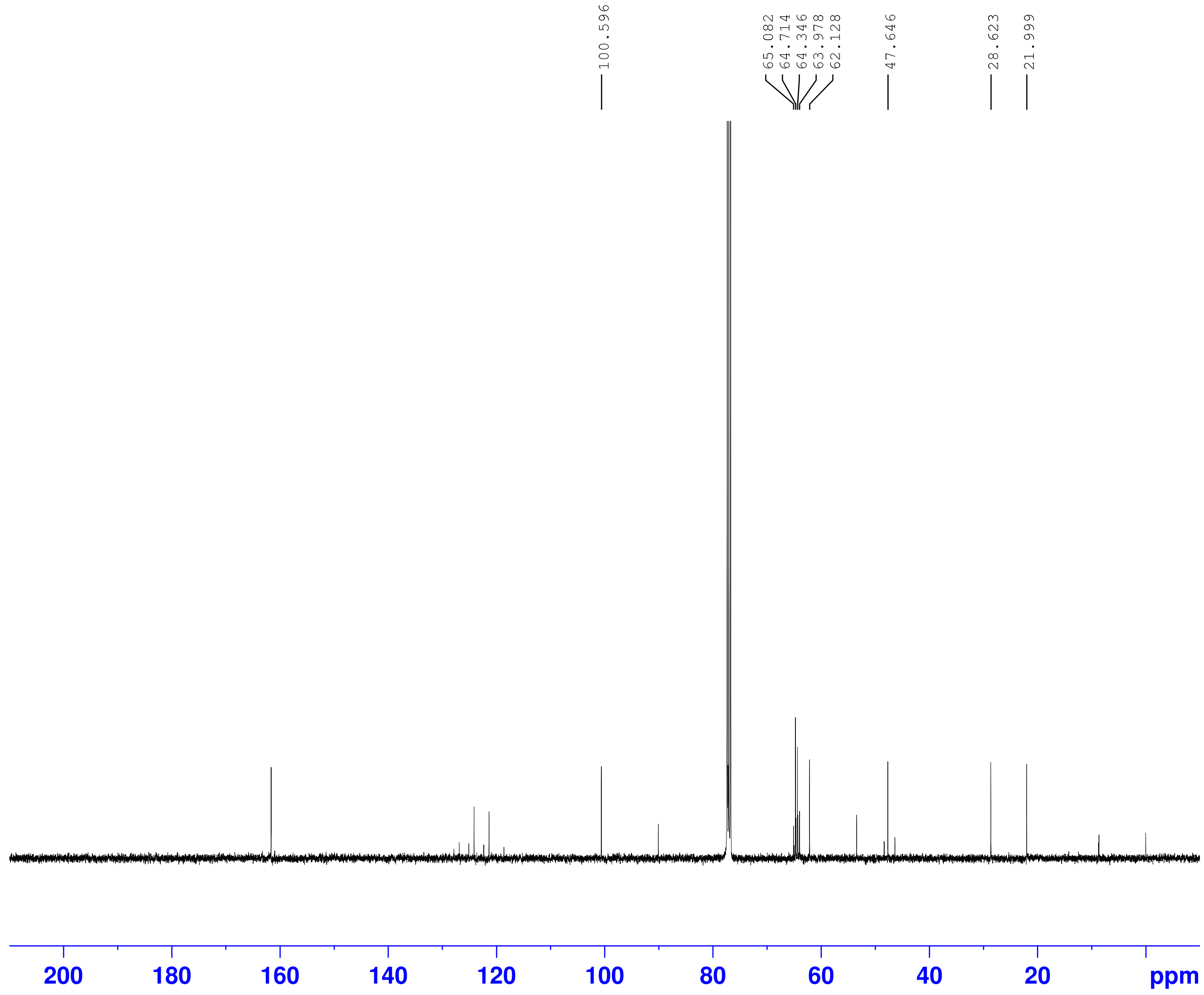


```
Current Data Parameters
NAME          kmd-5-129
EXPNO                 3
PROCNO                1

F2 - Acquisition Parameters
Date_          20210119
Time               2.29 h
INSTRUM           spect
PROBHD    Z150354_0001 (
PULPROG          zgpg30
TD                65356
SOLVENT           CDCl3
NS                  256
DS                    4
SWH           24038.461 Hz
FIDRES         0.735616 Hz
AQ            1.3594048 sec
RG                 31.4
DW               20.800 usec
DE                25.00 usec
TE                298.0 K
D1           2.00000000 sec
D11          0.03000000 sec
TD0                   1
SFO1        100.6655806 MHz
NUC1                13C
P1                10.00 usec
PLW1        19.75099945 W
SFO2        400.3016012 MHz
NUC2                 1H
CPDPRG[2        waltz16
PCPD2             80.00 usec
PLW2         4.67290020 W
PLW12        0.10514000 W
PLW13        0.05280100 W

F2 - Processing parameters
SI                32768
SF          100.6555151 MHz
WDW                  EM
SSB      0
LB                 1.00 Hz
GB       0
PC                 1.40
```

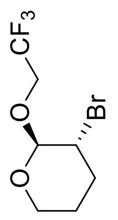
O
O
CF3
Br

8d

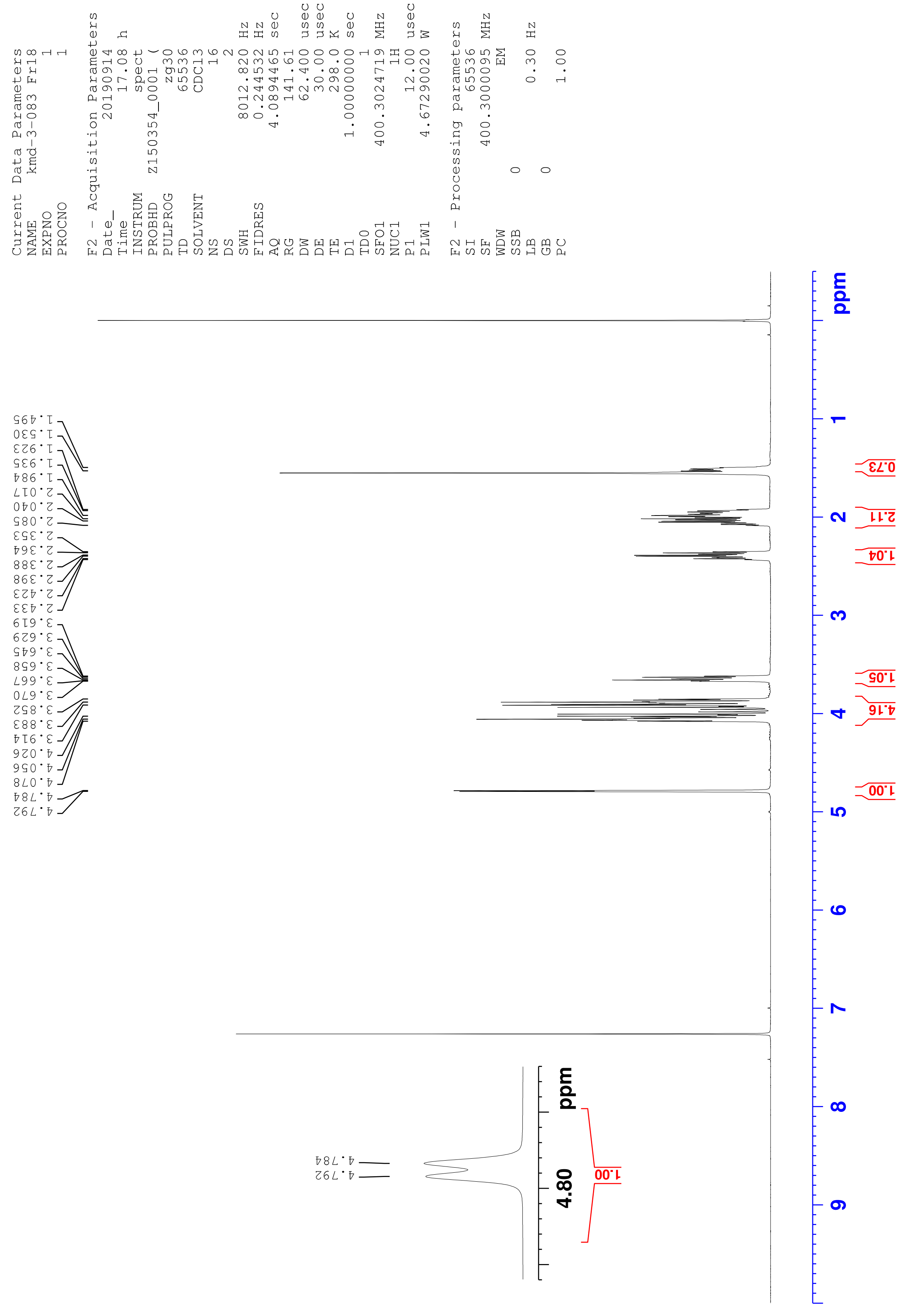
Current Data Parameters
NAME kmd-3-083 Fr18
EXPNO 1
PROCNO 1
F2 - Acquisition Parameters
Date_ 20190914
Time 17.08 h
INSTRUM spect
PROBHD Z150354_0001 (
PULPROG zg30
TD 65536
SOLVENT CDCl3
NS 16
DS 2
SWH 8012.820 Hz
FIDRES 0.244532 Hz
AQ 4.0894465 sec
RG 141.61
DW 62.400 usec
DE 30.00 usec
TE 298.0 K
D1 1.00000000 sec
TD0 1
SFO1 400.3024719 MHz
NUC1 1H
P1 12.00 usec
PLW1 4.67290020 W
F2 - Processing parameters
SI 65536
SF 400.3000095 MHz
WDW EM
SSB 0
LB 0.30 Hz
GB 0
PC 1.00
1.495
1.530
1.923
1.935
1.984
2.017
2.040
2.085
2.353
2.364
2.388
2.398
2.423
2.433
3.619
3.629
3.645
3.658
3.667
3.670
3.852
3.883
3.914
4.026
4.056
4.078
4.784
4.792
0.73
2.11
1.04
1.05
4.16
1.00
ppm
1
2
3
4
5
6
7
8
9
4.784
4.792
4.80
ppm
1.00

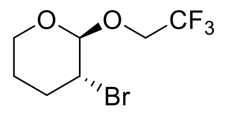

8d

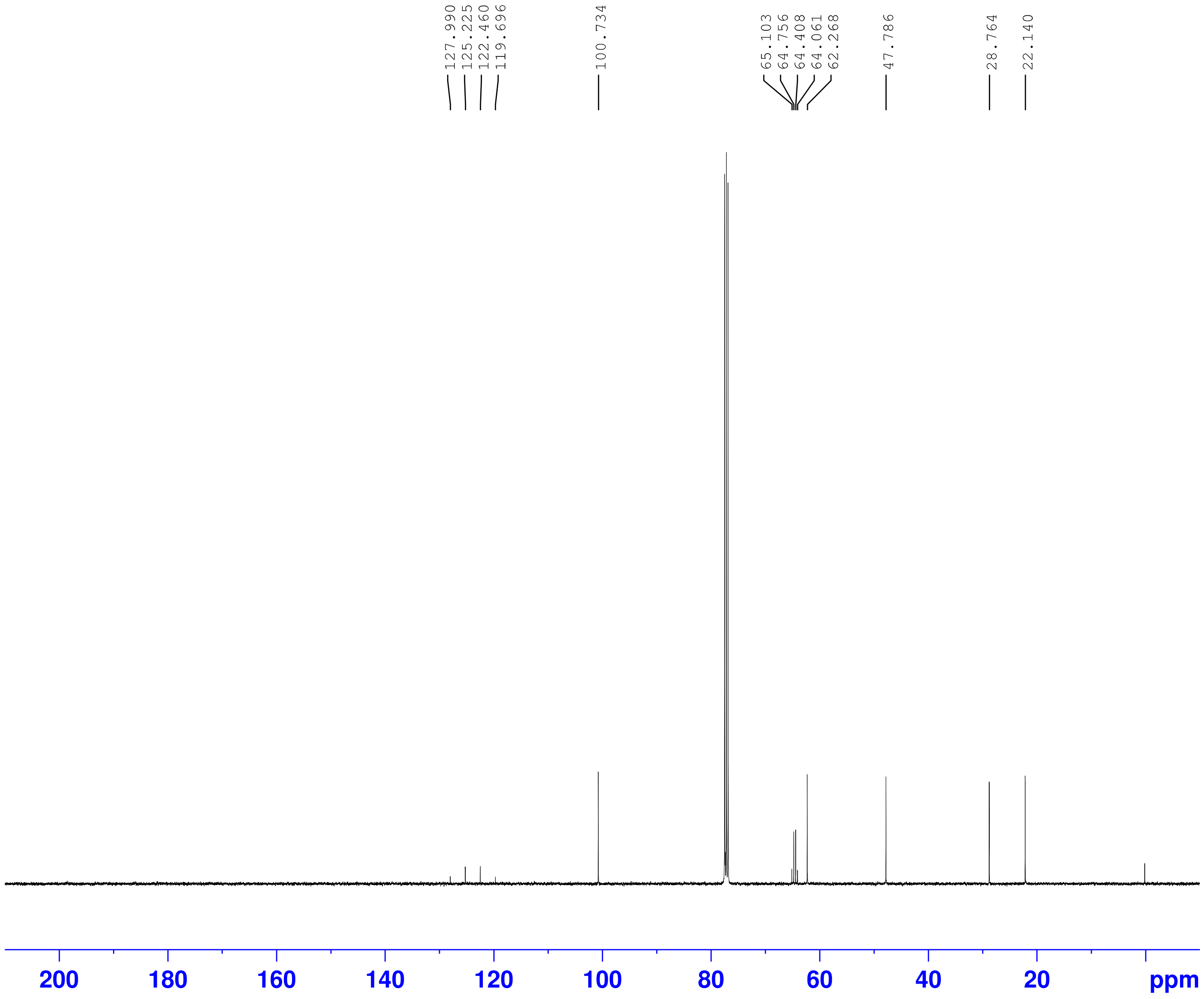


```
Current Data Parameters
NAME        kmd-3-083 Fr18
EXPNO                    3
PROCNO                   1

F2 - Acquisition Parameters
Date_             20190914
Time                 18.01 h
INSTRUM              spect
PROBHD       Z150354_0001 (
PULPROG            zgpg30
TD                   65356
SOLVENT              CDCl3
NS                     256
DS                       4
SWH              24038.461 Hz
FIDRES            0.735616 Hz
AQ               1.3594048 sec
RG                   29.21
DW                  20.800 usec
DE                   25.00 usec
TE                   298.0 K
D1              2.00000000 sec
D11             0.03000000 sec
TD0                      1
SFO1           100.6655806 MHz
NUC1                   13C
P1                   10.00 usec
PLW1           19.75099945 W
SFO2           400.3016012 MHz
NUC2                    1H
CPDPRG[2           waltz16
PCPD2                80.00 usec
PLW2            4.67290020 W
PLW12           0.10514000 W
PLW13           0.05280100 W

F2 - Processing parameters
SI                   32768
SF             100.6555012 MHz
WDW                     EM
SSB        0
LB                    1.00 Hz
GB         0
PC                    1.40
```

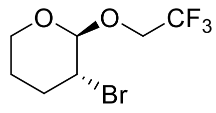

8d

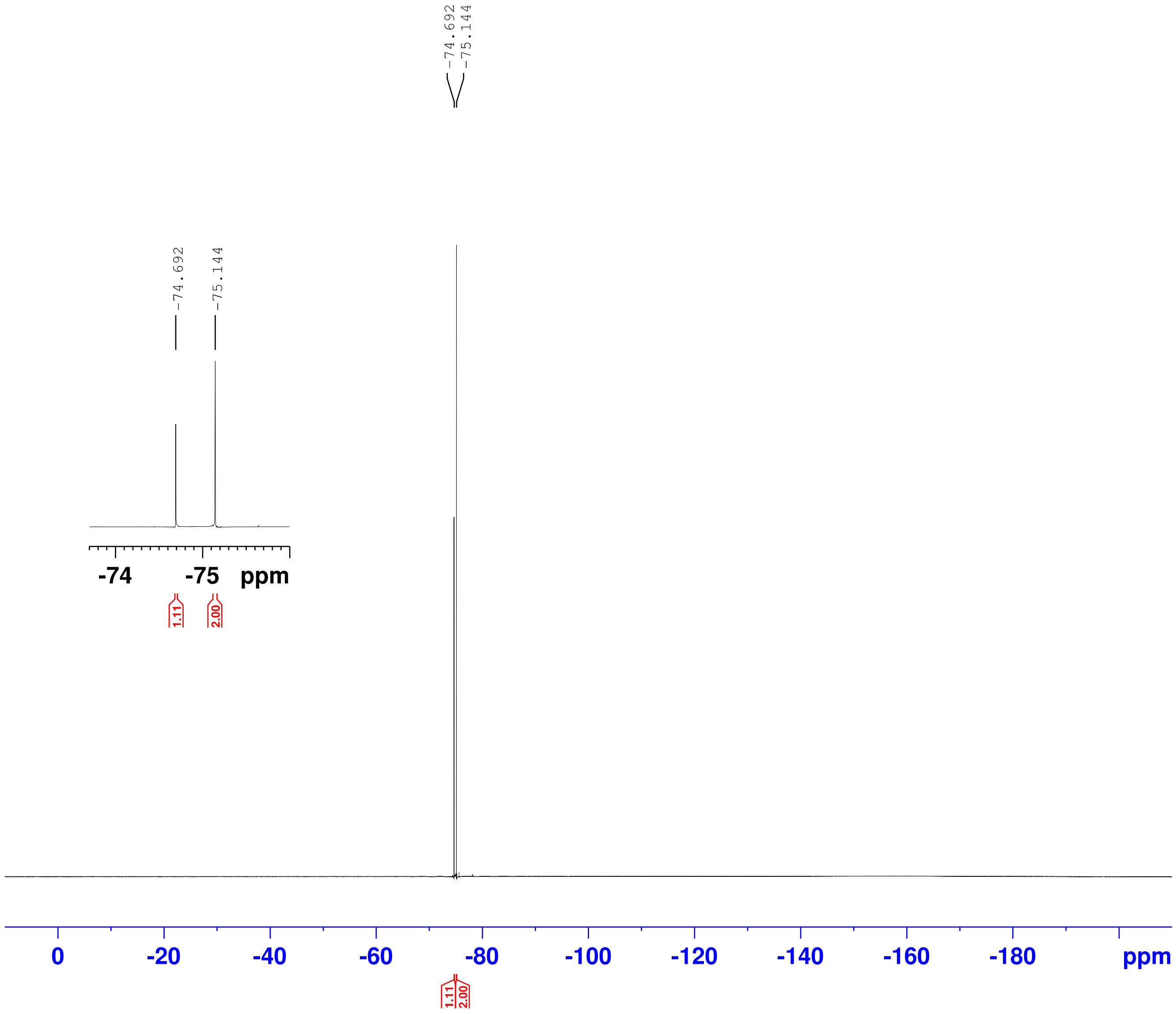


```
Current Data Parameters
NAME      kmd-5-129 Fr13-19 19F
EXPNO                    1
PROCNO                   1

F2 - Acquisition Parameters
Date_             20210120
Time                 11.28 h
INSTRUM              spect
PROBHD     Z108618_0422 (
PULPROG        zgfhigqn.2
TD                  131072
SOLVENT              CDCl3
NS                      16
DS                       4
SWH              89285.711 Hz
FIDRES            1.362392 Hz
AQ               0.7340032 sec
RG                  200.67
DW                   5.600 usec
DE                    6.50 usec
TE                   300.1 K
D1              1.00000000 sec
D11             0.03000000 sec
D12             0.00002000 sec
TD0                      1
SFO1           376.5453925 MHz
NUC1                   19F
P1                   15.00 usec
PLW1           16.89999962 W
SFO2           400.2216009 MHz
NUC2                    1H
CPDPRG[2           waltz16
PCPD2                90.00 usec
PLW2           14.69999981 W
PLW12           0.40832999 W

F2 - Processing parameters
SI                   65536
SF             376.5834174 MHz
WDW                     EM
SSB       0
LB                    0.30 Hz
GB        0
PC                    1.00
```

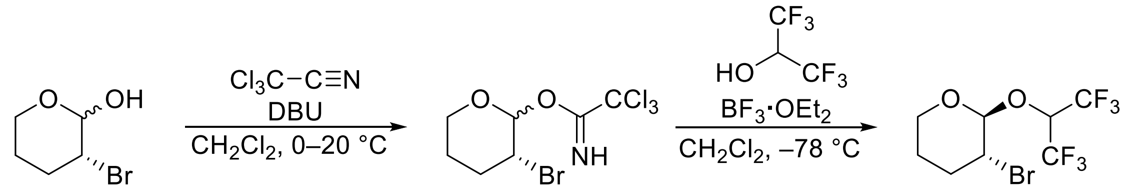

4 **6** **8e**

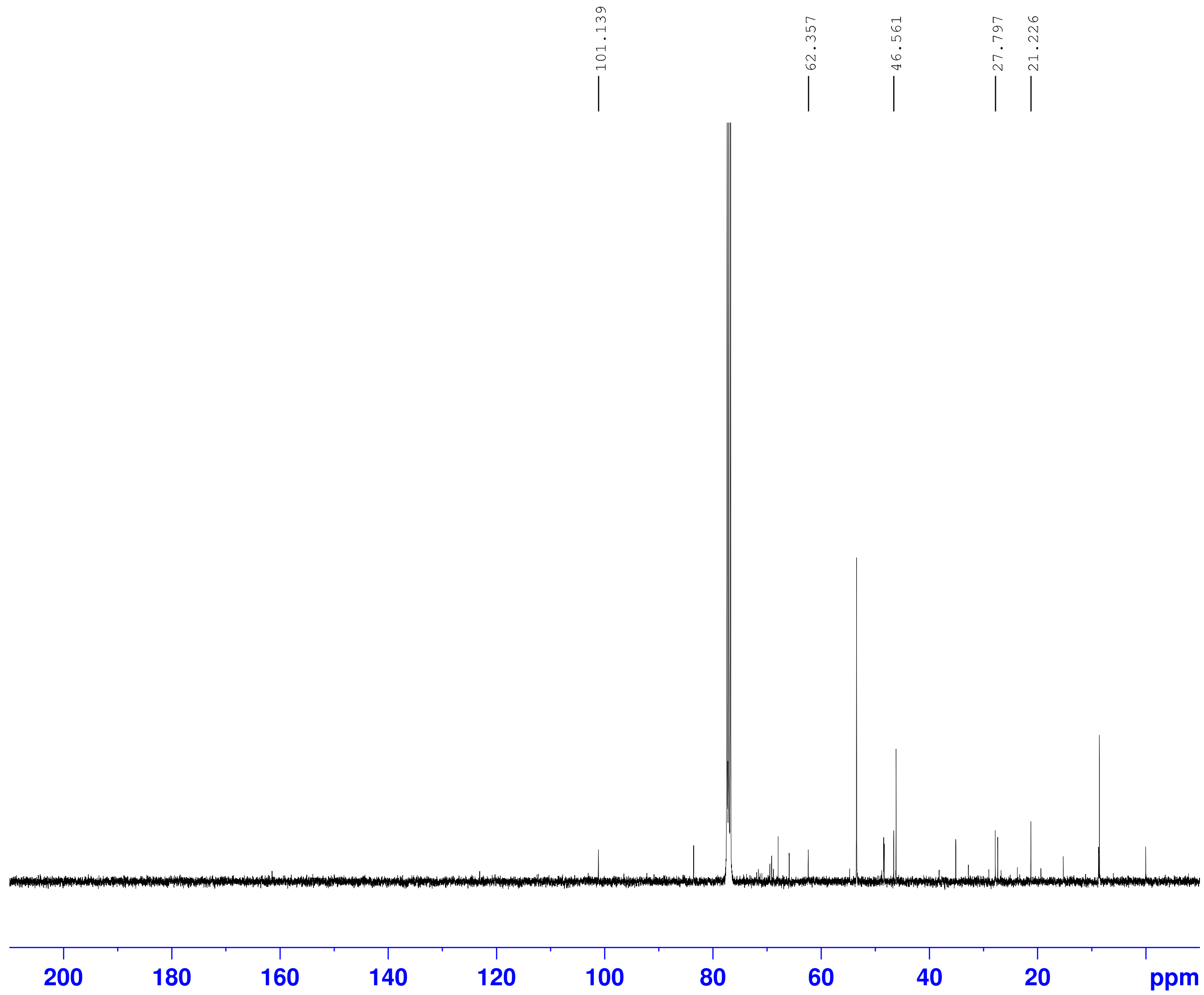


```
Current Data Parameters
NAME              kmd-5-130
EXPNO                     3
PROCNO                    1

F2 - Acquisition Parameters
Date_              20210119
Time                   2.52 h
INSTRUM               spect
PROBHD     Z150354_0001 (
PULPROG             zgpg30
TD                   65356
SOLVENT              CDCl3
NS                     256
DS                       4
SWH              24038.461 Hz
FIDRES            0.735616 Hz
AQ               1.3594048 sec
RG                   51.78
DW                  20.800 usec
DE                   25.00 usec
TE                   298.0 K
D1              2.00000000 sec
D11             0.03000000 sec
TD0                      1
SFO1          100.6655806 MHz
NUC1                   13C
P1                   10.00 usec
PLW1           19.75099945 W
SFO2          400.3016012 MHz
NUC2                    1H
CPDPRG[2           waltz16
PCPD2                80.00 usec
PLW2            4.67290020 W
PLW12           0.10514000 W
PLW13           0.05280100 W

F2 - Processing parameters
SI                   32768
SF            100.6555151 MHz
WDW                     EM
SSB       0
LB                    1.00 Hz
GB        0
PC                    1.40
```

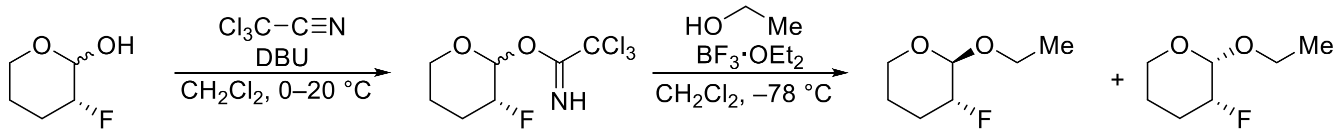

9 | 1a | *trans*-10a + *cis*-10a

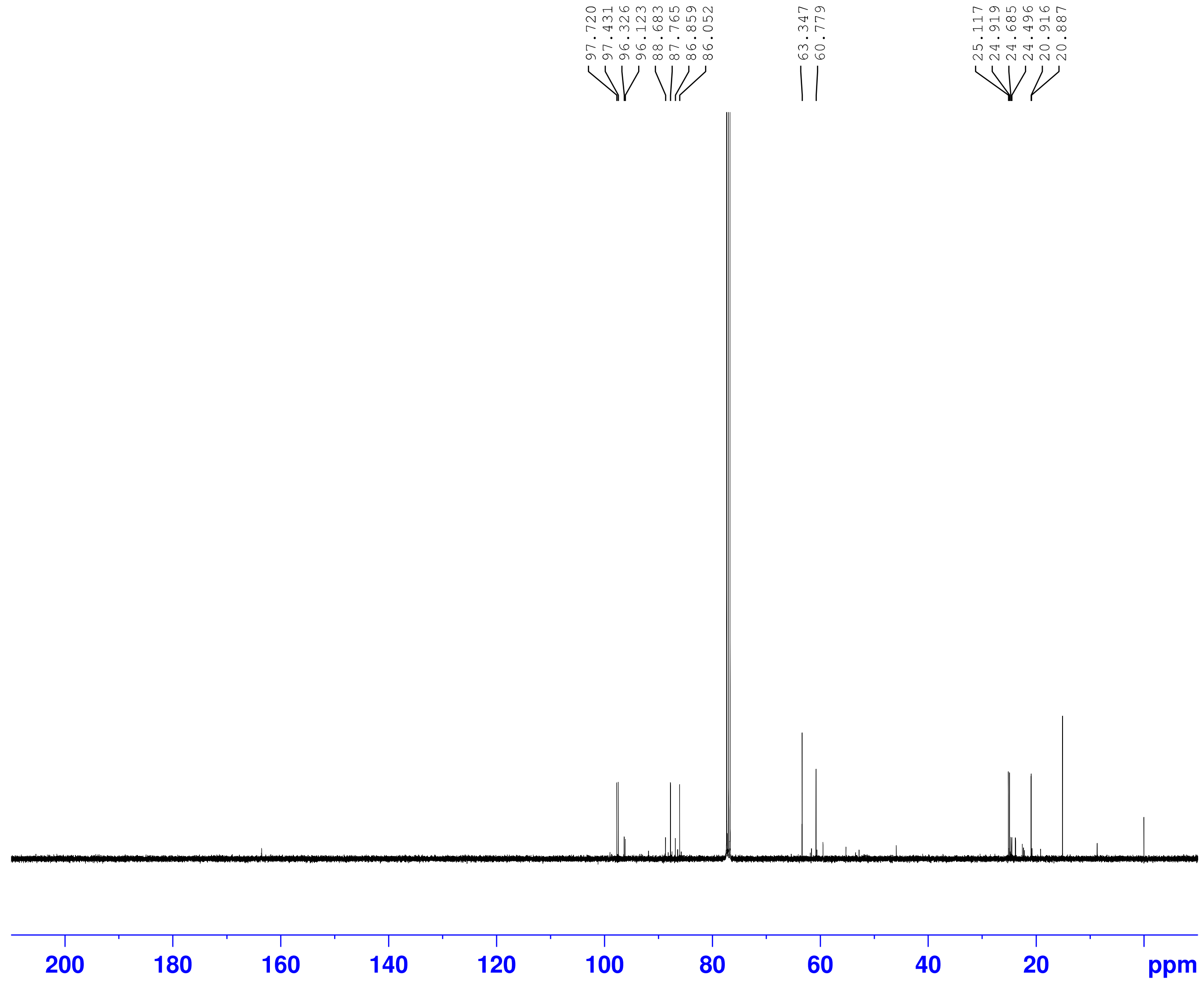


```
Current Data Parameters
NAME            kmd-5-039
EXPNO                   3
PROCNO                  1

F2 - Acquisition Parameters
Date_            20201101
Time                17.33 h
INSTRUM             spect
PROBHD    Z150354_0001 (
PULPROG           zgpg30
TD                 65356
SOLVENT            CDCl3
NS                   256
DS                     4
SWH            24038.461 Hz
FIDRES          0.735616 Hz
AQ             1.3594048 sec
RG                 29.21
DW                20.800 usec
DE                 25.00 usec
TE                 298.0 K
D1            2.00000000 sec
D11           0.03000000 sec
TD0                    1
SFO1         100.6655806 MHz
NUC1                 13C
P1                 10.00 usec
PLW1         19.75099945 W
SFO2         400.3016012 MHz
NUC2                  1H
CPDPRG[2         waltz16
PCPD2              80.00 usec
PLW2          4.67290020 W
PLW12         0.10514000 W
PLW13         0.05280100 W

F2 - Processing parameters
SI                131072
SF           100.6555151 MHz
WDW                   EM
SSB      0
LB       0 Hz
GB       0
PC                  1.40
```

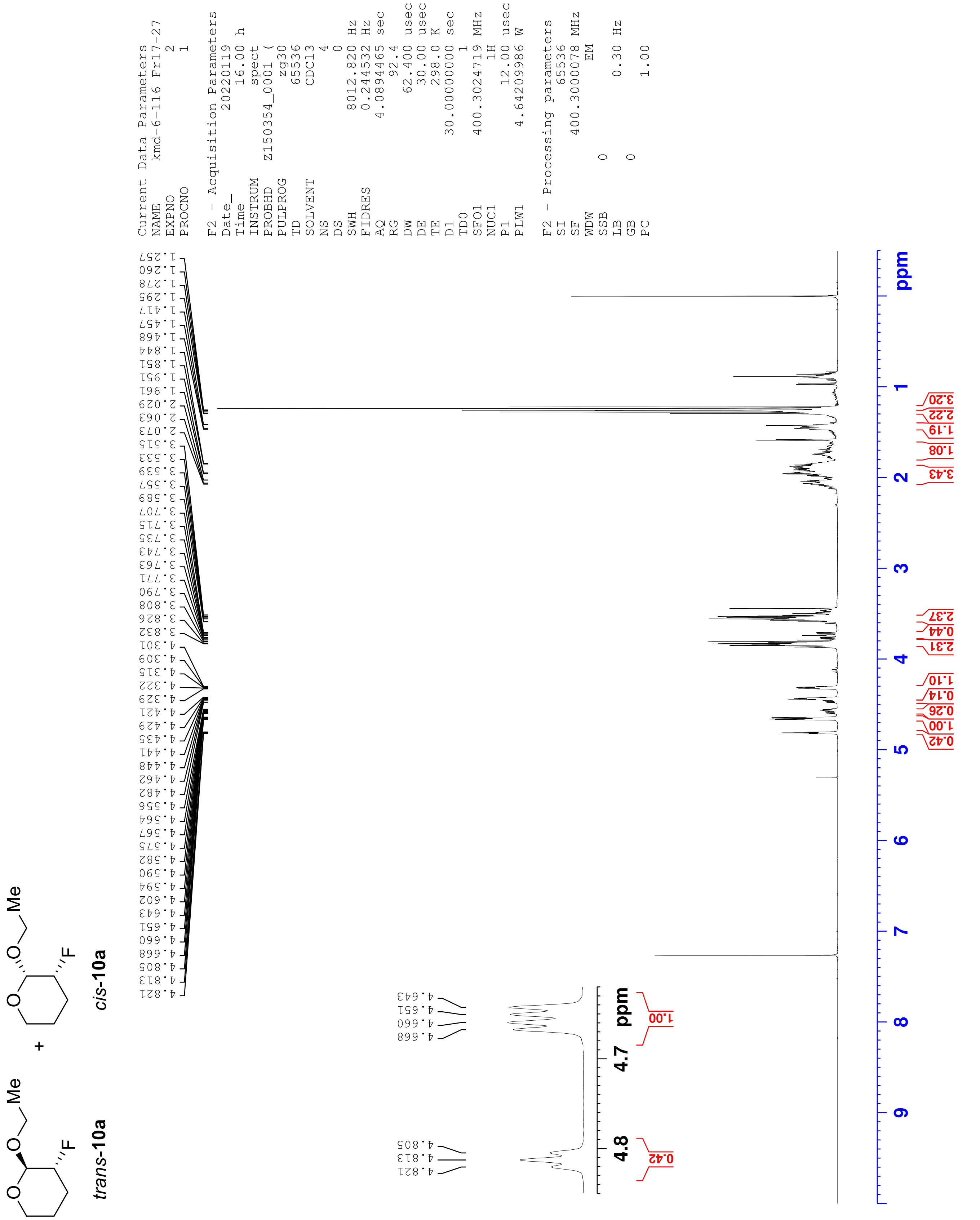
trans-10a
cis-10a
Me
Me
O
O
O
O
F
F
+
Current Data Parameters
NAME kmd-6-116 Fr17-27
EXPNO 2
PROCNO 1
F2 - Acquisition Parameters
Date_ 20220119
Time_ 16.00 h
INSTRUM spect
PROBHD Z150354_0001 (
PULPROG zg30
TD 65536
SOLVENT CDCl3
NS 4
DS 0
SWH 8012.820 Hz
FIDRES 0.244532 Hz
AQ 4.0894465 sec
RG 92.4
DW 62.400 usec
DE 30.00 usec
TE 298.0 K
D1 30.00000000 sec
TD0 1
SFO1 400.3024719 MHz
NUC1 1H
P1 12.00 usec
PLW1 4.64209986 W
F2 - Processing parameters
SI 65536
SF 400.3000078 MHz
WDW EM
SSB 0
LB 0.30 Hz
GB 0
PC 1.00
1.257
1.260
1.278
1.295
1.417
1.457
1.468
1.844
1.851
1.951
1.961
2.029
2.063
2.073
3.515
3.533
3.539
3.557
3.589
3.707
3.715
3.735
3.743
3.763
3.771
3.790
3.808
3.826
3.832
4.301
4.309
4.315
4.322
4.329
4.421
4.429
4.435
4.441
4.448
4.462
4.482
4.556
4.564
4.567
4.575
4.582
4.590
4.594
4.602
4.643
4.651
4.660
4.668
4.805
4.813
4.821
4.643
4.651
4.660
4.668
4.805
4.813
4.821
4.8
4.7
ppm
0.42
1.00
3.20
2.22
1.19
1.08
3.43
2.37
0.44
2.31
1.10
0.14
0.26
1.00
0.42
ppm
1
2
3
4
5
6
7
8
9

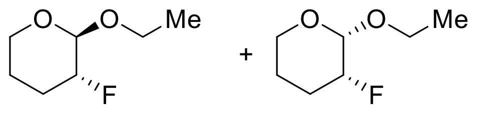

trans-**10a** + *cis*-**10a**

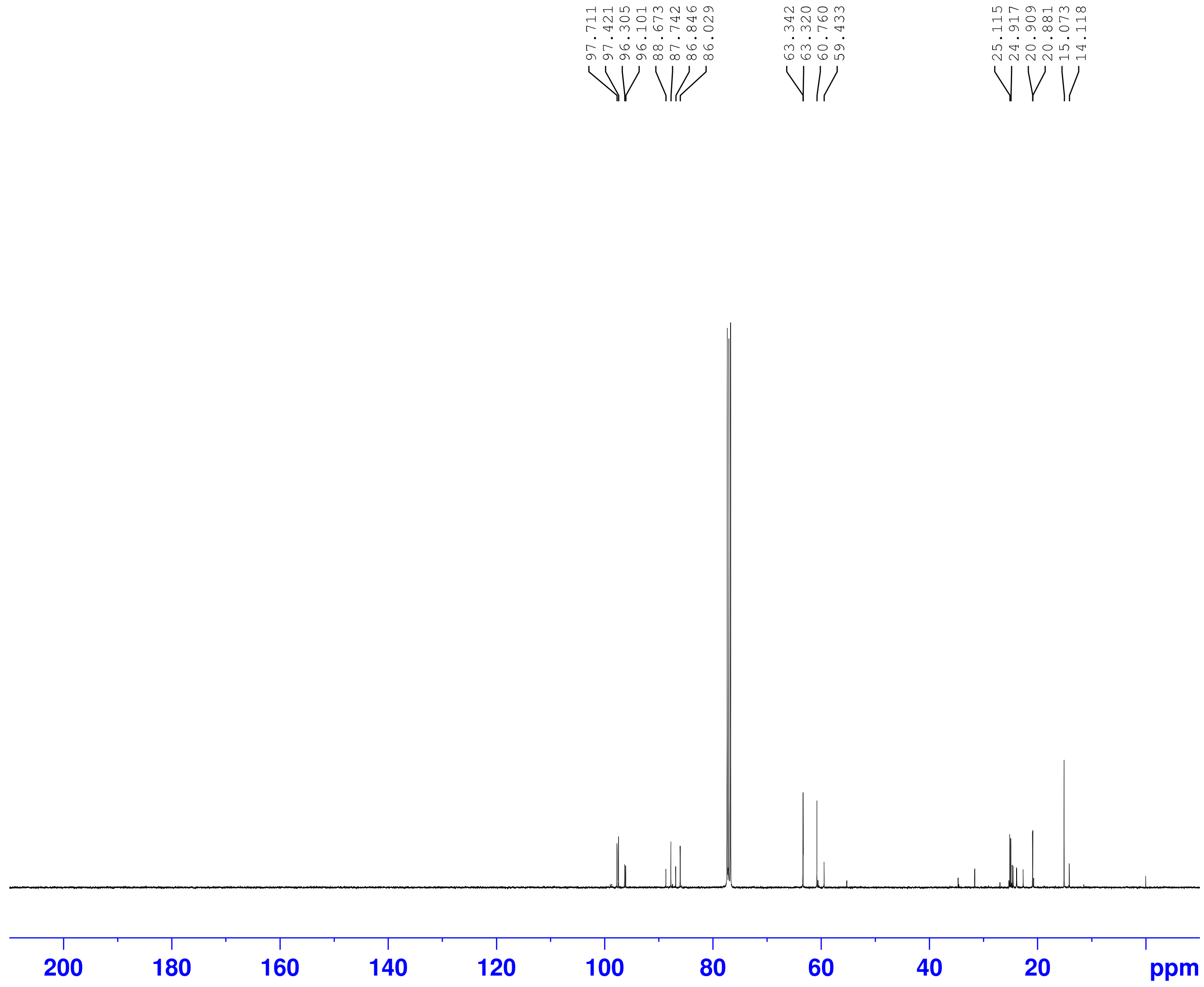


```
Current Data Parameters
NAME      kmd-6-116 Fr17-27
EXPNO                  3
PROCNO                 1

F2 - Acquisition Parameters
Date_           20220119
Time               20.20 h
INSTRUM            spect
PROBHD    Z150354_0001 (
PULPROG           zgpg30
TD                 65356
SOLVENT            CDCl3
NS                   256
DS                     4
SWH            24038.461 Hz
FIDRES          0.735616 Hz
AQ             1.3594048 sec
RG                 29.21
DW                20.800 usec
DE                 25.00 usec
TE                 298.0 K
D1            2.00000000 sec
D11           0.03000000 sec
TD0                    1
SFO1         100.6655806 MHz
NUC1                 13C
P1                 10.00 usec
PLW1         17.99699974 W
SFO2         400.3016012 MHz
NUC2                  1H
CPDPRG[2         waltz16
PCPD2              80.00 usec
PLW2          4.64209986 W
PLW12         0.10445000 W
PLW13         0.05245300 W

F2 - Processing parameters
SI                 32768
SF           100.6555153 MHz
WDW                   EM
SSB       0
LB                  1.00 Hz
GB        0
PC                  1.40
```

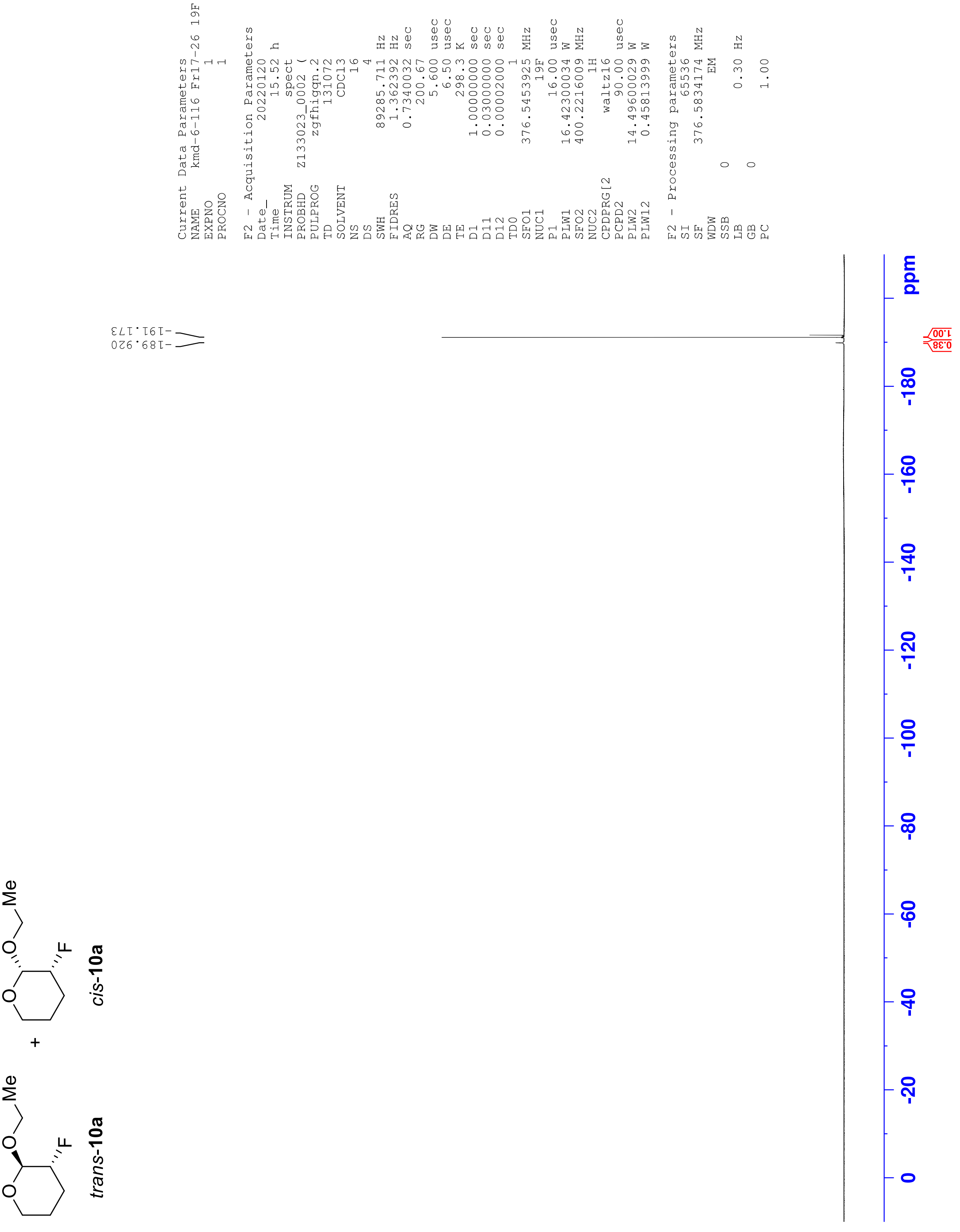
Me
O
O
F
trans-10a
+
Me
O
O
F
cis-10a
-189.920
-191.173
0.38
1.00
0
-20
-40
-60
-80
-100
-120
-140
-160
-180
ppm
Current Data Parameters
NAME kmd-6-116 Fr17-26 19F
EXPNO 1
PROCNO 1
F2 - Acquisition Parameters
Date_ 20220120
Time 15.52 h
INSTRUM spect
PROBHD Z133023_0002 (
PULPROG zgfhigqn.2
TD 131072
SOLVENT CDCl3
NS 16
DS 4
SWH 89285.711 Hz
FIDRES 1.362392 Hz
AQ 0.7340032 sec
RG 200.67
DW 5.600 usec
DE 6.50 usec
TE 298.3 K
D1 1.00000000 sec
D11 0.03000000 sec
D12 0.00002000 sec
TD0 1
SFO1 376.5453925 MHz
NUC1 19F
P1 16.00 usec
PLW1 16.42300034 W
SFO2 400.2216009 MHz
NUC2 1H
CPDPRG[2 waltz16
PCPD2 90.00 usec
PLW2 14.49600029 W
PLW12 0.45813999 W
F2 - Processing parameters
SI 65536
SF 376.5834174 MHz
WDW EM
SSB 0
LB 0.30 Hz
GB 0
PC 1.00

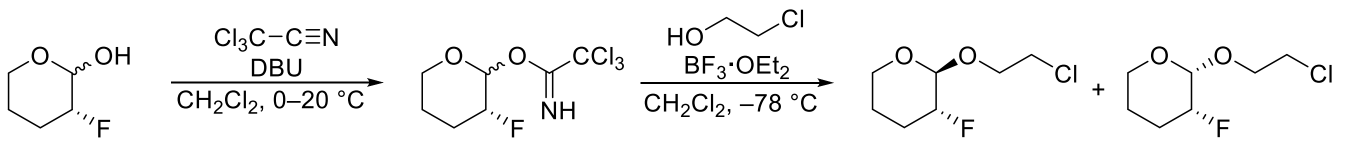

9 1a *trans*-**10b** *cis*-**10b**

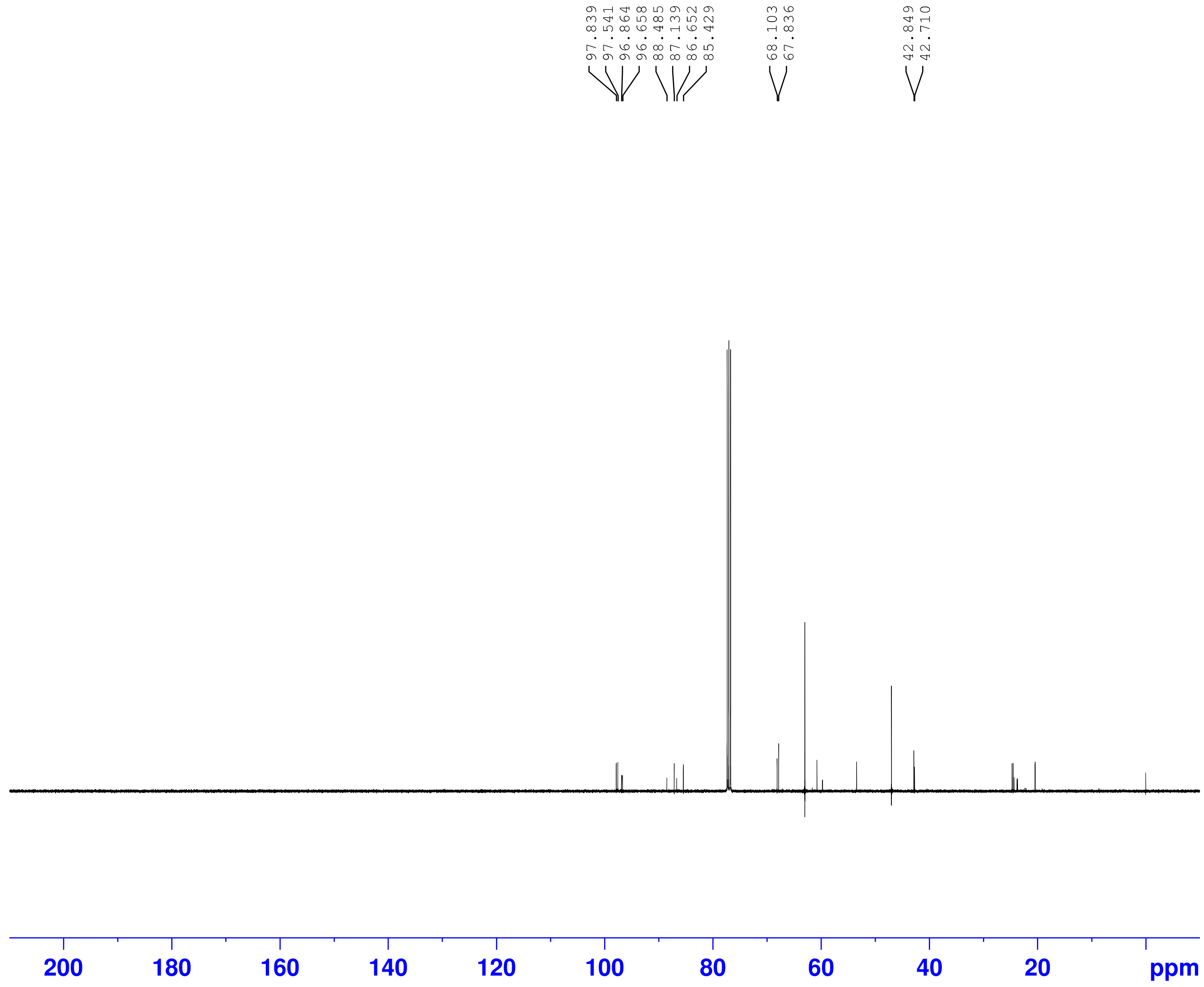


```
Current Data Parameters
NAME             kmd-6-117
EXPNO                    3
PROCNO                   1

F2 - Acquisition Parameters
Date_             20220119
Time                 19.57 h
INSTRUM              spect
PROBHD   Z150354_0001 (
PULPROG            zgpg30
TD                   65356
SOLVENT              CDCl3
NS                     256
DS                       4
SWH              24038.461 Hz
FIDRES            0.735616 Hz
AQ               1.3594048 sec
RG                   51.78
DW                  20.800 usec
DE                   25.00 usec
TE                   298.0 K
D1              2.00000000 sec
D11             0.03000000 sec
TD0                      1
SFO1          100.6655806 MHz
NUC1                   13C
P1                   10.00 usec
PLW1           17.99699974 W
SFO2          400.3016012 MHz
NUC2                    1H
CPDPRG[2           waltz16
PCPD2                80.00 usec
PLW2            4.64209986 W
PLW12           0.10445000 W
PLW13           0.05245300 W

F2 - Processing parameters
SI                  131072
SF            100.6555158 MHz
WDW                     EM
SSB      0
LB       0 Hz
GB       0
PC                    1.40
```

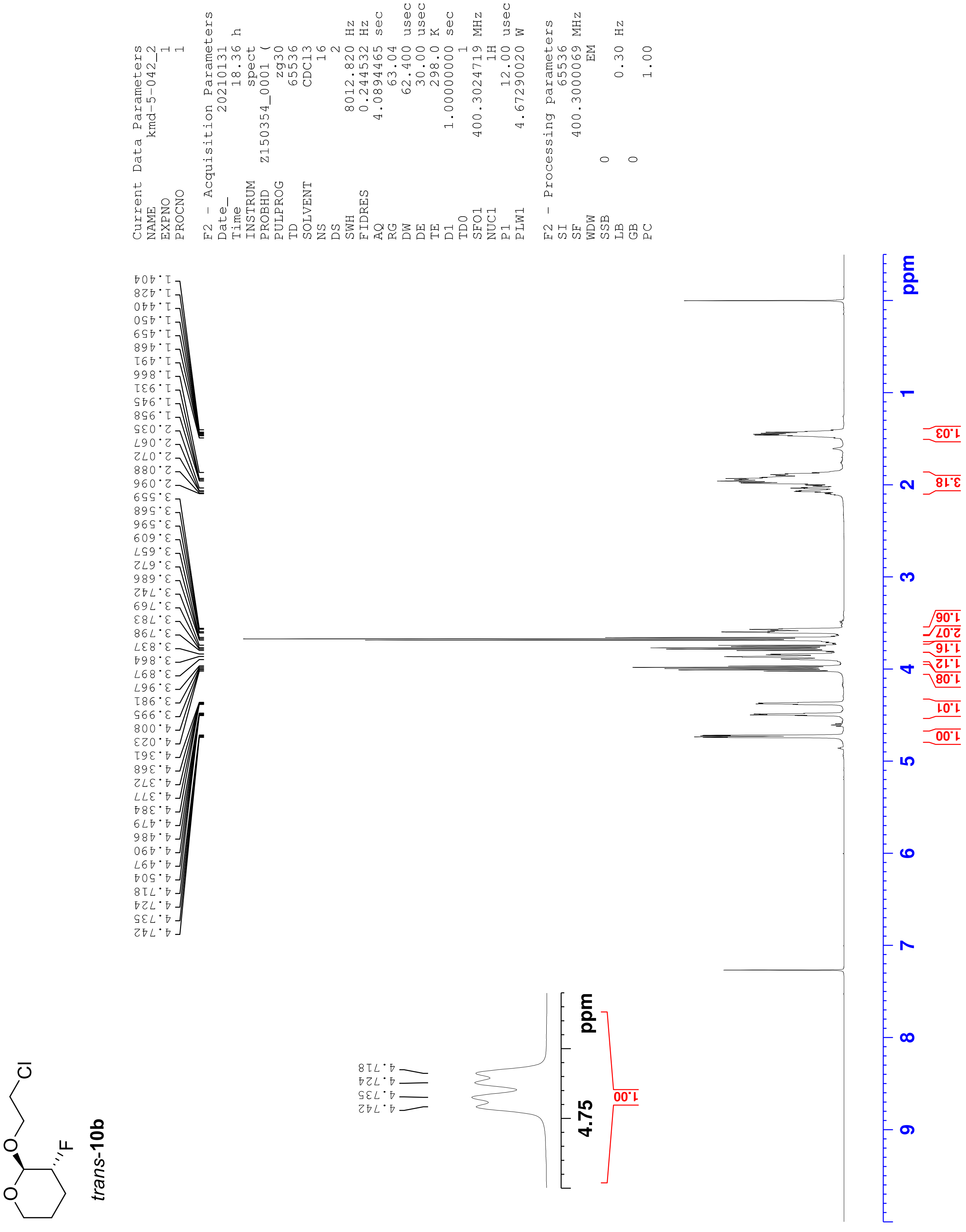
Cl
O
O
F
trans-10b
Current Data Parameters
NAME kmd-5-042_2
EXPNO 1
PROCNO 1
F2 - Acquisition Parameters
Date_ 20210131
Time 18.36 h
INSTRUM spect
PROBHD Z150354_0001 (
PULPROG zg30
TD 65536
SOLVENT CDCl3
NS 16
DS 2
SWH 8012.820 Hz
FIDRES 0.244532 Hz
AQ 4.0894465 sec
RG 63.04
DW 62.400 usec
DE 30.00 usec
TE 298.0 K
D1 1.00000000 sec
TD0 1
SFO1 400.3024719 MHz
NUC1 1H
P1 12.00 usec
PLW1 4.67290020 W
F2 - Processing parameters
SI 65536
SF 400.3000069 MHz
WDW EM
SSB 0
LB 0.30 Hz
GB 0
PC 1.00
1.404
1.428
1.440
1.450
1.459
1.468
1.491
1.866
1.931
1.945
1.958
2.035
2.067
2.072
2.088
2.096
3.559
3.568
3.596
3.609
3.657
3.672
3.686
3.742
3.769
3.783
3.798
3.837
3.864
3.897
3.967
3.981
3.995
4.008
4.023
4.361
4.368
4.372
4.377
4.384
4.479
4.486
4.490
4.497
4.504
4.718
4.724
4.735
4.742
1.03
3.18
1.06
2.07
1.16
1.12
1.08
1.01
1.00
ppm
1
2
3
4
5
6
7
8
9
4.718
4.724
4.735
4.742
4.75
ppm
1.00

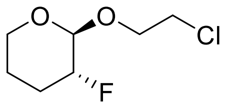

trans-**10b**

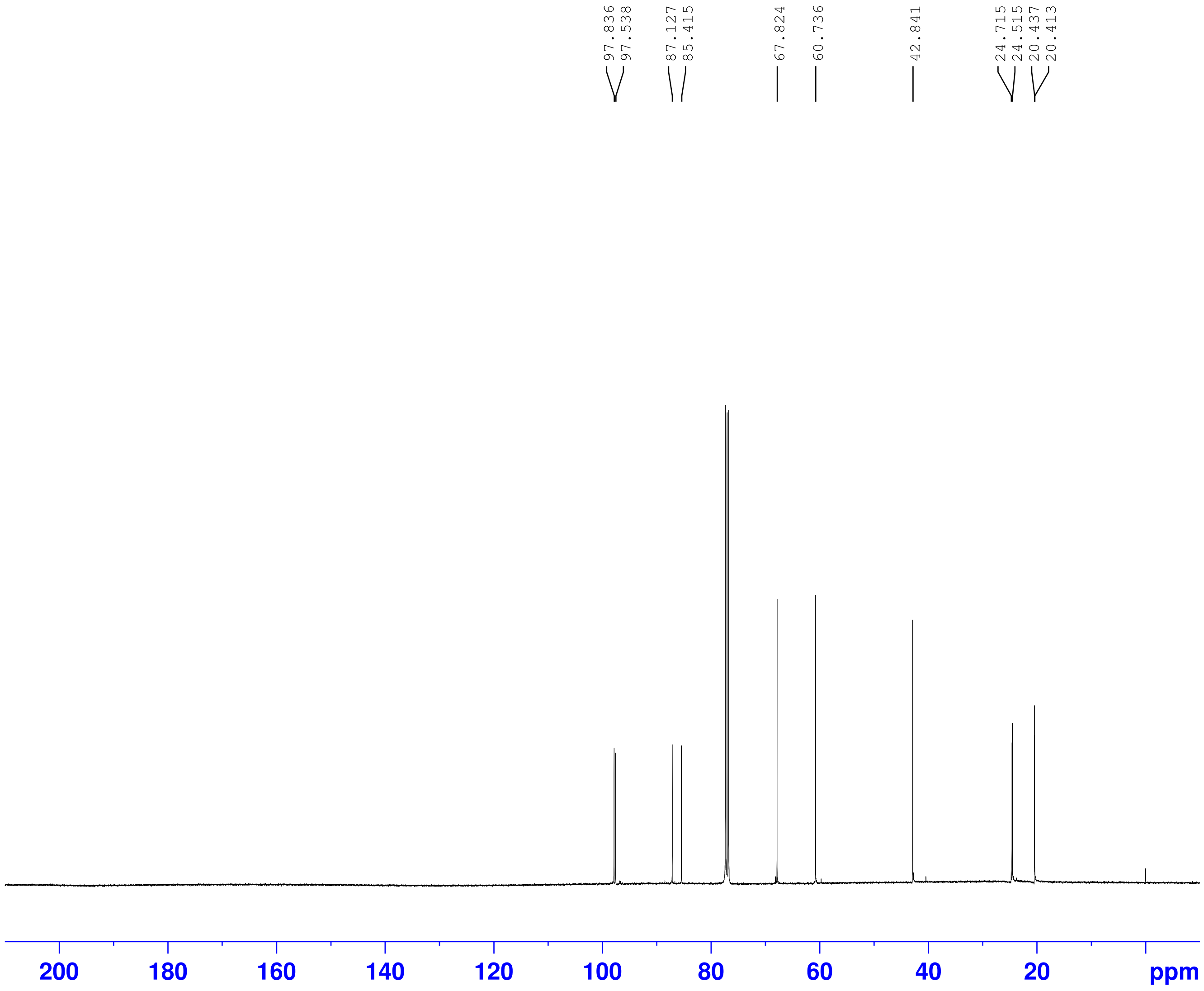

Current Data Parameters
NAME kmd-5-042_2
EXPNO 2
PROCNO 1

F2 - Acquisition Parameters
Date_ 20210131
Time 18.52 h
INSTRUM spect
PROBHD Z150354_0001 (
PULPROG zgpg30
TD 65536
SOLVENT CDCl3
NS 256
DS 4
SWH 24038.461 Hz
FIDRES 0.733596 Hz
AQ 1.3631488 sec
RG 51.78
DW 20.800 usec
DE 25.00 usec
TE 298.0 K
D1 2.00000000 sec
D11 0.03000000 sec
TD0 1
SFO1 100.6655806 MHz
NUC1 13C
P1 10.00 usec
PLW1 19.75099945 W
SFO2 400.3016012 MHz
NUC2 1H
CPDPRG[2 waltz16
PCPD2 80.00 usec
PLW2 4.67290020 W
PLW12 0.10514000 W
PLW13 0.05280100 W

F2 - Processing parameters
SI 32768
SF 100.6555156 MHz
WDW EM
SSB 0
LB 1.00 Hz
GB 0
PC 1.40

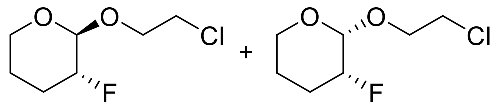

trans-**10b** + *cis*-**10b**

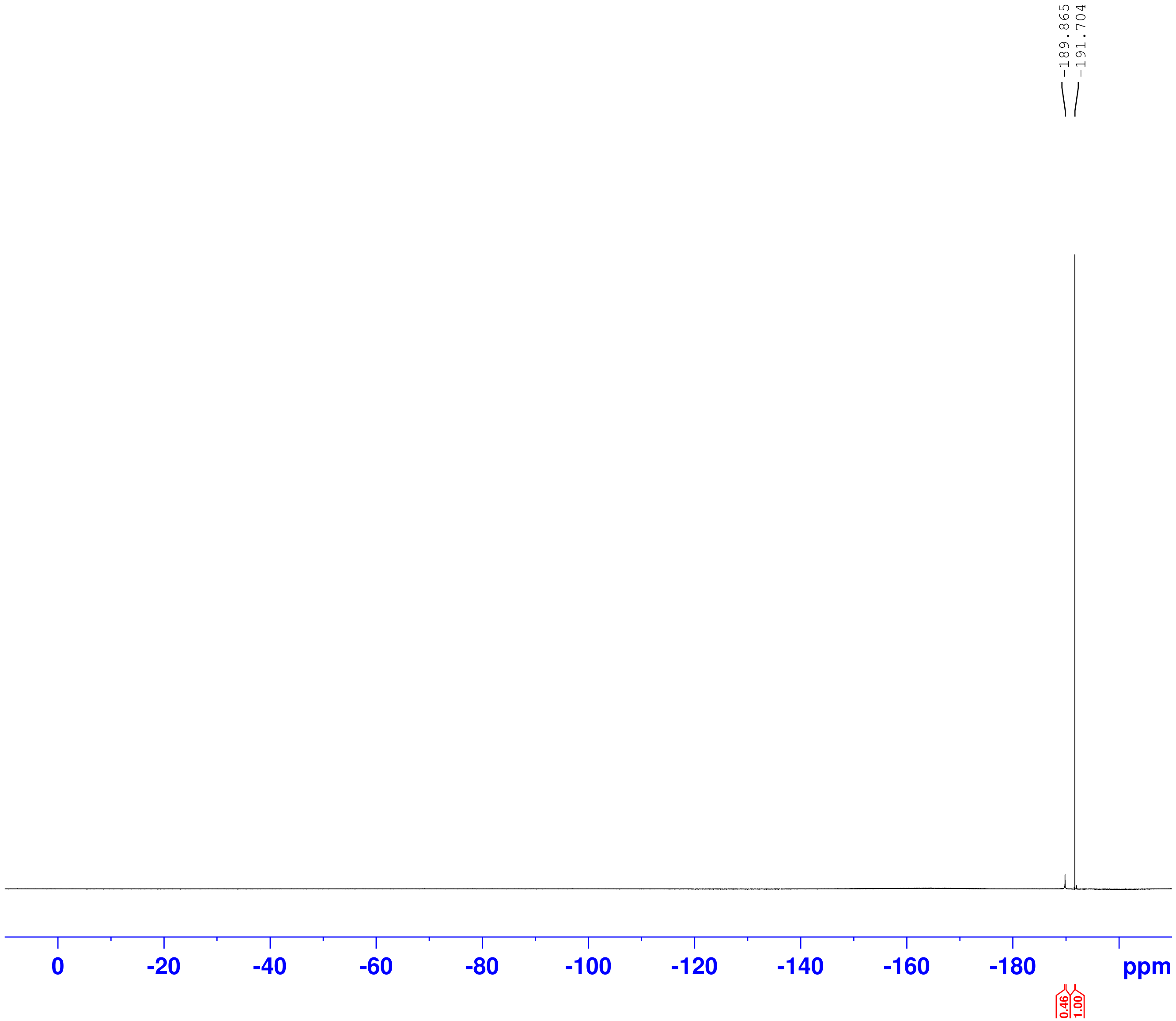


```
Current Data Parameters
NAME      kmd-6-117 pure 19F
EXPNO                    1
PROCNO                   1

F2 - Acquisition Parameters
Date_             20220120
Time                 15.58 h
INSTRUM              spect
PROBHD    Z133023_0002 (
PULPROG        zgfhigqn.2
TD                  131072
SOLVENT              CDCl3
NS                      16
DS                       4
SWH             89285.711 Hz
FIDRES           1.362392 Hz
AQ              0.7340032 sec
RG                 200.67
DW                  5.600 usec
DE                   6.50 usec
TE                  298.3 K
D1             1.00000000 sec
D11            0.03000000 sec
D12            0.00002000 sec
TD0                      1
SFO1          376.5453925 MHz
NUC1                   19F
P1                  16.00 usec
PLW1          16.42300034 W
SFO2          400.2216009 MHz
NUC2                    1H
CPDPRG[2          waltz16
PCPD2               90.00 usec
PLW2          14.49600029 W
PLW12          0.45813999 W

F2 - Processing parameters
SI                   65536
SF            376.5834168 MHz
WDW                     EM
SSB       0
LB                   0.30 Hz
GB        0
PC                   1.00
```

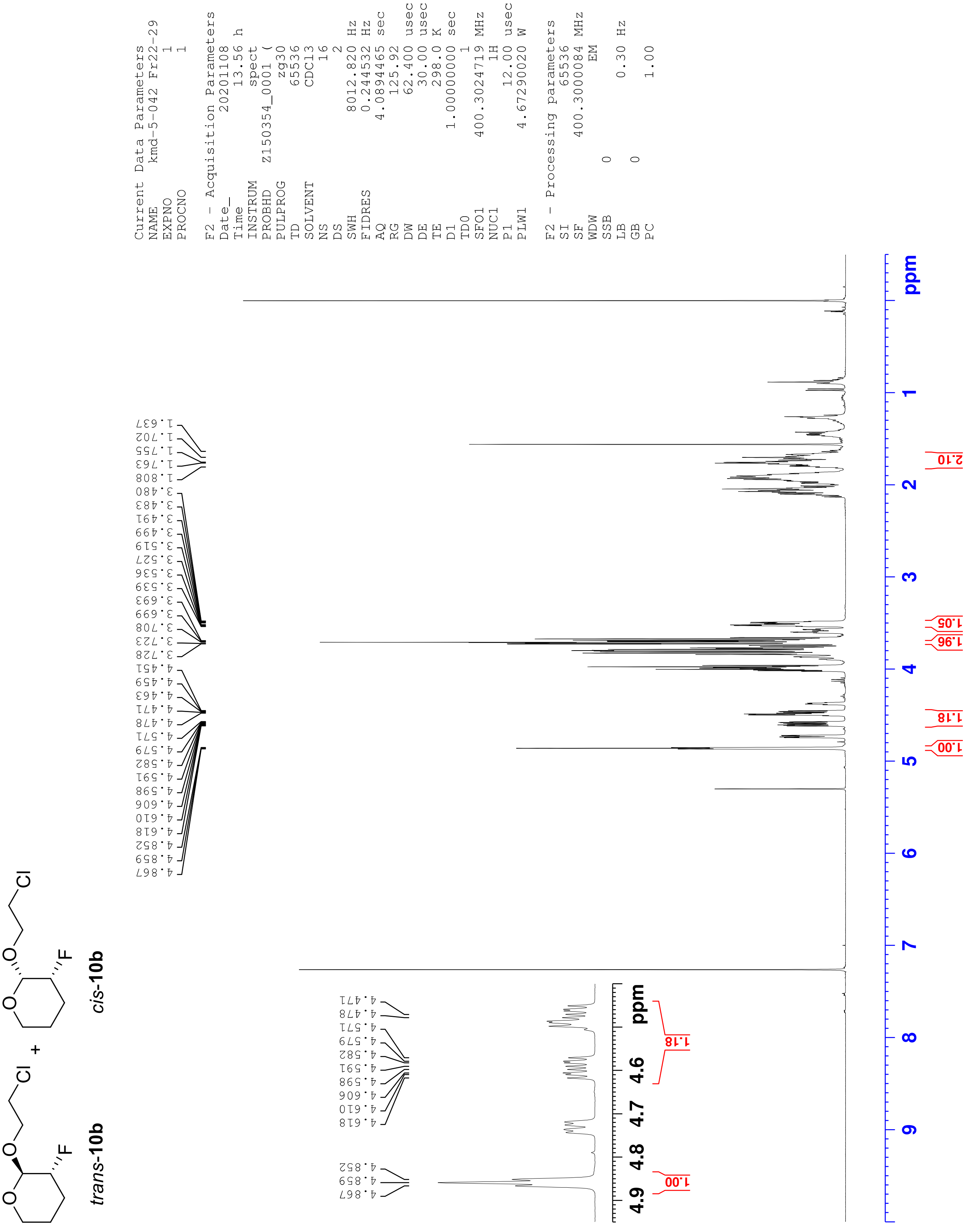
trans-10b
cis-10b
Current Data Parameters
NAME kmd-5-042 Fr22-29
EXPNO 1
PROCNO 1
F2 - Acquisition Parameters
Date_ 20201108
Time 13.56 h
INSTRUM spect
PROBHD Z150354_0001 (
PULPROG zg30
TD 65536
SOLVENT CDCl3
NS 16
DS 2
SWH 8012.820 Hz
FIDRES 0.244532 Hz
AQ 4.0894465 sec
RG 125.92
DW 62.400 usec
DE 30.00 usec
TE 298.0 K
D1 1.00000000 sec
TD0 1
SFO1 400.3024719 MHz
NUC1 1H
P1 12.00 usec
PLW1 4.67290020 W
F2 - Processing parameters
SI 65536
SF 400.3000084 MHz
WDW EM
SSB 0
LB 0.30 Hz
GB 0
PC 1.00
ppm

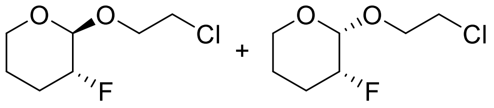

trans-**10b** + *cis*-**10b**

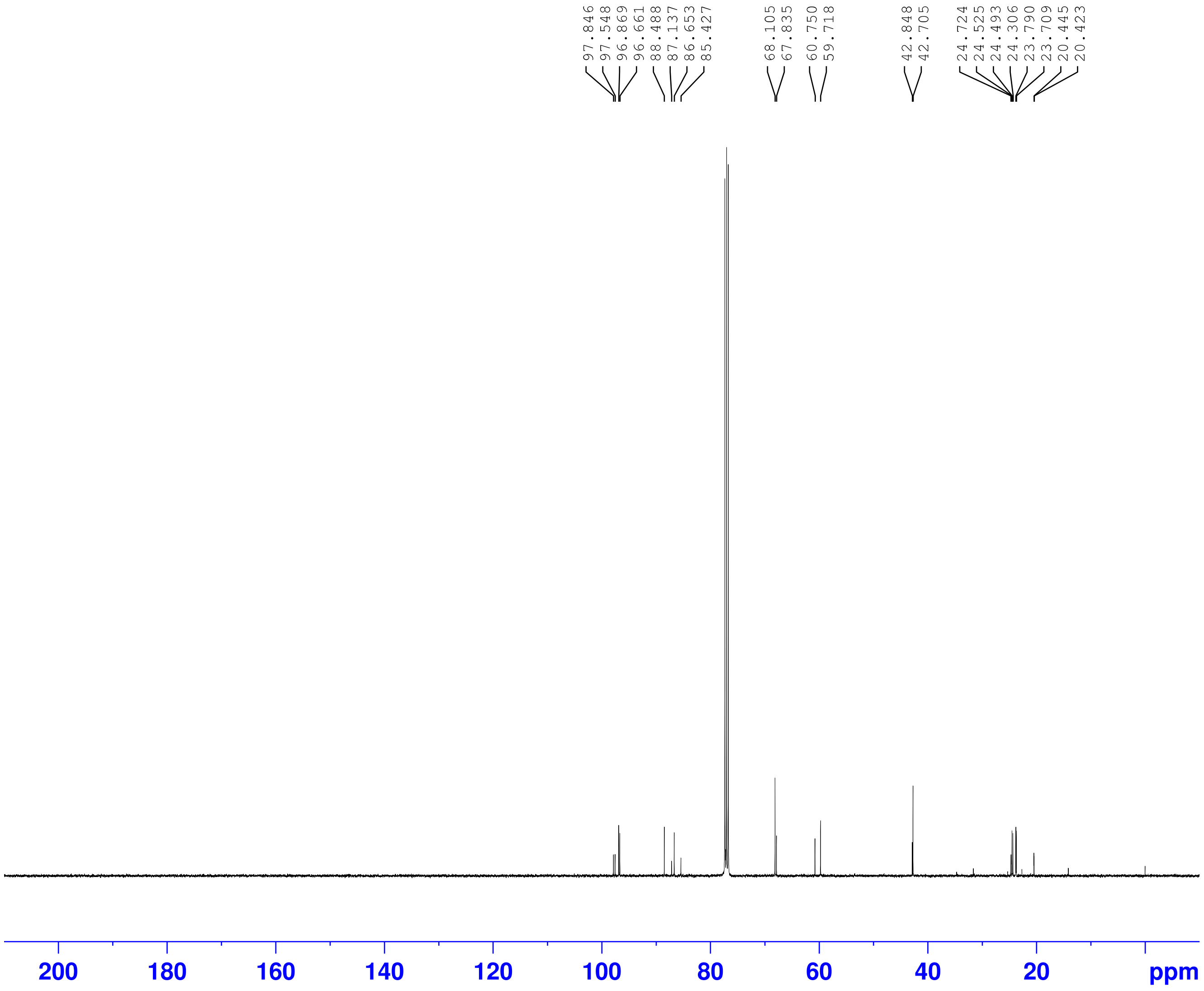


```
Current Data Parameters
NAME      kmd-5-042 Fr22-29
EXPNO                     3
PROCNO                    1

F2 - Acquisition Parameters
Date_              20201108
Time                  14.45 h
INSTRUM               spect
PROBHD    Z150354_0001 (
PULPROG             zgpg30
TD                   65356
SOLVENT              CDCl3
NS                     256
DS                       4
SWH              24038.461 Hz
FIDRES            0.735616 Hz
AQ               1.3594048 sec
RG                   29.21
DW                  20.800 usec
DE                   25.00 usec
TE                   298.0 K
D1              2.00000000 sec
D11             0.03000000 sec
TD0                      1
SFO1          100.6655806 MHz
NUC1                   13C
P1                   10.00 usec
PLW1           19.75099945 W
SFO2          400.3016012 MHz
NUC2                    1H
CPDPRG[2           waltz16
PCPD2                80.00 usec
PLW2            4.67290020 W
PLW12           0.10514000 W
PLW13           0.05280100 W

F2 - Processing parameters
SI                   32768
SF            100.6555151 MHz
WDW                     EM
SSB      0
LB                    1.00 Hz
GB       0
PC                    1.40
```

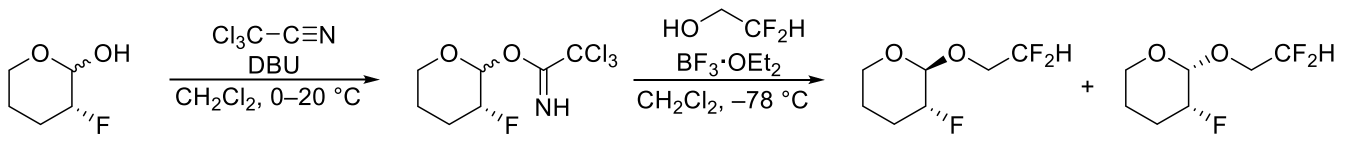

9 **1a** *trans*-**10c** *cis*-**10c**

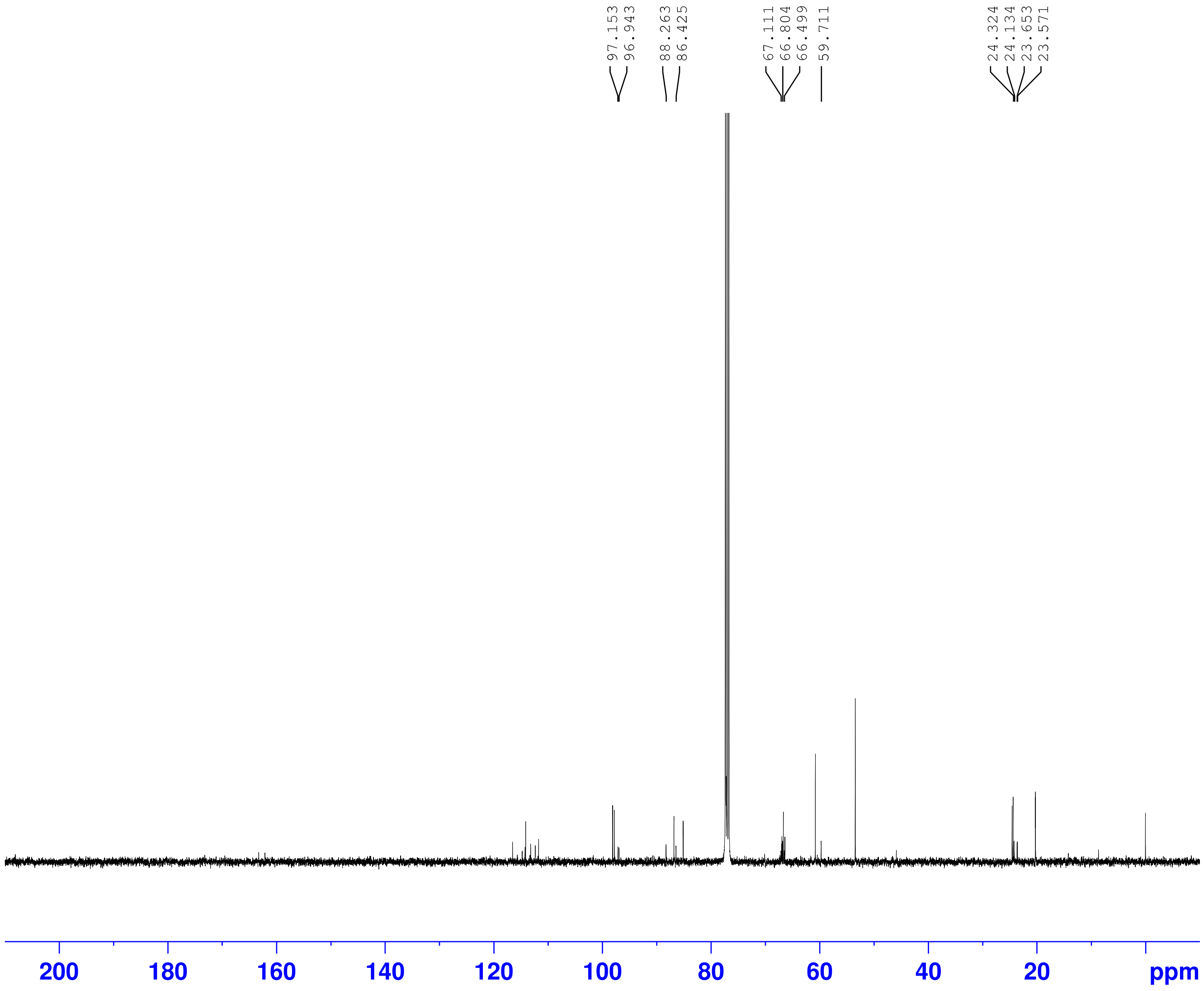


```
Current Data Parameters
NAME              kmd-5-041
EXPNO                     3
PROCNO                    1

F2 - Acquisition Parameters
Date_              20201101
Time                  18.20 h
INSTRUM               spect
PROBHD    Z150354_0001 (
PULPROG             zgpg30
TD                   65356
SOLVENT              CDCl3
NS                     256
DS                       4
SWH              24038.461 Hz
FIDRES            0.735616 Hz
AQ               1.3594048 sec
RG                    31.4
DW                  20.800 usec
DE                   25.00 usec
TE                   298.0 K
D1              2.00000000 sec
D11             0.03000000 sec
TD0                      1
SFO1          100.6655806 MHz
NUC1                   13C
P1                   10.00 usec
PLW1           19.75099945 W
SFO2          400.3016012 MHz
NUC2                    1H
CPDPRG[2           waltz16
PCPD2                80.00 usec
PLW2            4.67290020 W
PLW12           0.10514000 W
PLW13           0.05280100 W

F2 - Processing parameters
SI                   32768
SF            100.6555151 MHz
WDW                     EM
SSB      0
LB                    1.00 Hz
GB       0
PC                    1.40
```

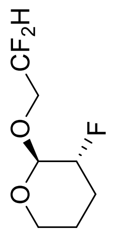

trans-10c

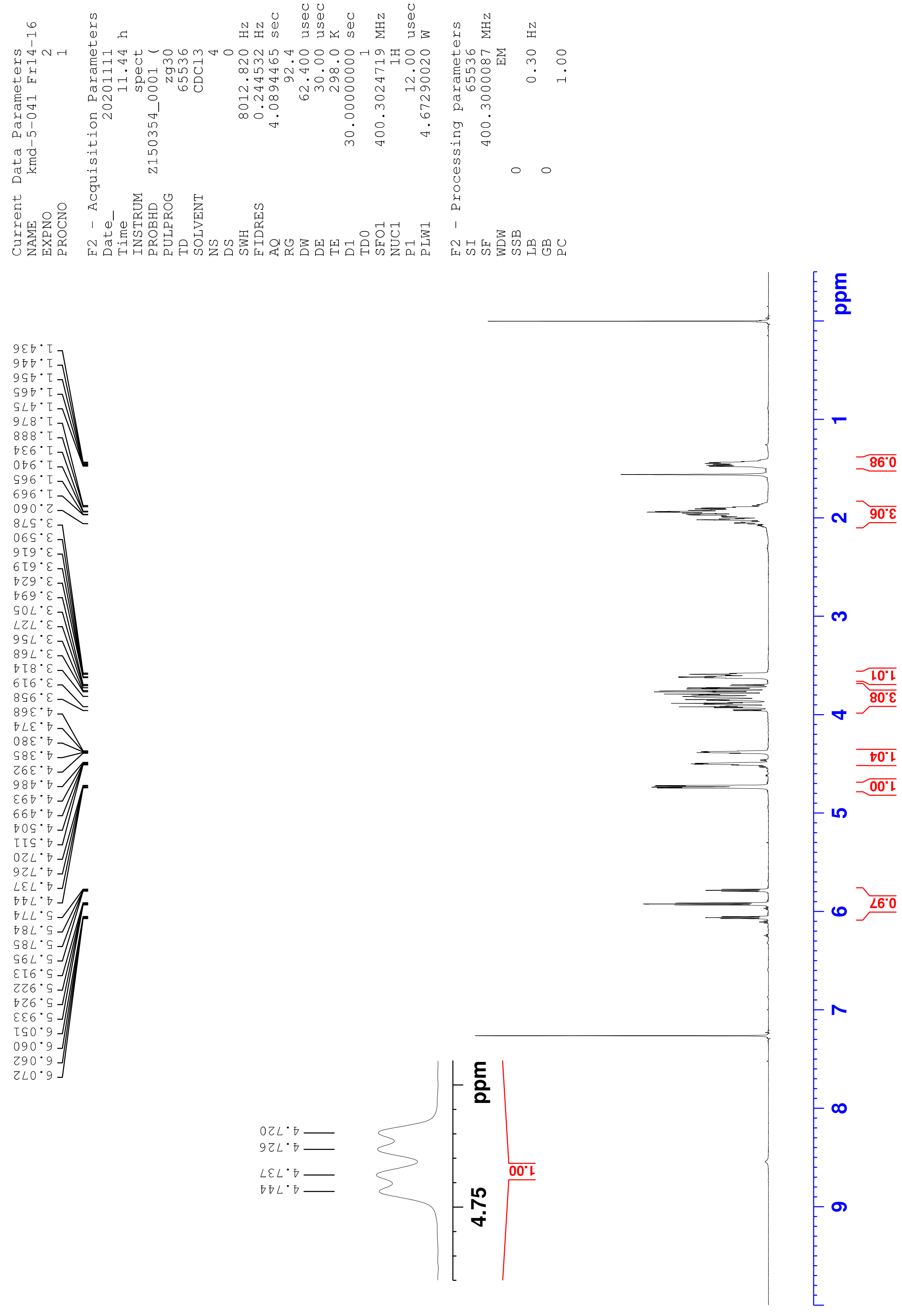
Current Data Parameters
NAME kmd-5-041 Fr14-16
EXPNO 2
PROCNO 1
F2 - Acquisition Parameters
Date_ 20201111
Time 11.44 h
INSTRUM spect
PROBHD Z150354_0001 (
PULPROG zg30
TD 65536
SOLVENT CDCl3
NS 4
DS 0
SWH 8012.820 Hz
FIDRES 0.244532 Hz
AQ 4.0894465 sec
RG 92.4
DW 62.400 usec
DE 30.00 usec
TE 298.0 K
D1 30.00000000 sec
TD0 1
SFO1 400.3024719 MHz
NUC1 1H
P1 12.00 usec
PLW1 4.67290020 W
F2 - Processing parameters
SI 65536
SF 400.3000087 MHz
WDW EM
SSB 0
LB 0.30 Hz
GB 0
PC 1.00
1.436 1.446 1.456 1.465 1.475 1.876 1.888 1.934 1.940 1.965 1.969 2.060 3.578 3.590 3.616 3.619 3.624 3.694 3.705 3.727 3.756 3.768 3.814 3.919 3.958 4.368 4.374 4.380 4.385 4.392 4.486 4.493 4.499 4.504 4.511 4.720 4.726 4.737 4.744 5.774 5.784 5.785 5.795 5.913 5.922 5.924 5.933 6.051 6.060 6.062 6.072
4.720 4.726 4.737 4.744
4.75
ppm
1.00
0.98 3.06 1.01 3.08 1.04 1.00 0.97
1 2 3 4 5 6 7 8 9
ppm

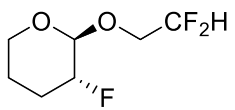

trans-**10c**

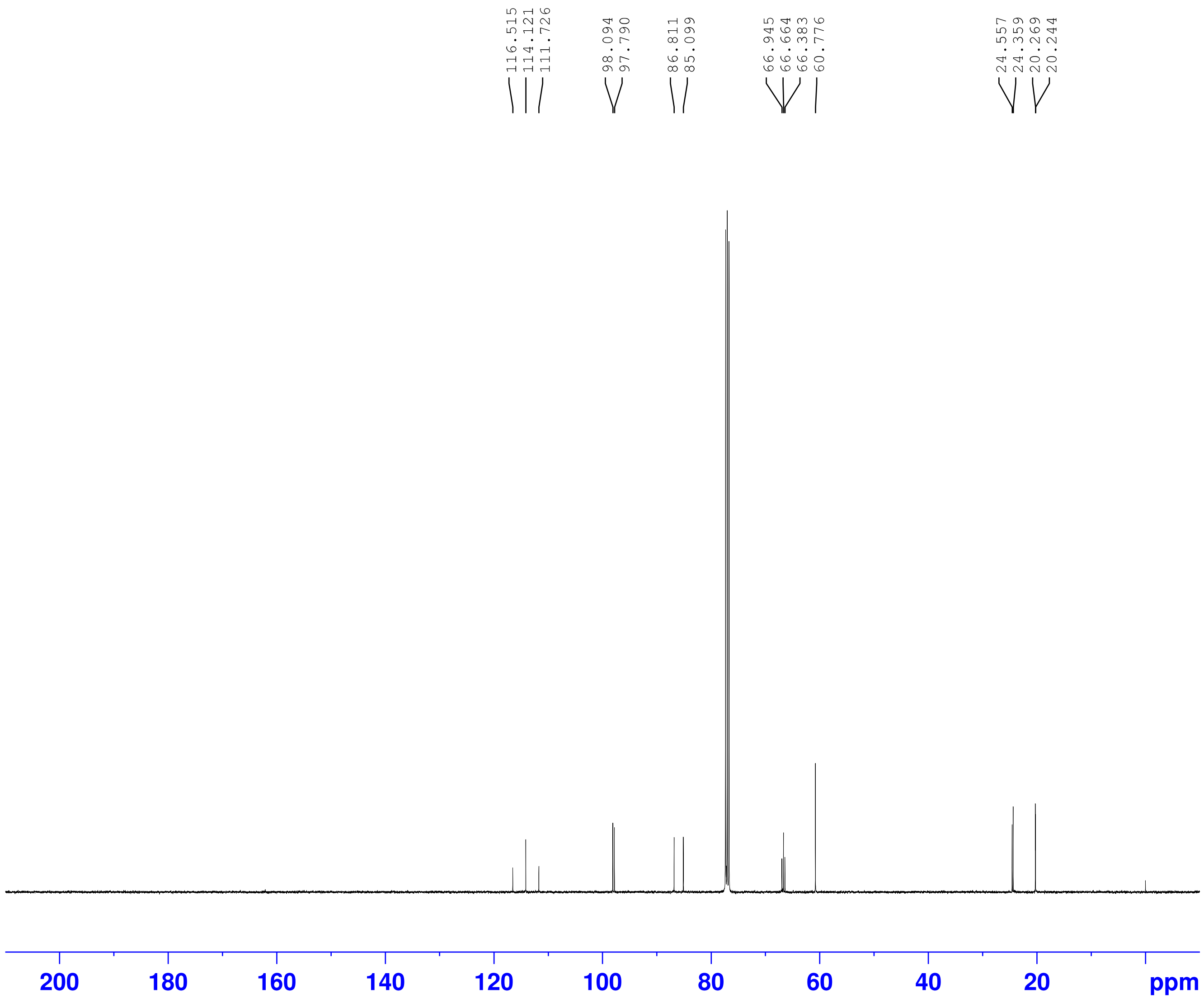


```
Current Data Parameters
NAME       kmd-5-041 Fr14-16
EXPNO                      3
PROCNO                     1

F2 - Acquisition Parameters
Date_               20201111
Time                 12.00 h
INSTRUM                spect
PROBHD     Z150354_0001 (
PULPROG              zgpg30
TD                    65356
SOLVENT               CDCl3
NS                      256
DS                        4
SWH             24038.461 Hz
FIDRES           0.735616 Hz
AQ              1.3594048 sec
RG                     31.4
DW                   20.800 usec
DE                    25.00 usec
TE                    298.0 K
D1               2.00000000 sec
D11              0.03000000 sec
TD0                       1
SFO1           100.6655806 MHz
NUC1                    13C
P1                    10.00 usec
PLW1            19.75099945 W
SFO2           400.3016012 MHz
NUC2                     1H
CPDPRG[2           waltz16
PCPD2                 80.00 usec
PLW2             4.67290020 W
PLW12            0.10514000 W
PLW13            0.05280100 W

F2 - Processing parameters
SI                    32768
SF             100.6555151 MHz
WDW                      EM
SSB       0
LB                     1.00 Hz
GB        0
PC                     1.40
```

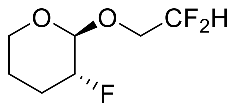

trans-**10c**

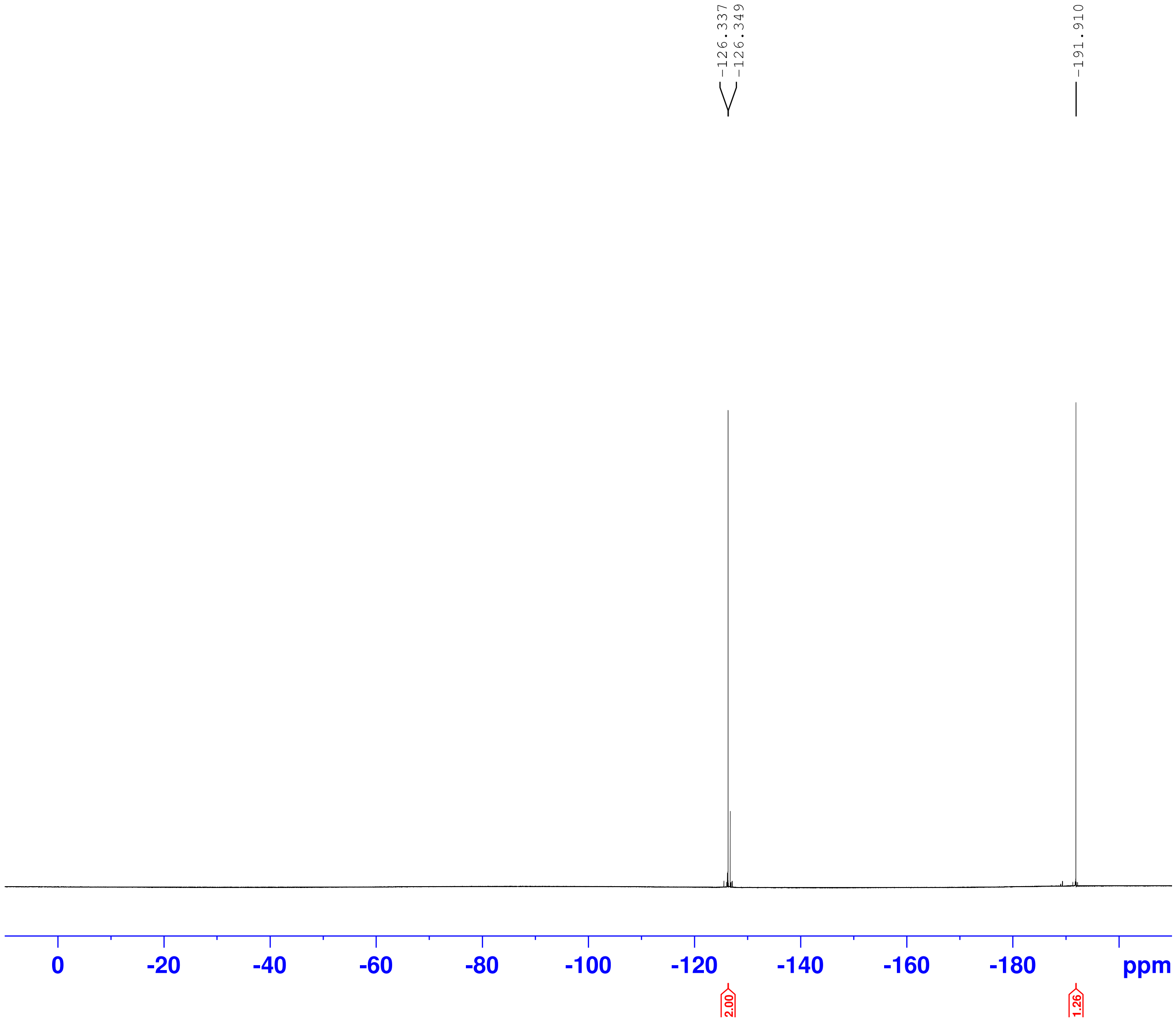

Current Data Parameters

NAME	kmd-5-041 Fr14-16 19F
EXPNO	1
PROCNO	1

F2 - Acquisition Parameters

Date_	20210105
Time	14.30 h
INSTRUM	spect
PROBHD	Z108618_0422 (
PULPROG	zgfhigqn.2
TD	131072
SOLVENT	CDCl3
NS	16
DS	4
SWH	89285.711 Hz
FIDRES	1.362392 Hz
AQ	0.7340032 sec
RG	200.67
DW	5.600 usec
DE	6.50 usec
TE	301.9 K
D1	1.00000000 sec
D11	0.03000000 sec
D12	0.00002000 sec
TD0	1
SFO1	376.5453925 MHz
NUC1	19F
P1	15.00 usec
PLW1	16.89999962 W
SFO2	400.2216009 MHz
NUC2	1H
CPDPRG[2	waltz16
PCPD2	90.00 usec
PLW2	14.69999981 W
PLW12	0.40832999 W

F2 - Processing parameters

SI	65536
SF	376.5834167 MHz
WDW	EM
SSB	0
LB	0.30 Hz
GB	0
PC	1.00

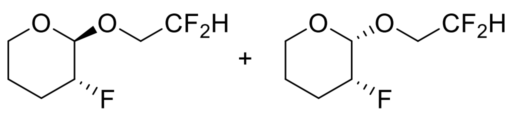

trans-**10c** + *cis*-**10c**

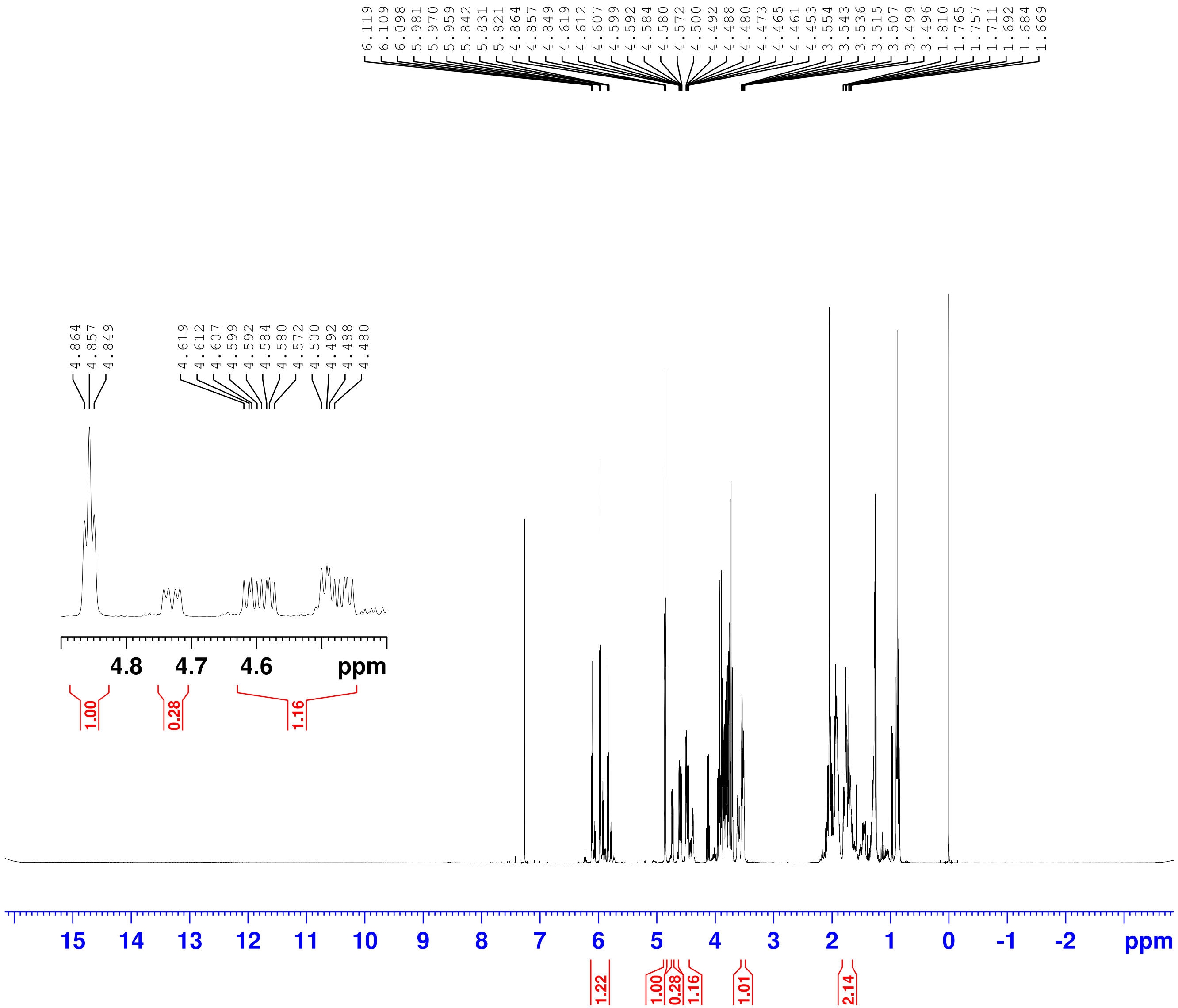

Current Data Parameters
NAME kmd-6-124 Fr28-38
EXPNO 2
PROCNO 1

F2 - Acquisition Parameters
Date_ 20220124
Time 20.52 h
INSTRUM spect
PROBHD Z150354_0001 (
PULPROG zg30
TD 65536
SOLVENT CDCl3
NS 4
DS 0
SWH 8012.820 Hz
FIDRES 0.244532 Hz
AQ 4.0894465 sec
RG 63.04
DW 62.400 usec
DE 30.00 usec
TE 298.0 K
D1 30.00000000 sec
TD0 1
SFO1 400.3024719 MHz
NUC1 1H
P1 12.00 usec
PLW1 4.64209986 W

F2 - Processing parameters
SI 65536
SF 400.3000077 MHz
WDW EM
SSB 0
LB 0.30 Hz
GB 0
PC 1.00

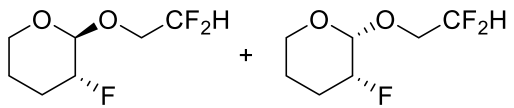

trans-**10c** + *cis*-**10c**

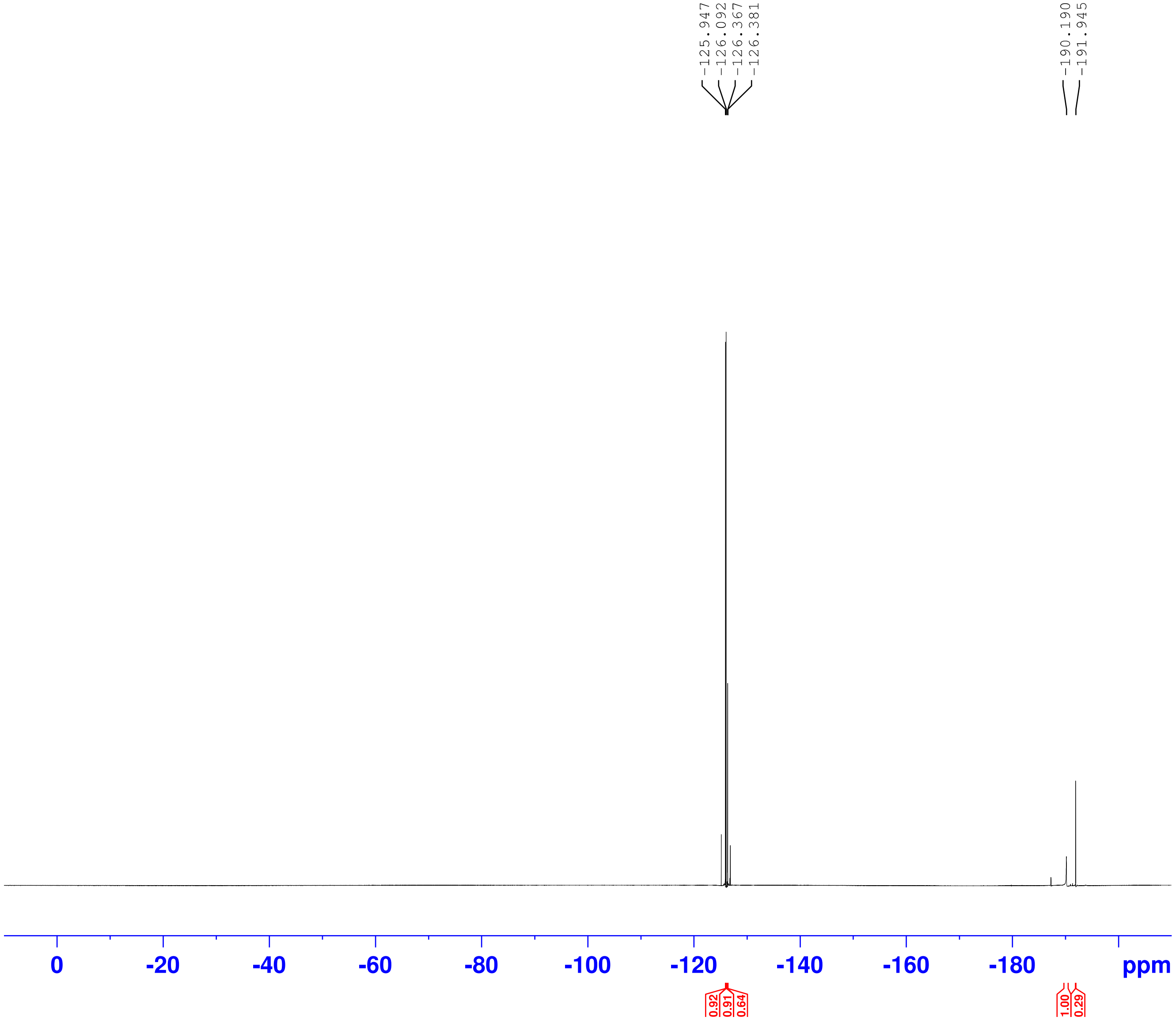


```
Current Data Parameters
NAME        kmd-6-124 Fr28-28 19F
EXPNO                          1
PROCNO                         1

F2 - Acquisition Parameters
Date_                   20220126
Time                       12.08 h
INSTRUM                    spect
PROBHD        Z133023_0002 (
PULPROG             zgfhigqn.2
TD                        131072
SOLVENT                    CDCl3
NS                            16
DS                             4
SWH                    89285.711 Hz
FIDRES                  1.362392 Hz
AQ                     0.7340032 sec
RG                        200.67
DW                         5.600 usec
DE                          6.50 usec
TE                         298.3 K
D1                    1.00000000 sec
D11                   0.03000000 sec
D12                   0.00002000 sec
TD0                            1
SFO1                 376.5453925 MHz
NUC1                         19F
P1                         16.00 usec
PLW1                 16.42300034 W
SFO2                 400.2216009 MHz
NUC2                          1H
CPDPRG[2                 waltz16
PCPD2                      90.00 usec
PLW2                 14.49600029 W
PLW12                 0.45813999 W

F2 - Processing parameters
SI                         65536
SF                   376.5834169 MHz
WDW                           EM
SSB          0
LB                          0.30 Hz
GB           0
PC                          1.00
```

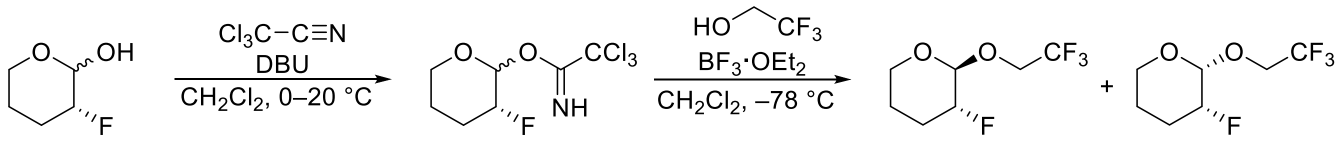

9 **1a** *trans*-**10d** *cis*-**10d**

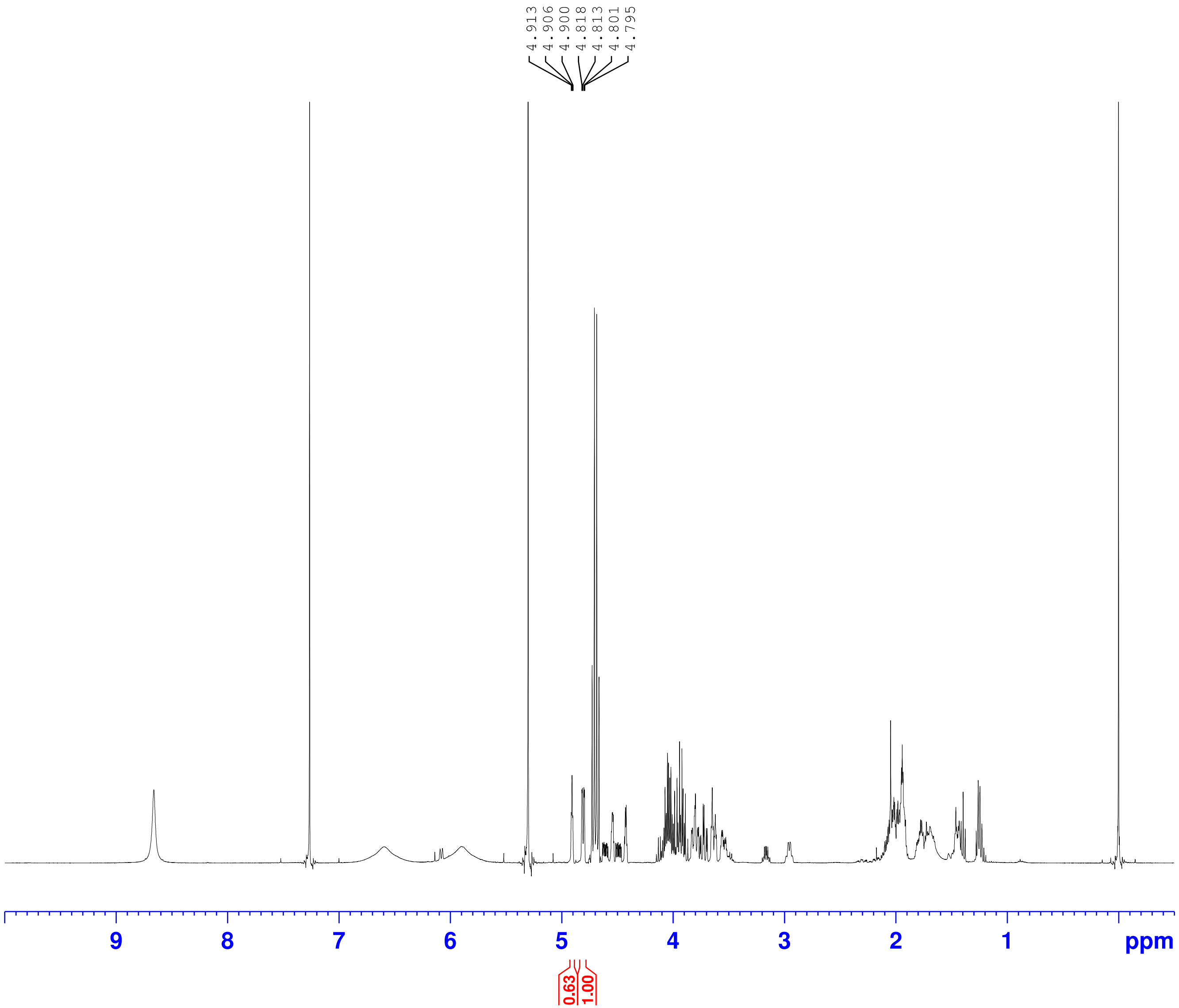


```
Current Data Parameters
NAME              kmd-5-040
EXPNO                     2
PROCNO                    1

F2 - Acquisition Parameters
Date_              20201101
Time                  17.14 h
INSTRUM               spect
PROBHD    Z150354_0001 (
PULPROG                zg30
TD                    65536
SOLVENT               CDCl3
NS                        4
DS                        0
SWH                8012.820 Hz
FIDRES             0.244532 Hz
AQ                4.0894465 sec
RG                   141.61
DW                   62.400 usec
DE                    30.00 usec
TE                    298.0 K
D1              30.00000000 sec
TD0                       1
SFO1            400.3024719 MHz
NUC1                     1H
P1                    12.00 usec
PLW1             4.67290020 W

F2 - Processing parameters
SI                    65536
SF              400.3000083 MHz
WDW                      EM
SSB       0
LB                     0.30 Hz
GB        0
PC                     1.00
```

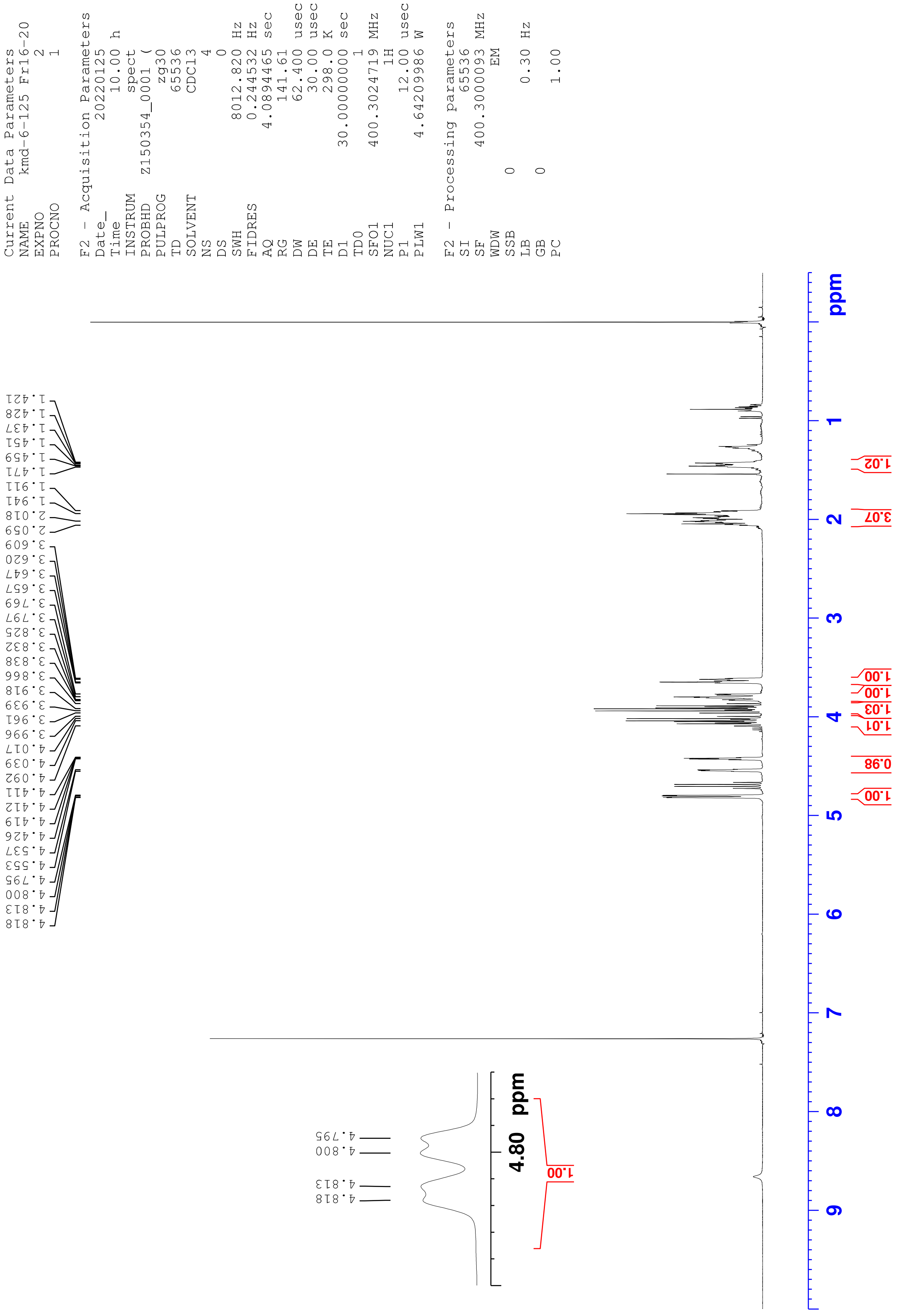
Current Data Parameters
NAME kmd-6-125 Fr16-20
EXPNO 2
PROCNO 1
F2 - Acquisition Parameters
Date_ 20220125
Time 10.00 h
INSTRUM spect
PROBHD Z150354_0001 (
PULPROG zg30
TD 65536
SOLVENT CDCl3
NS 4
DS 0
SWH 8012.820 Hz
FIDRES 0.244532 Hz
AQ 4.0894465 sec
RG 141.61
DW 62.400 usec
DE 30.00 usec
TE 298.0 K
D1 30.00000000 sec
TD0 1
SFO1 400.3024719 MHz
NUC1 1H
P1 12.00 usec
PLW1 4.64209986 W
F2 - Processing parameters
SI 65536
SF 400.3000093 MHz
WDW EM
SSB 0
LB 0.30 Hz
GB 0
PC 1.00
1.421
1.428
1.437
1.451
1.459
1.471
1.911
1.941
2.018
2.059
3.609
3.620
3.647
3.657
3.769
3.797
3.825
3.832
3.838
3.866
3.918
3.939
3.961
3.996
4.017
4.039
4.092
4.411
4.412
4.419
4.426
4.537
4.553
4.795
4.800
4.813
4.818
1.02
3.07
1.00
1.00
1.03
1.01
0.98
1.00
ppm
4.80 ppm
1.00

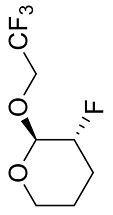
O
O
CF3
F

trans-**10d**

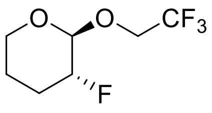

trans-**10d**

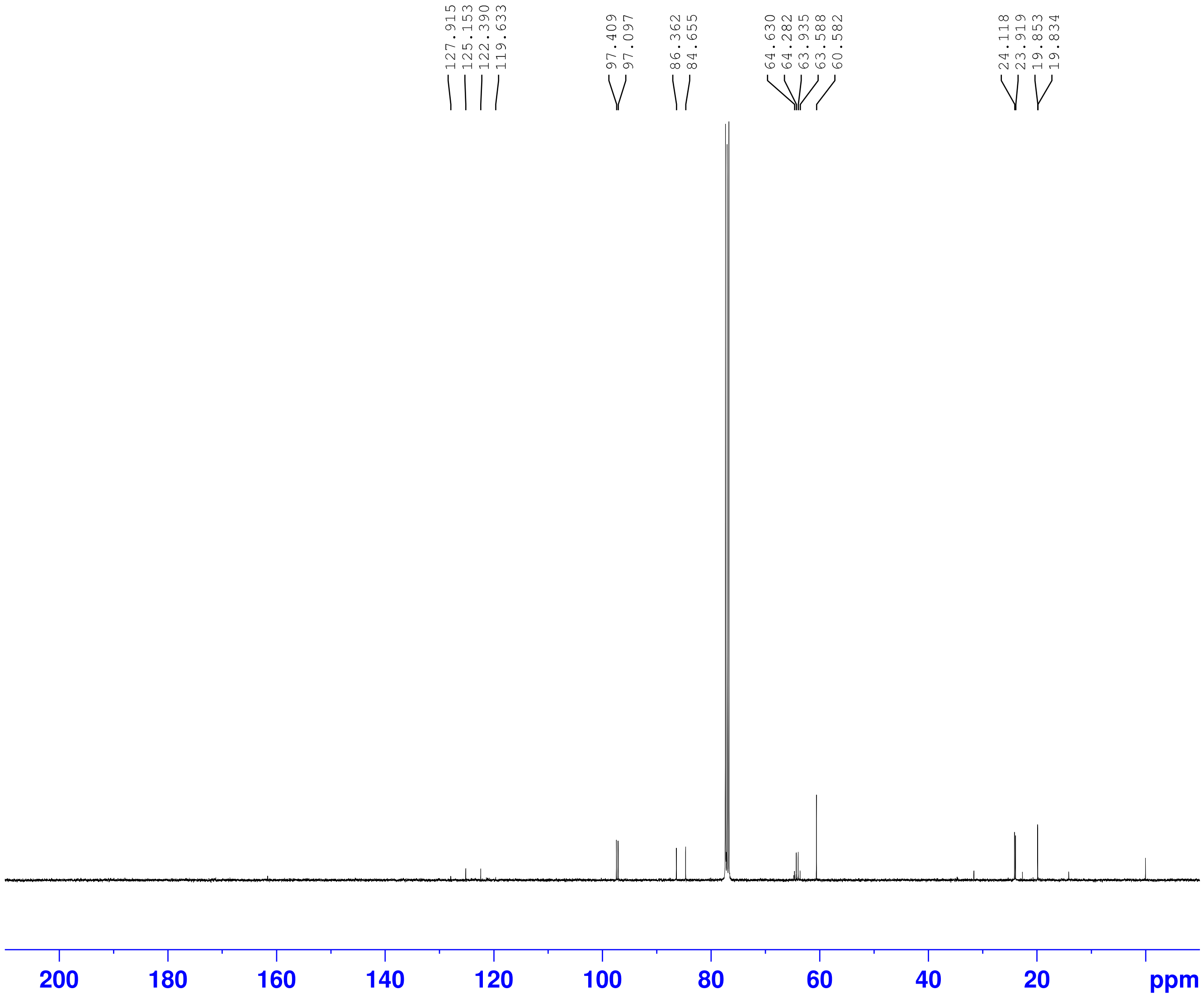


```
Current Data Parameters
NAME      kmd-6-125 Fr16-20
EXPNO                     3
PROCNO                    1

F2 - Acquisition Parameters
Date_              20220125
Time                  10.16 h
INSTRUM               spect
PROBHD     Z150354_0001 (
PULPROG             zgpg30
TD                    65356
SOLVENT               CDCl3
NS                      256
DS                        4
SWH               24038.461 Hz
FIDRES             0.735616 Hz
AQ                1.3594048 sec
RG                    25.77
DW                   20.800 usec
DE                    25.00 usec
TE                    298.0 K
D1               2.00000000 sec
D11              0.03000000 sec
TD0                       1
SFO1            100.6655806 MHz
NUC1                    13C
P1                    10.00 usec
PLW1            17.99699974 W
SFO2            400.3016012 MHz
NUC2                     1H
CPDPRG[2            waltz16
PCPD2                 80.00 usec
PLW2             4.64209986 W
PLW12            0.10445000 W
PLW13            0.05245300 W

F2 - Processing parameters
SI                    32768
SF              100.6555152 MHz
WDW                      EM
SSB       0
LB                     1.00 Hz
GB        0
PC                     1.40
```

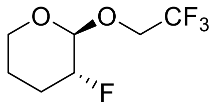

trans-**10d**

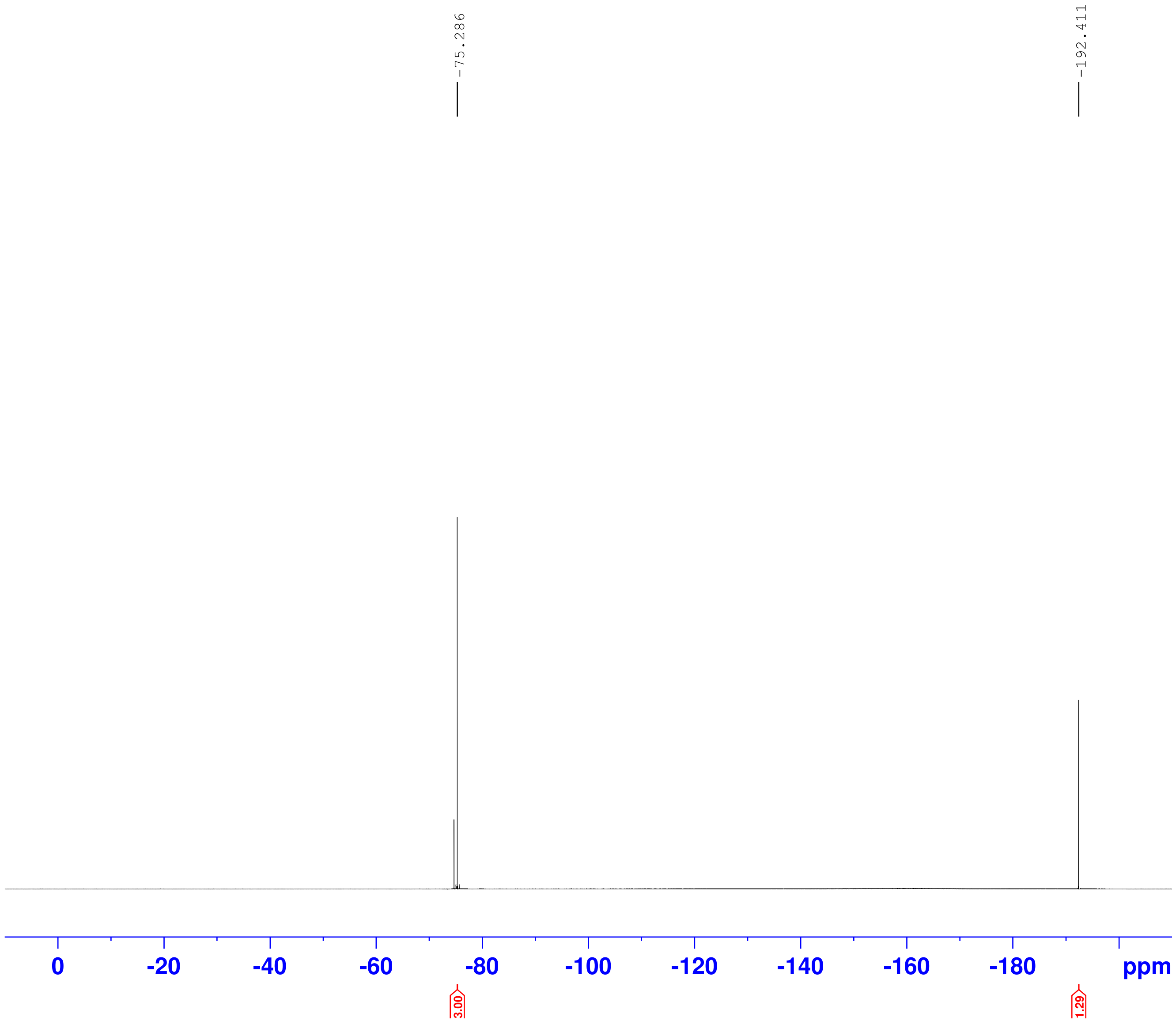


```
Current Data Parameters
NAME      kmd-6-125 Fr16-20 19F
EXPNO                  1
PROCNO                 1

F2 - Acquisition Parameters
Date_          20220125
Time              15.22 h
INSTRUM           spect
PROBHD    Z133023_0002 (
PULPROG      zgfhigqn.2
TD               131072
SOLVENT           CDCl3
NS                   16
DS                    4
SWH          89285.711 Hz
FIDRES        1.362392 Hz
AQ           0.7340032 sec
RG              200.67
DW               5.600 usec
DE                6.50 usec
TE               298.3 K
D1          1.00000000 sec
D11         0.03000000 sec
D12         0.00002000 sec
TD0                   1
SFO1       376.5453925 MHz
NUC1                19F
P1               16.00 usec
PLW1       16.42300034 W
SFO2       400.2216009 MHz
NUC2                 1H
CPDPRG[2        waltz16
PCPD2            90.00 usec
PLW2       14.49600029 W
PLW12       0.45813999 W

F2 - Processing parameters
SI                65536
SF          376.5834175 MHz
WDW                  EM
SSB       0
LB                 0.30 Hz
GB        0
PC                 1.00
```

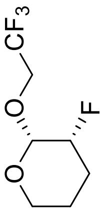

cis-**10d**

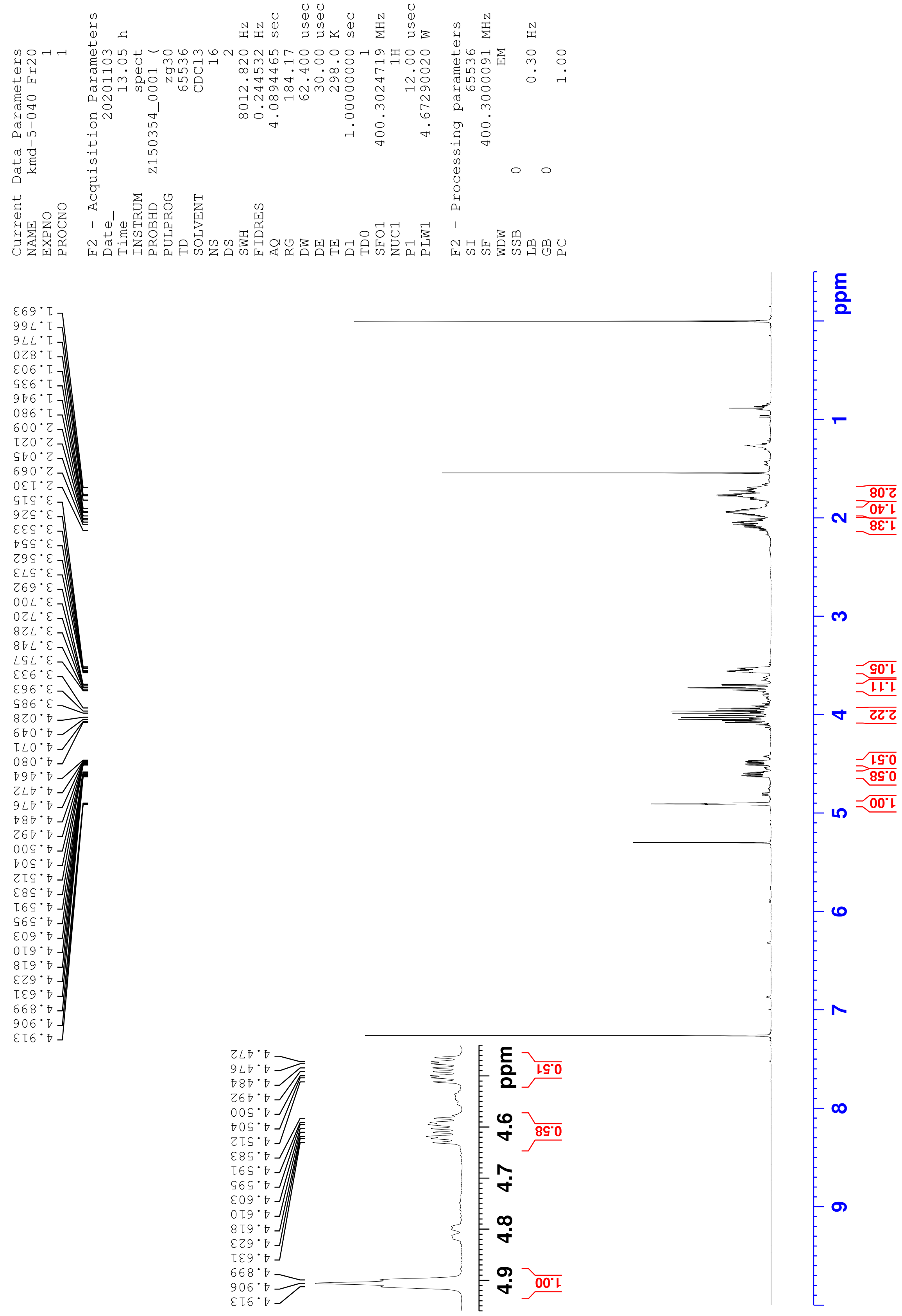
Current Data Parameters
NAME kmd-5-040 Fr20
EXPNO 1
PROCNO 1
F2 - Acquisition Parameters
Date_ 20201103
Time 13.05 h
INSTRUM spect
PROBHD Z150354_0001 (
PULPROG zg30
TD 65536
SOLVENT CDCl3
NS 16
DS 2
SWH 8012.820 Hz
FIDRES 0.244532 Hz
AQ 4.0894465 sec
RG 184.17
DW 62.400 usec
DE 30.00 usec
TE 298.0 K
D1 1.00000000 sec
TD0 1
SFO1 400.3024719 MHz
NUC1 1H
P1 12.00 usec
PLW1 4.67290020 W
F2 - Processing parameters
SI 65536
SF 400.3000091 MHz
WDW EM
SSB 0
LB 0.30 Hz
GB 0
PC 1.00
1.693 1.766 1.776 1.820 1.903 1.935 1.946 1.980 2.009 2.021 2.045 2.069 2.130 3.515 3.526 3.533 3.554 3.562 3.573 3.692 3.700 3.720 3.728 3.748 3.757 3.933 3.963 3.985 4.028 4.049 4.071 4.080 4.464 4.472 4.476 4.484 4.492 4.500 4.504 4.512 4.583 4.591 4.595 4.603 4.610 4.618 4.623 4.631 4.899 4.906 4.913
4.472 4.476 4.484 4.492 4.500 4.504 4.512 4.583 4.591 4.595 4.603 4.610 4.618 4.623 4.631 4.899 4.906 4.913
4.9 4.8 4.7 4.6 ppm
1.00 0.58 0.51
9 8 7 6 5 4 3 2 1 ppm
1.00 0.58 0.51 2.22 1.11 1.05 1.38 1.40 2.08

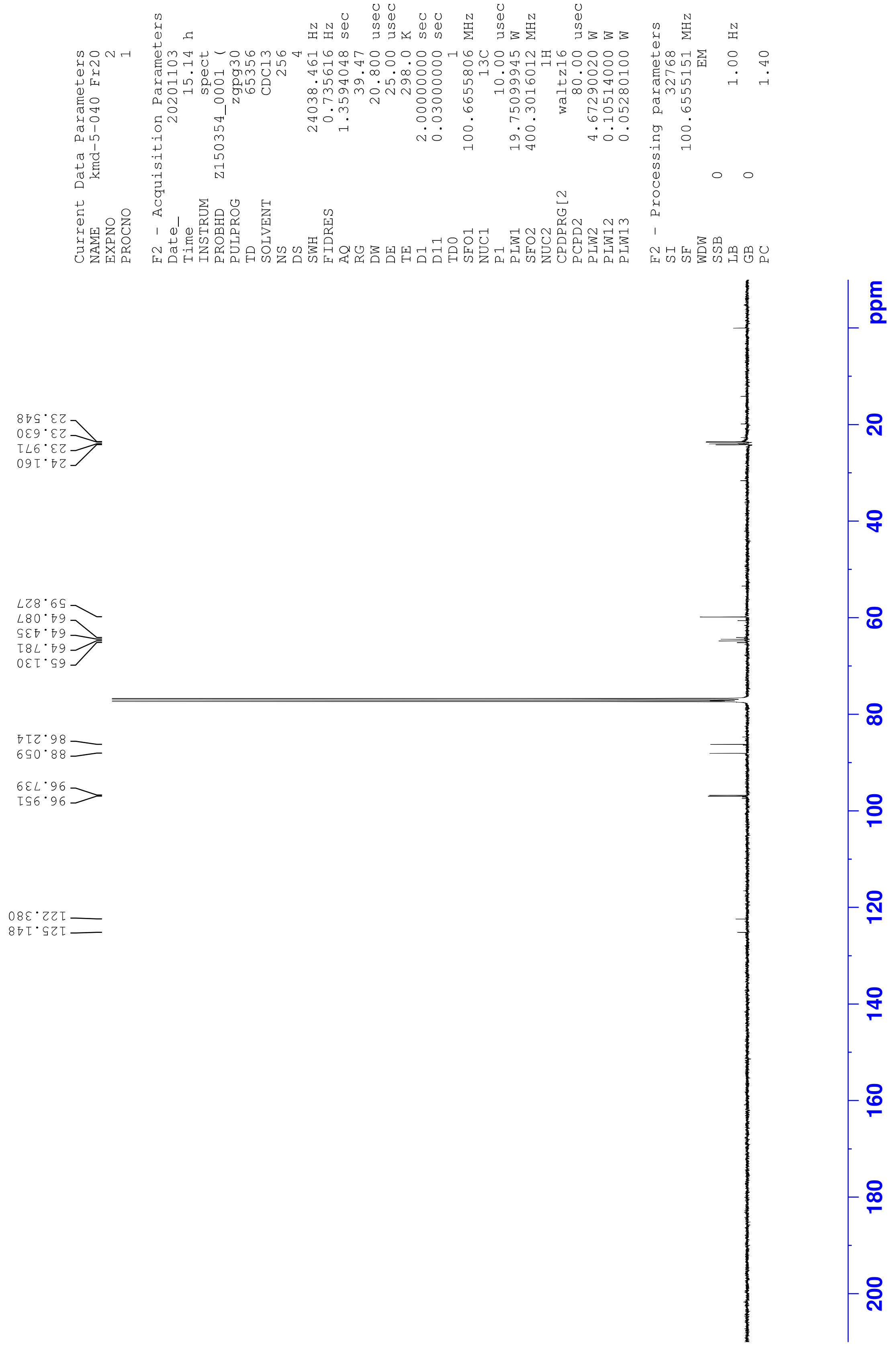
Current Data Parameters
NAME kmd-5-040 Fr20
EXPNO 2
PROCNO 1
F2 - Acquisition Parameters
Date_ 20201103
Time 15.14 h
INSTRUM spect
PROBHD Z150354_0001 (
PULPROG zgpg30
TD 65356
SOLVENT CDCl3
NS 256
DS 4
SWH 24038.461 Hz
FIDRES 0.735616 Hz
AQ 1.3594048 sec
RG 39.47
DW 20.800 usec
DE 25.00 usec
TE 298.0 K
D1 2.00000000 sec
D11 0.03000000 sec
TD0 1
SFO1 100.6655806 MHz
NUC1 13C
P1 10.00 usec
PLW1 19.75099945 W
SFO2 400.3016012 MHz
NUC2 1H
CPDPRG[2 waltz16
PCPD2 80.00 usec
PLW2 4.67290020 W
PLW12 0.10514000 W
PLW13 0.05280100 W
F2 - Processing parameters
SI 32768
SF 100.6555151 MHz
WDW EM
SSB 0
LB 1.00 Hz
GB 0
PC 1.40
23.548
23.630
23.971
24.160
59.827
64.087
64.435
64.781
65.130
86.214
88.059
96.739
96.951
122.380
125.148
ppm
20
40
60
80
100
120
140
160
180
200

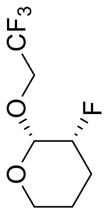
O
O
CF3
F

cis-**10d**

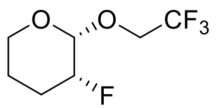

cis-**10d**

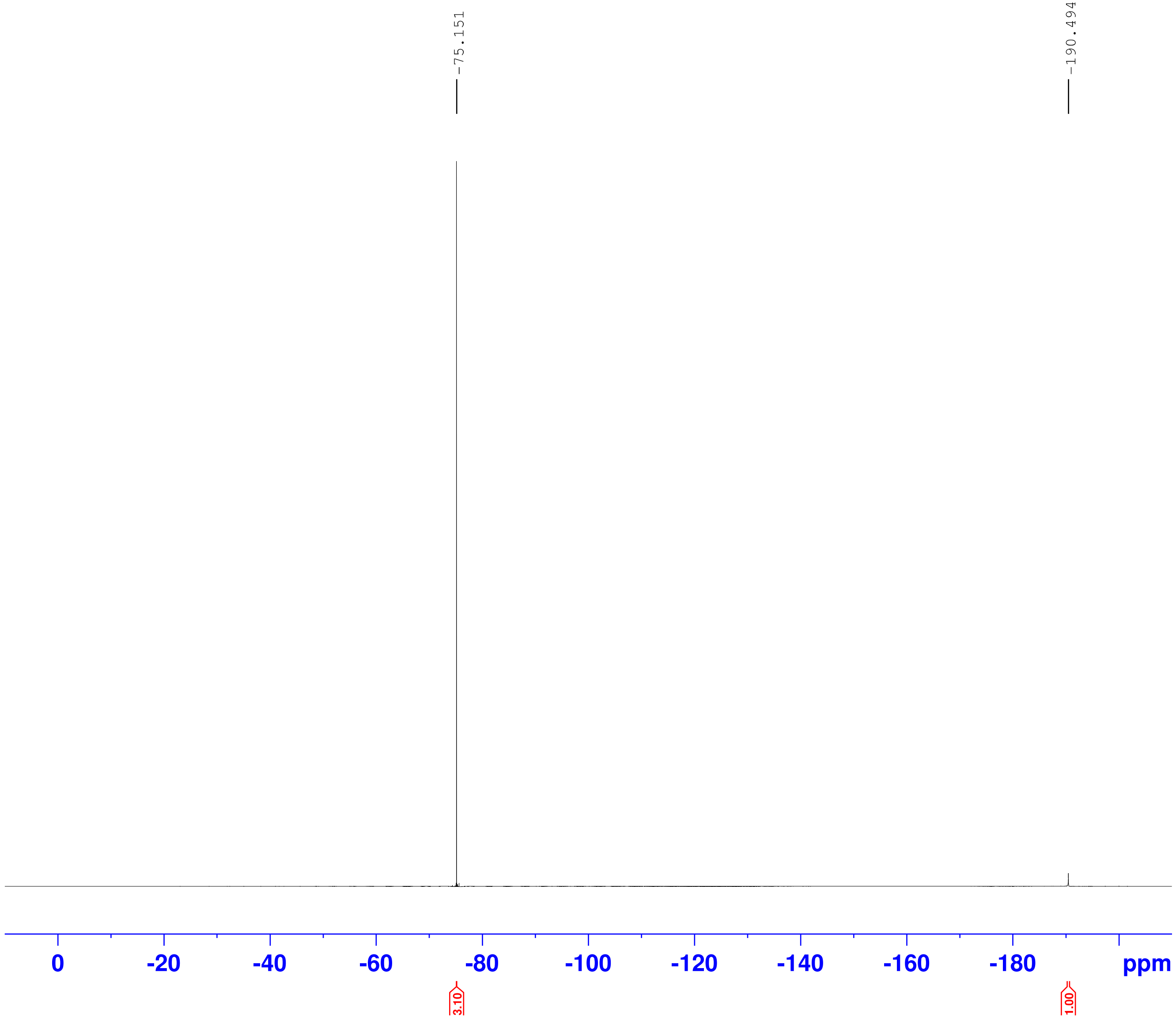


```
Current Data Parameters
NAME      kmd-6-125 Fr24-38 19F
EXPNO                     1
PROCNO                    1

F2 - Acquisition Parameters
Date_              20220125
Time                  15.27 h
INSTRUM               spect
PROBHD    Z133023_0002 (
PULPROG         zgfhigqn.2
TD                   131072
SOLVENT               CDCl3
NS                       16
DS                        4
SWH              89285.711 Hz
FIDRES            1.362392 Hz
AQ               0.7340032 sec
RG                   200.67
DW                    5.600 usec
DE                     6.50 usec
TE                    298.3 K
D1               1.00000000 sec
D11              0.03000000 sec
D12              0.00002000 sec
TD0                       1
SFO1            376.5453925 MHz
NUC1                    19F
P1                    16.00 usec
PLW1            16.42300034 W
SFO2            400.2216009 MHz
NUC2                     1H
CPDPRG[2            waltz16
PCPD2                 90.00 usec
PLW2            14.49600029 W
PLW12            0.45813999 W

F2 - Processing parameters
SI                    65536
SF              376.5834171 MHz
WDW                      EM
SSB       0
LB                     0.30 Hz
GB        0
PC                     1.00
```

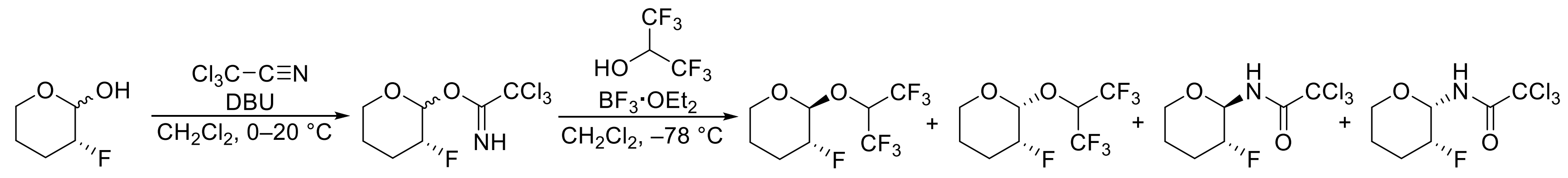

9 1a *trans*-10e *cis*-10e *trans*-S3 *cis*-S3

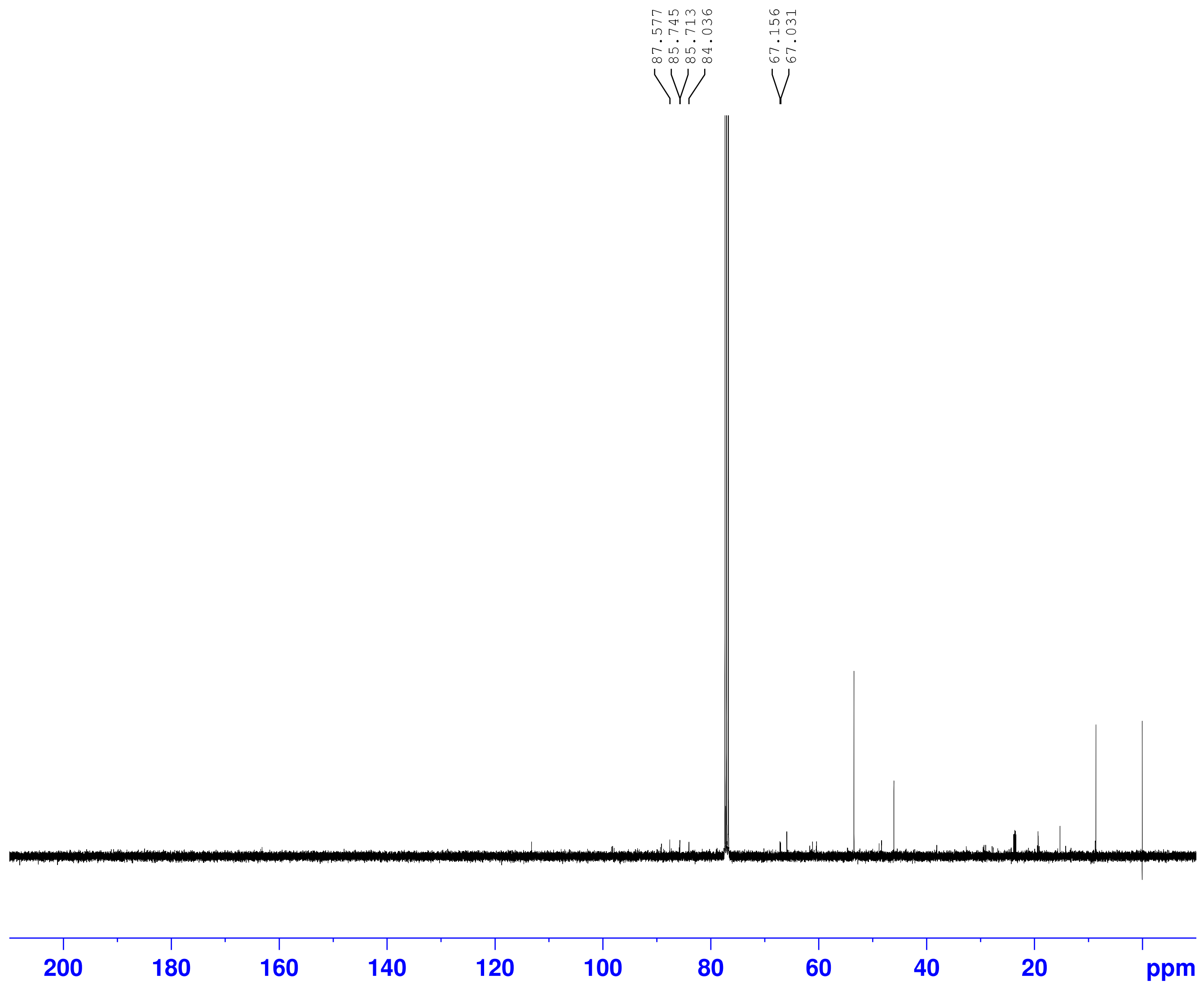


```
Current Data Parameters
NAME            kmd-5-159
EXPNO                   3
PROCNO                  1

F2 - Acquisition Parameters
Date_            20210223
Time                20.25 h
INSTRUM             spect
PROBHD   Z150354_0001 (
PULPROG           zgpg30
TD                 65356
SOLVENT            CDCl3
NS                   256
DS                     4
SWH            24038.461 Hz
FIDRES          0.735616 Hz
AQ             1.3594048 sec
RG                 29.21
DW                20.800 usec
DE                 25.00 usec
TE                 298.0 K
D1            2.00000000 sec
D11           0.03000000 sec
TD0                    1
SFO1         100.6655806 MHz
NUC1                 13C
P1                 10.00 usec
PLW1         19.75099945 W
SFO2         400.3016012 MHz
NUC2                  1H
CPDPRG[2         waltz16
PCPD2              80.00 usec
PLW2          4.67290020 W
PLW12         0.10514000 W
PLW13         0.05280100 W

F2 - Processing parameters
SI                131072
SF           100.6555147 MHz
WDW                   EM
SSB      0
LB       0 Hz
GB       0
PC                  1.40
```

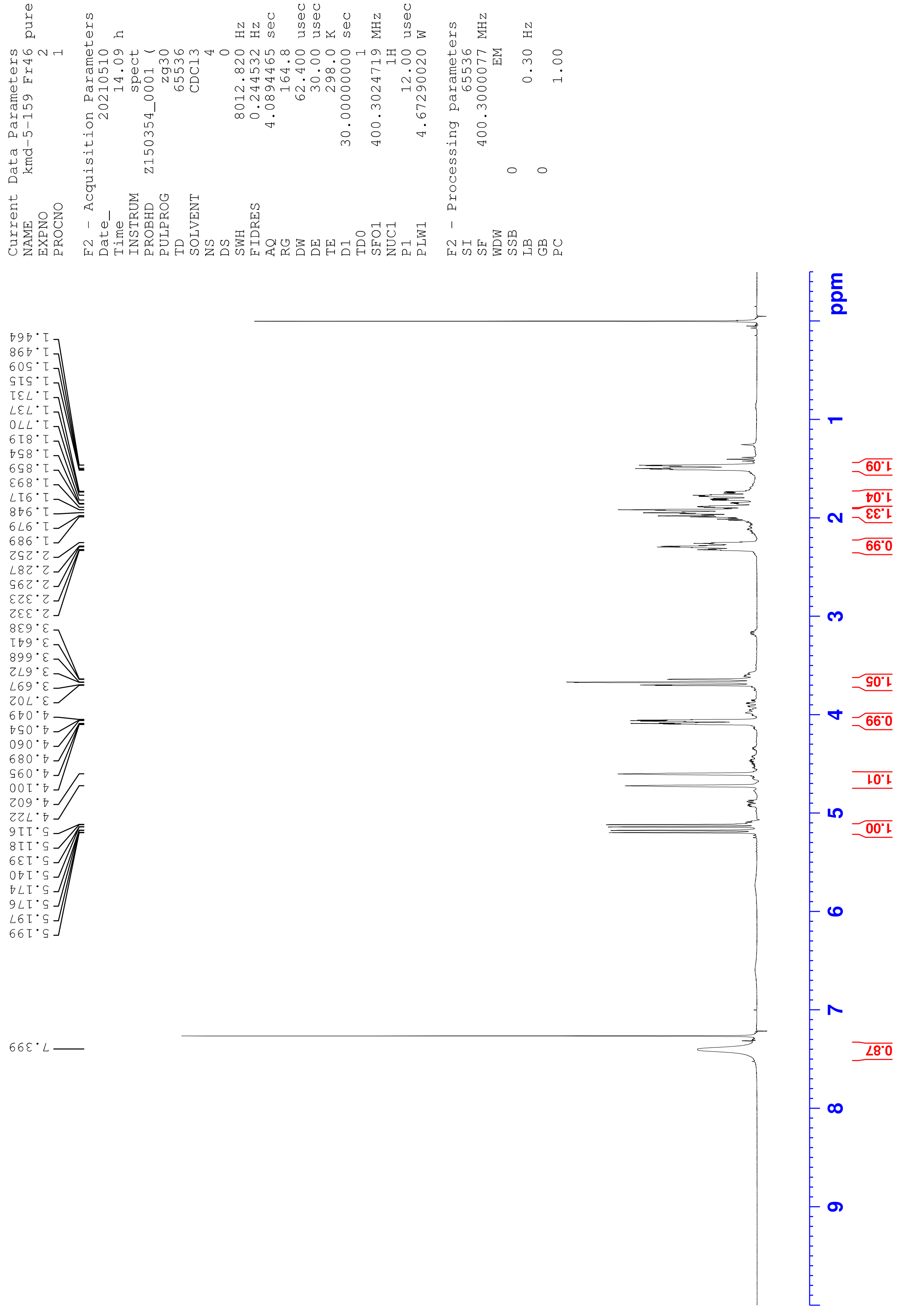
Current Data Parameters
NAME kmd-5-159 Fr46 pure
EXPNO 2
PROCNO 1
F2 - Acquisition Parameters
Date_ 20210510
Time 14.09 h
INSTRUM spect
PROBHD Z150354_0001 (
PULPROG zg30
TD 65536
SOLVENT CDCl3
NS 4
DS 0
SWH 8012.820 Hz
FIDRES 0.244532 Hz
AQ 4.0894465 sec
RG 164.8
DW 62.400 usec
DE 30.00 usec
TE 298.0 K
D1 30.00000000 sec
TD0 1
SFO1 400.3024719 MHz
NUC1 1H
P1 12.00 usec
PLW1 4.67290020 W
F2 - Processing parameters
SI 65536
SF 400.3000077 MHz
WDW EM
SSB 0
LB 0.30 Hz
GB 0
PC 1.00
1.464
1.498
1.509
1.515
1.731
1.737
1.770
1.819
1.854
1.859
1.893
1.917
1.948
1.979
1.989
2.252
2.287
2.295
2.323
2.332
3.638
3.641
3.668
3.672
3.697
3.702
4.049
4.054
4.060
4.089
4.095
4.100
4.602
4.722
5.116
5.118
5.139
5.140
5.174
5.176
5.197
5.199
7.399
1.09
1.04
1.33
0.99
1.05
0.99
1.01
1.00
0.87
ppm
1
2
3
4
5
6
7
8
9

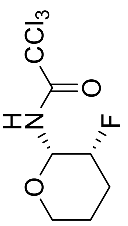
H
N
O
CCl3
F
O

cis-**S3**

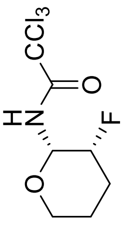

O
H
N
CCl_3
O
F

cis-**S3**

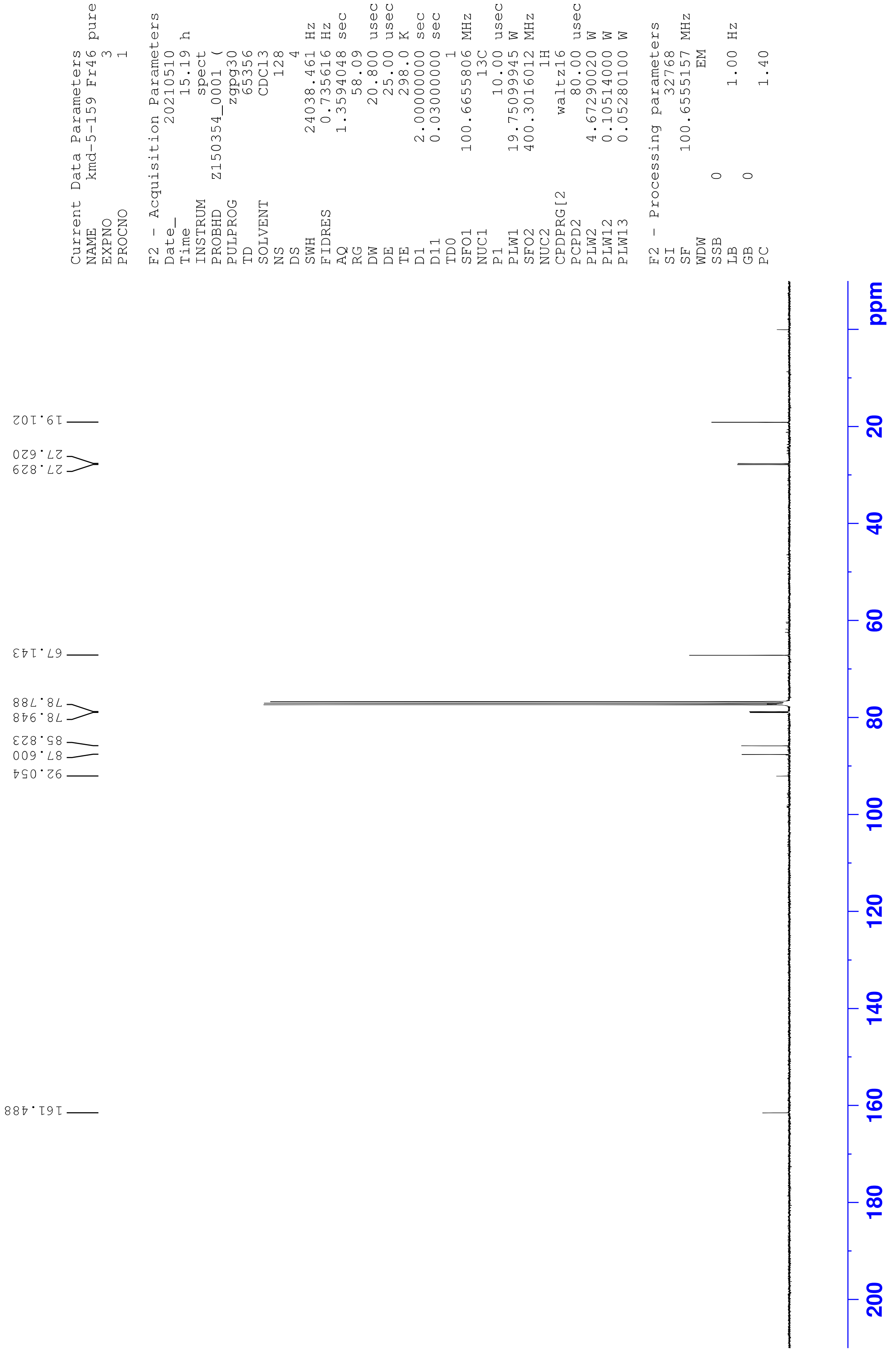

Current Data Parameters
NAME kmd-5-159 Fr46 pure
EXPNO 3
PROCNO 1
F2 - Acquisition Parameters
Date_ 20210510
Time 15.19 h
INSTRUM spect
PROBHD Z150354_0001 (
PULPROG zgpg30
TD 65356
SOLVENT CDCl3
NS 128
DS 4
SWH 24038.461 Hz
FIDRES 0.735616 Hz
AQ 1.3594048 sec
RG 58.09
DW 20.800 usec
DE 25.00 usec
TE 298.0 K
D1 2.00000000 sec
D11 0.03000000 sec
TD0 1
SFO1 100.6655806 MHz
NUC1 13C
P1 10.00 usec
PLW1 19.75099945 W
SFO2 400.3016012 MHz
NUC2 1H
CPDPRG[2 waltz16
PCPD2 80.00 usec
PLW2 4.67290020 W
PLW12 0.10514000 W
PLW13 0.05280100 W
F2 - Processing parameters
SI 32768
SF 100.6555157 MHz
WDW EM
SSB 0
LB 1.00 Hz
GB 0
PC 1.40
19.102
27.620
27.829
67.143
78.788
78.948
85.823
87.600
92.054
161.488
ppm
20
40
60
80
100
120
140
160
180
200

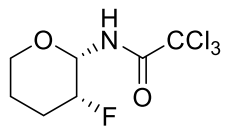

cis-**S3**

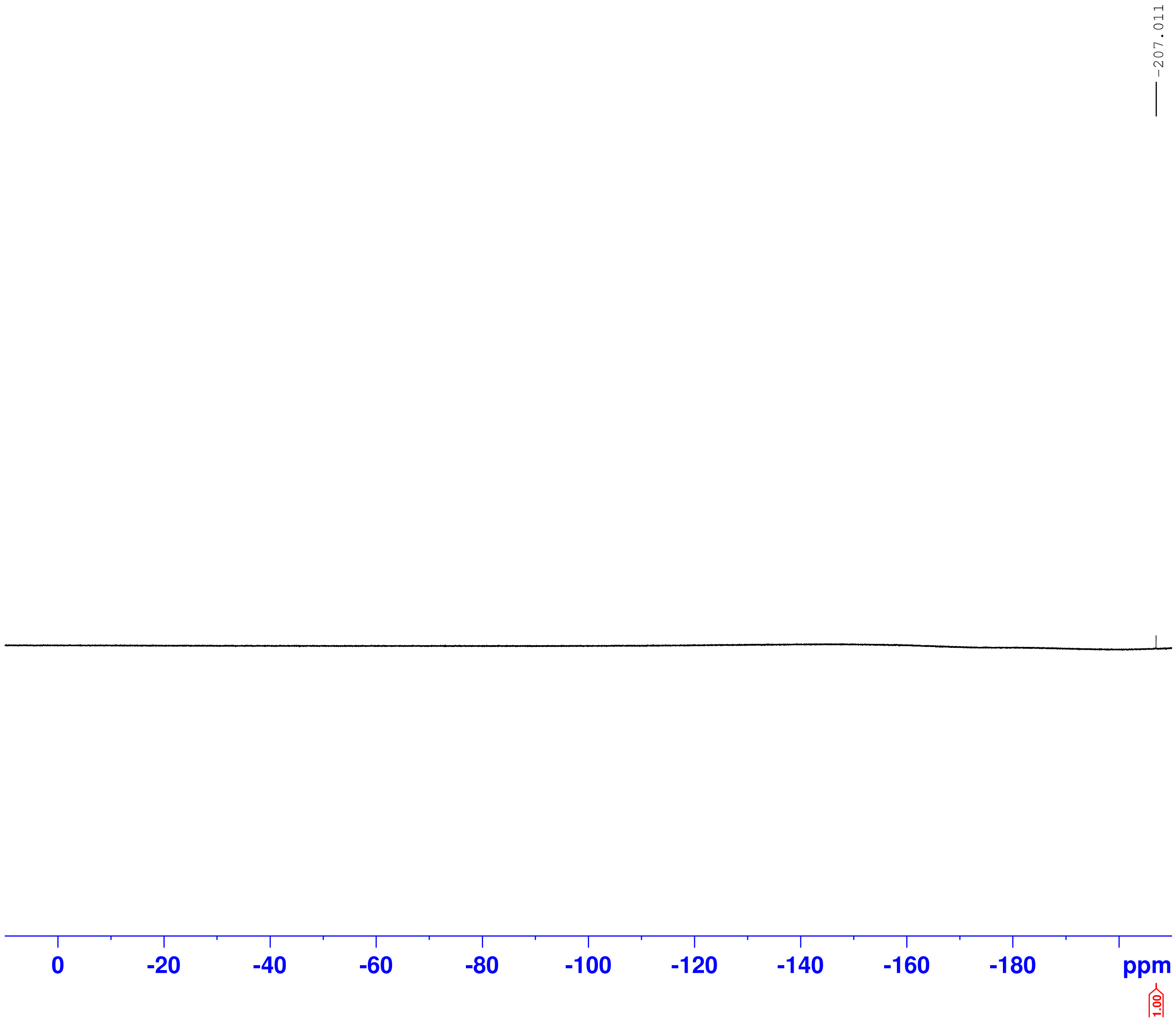


```
Current Data Parameters
NAME        kmd-5-159 Fr49-72 19F_2
EXPNO                     1
PROCNO                    1

F2 - Acquisition Parameters
Date_              20220502
Time                  15.16 h
INSTRUM               spect
PROBHD     Z133023_0002 (
PULPROG          zgfhigqn.2
TD                   131072
SOLVENT               CDCl3
NS                       16
DS                        4
SWH               89285.711 Hz
FIDRES             1.362392 Hz
AQ                0.7340032 sec
RG                   200.67
DW                    5.600 usec
DE                     6.50 usec
TE                    295.3 K
D1               1.00000000 sec
D11              0.03000000 sec
D12              0.00002000 sec
TD0                       1
SFO1            376.5453925 MHz
NUC1                    19F
P1                    16.00 usec
PLW1            16.42300034 W
SFO2            400.2216009 MHz
NUC2                     1H
CPDPRG[2            waltz16
PCPD2                 90.00 usec
PLW2            14.49600029 W
PLW12            0.45813999 W

F2 - Processing parameters
SI                    65536
SF              376.5834171 MHz
WDW                      EM
SSB      0
LB                     0.30 Hz
GB       0
PC                     1.00
```

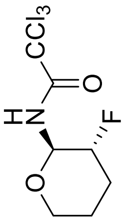

trans-**S3**

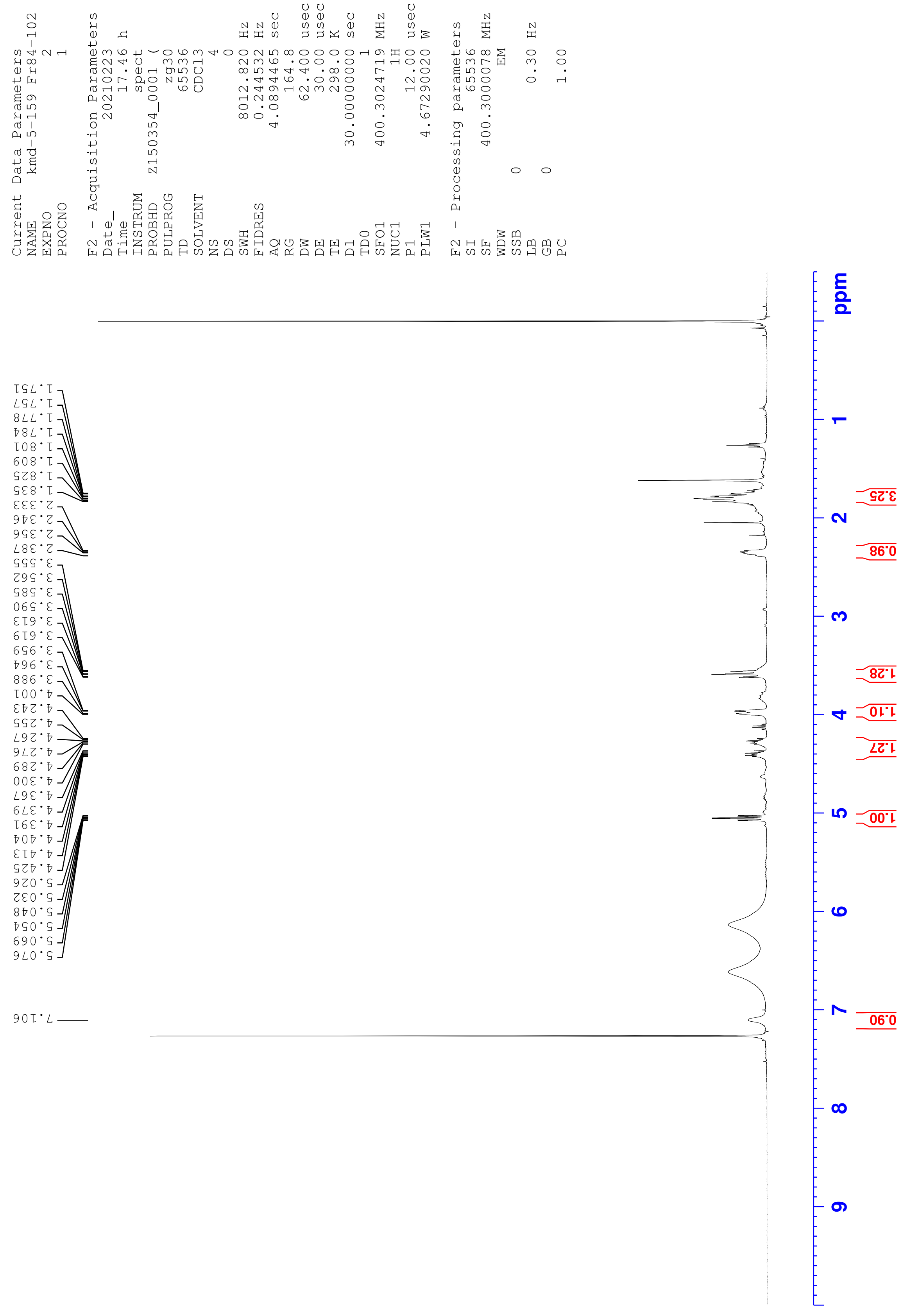
Current Data Parameters
NAME kmd-5-159 Fr84-102
EXPNO 2
PROCNO 1
F2 - Acquisition Parameters
Date_ 20210223
Time 17.46 h
INSTRUM spect
PROBHD Z150354_0001 (
PULPROG zg30
TD 65536
SOLVENT CDCl3
NS 4
DS 0
SWH 8012.820 Hz
FIDRES 0.244532 Hz
AQ 4.0894465 sec
RG 164.8
DW 62.400 usec
DE 30.00 usec
TE 298.0 K
D1 30.00000000 sec
TD0 1
SFO1 400.3024719 MHz
NUC1 1H
P1 12.00 usec
PLW1 4.67290020 W
F2 - Processing parameters
SI 65536
SF 400.3000078 MHz
WDW EM
SSB 0
LB 0.30 Hz
GB 0
PC 1.00
1.751
1.757
1.778
1.784
1.801
1.809
1.825
1.835
2.333
2.346
2.356
2.387
3.555
3.562
3.585
3.590
3.613
3.619
3.959
3.964
3.988
4.001
4.243
4.255
4.267
4.276
4.289
4.300
4.367
4.379
4.391
4.404
4.413
4.425
5.026
5.032
5.048
5.054
5.069
5.076
7.106
3.25
0.98
1.28
1.10
1.27
1.00
0.90
ppm
1
2
3
4
5
6
7
8
9

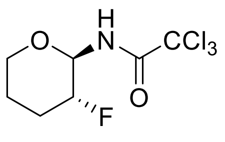

trans-**S3**

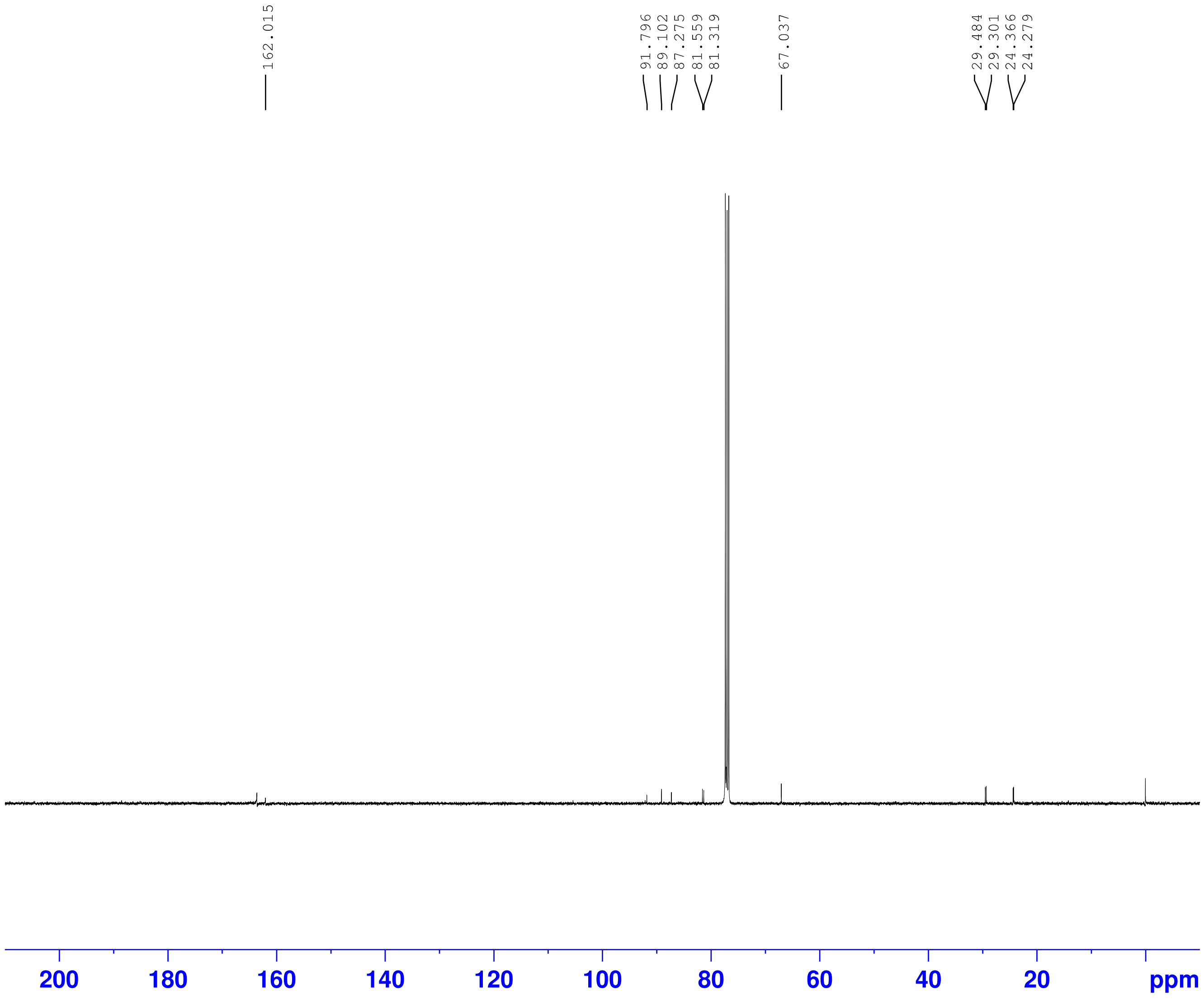


```
Current Data Parameters
NAME      kmd-5-159 Fr84-102
EXPNO                      3
PROCNO                     1

F2 - Acquisition Parameters
Date_               20210223
Time                   23.50 h
INSTRUM                spect
PROBHD     Z150354_0001 (
PULPROG              zgpg30
TD                    65356
SOLVENT               CDCl3
NS                      256
DS                        4
SWH               24038.461 Hz
FIDRES             0.735616 Hz
AQ                1.3594048 sec
RG                    29.21
DW                   20.800 usec
DE                    25.00 usec
TE                    298.0 K
D1               2.00000000 sec
D11              0.03000000 sec
TD0                       1
SFO1           100.6655806 MHz
NUC1                    13C
P1                    10.00 usec
PLW1            19.75099945 W
SFO2           400.3016012 MHz
NUC2                     1H
CPDPRG[2            waltz16
PCPD2                 80.00 usec
PLW2             4.67290020 W
PLW12            0.10514000 W
PLW13            0.05280100 W

F2 - Processing parameters
SI                    32768
SF             100.6555157 MHz
WDW                      EM
SSB       0
LB                     1.00 Hz
GB        0
PC                     1.40
```

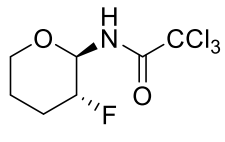

trans-**S3**

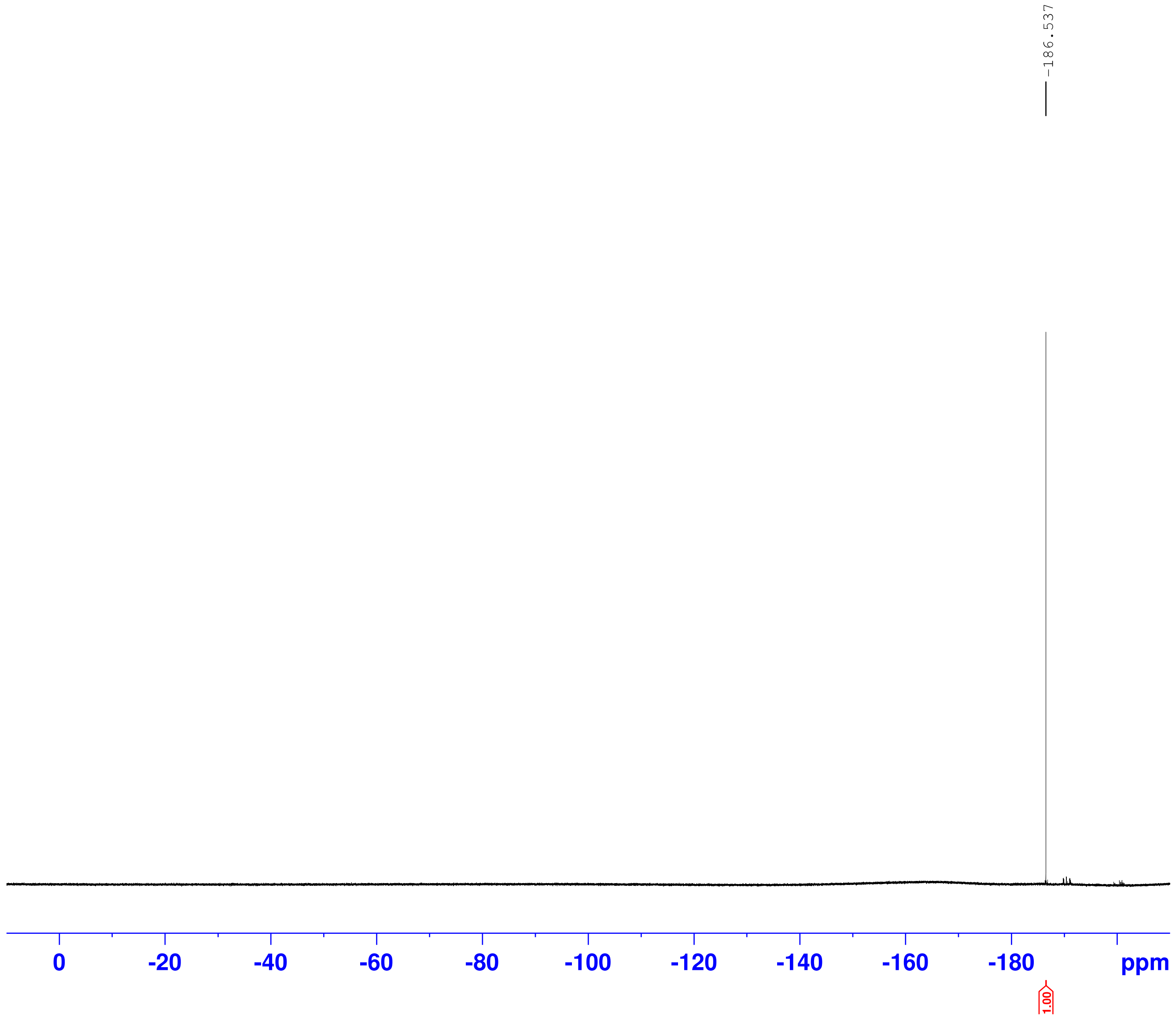

Current Data Parameters
NAME kmd-5-159 Fr84 19F
EXPNO 1
PROCNO 1

F2 - Acquisition Parameters
Date_ 20220627
Time 10.24 h
INSTRUM spect
PROBHD Z133023_0002 (
PULPROG zgfhigqn.2
TD 131072
SOLVENT CDCl3
NS 16
DS 4
SWH 89285.711 Hz
FIDRES 1.362392 Hz
AQ 0.7340032 sec
RG 200.67
DW 5.600 usec
DE 6.50 usec
TE 298.2 K
D1 1.00000000 sec
D11 0.03000000 sec
D12 0.00002000 sec
TD0 1
SFO1 376.5453925 MHz
NUC1 19F
P1 16.00 usec
PLW1 16.42300034 W
SFO2 400.2216009 MHz
NUC2 1H
CPDPRG[2 waltz16
PCPD2 90.00 usec
PLW2 14.49600029 W
PLW12 0.45813999 W

F2 - Processing parameters
SI 65536
SF 376.5834167 MHz
WDW EM
SSB 0
LB 0.30 Hz
GB 0
PC 1.00

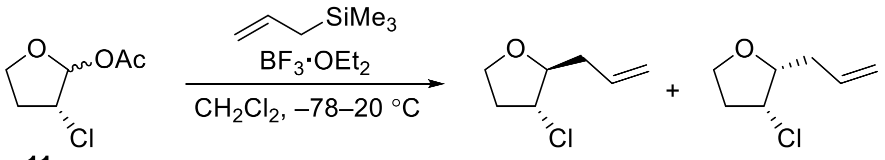

11 → *trans*-12 + *cis*-12

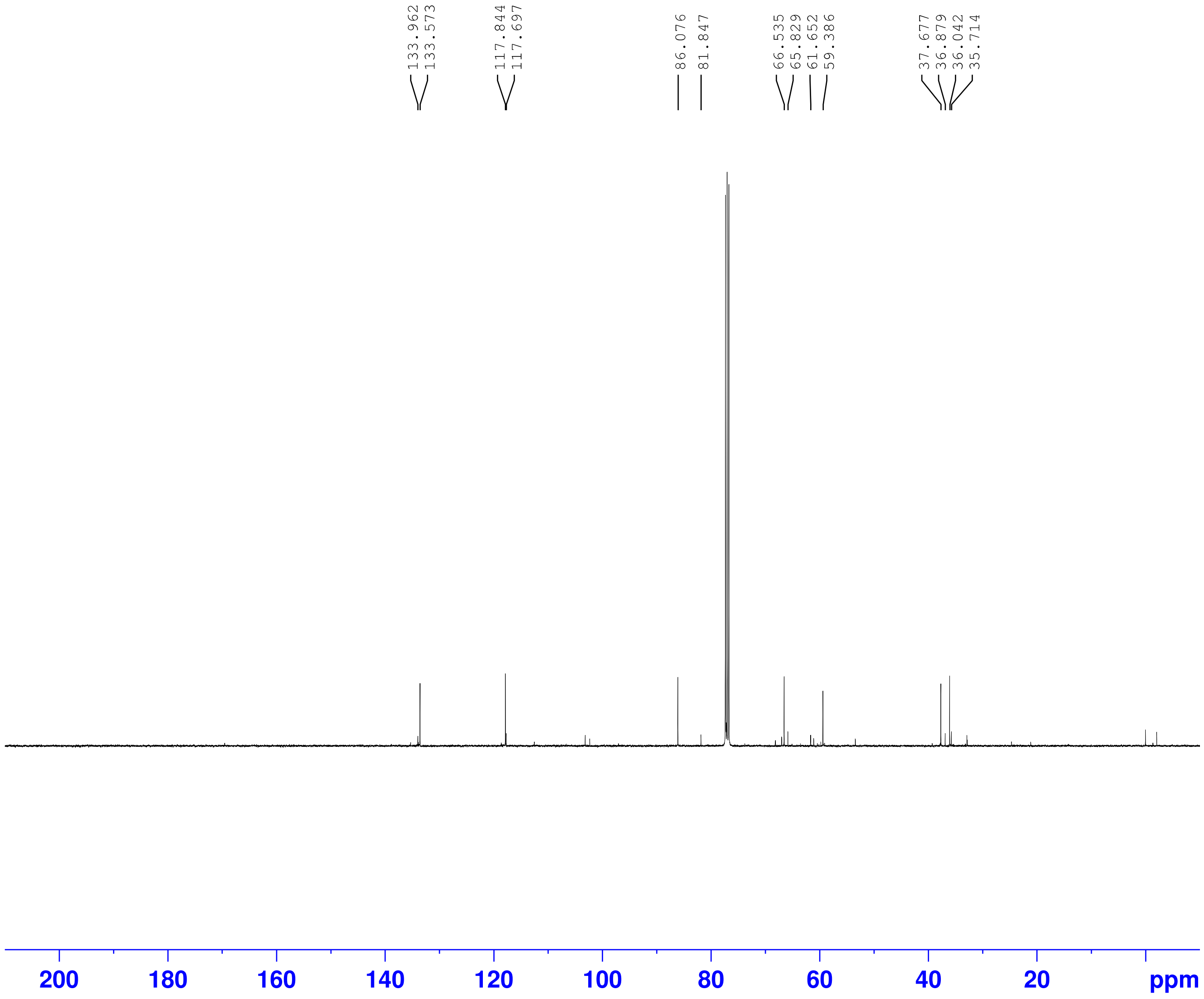


```
Current Data Parameters
NAME             kmd-7-061
EXPNO                    3
PROCNO                   1

F2 - Acquisition Parameters
Date_             20220616
Time                 18.05 h
INSTRUM              spect
PROBHD    Z150354_0001 (
PULPROG             zgpg30
TD                   65356
SOLVENT              CDCl3
NS                     256
DS                       4
SWH              24038.461 Hz
FIDRES            0.735616 Hz
AQ               1.3594048 sec
RG                   45.21
DW                  20.800 usec
DE                   25.00 usec
TE                   298.0 K
D1              2.00000000 sec
D11             0.03000000 sec
TD0                      1
SFO1           100.6655806 MHz
NUC1                   13C
P1                   10.00 usec
PLW1           17.99699974 W
SFO2           400.3016012 MHz
NUC2                    1H
CPDPRG[2           waltz16
PCPD2                80.00 usec
PLW2            4.64209986 W
PLW12           0.10445000 W
PLW13           0.05245300 W

F2 - Processing parameters
SI                   32768
SF             100.6555158 MHz
WDW                     EM
SSB      0
LB                    1.00 Hz
GB       0
PC                    1.40
```

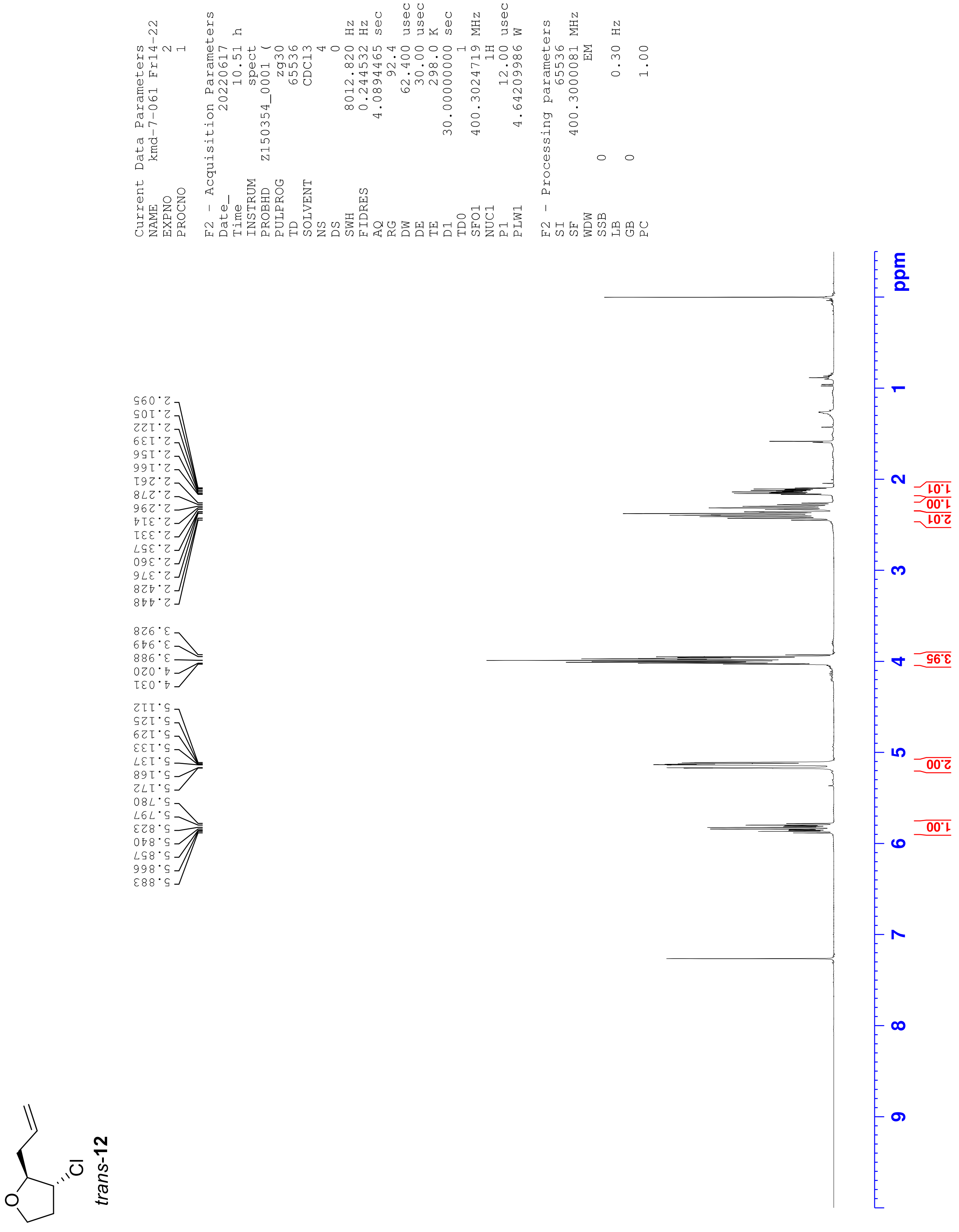
Current Data Parameters
NAME kmd-7-061 Fr14-22
EXPNO 2
PROCNO 1
F2 - Acquisition Parameters
Date_ 20220617
Time 10.51 h
INSTRUM spect
PROBHD Z150354_0001 (
PULPROG zg30
TD 65536
SOLVENT CDCl3
NS 4
DS 0
SWH 8012.820 Hz
FIDRES 0.244532 Hz
AQ 4.0894465 sec
RG 92.4
DW 62.400 usec
DE 30.00 usec
TE 298.0 K
D1 30.00000000 sec
TD0 1
SFO1 400.3024719 MHz
NUC1 1H
P1 12.00 usec
PLW1 4.64209986 W
F2 - Processing parameters
SI 65536
SF 400.3000081 MHz
WDW EM
SSB 0
LB 0.30 Hz
GB 0
PC 1.00
2.095
2.105
2.122
2.139
2.156
2.166
2.261
2.278
2.296
2.314
2.331
2.357
2.360
2.376
2.428
2.448
3.928
3.949
3.988
4.020
4.031
5.112
5.125
5.129
5.133
5.137
5.168
5.172
5.780
5.797
5.823
5.840
5.857
5.866
5.883
1.01
1.00
2.01
3.95
2.00
1.00
ppm
1
2
3
4
5
6
7
8
9
Cl
O
trans-12

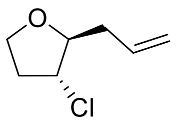

trans-**12**

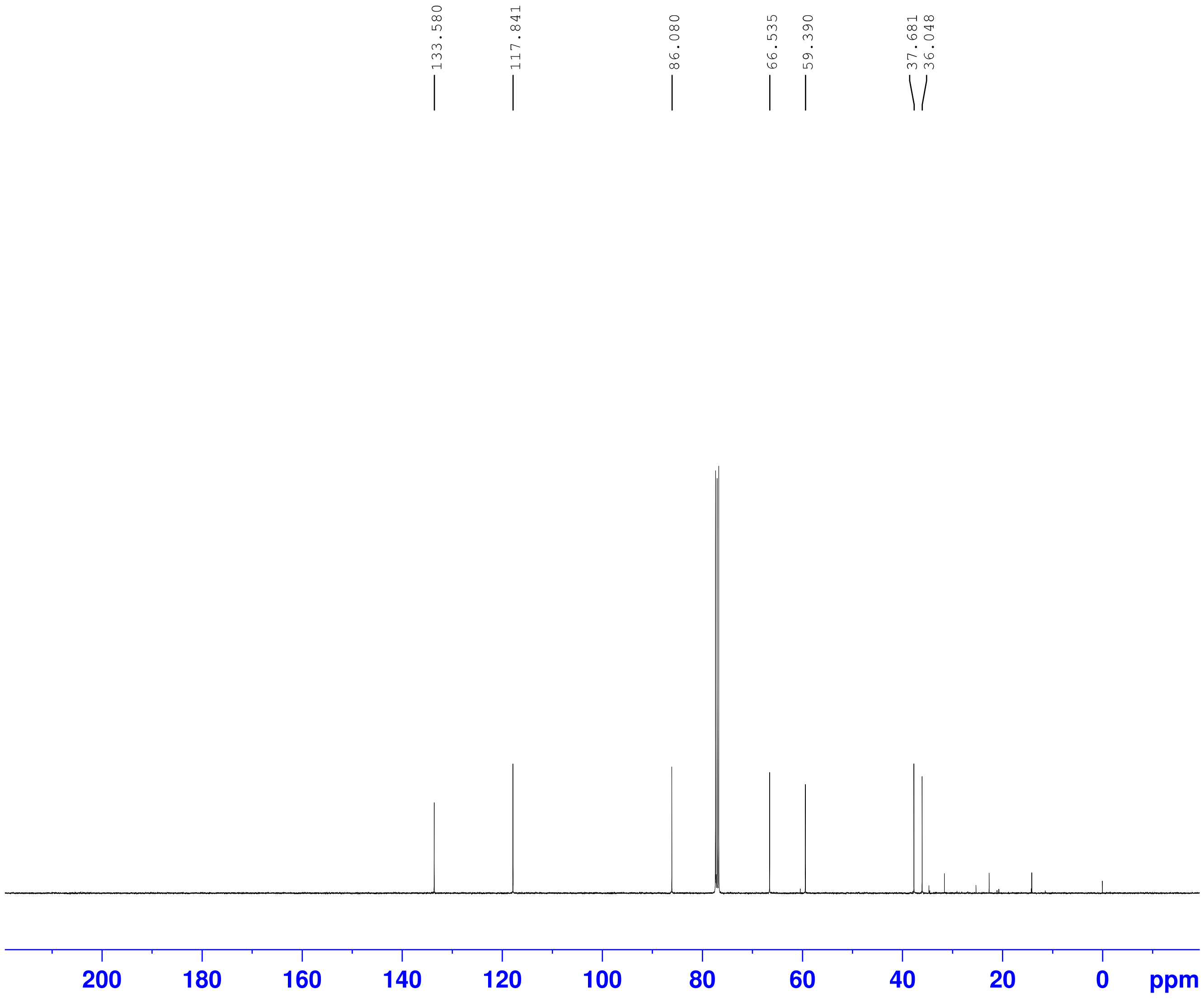


```
Current Data Parameters
NAME      kmd-5-166 Fr8-10
EXPNO                    3
PROCNO                   1

F2 - Acquisition Parameters
Date_             20210302
Time                 17.41 h
INSTRUM              spect
PROBHD    Z150354_0001 (
PULPROG            zgpg30
TD                  65356
SOLVENT             CDCl3
NS                    256
DS                      4
SWH             24038.461 Hz
FIDRES           0.735616 Hz
AQ              1.3594048 sec
RG                  45.21
DW                 20.800 usec
DE                  25.00 usec
TE                  298.0 K
D1             2.00000000 sec
D11            0.03000000 sec
TD0                     1
SFO1          100.6655806 MHz
NUC1                  13C
P1                  10.00 usec
PLW1          19.75099945 W
SFO2          400.3016012 MHz
NUC2                   1H
CPDPRG[2          waltz16
PCPD2               80.00 usec
PLW2           4.67290020 W
PLW12          0.10514000 W
PLW13          0.05280100 W

F2 - Processing parameters
SI                  32768
SF            100.6555154 MHz
WDW                    EM
SSB       0
LB                   1.00 Hz
GB        0
PC                   1.40
```

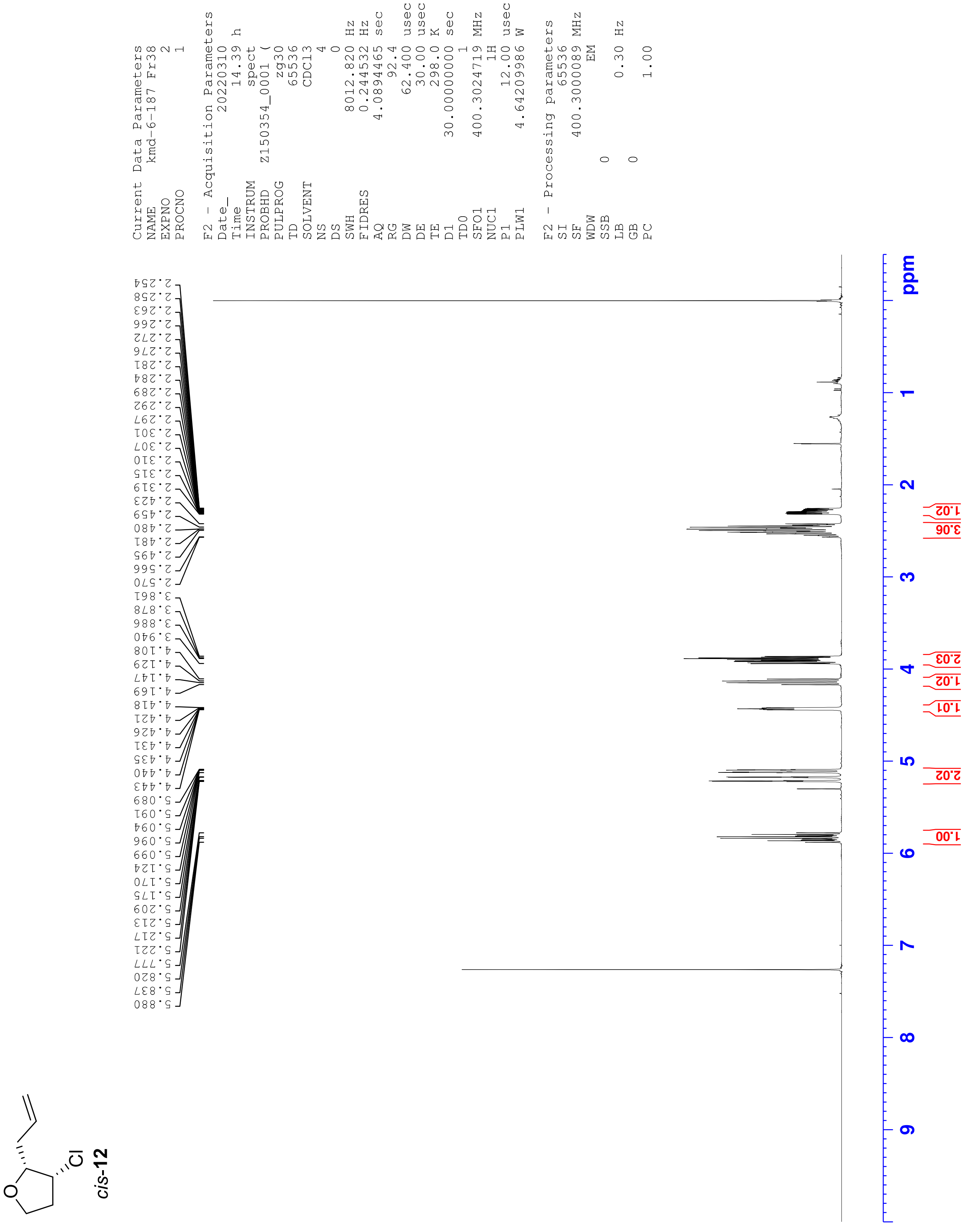
Current Data Parameters
NAME kmd-6-187 Fr38
EXPNO 2
PROCNO 1
F2 - Acquisition Parameters
Date_ 20220310
Time 14.39 h
INSTRUM spect
PROBHD Z150354_0001 (
PULPROG zg30
TD 65536
SOLVENT CDCl3
NS 4
DS 0
SWH 8012.820 Hz
FIDRES 0.244532 Hz
AQ 4.0894465 sec
RG 92.4
DW 62.400 usec
DE 30.00 usec
TE 298.0 K
D1 30.00000000 sec
TD0 1
SFO1 400.3024719 MHz
NUC1 1H
P1 12.00 usec
PLW1 4.64209986 W
F2 - Processing parameters
SI 65536
SF 400.3000089 MHz
WDW EM
SSB 0
LB 0.30 Hz
GB 0
PC 1.00
ppm

cis-12

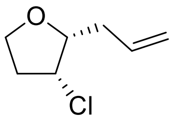

cis-**12**

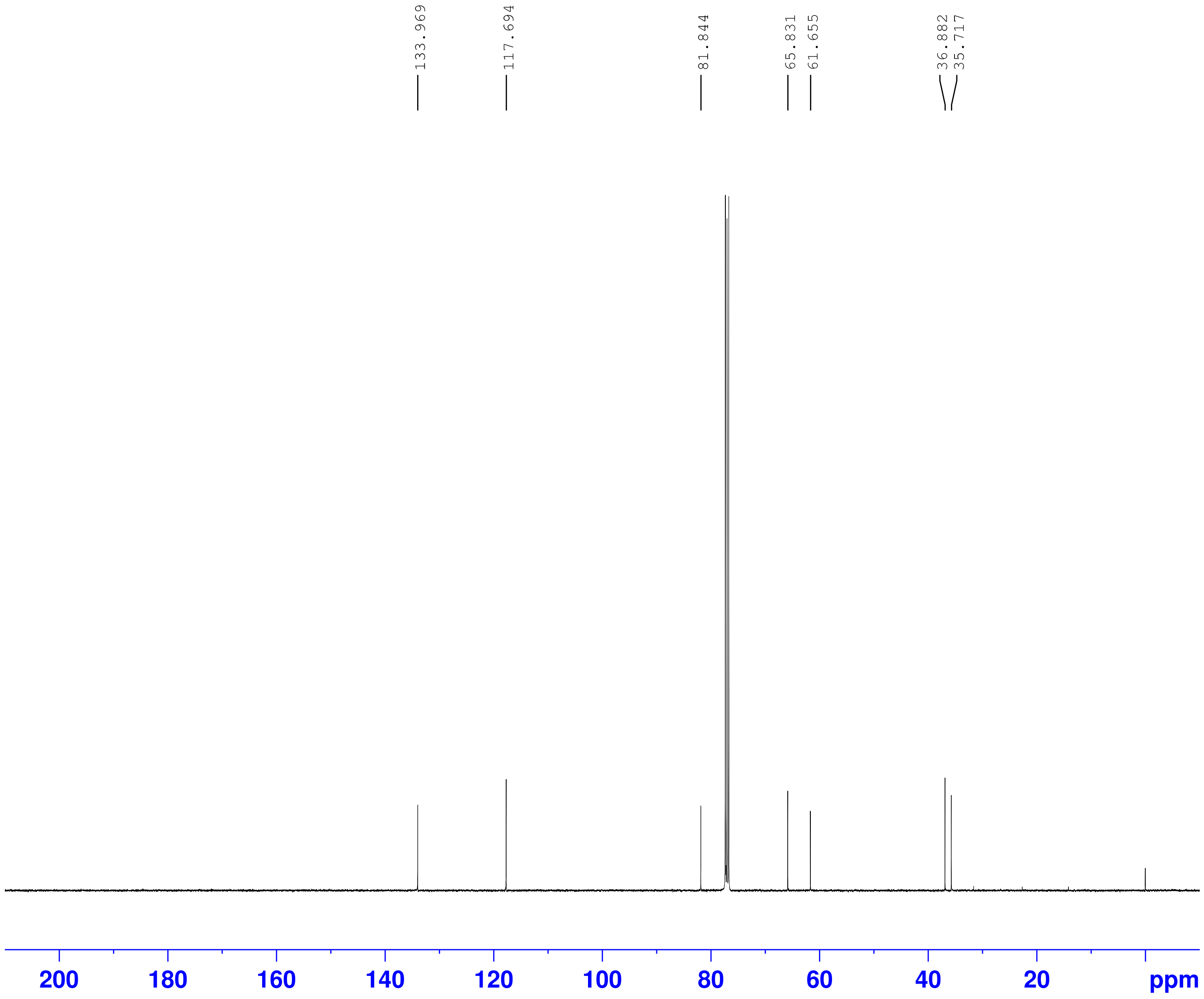


```
Current Data Parameters
NAME        kmd-6-187 Fr38
EXPNO                    3
PROCNO                   1

F2 - Acquisition Parameters
Date_             20220310
Time                 21.23 h
INSTRUM              spect
PROBHD    Z150354_0001 (
PULPROG            zgpg30
TD                   65356
SOLVENT              CDCl3
NS                     256
DS                       4
SWH              24038.461 Hz
FIDRES            0.735616 Hz
AQ               1.3594048 sec
RG                   51.78
DW                  20.800 usec
DE                   25.00 usec
TE                   298.0 K
D1              2.00000000 sec
D11             0.03000000 sec
TD0                      1
SFO1          100.6655806 MHz
NUC1                   13C
P1                   10.00 usec
PLW1           17.99699974 W
SFO2          400.3016012 MHz
NUC2                    1H
CPDPRG[2           waltz16
PCPD2                80.00 usec
PLW2            4.64209986 W
PLW12           0.10445000 W
PLW13           0.05245300 W

F2 - Processing parameters
SI                   32768
SF            100.6555157 MHz
WDW                     EM
SSB       0
LB                    1.00 Hz
GB        0
PC                    1.40
```

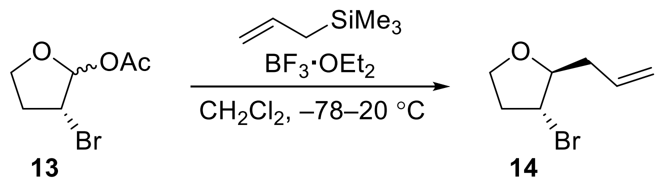

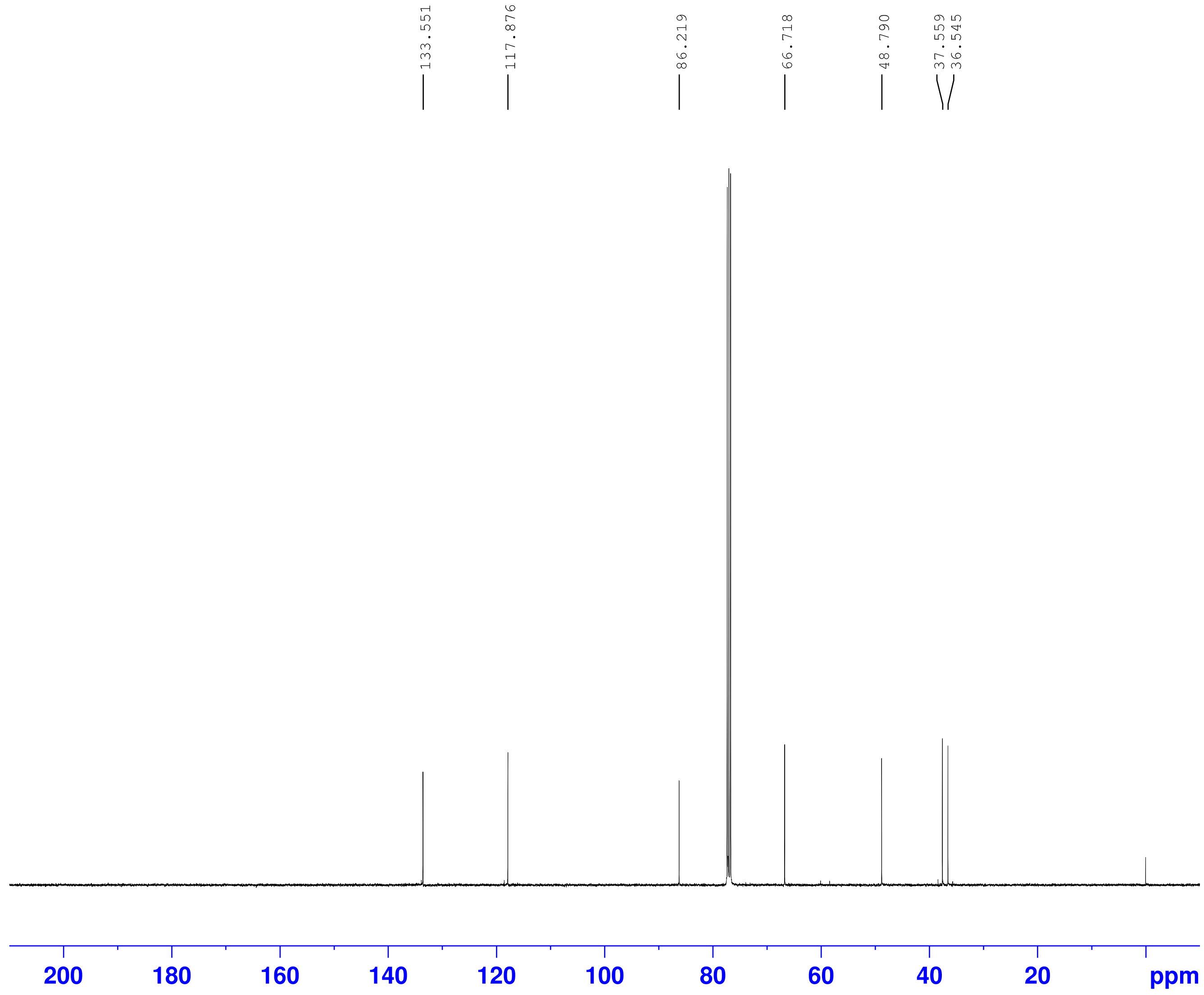


```
Current Data Parameters
NAME              kmd-3-189
EXPNO                     3
PROCNO                    1

F2 - Acquisition Parameters
Date_              20191126
Time                  12.24 h
INSTRUM               spect
PROBHD   Z150354_0001 (
PULPROG             zgpg30
TD                   65356
SOLVENT              CDCl3
NS                     256
DS                       4
SWH              24038.461 Hz
FIDRES            0.735616 Hz
AQ               1.3594048 sec
RG                   39.47
DW                  20.800 usec
DE                   25.00 usec
TE                   298.0 K
D1              2.00000000 sec
D11             0.03000000 sec
TD0                      1
SFO1           100.6655806 MHz
NUC1                   13C
P1                   10.00 usec
PLW1           19.75099945 W
SFO2           400.3016012 MHz
NUC2                    1H
CPDPRG[2           waltz16
PCPD2                80.00 usec
PLW2            4.67290020 W
PLW12           0.10514000 W
PLW13           0.05280100 W

F2 - Processing parameters
SI                   32768
SF             100.6555157 MHz
WDW                     EM
SSB      0
LB                    1.00 Hz
GB       0
PC                    1.40
```

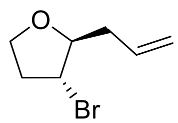

14

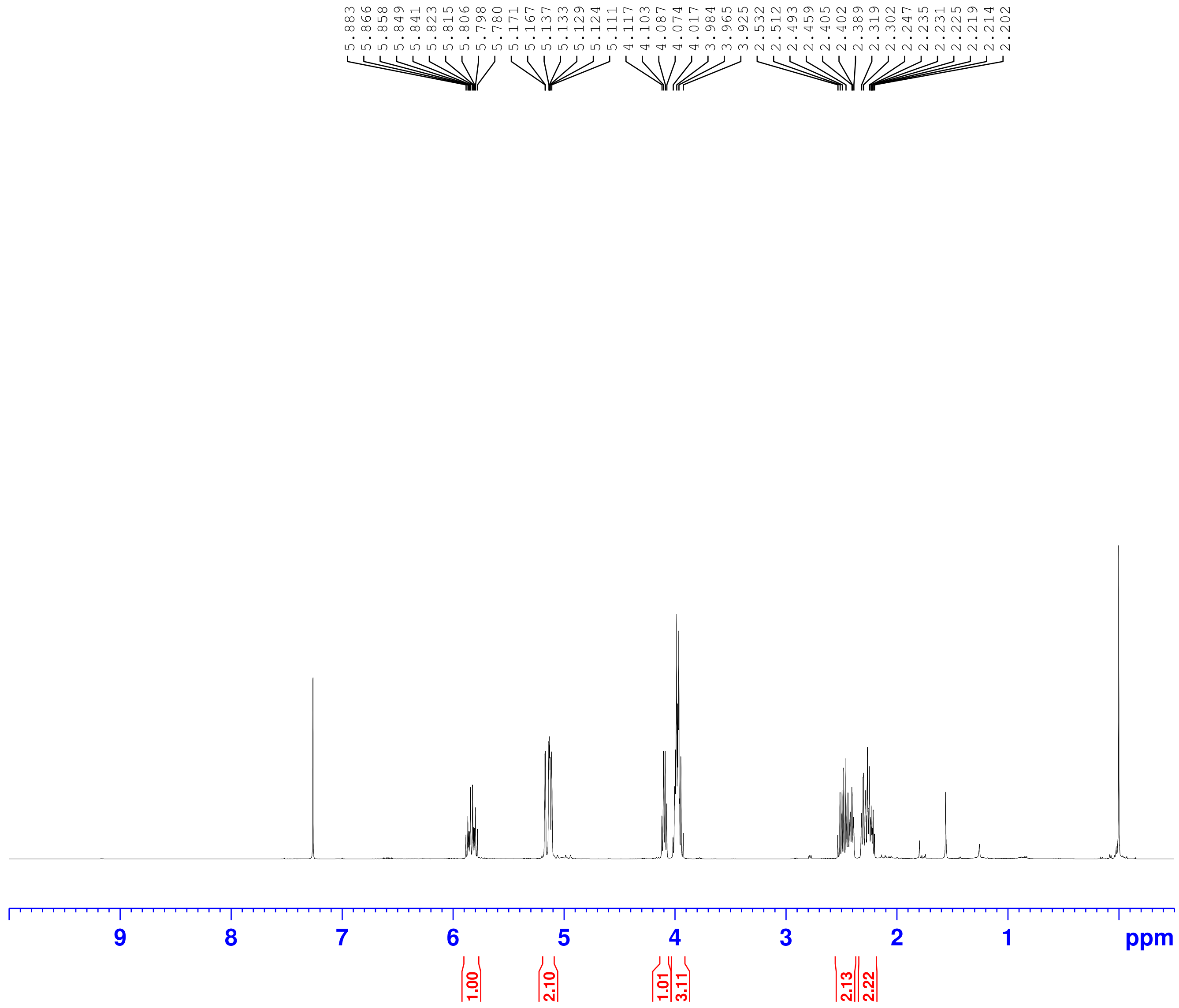


```
Current Data Parameters
NAME        kmd-3-189 plug
EXPNO                    1
PROCNO                   1

F2 - Acquisition Parameters
Date_             20191126
Time                 15.55 h
INSTRUM              spect
PROBHD      Z150354_0001 (
PULPROG               zg30
TD                   65536
SOLVENT              CDCl3
NS                      16
DS                       2
SWH               8012.820 Hz
FIDRES            0.244532 Hz
AQ               4.0894465 sec
RG                  117.47
DW                  62.400 usec
DE                   30.00 usec
TE                   298.0 K
D1              1.00000000 sec
TD0                      1
SFO1           400.3024719 MHz
NUC1                    1H
P1                   12.00 usec
PLW1            4.67290020 W

F2 - Processing parameters
SI                   65536
SF             400.3000086 MHz
WDW                     EM
SSB         0
LB                    0.30 Hz
GB          0
PC                    1.00
```

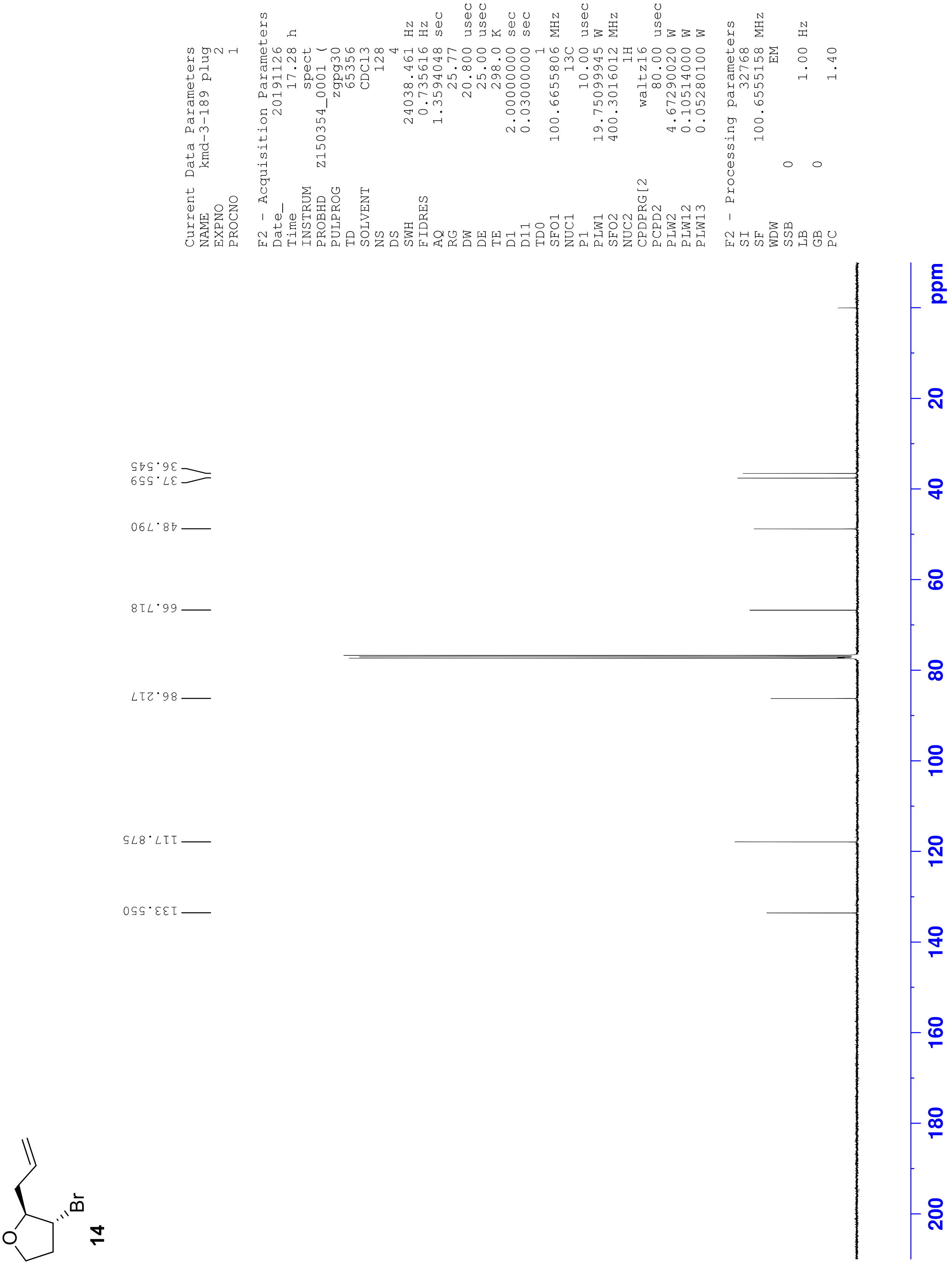
Current Data Parameters
NAME kmd-3-189 plug
EXPNO 2
PROCNO 1
F2 - Acquisition Parameters
Date_ 20191126
Time 17.28 h
INSTRUM spect
PROBHD Z150354_0001 (
PULPROG zgpg30
TD 65356
SOLVENT CDCl3
NS 128
DS 4
SWH 24038.461 Hz
FIDRES 0.735616 Hz
AQ 1.3594048 sec
RG 25.77
DW 20.800 usec
DE 25.00 usec
TE 298.0 K
D1 2.00000000 sec
D11 0.03000000 sec
TD0 1
SFO1 100.6655806 MHz
NUC1 13C
P1 10.00 usec
PLW1 19.75099945 W
SFO2 400.3016012 MHz
NUC2 1H
CPDPRG[2 waltz16
PCPD2 80.00 usec
PLW2 4.67290020 W
PLW12 0.10514000 W
PLW13 0.05280100 W
F2 - Processing parameters
SI 32768
SF 100.6555158 MHz
WDW EM
SSB 0
LB 1.00 Hz
GB 0
PC 1.40
36.545
37.559
48.790
66.718
86.217
117.875
133.550
ppm
20
40
60
80
100
120
140
160
180
200
O
Br
14

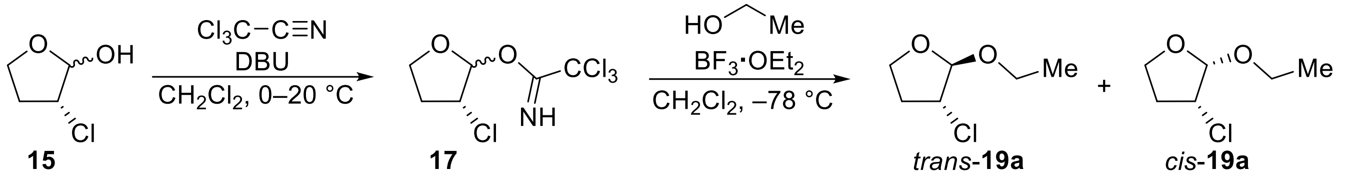

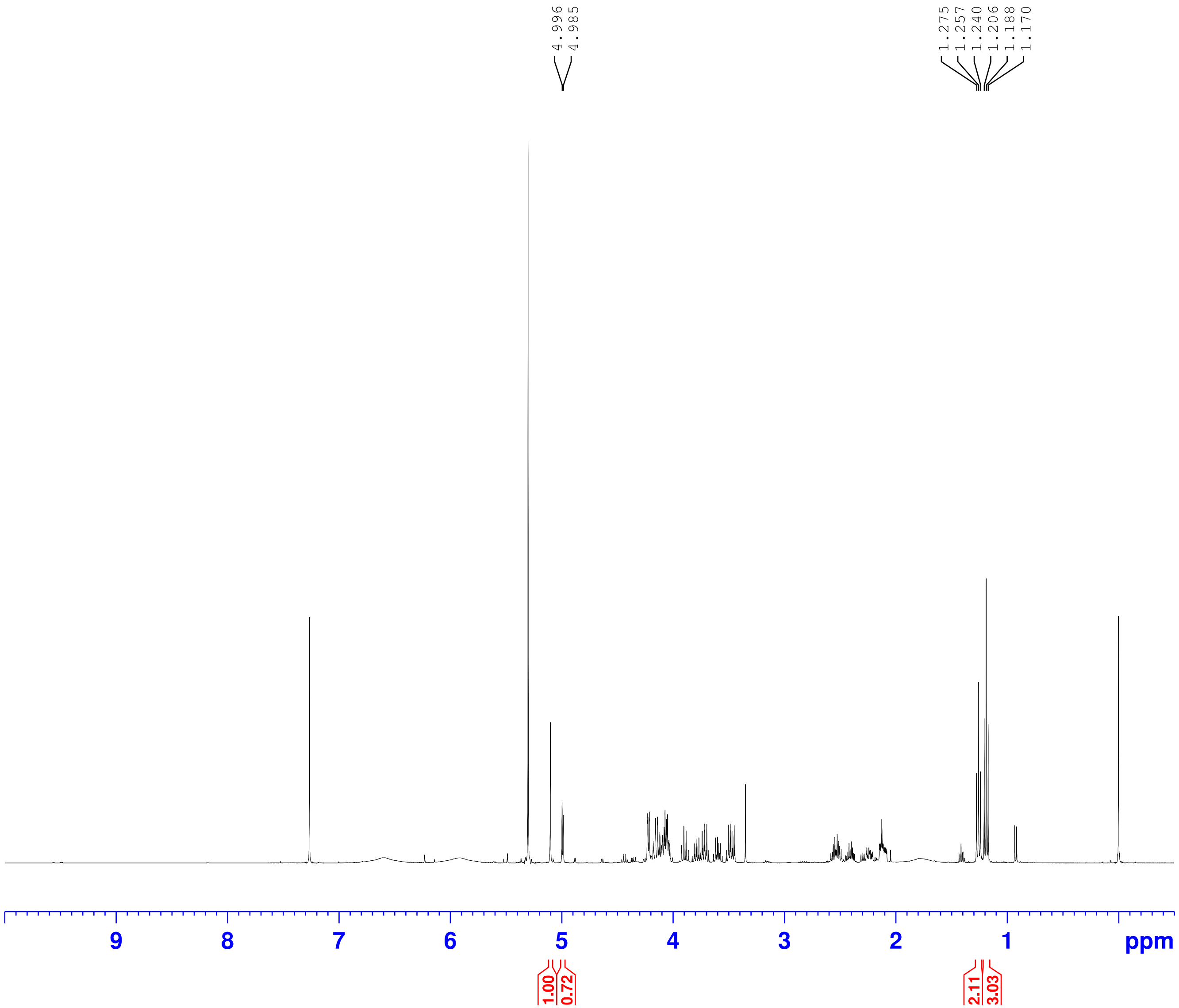


```
Current Data Parameters
NAME              kmd-5-046
EXPNO                     2
PROCNO                    1

F2 - Acquisition Parameters
Date_              20201109
Time                  18.03 h
INSTRUM               spect
PROBHD   Z150354_0001 (
PULPROG                zg30
TD                    65536
SOLVENT               CDCl3
NS                        4
DS                        0
SWH                8012.820 Hz
FIDRES             0.244532 Hz
AQ                4.0894465 sec
RG                     92.4
DW                   62.400 usec
DE                    30.00 usec
TE                    298.0 K
D1              30.00000000 sec
TD0                       1
SFO1            400.3024719 MHz
NUC1                     1H
P1                    12.00 usec
PLW1             4.67290020 W

F2 - Processing parameters
SI                    65536
SF              400.3000079 MHz
WDW                      EM
SSB      0
LB                     0.30 Hz
GB       0
PC                     1.00
```

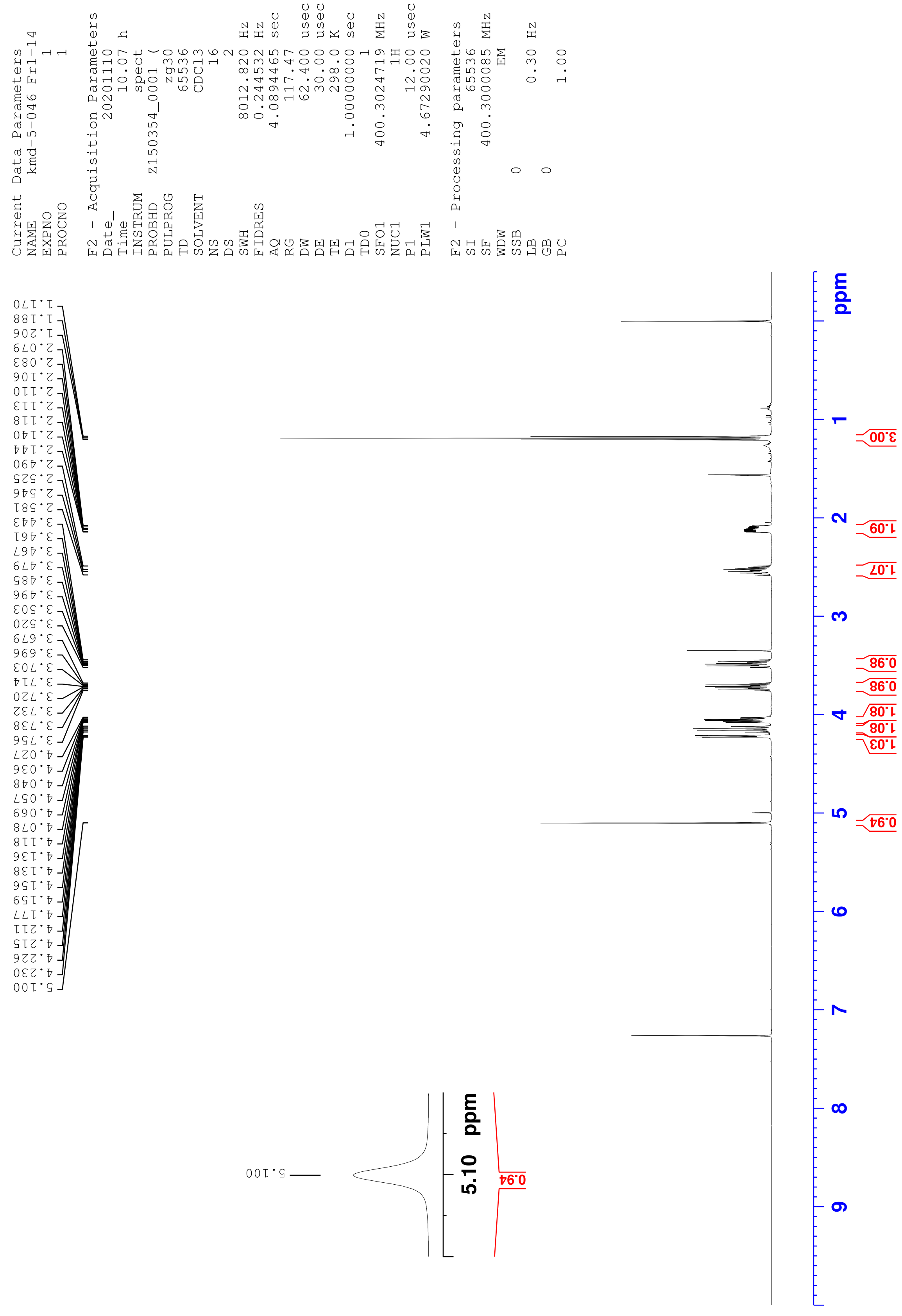
Current Data Parameters
NAME kmd-5-046 Fr1-14
EXPNO 1
PROCNO 1
F2 - Acquisition Parameters
Date_ 20201110
Time 10.07 h
INSTRUM spect
PROBHD Z150354_0001 (
PULPROG zg30
TD 65536
SOLVENT CDCl3
NS 16
DS 2
SWH 8012.820 Hz
FIDRES 0.244532 Hz
AQ 4.0894465 sec
RG 117.47
DW 62.400 usec
DE 30.00 usec
TE 298.0 K
D1 1.00000000 sec
TD0 1
SFO1 400.3024719 MHz
NUC1 1H
P1 12.00 usec
PLW1 4.67290020 W
F2 - Processing parameters
SI 65536
SF 400.3000085 MHz
WDW EM
SSB 0
LB 0.30 Hz
GB 0
PC 1.00
1.170
1.188
1.206
2.079
2.083
2.106
2.110
2.113
2.118
2.140
2.144
2.490
2.525
2.546
2.581
3.443
3.461
3.467
3.479
3.485
3.496
3.503
3.520
3.679
3.696
3.703
3.714
3.720
3.732
3.738
3.756
4.027
4.036
4.048
4.057
4.069
4.078
4.118
4.136
4.138
4.156
4.159
4.177
4.211
4.215
4.226
4.230
5.100
5.100
5.10 ppm
0.94
3.00
1.09
1.07
0.98
0.98
1.08
1.08
1.03
0.94
ppm
1
2
3
4
5
6
7
8
9

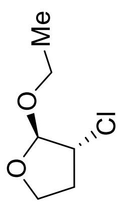
O
O
Me
Cl

trans-**19a**

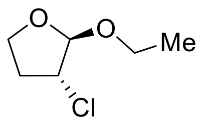

trans-**19a**

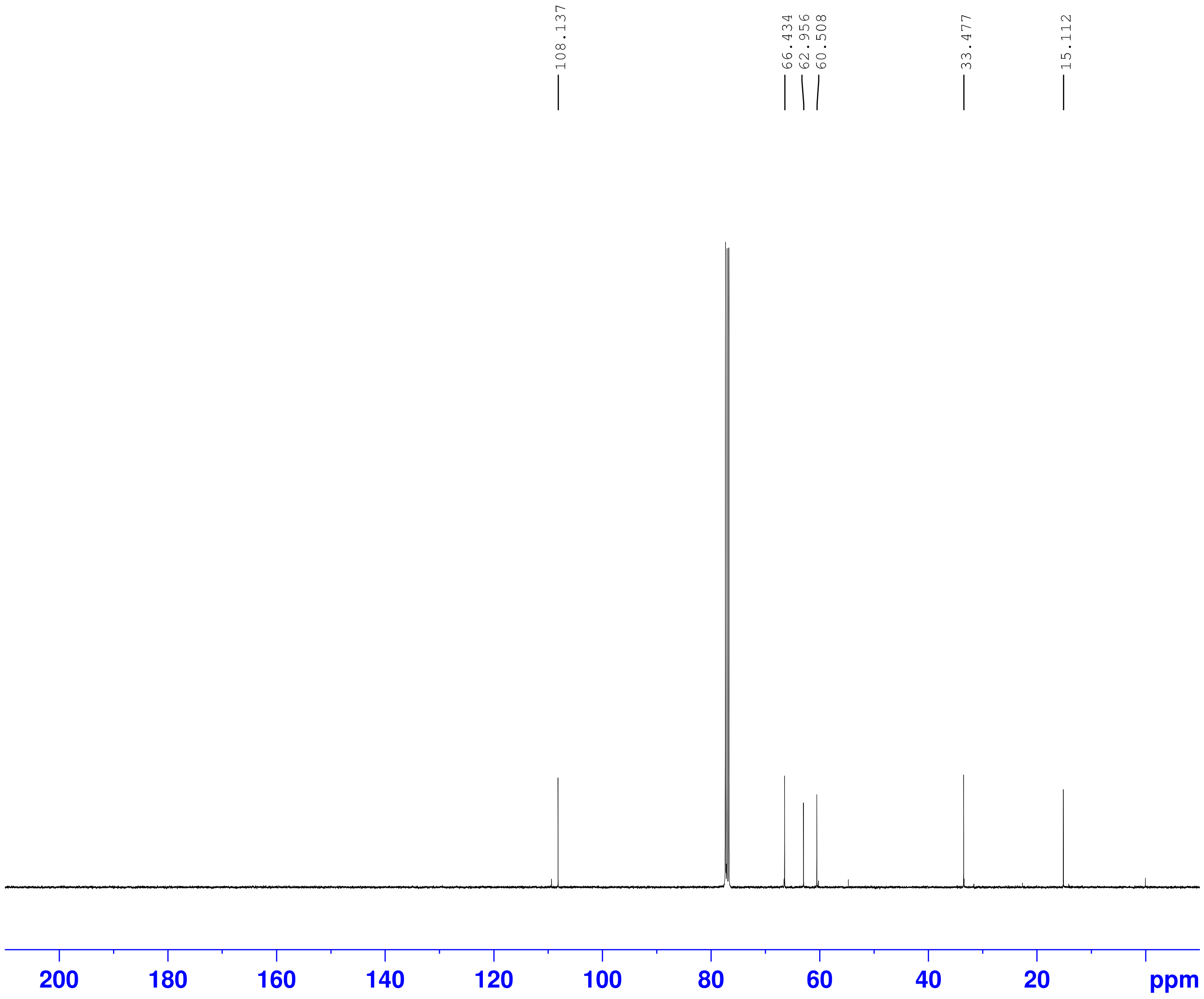


```
Current Data Parameters
NAME      kmd-5-046 Fr1-14
EXPNO                    3
PROCNO                   1

F2 - Acquisition Parameters
Date_             20201110
Time                 10.36 h
INSTRUM              spect
PROBHD   Z150354_0001 (
PULPROG            zgpg30
TD                  65356
SOLVENT             CDCl3
NS                    256
DS                      4
SWH             24038.461 Hz
FIDRES           0.735616 Hz
AQ              1.3594048 sec
RG                  36.68
DW                 20.800 usec
DE                  25.00 usec
TE                  298.0 K
D1             2.00000000 sec
D11            0.03000000 sec
TD0                     1
SFO1         100.6655806 MHz
NUC1                  13C
P1                  10.00 usec
PLW1          19.75099945 W
SFO2          400.3016012 MHz
NUC2                   1H
CPDPRG[2          waltz16
PCPD2               80.00 usec
PLW2           4.67290020 W
PLW12          0.10514000 W
PLW13          0.05280100 W

F2 - Processing parameters
SI                  32768
SF           100.6555151 MHz
WDW                    EM
SSB       0
LB                   1.00 Hz
GB        0
PC                   1.40
```

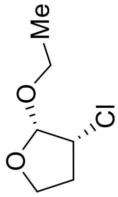

cis-**19a**

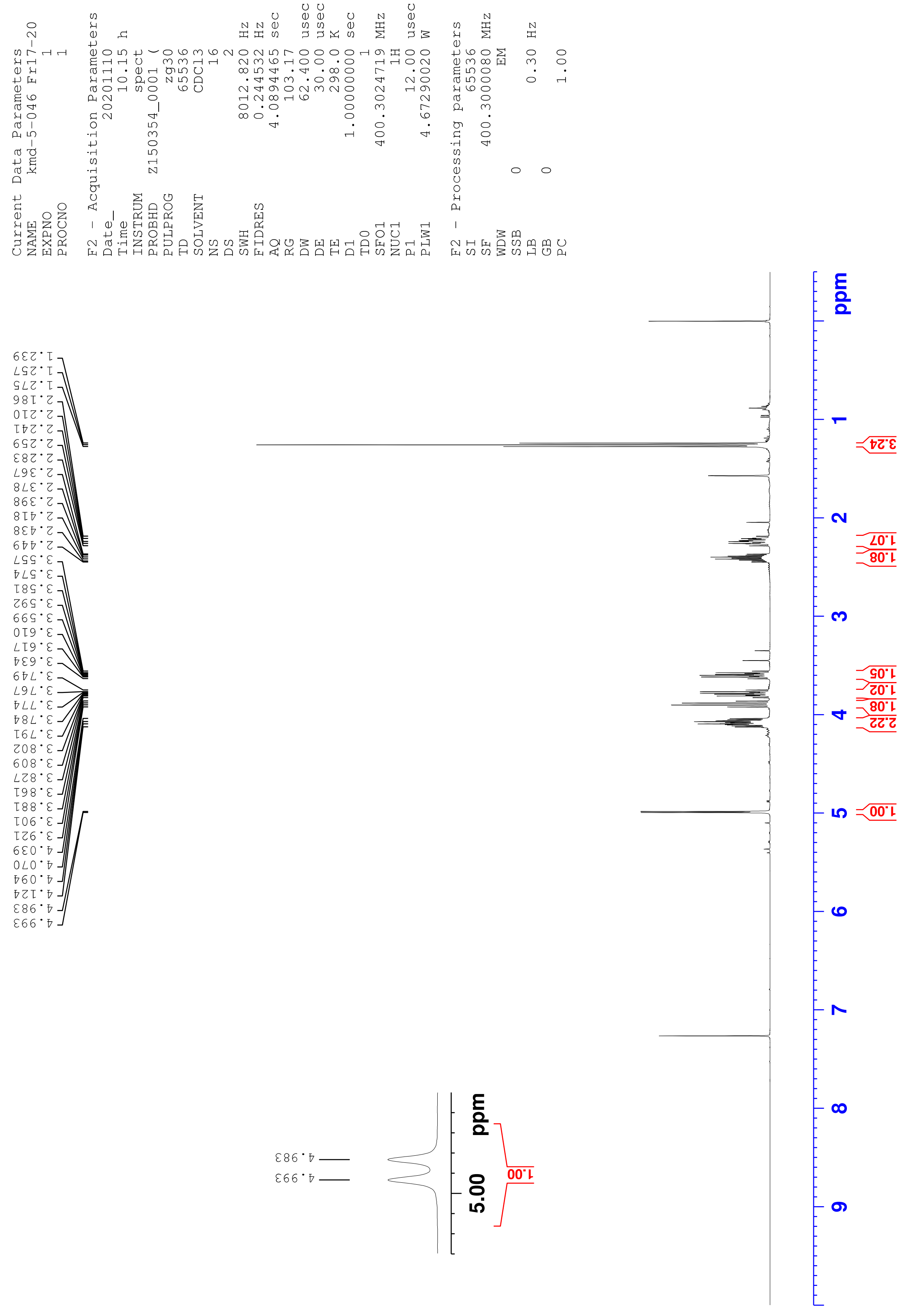
Current Data Parameters
NAME kmd-5-046 Fr17-20
EXPNO 1
PROCNO 1
F2 - Acquisition Parameters
Date_ 20201110
Time 10.15 h
INSTRUM spect
PROBHD Z150354_0001 (
PULPROG zg30
TD 65536
SOLVENT CDCl3
NS 16
DS 2
SWH 8012.820 Hz
FIDRES 0.244532 Hz
AQ 4.0894465 sec
RG 103.17
DW 62.400 usec
DE 30.00 usec
TE 298.0 K
D1 1.00000000 sec
TD0 1
SFO1 400.3024719 MHz
NUC1 1H
P1 12.00 usec
PLW1 4.67290020 W
F2 - Processing parameters
SI 65536
SF 400.3000080 MHz
WDW EM
SSB 0
LB 0.30 Hz
GB 0
PC 1.00
1.239
1.257
1.275
2.186
2.210
2.241
2.259
2.283
2.367
2.378
2.398
2.418
2.438
2.449
3.557
3.574
3.581
3.592
3.599
3.610
3.617
3.634
3.749
3.767
3.774
3.784
3.791
3.802
3.809
3.827
3.861
3.881
3.901
3.921
4.039
4.070
4.094
4.124
4.983
4.993
3.24
1.07
1.08
1.05
1.02
1.08
2.22
1.00
ppm
1
2
3
4
5
6
7
8
9
4.983
4.993
5.00
ppm
1.00

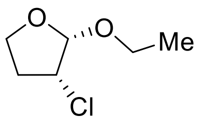

cis-**19a**

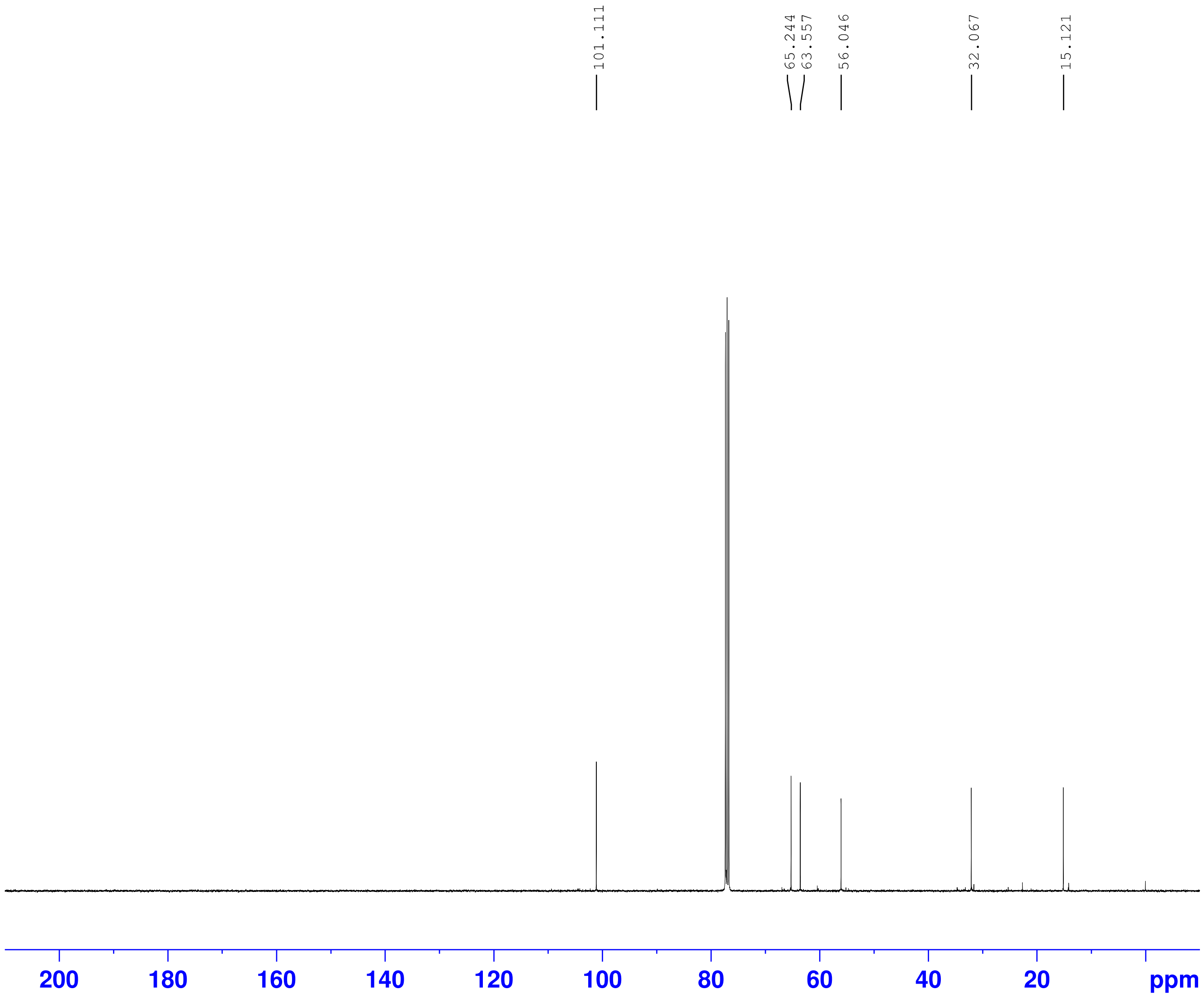


```
Current Data Parameters
NAME       kmd-5-046 Fr17-20
EXPNO                      3
PROCNO                     1

F2 - Acquisition Parameters
Date_               20201110
Time                   12.01 h
INSTRUM                spect
PROBHD     Z150354_0001 (
PULPROG              zgpg30
TD                     65356
SOLVENT                CDCl3
NS                       256
DS                         4
SWH                24038.461 Hz
FIDRES              0.735616 Hz
AQ                 1.3594048 sec
RG                     39.47
DW                    20.800 usec
DE                     25.00 usec
TE                     298.0 K
D1                2.00000000 sec
D11               0.03000000 sec
TD0                        1
SFO1             100.6655806 MHz
NUC1                     13C
P1                     10.00 usec
PLW1             19.75099945 W
SFO2             400.3016012 MHz
NUC2                      1H
CPDPRG[2             waltz16
PCPD2                  80.00 usec
PLW2              4.67290020 W
PLW12             0.10514000 W
PLW13             0.05280100 W

F2 - Processing parameters
SI                     32768
SF               100.6555151 MHz
WDW                       EM
SSB        0
LB                      1.00 Hz
GB         0
PC                      1.40
```

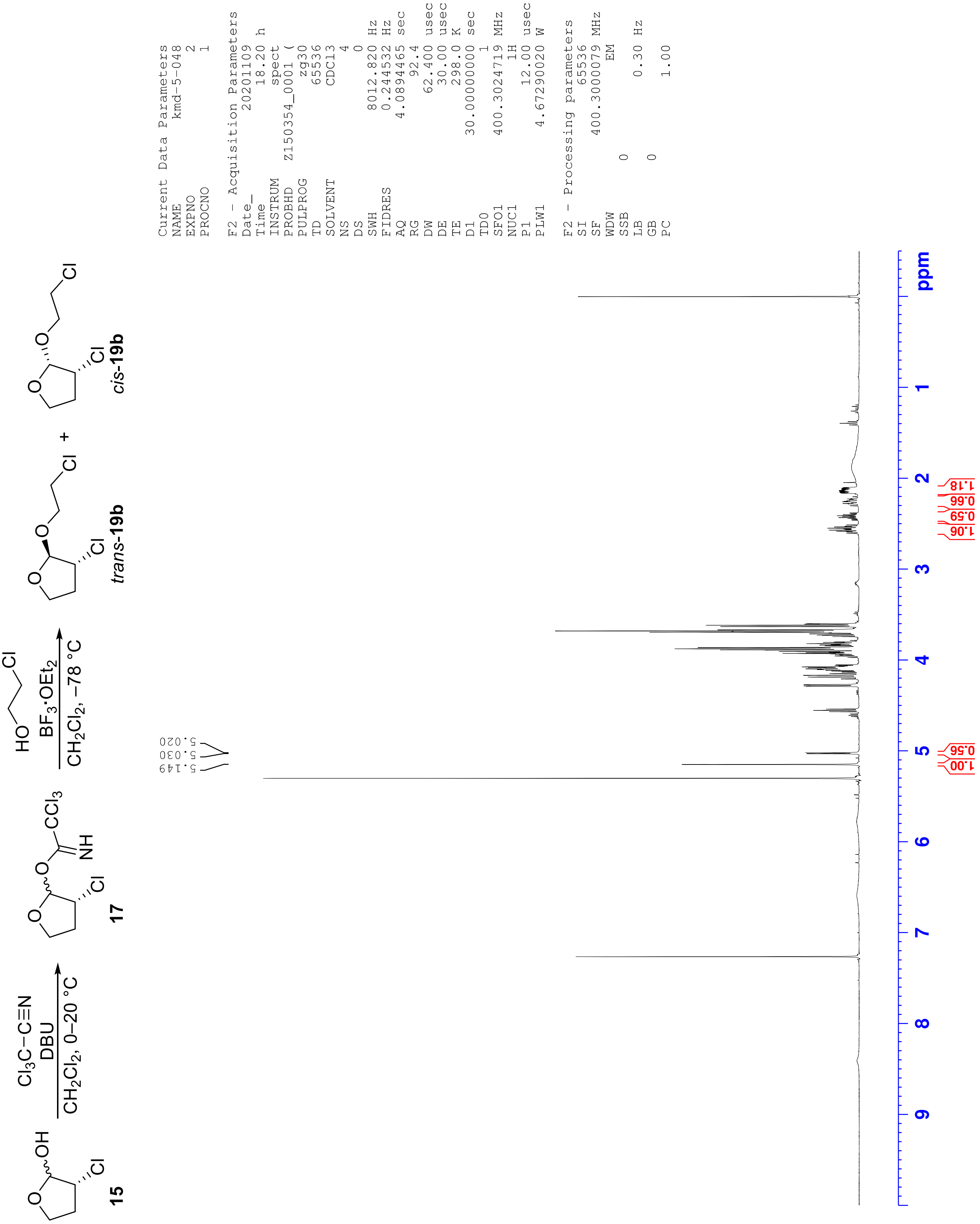
15
$Cl_3C-C{\equiv}N$
DBU
CH_2Cl_2, 0–20 °C
17
CCl_3
NH
HO
Cl
$BF_3{\cdot}OEt_2$
CH_2Cl_2, −78 °C
trans-19b
+
cis-19b
Current Data Parameters
NAME kmd-5-048
EXPNO 2
PROCNO 1
F2 - Acquisition Parameters
Date_ 20201109
Time 18.20 h
INSTRUM spect
PROBHD Z150354_0001 (
PULPROG zg30
TD 65536
SOLVENT CDCl3
NS 4
DS 0
SWH 8012.820 Hz
FIDRES 0.244532 Hz
AQ 4.0894465 sec
RG 92.4
DW 62.400 usec
DE 30.00 usec
TE 298.0 K
D1 30.00000000 sec
TD0 1
SFO1 400.3024719 MHz
NUC1 1H
P1 12.00 usec
PLW1 4.67290020 W
F2 - Processing parameters
SI 65536
SF 400.3000079 MHz
WDW EM
SSB 0
LB 0.30 Hz
GB 0
PC 1.00
5.020
5.030
5.149
1.06
0.59
0.66
1.18
0.56
1.00
ppm

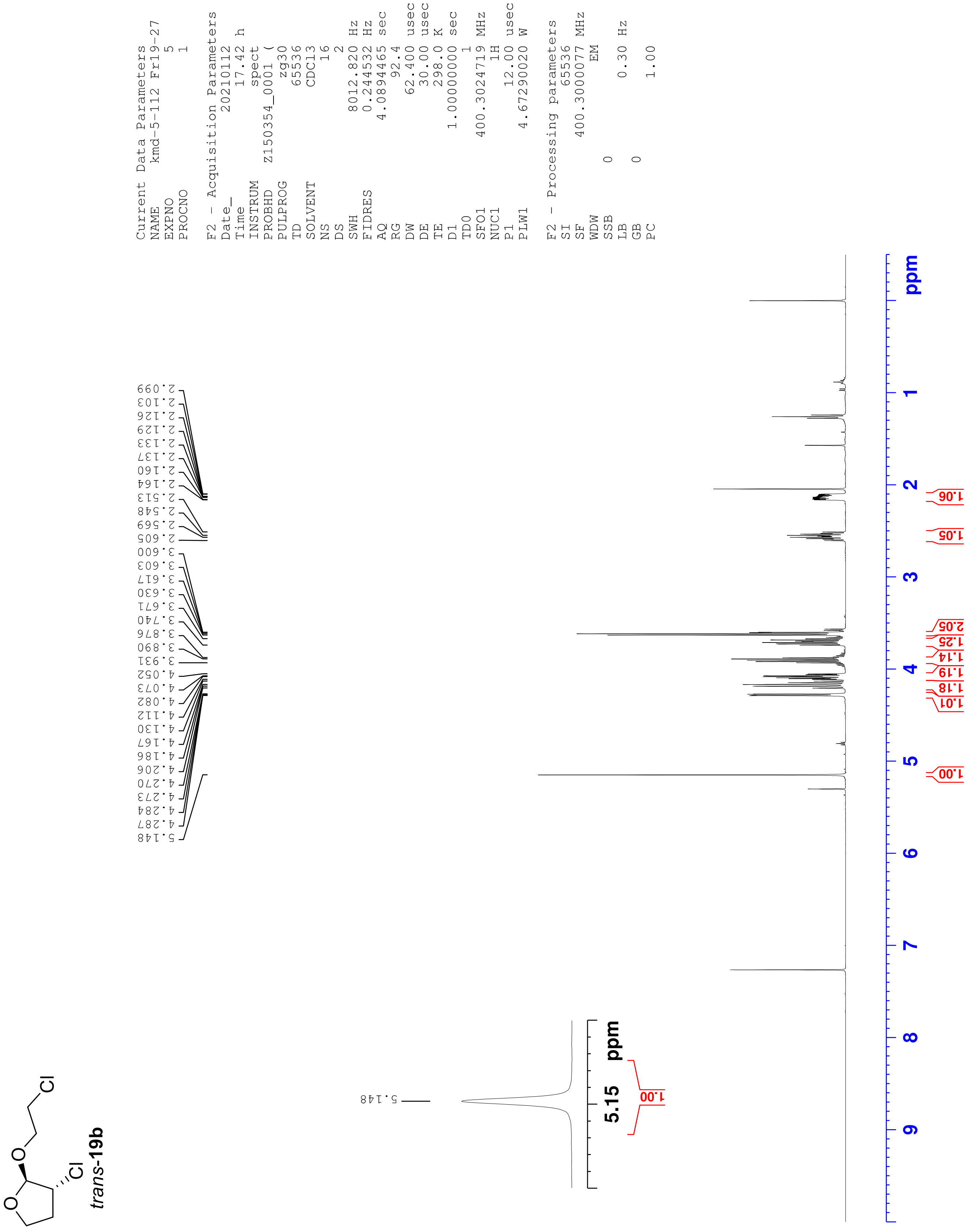
trans-19b
Current Data Parameters
NAME kmd-5-112 Fr19-27
EXPNO 5
PROCNO 1
F2 - Acquisition Parameters
Date_ 20210112
Time 17.42 h
INSTRUM spect
PROBHD Z150354_0001 (
PULPROG zg30
TD 65536
SOLVENT CDCl3
NS 16
DS 2
SWH 8012.820 Hz
FIDRES 0.244532 Hz
AQ 4.0894465 sec
RG 92.4
DW 62.400 usec
DE 30.00 usec
TE 298.0 K
D1 1.00000000 sec
TD0 1
SFO1 400.3024719 MHz
NUC1 1H
P1 12.00 usec
PLW1 4.67290020 W
F2 - Processing parameters
SI 65536
SF 400.3000077 MHz
WDW EM
SSB 0
LB 0.30 Hz
GB 0
PC 1.00
2.099 2.103 2.126 2.129 2.133 2.137 2.160 2.164 2.513 2.548 2.569 2.605 3.600 3.603 3.617 3.630 3.671 3.740 3.876 3.890 3.931 4.052 4.073 4.082 4.112 4.130 4.167 4.186 4.206 4.270 4.273 4.284 4.287 5.148
5.148
5.15 ppm
1.00
1.06 1.05 2.05 1.25 1.14 1.19 1.18 1.01 1.00
ppm

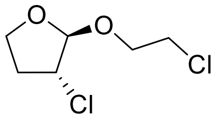

trans-**19b**

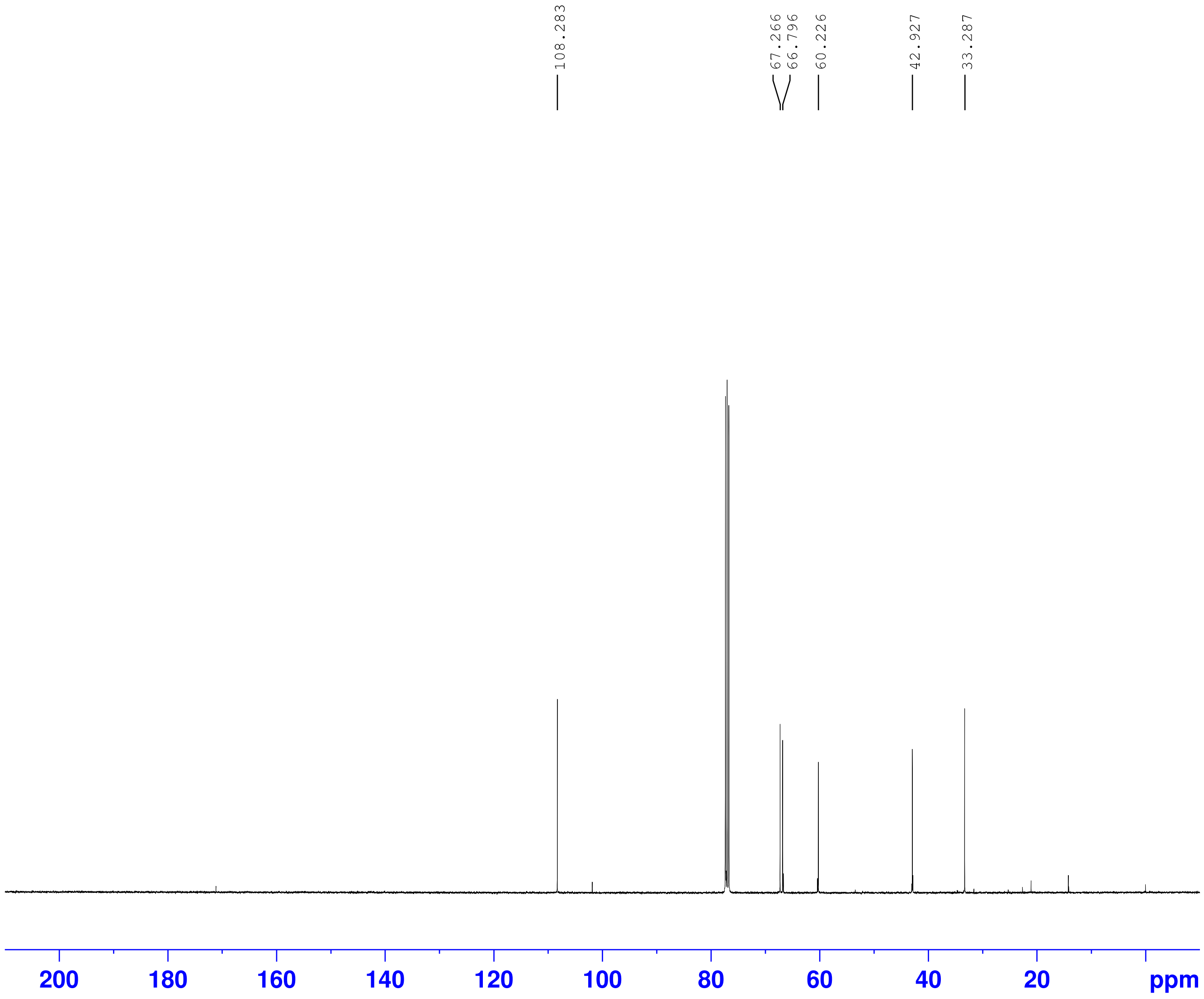


```
Current Data Parameters
NAME      kmd-5-112 Fr19-27
EXPNO                  4
PROCNO                 1

F2 - Acquisition Parameters
Date_           20210112
Time               17.09 h
INSTRUM            spect
PROBHD   Z150354_0001 (
PULPROG          zgpg30
TD                 65356
SOLVENT            CDCl3
NS                   256
DS                     4
SWH            24038.461 Hz
FIDRES          0.735616 Hz
AQ             1.3594048 sec
RG                  31.4
DW                20.800 usec
DE                 25.00 usec
TE                 298.0 K
D1            2.00000000 sec
D11           0.03000000 sec
TD0                    1
SFO1         100.6655806 MHz
NUC1                 13C
P1                 10.00 usec
PLW1         19.75099945 W
SFO2         400.3016012 MHz
NUC2                  1H
CPDPRG[2         waltz16
PCPD2              80.00 usec
PLW2          4.67290020 W
PLW12         0.10514000 W
PLW13         0.05280100 W

F2 - Processing parameters
SI                 32768
SF           100.6555158 MHz
WDW                   EM
SSB       0
LB                  1.00 Hz
GB        0
PC                  1.40
```

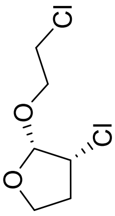

cis-**19b**

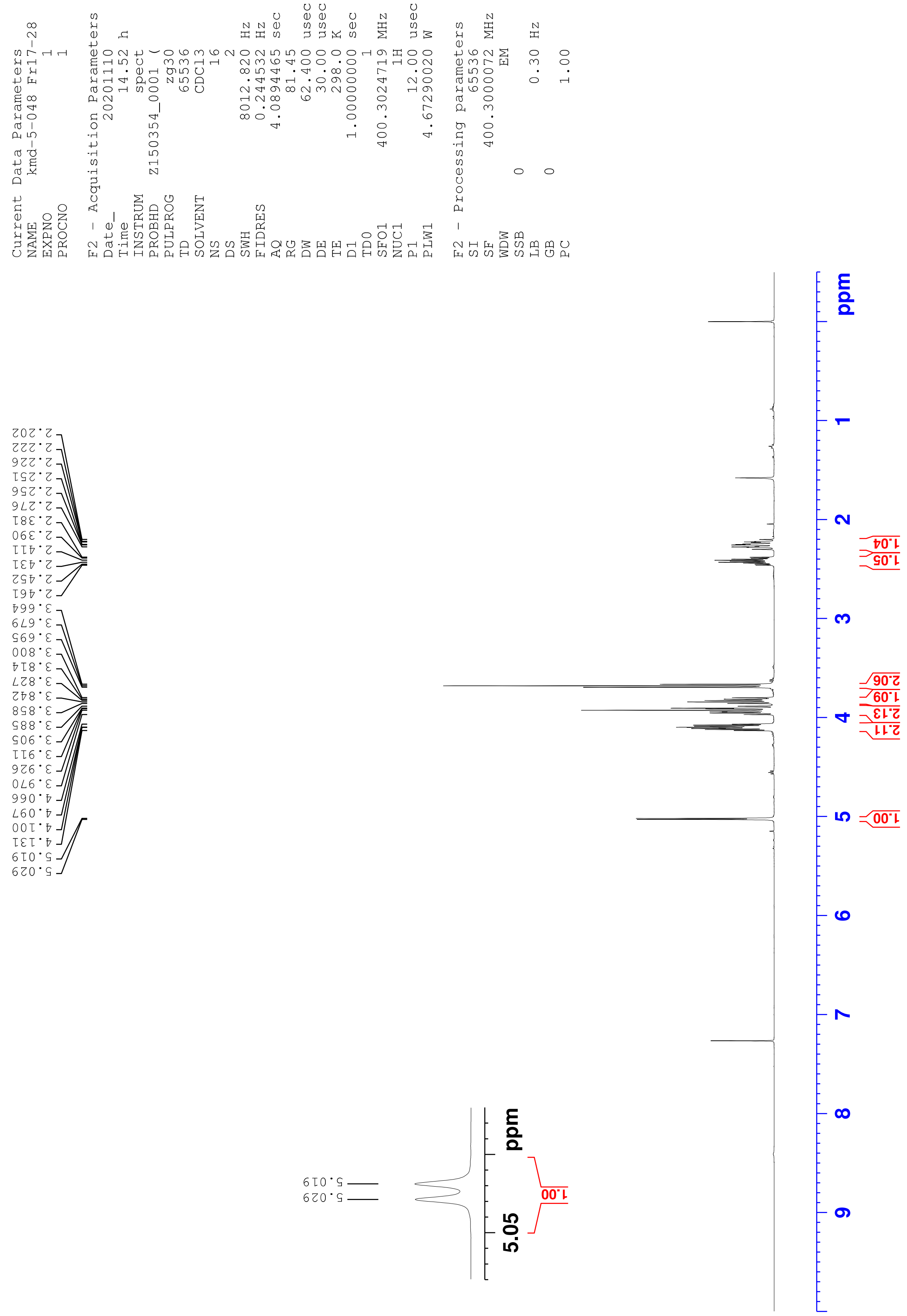
5.029
5.019
4.131
4.100
4.097
4.066
3.970
3.926
3.911
3.905
3.885
3.858
3.842
3.827
3.814
3.800
3.695
3.679
3.664
2.461
2.452
2.431
2.411
2.390
2.381
2.276
2.256
2.251
2.226
2.222
2.202
5.05
ppm
1.00
9
8
7
6
5
4
3
2
1
ppm
1.00
2.11
2.13
1.09
2.06
1.05
1.04
Current Data Parameters
NAME kmd-5-048 Fr17-28
EXPNO 1
PROCNO 1
F2 - Acquisition Parameters
Date_ 20201110
Time 14.52 h
INSTRUM spect
PROBHD Z150354_0001 (
PULPROG zg30
TD 65536
SOLVENT CDCl3
NS 16
DS 2
SWH 8012.820 Hz
FIDRES 0.244532 Hz
AQ 4.0894465 sec
RG 81.45
DW 62.400 usec
DE 30.00 usec
TE 298.0 K
D1 1.00000000 sec
TD0 1
SFO1 400.3024719 MHz
NUC1 1H
P1 12.00 usec
PLW1 4.67290020 W
F2 - Processing parameters
SI 65536
SF 400.3000072 MHz
WDW EM
SSB 0
LB 0.30 Hz
GB 0
PC 1.00

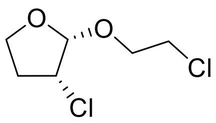

cis-**19b**

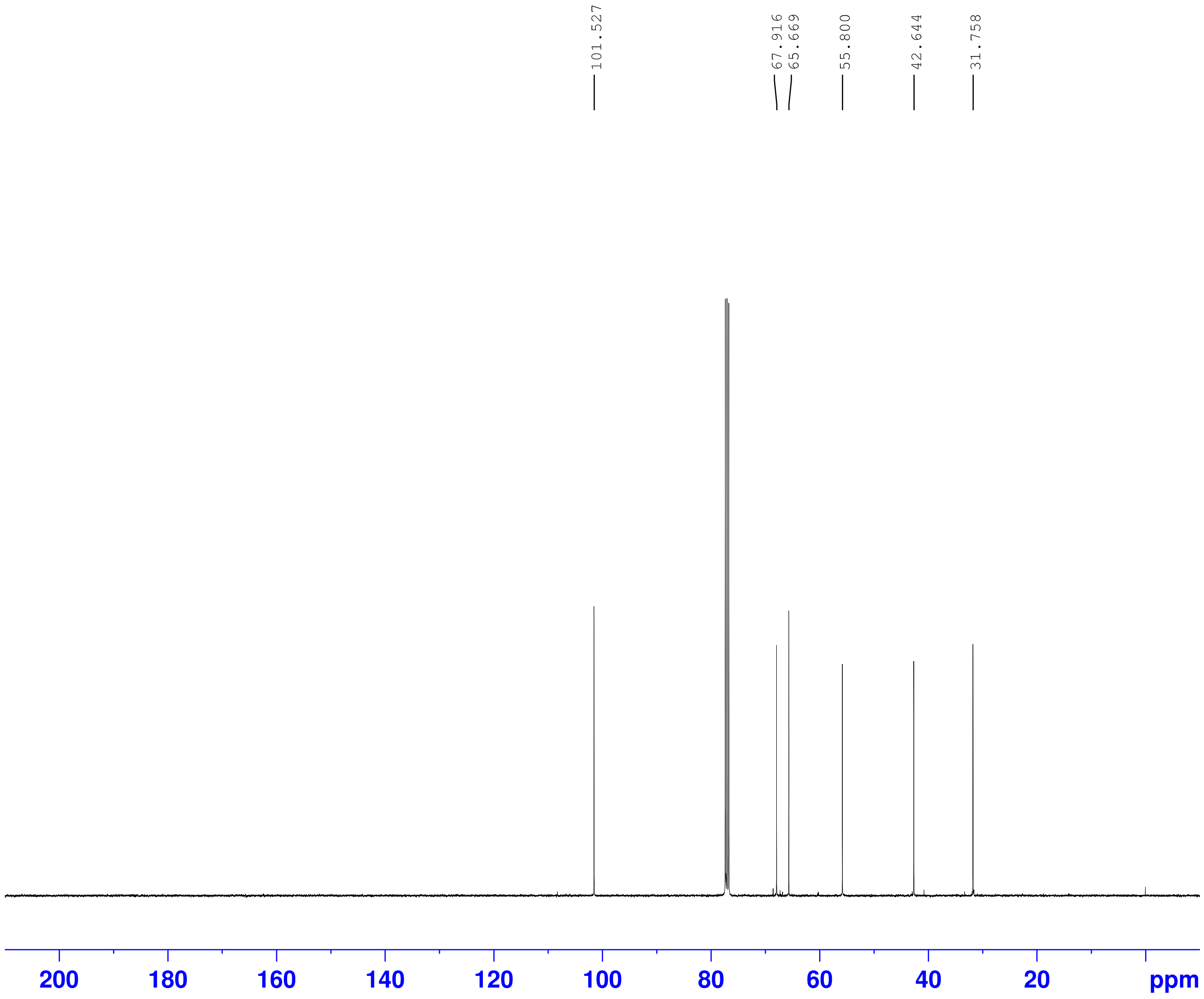


```
Current Data Parameters
NAME      kmd-5-048 Fr17-28
EXPNO                     3
PROCNO                    1

F2 - Acquisition Parameters
Date_              20201110
Time                  20.27 h
INSTRUM               spect
PROBHD    Z150354_0001 (
PULPROG             zgpg30
TD                   65356
SOLVENT              CDCl3
NS                     256
DS                       4
SWH              24038.461 Hz
FIDRES            0.735616 Hz
AQ               1.3594048 sec
RG                   39.47
DW                  20.800 usec
DE                   25.00 usec
TE                   298.0 K
D1              2.00000000 sec
D11             0.03000000 sec
TD0                      1
SFO1           100.6655806 MHz
NUC1                   13C
P1                   10.00 usec
PLW1           19.75099945 W
SFO2           400.3016012 MHz
NUC2                    1H
CPDPRG[2          waltz16
PCPD2                80.00 usec
PLW2            4.67290020 W
PLW12           0.10514000 W
PLW13           0.05280100 W

F2 - Processing parameters
SI                   32768
SF             100.6555151 MHz
WDW                     EM
SSB       0
LB                    1.00 Hz
GB        0
PC                    1.40
```

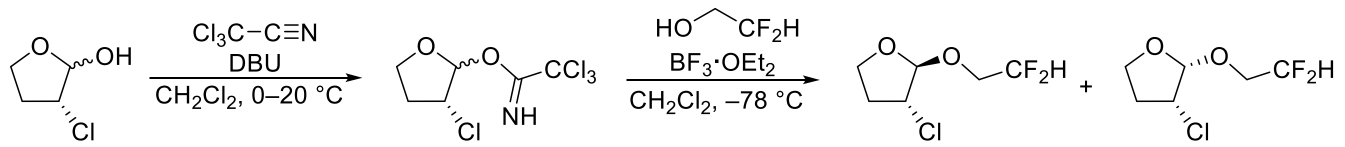

15 **17** *trans*-**19c** *cis*-**19c**

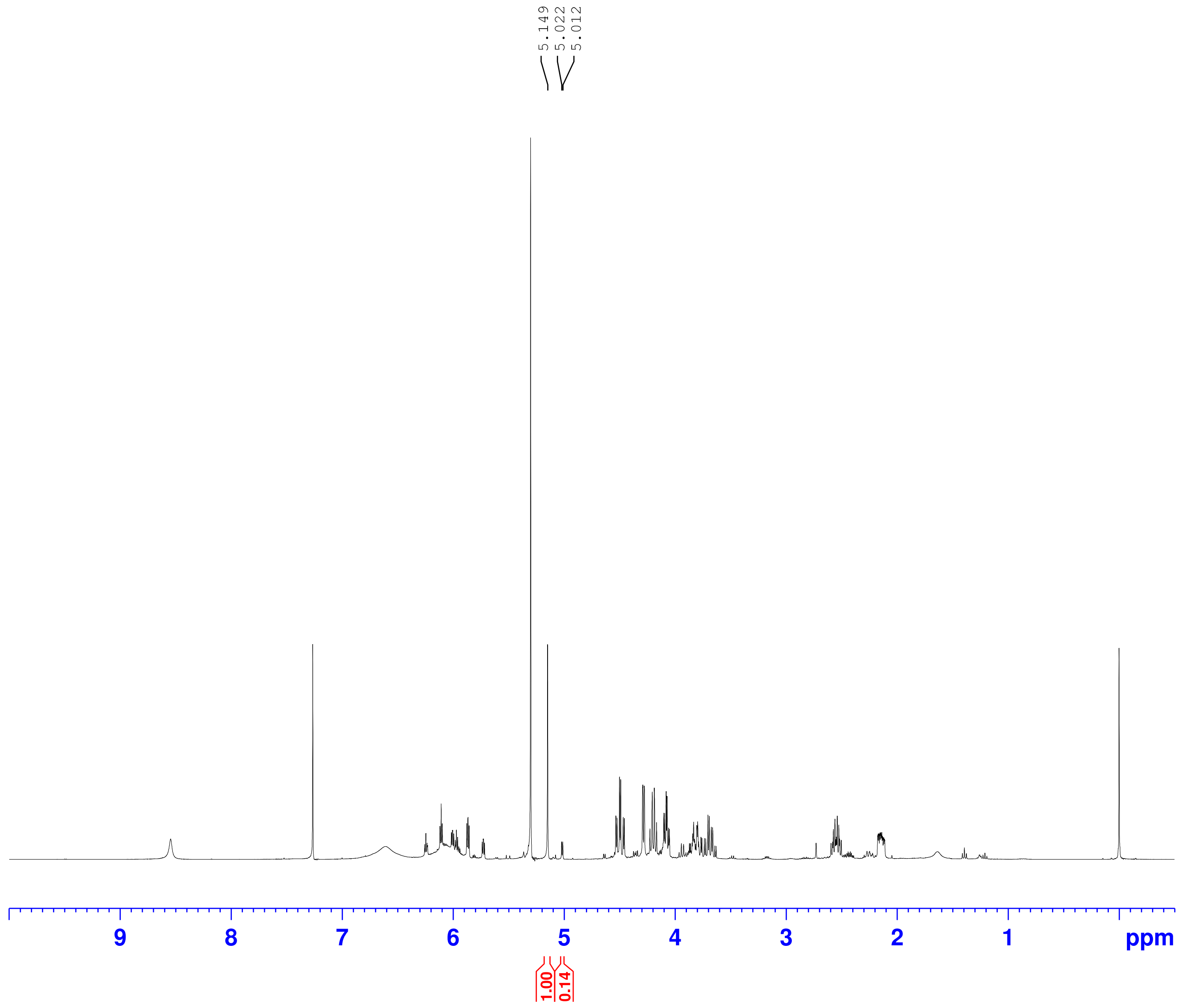


```
Current Data Parameters
NAME             kmd-6-103
EXPNO                    2
PROCNO                   1

F2 - Acquisition Parameters
Date_             20220114
Time                 16.24 h
INSTRUM              spect
PROBHD    Z150354_0001 (
PULPROG               zg30
TD                   65536
SOLVENT              CDCl3
NS                       4
DS                       0
SWH               8012.820 Hz
FIDRES            0.244532 Hz
AQ               4.0894465 sec
RG                  141.61
DW                  62.400 usec
DE                   30.00 usec
TE                   298.0 K
D1             30.00000000 sec
TD0                      1
SFO1           400.3024719 MHz
NUC1                    1H
P1                   12.00 usec
PLW1            4.64209986 W

F2 - Processing parameters
SI                   65536
SF             400.3000080 MHz
WDW                     EM
SSB      0
LB                    0.30 Hz
GB       0
PC                    1.00
```

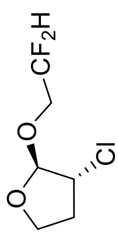

trans-**19c**

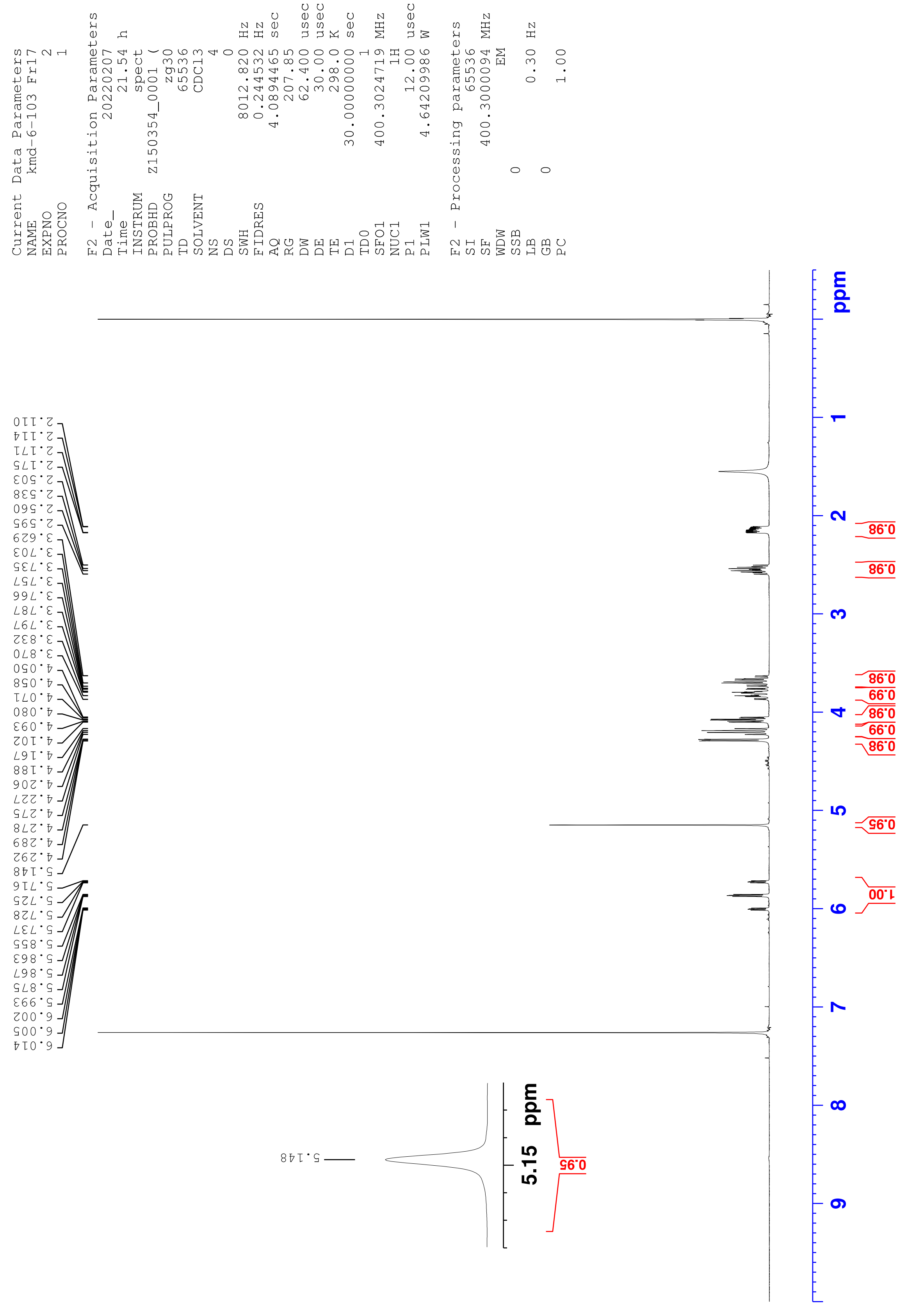
Current Data Parameters
NAME kmd-6-103 Fr17
EXPNO 2
PROCNO 1
F2 - Acquisition Parameters
Date_ 20220207
Time 21.54 h
INSTRUM spect
PROBHD Z150354_0001 (
PULPROG zg30
TD 65536
SOLVENT CDCl3
NS 4
DS 0
SWH 8012.820 Hz
FIDRES 0.244532 Hz
AQ 4.0894465 sec
RG 207.85
DW 62.400 usec
DE 30.00 usec
TE 298.0 K
D1 30.00000000 sec
TD0 1
SFO1 400.3024719 MHz
NUC1 1H
P1 12.00 usec
PLW1 4.64209986 W
F2 - Processing parameters
SI 65536
SF 400.3000094 MHz
WDW EM
SSB 0
LB 0.30 Hz
GB 0
PC 1.00
ppm

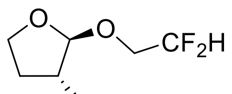

trans-**19c**

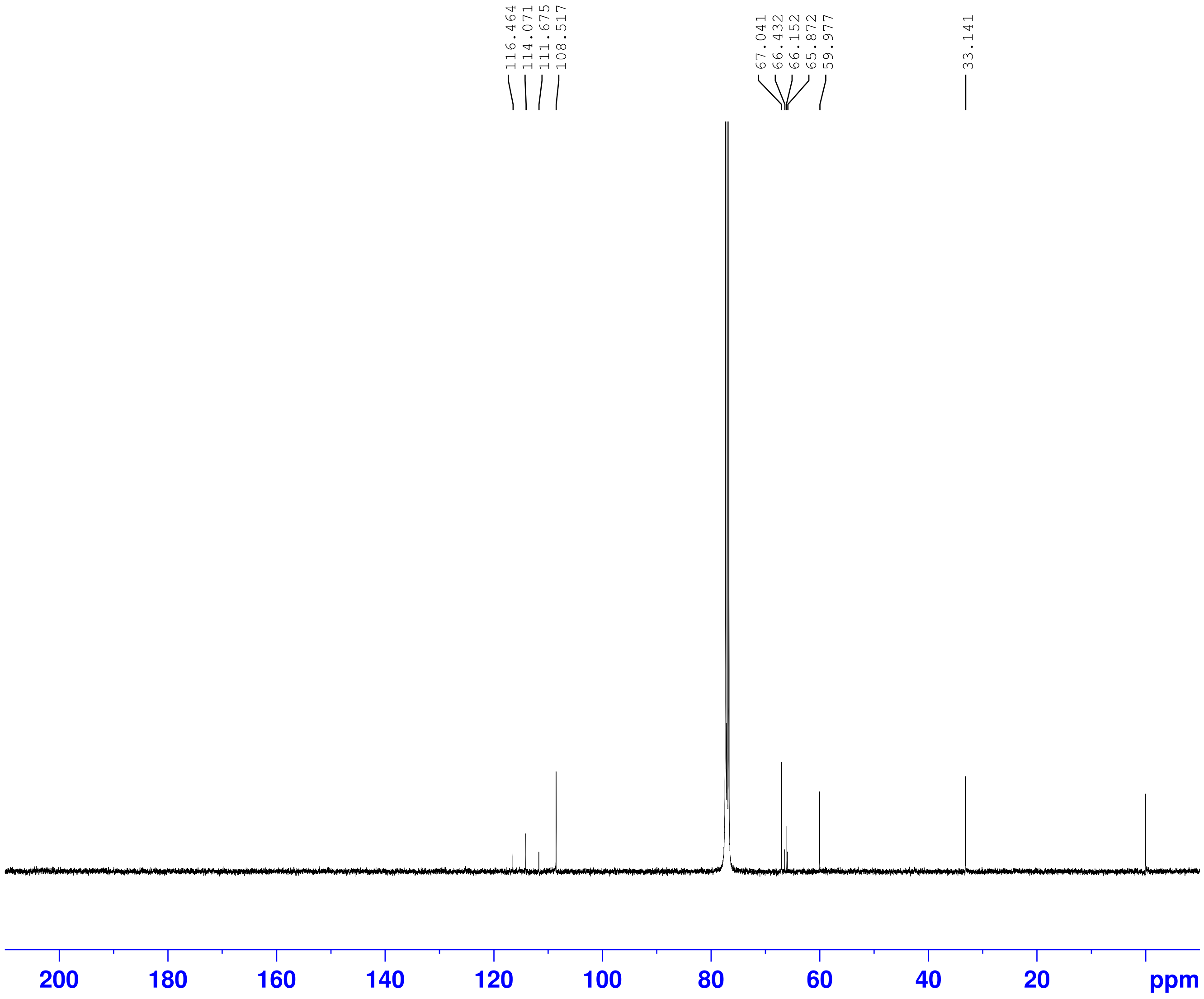


```
Current Data Parameters
NAME      kmd-6-103 Fr17 carbon
EXPNO                     1
PROCNO                    1

F2 - Acquisition Parameters
Date_              20220209
Time                   6.39 h
INSTRUM               spect
PROBHD    Z150354_0001 (
PULPROG             zgpg30
TD                   65356
SOLVENT              CDCl3
NS                    1024
DS                       4
SWH              24038.461 Hz
FIDRES            0.735616 Hz
AQ               1.3594048 sec
RG                   29.21
DW                  20.800 usec
DE                   25.00 usec
TE                   298.0 K
D1              2.00000000 sec
D11             0.03000000 sec
TD0                      1
SFO1           100.6655806 MHz
NUC1                   13C
P1                   10.00 usec
PLW1           17.99699974 W
SFO2           400.3016012 MHz
NUC2                    1H
CPDPRG[2           waltz16
PCPD2                80.00 usec
PLW2            4.64209986 W
PLW12           0.10445000 W
PLW13           0.05245300 W

F2 - Processing parameters
SI                   32768
SF             100.6555156 MHz
WDW                     EM
SSB       0
LB                    1.00 Hz
GB        0
PC                    1.40
```

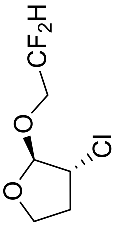

trans-19c

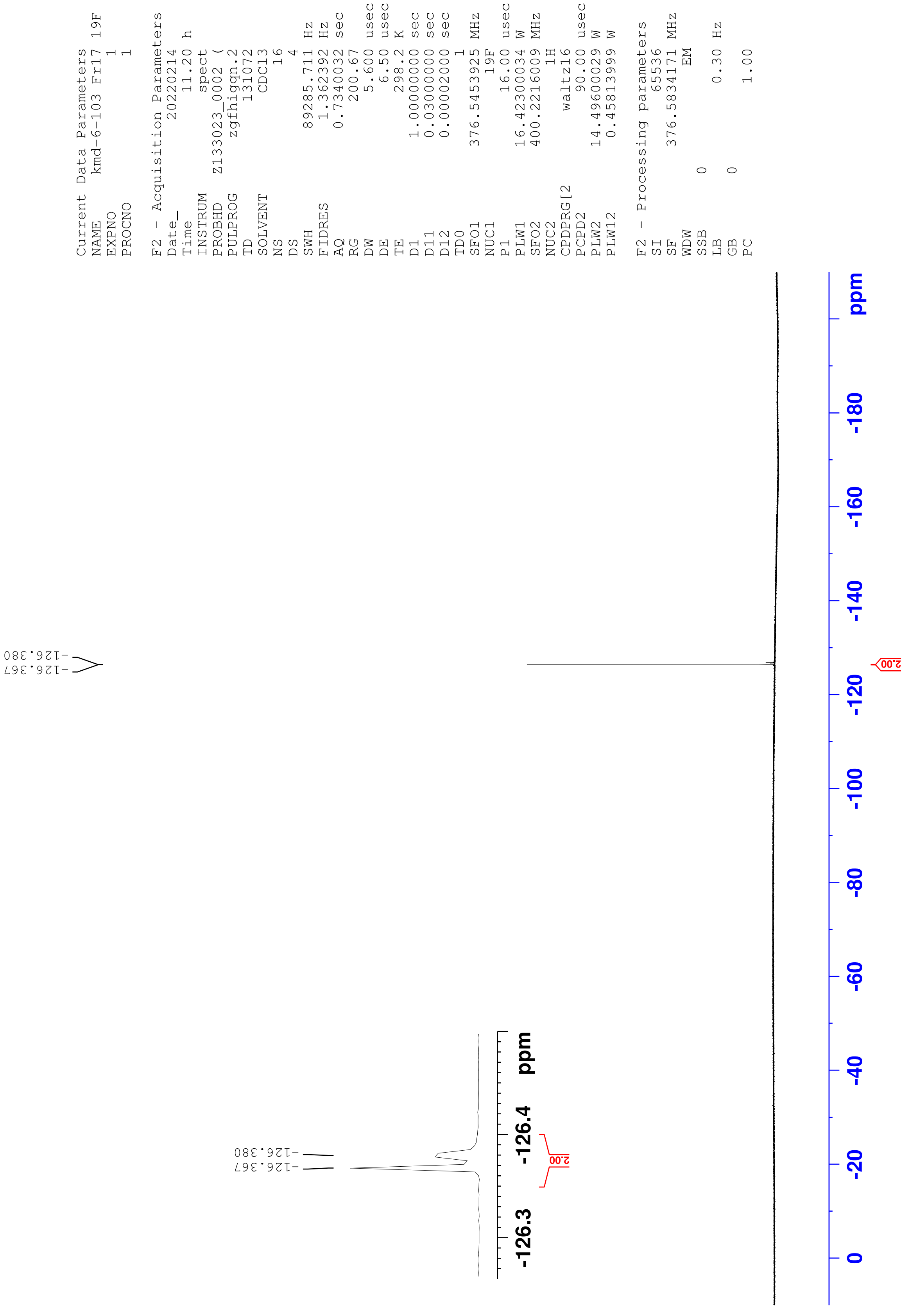
Current Data Parameters
NAME kmd-6-103 Fr17 19F
EXPNO 1
PROCNO 1
F2 - Acquisition Parameters
Date_ 20220214
Time 11.20 h
INSTRUM spect
PROBHD Z133023_0002 (
PULPROG zgfhigqn.2
TD 131072
SOLVENT CDCl3
NS 16
DS 4
SWH 89285.711 Hz
FIDRES 1.362392 Hz
AQ 0.7340032 sec
RG 200.67
DW 5.600 usec
DE 6.50 usec
TE 298.2 K
D1 1.00000000 sec
D11 0.03000000 sec
D12 0.00002000 sec
TD0 1
SFO1 376.5453925 MHz
NUC1 19F
P1 16.00 usec
PLW1 16.42300034 W
SFO2 400.2216009 MHz
NUC2 1H
CPDPRG[2 waltz16
PCPD2 90.00 usec
PLW2 14.49600029 W
PLW12 0.45813999 W
F2 - Processing parameters
SI 65536
SF 376.5834171 MHz
WDW EM
SSB 0
LB 0.30 Hz
GB 0
PC 1.00
-126.367
-126.380
2.00
-126.3
-126.4
ppm
0
-20
-40
-60
-80
-100
-120
-140
-160
-180
ppm

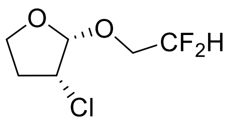

cis-**19c**

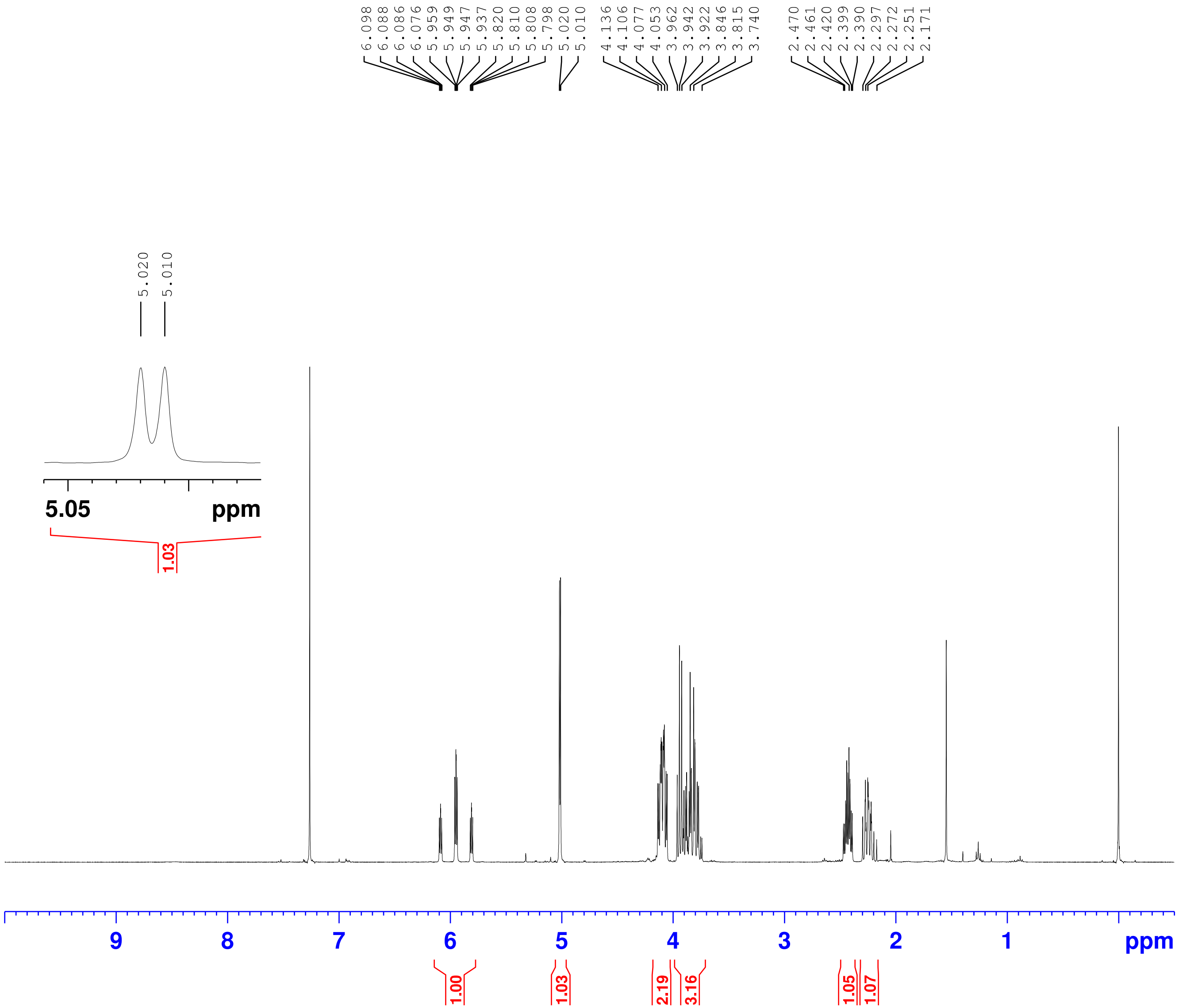

Current Data Parameters
NAME kmd-6-006 Fr24-28
EXPNO 2
PROCNO 1

F2 - Acquisition Parameters
Date_ 20210525
Time 11.26 h
INSTRUM spect
PROBHD Z150354_0001 (
PULPROG zg30
TD 65536
SOLVENT CDCl3
NS 4
DS 0
SWH 8012.820 Hz
FIDRES 0.244532 Hz
AQ 4.0894465 sec
RG 92.4
DW 62.400 usec
DE 30.00 usec
TE 298.0 K
D1 30.00000000 sec
TD0 1
SFO1 400.3024719 MHz
NUC1 1H
P1 12.00 usec
PLW1 4.67290020 W

F2 - Processing parameters
SI 65536
SF 400.3000088 MHz
WDW EM
SSB 0
LB 0.30 Hz
GB 0
PC 1.00

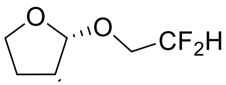

cis-**19c**

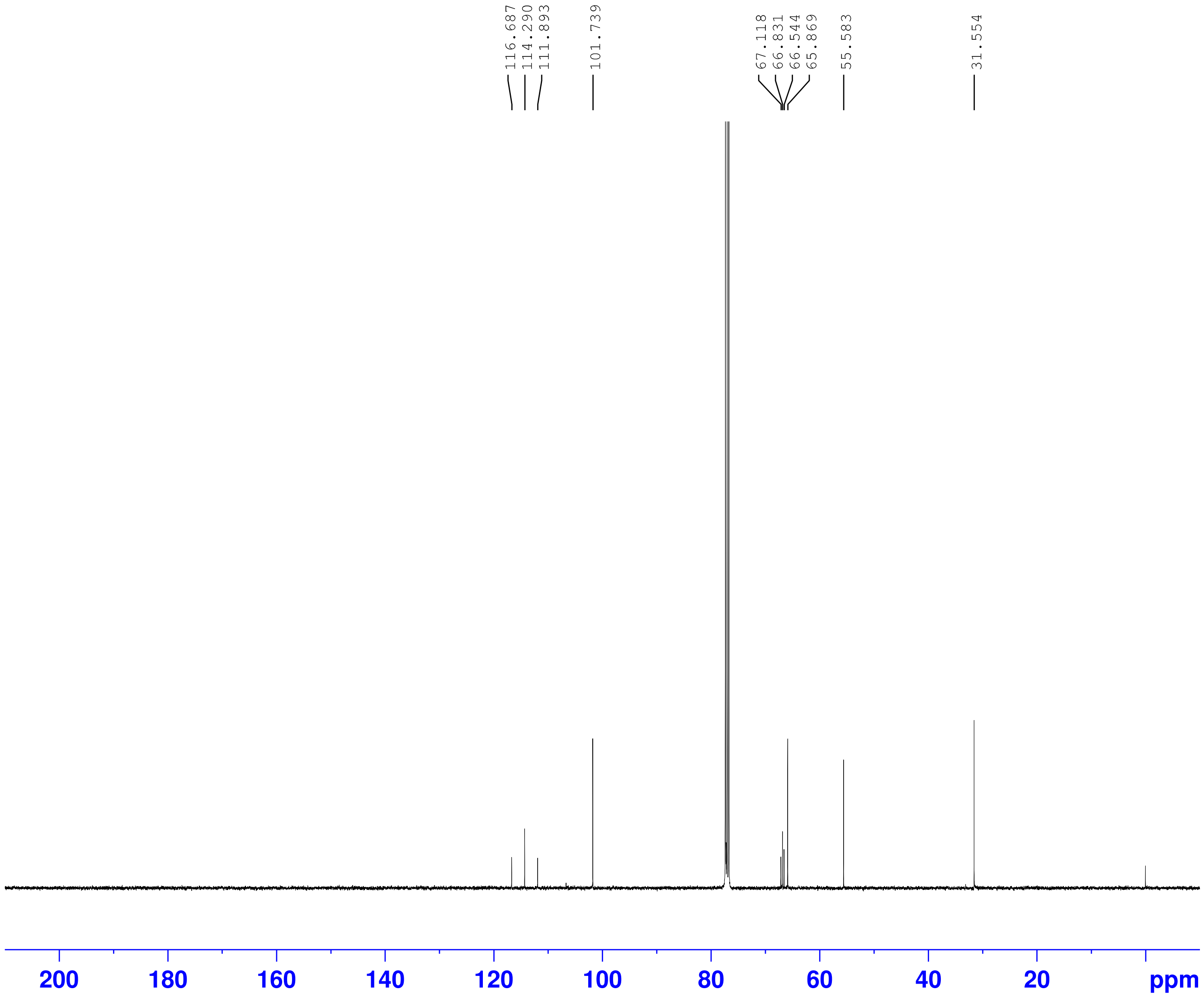

Current Data Parameters

NAME	kmd-6-006 Fr24-28
EXPNO	3
PROCNO	1

F2 - Acquisition Parameters

Date_	20210525
Time	13.22 h
INSTRUM	spect
PROBHD	Z150354_0001 (
PULPROG	zgpg30
TD	65356
SOLVENT	CDCl3
NS	256
DS	4
SWH	24038.461 Hz
FIDRES	0.735616 Hz
AQ	1.3594048 sec
RG	58.09
DW	20.800 usec
DE	25.00 usec
TE	298.0 K
D1	2.00000000 sec
D11	0.03000000 sec
TD0	1
SFO1	100.6655806 MHz
NUC1	13C
P1	10.00 usec
PLW1	19.75099945 W
SFO2	400.3016012 MHz
NUC2	1H
CPDPRG[2	waltz16
PCPD2	80.00 usec
PLW2	4.67290020 W
PLW12	0.10514000 W
PLW13	0.05280100 W

F2 - Processing parameters

SI	32768
SF	100.6555157 MHz
WDW	EM
SSB	0
LB	1.00 Hz
GB	0
PC	1.40

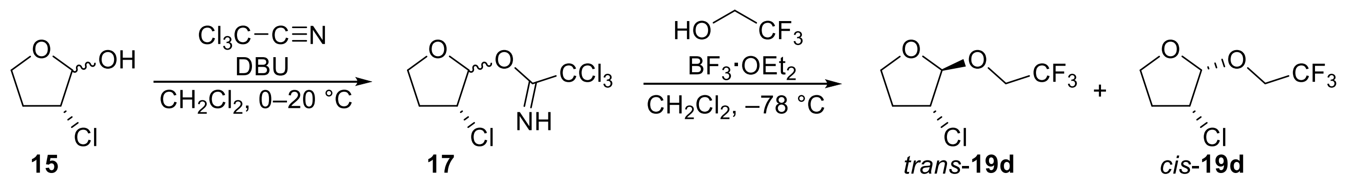

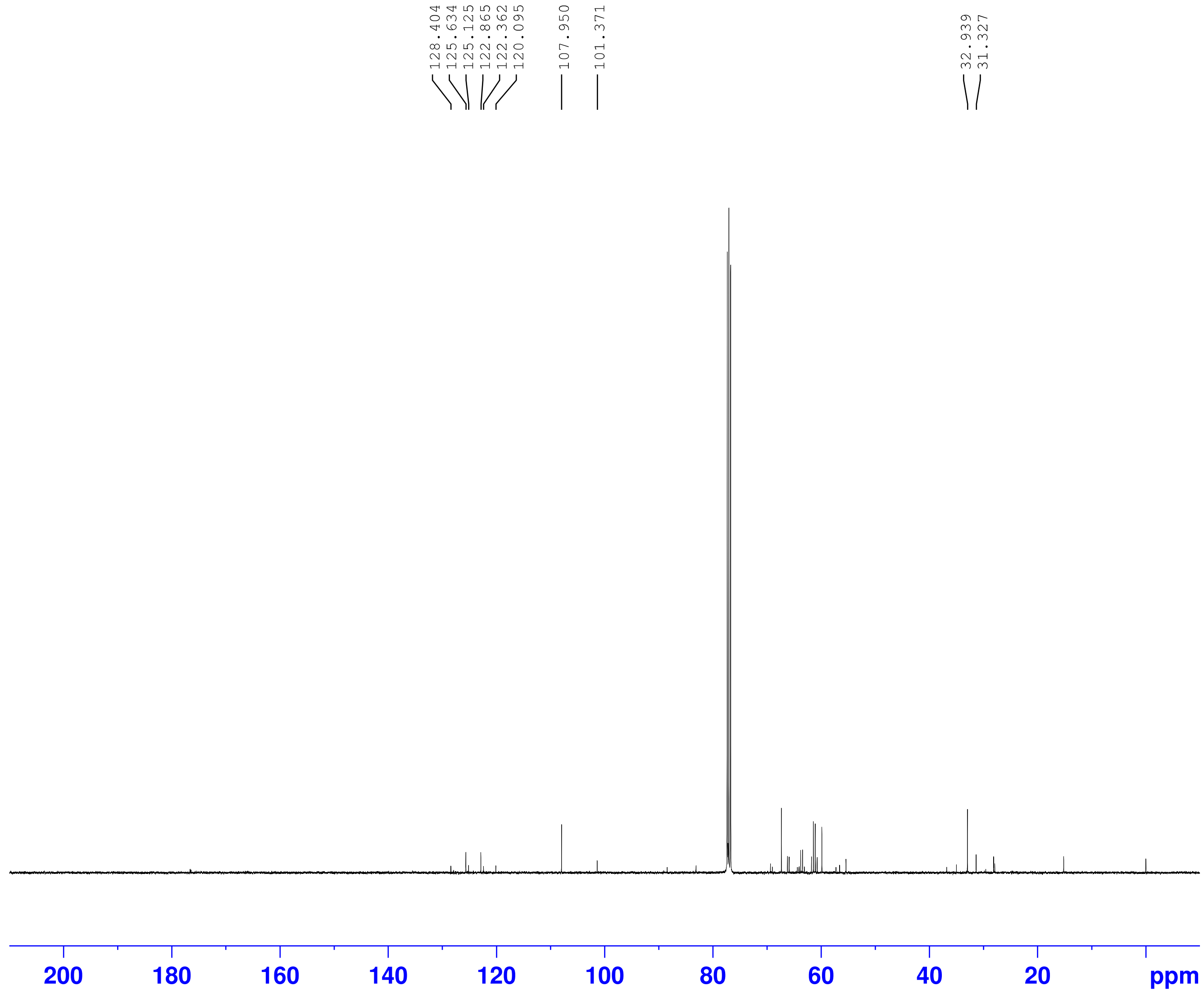


```
Current Data Parameters
NAME              kmd-5-111
EXPNO                     3
PROCNO                    1

F2 - Acquisition Parameters
Date_              20210111
Time                  12.08 h
INSTRUM               spect
PROBHD    Z150354_0001 (
PULPROG             zgpg30
TD                   65356
SOLVENT              CDCl3
NS                     256
DS                       4
SWH              24038.461 Hz
FIDRES            0.735616 Hz
AQ               1.3594048 sec
RG                   29.21
DW                  20.800 usec
DE                   25.00 usec
TE                   298.0 K
D1              2.00000000 sec
D11             0.03000000 sec
TD0                      1
SFO1           100.6655806 MHz
NUC1                   13C
P1                   10.00 usec
PLW1           19.75099945 W
SFO2           400.3016012 MHz
NUC2                    1H
CPDPRG[2           waltz16
PCPD2                80.00 usec
PLW2            4.67290020 W
PLW12           0.10514000 W
PLW13           0.05280100 W

F2 - Processing parameters
SI                   32768
SF             100.6555151 MHz
WDW                     EM
SSB      0
LB                    1.00 Hz
GB       0
PC                    1.40
```

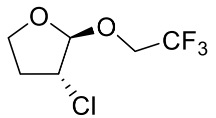

trans-**19d**

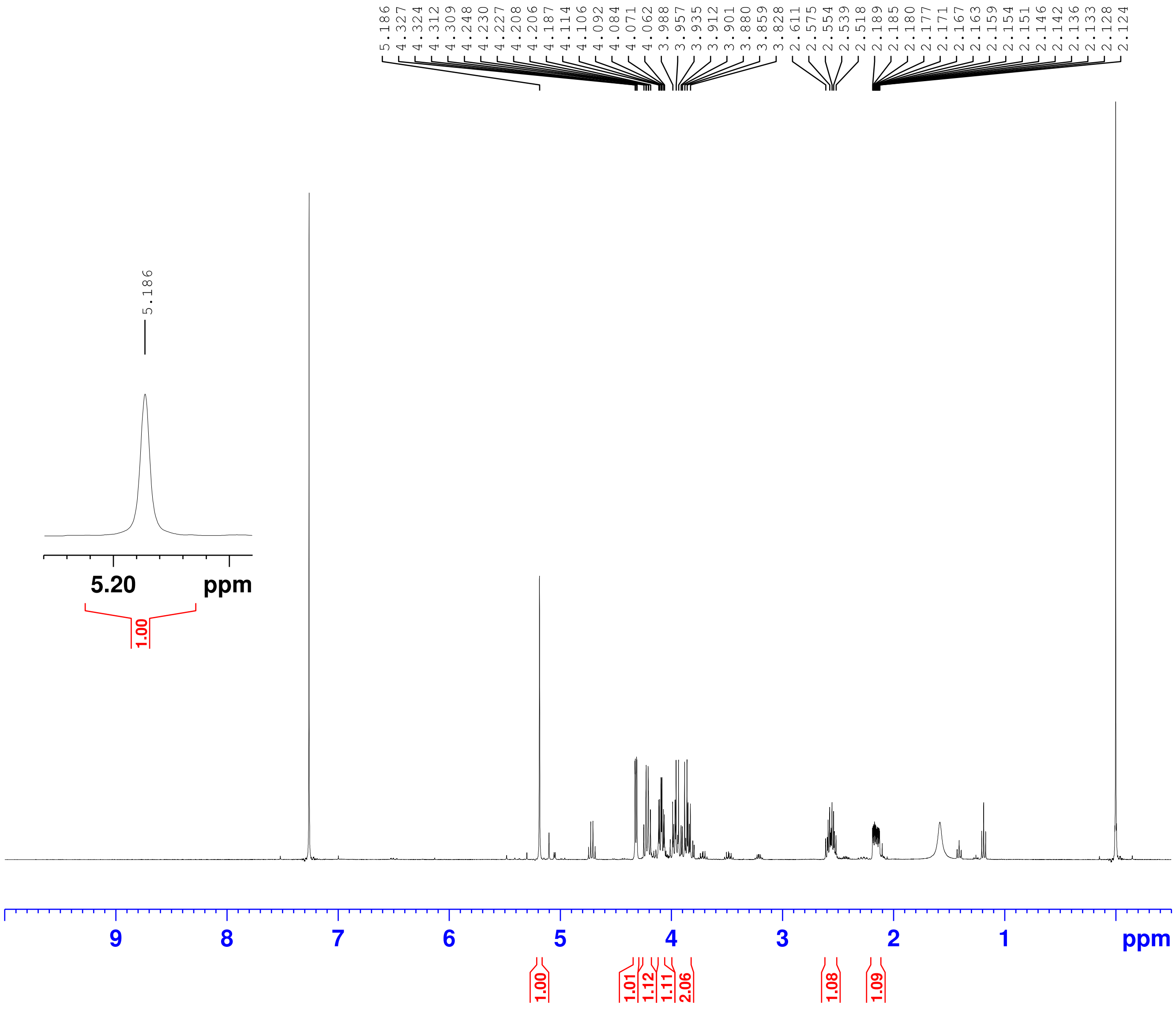


```
Current Data Parameters
NAME       kmd-7-045 d2 60
EXPNO                    2
PROCNO                   1

F2 - Acquisition Parameters
Date_             20220526
Time                 13.48 h
INSTRUM              spect
PROBHD     Z150354_0001 (
PULPROG               zg30
TD                   65536
SOLVENT              CDCl3
NS                       4
DS                       0
SWH               8012.820 Hz
FIDRES            0.244532 Hz
AQ               4.0894465 sec
RG                  184.17
DW                  62.400 usec
DE                   30.00 usec
TE                   298.0 K
D1             30.00000000 sec
TD0                      1
SFO1           400.3024719 MHz
NUC1                    1H
P1                   12.00 usec
PLW1            4.64209986 W

F2 - Processing parameters
SI                   65536
SF             400.3000091 MHz
WDW                     EM
SSB       0
LB                    0.30 Hz
GB        0
PC                    1.00
```

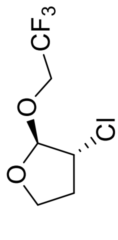
O
O
CF3
Cl

trans-**19d**

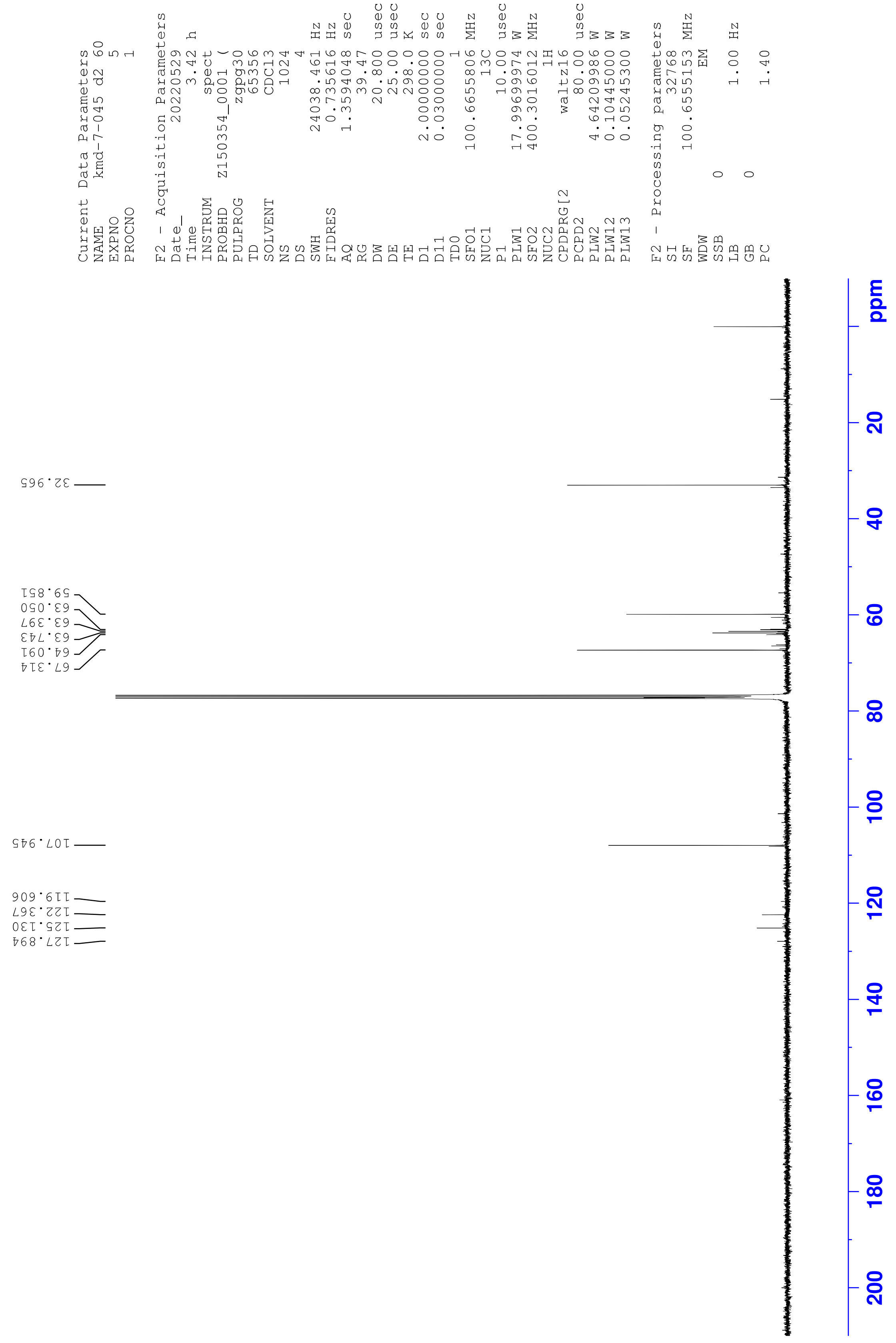
Current Data Parameters
NAME kmd-7-045 d2 60
EXPNO 5
PROCNO 1
F2 - Acquisition Parameters
Date_ 20220529
Time 3.42 h
INSTRUM spect
PROBHD Z150354_0001 (
PULPROG zgpg30
TD 65356
SOLVENT CDCl3
NS 1024
DS 4
SWH 24038.461 Hz
FIDRES 0.735616 Hz
AQ 1.3594048 sec
RG 39.47
DW 20.800 usec
DE 25.00 usec
TE 298.0 K
D1 2.00000000 sec
D11 0.03000000 sec
TD0 1
SFO1 100.6655806 MHz
NUC1 13C
P1 10.00 usec
PLW1 17.99699974 W
SFO2 400.3016012 MHz
NUC2 1H
CPDPRG[2 waltz16
PCPD2 80.00 usec
PLW2 4.64209986 W
PLW12 0.10445000 W
PLW13 0.05245300 W
F2 - Processing parameters
SI 32768
SF 100.6555153 MHz
WDW EM
SSB 0
LB 1.00 Hz
GB 0
PC 1.40
32.965
59.851
63.050
63.397
63.743
64.091
67.314
107.945
119.606
122.367
125.130
127.894
ppm
20
40
60
80
100
120
140
160
180
200

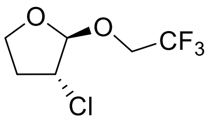

trans-**19d**

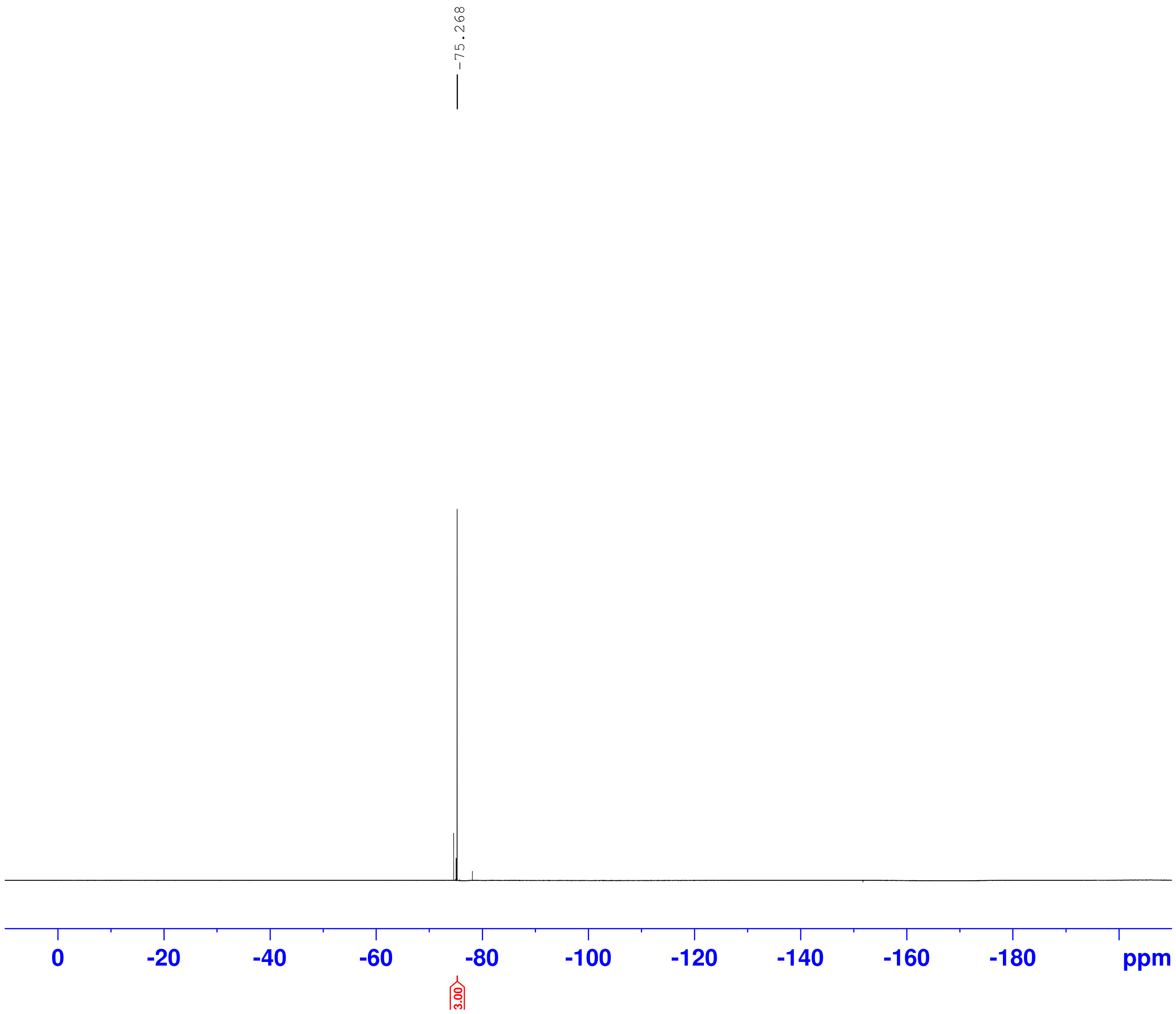


```
Current Data Parameters
NAME       kmd-7-045 d2 60 19F
EXPNO                    1
PROCNO                   1

F2 - Acquisition Parameters
Date_             20220527
Time                 12.03 h
INSTRUM              spect
PROBHD     Z133023_0002 (
PULPROG         zgfhigqn.2
TD                  131072
SOLVENT              CDCl3
NS                      16
DS                       4
SWH             89285.711 Hz
FIDRES           1.362392 Hz
AQ              0.7340032 sec
RG                  200.67
DW                   5.600 usec
DE                    6.50 usec
TE                   298.2 K
D1             1.00000000 sec
D11            0.03000000 sec
D12            0.00002000 sec
TD0                      1
SFO1          376.5453925 MHz
NUC1                   19F
P1                   16.00 usec
PLW1          16.42300034 W
SFO2          400.2216009 MHz
NUC2                    1H
CPDPRG[2           waltz16
PCPD2                90.00 usec
PLW2          14.49600029 W
PLW12          0.45813999 W

F2 - Processing parameters
SI                   65536
SF            376.5834164 MHz
WDW                     EM
SSB       0
LB                    0.30 Hz
GB        0
PC                    1.00
```

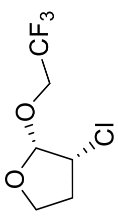

cis-**19d**

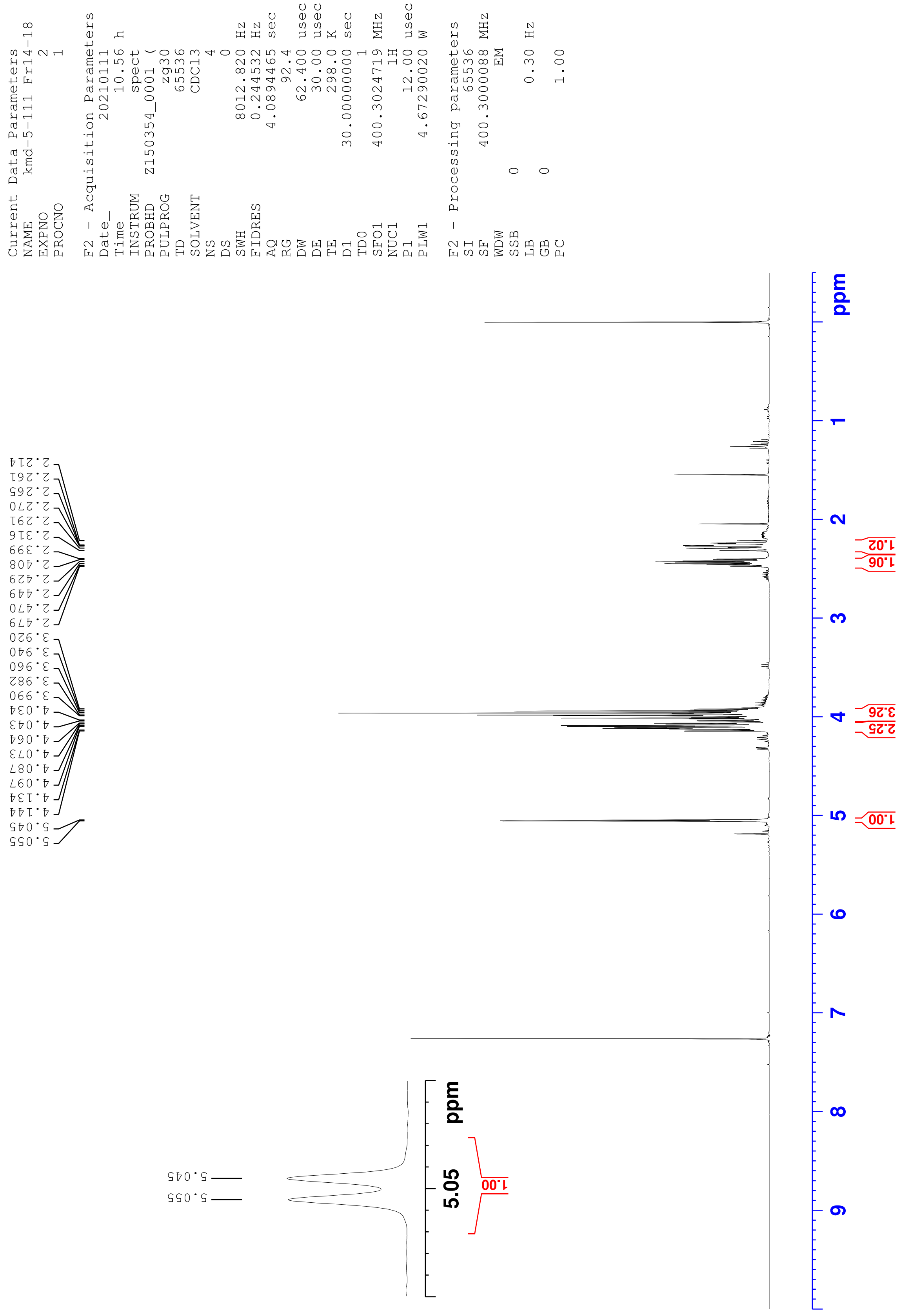
Current Data Parameters
NAME kmd-5-111 Fr14-18
EXPNO 2
PROCNO 1
F2 - Acquisition Parameters
Date_ 20210111
Time 10.56 h
INSTRUM spect
PROBHD Z150354_0001 (
PULPROG zg30
TD 65536
SOLVENT CDCl3
NS 4
DS 0
SWH 8012.820 Hz
FIDRES 0.244532 Hz
AQ 4.0894465 sec
RG 92.4
DW 62.400 usec
DE 30.00 usec
TE 298.0 K
D1 30.00000000 sec
TD0 1
SFO1 400.3024719 MHz
NUC1 1H
P1 12.00 usec
PLW1 4.67290020 W
F2 - Processing parameters
SI 65536
SF 400.3000088 MHz
WDW EM
SSB 0
LB 0.30 Hz
GB 0
PC 1.00
2.214
2.261
2.265
2.270
2.291
2.316
2.399
2.408
2.429
2.449
2.470
2.479
3.920
3.940
3.960
3.982
3.990
4.034
4.043
4.064
4.073
4.087
4.097
4.134
4.144
5.045
5.055
5.045
5.055
5.05
ppm
1.00
1.02
1.06
3.26
2.25
1.00
9
8
7
6
5
4
3
2
1
ppm

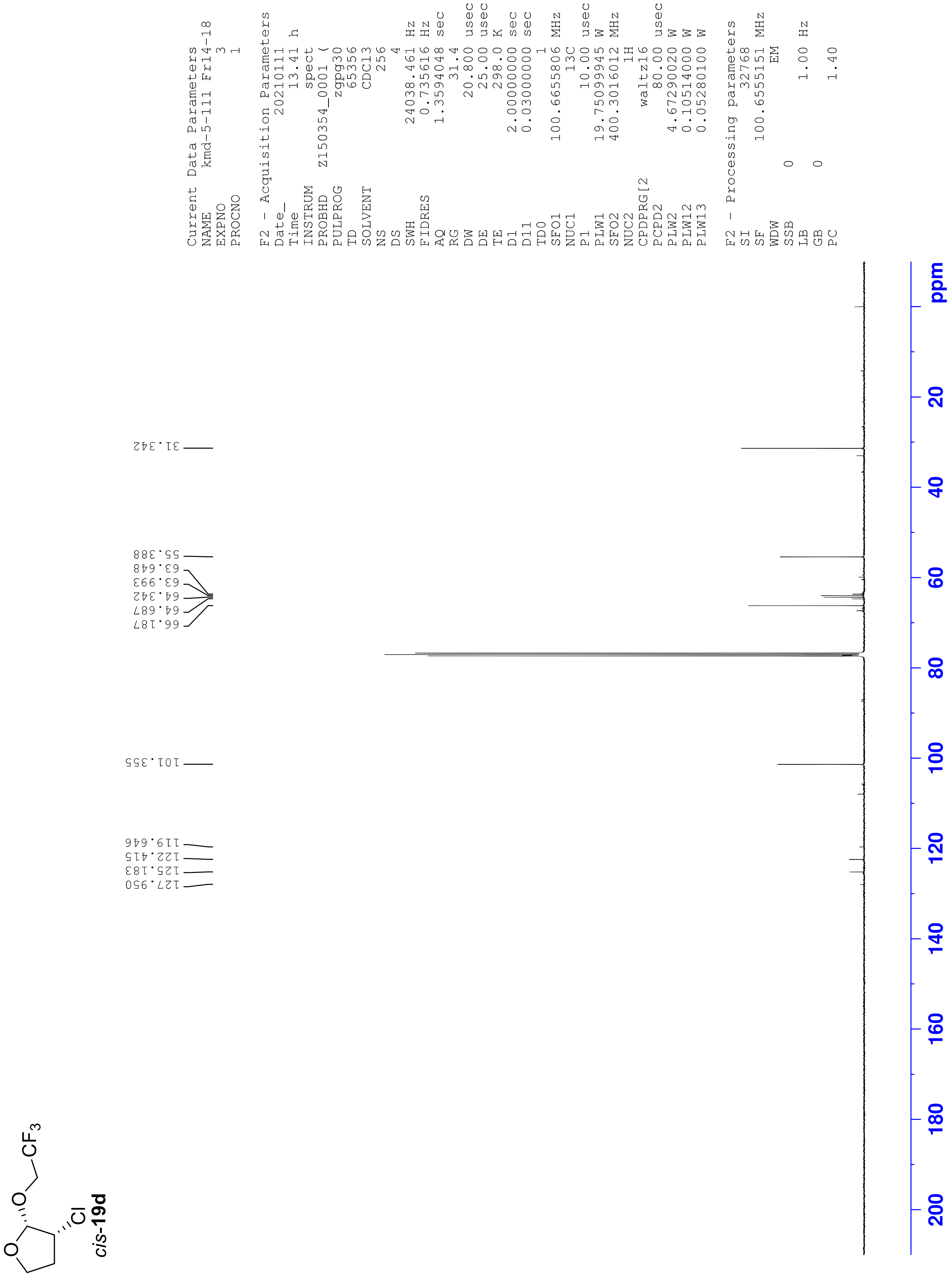
Current Data Parameters
NAME kmd-5-111 Fr14-18
EXPNO 3
PROCNO 1
F2 - Acquisition Parameters
Date_ 20210111
Time 13.41 h
INSTRUM spect
PROBHD Z150354_0001 (
PULPROG zgpg30
TD 65356
SOLVENT CDCl3
NS 256
DS 4
SWH 24038.461 Hz
FIDRES 0.735616 Hz
AQ 1.3594048 sec
RG 31.4
DW 20.800 usec
DE 25.00 usec
TE 298.0 K
D1 2.00000000 sec
D11 0.03000000 sec
TD0 1
SFO1 100.6655806 MHz
NUC1 13C
P1 10.00 usec
PLW1 19.75099945 W
SFO2 400.3016012 MHz
NUC2 1H
CPDPRG[2 waltz16
PCPD2 80.00 usec
PLW2 4.67290020 W
PLW12 0.10514000 W
PLW13 0.05280100 W
F2 - Processing parameters
SI 32768
SF 100.6555151 MHz
WDW EM
SSB 0
LB 1.00 Hz
GB 0
PC 1.40
31.342
55.388
63.648
63.993
64.342
64.687
66.187
101.355
119.646
122.415
125.183
127.950
ppm
20
40
60
80
100
120
140
160
180
200
CF3
O
O
Cl
cis-19d

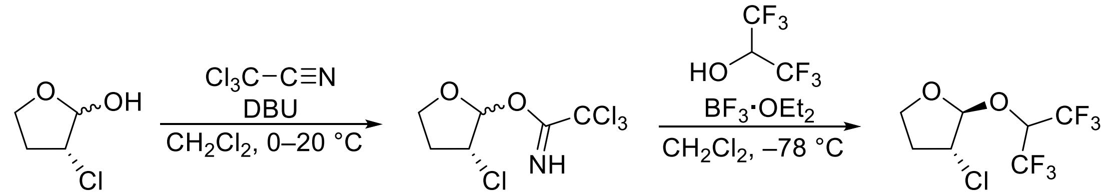

15 **17** **19e**

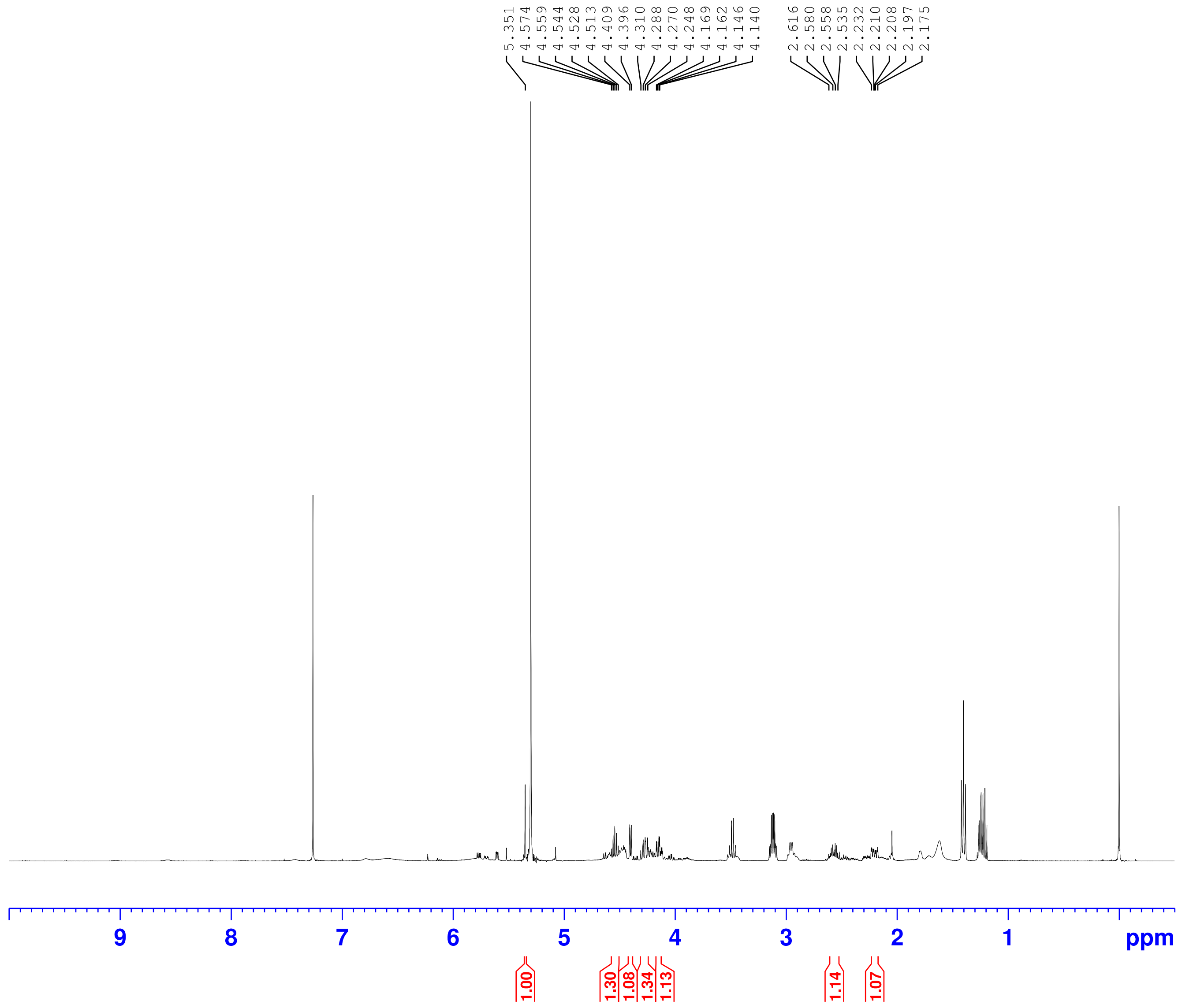


```
Current Data Parameters
NAME              kmd-5-049
EXPNO                     2
PROCNO                    1

F2 - Acquisition Parameters
Date_              20201109
Time                  18.29 h
INSTRUM               spect
PROBHD    Z150354_0001 (
PULPROG                zg30
TD                    65536
SOLVENT               CDCl3
NS                        4
DS                        0
SWH                8012.820 Hz
FIDRES             0.244532 Hz
AQ                4.0894465 sec
RG                     92.4
DW                   62.400 usec
DE                    30.00 usec
TE                    298.0 K
D1              30.00000000 sec
TD0                       1
SFO1            400.3024719 MHz
NUC1                     1H
P1                    12.00 usec
PLW1             4.67290020 W

F2 - Processing parameters
SI                    65536
SF              400.3000082 MHz
WDW                      EM
SSB       0
LB                     0.30 Hz
GB        0
PC                     1.00
```

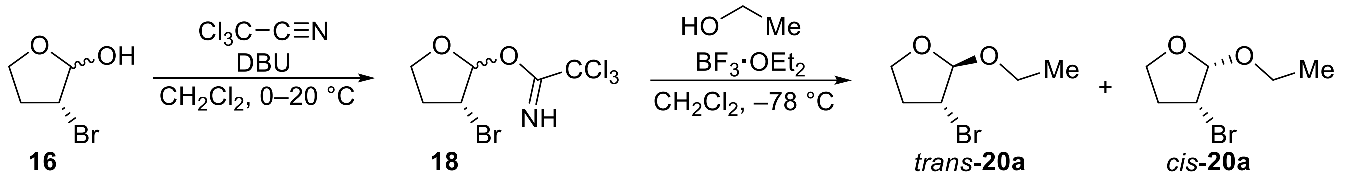

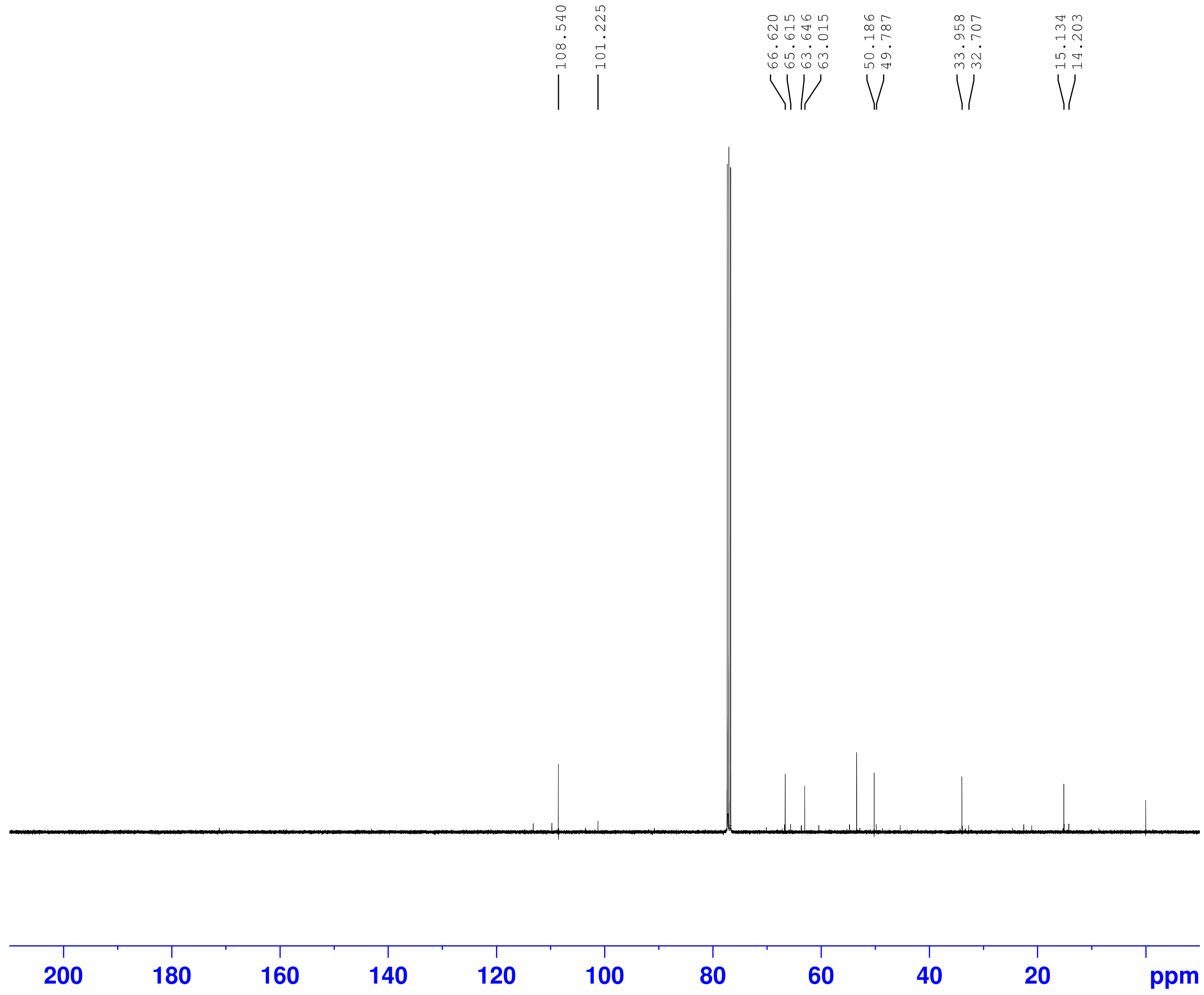


```
Current Data Parameters
NAME              kmd-5-034
EXPNO                     3
PROCNO                    1

F2 - Acquisition Parameters
Date_              20201026
Time                  23.56 h
INSTRUM               spect
PROBHD    Z150354_0001 (
PULPROG             zgpg30
TD                   65356
SOLVENT              CDCl3
NS                     512
DS                       4
SWH              24038.461 Hz
FIDRES            0.735616 Hz
AQ               1.3594048 sec
RG                   45.21
DW                  20.800 usec
DE                   25.00 usec
TE                   298.0 K
D1              2.00000000 sec
D11             0.03000000 sec
TD0                      1
SFO1          100.6655806 MHz
NUC1                   13C
P1                   10.00 usec
PLW1           19.75099945 W
SFO2          400.3016012 MHz
NUC2                    1H
CPDPRG[2           waltz16
PCPD2                80.00 usec
PLW2            4.67290020 W
PLW12           0.10514000 W
PLW13           0.05280100 W

F2 - Processing parameters
SI                  131072
SF            100.6555158 MHz
WDW                     EM
SSB       0
LB        0 Hz
GB        0
PC                    1.40
```

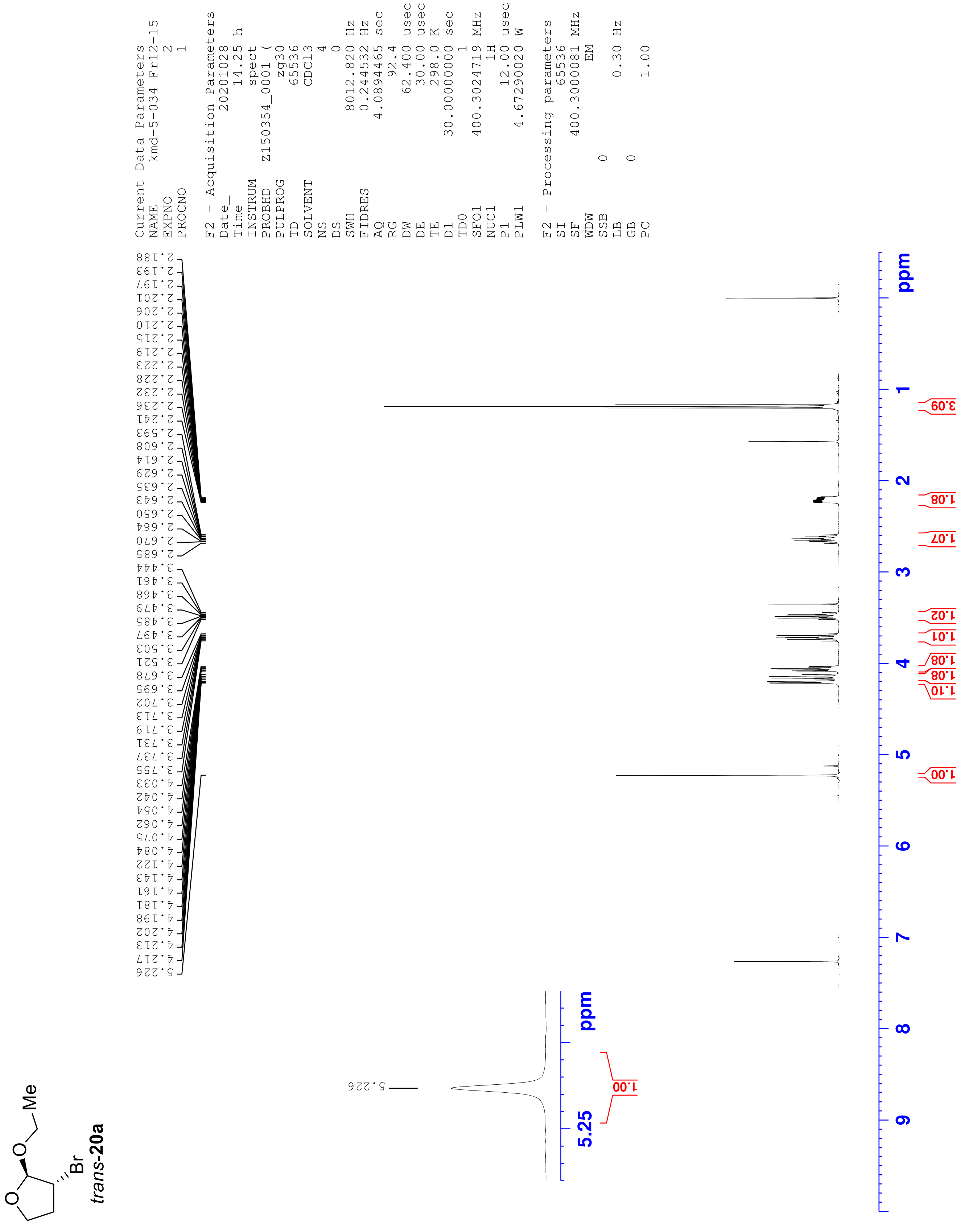
Current Data Parameters
NAME kmd-5-034 Fr12-15
EXPNO 2
PROCNO 1
F2 - Acquisition Parameters
Date_ 20201028
Time 14.25 h
INSTRUM spect
PROBHD Z150354_0001 (
PULPROG zg30
TD 65536
SOLVENT CDCl3
NS 4
DS 0
SWH 8012.820 Hz
FIDRES 0.244532 Hz
AQ 4.0894465 sec
RG 92.4
DW 62.400 usec
DE 30.00 usec
TE 298.0 K
D1 30.00000000 sec
TD0 1
SFO1 400.3024719 MHz
NUC1 1H
P1 12.00 usec
PLW1 4.67290020 W
F2 - Processing parameters
SI 65536
SF 400.3000081 MHz
WDW EM
SSB 0
LB 0.30 Hz
GB 0
PC 1.00
2.188
2.193
2.197
2.201
2.206
2.210
2.215
2.219
2.223
2.228
2.232
2.236
2.241
2.593
2.608
2.614
2.629
2.635
2.643
2.650
2.664
2.670
2.685
3.444
3.461
3.468
3.479
3.485
3.497
3.503
3.521
3.678
3.695
3.702
3.713
3.719
3.731
3.737
3.755
4.033
4.042
4.054
4.062
4.075
4.084
4.122
4.143
4.161
4.181
4.198
4.202
4.213
4.217
5.226
3.09
1.08
1.07
1.02
1.01
1.08
1.08
1.10
1.00
ppm
1
2
3
4
5
6
7
8
9
5.226
1.00
5.25
ppm
Me
O
O
Br
trans-20a

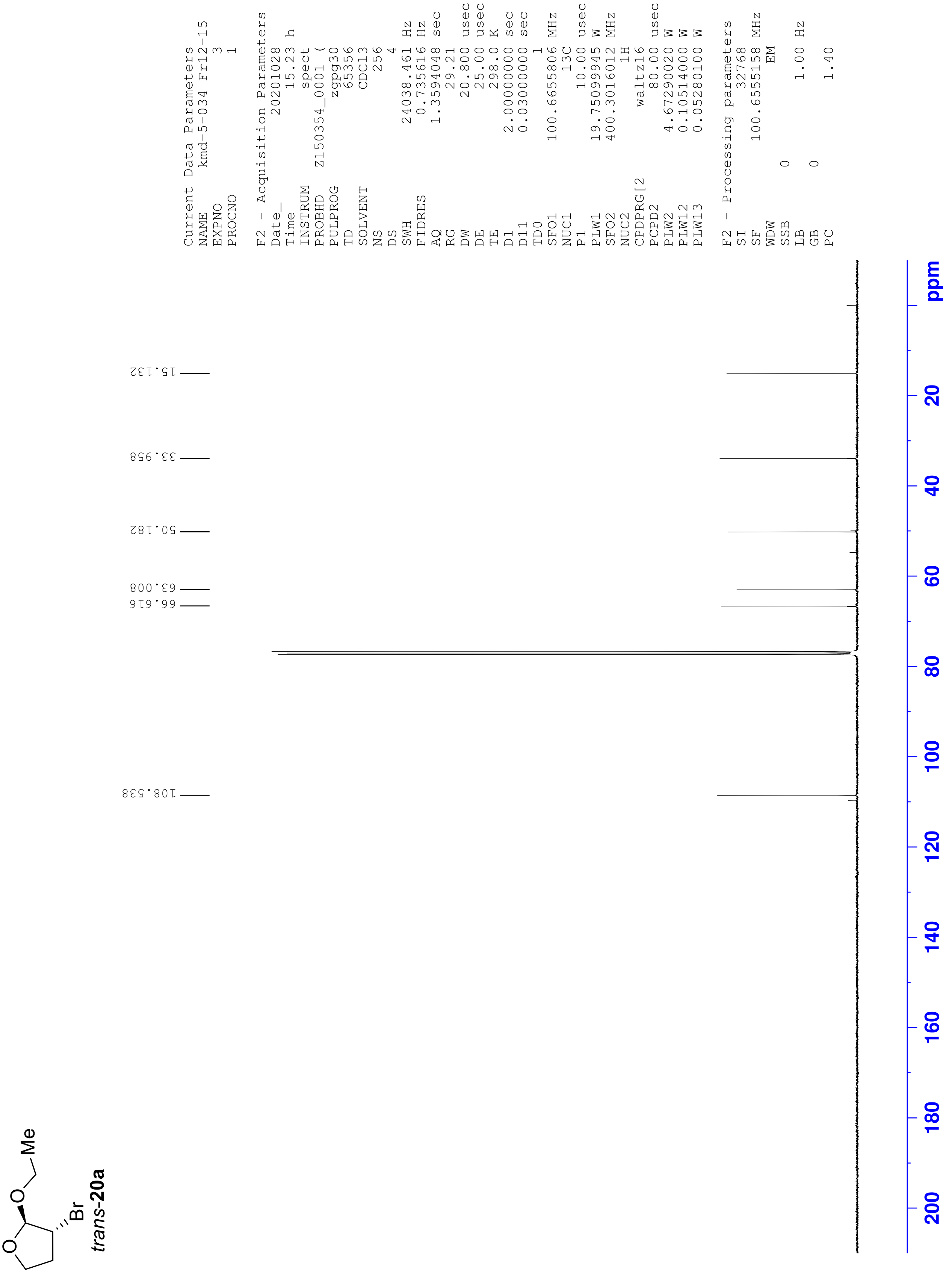
Current Data Parameters
NAME kmd-5-034 Fr12-15
EXPNO 3
PROCNO 1
F2 - Acquisition Parameters
Date_ 20201028
Time 15.23 h
INSTRUM spect
PROBHD Z150354_0001 (
PULPROG zgpg30
TD 65356
SOLVENT CDCl3
NS 256
DS 4
SWH 24038.461 Hz
FIDRES 0.735616 Hz
AQ 1.3594048 sec
RG 29.21
DW 20.800 usec
DE 25.00 usec
TE 298.0 K
D1 2.00000000 sec
D11 0.03000000 sec
TD0 1
SFO1 100.6655806 MHz
NUC1 13C
P1 10.00 usec
PLW1 19.75099945 W
SFO2 400.3016012 MHz
NUC2 1H
CPDPRG[2 waltz16
PCPD2 80.00 usec
PLW2 4.67290020 W
PLW12 0.10514000 W
PLW13 0.05280100 W
F2 - Processing parameters
SI 32768
SF 100.6555158 MHz
WDW EM
SSB 0
LB 1.00 Hz
GB 0
PC 1.40
15.132
33.958
50.182
63.008
66.616
108.538
ppm
20
40
60
80
100
120
140
160
180
200
O
O
Me
Br
trans-20a

WILEY-VCH

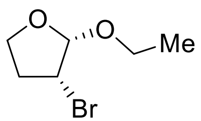

cis-**20a**

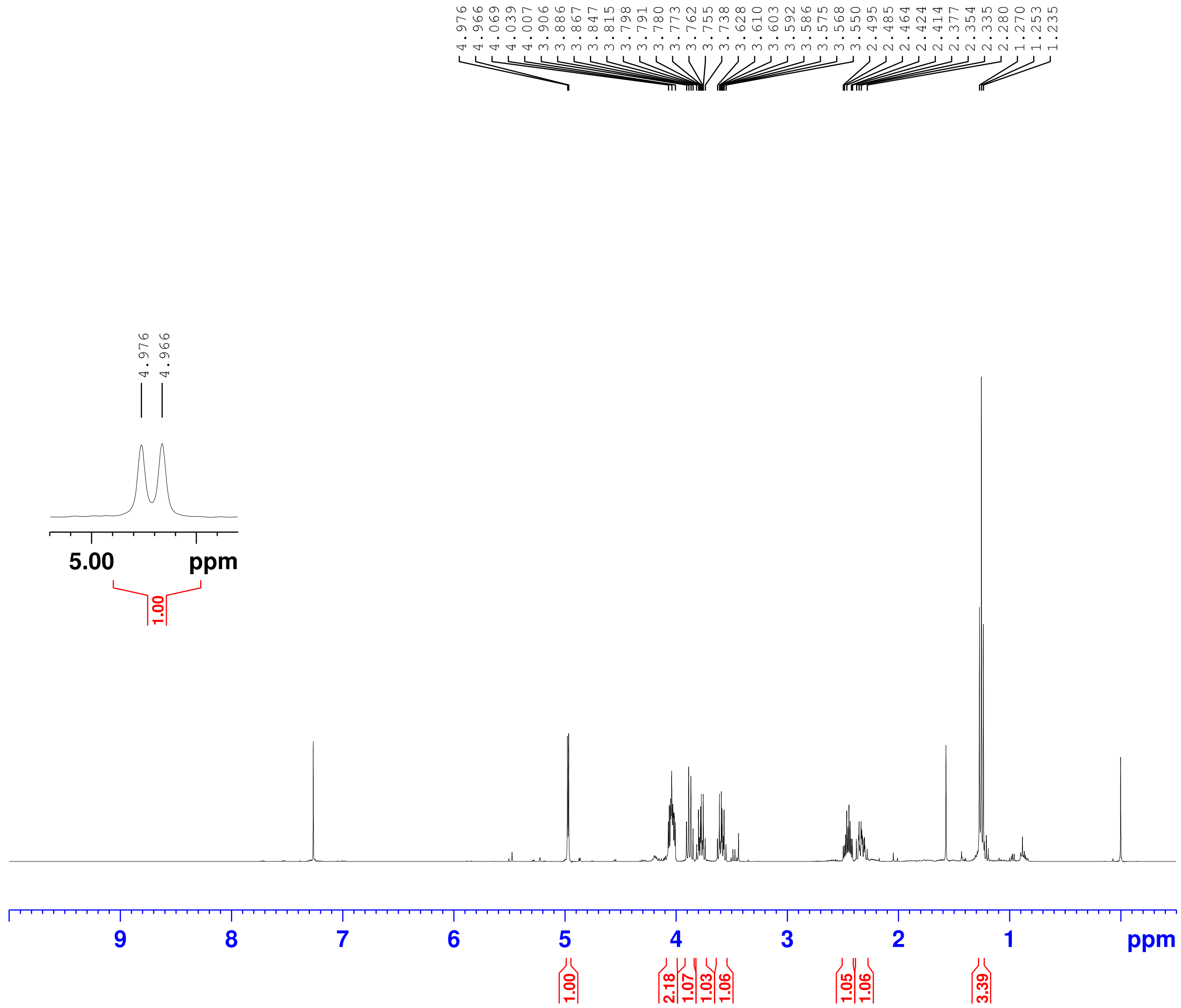


```
Current Data Parameters
NAME      kmd-5-034 Fr19-22
EXPNO                   2
PROCNO                  1

F2 - Acquisition Parameters
Date_            20201028
Time                14.34 h
INSTRUM             spect
PROBHD   Z150354_0001 (
PULPROG              zg30
TD                  65536
SOLVENT             CDCl3
NS                      4
DS                      0
SWH              8012.820 Hz
FIDRES           0.244532 Hz
AQ              4.0894465 sec
RG                   92.4
DW                 62.400 usec
DE                  30.00 usec
TE                  298.0 K
D1            30.00000000 sec
TD0                     1
SFO1          400.3024719 MHz
NUC1                   1H
P1                  12.00 usec
PLW1           4.67290020 W

F2 - Processing parameters
SI                  65536
SF            400.3000079 MHz
WDW                    EM
SSB      0
LB                   0.30 Hz
GB       0
PC                   1.00
```

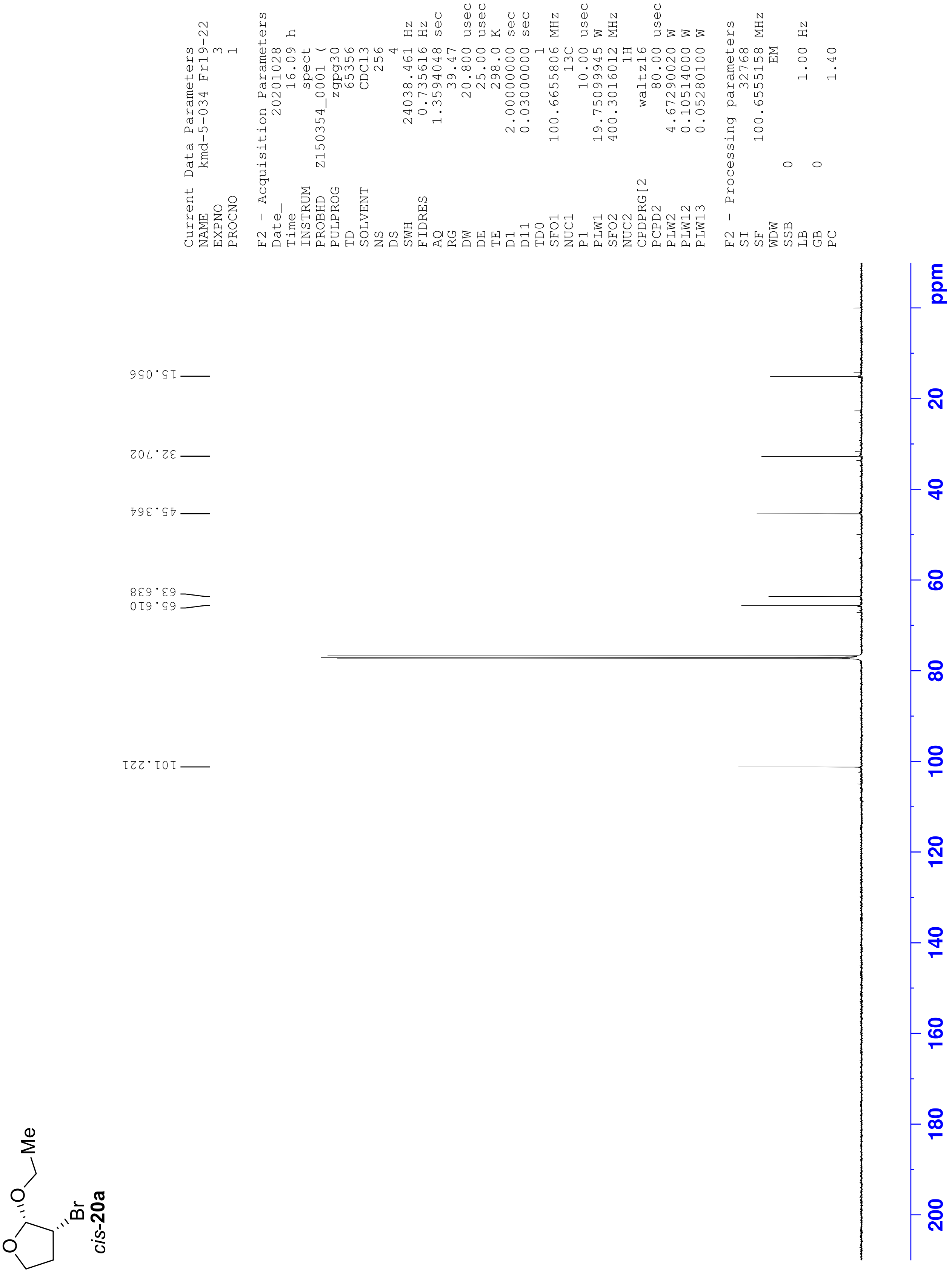
Current Data Parameters
NAME kmd-5-034 Fr19-22
EXPNO 3
PROCNO 1
F2 - Acquisition Parameters
Date_ 20201028
Time 16.09 h
INSTRUM spect
PROBHD Z150354_0001 (
PULPROG zgpg30
TD 65356
SOLVENT CDCl3
NS 256
DS 4
SWH 24038.461 Hz
FIDRES 0.735616 Hz
AQ 1.3594048 sec
RG 39.47
DW 20.800 usec
DE 25.00 usec
TE 298.0 K
D1 2.00000000 sec
D11 0.03000000 sec
TD0 1
SFO1 100.6655806 MHz
NUC1 13C
P1 10.00 usec
PLW1 19.75099945 W
SFO2 400.3016012 MHz
NUC2 1H
CPDPRG[2 waltz16
PCPD2 80.00 usec
PLW2 4.67290020 W
PLW12 0.10514000 W
PLW13 0.05280100 W
F2 - Processing parameters
SI 32768
SF 100.6555158 MHz
WDW EM
SSB 0
LB 1.00 Hz
GB 0
PC 1.40
15.056
32.702
45.364
63.638
65.610
101.221
ppm
20
40
60
80
100
120
140
160
180
200
Me
O
Br
cis-20a

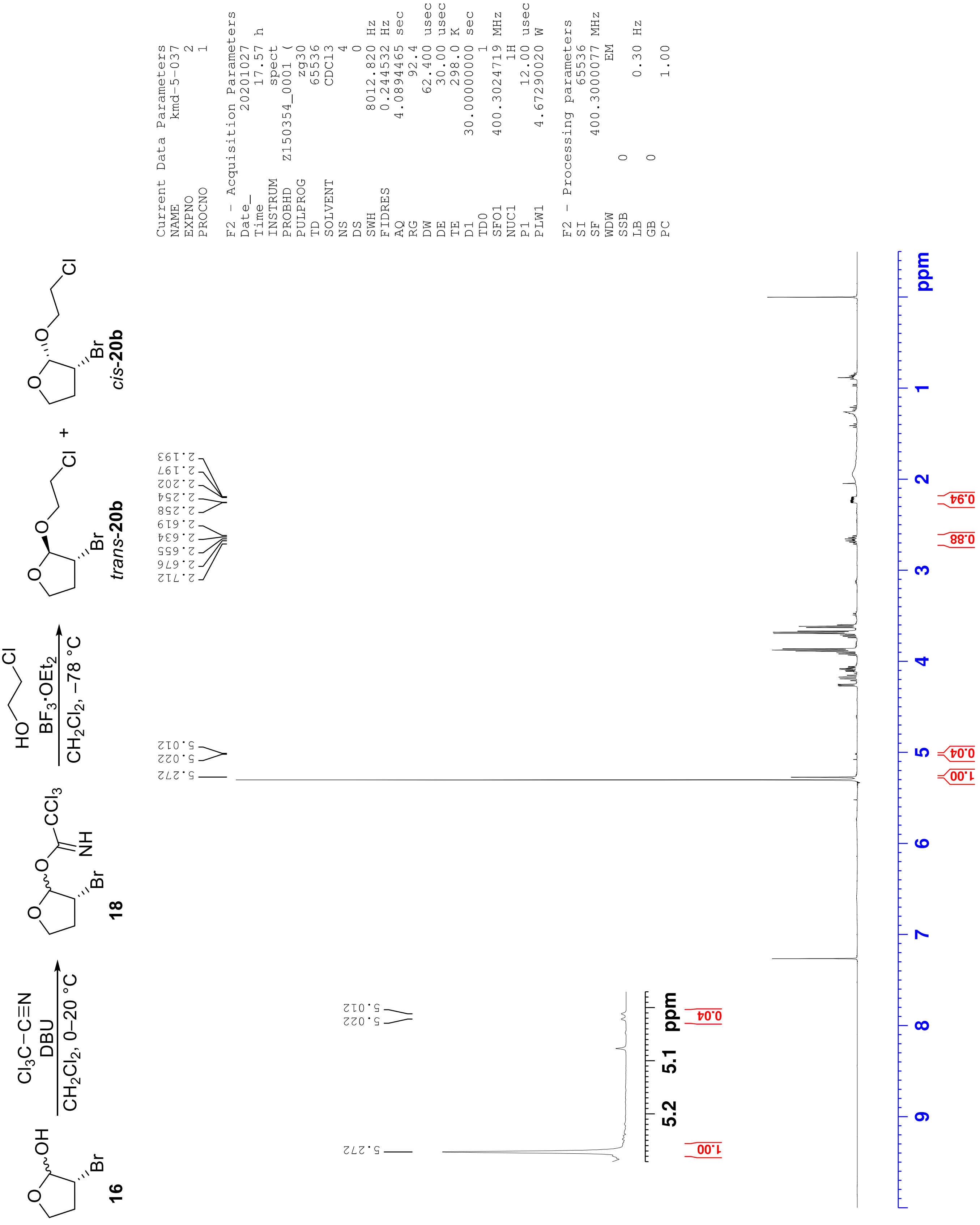
16
18
trans-20b
cis-20b
$Cl_3C-C{\equiv}N$
DBU
CH_2Cl_2, 0–20 °C
HO Cl
$BF_3{\cdot}OEt_2$
CH_2Cl_2, −78 °C
Current Data Parameters
NAME kmd-5-037
EXPNO 2
PROCNO 1
F2 - Acquisition Parameters
Date_ 20201027
Time 17.57 h
INSTRUM spect
PROBHD Z150354_0001 (
PULPROG zg30
TD 65536
SOLVENT CDCl3
NS 4
DS 0
SWH 8012.820 Hz
FIDRES 0.244532 Hz
AQ 4.0894465 sec
RG 92.4
DW 62.400 usec
DE 30.00 usec
TE 298.0 K
D1 30.00000000 sec
TD0 1
SFO1 400.3024719 MHz
NUC1 1H
P1 12.00 usec
PLW1 4.67290020 W
F2 - Processing parameters
SI 65536
SF 400.3000077 MHz
WDW EM
SSB 0
LB 0.30 Hz
GB 0
PC 1.00
2.193
2.197
2.202
2.254
2.258
2.619
2.634
2.655
2.676
2.712
5.012
5.022
5.272
0.94
0.88
0.04
1.00
ppm

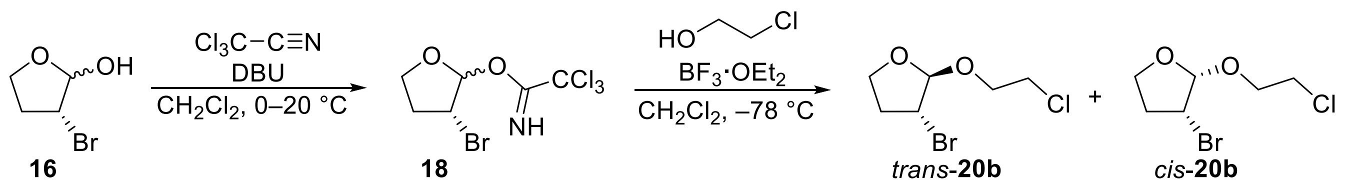

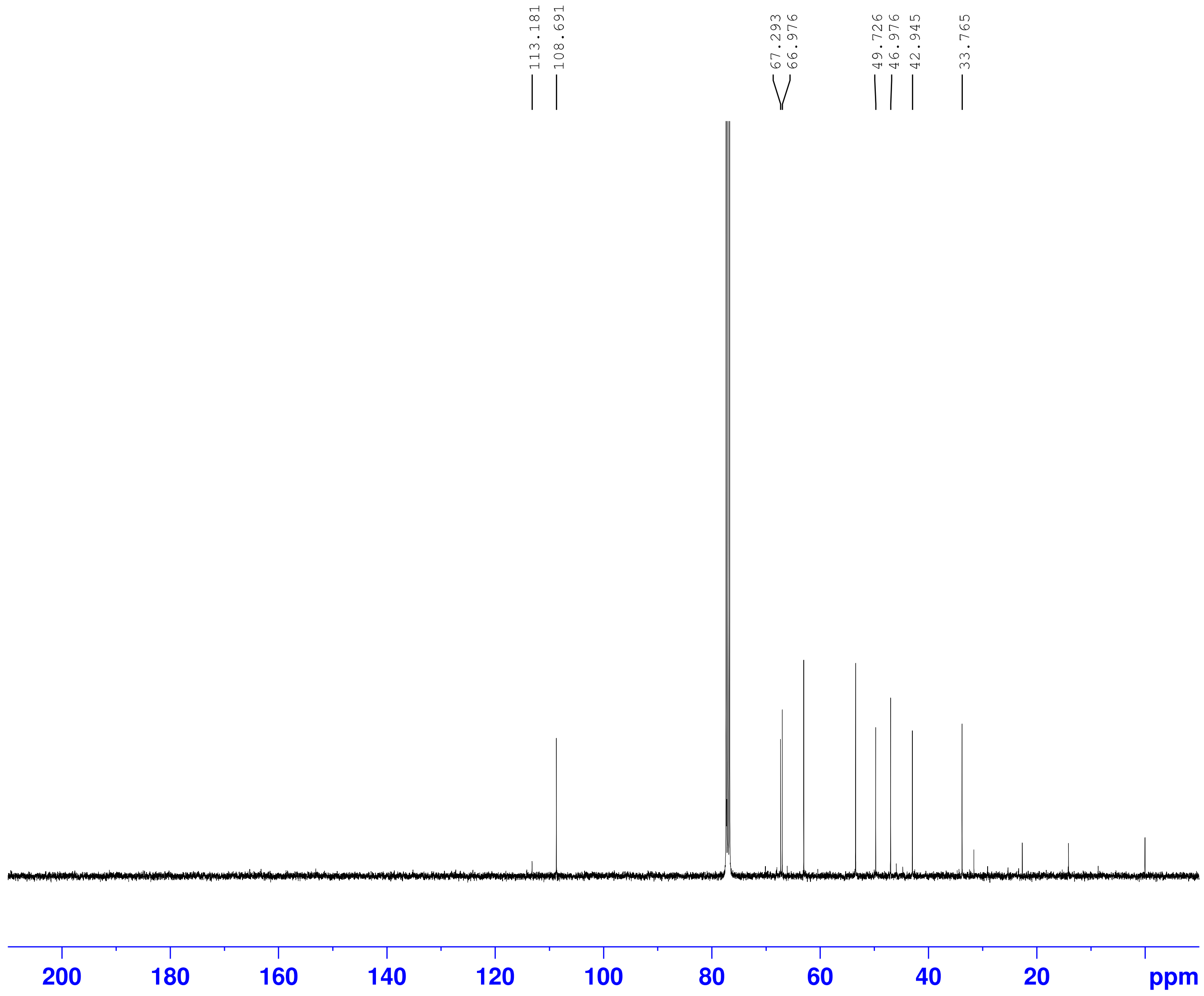


```
Current Data Parameters
NAME              kmd-5-037
EXPNO                     3
PROCNO                    1

F2 - Acquisition Parameters
Date_              20201027
Time                  18.55 h
INSTRUM               spect
PROBHD     Z150354_0001 (
PULPROG             zgpg30
TD                    65356
SOLVENT               CDCl3
NS                      256
DS                        4
SWH               24038.461 Hz
FIDRES             0.735616 Hz
AQ                1.3594048 sec
RG                    29.21
DW                   20.800 usec
DE                    25.00 usec
TE                    298.0 K
D1               2.00000000 sec
D11              0.03000000 sec
TD0                       1
SFO1           100.6655806 MHz
NUC1                    13C
P1                    10.00 usec
PLW1            19.75099945 W
SFO2           400.3016012 MHz
NUC2                     1H
CPDPRG[2            waltz16
PCPD2                 80.00 usec
PLW2             4.67290020 W
PLW12            0.10514000 W
PLW13            0.05280100 W

F2 - Processing parameters
SI                    32768
SF             100.6555156 MHz
WDW                      EM
SSB       0
LB                     1.00 Hz
GB        0
PC                     1.40
```

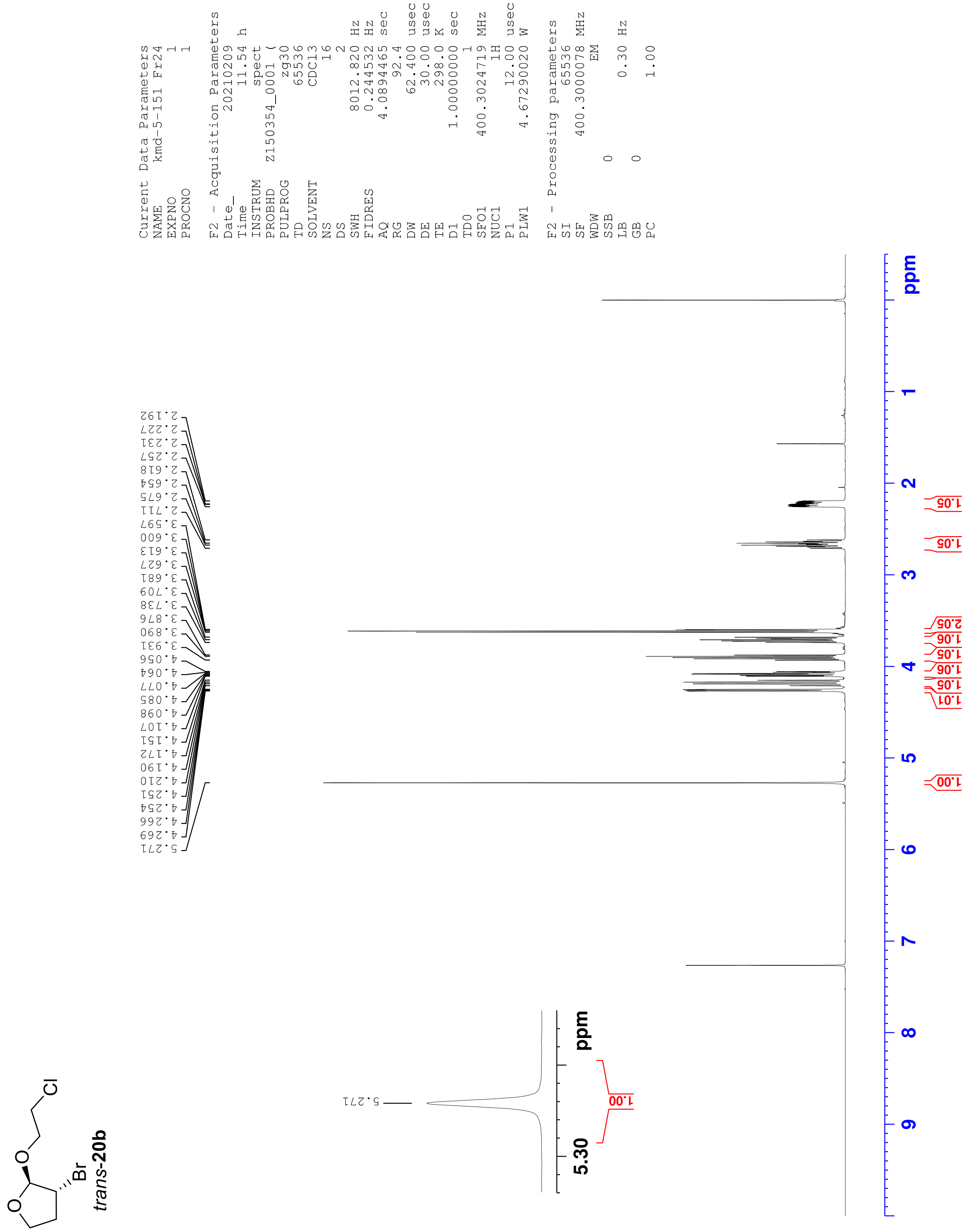
Current Data Parameters
NAME kmd-5-151 Fr24
EXPNO 1
PROCNO 1
F2 - Acquisition Parameters
Date_ 20210209
Time 11.54 h
INSTRUM spect
PROBHD Z150354_0001 (
PULPROG zg30
TD 65536
SOLVENT CDCl3
NS 16
DS 2
SWH 8012.820 Hz
FIDRES 0.244532 Hz
AQ 4.0894465 sec
RG 92.4
DW 62.400 usec
DE 30.00 usec
TE 298.0 K
D1 1.00000000 sec
TD0 1
SFO1 400.3024719 MHz
NUC1 1H
P1 12.00 usec
PLW1 4.67290020 W
F2 - Processing parameters
SI 65536
SF 400.3000078 MHz
WDW EM
SSB 0
LB 0.30 Hz
GB 0
PC 1.00
2.192
2.227
2.231
2.257
2.618
2.654
2.675
2.711
3.597
3.600
3.613
3.627
3.681
3.709
3.738
3.876
3.890
3.931
4.056
4.064
4.077
4.085
4.098
4.107
4.151
4.172
4.190
4.210
4.251
4.254
4.266
4.269
5.271
1.05
1.05
2.05
1.06
1.05
1.06
1.05
1.01
1.00
ppm
5.30
ppm
Cl
O
Br
trans-20b

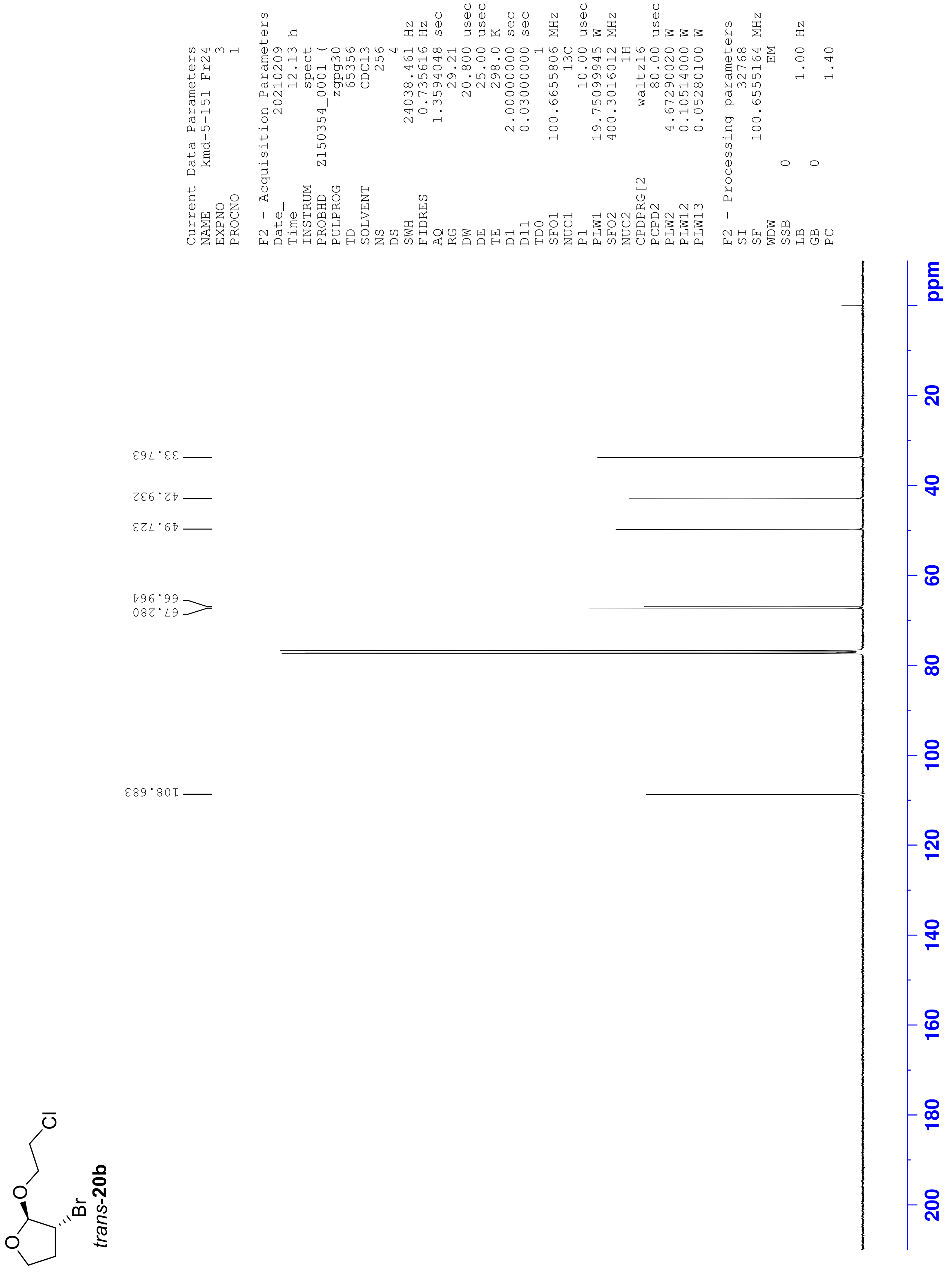
Current Data Parameters
NAME kmd-5-151 Fr24
EXPNO 3
PROCNO 1
F2 - Acquisition Parameters
Date_ 20210209
Time 12.13 h
INSTRUM spect
PROBHD Z150354_0001 (
PULPROG zgpg30
TD 65356
SOLVENT CDCl3
NS 256
DS 4
SWH 24038.461 Hz
FIDRES 0.735616 Hz
AQ 1.3594048 sec
RG 29.21
DW 20.800 usec
DE 25.00 usec
TE 298.0 K
D1 2.00000000 sec
D11 0.03000000 sec
TD0 1
SFO1 100.6655806 MHz
NUC1 13C
P1 10.00 usec
PLW1 19.75099945 W
SFO2 400.3016012 MHz
NUC2 1H
CPDPRG[2 waltz16
PCPD2 80.00 usec
PLW2 4.67290020 W
PLW12 0.10514000 W
PLW13 0.05280100 W
F2 - Processing parameters
SI 32768
SF 100.6555164 MHz
WDW EM
SSB 0
LB 1.00 Hz
GB 0
PC 1.40
33.763
42.932
49.723
66.964
67.280
108.683
ppm
20
40
60
80
100
120
140
160
180
200
Cl
O
O
Br
trans-20b

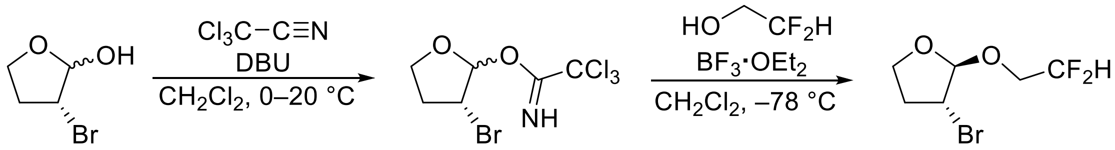

16 18 20c

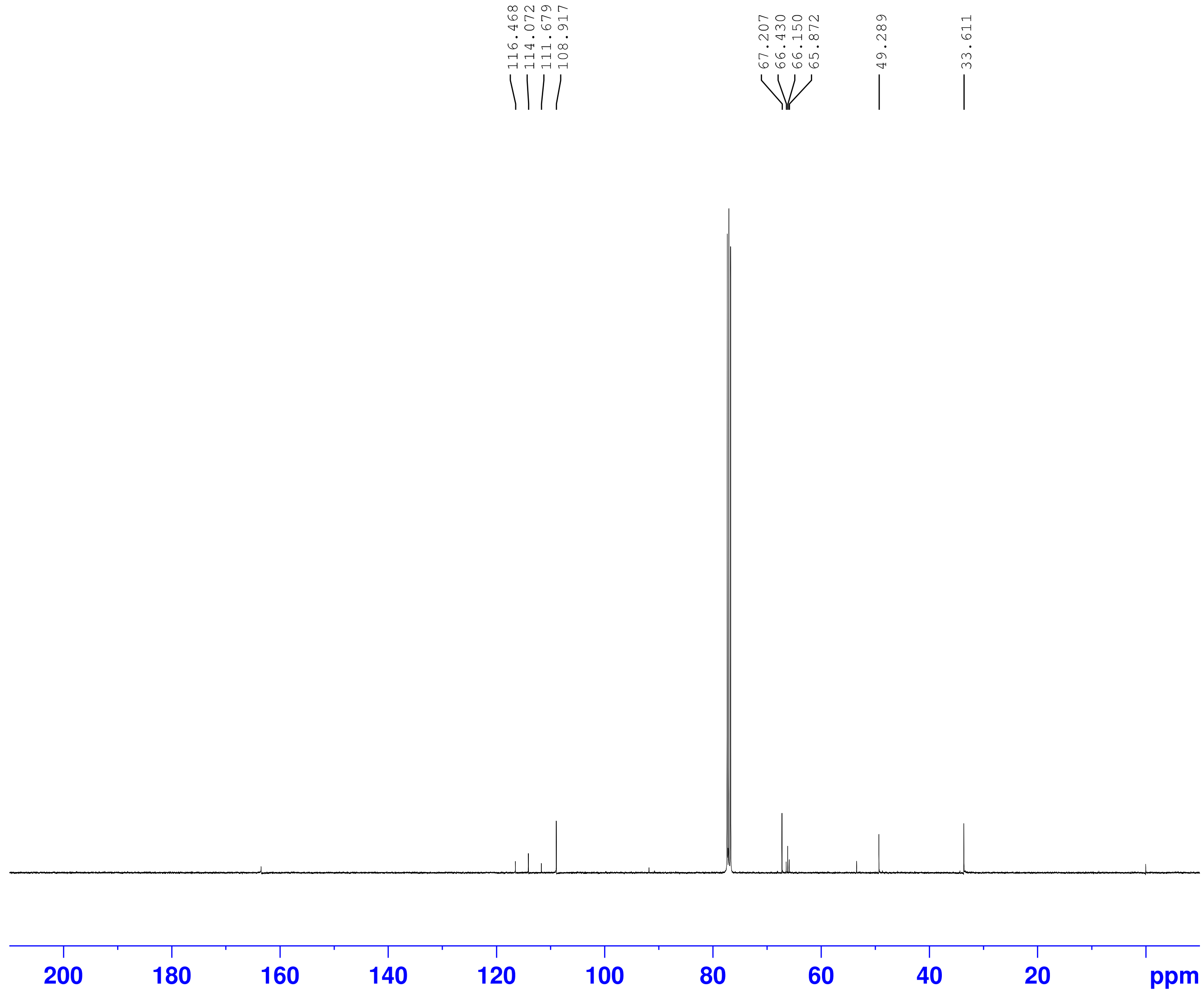


```
Current Data Parameters
NAME              kmd-5-036
EXPNO                     3
PROCNO                    1

F2 - Acquisition Parameters
Date_              20201027
Time                   1.03 h
INSTRUM               spect
PROBHD     Z150354_0001 (
PULPROG             zgpg30
TD                   65356
SOLVENT              CDCl3
NS                     512
DS                       4
SWH              24038.461 Hz
FIDRES            0.735616 Hz
AQ               1.3594048 sec
RG                   45.21
DW                  20.800 usec
DE                   25.00 usec
TE                   298.0 K
D1              2.00000000 sec
D11             0.03000000 sec
TD0                      1
SFO1          100.6655806 MHz
NUC1                   13C
P1                   10.00 usec
PLW1           19.75099945 W
SFO2          400.3016012 MHz
NUC2                    1H
CPDPRG[2           waltz16
PCPD2                80.00 usec
PLW2            4.67290020 W
PLW12           0.10514000 W
PLW13           0.05280100 W

F2 - Processing parameters
SI                   32768
SF            100.6555158 MHz
WDW                     EM
SSB       0
LB                    1.00 Hz
GB        0
PC                    1.40
```

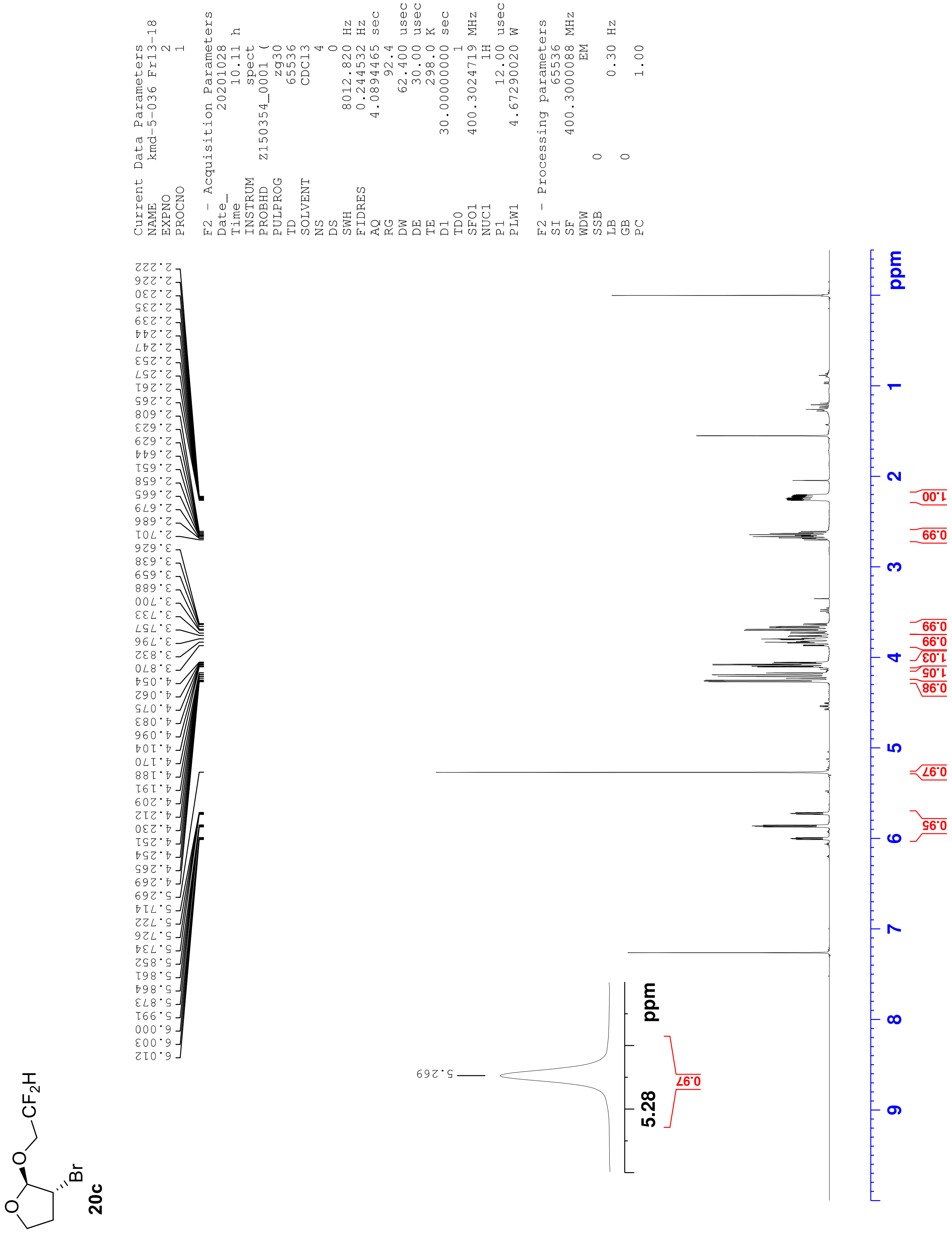
20c
CF2H
Br
O
Current Data Parameters
NAME kmd-5-036 Fr13-18
EXPNO 2
PROCNO 1
F2 - Acquisition Parameters
Date_ 20201028
Time 10.11 h
INSTRUM spect
PROBHD Z150354_0001 (
PULPROG zg30
TD 65536
SOLVENT CDCl3
NS 4
DS 0
SWH 8012.820 Hz
FIDRES 0.244532 Hz
AQ 4.0894465 sec
RG 92.4
DW 62.400 usec
DE 30.00 usec
TE 298.0 K
D1 30.00000000 sec
TD0 1
SFO1 400.3024719 MHz
NUC1 1H
P1 12.00 usec
PLW1 4.67290020 W
F2 - Processing parameters
SI 65536
SF 400.3000088 MHz
WDW EM
SSB 0
LB 0.30 Hz
GB 0
PC 1.00
2.222 2.226 2.230 2.235 2.239 2.244 2.247 2.253 2.257 2.261 2.265 2.608 2.623 2.629 2.644 2.651 2.658 2.665 2.679 2.686 2.701 3.626 3.638 3.659 3.688 3.700 3.733 3.757 3.796 3.832 3.870 4.054 4.062 4.075 4.083 4.096 4.104 4.170 4.188 4.191 4.209 4.212 4.230 4.251 4.254 4.265 4.269 5.269 5.714 5.722 5.726 5.734 5.852 5.861 5.864 5.873 5.991 6.000 6.003 6.012
5.269
5.28
ppm
0.97
1.00
0.99
0.99
0.99
1.03
1.05
0.98
0.97
0.95
ppm
1
2
3
4
5
6
7
8
9

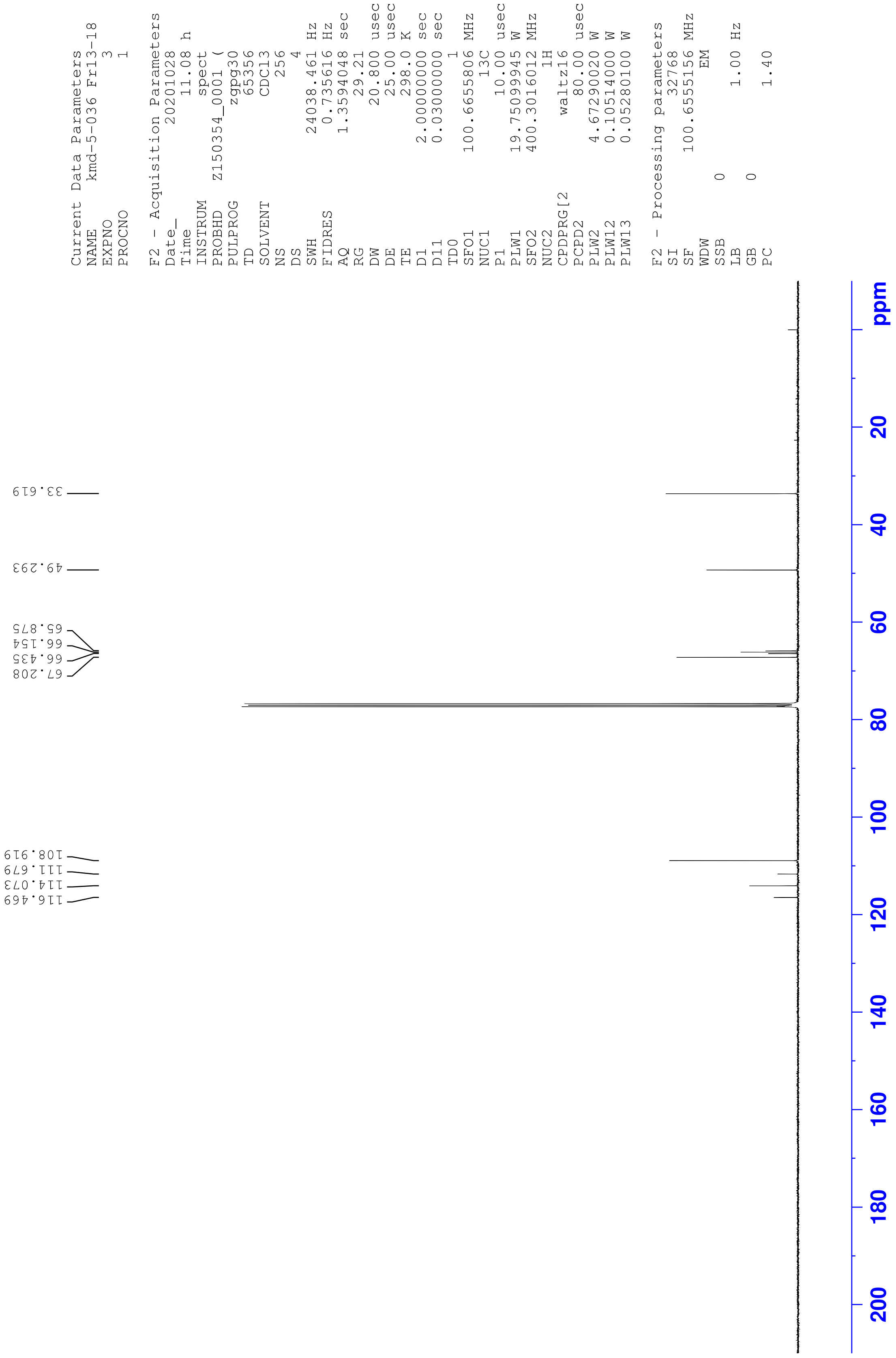
Current Data Parameters
NAME kmd-5-036 Fr13-18
EXPNO 3
PROCNO 1
F2 - Acquisition Parameters
Date_ 20201028
Time 11.08 h
INSTRUM spect
PROBHD Z150354_0001 (
PULPROG zgpg30
TD 65356
SOLVENT CDCl3
NS 256
DS 4
SWH 24038.461 Hz
FIDRES 0.735616 Hz
AQ 1.3594048 sec
RG 29.21
DW 20.800 usec
DE 25.00 usec
TE 298.0 K
D1 2.00000000 sec
D11 0.03000000 sec
TD0 1
SFO1 100.6655806 MHz
NUC1 13C
P1 10.00 usec
PLW1 19.75099945 W
SFO2 400.3016012 MHz
NUC2 1H
CPDPRG[2 waltz16
PCPD2 80.00 usec
PLW2 4.67290020 W
PLW12 0.10514000 W
PLW13 0.05280100 W
F2 - Processing parameters
SI 32768
SF 100.6555156 MHz
WDW EM
SSB 0
LB 1.00 Hz
GB 0
PC 1.40
33.619
49.293
65.875
66.154
66.435
67.208
108.919
111.679
114.073
116.469
ppm
20
40
60
80
100
120
140
160
180
200

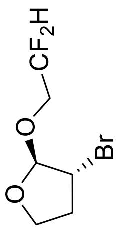
O
O
CF2H
Br

20c

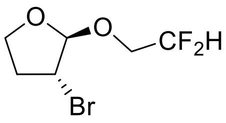

20c

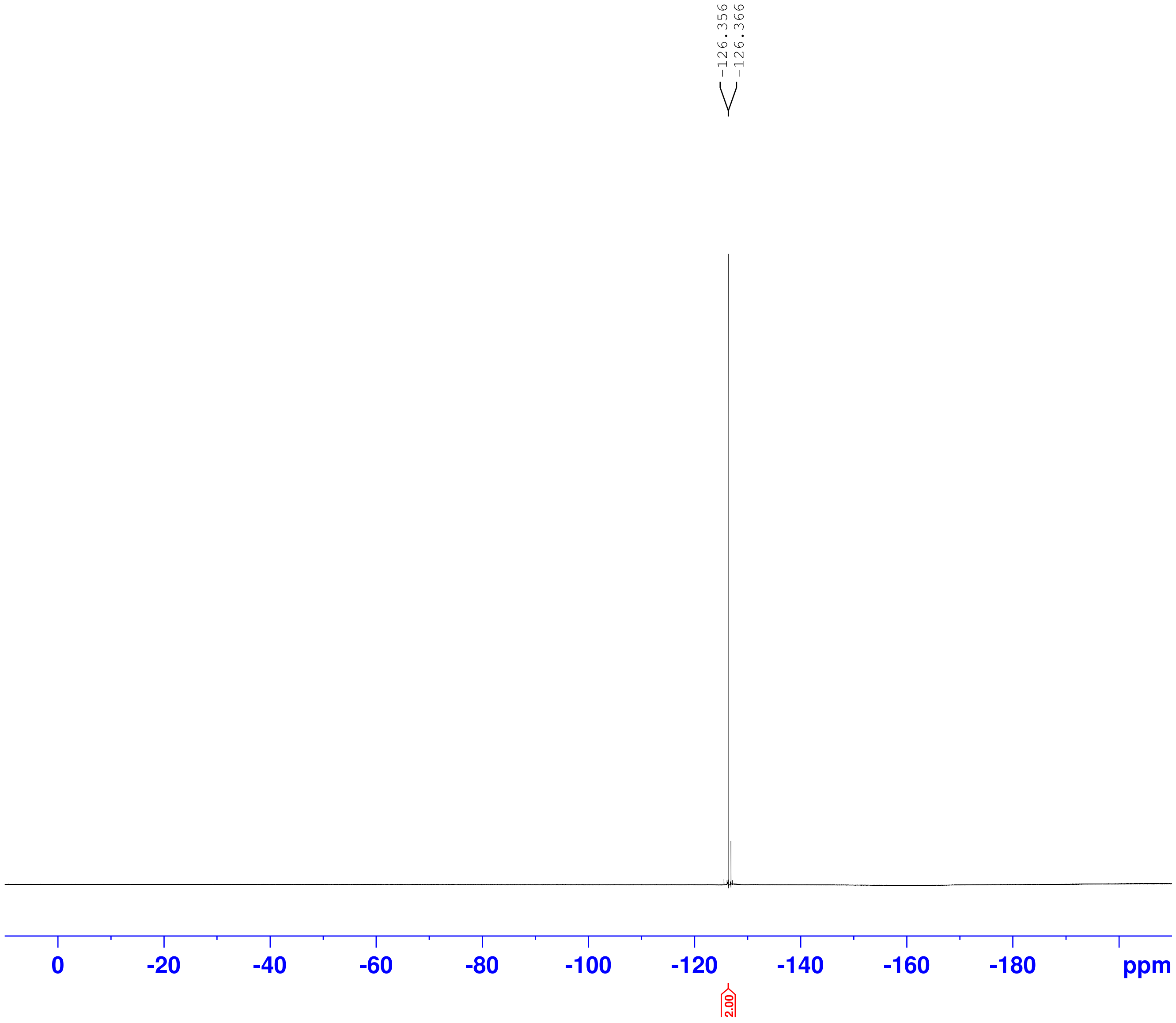

Current Data Parameters	
NAME	kmd-5-036 Fr13-18 19F
EXPNO	1
PROCNO	1

F2 - Acquisition Parameters	
Date_	20201028
Time	17.09 h
INSTRUM	spect
PROBHD	Z108618_0422 (
PULPROG	zgfhigqn.2
TD	131072
SOLVENT	CDCl3
NS	16
DS	4
SWH	89285.711 Hz
FIDRES	1.362392 Hz
AQ	0.7340032 sec
RG	200.67
DW	5.600 usec
DE	6.50 usec
TE	298.2 K
D1	1.00000000 sec
D11	0.03000000 sec
D12	0.00002000 sec
TD0	1
SFO1	376.5453925 MHz
NUC1	19F
P1	15.00 usec
PLW1	16.89999962 W
SFO2	400.2216009 MHz
NUC2	1H
CPDPRG[2	waltz16
PCPD2	90.00 usec
PLW2	14.69999981 W
PLW12	0.40832999 W

F2 - Processing parameters	
SI	65536
SF	376.5834167 MHz
WDW	EM
SSB	0
LB	0.30 Hz
GB	0
PC	1.00

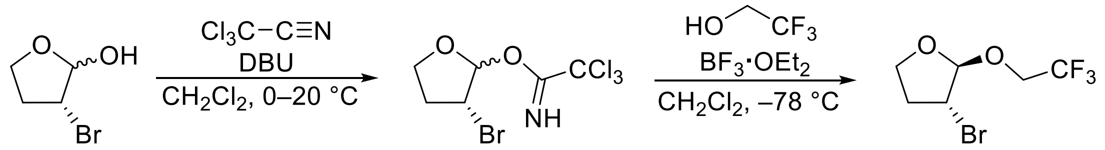

16 18 20d

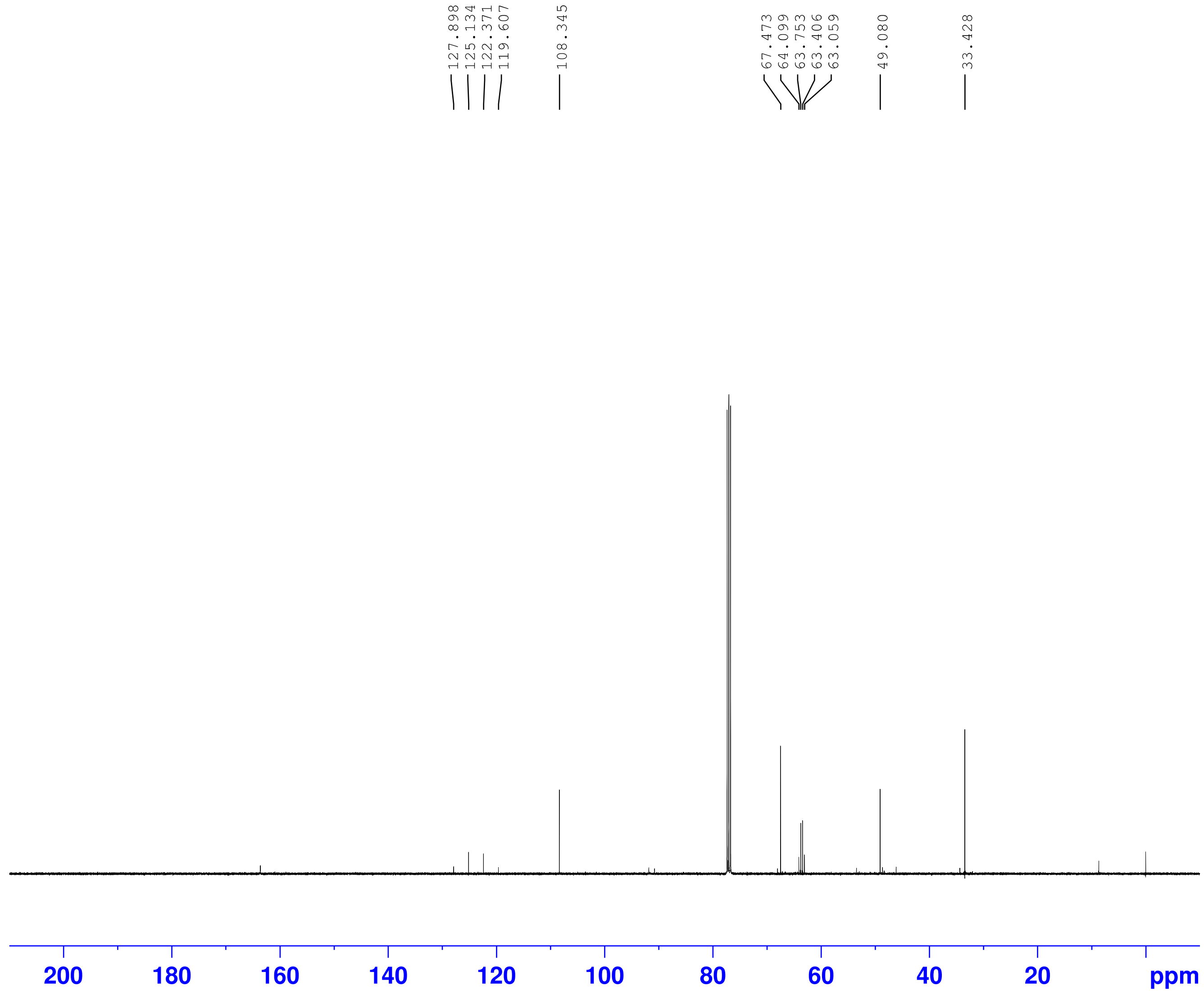


```
Current Data Parameters
NAME           kmd-5-035
EXPNO                  3
PROCNO                 1

F2 - Acquisition Parameters
Date_           20201027
Time                0.30 h
INSTRUM            spect
PROBHD   Z150354_0001 (
PULPROG          zgpg30
TD                65356
SOLVENT           CDCl3
NS                  512
DS                    4
SWH           24038.461 Hz
FIDRES         0.735616 Hz
AQ            1.3594048 sec
RG                63.04
DW               20.800 usec
DE                25.00 usec
TE                298.0 K
D1           2.00000000 sec
D11          0.03000000 sec
TD0                   1
SFO1        100.6655806 MHz
NUC1                13C
P1                10.00 usec
PLW1        19.75099945 W
SFO2        400.3016012 MHz
NUC2                 1H
CPDPRG[2        waltz16
PCPD2             80.00 usec
PLW2         4.67290020 W
PLW12        0.10514000 W
PLW13        0.05280100 W

F2 - Processing parameters
SI               131072
SF          100.6555152 MHz
WDW                  EM
SSB      0
LB       0 Hz
GB       0
PC                 1.40
```

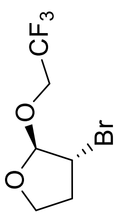

20d

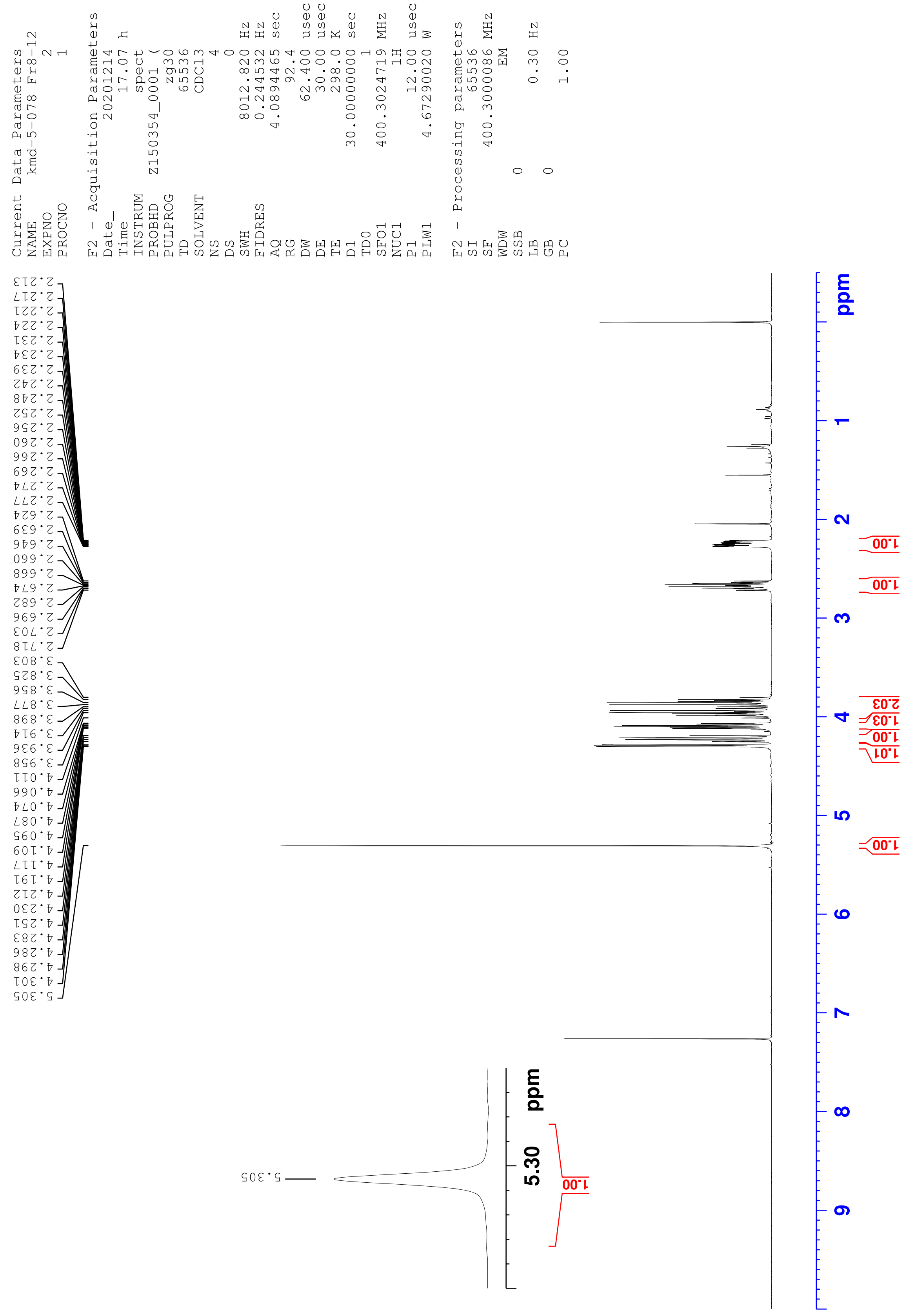
Current Data Parameters
NAME kmd-5-078 Fr8-12
EXPNO 2
PROCNO 1
F2 - Acquisition Parameters
Date_ 20201214
Time 17.07 h
INSTRUM spect
PROBHD Z150354_0001 (
PULPROG zg30
TD 65536
SOLVENT CDCl3
NS 4
DS 0
SWH 8012.820 Hz
FIDRES 0.244532 Hz
AQ 4.0894465 sec
RG 92.4
DW 62.400 usec
DE 30.00 usec
TE 298.0 K
D1 30.00000000 sec
TD0 1
SFO1 400.3024719 MHz
NUC1 1H
P1 12.00 usec
PLW1 4.67290020 W
F2 - Processing parameters
SI 65536
SF 400.3000086 MHz
WDW EM
SSB 0
LB 0.30 Hz
GB 0
PC 1.00
2.213 2.217 2.221 2.224 2.231 2.234 2.239 2.242 2.248 2.252 2.256 2.260 2.266 2.269 2.274 2.277 2.624 2.639 2.646 2.660 2.668 2.674 2.682 2.696 2.703 2.718 3.803 3.825 3.856 3.877 3.898 3.914 3.936 3.958 4.011 4.066 4.074 4.087 4.095 4.109 4.117 4.191 4.212 4.230 4.251 4.283 4.286 4.298 4.301 5.305
5.305
5.30
ppm
1.00
1.00
1.00
2.03
1.03
1.00
1.01
1.00
ppm
1
2
3
4
5
6
7
8
9

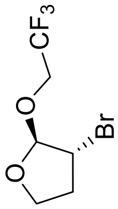
CF3
O
O
Br

20d

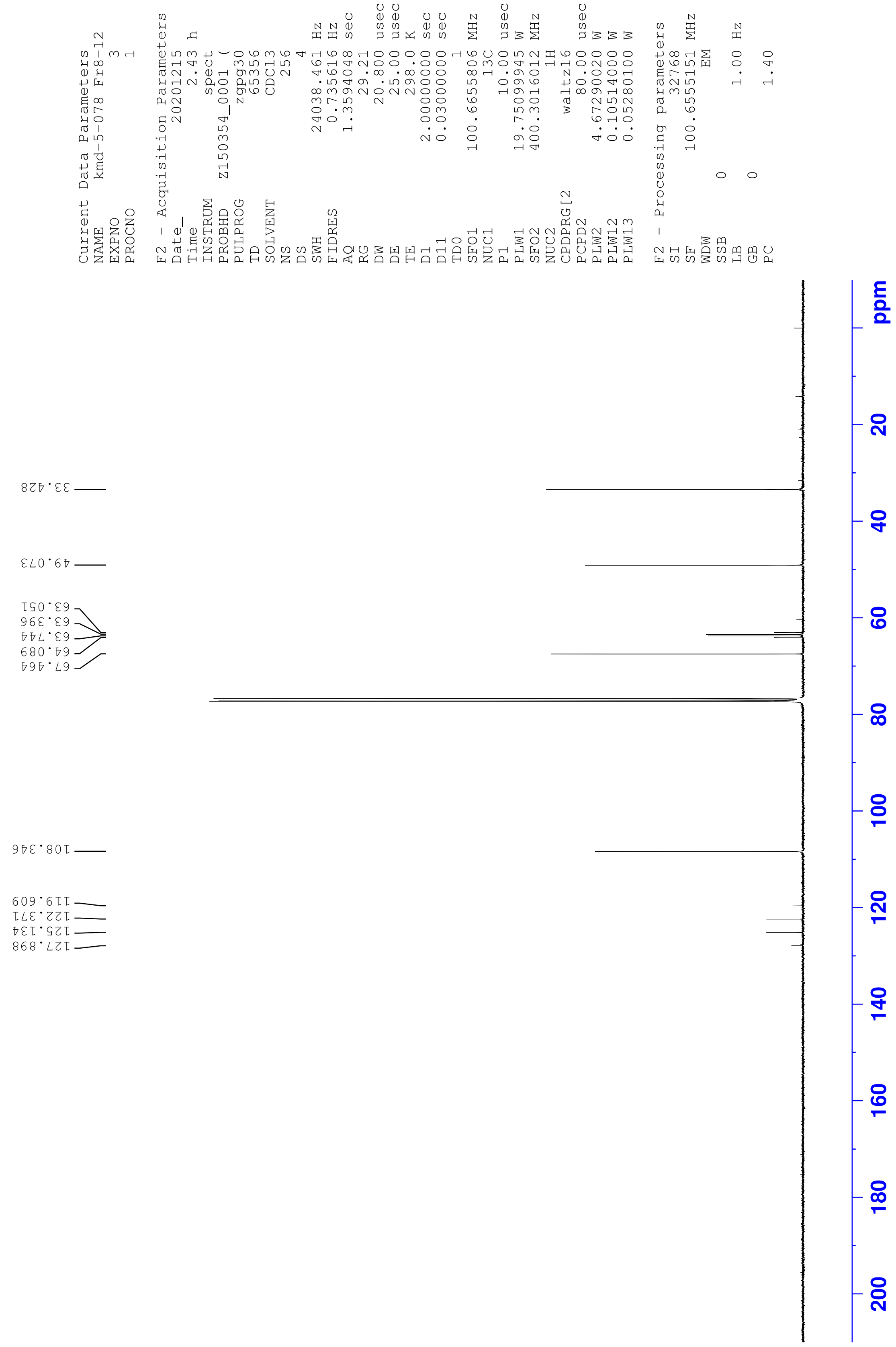
Current Data Parameters
NAME kmd-5-078 Fr8-12
EXPNO 3
PROCNO 1
F2 - Acquisition Parameters
Date_ 20201215
Time 2.43 h
INSTRUM spect
PROBHD Z150354_0001 (
PULPROG zgpg30
TD 65356
SOLVENT CDCl3
NS 256
DS 4
SWH 24038.461 Hz
FIDRES 0.735616 Hz
AQ 1.3594048 sec
RG 29.21
DW 20.800 usec
DE 25.00 usec
TE 298.0 K
D1 2.00000000 sec
D11 0.03000000 sec
TD0 1
SFO1 100.6655806 MHz
NUC1 13C
P1 10.00 usec
PLW1 19.75099945 W
SFO2 400.3016012 MHz
NUC2 1H
CPDPRG[2 waltz16
PCPD2 80.00 usec
PLW2 4.67290020 W
PLW12 0.10514000 W
PLW13 0.05280100 W
F2 - Processing parameters
SI 32768
SF 100.6555151 MHz
WDW EM
SSB 0
LB 1.00 Hz
GB 0
PC 1.40
33.428
49.073
63.051
63.396
63.744
64.089
67.464
108.346
119.609
122.371
125.134
127.898
ppm
20
40
60
80
100
120
140
160
180
200

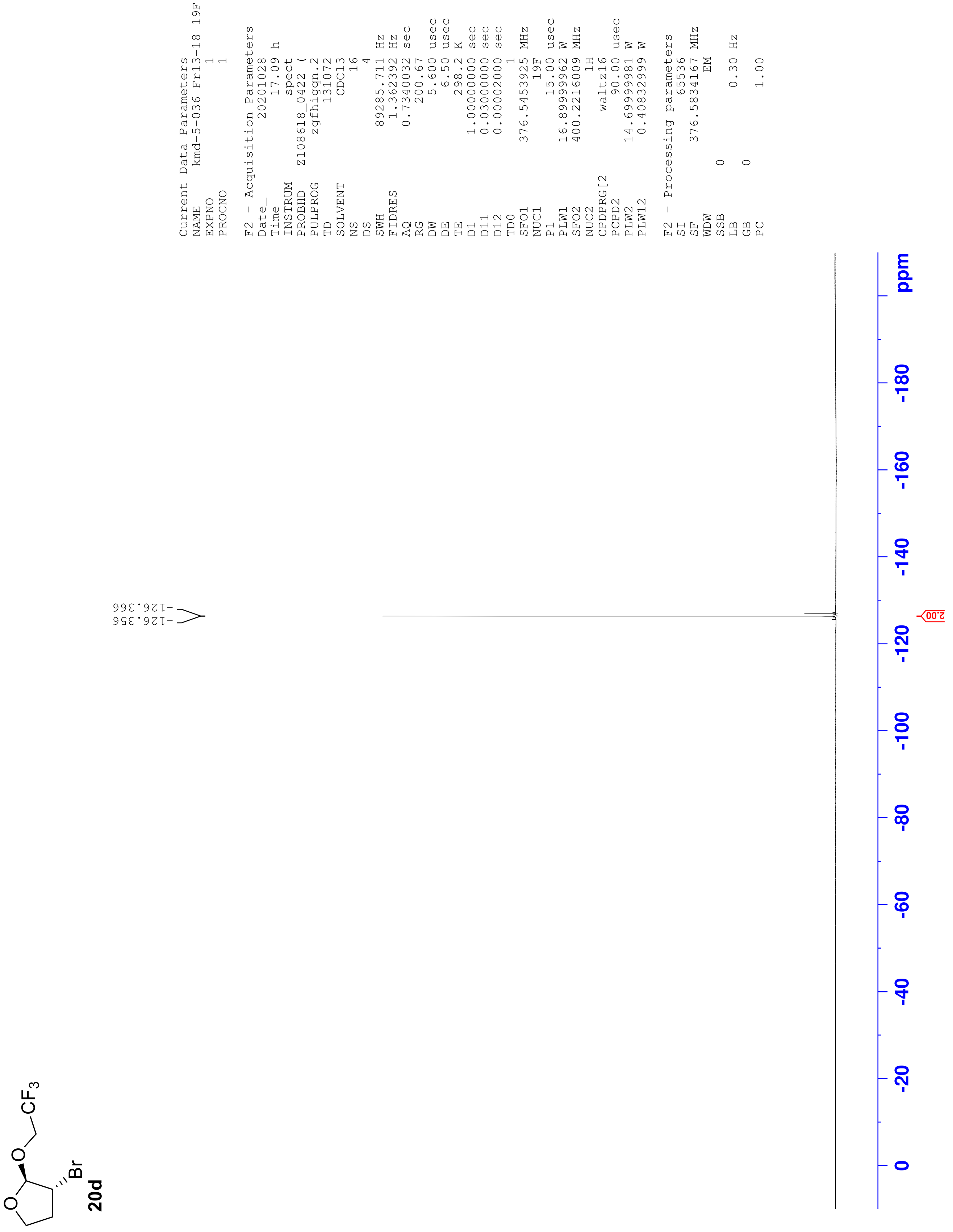
Current Data Parameters
NAME kmd-5-036 Fr13-18 19F
EXPNO 1
PROCNO 1
F2 - Acquisition Parameters
Date_ 20201028
Time 17.09 h
INSTRUM spect
PROBHD Z108618_0422 (
PULPROG zgfhigqn.2
TD 131072
SOLVENT CDCl3
NS 16
DS 4
SWH 89285.711 Hz
FIDRES 1.362392 Hz
AQ 0.7340032 sec
RG 200.67
DW 5.600 usec
DE 6.50 usec
TE 298.2 K
D1 1.00000000 sec
D11 0.03000000 sec
D12 0.00002000 sec
TD0 1
SFO1 376.5453925 MHz
NUC1 19F
P1 15.00 usec
PLW1 16.89999962 W
SFO2 400.2216009 MHz
NUC2 1H
CPDPRG[2 waltz16
PCPD2 90.00 usec
PLW2 14.69999981 W
PLW12 0.40832999 W
F2 - Processing parameters
SI 65536
SF 376.5834167 MHz
WDW EM
SSB 0
LB 0.30 Hz
GB 0
PC 1.00
-126.356
-126.366
2.00
ppm
-180
-160
-140
-120
-100
-80
-60
-40
-20
0
CF3
O
O
Br
20d

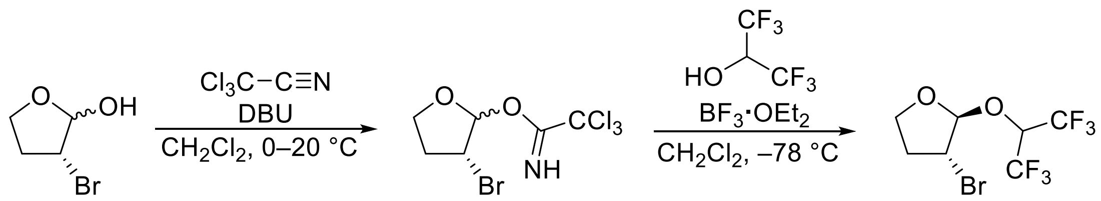

16 **18** **20e**

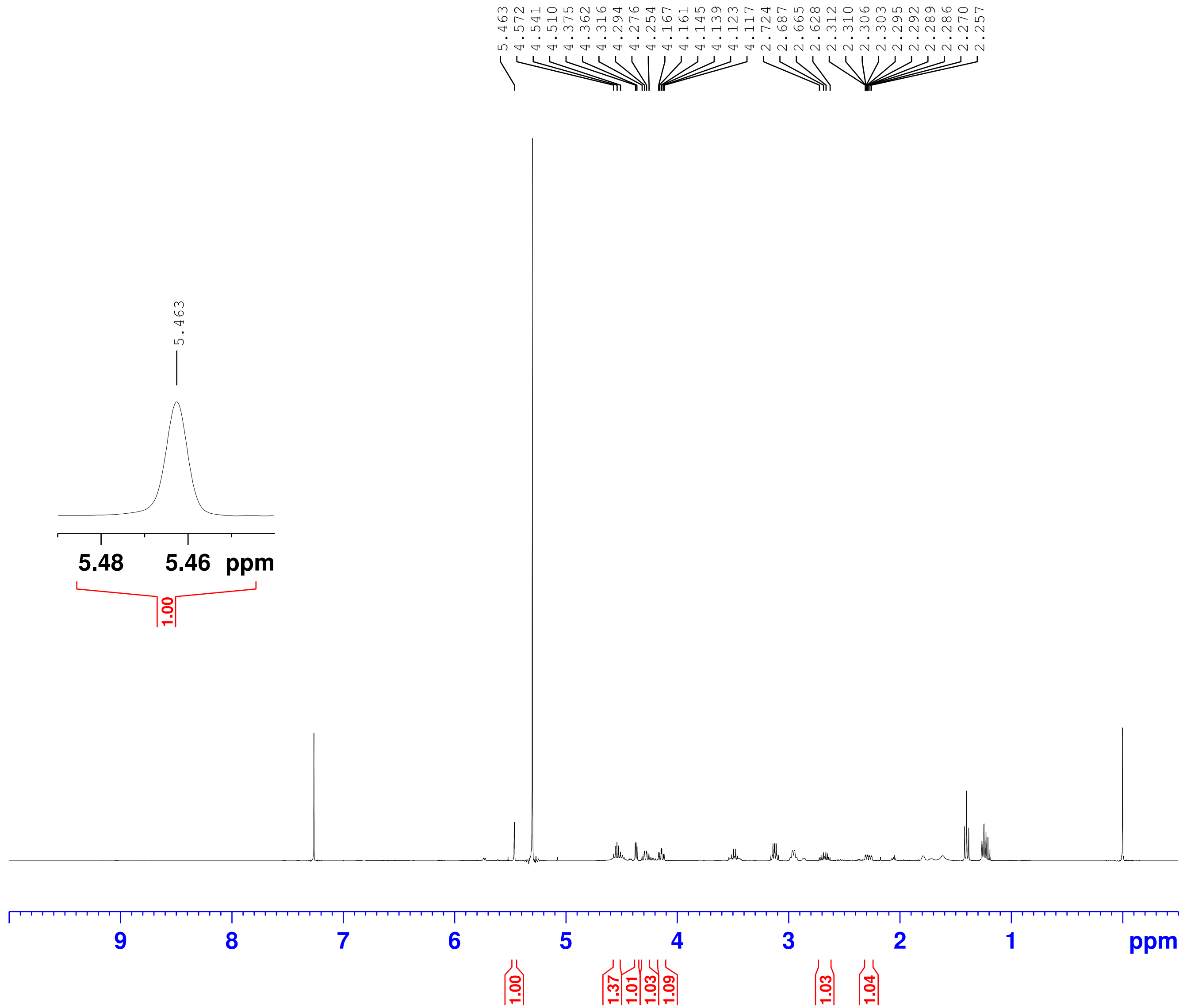


```
Current Data Parameters
NAME              kmd-5-038
EXPNO                     2
PROCNO                    1

F2 - Acquisition Parameters
Date_              20201027
Time                  18.06 h
INSTRUM               spect
PROBHD    Z150354_0001 (
PULPROG                zg30
TD                    65536
SOLVENT               CDCl3
NS                        4
DS                        0
SWH                8012.820 Hz
FIDRES             0.244532 Hz
AQ                4.0894465 sec
RG                    164.8
DW                   62.400 usec
DE                    30.00 usec
TE                    298.0 K
D1              30.00000000 sec
TD0                       1
SFO1            400.3024719 MHz
NUC1                     1H
P1                    12.00 usec
PLW1             4.67290020 W

F2 - Processing parameters
SI                    65536
SF              400.3000083 MHz
WDW                      EM
SSB       0
LB                     0.30 Hz
GB        0
PC                     1.00
```

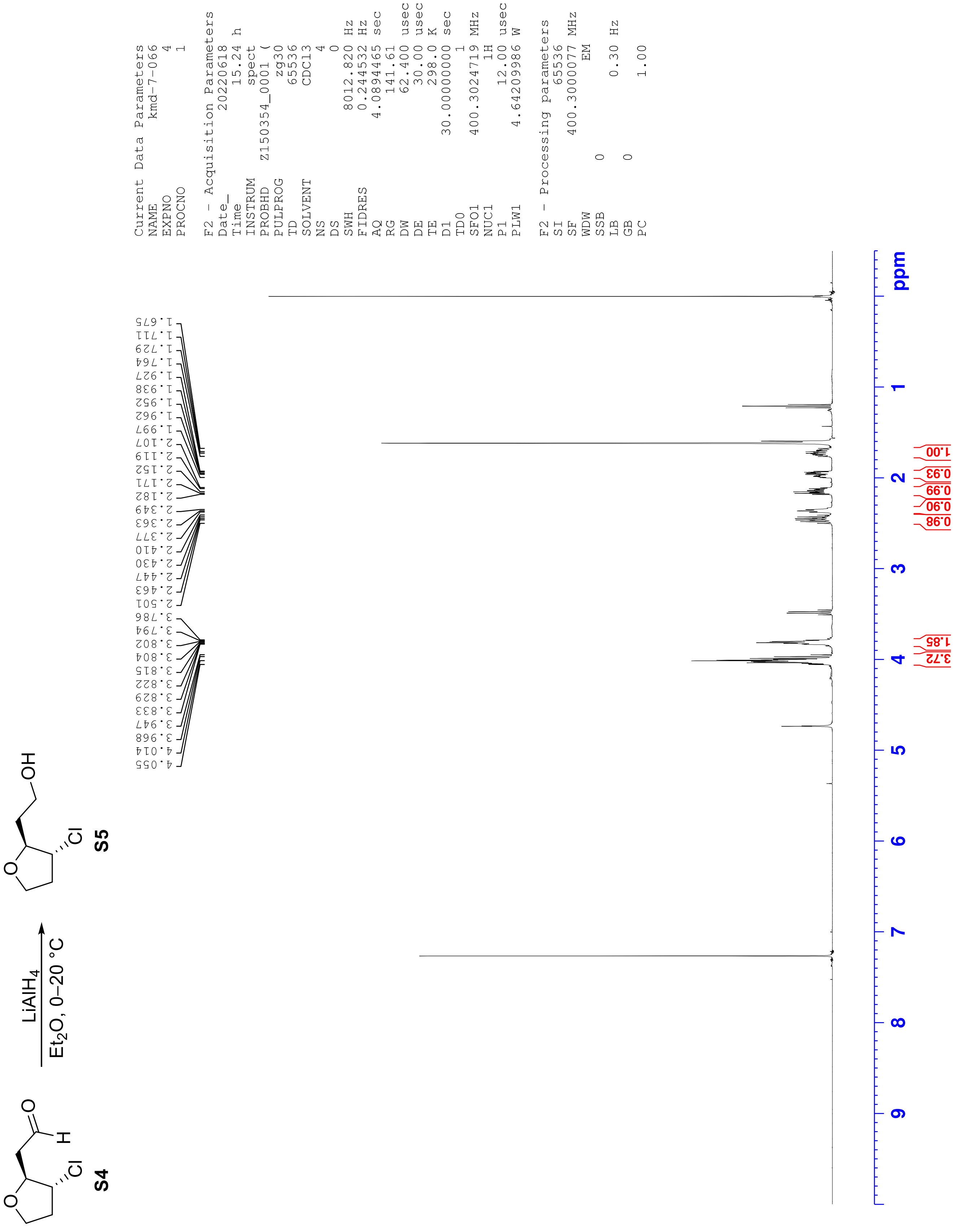
S4
LiAlH4
Et2O, 0–20 °C
S5
OH
Cl
H
O
1.675
1.711
1.729
1.764
1.927
1.938
1.952
1.962
1.997
2.107
2.119
2.152
2.171
2.182
2.349
2.363
2.377
2.410
2.430
2.447
2.463
2.501
3.786
3.794
3.802
3.804
3.815
3.822
3.829
3.833
3.947
3.968
4.014
4.055
Current Data Parameters
NAME kmd-7-066
EXPNO 4
PROCNO 1
F2 - Acquisition Parameters
Date_ 20220618
Time 15.24 h
INSTRUM spect
PROBHD Z150354_0001 (
PULPROG zg30
TD 65536
SOLVENT CDCl3
NS 4
DS 0
SWH 8012.820 Hz
FIDRES 0.244532 Hz
AQ 4.0894465 sec
RG 141.61
DW 62.400 usec
DE 30.00 usec
TE 298.0 K
D1 30.00000000 sec
TD0 1
SFO1 400.3024719 MHz
NUC1 1H
P1 12.00 usec
PLW1 4.64209986 W
F2 - Processing parameters
SI 65536
SF 400.3000077 MHz
WDW EM
SSB 0
LB 0.30 Hz
GB 0
PC 1.00
1.00
0.93
0.99
0.90
0.98
1.85
3.72
ppm
1
2
3
4
5
6
7
8
9

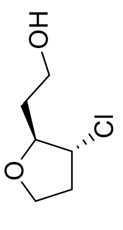

S5

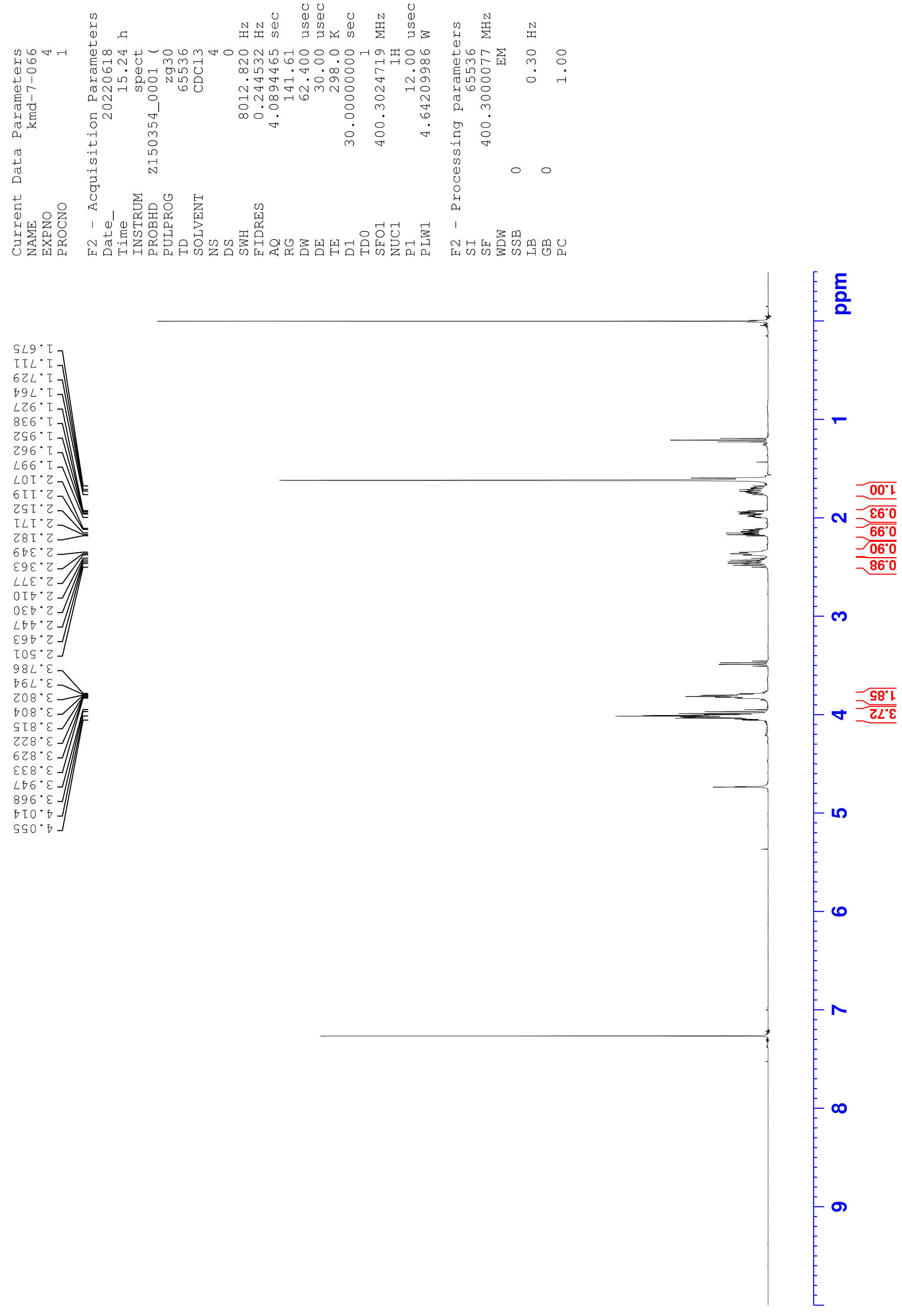
Current Data Parameters
NAME kmd-7-066
EXPNO 4
PROCNO 1
F2 - Acquisition Parameters
Date_ 20220618
Time 15.24 h
INSTRUM spect
PROBHD Z150354_0001 (
PULPROG zg30
TD 65536
SOLVENT CDCl3
NS 4
DS 0
SWH 8012.820 Hz
FIDRES 0.244532 Hz
AQ 4.0894465 sec
RG 141.61
DW 62.400 usec
DE 30.00 usec
TE 298.0 K
D1 30.00000000 sec
TD0 1
SFO1 400.3024719 MHz
NUC1 1H
P1 12.00 usec
PLW1 4.64209986 W
F2 - Processing parameters
SI 65536
SF 400.3000077 MHz
WDW EM
SSB 0
LB 0.30 Hz
GB 0
PC 1.00
1.675
1.711
1.729
1.764
1.927
1.938
1.952
1.962
1.997
2.107
2.119
2.152
2.171
2.182
2.349
2.363
2.377
2.410
2.430
2.447
2.463
2.501
3.786
3.794
3.802
3.804
3.815
3.822
3.829
3.833
3.947
3.968
4.014
4.055
1.00
0.93
0.99
0.90
0.98
1.85
3.72
ppm
1
2
3
4
5
6
7
8
9

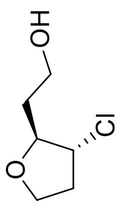

S5

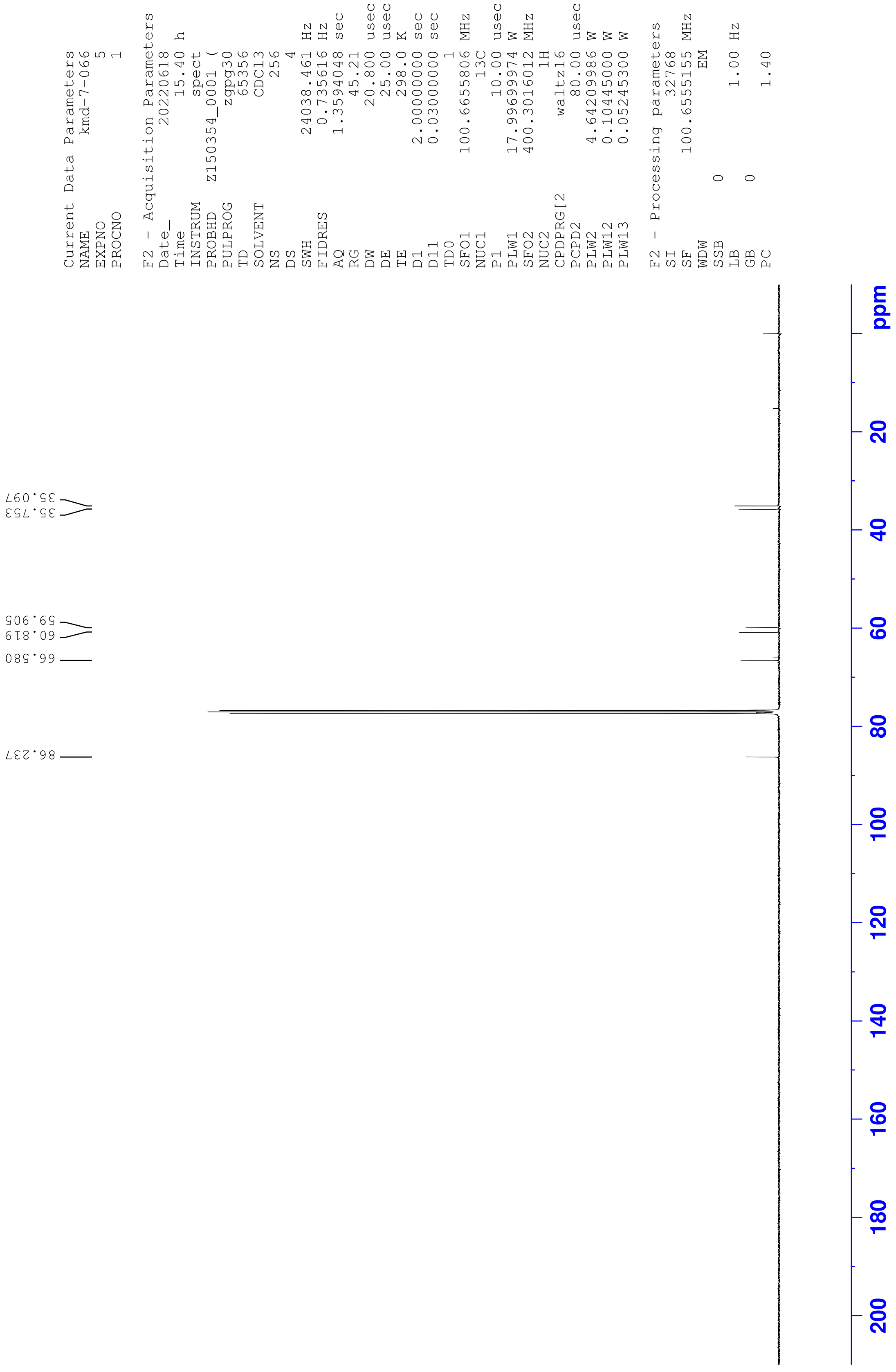
86.237
66.580
60.819
59.905
35.753
35.097
Current Data Parameters
NAME kmd-7-066
EXPNO 5
PROCNO 1
F2 - Acquisition Parameters
Date_ 20220618
Time 15.40 h
INSTRUM spect
PROBHD Z150354_0001 (
PULPROG zgpg30
TD 65356
SOLVENT CDCl3
NS 256
DS 4
SWH 24038.461 Hz
FIDRES 0.735616 Hz
AQ 1.3594048 sec
RG 45.21
DW 20.800 usec
DE 25.00 usec
TE 298.0 K
D1 2.00000000 sec
D11 0.03000000 sec
TD0 1
SFO1 100.6655806 MHz
NUC1 13C
P1 10.00 usec
PLW1 17.99699974 W
SFO2 400.3016012 MHz
NUC2 1H
CPDPRG[2 waltz16
PCPD2 80.00 usec
PLW2 4.64209986 W
PLW12 0.10445000 W
PLW13 0.05245300 W
F2 - Processing parameters
SI 32768
SF 100.6555155 MHz
WDW EM
SSB 0
LB 1.00 Hz
GB 0
PC 1.40
200
180
160
140
120
100
80
60
40
20
ppm

WILEY-VCH

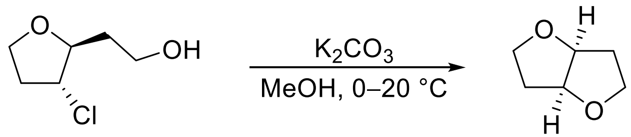

S5 **S6**

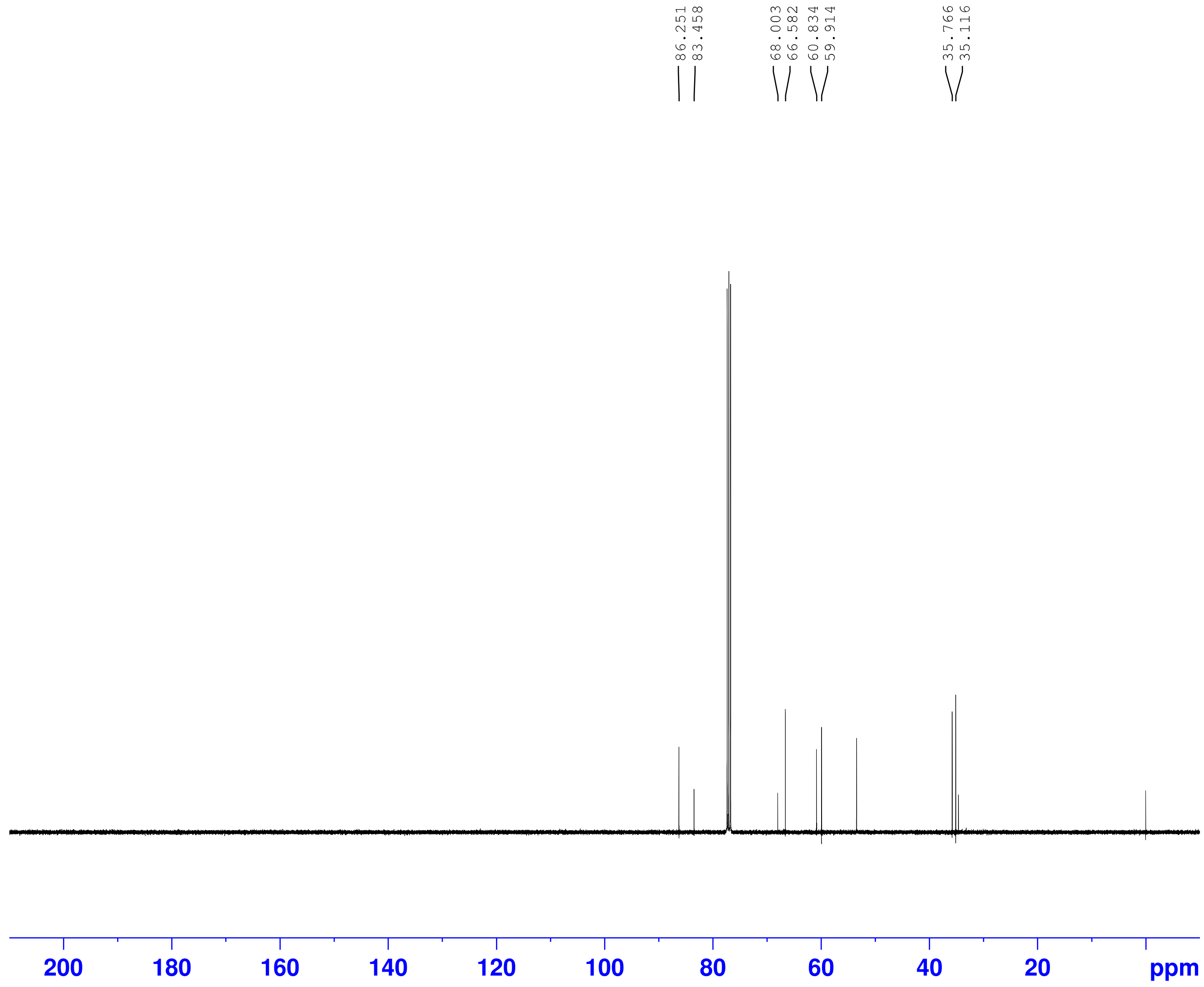


```
Current Data Parameters
NAME             kmd-7-070
EXPNO                    3
PROCNO                   1

F2 - Acquisition Parameters
Date_             20220620
Time                 15.29 h
INSTRUM              spect
PROBHD    Z150354_0001 (
PULPROG            zgpg30
TD                   65356
SOLVENT              CDCl3
NS                     256
DS                       4
SWH              24038.461 Hz
FIDRES            0.735616 Hz
AQ               1.3594048 sec
RG                    31.4
DW                  20.800 usec
DE                   25.00 usec
TE                   298.0 K
D1              2.00000000 sec
D11             0.03000000 sec
TD0                      1
SFO1          100.6655806 MHz
NUC1                   13C
P1                   10.00 usec
PLW1           17.99699974 W
SFO2          400.3016012 MHz
NUC2                    1H
CPDPRG[2           waltz16
PCPD2                80.00 usec
PLW2            4.64209986 W
PLW12           0.10445000 W
PLW13           0.05245300 W

F2 - Processing parameters
SI                  131072
SF            100.6555154 MHz
WDW                     EM
SSB      0
LB       0 Hz
GB       0
PC                    1.40
```

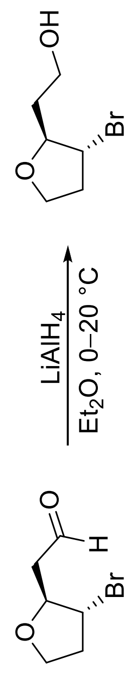
LiAlH$_4$
Et$_2$O, 0–20 °C
O
OH
Br
H

S7 S8

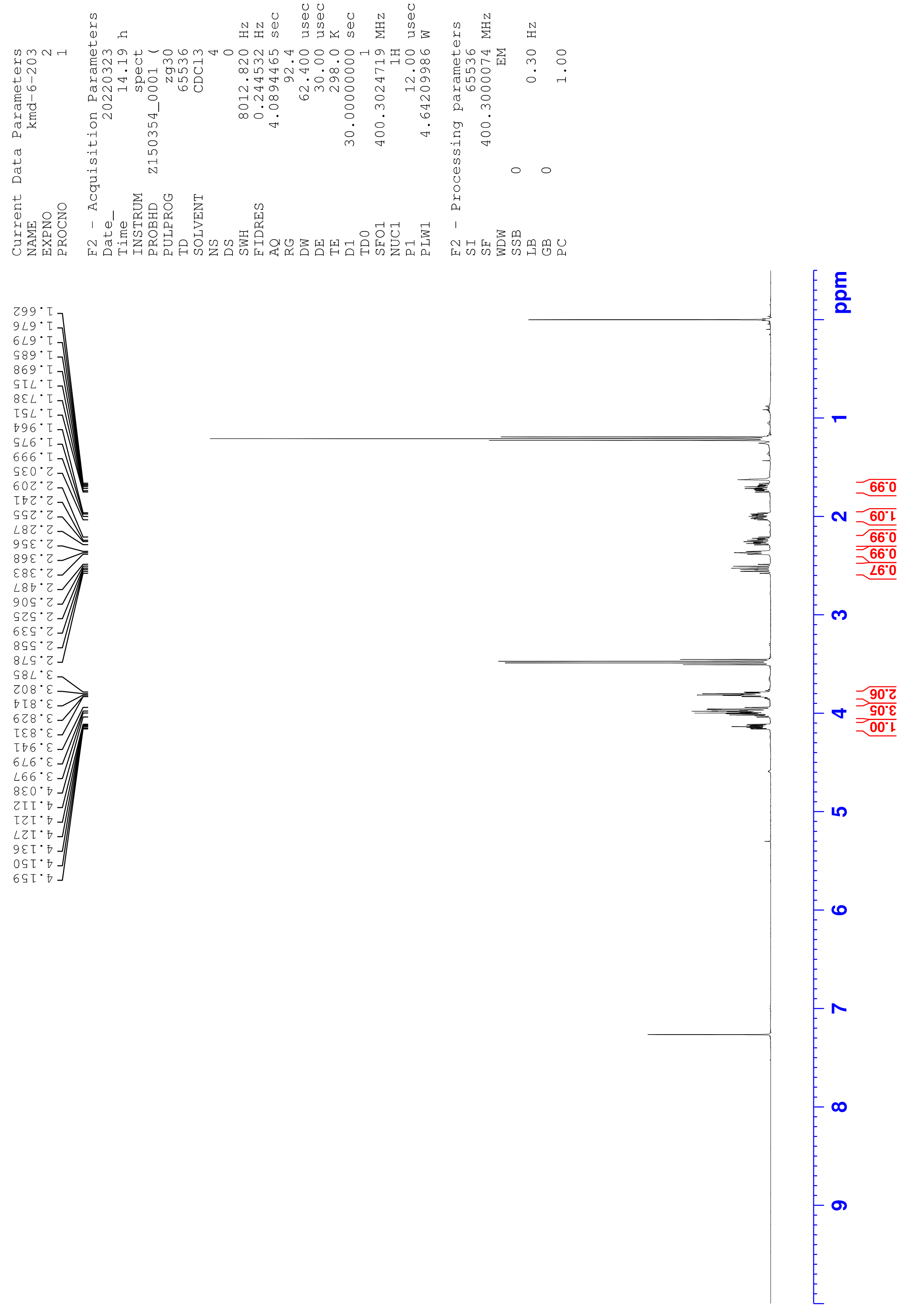
Current Data Parameters
NAME kmd-6-203
EXPNO 2
PROCNO 1
F2 - Acquisition Parameters
Date_ 20220323
Time 14.19 h
INSTRUM spect
PROBHD Z150354_0001 (
PULPROG zg30
TD 65536
SOLVENT CDCl3
NS 4
DS 0
SWH 8012.820 Hz
FIDRES 0.244532 Hz
AQ 4.0894465 sec
RG 92.4
DW 62.400 usec
DE 30.00 usec
TE 298.0 K
D1 30.00000000 sec
TD0 1
SFO1 400.3024719 MHz
NUC1 1H
P1 12.00 usec
PLW1 4.64209986 W
F2 - Processing parameters
SI 65536
SF 400.3000074 MHz
WDW EM
SSB 0
LB 0.30 Hz
GB 0
PC 1.00
4.159
4.150
4.136
4.127
4.121
4.112
4.038
3.997
3.979
3.941
3.831
3.829
3.814
3.802
3.785
2.578
2.558
2.539
2.525
2.506
2.487
2.383
2.368
2.356
2.287
2.255
2.241
2.209
2.035
1.999
1.975
1.964
1.751
1.738
1.715
1.698
1.685
1.679
1.676
1.662
1.00
3.05
2.06
0.97
0.99
0.99
1.09
0.99
ppm
9
8
7
6
5
4
3
2
1

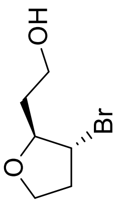

S8

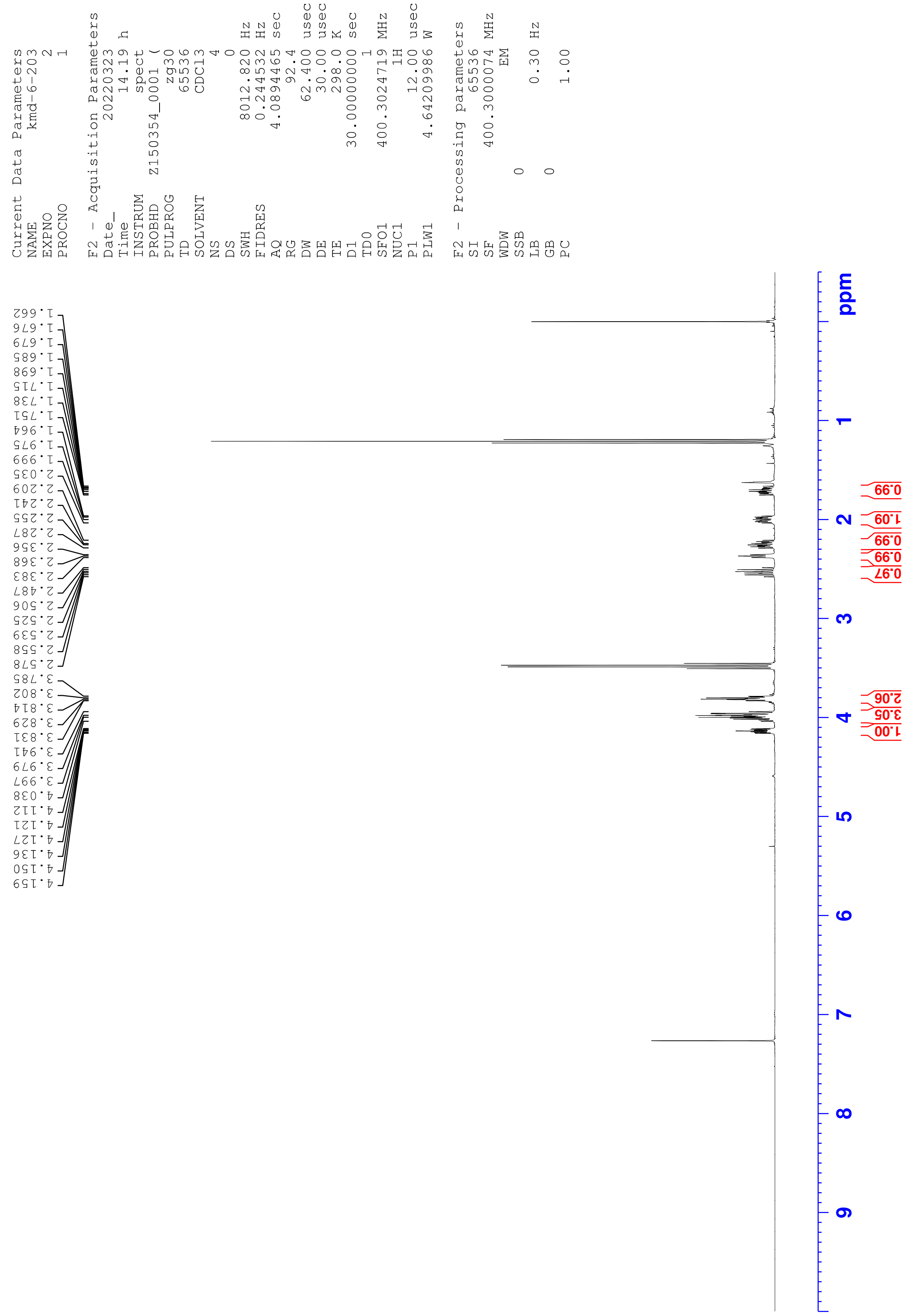
Current Data Parameters
NAME kmd-6-203
EXPNO 2
PROCNO 1
F2 - Acquisition Parameters
Date_ 20220323
Time 14.19 h
INSTRUM spect
PROBHD Z150354_0001 (
PULPROG zg30
TD 65536
SOLVENT CDCl3
NS 4
DS 0
SWH 8012.820 Hz
FIDRES 0.244532 Hz
AQ 4.0894465 sec
RG 92.4
DW 62.400 usec
DE 30.00 usec
TE 298.0 K
D1 30.00000000 sec
TD0 1
SFO1 400.3024719 MHz
NUC1 1H
P1 12.00 usec
PLW1 4.64209986 W
F2 - Processing parameters
SI 65536
SF 400.3000074 MHz
WDW EM
SSB 0
LB 0.30 Hz
GB 0
PC 1.00
1.662 1.676 1.679 1.685 1.698 1.715 1.738 1.751 1.964 1.975 1.999 2.035 2.209 2.241 2.255 2.287 2.356 2.368 2.383 2.487 2.506 2.525 2.539 2.558 2.578 3.785 3.802 3.814 3.829 3.831 3.941 3.979 3.997 4.038 4.112 4.121 4.127 4.136 4.150 4.159
0.99 1.09 0.99 0.99 0.97 2.06 3.05 1.00
ppm

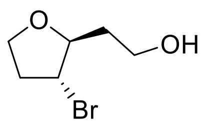

S8

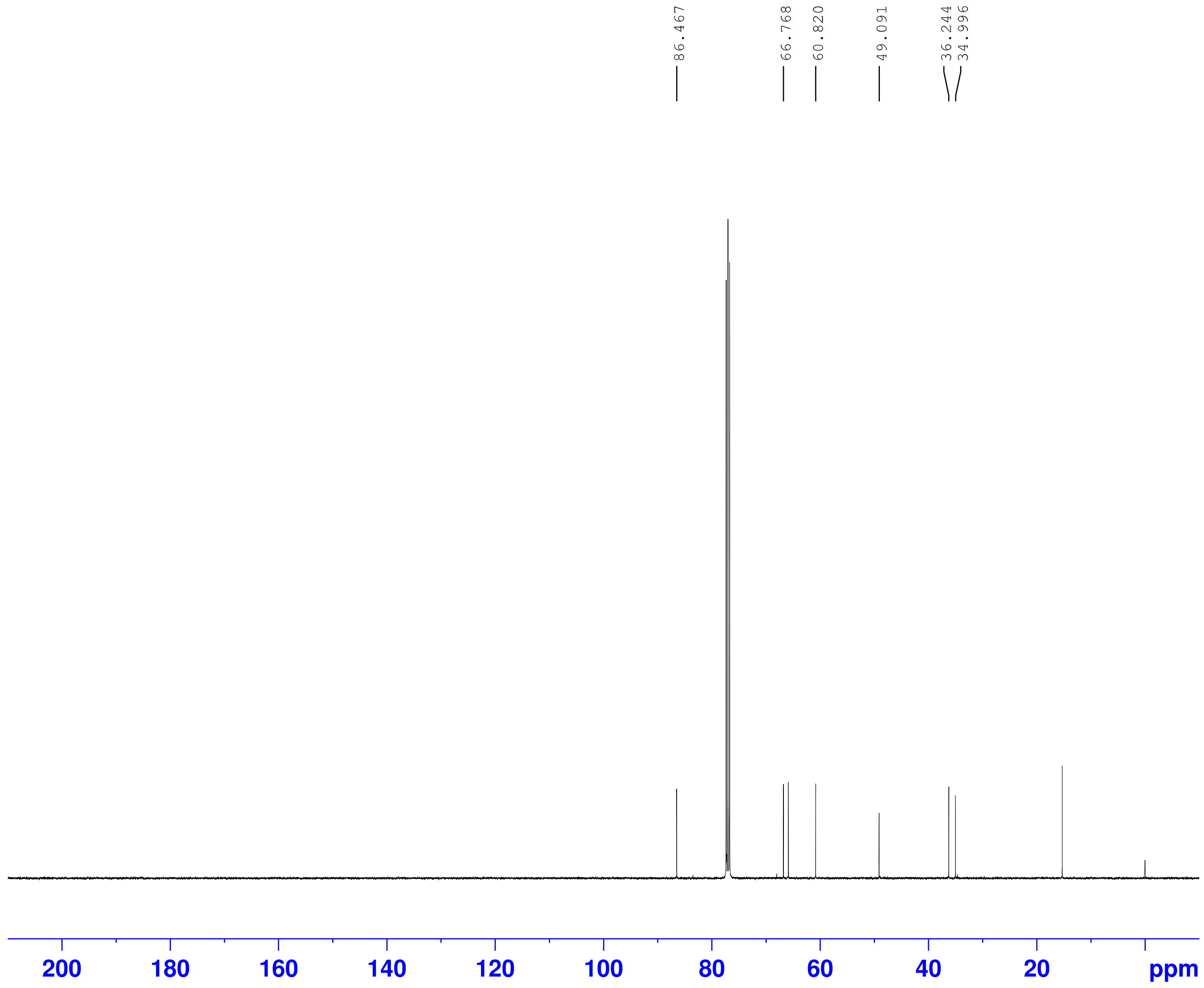


```
Current Data Parameters
NAME                kmd-6-203
EXPNO                       3
PROCNO                      1

F2 - Acquisition Parameters
Date_              20220323
Time                  19.23 h
INSTRUM               spect
PROBHD   Z150354_0001 (
PULPROG             zgpg30
TD                   65356
SOLVENT              CDCl3
NS                     256
DS                       4
SWH              24038.461 Hz
FIDRES            0.735616 Hz
AQ               1.3594048 sec
RG                   25.77
DW                  20.800 usec
DE                   25.00 usec
TE                   298.0 K
D1              2.00000000 sec
D11             0.03000000 sec
TD0                      1
SFO1           100.6655806 MHz
NUC1                   13C
P1                   10.00 usec
PLW1           17.99699974 W
SFO2           400.3016012 MHz
NUC2                    1H
CPDPRG[2           waltz16
PCPD2                80.00 usec
PLW2            4.64209986 W
PLW12           0.10445000 W
PLW13           0.05245300 W

F2 - Processing parameters
SI                   32768
SF             100.6555153 MHz
WDW                     EM
SSB      0
LB                    1.00 Hz
GB       0
PC                    1.40
```

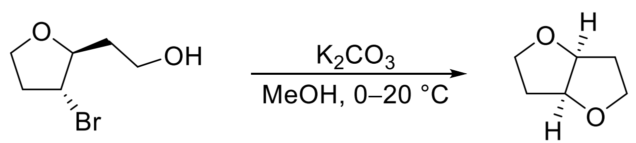

S8 **S6**

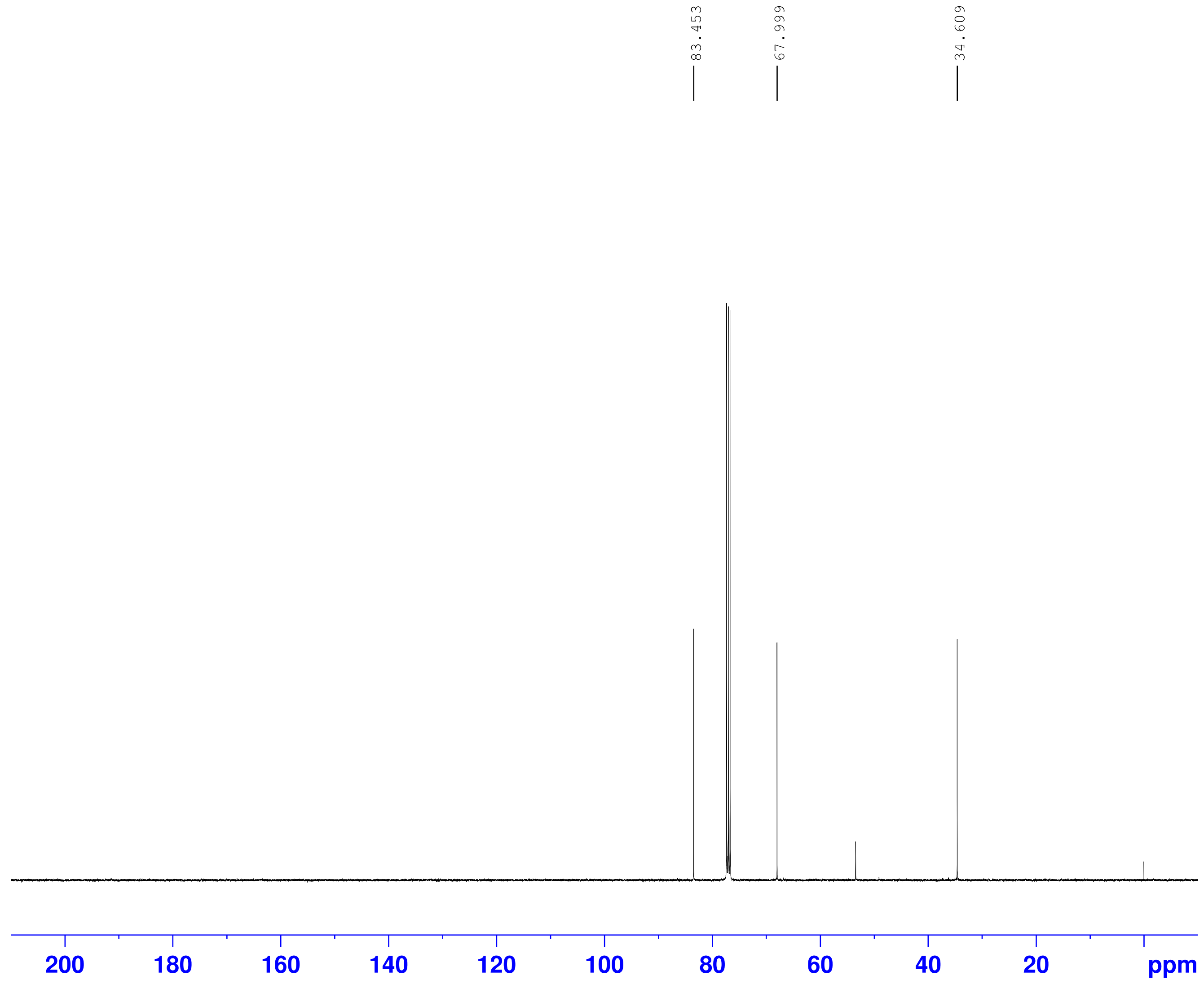


```
Current Data Parameters
NAME             kmd-7-071
EXPNO                    3
PROCNO                   1

F2 - Acquisition Parameters
Date_             20220620
Time                 14.18 h
INSTRUM              spect
PROBHD   Z150354_0001 (
PULPROG            zgpg30
TD                   65356
SOLVENT              CDCl3
NS                     256
DS                       4
SWH              24038.461 Hz
FIDRES            0.735616 Hz
AQ               1.3594048 sec
RG                   29.21
DW                  20.800 usec
DE                   25.00 usec
TE                   298.0 K
D1              2.00000000 sec
D11             0.03000000 sec
TD0                      1
SFO1           100.6655806 MHz
NUC1                   13C
P1                   10.00 usec
PLW1           17.99699974 W
SFO2           400.3016012 MHz
NUC2                    1H
CPDPRG[2           waltz16
PCPD2                80.00 usec
PLW2            4.64209986 W
PLW12           0.10445000 W
PLW13           0.05245300 W

F2 - Processing parameters
SI                   32768
SF             100.6555150 MHz
WDW                     EM
SSB      0
LB                    1.00 Hz
GB       0
PC                    1.40
```

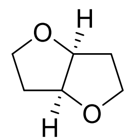

S6

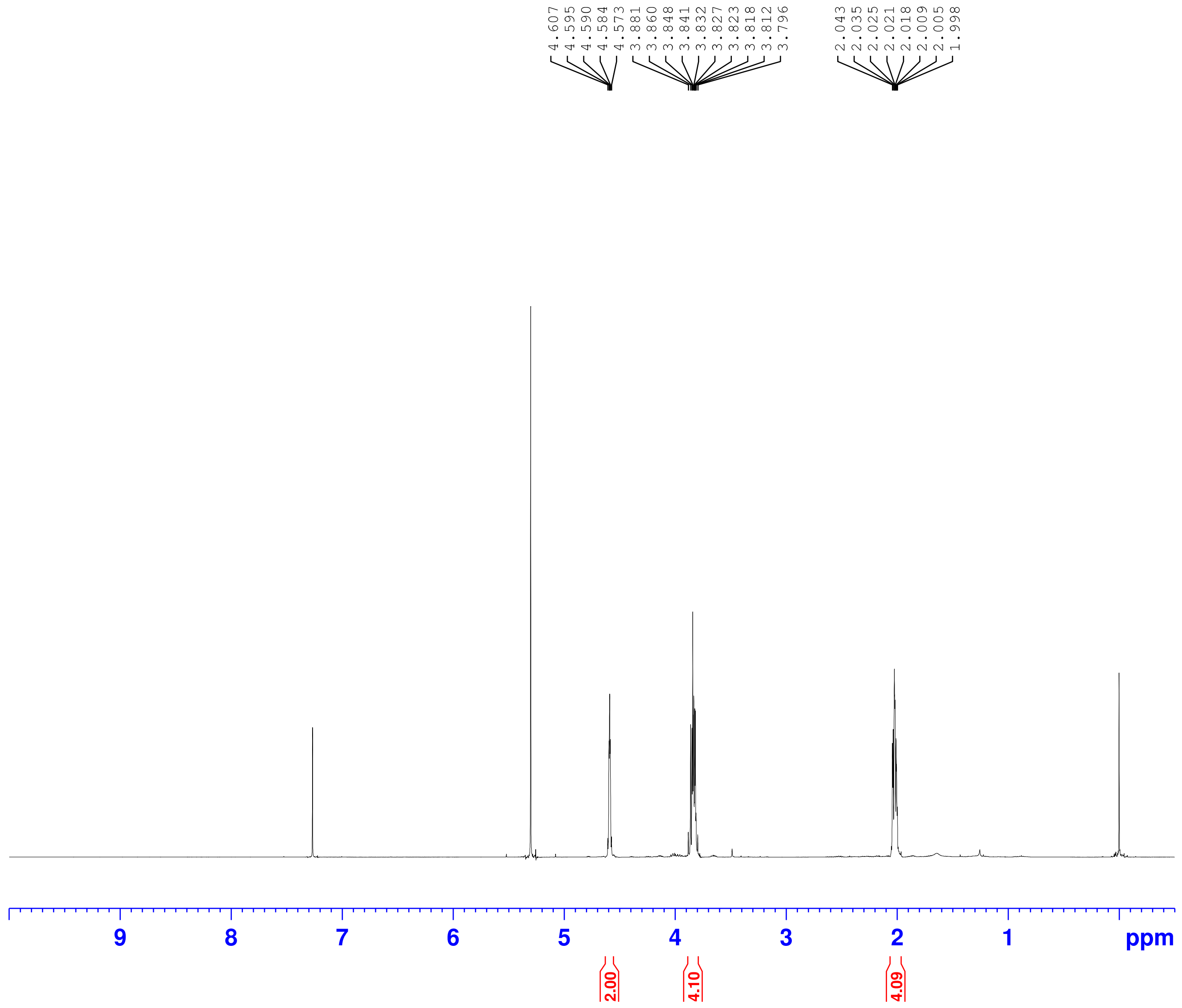


```
Current Data Parameters
NAME             kmd-7-071
EXPNO                    2
PROCNO                   1

F2 - Acquisition Parameters
Date_             20220620
Time                 12.01 h
INSTRUM              spect
PROBHD    Z150354_0001 (
PULPROG               zg30
TD                   65536
SOLVENT              CDCl3
NS                       4
DS                       0
SWH               8012.820 Hz
FIDRES            0.244532 Hz
AQ               4.0894465 sec
RG                    92.4
DW                  62.400 usec
DE                   30.00 usec
TE                   298.0 K
D1             30.00000000 sec
TD0                      1
SFO1           400.3024719 MHz
NUC1                    1H
P1                   12.00 usec
PLW1            4.64209986 W

F2 - Processing parameters
SI                   65536
SF             400.3000068 MHz
WDW                     EM
SSB       0
LB                    0.30 Hz
GB        0
PC                    1.00
```

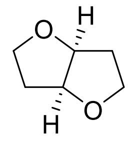

S6

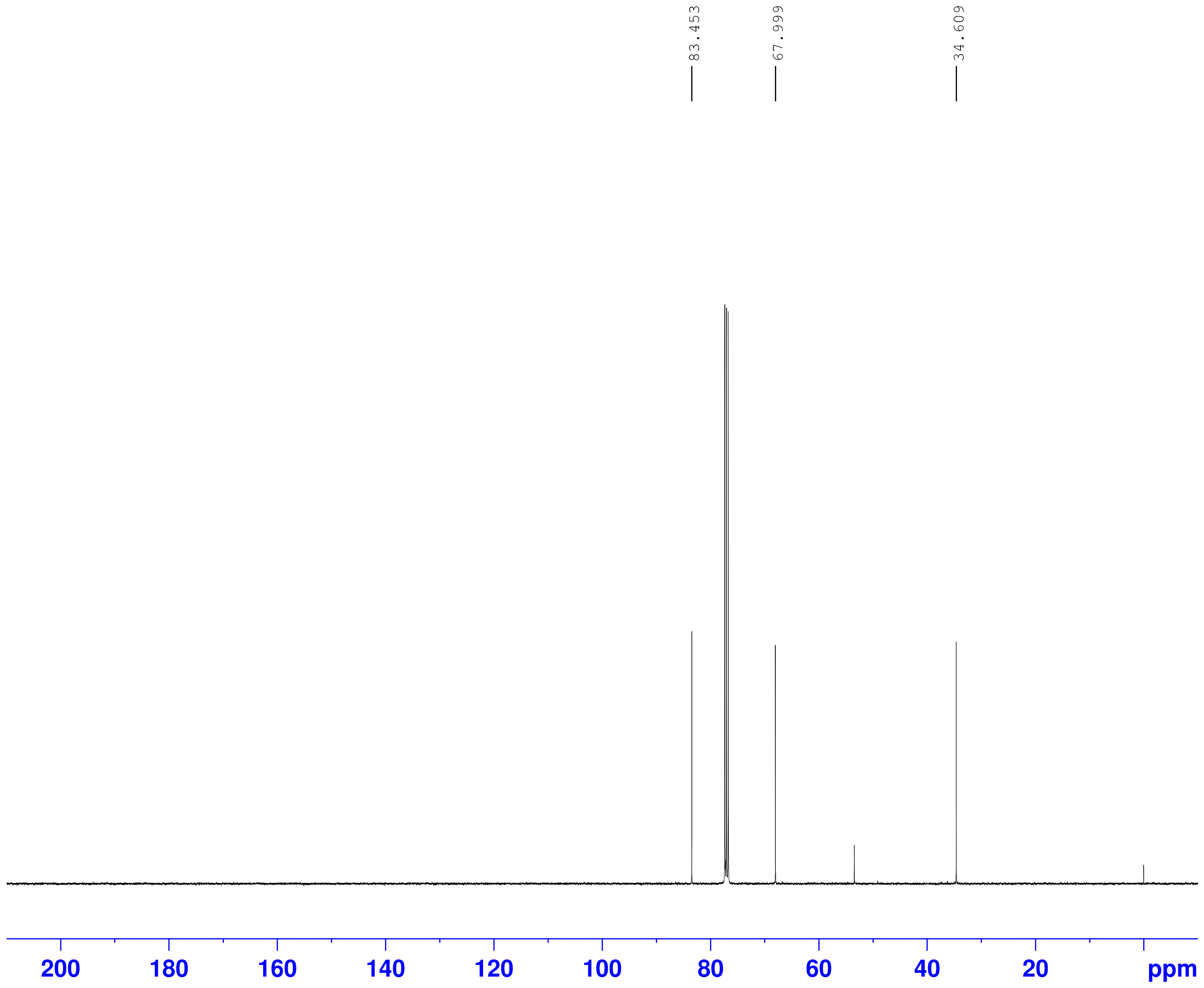

Current Data Parameters
NAME kmd-7-071
EXPNO 3
PROCNO 1

F2 - Acquisition Parameters
Date_ 20220620
Time 14.18 h
INSTRUM spect
PROBHD Z150354_0001 (
PULPROG zgpg30
TD 65356
SOLVENT CDCl3
NS 256
DS 4
SWH 24038.461 Hz
FIDRES 0.735616 Hz
AQ 1.3594048 sec
RG 29.21
DW 20.800 usec
DE 25.00 usec
TE 298.0 K
D1 2.00000000 sec
D11 0.03000000 sec
TD0 1
SFO1 100.6655806 MHz
NUC1 13C
P1 10.00 usec
PLW1 17.99699974 W
SFO2 400.3016012 MHz
NUC2 1H
CPDPRG[2 waltz16
PCPD2 80.00 usec
PLW2 4.64209986 W
PLW12 0.10445000 W
PLW13 0.05245300 W

F2 - Processing parameters
SI 32768
SF 100.6555150 MHz
WDW EM
SSB 0
LB 1.00 Hz
GB 0
PC 1.40
